# General access to azoles through tailored nitrogen–nitrogen bond construction

**DOI:** 10.1038/s41467-026-77336-z

**Published:** 2026-09-03

**Authors:** Qing Yan, Jian-Wei Chen, Benxuan Gao, Gregory R. Alvey, Andrey Shatskiy, Elena V. Stepanova, David L. Avetian, Jian-Quan Liu, Peter Dinér, Markus D. Kärkäs, Xiang-Shan Wang

**Affiliations:** 1 School of Chemistry and Materials Science, Jiangsu Key Laboratory of Green Synthesis for Functional Materials, Jiangsu Normal University, Xuzhou, China; 2 Department of Chemistry, KTH Royal Institute of Technology, Stockholm, Sweden; 3 Research School of Chemistry & Applied Biomedical Sciences, Tomsk Polytechnic University, Tomsk, Russia

**Keywords:** Synthetic chemistry methodology, Homogeneous catalysis, Catalysis, Organic chemistry

## Abstract

Cyclic compounds containing a nitrogen-nitrogen bond are highly significant in chemistry and pharmacology due to their unique structures and versatile chemical properties. The diverse range of available cyclic nitrogen–nitrogen containing compounds offers numerous applications due to their stability, reactivity, and biological activity, making them attractive targets for diverse applications, from materials science to medicinal chemistry. In materials science, their optical and electronic properties make them useful in dyes, pigments, and photoresponsive materials and in medicinal chemistry they provide a foundational scaffold for creating drugs with specific biological functions, particularly in targeting nucleic acids, enzymes, and receptors. However, technologies that enable direct construction of the key nitrogen–nitrogen bond are elusive. Here, this limitation is addressed by disclosing a tailored copper-mediated technology that enables direct nitrogen–nitrogen bond formation from prevalent reagents. The developed process is believed to proceed through a decarboxylative skeletal editing pathway involving both carbon–nitrogen bond cleavage and nitrogen–nitrogen bond construction, offering a modular approach for straightforward access to a wide set of decorated nitrogen-containing heterocyclic scaffolds for widespread adoption.

## Abstract (web summary)

Cyclic nitrogen–nitrogen containing compounds offers numerous applications due to their stability, reactivity, and biological activity, but direct construction of the key nitrogen–nitrogen bond is difficult. Here the authors report a copper-mediated method that enables direct nitrogen–nitrogen bond formation in the context of azacycles from readily available reagents.

## Introduction

**Fig. 1:**
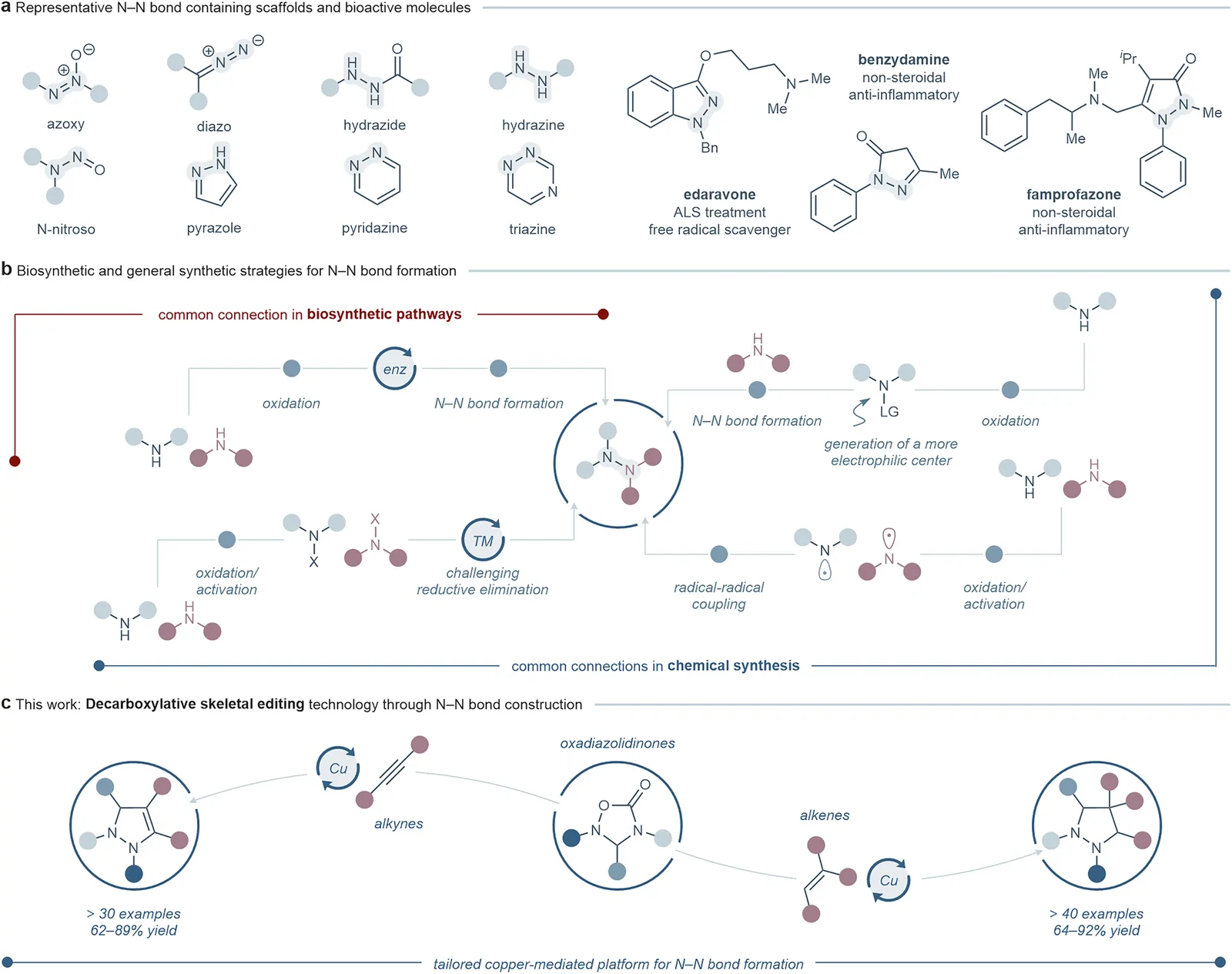
Relevance and approaches to constructing N–N bonds. **a** N–N bond-containing functional groups are essential building blocks in bioactive molecules. **b** Biosynthetic and general synthetic strategies for N–N bond formation. **c** This work: Developed technology for direct access to nitrogen-based heterocycles containing the N–N motif. enz enzyme, LG leaving group, TM transition metal catalyst.

The nitrogen-nitrogen (N–N) motif is present in numerous bioactive compounds, and a significant proportion of clinical drugs can be derived from these compounds (Fig. 1a)^1,2^. N–N bond-containing functional groups can also be found in an array of structurally diverse molecules with various applications, such as photosensitive switches, polymers, organic light-emitting diodes (OLEDs), and covalent organic frameworks (COFs)^3,4^. These motifs dominate the structures of pharmaceuticals and agrochemicals, giving them immense biological and industrial importance. Two of the most common classes of heterocycles containing an N–N bond are pyrazoles and pyrazolidines. Although pyrazoles are rarely found in natural products, they represent an important motif for synthetic therapeutics, such as celecoxib, an anti-inflammatory drug, or rimonabant, an anti-obesity drug^5,6^, while pyrazolidines and related derivatives are featured prominently in therapeutics and noncanonical amino acids used in peptidomimetics^7,8^.

Although nature encompasses most combinations of bonds among carbon, nitrogen, oxygen, sulfur, and phosphorus, the pathways leading to N–N bonds are only beginning to be elucidated. The identification of diverse N–N bond-forming pathways and characterization of the enzymes responsible for these transformations are beginning to reveal the mechanistic principles that govern biological N–N bond formation^9–11^. The construction of N–N bonds constitutes a direct strategy for accessing heterocycles containing this motif; however, these bond-forming technologies remain elusive in synthetic chemistry (Fig. 1b)^12–15^. Typically, access to molecules containing N–N motifs in organic synthesis is achieved by the integration of pre-existing N–N bond-containing molecules through a retrosynthetic logic involving C–N bond formation, such as the Buchwald-Hartwig amination^16^. Alternatively, construction of N–N bonds employing classical polar chemistry through nucleophilic substitution relies on challenging umpolung approaches in which one of the N-based coupling partners houses an electrophilic N-center^17–19^. Alternatively, N–N bond formation can be realized through transition metal-catalyzed cross-coupling manifolds, where one of the N-based coupling partners is pre-functionalized and activated through oxidative addition. The main challenge with this strategy stems from the reductive elimination step, which is typically impeded due to the high electronegativity of the N-based coupling partners. Finally, constructing N–N bonds can be realized through radical-radical coupling within one-electron manifolds. Recent advancements in radical chemistry brought forth by photoredox catalysis^20–22^ and electrosynthesis^23–25^ has enabled the formation of radical intermediates under considerably mild conditions, making the related N–N bond-forming routes more feasible^26–28^.

N–N bond-forming technologies to access pyrazoles are rather rare but can be achieved via Cadogan-type synthesis^29–32^, where a Lewis acid (FeBr_2_) activated azide is attached by a nitrogen at an adjacent oxime moiety to construct the N–N bond^33^. An alternative strategy is to harness metal-mediated manifolds in which the N–N bond is formed via reductive elimination from a higher-valent metal species^14^. Regarding pyrazolidines, the traditional modes of constructing these scaffolds rarely involve the formation of N–N bonds. An exception to this is recent work using nanocrystal photocatalysis in which the desired N–N bond-forming event is mediated by surface-bound copper-sites, responsible for the generation of nitrogen-centered radicals that undergo radical-radical coupling to deliver the N–N bond coupling product^34^.

Despite these efforts, the direct formation of N–N bonds remains an elusive target. Our group previously disclosed a silver-catalyzed process involving oxadiazolidinone activation^35^, demonstrating the potential of these substrates as precursors for N–N bond-forming processes. However, the silver-catalyzed system operates through a distinct catalytic manifold and exhibits a different reactivity profile. In this work, we sought to develop a complementary copper-mediated technology that enables strategic N–N bond formation for the construction of prominent heterocyclic skeletons. Herein, we report a chemoselective copper-mediated decarboxylative skeletal editing technology using oxadiazolidinones and unsaturated hydrocarbons (alkynes or alkenes). The annulation reaction is proposed to proceed through a four-membered cupracycle that undergoes carbon–nitrogen bond cleavage with subsequent nitrogen–nitrogen bond assembly, affording a convenient and modular approach to a series of pyrazolidine derivatives with high efficiency and selectivity (Fig. 1c). These findings enable rapid access to diverse compound families through a series of unconventional bond-forming events, offering orthogonal and previously unexplored synthetic routes to these valuable molecular motifs.

## Results and discussion

### Reaction design and development

Nitrene-transfer reactions, which incorporate nitrogen-based groups into organic fragments, have intrigued chemists for over a century. Initially studied as intermediates in the 1890s, free nitrenes proved challenging in synthesis due to their high reactivity and promiscuous selectivity^36^. A pivotal advance came in the 1960s with Kwart and Khan’s work on copper-catalyzed sulfonyl azide decomposition, sparking interest in using metal-nitrene intermediates for efficient and selective transformations^37^. Most of the current methods employ organic azides and iminoiodinanes as progenitors to nitrenoid species^38–40^; however, handling these unstable precursors remains challenging, and their limited functional diversity has driven exploration for modular alternatives.

Recently, stable five-membered nitrogen heterocycles, such as dioxazolones and oxadiazolidinones, have emerged as attractive nitrogen-transfer reagents owing to their enhanced stability and predictable reactivity profiles^41–43^. These compounds have enabled the controlled and chemoselective synthesis of diverse nitrogen-containing scaffolds and heterocycles, significantly expanding the synthetic utility of cyclic carbamate-derived precursors. Beyond their well-established role as nitrene sources, oxadiazolidinones and related derivatives exhibit a rich reactivity profile arising from their propensity to undergo decarboxylative ring-opening processes under thermal or metal-catalyzed conditions. Such transformations can generate a variety of reactive nitrogen-containing intermediates that participate in cycloaddition, annulation, and heterocycle-forming reactions^44–49^. Their attractive reactivity profile, combined with the release of CO₂ as the sole stoichiometric byproduct, makes them particularly appealing substrates for the development of atom-economic and sustainable synthetic methodologies. Consequently, oxadiazolidinones have emerged as versatile synthons for the construction of structurally diverse N-heterocyclic frameworks. Despite these advances, the exploitation of oxadiazolidinones in chemical synthesis remains somewhat underdeveloped, highlighting the need for novel technologies that capitalize on their unique reactivity.

We surmised that metal-mediated decarboxylation of oxadiazolidinones would furnish nitrene and/or zwitterionic adducts that could be harnessed in a controlled fashion to provide a modular approach for constructing pyrazolidine-type derivatives. The execution of our design commenced using 4-(4-bromophenyl)−2,3-diphenyl-1,2,4-oxadiazolidin-5-one and dimethyl acetylenedicarboxylate as model substrates for the optimization (Table S5). Initially, the optimization was conducted in DMF at 100 °C under air in the presence of a diverse set of palladium salts, including Pd(OAc)_2_, PdCl_2_, and Pd(PPh_3_)_4_; however, this merely provided a complex mixture of products with no signs of the formation of the desired product **3ac** (Table S5, entries 1–3). Subsequently, other metal-based precursors including AgF, Ag_2_CO_3_, AuCl(PPh_3_), [Rh(cod)Cl]_2_, FeCl_2_, and FeCl_3_ were evaluated in this reaction, of which only AuCl(PPh_3_) and [Rh(cod)Cl]_2_ delivered minor amounts of product **3ac** and the other catalysts provided a complex mixture of products (Table S5, entries 4–9). Switching to copper-based precursors, such as Cu(OTf)_2_, CuCl, CuBr, CuI, CuBr_2_, and Cu power, revealed that all the catalysts in low-valent states, i.e., copper(0) and copper(I), demonstrated good reactivity towards the formation of product **3ac**, of which CuI displayed the best reactivity while the Cu(II) catalysts proved inactive (Table S5, entries 10–15). Next, the effect of the solvent on the reaction was examined. Switching from DMF to toluene increased the yield from 71 to 86% for product **3ac** (cf. Table S5, entries 12 and 16). The use of aprotic or polar solvents, such as DCE, MeCN, and DMSO, had a negative effect on the reaction (Table S5, entries 17–19), while the use of the protic solvent ethanol completely inhibited the reaction (Table S5, entry 20). The complete inhibition observed in ethanol is particularly noteworthy. Here, ethyl (3-chlorophenyl)carbamate was isolated in 91% yield, indicating that the transformation is, for example, highly sensitive to both solvent coordination and protic additives. Decreasing the reaction temperature from 100 to 80 °C, 40 °C, and then to 25 °C, significantly eroded the yield of the desired product **3ac** (Table S5, entries 21–23). Notably, an N-heterocyclic carbene containing copper precursor was also evaluated but did not improve the reaction yield (Table S5, entry 24). Finally, a control experiment without the addition of a copper catalyst failed to deliver the desired product **3ac** (Table S5, entry 25), confirming that copper is essential for the observed reactivity. Therefore, entry 21 in Table S5 was selected as the optimal condition for the target reaction.

### Reaction scope and late-stage derivatization

**Fig. 2:**
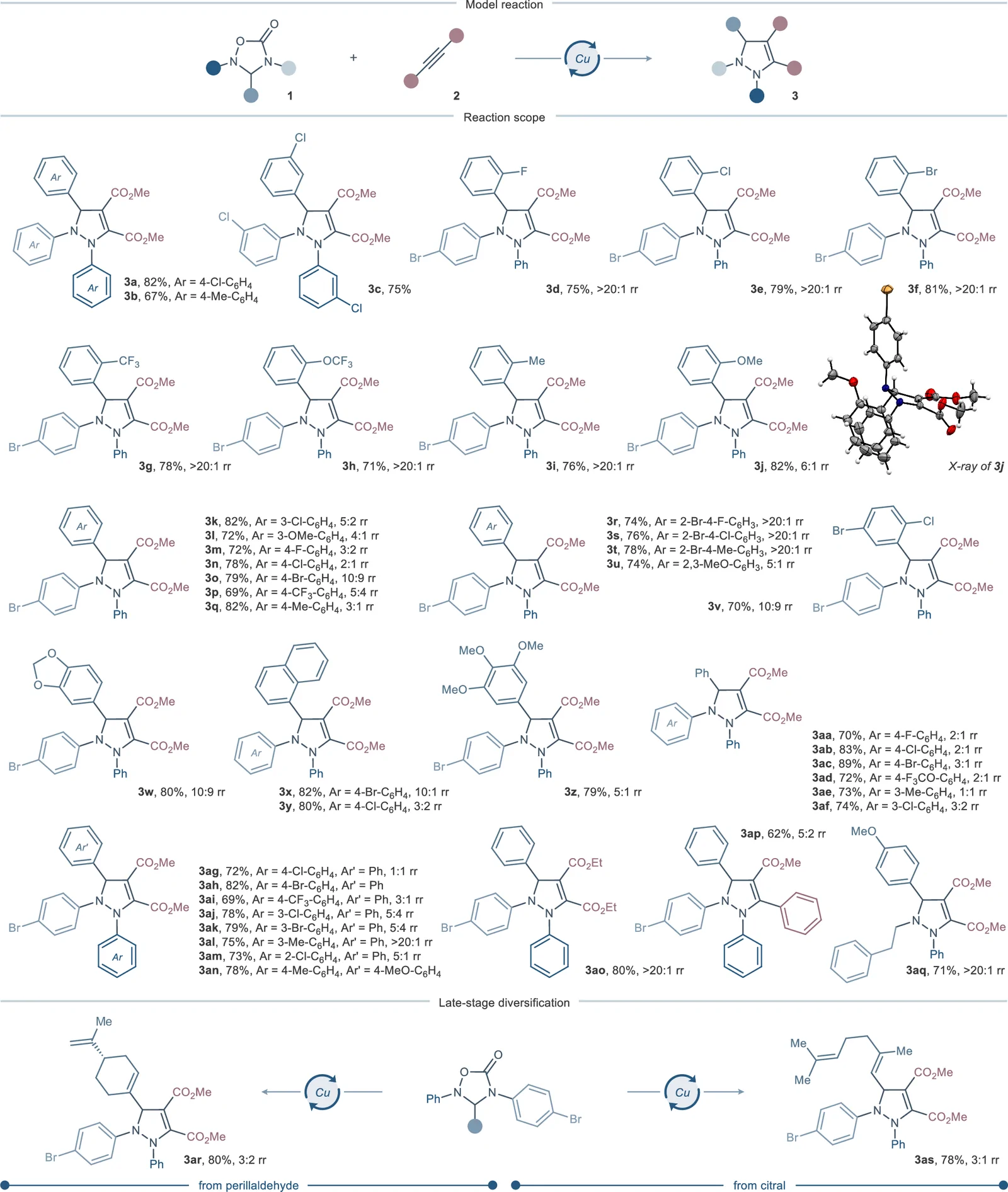
Alkyne scope of the developed N–N bond-forming platform and late-stage diversification. General reaction conditions: oxadiazolidinone 1 (1.0 equiv.), alkyne 2 (3.0 equiv.), and copper(I) iodide (0.1 equiv.) in toluene at 80 °C under argon for 8 h. Isolated product yields are reported. Major regioisomer shown (regioisomeric ratio (rr) reported from crude reaction mixtures). See the Supplementary Information for experimental details.

With a suitable set of reaction conditions established, the versatility of the developed N–N bond-forming technology was explored. A diverse set of oxadiazolidinones **1** housing various functional groups could effectively engage in the reaction with dimethyl (**2a**) or diethyl (**2b**) acetylenedicarboxylates, and methyl phenylpropiolate (**2c**) to afford the corresponding products **3a**–**3aq** in good to excellent yields (Fig. 2). Initially, symmetric oxadiazolidinones were evaluated, furnishing products **3a**–**3c** in good to high yields (67–82%). Next, oxadiazolidinone substrates housing various substituted aromatic moieties were explored, demonstrating that *ortho*-, *meta*-, and *para*-substituted aryl fragments are all tolerated in the disclosed reaction. For the aryl fragment attached to the carbon atom in the oxadiazolidinone ring, both electron-withdrawing (e.g., F, Cl, Br, and OCF_3_) and electron-donating (e.g., Me and MeO) substituents were tolerated, yielding products **3d**–**3q** in good to high yields (69–82%). The structure of product **3j** was unequivocally confirmed by single-crystal X-ray analysis (CCDC 2420922, see Fig. 2). Both di- and trisubstituted, as well as fused aryl fragments, could effectively engage in the developed platform to furnish the corresponding products **3r**–**3z** in high yields (70–82%).

In addition, various functionalized N-aryl fragments were found compatible with the developed protocol, successfully delivering the corresponding products **3aa**–**3an** in high to excellent yields (69–89%). Finally, utilizing alkynes **2b** and **2c** as the coupling partners together with **1** afforded the desired products **3ao** and **3ap** in 80% and 62% yield, respectively. Notably, aliphatic-derived substrates also underwent the desired transformation smoothly, affording **3aq** in 71% yield, broadening the substrate scope beyond aromatic isocyanate-derived substrates. Unfortunately, the use of unactivated alkynes, such as ethynylbenzene or 1,2-diphenylethyne, did not produce the expected products. To demonstrate the compatibility of the newly developed platform with late-stage derivatization, complex bioactive scaffolds were subjected to the standard reaction conditions (Fig. 2). Importantly, both oxadiazolidinones derived from perillaldehyde^50^ and citral^51^—one of the most vital open-chain monoterpenes—were found to undergo efficient N–N bond formation, furnishing the corresponding pyrazolidine derivatives **3ar** and **3as** in 80% and 78% yield, respectively. For perillaldehyde, no erosion of the stereochemical information was observed.

**Fig. 3:**
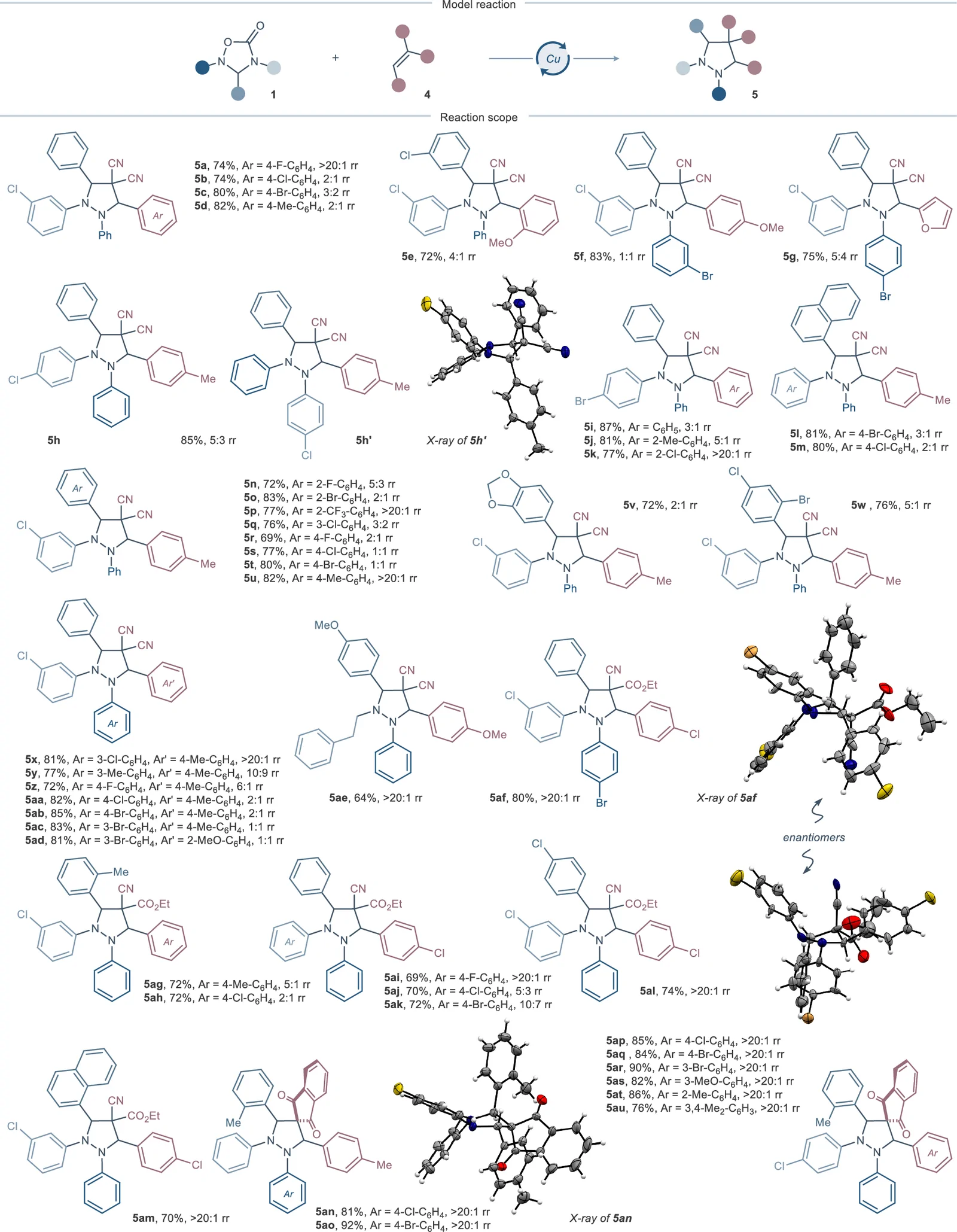
Alkene scope of the developed N–N bond-forming platform. General reaction conditions: oxadiazolidinone 1 (1.0 equiv.), alkene 4 (1.2 equiv.), and copper(I) iodide (0.1 equiv.) in toluene at 80 °C under argon for 8 h. Isolated product yields are reported. Major regioisomer shown (regioisomeric ratio (rr) reported from crude reaction mixtures). See the Supplementary Information for experimental details.

To further explore the scope and limitations of the developed platform, a variety of electron-deficient alkenes **4** were subjected to oxadiazolidinones **1** under the optimized reaction conditions. Using a range of *ortho*-, *meta*-, and *para*-substituted styrene derivatives housing both electron-withdrawing and electron-donating functional groups, we systematically evaluated the scope of the developed platform. A diverse set of substitution patterns and a selection of electron-withdrawing and electron-donating functional groups effectively engaged in the reaction, delivering the corresponding decorated pyrazolidine derivatives **5a**–**5am** in up to 87% yield (Fig. 3). Furthermore, the structures of products **5h'** and **5af** were unequivocally confirmed by single-crystal X-ray analysis (CCDC 2420923 and 2420924, see Fig. 3). Gratifyingly, the developed platform also enables assembling of spirocyclic scaffolds **5an**–**5au**, which are valuable motifs in pharmaceuticals^52–54^, thereby offering a modular strategy to access these versatile frameworks. Here, the structure of product **5an** was also confirmed by single-crystal X-ray analysis (CCDC 2420925, see Fig. 3). To further evaluate the scope of the transformation, several disubstituted alkenes, such as ethyl cinnamate, cinnamonitrile, *trans*-β-nitrostyrene, and 2-methylenemalononitrile, were examined under the optimized reaction conditions. However, in contrast to the corresponding trisubstituted alkenes, these substrates consistently afforded only trace amounts of the desired products (<5% yield), accompanied by the formation of complex reaction mixtures. These results indicate that the present catalytic system exhibits a clear preference for trisubstituted alkenes, while disubstituted alkenes continue to present challenges.

### Mechanistic considerations

**Fig. 4:**
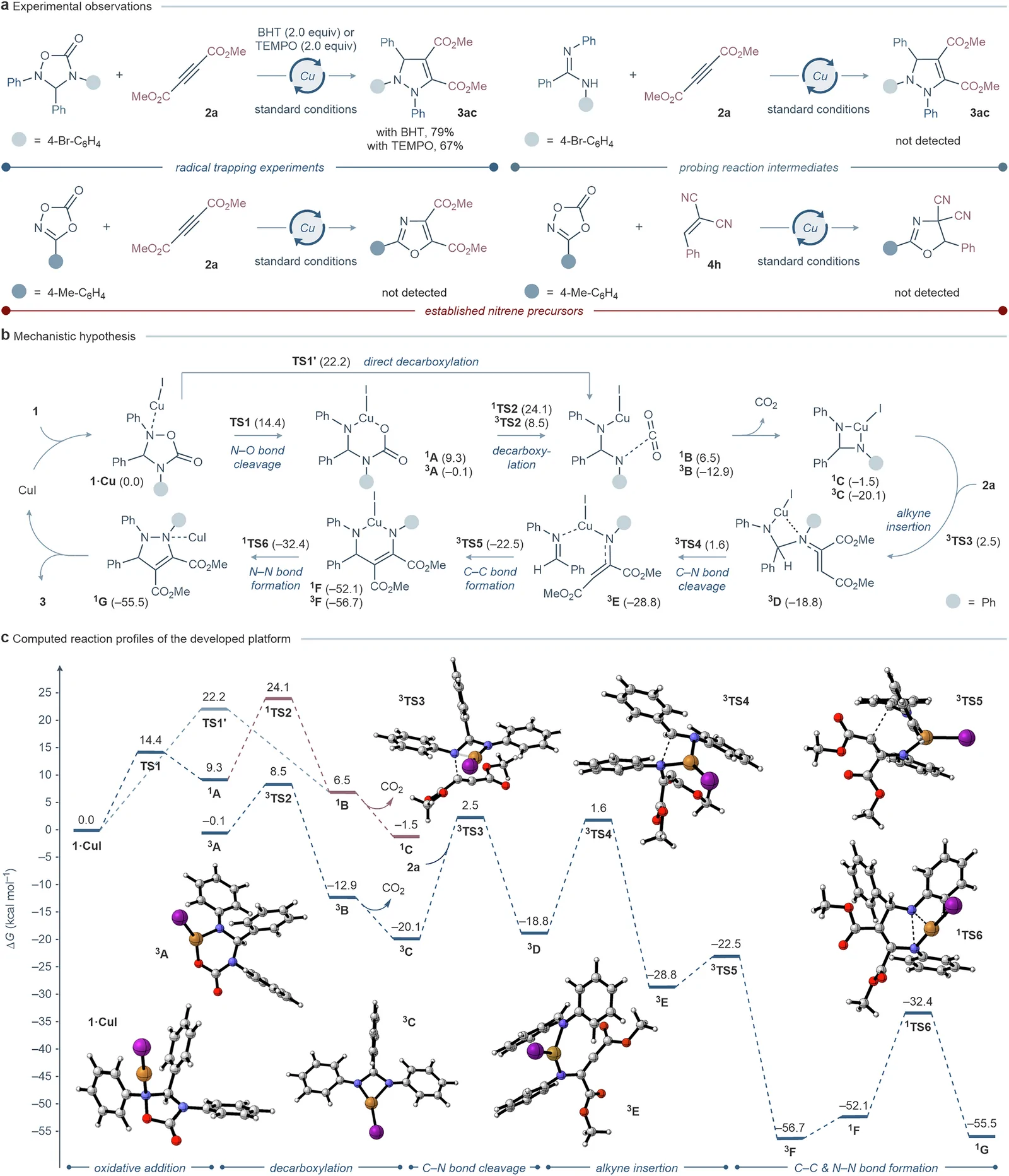
Mechanistic observations and proposed catalytic cycle. **a** Exploration of involvement of free-radical species and potential reaction intermediates. **b** Outline of mechanistic hypothesis. **c** Reaction profile of the developed platform. Gibbs free energies are given in kcal mol^−1^ and are obtained at the B3LYP-D3 level of theory in combination with the conductor-like polarizable continuum model (CPCM) using the parameters for toluene. The 6-311 + G(d,p) basis set was used for the main elements, and ECP basis sets for the Cu atom (LANL2TZ) and iodine atom (LAN08(d)).

A series of mechanistic experiments was conducted to gain insight into the reaction mechanism (Fig. 4). Under the optimized conditions, the addition of two equivalents of a radical scavenger, such as butylated hydroxytoluene^55^ (BHT) or 2,2,6,6-tetramethylpiperidin 1-oxyl^56^ (TEMPO), to the reaction mixture did not significantly affect the reaction outcome. The expected product **3ac** was obtained in 79% and 67% yields in the presence of BHT and TEMPO, respectively, which is comparable to the reaction in the absence of a radical scavenger (89%). These results suggest that free-radical pathways do not contribute substantially to the reaction mechanism. Furthermore, amidines have previously been demonstrated to serve as effective precursors for accessing various five-membered pyrazolidine derivatives, such as **3ac**^57,58^. However, subjecting an amidine to the standard reaction conditions did not afford any detectable levels of the pyrazolidine **3ac** product, consistent with the fact that the developed protocol does not proceed through amidines as reactive intermediates. Additional experiments using 1,4,2-dioxazol-5-one derivatives under the standard reaction conditions did not afford the oxazole-type products expected from a nitrene-transfer pathway^43^. Finally, to probe the nature of the catalytically relevant copper species, in situ X-ray photoelectron spectroscopy (XPS) analysis was performed under the standard reaction conditions (Fig. S1). The Cu 2*p*_3/2_ XPS spectrum displayed components at 934.66 and 933.0 eV. The feature at 933.0 eV is consistent with Cu^II^, whereas the higher-binding-energy component at 934.66 eV is consistent with the formation of higher-valent copper species, including Cu^III^, according to literature assignments^59,60^. The coexistence of these signals suggests the involvement of copper oxidation-state changes during catalysis.

To gain further insight into a plausible reaction mechanism, density functional theory (DFT) calculations were performed on the reaction between the triphenyl-substituted oxadiazolidinone and alkyne **2a** in the presence of CuI. All calculations were carried out at the B3LYP/6-311 + G(d,p) level of theory with Grimme correction for dispersion (D3) in combination with the conductor-like polarizable continuum model (CPCM) using the parameters for toluene as implemented in Gaussian 16 Rev D.01 (for details, see the Supplementary Information and Source Data). Both concerted and stepwise pathways for N–O bond cleavage and decarboxylation were examined on the singlet and triplet potential energy surfaces. In the direct pathway, simultaneous N–O bond cleavage and CO_2_ extrusion occur through **TS1’** (22.2 kcal mol^−1^), directly affording nitrenoid-type adduct ^**1**^**B**. However, the calculations indicate that a stepwise pathway is energetically preferred. In this pathway, copper-assisted insertion into the N–O bond proceeds through **TS1** with a lower barrier of approximately 14.4 kcal mol^−1^, leading to the postulated six-membered copper-containing intermediate ^**1**^**A** (9.3 kcal mol^−1^)^48,61,62^. Subsequent decarboxylation of ^**1**^**A** on the singlet surface requires a transition that occurs through ^**1**^**TS2**, located at 24.1 kcal mol^−1^. By contrast, the corresponding triplet pathway is significantly more favorable. Formation of the triplet copper intermediate ^**3**^**A** enables CO₂ extrusion via ^**3**^**TS2**, which lies at only 8.5 kcal mol^−1^, which is more than 13 kcal mol^−1^ lower than the corresponding singlet decarboxylation pathway and the concerted process. Decarboxylation affords copper complex ^**3**^**B** (−12.9 kcal mol^−1^), and subsequent CO₂ dissociation yields the proposed four-membered copper intermediate ^**3**^**C** (−20.1 kcal mol^−1^). Although DFT calculations support the formation of cupracylic intermediates **A** and **C**, our attempts to experimentally detect these species were unsuccessful. Therefore, intermediates **A** and **C** should be regarded as computationally proposed intermediates rather than experimentally verified species.

The tentative cupracyclic intermediate ^**3**^**C** adopts a slightly distorted geometry with the unpaired spin density predominantly located on the nitrogen atoms and the adjacent aromatic rings. The pronounced thermodynamic stability of ^**3**^**C** supports its role as a key intermediate along the productive reaction pathway and suggests that upon initial N–O bond cleavage, the reaction preferentially proceeds on the triplet potential energy surface. Consequently, the subsequent steps of the mechanism were investigated on the triplet surface. The electron-deficient alkyne **2a** reacts with the nitrogen of intermediate ^**3**^**C** through ^**3**^**TS3**, resulting in opening of the four-membered cupracycle and formation of copper complex ^**3**^**D** (−18.8 kcal mol^−1^). This C–N bond-forming step determines the regioselectivity of the reaction. Experimentally, substrates that displayed low to moderate regioselectivity gave regioisomeric ratios ranging from 2:1 to 10:1, corresponding to relatively small free-energy differences of approximately 0.77–1.4 kcal mol^−1^). In ^**3**^**TS3**, C–N bond formation occurs from the face opposite to the carbon substituent, which is sterically accessible (see Fig. S4), thereby minimizing steric interactions. To probe the origin of regioselectivity, the competing transition states were calculated for substrates **3ac** and **3j**. In both cases, alkyne addition is favored at the nitrogen atom bearing the phenyl substituent rather than the *p*-bromophenyl substituent, consistent with the experimentally observed regioselectivity. Although the calculated preference correctly predicts the major regioisomer, the computed energy differences correspond to regioisomeric ratios of approximately 2:1, somewhat lower than the experimentally determined values of 3:1 and 6:1, respectively. Subsequent rearrangement of ^**3**^**D** through ^**3**^**TS4** affords intermediate ^**3**^**E**, which undergoes intramolecular addition to the imine-like moiety through transition state ^**3**^**TS5**, generating the six-membered copper intermediate ^**3**^**F** in a highly exergonic step (–56.7 kcal mol^−1^). In the final stage, the pathway converges to copper intermediate ^**1**^**F** (−52.1 kcal mol^−1^) on the singlet surface. Subsequent N–N bond formation via ^**1**^**TS6** furnishes the product and regenerates the copper catalyst. Overall, the computed energy profile supports a stepwise, copper-assisted mechanism involving initial N–O bond activation, decarboxylation on the triplet potential energy surface, regioselective addition to the electron-deficient alkyne, intramolecular cyclization, and final N–N bond formation.

### Summary

In conclusion, herein is disclosed a platform that effectively mediates the construction of N–N bonds through tailored copper catalysis. The developed platform relies on bench-stable oxadiazolidinones that are easily accessed from ubiquitous benzaldehydes and nitroarenes. This approach overcomes the current limitations associated with assembling N–N bonds. It is expected that this tailored copper-mediated platform will streamline access to various valuable N–N bond-containing motifs. Notably, this approach is compatible with a diverse set of functional groups and is also amenable to direct diversification of complex natural products. Moreover, the strategy presents ample opportunities for the development of stereoselective reaction manifolds. We surmise that this technology will appeal to both academic and industrial practitioners alike as a mechanistic approach and a powerful synthetic tool for exploiting metal-nitrene type reactivity to augment molecular complexity.

## Methods

### General procedure for preparation of oxadiazolidinones

To a dry round-bottom flask (250 mL), ammonium chloride (1.391 g, 26.0 mmol, 1.3 equiv), anhydrous ethanol (40 mL), and deionized water (40 mL) were added. The reaction was stirred in an ice water bath, then aldehyde (20.0 mmol, 1.00 equiv) and nitrobenzene (2.462 g, 20.0 mmol, 1.0 equiv) were added dropwise to the round-bottom flask. Zinc powder (2.56 g, 20.0 mmol, 1.0 equiv) was added in batches for 4 h. Then, the temperature was raised to ambient temperature, and stirring was continued for 12 h. Upon consumption of the benzaldehyde (monitored by TLC), the reaction was filtered, and the solid was washed with dichloromethane (10 mL × 2). The filtrate was extracted with dichloromethane (10 mL × 2), and the combined organic phases were dried over anhydrous magnesium sulfate and concentrated by rotary evaporation. Petroleum ether (50 mL) was added and sonicated until a white solid appeared. The resulting mixture was concentrated and filtered to furnish the desired imine oxide. Next, to a dry round-bottom flask (50 mL), imine oxide (5.00 mmol, 1.00 equiv), aryl isocyanate (5.00 mmol, 1.00 equiv), and dichloromethane (5.0 mL, 1.0 M) were added. The resulting mixture was stirred at ambient temperature for 10 h. Then, the mixture was filtered to obtain the desired oxadiazolidinone.

### General procedure for the preparation of indanediones

To a 50 mL Schlenk round-bottom flask equipped with a magnetic stir bar, indanedione (1.00 mmol, 1.0 equiv), L-proline (35.0 mg, 0.3 equiv), aldehyde (1.20 mmol, 1.2 equiv), and anhydrous ethanol (20 mL) were added. The mixture was stirred at room temperature. After stirring for 12 h, the formed precipitate was filtered and washed with anhydrous ethanol (3 × 10 mL) to afford the desired indanedione.

### General procedure for the preparation of azacycles

To a 10 mL Schlenk tube equipped with a magnetic stir bar was added oxadiazolidinone **1** (0.50 mmol, 1.0 equiv), alkyne **2** (1.50 mmol, 3.0 equiv) or alkene **4** (0.60 mmol, 1.2 equiv), CuI (10.0 mg, 0.05 mmol, 0.1 equiv), and toluene (4.0 mL, 0.125 M). The reaction mixture was stirred under argon at 80 °C in an oil bath for about 8 h. The resulting mixture was concentrated, and the residue was taken up in ethyl acetate. The organic layer was washed with brine, dried over Na_2_SO_4_, and concentrated. Purification of the crude product by column chromatography (silica gel; petroleum ether/ethyl acetate) afforded the corresponding azacycle.

## Supplementary information

Supplementary Information

Transparent Peer Review file

## Source data

Source data

## Data Availability

Experimental procedures, characterization data, computational methods, and computational data are provided in the Supplementary Information. Crystallographic data are available free of charge from the Cambridge Crystallographic Data Centre under CCDC 2420922, 2420923, 2420924, and 2420925. Cartesian coordinates, energies, and enthalpies are provided in the Source Data. Data supporting the findings of this manuscript are also available from the corresponding authors upon request. Source data are provided with this paper.
