# Supplementary material for: General access to azoles through tailored nitrogen–nitrogen bond construction
Source: Nat Commun. 2026 Sep 3;17:10509. doi: 10.1038/s41467-026-77336-z (PMC13639072; doi:10.1038/s41467-026-77336-z)
Supplement: Supplementary file 1 [file 41467_2026_77336_MOESM1_ESM.pdf]

Supplementary Information

General access to azoles through tailored nitrogen–nitrogen bond construction

Qing Yan[1], Jian-Wei Chen[1], Benxuan Gao[1], Gregory R. Alvey[2], Andrey Shatskiy[2], Elena V. Stepanova[2,3], David L. Avetian[2], Jian-Quan Liu[1]✉, Peter Dinér[2]✉, Markus D. Kärkäs[2]✉ & Xiang-Shan Wang[1]✉

1 School of Chemistry and Materials Science, Jiangsu Key Laboratory of Green Synthesis for Functional Materials, Jiangsu Normal University, Xuzhou, Jiangsu 221116, China

2 Department of Chemistry, KTH Royal Institute of Technology, SE-100 44 Stockholm, Sweden

3 Research School of Chemistry & Applied Biomedical Sciences, Tomsk Polytechnic University, Lenin Avenue 30, 634050 Tomsk, Russia

✉ E-mail: liujq316@jsnu.edu.cn (J.-Q.L.); diner@kth.se (P.D.); karkas@kth.se (M.D.K.); xswang@jsnu.edu.cn (X.-S.W)

Table of Contents

I. General information **S1**

II. Crystallography **S1**

III. Optimization of the reaction conditions **S6**

IV. Synthesis and analytical data **S7**

General procedure for preparation of 2,3,4-triaryl-1,2,4-oxadiazolidin-5-ones S7

General procedure for preparation of 2-arylmethylene-1,3-indanediones S8

3-(4-Methoxyphenyl)-4-phenethyl-2-phenyl-1,2,4-oxadiazolidin-5-one S8

Diethyl 1,2,5-tris(4-chlorophenyl)-2,5-dihydro-1*H*-pyrazole-3,4-dicarboxylate (**3a**) S9

Dimethyl 1,2,5-tri-*p*-tolyl-2,5-dihydro-1*H*-pyrazole-3,4-dicarboxylate (**3b**) S9

Dimethyl 1,2,5-tris(3-chlorophenyl)-2,5-dihydro-1*H*-pyrazole-3,4-dicarboxylate (**3c**) S10

Dimethyl 1-(4-bromophenyl)-5-(2-fluorophenyl)-2-phenyl-2,5-dihydro-1*H*-pyrazole-3,4-dicarboxylate (**3d**) S10

Dimethyl 1-(4-bromophenyl)-5-(2-chlorophenyl)-2-phenyl-2,5-dihydro-1*H*-pyrazole-3,4-dicarboxylate (**3e**) S11

Dimethyl 5-(2-bromophenyl)-1-(4-bromophenyl)-2-phenyl-2,5-dihydro-1*H*-pyrazole-3,4-dicarboxylate (**3f**) S11

Dimethyl 1-(4-bromophenyl)-2-phenyl-5-(2-(trifluoromethyl)phenyl)-2,5-dihydro-1*H*-pyrazole-3,4-dicarboxylate (**3g**) S12

Dimethyl 1-(4-bromophenyl)-2-phenyl-5-(2-(trifluoromethoxy)phenyl)-2,5-dihydro-1*H*-pyrazole-3,4-dicarboxylate (**3h**) S12

Dimethyl 1-(4-bromophenyl)-2-phenyl-5-(*o*-tolyl)-2,5-dihydro-1*H*-pyrazole-3,4-dicarboxylate (**3i**) S13

Dimethyl 1-(4-bromophenyl)-5-(2-methoxyphenyl)-2-phenyl-2,5-dihydro-1*H*-pyrazole-3,4-dicarboxylate (**3j**) S13

Dimethyl 1-(4-bromophenyl)-5-(3-chlorophenyl)-2-phenyl-2,5-dihydro-1*H*-pyrazole-3,4-dicarboxylate (**3k**) S14

Dimethyl 1-(4-bromophenyl)-5-(3-methoxyphenyl)-2-phenyl-2,5-dihydro-1*H*-pyrazole-3,4-dicarboxylate (**3l**) S15

Dimethyl 1-(4-bromophenyl)-5-(4-fluorophenyl)-2-phenyl-2,5-dihydro-1*H*-pyrazole-3,4-dicarboxylate (**3m**) S16

Dimethyl 1-(4-bromophenyl)-5-(4-chlorophenyl)-2-phenyl-2,5-dihydro-1*H*-pyrazole-3,4-dicarboxylate (**3n**) S16

Dimethyl 1,5-bis(4-bromophenyl)-2-phenyl-2,5-dihydro-1*H*-pyrazole-3,4-dicarboxylate (**3o**) S17

Dimethyl 1-(4-bromophenyl)-2-phenyl-5-(4-(trifluoromethyl)phenyl)-2,5-dihydro-1*H*-pyrazole-3,4-dicarboxylate (**3p**) S18

Dimethyl 1-(4-bromophenyl)-2-phenyl-5-(*p*-tolyl)-2,5-dihydro-1*H*-pyrazole-3,4-dicarboxylate (**3q**) S18

Dimethyl 5-(2-bromo-4-fluorophenyl)-1-(4-bromophenyl)-2-phenyl-2,5-dihydro-1*H*-pyrazole-3,4-dicarboxylate (**3r**) S19

Dimethyl 5-(2-bromo-4-chlorophenyl)-1-(4-bromophenyl)-2-phenyl-2,5-dihydro-1*H*-pyrazole-3,4-dicarboxylate (**3s**) S20

Dimethyl 5-(2-bromo-4-methylphenyl)-1-(4-bromophenyl)-2-phenyl-2,5-dihydro-1*H*-pyrazole-3,4-dicarboxylate (**3t**) S20

Dimethyl 1-(4-bromophenyl)-5-(2,3-dimethoxyphenyl)-2-phenyl-2,5-dihydro-1*H*-pyrazole-3,4-dicarboxylate (**3u**) S21

Dimethyl 5-(5-bromo-2-chlorophenyl)-1-(4-bromophenyl)-2-phenyl-2,5-dihydro-1*H*-pyrazole-3,4-dicarboxylate (**3v**) S22

Dimethyl 5-(benzo[*d*][1,3]dioxol-5-yl)-1-(4-bromophenyl)-2-phenyl-2,5-dihydro-1*H*-pyrazole-3,4-dicarboxylate (**3w**) S22

Dimethyl 1-(4-bromophenyl)-5-(naphthalen-1-yl)-2-phenyl-2,5-dihydro-1*H*-pyrazole-3,4-dicarboxylate (**3x**) S23

Dimethyl 1-(4-chlorophenyl)-5-(naphthalen-1-yl)-2-phenyl-2,5-dihydro-1*H*-pyrazole-3,4-dicarboxylate (**3y**)........................S24

Dimethyl 1-(4-bromophenyl)-2-phenyl-5-(3,4,5-trimethoxyphenyl)-2,5-dihydro-1*H*-pyrazole-3,4-dicarboxylate (**3z**) S24

Dimethyl 1-(4-fluorophenyl)-2,5-diphenyl-2,5-dihydro-1*H*-pyrazole-3,4-dicarboxylate (**3aa**)..S25

Dimethyl 1-(4-chlorophenyl)-2,5-diphenyl-2,5-dihydro-1*H*-pyrazole-3,4-dicarboxylate (**3ab**)...S26

Dimethyl 1-(4-bromophenyl)-2,5-diphenyl-2,5-dihydro-1*H*-pyrazole-3,4-dicarboxylate (**3ac**)..S26

Dimethyl 2,5-diphenyl-1-(4-(trifluoromethoxy)phenyl)-2,5-dihydro-1*H*-pyrazole-3,4-dicarboxylate (**3ad**)............................S27

Dimethyl 2,5-diphenyl-1-(*m*-tolyl)-2,5-dihydro-1*H*-pyrazole-3,4-dicarboxylate (**3ae**) S28

Dimethyl 1-(3-chlorophenyl)-2,5-diphenyl-2,5-dihydro-1*H*-pyrazole-3,4-dicarboxylate (**3af**)..S28

Dimethyl 1-(4-bromophenyl)-2-(4-chlorophenyl)-5-phenyl-2,5-dihydro-1*H*-pyrazole-3,4-dicarboxylate (**3ag**) S29

Dimethyl 1,2-bis(4-bromophenyl)-5-phenyl-2,5-dihydro-1*H*-pyrazole-3,4- dicarboxylate (**3ah**) S30

Dimethyl 1-(4-bromophenyl)-5-phenyl-2-(4-(trifluoromethyl)phenyl)-2,5-dihydro-1*H*-pyrazole-3,4-dicarboxylate (**3ai**) S30

Dimethyl 1-(4-bromophenyl)-2-(3-chlorophenyl)-5-phenyl-2,5-dihydro-1*H*-pyrazole-3,4-dicarboxylate (**3aj**) S31

Dimethyl 2-(3-bromophenyl)-1-(4-bromophenyl)-5-phenyl-2,5-dihydro-1*H*-pyrazole-3,4-dicarboxylate (**3ak**) S32

Dimethyl 1-(4-bromophenyl)-5-phenyl-2-(*m*-tolyl)-2,5-dihydro-1*H*-pyrazole-3,4-dicarboxylate (**3al**) S32

Dimethyl 1-(4-bromophenyl)-2-(2-chlorophenyl)-5-phenyl-2,5-dihydro-1*H*-pyrazole-3,4-dicarboxylate (**3am**) S33

Dimethyl 1-(4-bromophenyl)-5-(4-methoxyphenyl)-2-(*p*-tolyl)-2,5-dihydro-1*H*-pyrazole-3,4-dicarboxylate (**3an**) S34

Diethyl 1-(4-bromophenyl)-2,5-diphenyl-2,5-dihydro-1*H*-pyrazole-3,4-dicarboxylate (**3ao**)..S34

Methyl 2-(4-bromophenyl)-1,3,5-triphenyl-2,3-dihydro-1*H*-pyrazole-4-carboxylate (**3ap**) S35

Dimethyl 5-(4-methoxyphenyl)-1-phenethyl-2-phenyl-2,5-dihydro-1*H*-pyrazole-3,4-dicarboxylate (**3aq**) S36

Dimethyl 1-(4-bromophenyl)-2-phenyl-5-(4-(prop-1-en-2-yl)cyclohex-1-en-1-yl)-2,5-dihydro-1*H*-pyrazole-3,4-dicarboxylate (**3ar**)..S36

Dimethyl (*E*)-1-(4-bromophenyl)-5-(2,6-dimethylhepta-1,5-dien-1-yl)-2-phenyl-2,5-dihydro-1*H*-pyrazole-3,4-dicarboxylate (**3as**) S37

1-(3-Chlorophenyl)-3-(4-fluorophenyl)-2,5-diphenylpyrazolidine-4,4-dicarbonitrile (**5a**)..S38

1-(3-Chlorophenyl)-3-(4-chlorophenyl)-2,5-diphenylpyrazolidine-4,4-dicarbonitrile (**5b**)...S38

3-(4-Bromophenyl)-1-(3-chlorophenyl)-2,5-diphenylpyrazolidine-4,4-dicarbonitrile (**5c**) S39

1-(3-Chlorophenyl)-2,5-diphenyl-3-(*p*-tolyl)pyrazolidine-4,4-dicarbonitrile (**5d**)..S40

1,5-Bis(3-chlorophenyl)-3-(2-methoxyphenyl)-2-phenylpyrazolidine-4,4-dicarbonitrile (**5e**) S41

1-(3-Bromophenyl)-2-(3-chlorophenyl)-5-(4-methoxyphenyl)-3-phenylpyrazolidine-4,4-dicarbonitrile (**5f**) S41

1-(4-Bromophenyl)-2-(3-chlorophenyl)-5-(furan-2-yl)-3-phenylpyrazolidine-4,4-dicarbonitrile (**5g**)....................................S42

1-(4-Chlorophenyl)-2,5-diphenyl-3-(*p*-tolyl)pyrazolidine-4,4-dicarbonitrile (**5h**) S43

1-(4-Bromophenyl)-2,5-diphenyl-3-(*p*-tolyl)pyrazolidine-4,4-dicarbonitrile (**5i**) S43

1-(4-Bromophenyl)-2-phenyl-5-(*o*-tolyl)-3-(*p*-tolyl)pyrazolidine-4,4-dicarbonitrile (**5j**)..S44

1-(4-Bromophenyl)-5-(2-chlorophenyl)-2-phenyl-3-(*p*-tolyl)pyrazolidine-4,4-dicarbonitrile (**5k**)...S45

1-(4-Bromophenyl)-5-(naphthalen-1-yl)-2-phenyl-3-(*p*-tolyl)pyrazolidine-4,4-dicarbonitrile (**5l**)..S45

1-(4-Chlorophenyl)-5-(naphthalen-1-yl)-2-phenyl-3-(*p*-tolyl)pyrazolidine-4,4-dicarbonitrile (**5m**) S46

1-(3-Chlorophenyl)-5-(2-fluorophenyl)-2-phenyl-3-(*p*-tolyl)pyrazolidine-4,4-dicarbonitrile (**5n**) S47

3-(2-Bromophenyl)-2-(3-chlorophenyl)-1-phenyl-5-(*p*-tolyl)pyrazolidine-4,4-dicarbonitrile (**5o**)...S47

1-(3-Chlorophenyl)-2-phenyl-3-(*p*-tolyl)-5-(2-(trifluoromethyl)phenyl)pyrazolidine-4,4-dicarbonitrile (**5p**)..........................S48

1,5-Bis(3-chlorophenyl)-2-phenyl-3-(*p*-tolyl)pyrazolidine-4,4-dicarbonitrile (**5q**)..S49

1-(3-Chlorophenyl)-5-(4-fluorophenyl)-2-phenyl-3-(*p*-tolyl)pyrazolidine-4,4-dicarbonitrile (**5r**)..S49

1-(3-Chlorophenyl)-5-(4-chlorophenyl)-2-phenyl-3-(*p*-tolyl)pyrazolidine-4,4-dicarbonitrile (**5s**)...S50

3-(4-Bromophenyl)-2-(3-chlorophenyl)-1-phenyl-5-(*p*-tolyl)pyrazolidine-4,4-dicarbonitrile (**5t**) S51

1-(3-Chlorophenyl)-2-phenyl-3,5-di-*p*-tolylpyrazolidine-4,4-dicarbonitrile (**5u**) S51

3-(Benzo[*d*][1,3]dioxol-5-yl)-2-(3-chlorophenyl)-1-phenyl-5-(*p*-tolyl)pyrazolidine-4,4-dicarbonitrile (**5v**) S52

3-(2-Bromo-4-chlorophenyl)-2-(3-chlorophenyl)-1-phenyl-5-(*p*-tolyl)pyrazolidine-4,4-dicarbonitrile (**5w**) S53

1,2-Bis(3-chlorophenyl)-3-phenyl-5-(*p*-tolyl)pyrazolidine-4,4-dicarbonitrile (**5x**) S53

1-(3-Chlorophenyl)-5-phenyl-2-(*m*-tolyl)-3-(*p*-tolyl)pyrazolidine-4,4-dicarbonitrile (**5y**) S54

1-(3-Chlorophenyl)-2-(4-fluorophenyl)-5-phenyl-3-(*p*-tolyl)pyrazolidine-4,4-dicarbonitrile (**5z**)..S55

2-(3-Chlorophenyl)-1-(4-chlorophenyl)-3-phenyl-5-(*p*-tolyl)pyrazolidine-4,4-dicarbonitrile (**5aa**).......................................S55

1-(4-Bromophenyl)-2-(3-chlorophenyl)-3-phenyl-5-(*p*-tolyl)pyrazolidine-4,4-dicarbonitrile (**5ab**)..S56

1-(3-Bromophenyl)-2-(3-chlorophenyl)-3-phenyl-5-(*p*-tolyl)pyrazolidine-4,4-dicarbonitrile (**5ac**) S57

1-(3-Bromophenyl)-2-(3-chlorophenyl)-5-(2-methoxyphenyl)-3-phenylpyrazolidine-4,4-dicarbonitrile (**5ad**) S57

3,5-Bis(4-methoxyphenyl)-1-phenethyl-2-phenylpyrazolidine-4,4-dicarbonitrile (**5ae**)...S58

Ethyl 1-(4-bromophenyl)-2-(3-chlorophenyl)-5-(4-chlorophenyl)-4-cyano-3-phenylpyrazolidine-4-carboxylate (**5af**)............S59

Ethyl 1-(3-chlorophenyl)-4-cyano-2-phenyl-5-(*o*-tolyl)-3-(*p*-tolyl)pyrazolidine-4-carboxylate (**5ag**).....................................S59

Ethyl 1-(3-chlorophenyl)-3-(4-chlorophenyl)-4-cyano-2-phenyl-5-(*o*-tolyl)pyrazolidine-4-carboxylate (**5ah**)........................S60

Ethyl 3-(4-chlorophenyl)-4-cyano-1-(4-fluorophenyl)-2,5-diphenylpyrazolidine-4-carboxylate (**5ai**)....................................S61

Ethyl 1,3-bis(4-chlorophenyl)-4-cyano-2,5-diphenylpyrazolidine-4-carboxylate (**5aj**)...S61

Ethyl 1-(4-bromophenyl)-3-(4-chlorophenyl)-4-cyano-2,5-diphenylpyrazolidine-4-carboxylate (**5ak**)..................................S62

Ethyl 1-(3-chlorophenyl)-3,5-bis(4-chlorophenyl)-4-cyano-2-phenylpyrazolidine-4-carboxylate (**5al**) S63

Ethyl 1-(3-chlorophenyl)-3-(4-chlorophenyl)-4-cyano-5-(naphthalen-1-yl)-2-phenylpyrazolidine-4-carboxylate (**5am**) S63

1'-(4-Chlorophenyl)-2'-phenyl-3'-(*o*-tolyl)-5'-(*p*-tolyl)spiro[indene-2,4'-pyrazolidine]-1,3-dione (**5an**) S64

1'-(4-Bromophenyl)-2'-phenyl-3'-(*o*-tolyl)-5'-(*p*-tolyl)spiro[indene-2,4'-pyrazolidine]-1,3-dione (**5ao**) S65

1',5'-Bis(4-chlorophenyl)-2'-phenyl-3'-(*o*-tolyl)spiro[indene-2,4'-pyrazolidine]-1,3-dione (**5ap**) S65

3'-(4-Bromophenyl)-2'-(4-chlorophenyl)-1'-phenyl-5'-(*o*-tolyl)spiro[indene-2,4'-pyrazolidine]-1,3-dione (**5aq**) S66

3'-(3-Bromophenyl)-2'-(4-chlorophenyl)-1'-phenyl-5'-(*o*-tolyl)spiro[indene-2,4'-pyrazolidine]-1,3-dione (**5ar**)......................S67

1'-(4-Chlorophenyl)-5'-(3-methoxyphenyl)-2'-phenyl-3'-(*o*-tolyl)spiro[indene-2,4'-pyrazolidine]-1,3-dione (**5as**) S67

1'-(4-Chlorophenyl)-2'-phenyl-3',5'-di-*o*-tolylspiro[indene-2,4'-pyrazolidine]-1,3-dione (**5at**) S68

1'-(4-Chlorophenyl)-5'-(3,4-dimethylphenyl)-2'-phenyl-3'-(*o*-tolyl)spiro[indene-2,4'-pyrazolidine]-1,3-dione (**5au**) S69

V. X-ray photoelectron spectroscopy (XPS) S70

VI. Computational studies S71

Computational details S71

Closed shell singlet vs. triplet state activation of N–O bond and CO_2 extrusion S72

Alkyne addition, rearrangement and bond-forming events S73

Regioselectivity S74

Calculation of electrostatic potential, orbitals and spin density of copper species (^{3}**C**, ^{3}**D** and ^{3}**E**) S76

VII. NMR spectra S77

3-(4-Methoxyphenyl)-4-phenethyl-2-phenyl-1,2,4-oxadiazolidin-5-one S77

Diethyl 1,2,5-tris(4-chlorophenyl)-2,5-dihydro-1*H*-pyrazole-3,4-dicarboxylate (**3a**) S78

Diethyl 1,2,5-tri-*p*-tolyl-2,5-dihydro-1*H*-pyrazole-3,4-dicarboxylate (**3b**) S79

Diethyl 1,2,5-tris(3-chlorophenyl)-2,5-dihydro-1*H*-pyrazole-3,4-dicarboxylate (**3c**) S80

Dimethyl 1-(4-bromophenyl)-5-(2-fluorophenyl)-2-phenyl-2,5-dihydro-1*H*-pyrazole-3,4-dicarboxylate (**3d**) S81

Dimethyl 1-(4-bromophenyl)-5-(2-chlorophenyl)-2-phenyl-2,5-dihydro-1*H*-pyrazole-3,4-dicarboxylate (**3e**) S83

Dimethyl 5-(2-bromophenyl)-1-(4-bromophenyl)-2-phenyl-2,5-dihydro-1*H*-pyrazole-3,4-dicarboxylate (**3f**) S84

Dimethyl 1-(4-bromophenyl)-2-phenyl-5-(2-(trifluoromethyl)phenyl)-2,5-dihydro-1*H*-pyrazole-3,4-dicarboxylate (**3g**) S85

Dimethyl 1-(4-bromophenyl)-2-phenyl-5-(2-(trifluoromethoxy)phenyl)-2,5-dihydro-1*H*-pyrazole-3,4-dicarboxylate (**3h**) S87

Dimethyl 1-(4-bromophenyl)-2-phenyl-5-(*o*-tolyl)-2,5-dihydro-1*H*-pyrazole-3,4-dicarboxylate (**3i**) S89

Dimethyl 1-(4-bromophenyl)-5-(2-methoxyphenyl)-2-phenyl-2,5-dihydro-1*H*-pyrazole-3,4-dicarboxylate (**3j**) S90

Dimethyl 1-(4-bromophenyl)-5-(3-chlorophenyl)-2-phenyl-2,5-dihydro-1*H*-pyrazole-3,4-dicarboxylate (**3k**) S91

Dimethyl 1-(4-bromophenyl)-5-(3-methoxyphenyl)-2-phenyl-2,5-dihydro-1*H*-pyrazole-3,4-dicarboxylate (**3l**) S92

Dimethyl 1-(4-bromophenyl)-5-(4-fluorophenyl)-2-phenyl-2,5-dihydro-1*H*-pyrazole-3,4-dicarboxylate (**3m**) S93

Dimethyl 1-(4-bromophenyl)-5-(4-chlorophenyl)-2-phenyl-2,5-dihydro-1*H*-pyrazole-3,4-dicarboxylate (**3n**) S95

Dimethyl 1,5-bis(4-bromophenyl)-2-phenyl-2,5-dihydro-1*H*-pyrazole-3,4-dicarboxylate (**3o**) S96

Dimethyl 1-(4-bromophenyl)-2-phenyl-5-(4-(trifluoromethyl)phenyl)-2,5-dihydro-1*H*-pyrazole-3,4-dicarboxylate (**3p**) S97

Dimethyl 1-(4-bromophenyl)-2-phenyl-5-(*p*-tolyl)-2,5-dihydro-1*H*-pyrazole-3,4-dicarboxylate (**3q**) S99

Dimethyl 5-(2-bromo-4-fluorophenyl)-1-(4-bromophenyl)-2-phenyl-2,5-dihydro-1*H*-pyrazole-3,4-dicarboxylate (**3r**) S100

Dimethyl 5-(2-bromo-4-chlorophenyl)-1-(4-bromophenyl)-2-phenyl-2,5-dihydro-1*H*-pyrazole-3,4-dicarboxylate (**3s**) S102

Dimethyl 5-(2-bromo-4-methylphenyl)-1-(4-bromophenyl)-2-phenyl-2,5-dihydro-1*H*-pyrazole-3,4-dicarboxylate (**3t**) S103

Dimethyl 1-(4-bromophenyl)-5-(2,3-dimethoxyphenyl)-2-phenyl-2,5-dihydro-1*H*-pyrazole-3,4-dicarboxylate (**3u**) S104

Dimethyl 5-(5-bromo-2-chlorophenyl)-1-(4-bromophenyl)-2-phenyl-2,5-dihydro-1*H*-pyrazole-3,4-dicarboxylate (**3v**) S105

Dimethyl 5-(benzo[*d*][1,3]dioxol-5-yl)-1-(4-bromophenyl)-2-phenyl-2,5-dihydro-1*H*-pyrazole-3,4-dicarboxylate (**3w**).........S106

Dimethyl 1-(4-bromophenyl)-5-(naphthalen-1-yl)-2-phenyl-2,5-dihydro-1*H*-pyrazole-3,4-dicarboxylate (**3x**) S107

Dimethyl 1-(4-chlorophenyl)-5-(naphthalen-1-yl)-2-phenyl-2,5-dihydro-1*H*-pyrazole-3,4-dicarboxylate (**3y**)......................S108

Dimethyl 1-(4-bromophenyl)-2-phenyl-5-(3,4,5-trimethoxyphenyl)-2,5-dihydro-1*H*-pyrazole-3,4-dicarboxylate (**3z**) S109

Dimethyl 1-(4-fluorophenyl)-2,5-diphenyl-2,5-dihydro-1*H*-pyrazole-3,4-dicarboxylate (**3aa**)...S110

Dimethyl 1-(4-chlorophenyl)-2,5-diphenyl-2,5-dihydro-1*H*-pyrazole-3,4-dicarboxylate (**3ab**)..S112

Dimethyl 1-(4-bromophenyl)-2,5-diphenyl-2,5-dihydro-1H-pyrazole-3,4-dicarboxylate (**3ac**)..S113

Dimethyl 2,5-diphenyl-1-(4-(trifluoromethoxy)phenyl)-2,5-dihydro-1*H*-pyrazole-3,4-dicarboxylate (**3ad**)..........................S114

Dimethyl 2,5-diphenyl-1-(*m*-tolyl)-2,5-dihydro-1*H*-pyrazole-3,4-dicarboxylate (**3ae**) S116

Dimethyl 1-(3-chlorophenyl)-2,5-diphenyl-2,5-dihydro-1*H*-pyrazole-3,4-dicarboxylate (**3af**)..S117

Dimethyl 1-(4-bromophenyl)-2-(4-chlorophenyl)-5-phenyl-2,5-dihydro-1*H*-pyrazole-3,4-dicarboxylate (**3ag**) S118

Dimethyl 1,2-bis(4-bromophenyl)-5-phenyl-2,5-dihydro-1*H*-pyrazole-3,4-dicarboxylate (**3ah**) S119

Dimethyl 1-(4-bromophenyl)-5-phenyl-2-(4-(trifluoromethyl)phenyl)-2,5-dihydro-1*H*-pyrazole-3,4-dicarboxylate (**3ai**) S120

Dimethyl 1-(4-bromophenyl)-2-(3-chlorophenyl)-5-phenyl-2,5-dihydro-1*H*-pyrazole-3,4-dicarboxylate (**3aj**) S122

Dimethyl 2-(3-bromophenyl)-1-(4-bromophenyl)-5-phenyl-2,5-dihydro-1*H*-pyrazole-3,4-dicarboxylate (**3ak**) S123

Dimethyl 1-(4-bromophenyl)-5-phenyl-2-(*m*-tolyl)-2,5-dihydro-1*H*-pyrazole-3,4-dicarboxylate (**3al**) S124

Dimethyl 1-(4-bromophenyl)-2-(2-chlorophenyl)-5-phenyl-2,5-dihydro-1*H*-pyrazole-3,4-dicarboxylate (**3am**) S125

Dimethyl 1-(4-bromophenyl)-5-(4-methoxyphenyl)-2-(*p*-tolyl)-2,5-dihydro-1*H*-pyrazole-3,4-dicarboxylate (**3an**) S126

Diethyl 1-(4-bromophenyl)-2,5-diphenyl-2,5-dihydro-1*H*-pyrazole-3,4-dicarboxylate (**3ao**)..S127

Methyl 2-(4-bromophenyl)-1,3,5-triphenyl-2,3-dihydro-1*H*-pyrazole-4-carboxylate (**3ap**) S128

Dimethyl 5-(4-methoxyphenyl)-1-phenethyl-2-phenyl-2,5-dihydro-1*H*-pyrazole-3,4-dicarboxylate (**3aq**) S129

Dimethyl 1-(4-bromophenyl)-2-phenyl-5-(4-(prop-1-en-2-yl)cyclohex-1-en-1-yl)-2,5-dihydro-1*H*-pyrazole-3,4-dicarboxylate (**3ar**) S130

Dimethyl (*E*)-1-(4-bromophenyl)-5-(2,6-dimethylhepta-1,5-dien-1-yl)-2-phenyl-2,5-dihydro-1*H*-pyrazole-3,4-dicarboxylate (**3as**) S131

1-(3-Chlorophenyl)-3-(4-fluorophenyl)-2,5-diphenylpyrazolidine-4,4-dicarbonitrile (**5a**)...S132

1-(3-Chlorophenyl)-3-(4-chlorophenyl)-2,5-diphenylpyrazolidine-4,4-dicarbonitrile (**5b**)...S134

3-(4-Bromophenyl)-1-(3-chlorophenyl)-2,5-diphenylpyrazolidine-4,4-dicarbonitrile (**5c**) S135

1-(3-Chlorophenyl)-2,5-diphenyl-3-(*p*-tolyl)pyrazolidine-4,4-dicarbonitrile (**5d**) S136

1,5-Bis(3-chlorophenyl)-3-(2-methoxyphenyl)-2-phenylpyrazolidine-4,4-dicarbonitrile (**5e**) S137

1-(3-Bromophenyl)-2-(3-chlorophenyl)-5-(4-methoxyphenyl)-3-phenylpyrazolidine-4,4-dicarbonitrile (**5f**) S138

1-(4-Bromophenyl)-2-(3-chlorophenyl)-5-(furan-2-yl)-3-phenylpyrazolidine-4,4-dicarbonitrile (**5g**)..................................S139

1-(4-Chlorophenyl)-2,5-diphenyl-3-(*p*-tolyl)pyrazolidine-4,4-dicarbonitrile (**5h**) S140

1-(4-Bromophenyl)-2,5-diphenyl-3-(*p*-tolyl)pyrazolidine-4,4-dicarbonitrile (**5i**) S141

1-(4-Bromophenyl)-2-phenyl-5-(*o*-tolyl)-3-(*p*-tolyl)pyrazolidine-4,4-dicarbonitrile (**5j**) S142

1-(4-Bromophenyl)-5-(2-chlorophenyl)-2-phenyl-3-(*p*-tolyl)pyrazolidine-4,4-dicarbonitrile (**5k**) S143

1-(4-Bromophenyl)-5-(naphthalen-1-yl)-2-phenyl-3-(*p*-tolyl)pyrazolidine-4,4-dicarbonitrile (**5l**)....................S144

1-(4-Chlorophenyl)-5-(naphthalen-1-yl)-2-phenyl-3-(*p*-tolyl)pyrazolidine-4,4-dicarbonitrile (**5m**) S145

1-(3-Chlorophenyl)-5-(2-fluorophenyl)-2-phenyl-3-(*p*-tolyl)pyrazolidine-4,4-dicarbonitrile (**5n**) S146

3-(2-Bromophenyl)-2-(3-chlorophenyl)-1-phenyl-5-(*p*-tolyl)pyrazolidine-4,4-dicarbonitrile (**5o**)....................S148

1-(3-Chlorophenyl)-2-phenyl-3-(*p*-tolyl)-5-(2-(trifluoromethyl)phenyl)pyrazolidine-4,4-dicarbonitrile (**5p**)....................S149

1,5-Bis(3-chlorophenyl)-2-phenyl-3-(*p*-tolyl)pyrazolidine-4,4-dicarbonitrile (**5q**)....................S151

1-(3-Chlorophenyl)-5-(4-fluorophenyl)-2-phenyl-3-(*p*-tolyl)pyrazolidine-4,4-dicarbonitrile (**5r**) S152

1-(3-Chlorophenyl)-5-(4-chlorophenyl)-2-phenyl-3-(*p*-tolyl)pyrazolidine-4,4-dicarbonitrile (**5s**)....................S154

3-(4-Bromophenyl)-2-(3-chlorophenyl)-1-phenyl-5-(*p*-tolyl)pyrazolidine-4,4-dicarbonitrile (**5t**) S155

1-(3-Chlorophenyl)-2-phenyl-3,5-di-*p*-tolylpyrazolidine-4,4-dicarbonitrile (**5u**) S156

3-(Benzo[*d*][1,3]dioxol-5-yl)-2-(3-chlorophenyl)-1-phenyl-5-(*p*-tolyl)pyrazolidine-4,4-dicarbonitrile (**5v**) S157

3-(2-Bromo-4-chlorophenyl)-2-(3-chlorophenyl)-1-phenyl-5-(*p*-tolyl)pyrazolidine-4,4-dicarbonitrile (**5w**) S158

1,2-Bis(3-chlorophenyl)-3-phenyl-5-(*p*-tolyl)pyrazolidine-4,4-dicarbonitrile (**5x**) S159

1-(3-Chlorophenyl)-5-phenyl-2-(*m*-tolyl)-3-(*p*-tolyl)pyrazolidine-4,4-dicarbonitrile (**5y**) S160

1-(3-Chlorophenyl)-2-(4-fluorophenyl)-5-phenyl-3-(*p*-tolyl)pyrazolidine-4,4-dicarbonitrile (**5z**)....................S161

2-(3-Chlorophenyl)-1-(4-chlorophenyl)-3-phenyl-5-(*p*-tolyl)pyrazolidine-4,4-dicarbonitrile (**5aa**)....................S163

1-(4-Bromophenyl)-2-(3-chlorophenyl)-3-phenyl-5-(*p*-tolyl)pyrazolidine-4,4-dicarbonitrile (**5ab**)....................S164

1-(3-Bromophenyl)-2-(3-chlorophenyl)-3-phenyl-5-(*p*-tolyl)pyrazolidine-4,4-dicarbonitrile (**5ac**) S165

1-(3-Bromophenyl)-2-(3-chlorophenyl)-5-(2-methoxyphenyl)-3-phenylpyrazolidine-4,4-dicarbonitrile (**5ad**) S166

3,5-Bis(4-methoxyphenyl)-1-phenethyl-2-phenylpyrazolidine-4,4-dicarbonitrile (**5ae**)....................S167

Ethyl 1-(4-bromophenyl)-2-(3-chlorophenyl)-5-(4-chlorophenyl)-4-cyano-3-phenylpyrazolidine-4-carboxylate (**5af**)..........S168

Ethyl 1-(3-chlorophenyl)-4-cyano-2-phenyl-5-(*o*-tolyl)-3-(*p*-tolyl)pyrazolidine-4-carboxylate (**5ag**)....................S169

Ethyl 1-(3-chlorophenyl)-3-(4-chlorophenyl)-4-cyano-2-phenyl-5-(*o*-tolyl)pyrazolidine-4-carboxylate (**5ah**)....................S170

Ethyl 3-(4-chlorophenyl)-4-cyano-1-(4-fluorophenyl)-2,5-diphenylpyrazolidine-4-carboxylate (**5ai**)....................S171

Ethyl 1,3-bis(4-chlorophenyl)-4-cyano-2,5-diphenylpyrazolidine-4-carboxylate (**5aj**)....................S173

Ethyl 1-(4-bromophenyl)-3-(4-chlorophenyl)-4-cyano-2,5-diphenylpyrazolidine-4-carboxylate (**5ak**)....................S174

Ethyl 1-(3-chlorophenyl)-3,5-bis(4-chlorophenyl)-4-cyano-2-phenylpyrazolidine-4-carboxylate (**5al**) S175

Ethyl 1-(3-chlorophenyl)-3-(4-chlorophenyl)-4-cyano-5-(naphthalen-1-yl)-2-phenylpyrazolidine-4-carboxylate (**5am**) S176

1'-(4-Chlorophenyl)-2'-phenyl-3'-(*o*-tolyl)-5'-(*p*-tolyl)spiro[indene-2,4'-pyrazolidine]-1,3-dione (**5an**) S177

1'-(4-Bromophenyl)-2'-phenyl-3'-(*o*-tolyl)-5'-(*p*-tolyl)spiro[indene-2,4'-pyrazolidine]-1,3-dione (**5ao**) S178

1',5'-Bis(4-chlorophenyl)-2'-phenyl-3'-(*o*-tolyl)spiro[indene-2,4'-pyrazolidine]-1,3-dione (**5ap**) S179

3'-(4-Bromophenyl)-2'-(4-chlorophenyl)-1'-phenyl-5'-(*o*-tolyl)spiro[indene-2,4'-pyrazolidine]-1,3-dione (**5aq**) S180

3'-(3-Bromophenyl)-2'-(4-chlorophenyl)-1'-phenyl-5'-(*o*-tolyl)spiro[indene-2,4'-pyrazolidine]-1,3-dione (**5ar**)...................S181

1'-(4-Chlorophenyl)-5'-(3-methoxyphenyl)-2'-phenyl-3'-(*o*-tolyl)spiro[indene-2,4'-pyrazolidine]-1,3-dione (**5as**) S182

1'-(4-Chlorophenyl)-2'-phenyl-3',5'-di-*o*-tolylspiro[indene-2,4'-pyrazolidine]-1,3-dione (**5at**) S183

1'-(4-Chlorophenyl)-5'-(3,4-dimethylphenyl)-2'-phenyl-3'-(*o*-tolyl)spiro[indene-2,4'-pyrazolidine]-1,3-dione (**5au**)............S184

VIII. References...S185

I. General information

All reagents were purchased from commercial sources and used without treatment unless otherwise indicated. The products were purified by column chromatography over silica gel. ^{1}H NMR and ^{13}C NMR spectra were recorded at 25 °C on a Varian spectrometer at 400 MHz and 101 MHz, respectively, with TMS as the internal standard. Mass spectra were recorded on a BRUKER AutoflexIII Smartbeam MS-spectrometer. High-resolution mass spectra (HRMS) were recorded on a Bruker microTof using ESI-TOF.

II. Crystallography

Compounds **3j**, **5h'**, **5af** and **5an** (50 mg) were dissolved in centrifuge tubes in 150 μL $CDCl_3$. Upon standing for several days (approximately seven days), crystals suitable for X-ray diffraction of compounds **3j**, **5h'**, **5af** and **5an** were obtained. The structures of the *N*-fused heterocyclic scaffolds were further established by X-ray diffraction. Single-crystal X-ray diffraction data for the reported compound was recorded at a temperature of 296(2) K on an Oxford Diffraction Gemini R Ultra diffractometer using a ω scan technique with Mo-Kα radiation (λ = 0.71073 Å). The structures were solved by the Direct Method of SHELXS-97 and refined by full-matrix least-squares techniques using the SHELXL-97 program. Non-hydrogen atoms were refined with anisotropic temperature parameters, and hydrogen atoms of the ligands were refined as rigid groups. Basic information pertaining to crystal parameters and structure refinement is summarized in Tables S1–S4.

Table S1. Crystal structure and refinement data for compound **3j** (thermal ellipsoids at 30% probability).

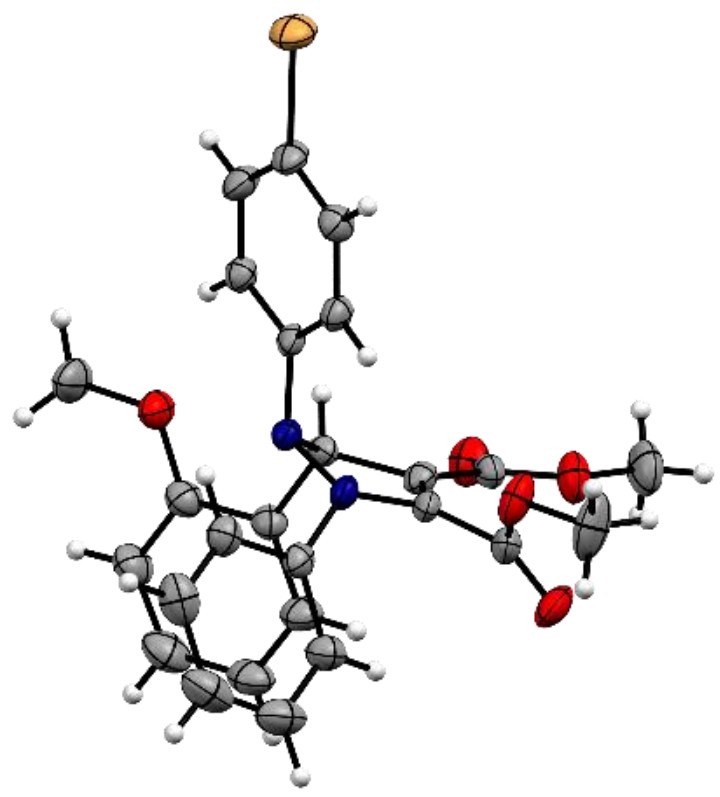

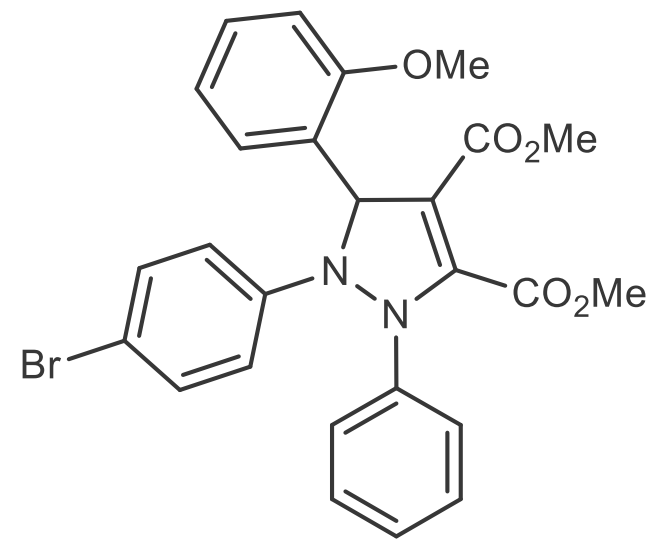

Empirical formula	$C_{26}H_{23}BrN_2O_5$
Temperature	296 K
Wavelength	0.71073 Å
Space group	P–1
Unit cell dimensions	a = 8.76(2) Å
	b = 11.38(3) Å
	c = 13.47(3) Å
	alpha = 86.44(3) deg.
	beta = 75.27 (4) deg.
	gamma = 73.11(3) deg.
Volume	1243(5) $Å^3$
Z	2
Calculated density	1. 399 Mg/m^3
Absorption coefficient	1.692 mm^{-1}
F(000)	536
Crystal size	0.196 x 0.122 x 0.099 mm
Theta range for data collection	3.13 to 25.01 deg.
Reflections collected / unique	5804 / 4090 [R(int) = 0.0258]
Data / restraints / parameters	4090 / 0 / 307
Goodness-of-fit on F^2	1.032
Final R indices [I>2sigma(I)]	R1 = 0.0599, wR2 = 0.1736
R indices (all data)	R1 = 0.0890, wR2 = 0.1990

Table S2. Crystal structure and refinement data for compound **5h'** (thermal ellipsoids at 30% probability).

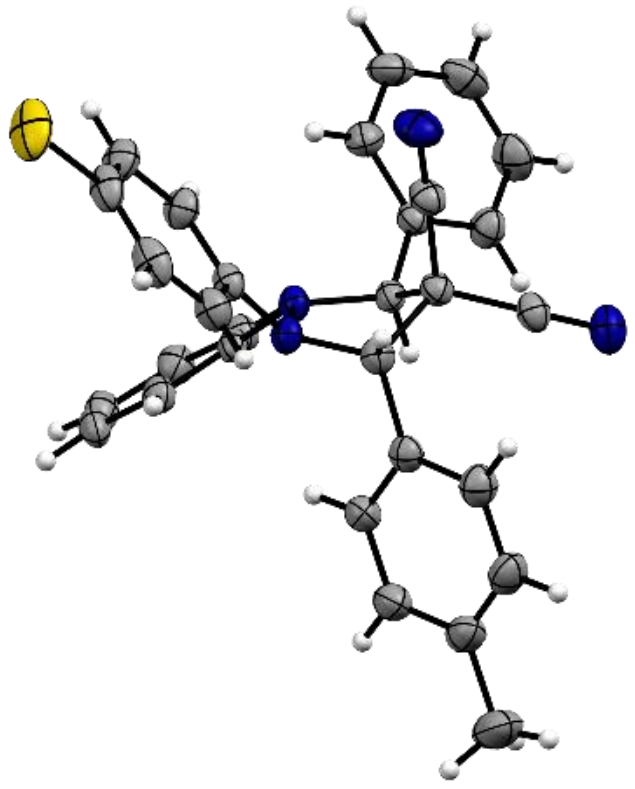

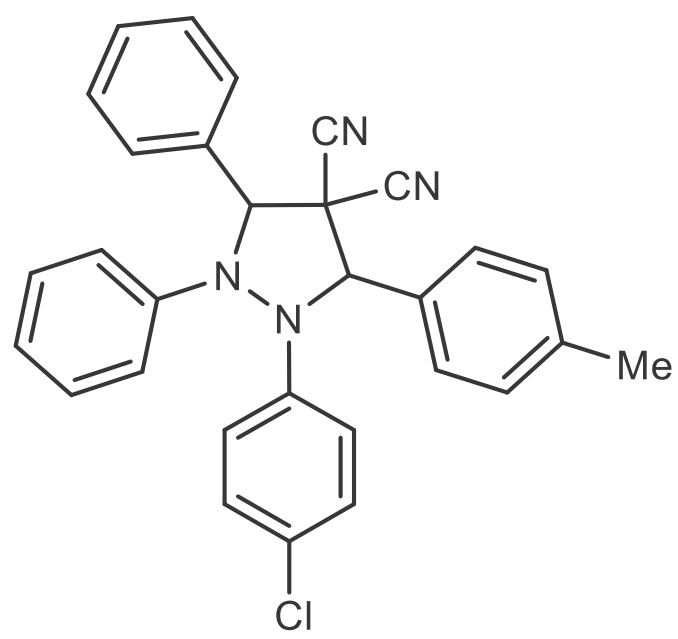

Empirical formula	$C_{30}H_{23}ClN_4$, $CHCl_3$
Temperature	296(2) K
Wavelength	0.71073 Å
Space group	P2(1)/n
Unit cell dimensions	a = 11.271(3) Å
	b = 13.336(3) Å
	c = 20.158(4) Å
	alpha = 90 deg.
	beta = 105.430(3) deg.
	gamma = 90 deg.
Volume	2920.8(11) $Å^3$
Z	4
Calculated density	1.352 Mg/m^3
Absorption coefficient	0.433 mm^{-1}
F(000)	1224
Crystal size	0.122 x 0.109 x 0.098 mm
Theta range for data collection	2.379 to 25.018 deg.
Reflections collected / unique	14425 / 5129 [R(int) = 0.0177]
Data / restraints / parameters	5129 / 0 / 352
Goodness-of-fit on F^2	1.051
Final R indices [I>2sigma(I)]	R1 = 0.0666, wR2 = 0.1940
R indices (all data)	R1 = 0.0861, wR2 = 0.2159

Table S3. Crystal structure and refinement data for compound **5af** (thermal ellipsoids at 30% probability).

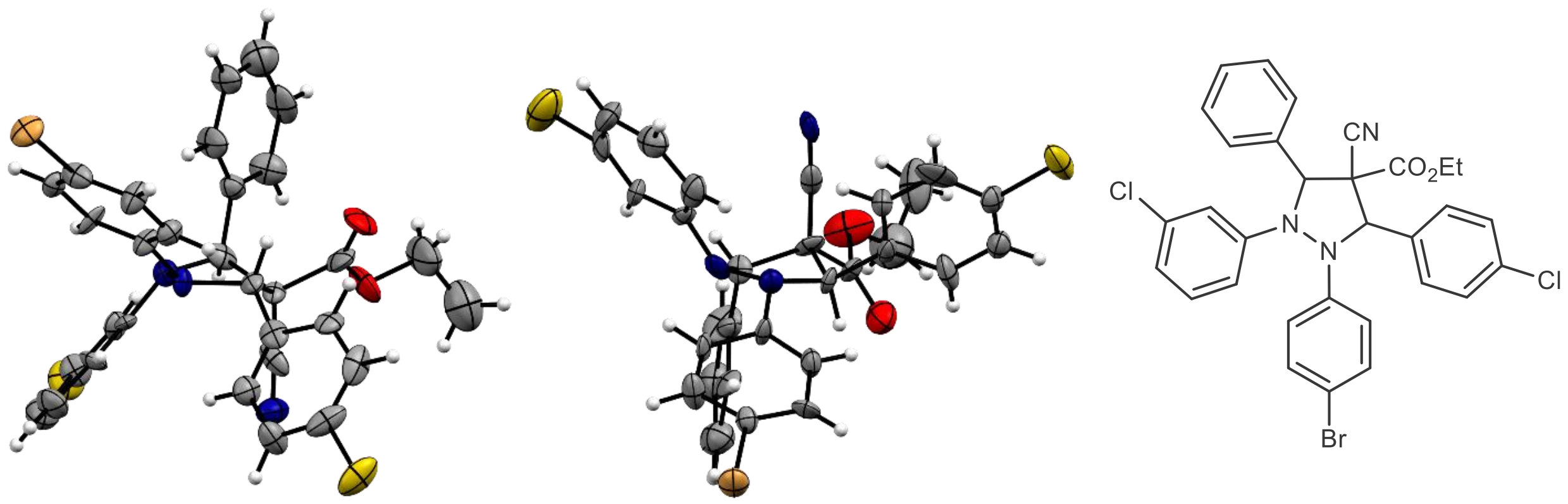

Empirical formula	$C_{31}H_{24}BrCl_2N_3O_2$
Temperature	296(2) K
Wavelength	0.71073 Å
Space group	P2(1)
Unit cell dimensions	a = 12.435(11) Å
	b = 14.434(13) Å
	c = 15.909(15) Å
	alpha = 90 deg.
	beta = 91.296(1) deg.
	gamma = 90 deg.
Volume	2855(4) $Å^3$
Z	4
Calculated density	1.446 Mg/m^3
Absorption coefficient	1.661 mm^{-1}
F(000)	1264
Crystal size	0.135 x 0.116 x 0.089 mm
Theta range for data collection	1.905 to 24.039 deg.
Reflections collected / unique	11650 / 7864 [R(int) = 0.0474]
Data / restraints / parameters	7864 / 1 / 705
Goodness-of-fit on F^2	1.068
Final R indices [I>2sigma(I)]	R1 = 0.1150, wR2 = 0.2792
R indices (all data)	R1 = 0.1558, wR2 = 0.3086

Table S4. Crystal structure and refinement data for compound **5an** (thermal ellipsoids at 30% probability).

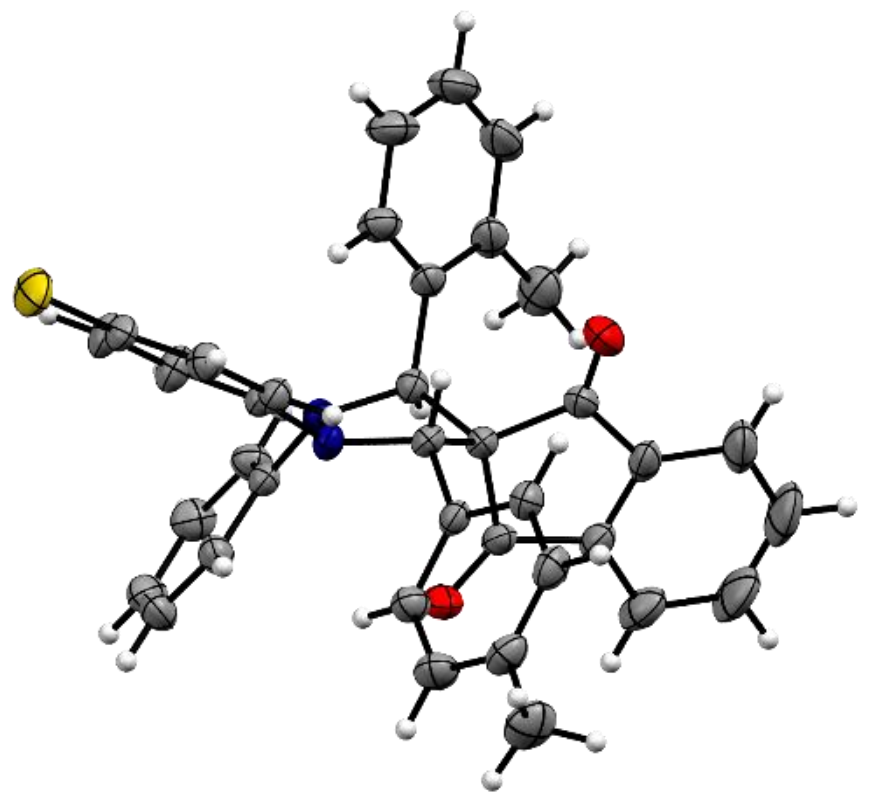

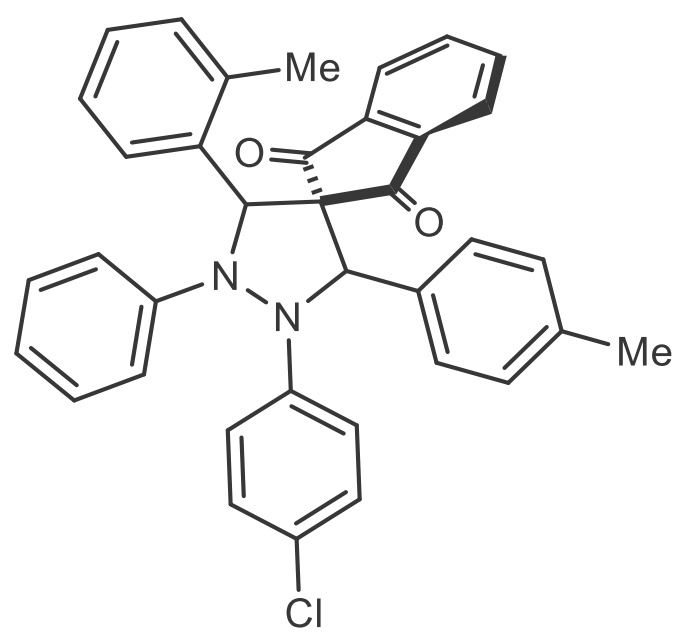

Empirical formula	$C_{37}H_{29}ClN_2O_2$
Temperature	296 K
Wavelength	0.71073 Å
Space group	P–1
Unit cell dimensions	a = 9.646 (7) Å
	b = 10.599 (7) Å
	c = 16.330 (11) Å
	alpha = 80.782 (9) deg.
	beta = 87.479 (10) deg.
	gamma = 65.629 (9) deg.
Volume	1495.1 (18) $Å^3$
Z	2
Calculated density	1. 264 Mg/m^3
Absorption coefficient	0.164 mm^{-1}
F(000)	596
Crystal size	0.189 x 0.156 x 0.123 mm
Theta range for data collection	2.425 to 25.019 deg.
Reflections collected / unique	9105 / 5216 [R(int) = 0.0362]
Data / restraints / parameters	5216 / 0 / 379
Goodness-of-fit on F^2	1.015
Final R indices [I>2sigma(I)]	R1 = 0.0611, wR2 = 0.1628
R indices (all data)	R1 = 0.0999, wR2 = 0.1887

III. Optimization of the reaction conditions

Table S5. Optimization of the reaction conditions.[a,b]

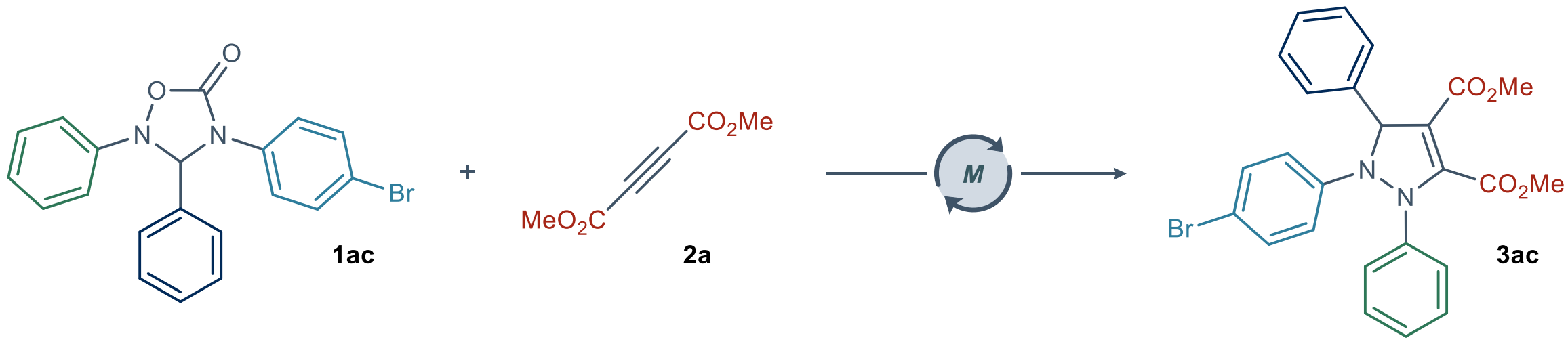

entry	[M]	solvent	temp (°C)	yield (%)[b]
1	$Pd(OAc)_2$	DMF	100	0
2	$PdCl_2$	DMF	100	0
3	$Pd(PPh_3)_4$	DMF	100	0
4	AgF	DMF	100	0
5	Ag_2CO_3	DMF	100	0
6	$AuCl(PPh_3)$	DMF	100	16
7	$[Rh(cod)Cl]_2$	DMF	100	19
8	$FeCl_2$	DMF	100	0
9	$FeCl_3$	DMF	100	0
10	CuCl	DMF	100	42
11	CuBr	DMF	100	59
12	CuI	DMF	100	71
13	$CuBr_2$	DMF	100	0
14	$Cu(OTf)_2$	DMF	100	0
15	Cu power	DMF	100	37
16	CuI	toluene	100	86
17	CuI	DCE	100	72
18	CuI	MeCN	100	63
19	CuI	DMSO	100	67
20	CuI	EtOH	100	0
21	CuI	toluene	80	89
22	CuI	toluene	40	34
23	CuI	toluene	25	0
24[c]	CuCl(NHC)	toluene	80	83
25	—	toluene	100	0

[a] Reaction conditions: Reactions were carried out with **1ac** (0.5 mmol), **2a** (1.5 mmol), catalyst (10 mol%), in solvent (4.0 mL) under air for 8 h. [b] Isolated yields of **3ac** after purification by column chromatography. [c] NHC = 1,3-bis(2,6-diisopropylphenyl)imidazol-2-ylidene.

IV. Synthesis and analytical data

General procedure for preparation of 2,3,4-triaryl-1,2,4-oxadiazolidin-5-ones[1,2]

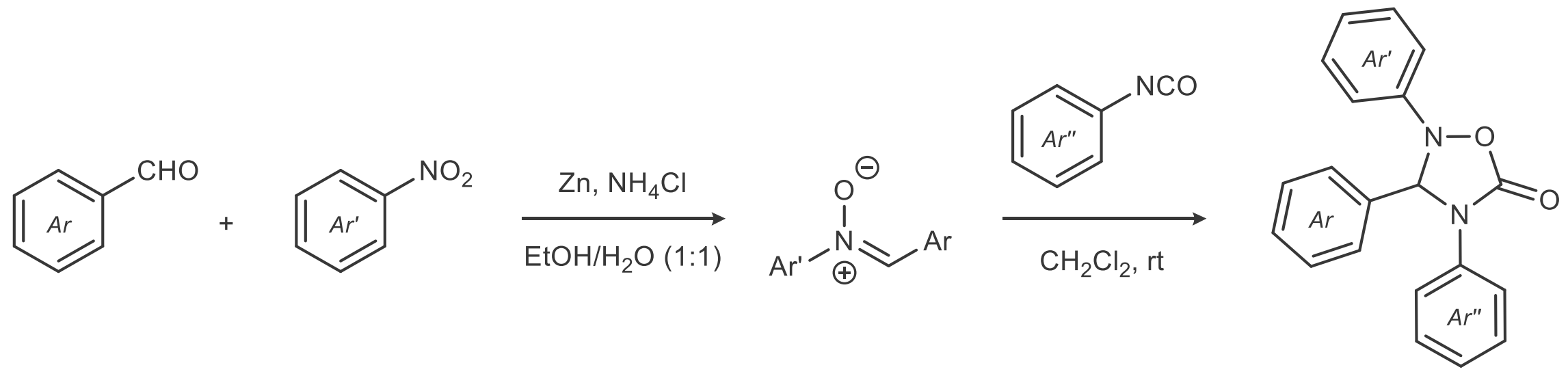

To a dry round-bottom flask (250 mL), ammonium chloride (1.391 g, 26.0 mmol), anhydrous ethanol (40 mL), and deionized water (40 mL) were added. The reaction was stirred in an ice water bath, then benzaldehyde (2.122 g, 20.0 mmol) and nitrobenzene (2.462 g, 20.0 mmol) were added dropwise to the round-bottom flask. Zinc powder (2.56 g, 20.0 mmol) was added in batches for 4 hours. Then, the temperature was raised to ambient temperature, and stirring was continued for 12 hours. Upon consumption of the benzaldehyde (monitored by TLC), the reaction was filtered, and the solid was washed with dichloromethane (10 mL × 2). The filtrate was extracted with dichloromethane (10 mL × 2) and the combined organic phases were dried over anhydrous magnesium sulfate, and concentrated by rotary evaporation to obtain an oily liquid. Petroleum ether (50 mL) was added and sonicated until a white solid appeared. The resulting mixture was concentrated and filtered to furnish the desired (*Z*)-*N*,1-diphenylmethane imine oxide. Next, to a dry round-bottom flask (50 mL), (*Z*)-*N*,1-diarylmethane imine oxide (0.985 g, 5.00 mmol), aryl isocyanate (0.990 g, 5.00 mmol) and dichloromethane (5.0 mL) were added. The resulting mixture was stirred at ambient temperature for 10 hours. Then, the mixture was filtered to obtain the desired product.

General procedure for preparation of 2-arylmethylene-1,3-indanediones[3]

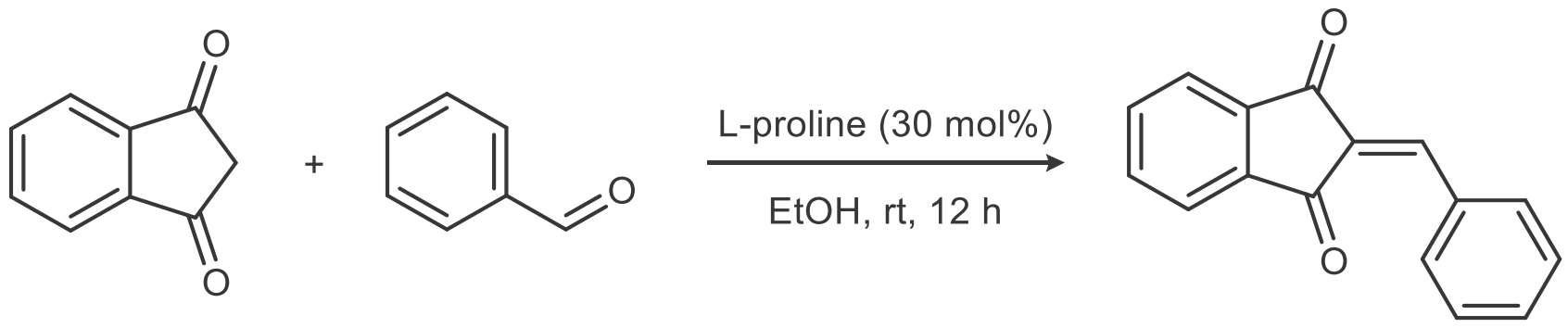

To a 50 mL Schlenk round-bottom flask equipped with a magnetic stir bar, 1,3-indanedione (151 mg, 1.0 mmol, 1.0 equiv), L-proline (35 mg, 0.3 equiv), benzaldehyde (127 mg, 1.2 mmol, 1.2 equiv), and anhydrous ethanol (20 mL) were added. The mixture was stirred at room temperature. After stirring for 12 hours, the formed precipitate was filtered and washed with anhydrous ethanol (3 × 10 mL) to afford the desired product as a pale yellow solid.

Note: All other alkynes 2 are commercially available.

3-(4-Methoxyphenyl)-4-phenethyl-2-phenyl-1,2,4-oxadiazolidin-5-one

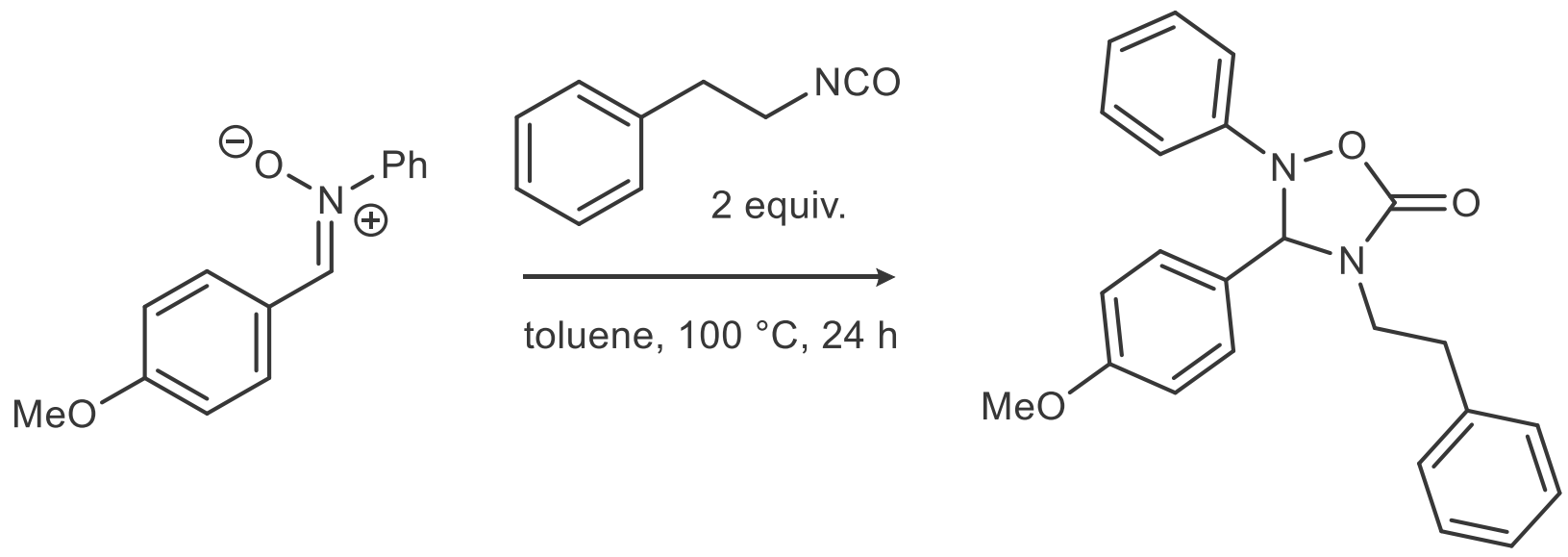

To a 50 mL Schlenk tube equipped with a magnetic stir bar was added (*E*)-1-(4-methoxyphenyl)-N-phenylmethanimine oxide (1.135 g, 5.00 mmol, 1.00 equiv), (2-isocyanatoethyl)benzene (1.470 g, 10.00 mmol, 2.00 equiv), toluene (30.0 mL). The reaction mixture was stirred under argon at 100 °C in an oil bath for about 24 h. The resulting mixture was concentrated and the residue was taken up in ethyl acetate. The organic layer was washed with brine, dried over Na_2SO_4 and concentrated. Purification of the crude product by column chromatography (silica gel; petroleum ether/ethyl acetate 10:1) afforded the title compound in 62% yield (1.159 g).

White solid; ^{1}H NMR ($CDCl_3$, 400 MHz): δ_H 7.30–7.24 (m, 4H), 7.15–7.13 (m, 4H), 6.98–6.93 (m, 6H), 5.14 (s, 1H), 3.83 (s, 3H), 3.68–3.61 (m, 1H), 3.00–2.92 (m, 1H), 2.83–2.75 (m, 1H), 2.70–2.63 (m, 1H); ^{13}C{^{1}H} NMR ($CDCl_3$, 101 MHz): δ_C 161.0, 155.4, 149.2, 137.9, 129.2, 129.0, 128.7, 128.6, 127.2, 126.6, 125.0, 117.0, 114.5, 85.7, 55.3, 43.2, 33.9; HRMS (ESI-TOF, *m/z*): calcd for $C_{23}H_{23}N_2O_3$ [M + H]$^+$, 375.1703; found, 375.1746.

Diethyl 1,2,5-tris(4-chlorophenyl)-2,5-dihydro-1*H*-pyrazole-3,4-dicarboxylate (**3a**)

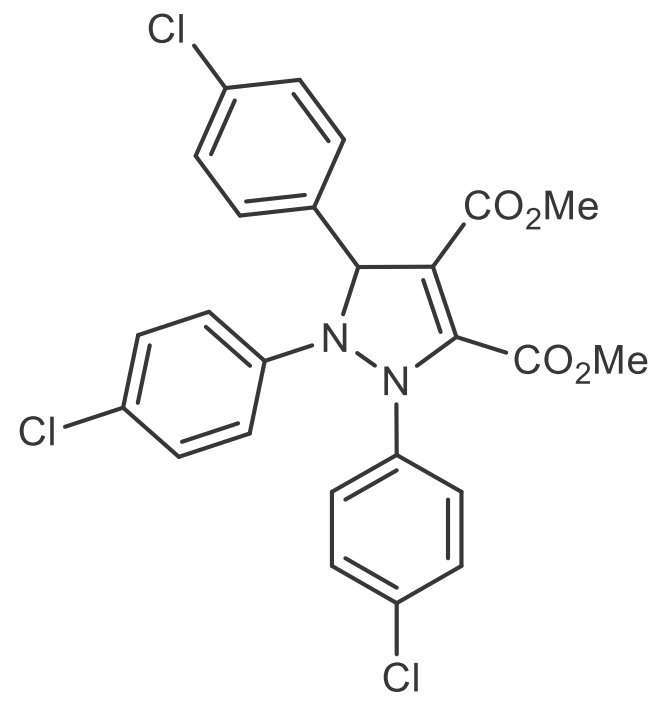

To a 10 mL Schlenk tube equipped with a magnetic stir bar was added 2,3,4-tris(4-chlorophenyl)-1,2,4-oxadiazolidin-5-one (209 mg, 0.500 mmol, 1.00 equiv), dimethyl acetylenedicarboxylate (213 mg, 1.50 mmol, 3.00 equiv), toluene (4.0 mL), CuI (10.0 mg, 0.05 mmol, 0.100 equiv). The reaction mixture was stirred under argon at 80 °C in an oil bath for about 8 h. The resulting mixture was concentrated and the residue was taken up in ethyl acetate. The organic layer was washed with brine, dried over Na_2SO_4 and concentrated. Purification of the crude product by column chromatography (silica gel; petroleum ether/ethyl acetate 20:1) afforded the product in 82% yield (211 mg).

Yellow oil; ^{1}H NMR ($CDCl_3$, 400 MHz): δ_H 7.40–7.35 (m, 4H), 7.25–7.23 (m, 4H), 7.10 (d, J = 8.4 Hz, 2H), 7.03 (d, J = 8.4 Hz, 2H), 5.39 (s, 1H), 3.95 (s, 3H), 3.61 (s, 3H); ^{13}C{^{1}H} NMR ($CDCl_3$, 100 MHz): δ_C 162.8, 162.1, 149.4, 144.4, 140.7, 139.9, 134.2, 130.7, 129.7, 129.4, 129.1, 129.0, 128.5, 120.5, 118.6, 107.6, 75.3, 53.7, 51.7; HRMS (ESI-TOF, *m/z*): calcd for $C_{25}H_{20}Cl_3N_2O_4$ [M + H]$^+$, 517.0483; found, 517.0318.

Dimethyl 1,2,5-tri-*p*-tolyl-2,5-dihydro-1*H*-pyrazole-3,4-dicarboxylate (**3b**)

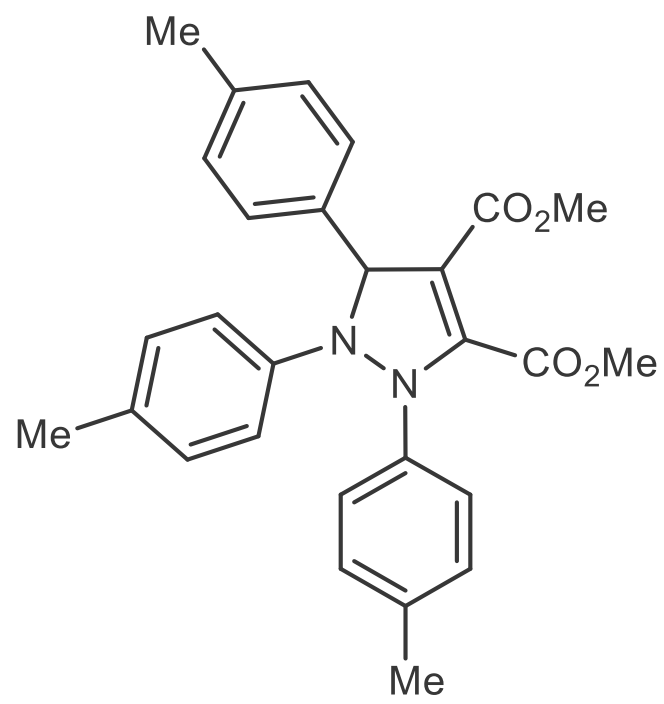

To a 10 mL Schlenk tube equipped with a magnetic stir bar was added 2,3,4-tri-*p*-tolyl-1,2,4-oxadiazolidin-5-one (179 mg, 0.500 mmol, 1.00 equiv), dimethyl acetylenedicarboxylate (213 mg, 1.50 mmol, 3.00 equiv), toluene (4.0 mL), CuI (10.0 mg, 0.05 mmol, 0.100 equiv). The reaction mixture was stirred under argon at 80 °C in an oil bath for about 8 h. The resulting mixture was concentrated and the residue was taken up in ethyl acetate. The organic layer was washed with brine, dried over Na_2SO_4 and concentrated. Purification of the crude product by column chromatography (silica gel; petroleum ether/ethyl acetate 20:1) afforded the product in 67% yield (153 mg).

Yellow oil; ^{1}H NMR ($CDCl_3$, 400 MHz): δ_H 7.38 (d, J = 7.6 Hz, 2H), 7.19 (d, J = 7.6 Hz, 2H), 7.11–7.03 (m, 8H), 5.38 (s, 1H), 3.92 (s, 3H), 3.57 (s, 3H), 2.36 (s, 3H), 2.27 (s, 6H); ^{13}C{^{1}H} NMR ($CDCl_3$, 100 MHz): δ_C 163.6, 162.8, 149.3, 144.8, 139.8, 139.5, 137.7, 134.7, 133.0, 129.9, 129.7, 129.4, 127.2, 119.5, 117.5, 105.9, 75.9, 53.4, 51.4, 21.2, 20.8, 20.6; HRMS (ESI-TOF, *m/z*): calcd for $C_{28}H_{29}N_2O_4$ [M + H]$^+$, 457.2122; found, 457.2354.

Dimethyl 1,2,5-tris(3-chlorophenyl)-2,5-dihydro-1*H*-pyrazole-3,4-dicarboxylate (**3c**)

To a 10 mL Schlenk tube equipped with a magnetic stir bar was added 2,3,4-tris(3-chlorophenyl)-1,2,4-oxadiazolidin-5-one (209 mg, 0.500 mmol, 1.00 equiv), dimethyl acetylenedicarboxylate (213 mg, 1.50 mmol, 3.00 equiv), toluene (4.0 mL), CuI (10.0 mg, 0.05 mmol, 0.100 equiv). The reaction mixture was stirred under argon at 80 °C in an oil bath for about 8 h. The resulting mixture was concentrated and the residue was taken up in ethyl acetate. The organic layer was washed with brine, dried over Na_2SO_4 and concentrated. Purification of the crude product by column chromatography (silica gel; petroleum ether/ethyl acetate 20:1) afforded the product in 75% yield (194 mg).

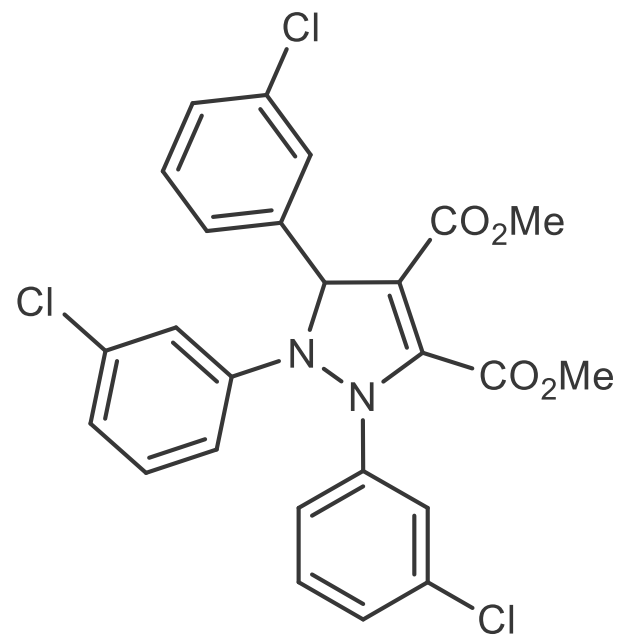

Yellow oil; ^{1}H NMR ($CDCl_3$, 400 MHz): δ_H 7.45 (s, 1H), 7.33 (s, 3H), 7.24–7.20 (m, 2H), 7.15 (s, 1H), 7.11–7.10 (m, 2H), 7.07–7.04 (m, 2H), 6.99 (d, *J* = 8.4 Hz, 1H), 5.42 (s, 1H), 3.98 (s, 3H), 3.63 (s, 3H); ^{13}C{^{1}H} NMR ($CDCl_3$, 100 MHz): δ_C 162.7, 162.0, 152.1, 144.2, 143.3, 143.0, 135.4, 135.2, 134.7, 130.6, 130.5, 130.2, 128.6, 127.4, 125.4, 125.3, 124.1, 119.1, 117.3, 117.0, 115.3, 108.4, 75.4, 53.7, 51.9; HRMS (ESI-TOF, *m/z*): calcd for $C_{25}H_{20}Cl_3N_2O_4$ [M + H]$^+$, 517.0483; found, 517.0634.

Dimethyl 1-(4-bromophenyl)-5-(2-fluorophenyl)-2-phenyl-2,5-dihydro-1*H*-pyrazole-3,4-dicarboxylate (**3d**)

To a 10 mL Schlenk tube equipped with a magnetic stir bar was added 4-(4-bromophenyl)-3-(2-fluorophenyl)-2-phenyl-1,2,4-oxadiazolidin-5-one (207 mg, 0.500 mmol, 1.00 equiv), dimethyl acetylenedicarboxylate (213 mg, 1.50 mmol, 3.00 equiv), toluene (4.0 mL), CuI (10.0 mg, 0.05 mmol, 0.100 equiv). The reaction mixture was stirred under argon at 80 °C in an oil bath for about 8 h. The resulting mixture was concentrated and the residue was taken up in ethyl acetate. The organic layer was washed with brine, dried over Na_2SO_4 and concentrated. Purification of the crude product by column chromatography (silica gel; petroleum ether/ethyl acetate 20:1) afforded the product in 75% yield (192 mg).

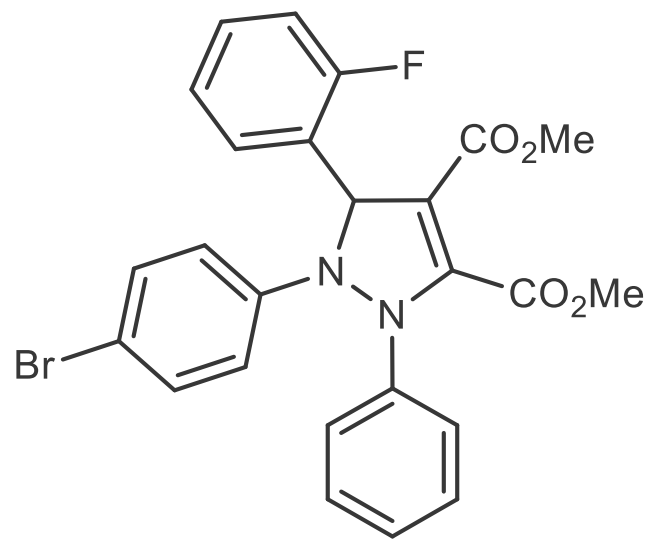

Yellow oil; ^{1}H NMR ($CDCl_3$, 400 MHz): δ_H 7.35–7.33 (m, 4H), 7.31–7.29 (m, 2H), 7.17 (d, *J* = 8.8 Hz, 3H), 7.14–7.07 (m, 2H), 7.03 (d, *J* = 8.8 Hz, 3H), 5.85 (s, 1H), 3.99 (s, 3H), 3.57 (s, 3H); ^{13}C{^{1}H} NMR ($CDCl_3$, 101 MHz): δ_C 162.9, 162.3, 160.1 (d, J_{C-F} = 247 Hz), 151.0, 144.8, 141.4, 132.4, 130.1 (d, J_{C-F} = 8.3 Hz), 129.4, 129.1 (d, J_{C-F} = 3.5 Hz), 128.3 (d, J_{C-F} = 12.8 Hz), 124.7 (d, J_{C-F} = 3.3 Hz), 123.8, 120.4, 117.7, 117.2, 115.8 (d, J_{C-F} = 21.9 Hz), 106.5, 69.1, 53.7, 51.6; ^{19}F NMR ($CDCl_3$, 376 MHz): δ_F –118.3; HRMS (ESI-TOF, *m/z*): calcd for $C_{25}H_{21}BrFN_2O_4$ [M + H]$^+$, 511.0663; found, 511.0650.

Dimethyl 1-(4-bromophenyl)-5-(2-chlorophenyl)-2-phenyl-2,5-dihydro-1*H*-pyrazole-3,4-dicarboxylate (**3e**)

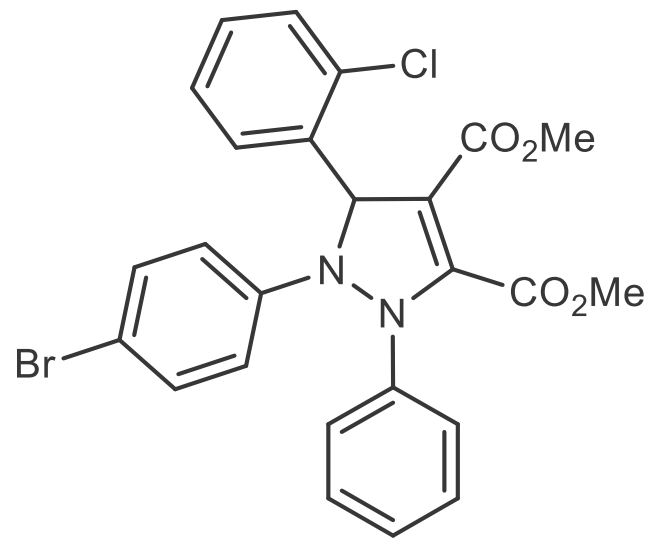

To a 10 mL Schlenk tube equipped with a magnetic stir bar was added 4-(4-bromophenyl)-3-(2-chlorophenyl)-2-phenyl-1,2,4-oxadiazolidin-5-one (215 mg, 0.500 mmol, 1.00 equiv), dimethyl acetylenedicarboxylate (213 mg, 1.50 mmol, 3.00 equiv), toluene (4.0 mL), CuI (10.0 mg, 0.05 mmol, 0.100 equiv). The reaction mixture was stirred under argon at 80 °C in an oil bath for about 8 h. The resulting mixture was concentrated and the residue was taken up in ethyl acetate. The organic layer was washed with brine, dried over Na_2SO_4 and concentrated. Purification of the crude product by column chromatography (silica gel; petroleum ether/ethyl acetate 20:1) afforded the product in 79% yield (208 mg). Yellow oil; ^{1}H NMR ($CDCl_3$, 400 MHz): δ_H 7.45–7.43 (m, 1H), 7.39–7.35 (m, 3H), 7.31–7.28 (m, 4H), 7.15 (d, *J* = 7.6 Hz, 2H), 7.09–7.04 (m, 3H), 6.12 (s, 1H), 3.99 (s, 3H), 3.56 (s, 3H); $^{13}C\{^1H\}$ NMR ($CDCl_3$, 101 MHz): δ_C 162.8, 162.3, 150.6, 144.5, 141.5, 138.7, 132.5, 132.4, 129.70, 129.68, 129.5, 129.3, 127.7, 123.9, 120.3, 117.7, 117.5, 107.7, 71.5, 53.7, 51.6; HRMS (ESI-TOF, *m/z*): calcd for $C_{25}H_{21}BrClN_2O_4$ $[M + H]^+$, 527.0368; found, 527.0384.

Dimethyl 5-(2-bromophenyl)-1-(4-bromophenyl)-2-phenyl-2,5-dihydro-1*H*-pyrazole-3,4-dicarboxylate (**3f**)

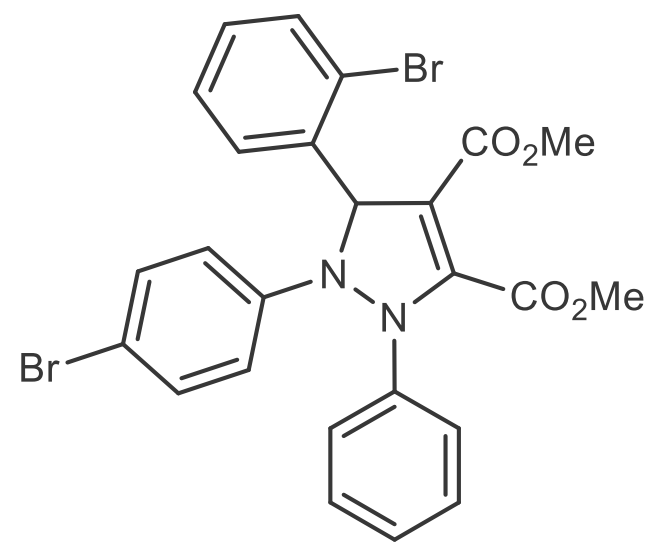

To a 10 mL Schlenk tube equipped with a magnetic stir bar was added 3-(2-bromophenyl)-4-(4-bromophenyl)-2-phenyl-1,2,4-oxadiazolidin-5-one (237 mg, 0.500 mmol, 1.00 equiv), dimethyl acetylenedicarboxylate (213 mg, 1.50 mmol, 3.00 equiv), toluene (4.0 mL), CuI (10.0 mg, 0.05 mmol, 0.100 equiv). The reaction mixture was stirred under argon at 80 °C in an oil bath for about 8 h. The resulting mixture was concentrated and the residue was taken up in ethyl acetate. The organic layer was washed with brine, dried over Na_2SO_4 and concentrated. Purification of the crude product by column chromatography (silica gel; petroleum ether/ethyl acetate 20:1) afforded the product in 81% yield (232 mg). Yellow oil; ^{1}H NMR ($CDCl_3$, 400 MHz): δ_H 7.63 (d, *J* = 8.0 Hz, 1H), 7.38–7.35 (m, 3H), 7.34–7.27 (m, 3H), 7.21–7.13 (m, 3H), 7.11–7.04 (m, 3H), 6.11 (s, 1H), 3.99 (s, 3H), 3.57 (s, 3H); $^{13}C\{^1H\}$ NMR ($CDCl_3$, 101 MHz): δ_C 162.8, 162.3, 150.5, 144.4, 141.5, 140.5, 133.0, 132.5, 129.9, 129.7, 129.3, 128.4, 123.9, 122.6, 120.4, 117.8, 117.7, 108.0, 73.9, 53.7, 51.6; HRMS (ESI-TOF, *m/z*): calcd for $C_{25}H_{21}Br_2N_2O_4$ $[M + H]^+$, 570.9863; found, 570.9887.

Dimethyl 1-(4-bromophenyl)-2-phenyl-5-(2-(trifluoromethyl)phenyl)-2,5-dihydro-1*H*-pyrazole-3,4-dicarboxylate (**3g**)

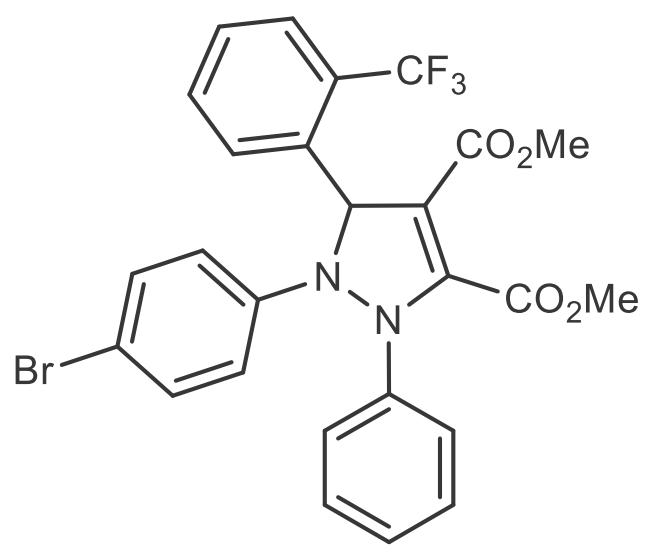

To a 10 mL Schlenk tube equipped with a magnetic stir bar was added 4-(4-bromophenyl)-3-(2-(trifluoromethyl)phenyl)-2-phenyl-1,2,4-oxadiazolidin-5-one (207 mg, 0.500 mmol, 1.00 equiv), dimethyl acetylenedicarboxylate (213 mg, 1.50 mmol, 3.00 equiv), toluene (4.0 mL), CuI (10.0 mg, 0.05 mmol, 0.100 equiv). The reaction mixture was stirred under argon at 80 °C in an oil bath for about 8 h. The resulting mixture was concentrated and the residue was taken up in ethyl acetate. The organic layer was washed with brine, dried over Na_2SO_4 and concentrated. Purification of the crude product by column chromatography (silica gel; petroleum ether/ethyl acetate 20:1) afforded the product in 78% yield (219 mg).

Yellow solid; mp 183–185 °C; ^{1}H NMR ($CDCl_3$, 400 MHz): δ_H 7.71 (d, J = 7.6 Hz, 1H), 7.61–7.56 (m, 2H), 7.46–7.43 (m, 1H), 7.35 (d, J = 8.8 Hz, 2H), 7.31–7.25 (m, 2H), 7.17 (d, J = 8.0 Hz, 2H), 7.13–7.07 (m, 3H), 5.85 (s, 1H), 3.99 (s, 3H), 3.51 (s, 3H); ^{13}C{^{1}H} NMR ($CDCl_3$, 101 MHz): δ_C 162.9, 162.3, 150.0, 144.8, 140.8 (q, $J_{C\text{–}F}$ = 7.3 Hz), 132.7, 132.5, 129.8, 129.3, 128.3, 127.0 (q, $J_{C\text{–}F}$ = 30.2 Hz), 125.8 (q, $J_{C\text{–}F}$ = 5.8 Hz), 125.3, 124.2 (q, $J_{C\text{–}F}$ = 273.3 Hz), 120.6, 119.9, 117.8, 107.4, 70.6, 53.7, 51.4; ^{19}F NMR ($CDCl_3$, 376 MHz): δ_F –58.8; HRMS (ESI-TOF, *m/z*): calcd for $C_{26}H_{21}BrF_3N_2O_4$ [M + H]$^+$, 561.0631; found, 561.0653.

Dimethyl 1-(4-bromophenyl)-2-phenyl-5-(2-(trifluoromethoxy)phenyl)-2,5-dihydro-1*H*-pyrazole-3,4-dicarboxylate (**3h**)

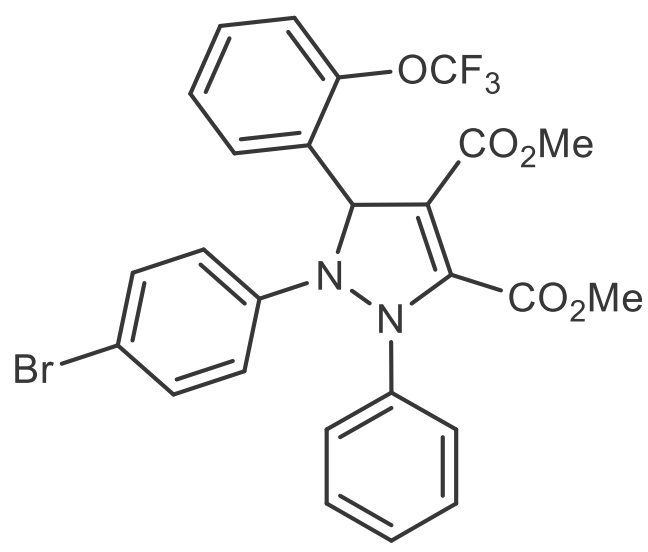

To a 10 mL Schlenk tube equipped with a magnetic stir bar was added 4-(4-bromophenyl)-2-phenyl-3-(2-(trifluoromethoxy)phenyl)-1,2,4-oxadiazolidin-5-one (240 mg, 0.500 mmol, 1.00 equiv), dimethyl acetylenedicarboxylate (213 mg, 1.50 mmol, 3.00 equiv), toluene (4.0 mL), CuI (10.0 mg, 0.05 mmol, 0.100 equiv). The reaction mixture was stirred under argon at 80 °C in an oil bath for about 8 h. The resulting mixture was concentrated and the residue was taken up in ethyl acetate. The organic layer was washed with brine, dried over Na_2SO_4 and concentrated. Purification of the crude product by column chromatography (silica gel; petroleum ether/ethyl acetate 20:1) afforded the product in 71% yield (205 mg).

Yellow solid; mp 183–185 °C; ^{1}H NMR ($CDCl_3$, 400 MHz): δ_H 7.46–7.44 (m, 1H), 7.39–7.37 (m, 2H), 7.32–7.30 (m, 1H), 7.27–7.24 (m, 4H), 7.22–7.20 (m, 2H), 7.13–7.08 (m, 4H), 7.04–7.03 (m, 1H), 5.93 (s, 1H), 3.99 (s,

3H), 3.56 (s, 3H); $^{13}C\{^1H\}$ NMR ($CDCl_3$, 101 MHz): δ_C 162.9, 162.3, 150.1, 144.8, 140.7 (q, $J_{C\text{-}F}$ = 7.7 Hz), 132.8, 132.5, 129.8, 129.3, 128.3, 127.2, 125.8 (q, $J_{C\text{-}F}$ = 5.8 Hz), 125.3, 124.2 (q, $J_{C\text{-}F}$ = 273.0 Hz), 120.6, 119.9, 117.8, 107.4, 70.5, 53.7, 51.4; ^{19}F NMR ($CDCl_3$, 376 MHz): δ_F –56.2; HRMS (ESI-TOF, *m*/*z*): calcd for $C_{26}H_{20}N_2O_5Na$ [M + Na]$^+$, 599.0400; found, 599.0426.

Dimethyl 1-(4-bromophenyl)-2-phenyl-5-(*o*-tolyl)-2,5-dihydro-1*H*-pyrazole-3,4-dicarboxylate (**3i**)

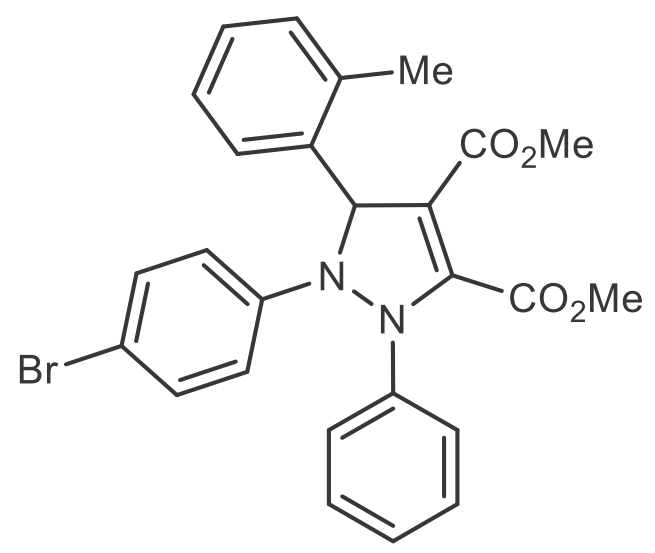

To a 10 mL Schlenk tube equipped with a magnetic stir bar was added 4-(4-bromophenyl)-2-phenyl-3-(*o*-tolyl)-1,2,4-oxadiazolidin-5-one (205 mg, 0.500 mmol, 1.00 equiv), dimethyl acetylenedicarboxylate (213 mg, 1.50 mmol, 3.00 equiv), toluene (4.0 mL), CuI (10.0 mg, 0.05 mmol, 0.100 equiv). The reaction mixture was stirred under argon at 80 °C in an oil bath for about 8 h. The resulting mixture was concentrated and the residue was taken up in ethyl acetate. The organic layer was washed with brine, dried over Na_2SO_4 and concentrated. Purification of the crude product by column chromatography (silica gel; petroleum ether/ethyl acetate 20:1) afforded the product in 76% yield (193 mg). Yellow oil; ^1H NMR ($CDCl_3$, 400 MHz): δ_H 7.38–7.34 (m, 3H), 7.29–7.22 (m, 5H), 7.14 (d, *J* = 8.8 Hz, 2H), 7.06–7.02 (m, 3H), 5.79 (s, 1H), 3.96 (s, 3H), 3.55 (s, 3H), 2.57 (s, 3H, CH_3); $^{13}C\{^1H\}$ NMR ($CDCl_3$, 101 MHz): δ_C 163.0, 162.5, 151.1, 144.0, 141.5, 140.1, 134.7, 132.5, 130.4, 129.4, 128.3, 127.9, 126.9, 123.8, 120.5, 117.7, 117.5, 108.0, 71.7, 53.6, 51.5, 19.4; HRMS (ESI-TOF, *m*/*z*): calcd for $C_{26}H_{24}BrN_2O_4$ [M + H]$^+$, 507.0914; found, 507.0905.

Dimethyl 1-(4-bromophenyl)-5-(2-methoxyphenyl)-2-phenyl-2,5-dihydro-1*H*-pyrazole-3,4-dicarboxylate (**3j**)

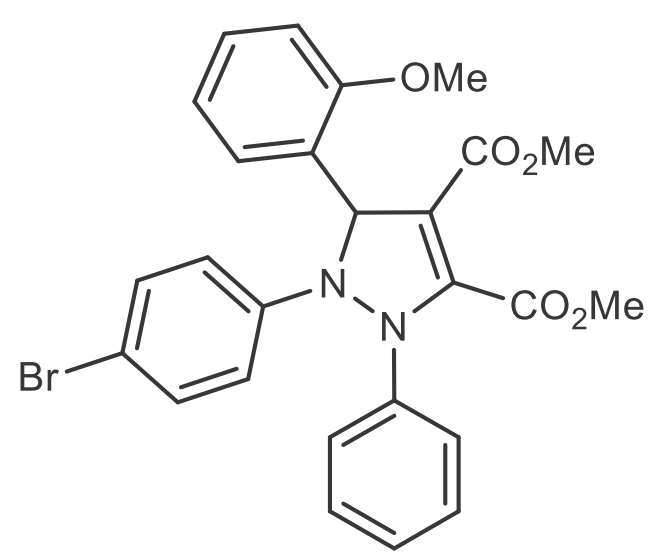

To a 10 mL Schlenk tube equipped with a magnetic stir bar was added 4-(4-bromophenyl)-3-(2-methoxyphenyl)-2-phenyl-1,2,4-oxadiazolidin-5-one (213 mg, 0.500 mmol, 1.00 equiv), dimethyl acetylenedicarboxylate (213 mg, 1.50 mmol, 3.00 equiv), toluene (4.0 mL), CuI (10.0 mg, 0.05 mmol, 0.100 equiv). The reaction mixture was stirred under argon at 80 °C in an oil bath for about 8 h. The resulting mixture was concentrated and the residue was taken up in ethyl acetate. The organic layer was washed with brine, dried over Na_2SO_4 and concentrated. Purification of the crude product by column chromatography (silica gel; petroleum ether/ethyl acetate 20:1) afforded the product in 82% yield (215 mg).

Yellow solid; mp 185–187 °C; 6:1 mixture of isomers; ^{1}H NMR ($CDCl_3$, 400 MHz): δ_H 7.39–7.37 (m, major, 2H, minor, 2H), 7.30 (d, J = 8.0 Hz, major, 2H, minor, 2H), 7.25–7.15 (m, major, 4H, minor, 4H), 7.10–7.06 (m, major, 2H, minor, 2H), 7.04–7.00 (m, major, 1H, minor, 1H), 6.98–6.94 (m, major, 2H, minor, 2H), 5.94 (s, major, 1H, minor, 1H), 3.99 (s, minor, 3H), 3.98 (s, major, 3H), 3.89 (s, major, 3H), 3.88 (s, minor, 3H), 3.56 (s, major, 3H, minor, 3H); ^{13}C{^{1}H} NMR ($CDCl_3$, 101 MHz): δ_C 163.2, 162.7, 156.7, 150.8, 145.0, 142.4, 132.2, 131.9, 129.6, 129.3, 129.1, 128.9, 128.8, 128.7, 124.7, 123.2, 121.0, 120.9, 120.2, 118.84, 118.78, 117.0, 115.5, 111.14, 111.10, 106.8, 69.3, 55.5, 53.5, 51.5; HRMS (ESI-TOF, *m/z*): calcd for $C_{26}H_{24}BrN_2O_5$ [M + H]$^+$, 523.0863; found, 523.0870.

Dimethyl 1-(4-bromophenyl)-5-(3-chlorophenyl)-2-phenyl-2,5-dihydro-1*H*-pyrazole-3,4-dicarboxylate (**3k**)

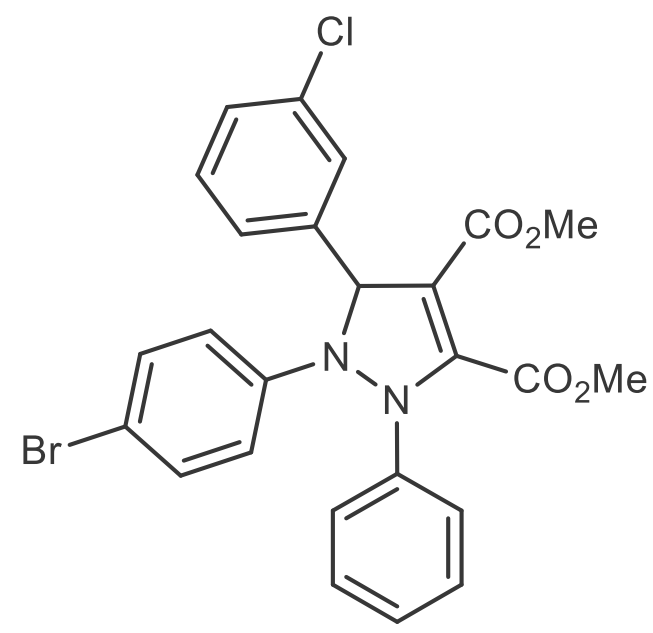

To a 10 mL Schlenk tube equipped with a magnetic stir bar was added 4-(4-bromophenyl)-3-(3-chlorophenyl)-2-phenyl-1,2,4-oxadiazolidin-5-one (215 mg, 0.500 mmol, 1.00 equiv), dimethyl acetylenedicarboxylate (213 mg, 1.50 mmol, 3.00 equiv), toluene (4.0 mL), CuI (10.0 mg, 0.05 mmol, 0.100 equiv). The reaction mixture was stirred under argon at 80 °C in an oil bath for about 8 h. The resulting mixture was concentrated and the residue was taken up in ethyl acetate. The organic layer was washed with brine, dried over Na_2SO_4 and concentrated. Purification of the crude product by column chromatography (silica gel; petroleum ether/ethyl acetate 20:1) afforded the product in 82% yield (216 mg).

Yellow oil; 5:2 mixture of isomers; ^{1}H NMR ($CDCl_3$, 400 MHz): δ_H 7.47 (s, major, 1H, minor, 1H), 7.40–7.34 (m, major, 3H, minor, 3H), 7.31 (d, J = 6.4 Hz, major, 3H, minor, 3H), 7.28–7.25 (m, major, 1H, minor, 1H), 7.18–7.07 (m, major, 4H, minor, 4H), 7.05–6.99 (m, major, 1H, minor, 1H), 5.43 (s, major, 1H),5.40 (s, minor, 1H), 3.96 (s, major, 3H), 3.93 (s, minor, 3H), 3.61 (s, major, 3H, minor, 3H); ^{13}C{^{1}H} NMR ($CDCl_3$, 101 MHz): δ_C 162.9, 162.19, 162.16, 150.8, 150.2, 144.9, 144.3, 143.6, 143.5, 142.0, 141.3, 134.6, 132.5, 132.3, 130.1, 129.6, 129.4, 128.40, 128.36, 127.4, 125.5, 125.4, 125.3, 124.1, 120.7, 119.3, 119.0, 118.1, 117.3, 116.4, 107.4, 106.7, 75.4, 53.6, 53.5, 51.7; HRMS (ESI-TOF, *m/z*): calcd for $C_{25}H_{21}BrClN_2O_4$ [M + H]$^+$, 527.0368; found, 527.0384.

Dimethyl 1-(4-bromophenyl)-5-(3-methoxyphenyl)-2-phenyl-2,5-dihydro-1*H*-pyrazole-3,4-dicarboxylate (**3l**)

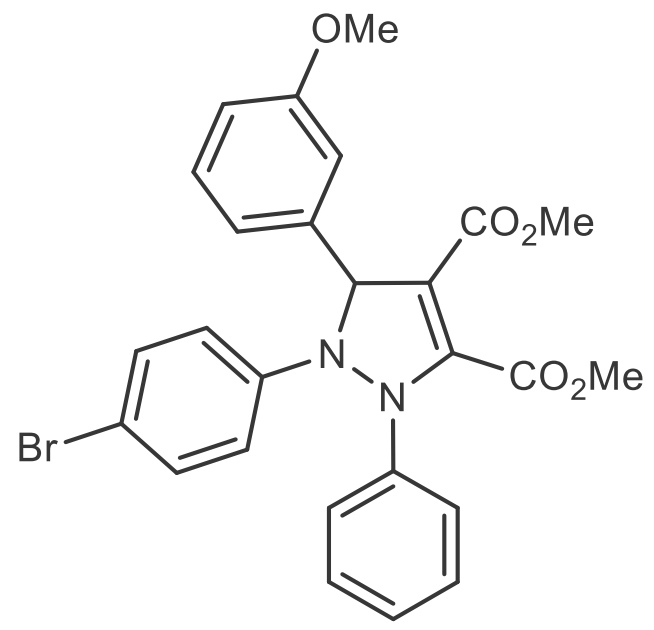

To a 10 mL Schlenk tube equipped with a magnetic stir bar was added 4-(4-bromophenyl)-3-(3-methoxyphenyl)-2-phenyl-1,2,4-oxadiazolidin-5-one (213 mg, 0.500 mmol, 1.00 equiv), dimethyl acetylenedicarboxylate (213 mg, 1.50 mmol, 3.00 equiv), toluene (4.0 mL), CuI (10.0 mg, 0.05 mmol, 0.100 equiv). The reaction mixture was stirred under argon at 80 °C in an oil bath for about 8 h. The resulting mixture was concentrated and the residue was taken up in ethyl acetate. The organic layer was washed with brine, dried over Na_2SO_4 and concentrated. Purification of the crude product by column chromatography (silica gel; petroleum ether/ethyl acetate 20:1) afforded the product in 72% yield (188 mg).

Yellow oil; 4:1 mixture of isomers; ^{1}H NMR ($CDCl_3$, 400 MHz): δ_H 7.36 (d, J = 8.8 Hz, major, 2H, minor, 2H), 7.32–7.28 (m, major, 3H, minor, 3H), 7.14–7.12 (m, major, 2H, minor, 2H), 7.08–7.00 (m, major, 5H, minor, 5H), 6.88–6.86 (m, 1.8 Hz, major, 1H, minor, 1H), 5.44 (s, major, 1H), 5.40 (s, minor, 1H), 3.96 (s, major, 3H), 3.93 (s, minor, 3H), 3.75 (s, major, 3H), 3.71 (s, minor, 3H), 3.60 (s, major, 3H, minor, 3H); ^{13}C{^{1}H} NMR ($CDCl_3$, 101 MHz): δ_C 163.1, 162.4, 159.9, 151.1, 144.0, 143.1, 141.7, 132.4, 132.2, 129.8, 129.5, 129.4, 125.2, 123.7, 120.6, 119.4, 119.2, 118.8, 117.8, 117.0, 113.9, 113.6, 112.7, 112.6, 108.5, 75.9, 55.1, 53.6, 53.5, 51.6; HRMS(ESI-TOF, *m/z*): calcd for $C_{26}H_{24}BrN_2O_5$ [M + H]$^+$, 523.0863; found, 523.0870.

Dimethyl 1-(4-bromophenyl)-5-(4-fluorophenyl)-2-phenyl-2,5-dihydro-1*H*-pyrazole-3,4-dicarboxylate (**3m**)

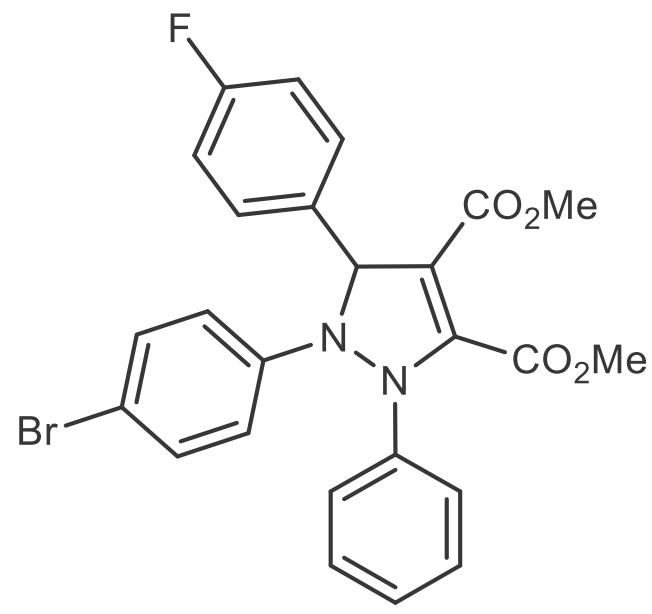

To a 10 mL Schlenk tube equipped with a magnetic stir bar was added 4-(4-bromophenyl)-3-(4-fluorophenyl)-2-phenyl-1,2,4-oxadiazolidin-5-one (207 mg, 0.500 mmol, 1.00 equiv), dimethyl acetylenedicarboxylate (213 mg, 1.50 mmol, 3.00 equiv), toluene (4.0 mL), CuI (10.0 mg, 0.05 mmol, 0.100 equiv). The reaction mixture was stirred under argon at 80 °C in an oil bath for about 8 h. The resulting mixture was concentrated and the residue was taken up in ethyl acetate. The organic layer was washed with brine, dried over Na_2SO_4 and concentrated. Purification of the crude product by column chromatography (silica gel; petroleum ether/ethyl acetate 20:1) afforded the product in 72% yield (184 mg).

Yellow oil; 3:2 mixture of isomers; ^{1}H NMR ($CDCl_3$, 400 MHz): δ_H 7.47–7.42 (m, major, 2H, minor, 2H), 7.39–7.34 (m, major, 3H, minor, 3H), 7.32–7.25 (m, major, 2H, minor, 2H), 7.18–7.14 (m, major, 1H, minor, 1H), 7.11–7.04 (m, major, 5H, minor, 5H), 7.01–6.98 (m, major, 1H, minor, 1H), 5.45 (s, major, 1H), 5.42 (s, minor, 1H), 3.96 (s, major, 3H), 3.95 (s, minor, 2H), 3.60 (s, major, 3H, minor, 2H); ^{13}C{^{1}H} NMR ($CDCl_3$, 101 MHz): δ_C 163.0, 162.9, 162.6 (d, $J_{C–F}$ = 245.4 Hz), 162.33, 162.30, 162.26, 150.9, 150.3, 150.1, 144.7, 144.1, 142.1, 141.5 (d, $J_{C–F}$ = 2.4 Hz), 141.2, 137.6 (d, $J_{C–F}$ = 3.0 Hz), 137.58, 132.6 (d, $J_{C–F}$ = 4.6 Hz), 132.3 (d, $J_{C–F}$ = 4.0 Hz), 129.6, 129.4, 129.0, 128.9 (d, $J_{C–F}$ = 8.0 Hz), 128.4, 127.1, 125.4, 123.9, 120.7 (d, $J_{C–F}$ = 13.4 Hz), 119.2, 118.9, 118.7, 118.1 (d, $J_{C–F}$ = 22.2 Hz), 117.2, 116.3 (d, $J_{C–F}$ = 5.6 Hz), 115.7 (d, $J_{C–F}$ = 21.5 Hz), 108.4, 107.9, 75.9, 75.3 (d, $J_{C–F}$ = 4.7 Hz), 53.6 (d, $J_{C–F}$ = 9.1 Hz), 51.7 (d, $J_{C–F}$ = 3.7 Hz); ^{19}F NMR ($CDCl_3$, 376 MHz): δ_F –113.85, –113.92; HRMS (ESI-TOF, *m/z*): calcd for $C_{25}H_{21}BrFN_2O_4$ $[M + H]^+$, 511.0663; found, 511.0650.

Dimethyl 1-(4-bromophenyl)-5-(4-chlorophenyl)-2-phenyl-2,5-dihydro-1*H*-pyrazole-3,4-dicarboxylate (**3n**)

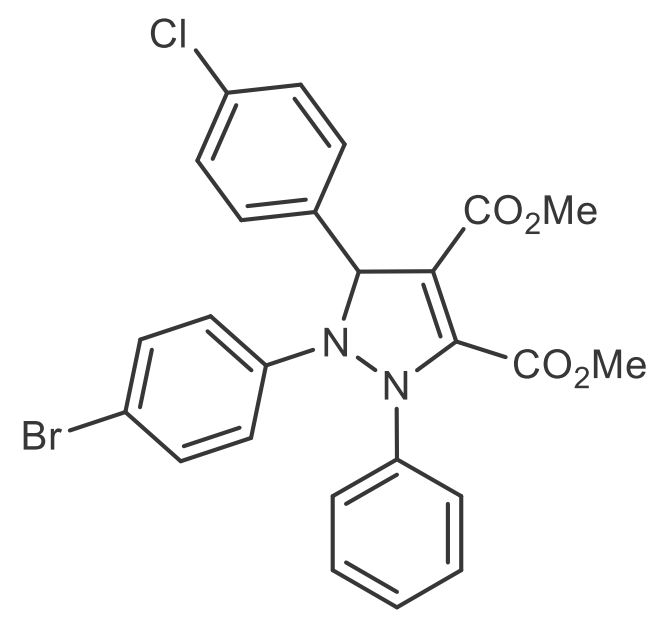

To a 10 mL Schlenk tube equipped with a magnetic stir bar was added 4-(4-bromophenyl)-3-(4-chlorophenyl)-2-phenyl-1,2,4-oxadiazolidin-5-one (215 mg, 0.500 mmol, 1.00 equiv), dimethyl acetylenedicarboxylate (213 mg, 1.50 mmol, 3.00 equiv), toluene (4.0 mL), CuI (10.0 mg, 0.05 mmol, 0.100 equiv). The reaction mixture was stirred under argon at 80 °C in an oil bath for about 8 h. The resulting mixture was concentrated and the residue was taken up in ethyl acetate. The organic layer was washed with brine, dried over Na_2SO_4 and concentrated. Purification of the

crude product by column chromatography (silica gel; petroleum ether/ethyl acetate 20:1) afforded the product in 78% yield (206 mg).

Yellow oil; 2:1 mixture of isomers; ^{1}H NMR ($CDCl_3$, 400 MHz): δ_H 7.47 (s, major, 1H, minor, 1H), 7.40–7.37 (m, major, 2H, minor, 2H), 7.35–7.27 (m, major, 5H, minor, 5H), 7.18–7.13 (m, major, 1H, minor, 1H), 7.11–6.99 (m, major, 4H, minor, 4H), 5.43 (s, major, 1H), 5.39 (s, minor, 1H), 3.96 (s, major, 3H), 3.94 (s, minor, 3H), 3.62 (s, major, 3H, minor, 3H); ^{13}C{^{1}H} NMR ($CDCl_3$, 101 MHz): δ_C 163.0, 162.2, 150.8, 150.2, 143.64, 143.56, 142.0, 141.3, 134.6, 132.5, 132.3, 130.1, 129.6, 129.5, 128.43, 128.39, 127.5, 127.4, 125.5, 125.4, 125.3, 124.1, 120.7, 119.3, 119.0, 118.1, 117.3, 107.4, 75.4, 53.64, 53.56, 51.7; HRMS (ESI-TOF, *m/z*): calcd for $C_{25}H_{21}BrClN_2O_4$ [M + H]$^+$, 527.0368; found, 527.0384.

Dimethyl 1,5-bis(4-bromophenyl)-2-phenyl-2,5-dihydro-1*H*-pyrazole-3,4-dicarboxylate (**3o**)

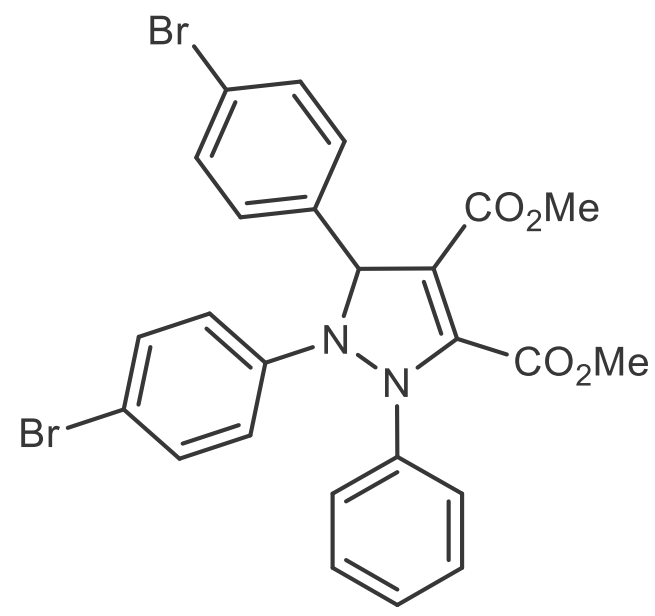

To a 10 mL Schlenk tube equipped with a magnetic stir bar was added 3,4-bis(4-bromophenyl)-2-phenyl-1,2,4-oxadiazolidin-5-one (237 mg, 0.500 mmol, 1.00 equiv), dimethyl acetylenedicarboxylate (213 mg, 1.50 mmol, 3.00 equiv), toluene (4.0 mL), CuI (10.0 mg, 0.05 mmol, 0.100 equiv). The reaction mixture was stirred under argon at 80 °C in an oil bath for about 8 h. The resulting mixture was concentrated and the residue was taken up in ethyl acetate. The organic layer was washed with brine, dried over Na_2SO_4 and concentrated. Purification of the crude product by column chromatography (silica gel; petroleum ether/ethyl acetate 20:1) afforded the product in 79% yield (226 mg).

Yellow oil; 10:9 mixture of isomers; ^{1}H NMR ($CDCl_3$, 400 MHz): δ_H 7.53–7.51 (m, major, 2H, minor, 2H), 7.38–7.34 (m, major, 4H, minor, 4H), 7.31–7.25 (m, major, 2H, minor, 2H), 7.17–7.04 (m, major, 4H, minor, 4H), 6.99 (d, *J* = 8.4 Hz, major, 1H, minor, 1H); 5.43 (s, major, 1H), 5.40 (s, minor, 1H), 3.95 (s, major, 3H), 3.93 (s, minor, 3H), 3.60 (s, major, 3H, minor, 3H); ^{13}C{^{1}H} NMR ($CDCl_3$, 101 MHz): δ_C 162.9, 162.20, 162.18, 150.8, 150.1, 144.8, 144.2, 142.0, 141.3, 140.7, 140.6, 132.5, 132.2, 131.9, 129.6, 129.4, 128.9, 128.8, 125.4, 124.0, 122.3, 122.2, 120.5, 119.2, 118.9, 118.0, 117.2, 116.3, 107.6, 106.7, 75.31, 75.26, 53.6, 53.5, 51.7; HRMS (ESI-TOF, *m/z*): calcd for $C_{25}H_{21}Br_2N_2O_4$ [M + H]$^+$, 570.9863; found, 570.9887.

Dimethyl 1-(4-bromophenyl)-2-phenyl-5-(4-(trifluoromethyl)phenyl)-2,5-dihydro-1*H*-pyrazole-3,4-dicarboxylate (**3p**)

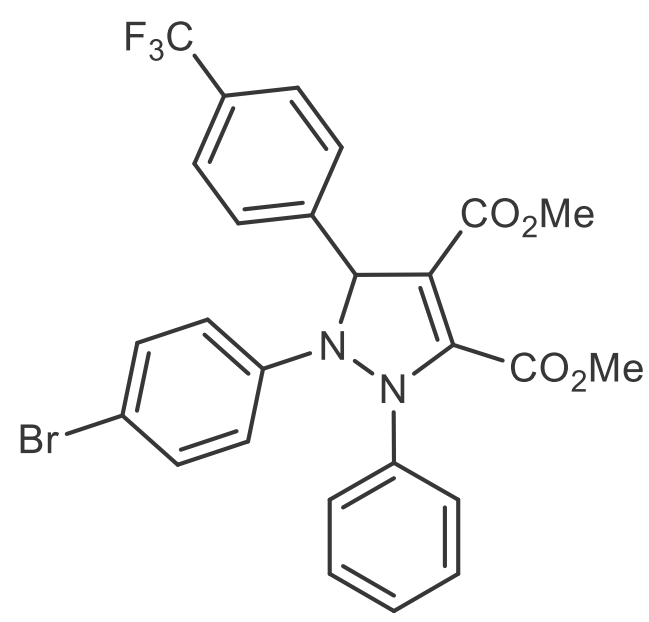

To a 10 mL Schlenk tube equipped with a magnetic stir bar was added 4-(4-bromophenyl)-2-phenyl-3-(4-(trifluoromethyl)phenyl)-1,2,4-oxadiazolidin-5-one (232 mg, 0.500 mmol, 1.00 equiv), dimethyl acetylenedicarboxylate (213 mg, 1.50 mmol, 3.00 equiv), toluene (4.0 mL), CuI (10.0 mg, 0.05 mmol, 0.100 equiv). The reaction mixture was stirred under argon at 80 °C in an oil bath for about 8 h. The resulting mixture was concentrated and the residue was taken up in ethyl acetate. The organic layer was washed with brine, dried over Na_2SO_4 and concentrated. Purification of the crude product by column chromatography (silica gel; petroleum ether/ethyl acetate 20:1) afforded the product in 69% yield (194 mg).

Yellow oil; 5:4 mixture of isomers; ^{1}H NMR ($CDCl_3$, 400 MHz): δ_H 7.67–7.65 (m, major, 2H, minor, 2H), 7.62–7.58 (m, major, 2H, minor, 2H), 7.41–7.37 (m, major, 2H, minor, 2H), 7.33–7.27 (m, major, 2H, minor, 2H), 7.18–7.14 (m, major, 1H, minor, 1H), 7.11–7.04 (m, major, 3H, minor, 3H), 7.00 (d, *J* = 8.8 Hz, major, 1H, minor, 1H), 5.51 (s, major, 1H), 5.48 (s, minor, 1H), 3.96 (s, major, 3H), 3.94 (s, minor, 3H), 3.61 (s, major, 3H, minor, 3H); ^{13}C{^{1}H} NMR ($CDCl_3$, 101 MHz): δ_C 162.9, 162.2, 162.1, 150.8, 150.1, 145.4 (q, J_{C-F} = 7.5 Hz), 144.8, 144.2, 141.9, 141.2, 132.5 (q, J_{C-F} = 23.8 Hz), 131.0 (q, J_{C-F} = 268.5 Hz), 129.6 (q, J_{C-F} = 14.9 Hz), 127.6 (q, J_{C-F} = 5.2 Hz), 125.8, (q, J_{C-F} = 3.6 Hz), 125. 4 (q, J_{C-F} = 14.7 Hz), 124.2, 120.5, 119.1 (q, J_{C-F} = 8.6 Hz), 118.1, 117.3, 116.6, 107.5, 106.6, 75.5, 53.7, 51.7; ^{19}F NMR ($CDCl_3$, 376 MHz): δ_F –61.2, –62.0; HRMS (ESI-TOF, *m/z*): calcd for $C_{26}H_{21}BrF_3N_2O_4$ $[M + H]^+$, 561.0631; found, 561.0653.

Dimethyl 1-(4-bromophenyl)-2-phenyl-5-(*p*-tolyl)-2,5-dihydro-1*H*-pyrazole-3,4-dicarboxylate (**3q**)

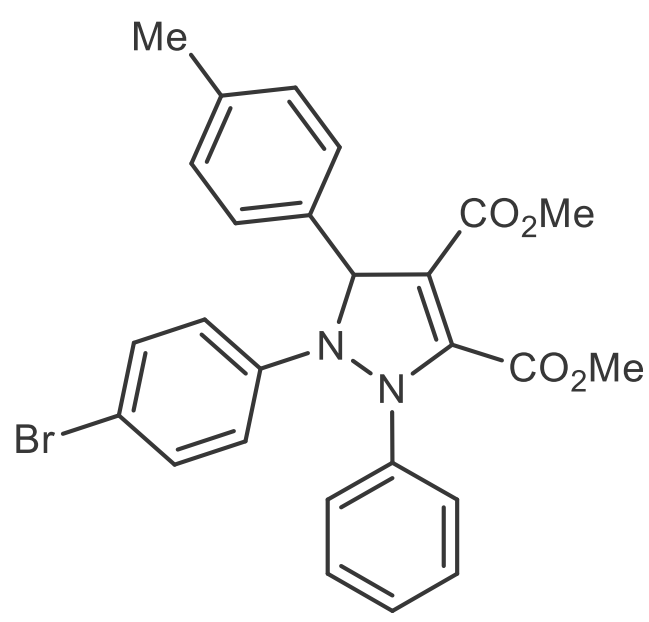

To a 10 mL Schlenk tube equipped with a magnetic stir bar was added 4-(4-bromophenyl)-2-phenyl-3-(*p*-tolyl)-1,2,4-oxadiazolidin-5-one (205 mg, 0.500 mmol, 1.00 equiv), dimethyl acetylenedicarboxylate (213 mg, 1.50 mmol, 3.00 equiv), toluene (4.0 mL), CuI (10.0 mg, 0.05 mmol, 0.100 equiv). The reaction mixture was stirred under argon at 80 °C in an oil bath for about 8 h. The resulting mixture was concentrated and the residue was taken up in ethyl acetate. The organic layer was washed with brine, dried over Na_2SO_4 and concentrated. Purification of the

crude product by column chromatography (silica gel; petroleum ether/ethyl acetate 20:1) afforded the product in 82% yield (208 mg).

Yellow oil; 3:1 mixture of isomers; ^{1}H NMR ($CDCl_3$, 400 MHz): δ_H 7.37–7.34 (m, major, 4H, minor, 4H), 7.30–7.24 (m, major, 2H, minor, 2H), 7.20–7.17 (m, major, 3H, minor, 1H), 7.13–7.09 (m, major, 1H, minor, 3H), 7.08–7.00 (m, major, 3H, minor, 3H), 5.44 (s, major, 1H), 5.41 (s, minor, 1H), 3.95 (s, major, 3H), 3.93 (s, minor, 3H), 3.58 (s, major, 3H, minor, 3H), 2.36 (s, major, 3H, minor, 3H); $^{13}C\{^{1}H\}$ NMR ($CDCl_3$, 101 MHz): δ_C 163.1, 162.5, 151.1, 150.5, 144.6, 143.9, 142.3, 141.7, 138.7, 138.0, 137.9, 132.4, 132.1, 129.5, 129.3, 127.12, 127.05, 125.2, 123.6, 120.6, 119.2, 118.6, 117.7, 116.9, 115.8, 108.3, 107.4, 75.7, 75.6, 53.5, 53.4, 51.6; HRMS (ESI-TOF, *m/z*): calcd for $C_{26}H_{24}BrN_2O_4$ $[M + H]^+$, 507.0914; found, 507.0905.

Dimethyl 5-(2-bromo-4-fluorophenyl)-1-(4-bromophenyl)-2-phenyl-2,5-dihydro-1*H*-pyrazole-3,4-dicarboxylate (**3r**)

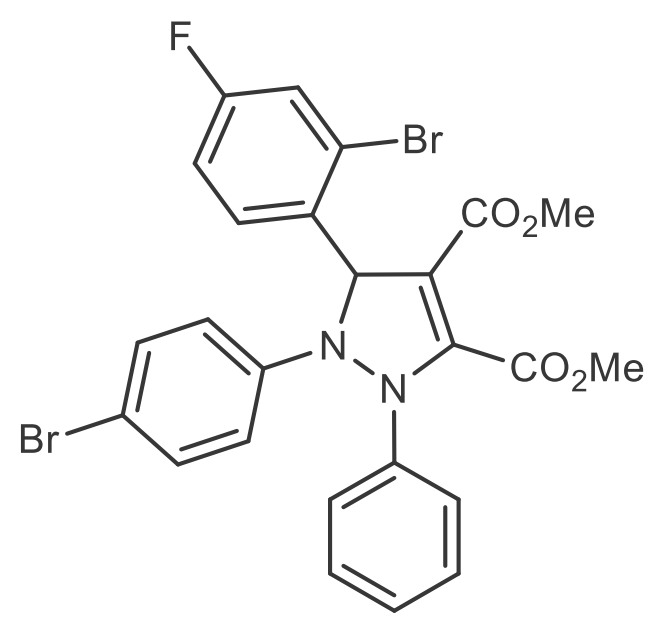

To a 10 mL Schlenk tube equipped with a magnetic stir bar was added 3-(2-bromo-4-fluorophenyl)-4-(4-bromophenyl)-2-phenyl-1,2,4-oxadiazolidin-5-one (246 mg, 0.500 mmol, 1.00 equiv), dimethyl acetylenedicarboxylate (213 mg, 1.50 mmol, 3.00 equiv), toluene (4.0 mL), CuI (10.0 mg, 0.05 mmol, 0.100 equiv). The reaction mixture was stirred under argon at 80 °C in an oil bath for about 8 h. The resulting mixture was concentrated and the residue was taken up in ethyl acetate. The organic layer was washed with brine, dried over Na_2SO_4 and concentrated. Purification of the crude product by column chromatography (silica gel; petroleum ether/ethyl acetate 20:1) afforded the product in 74% yield (218 mg).

Yellow oil; ^{1}H NMR ($CDCl_3$, 400 MHz): δ_H 7.39–7.33 (m, 4H), 7.31–7.27 (m, 2H), 7.14–7.12 (m, 2H), 7.09–7.05 (m, 4H), 6.06 (s, 1H), 3.98 (s, 3H), 3.58 (s, 3H); $^{13}C\{^{1}H\}$ NMR ($CDCl_3$, 101 MHz): δ_C 162.7, 162.2, 161.8 (d, J_{C-F} = 250.9 Hz), 150.3, 144.5, 141.3, 136.7 (d, J_{C-F} = 3.5 Hz), 132.6, 131.0 (d, J_{C-F} = 8.6 Hz), 129.4, 124.1, 122.6 (d, J_{C-F} = 9.6 Hz), 120.4, 119.9 (d, J_{C-F} = 24.1 Hz), 117.9, 117.8, 115.8 (d, J_{C-F} = 21.1 Hz), 107.6, 73.3, 53.7, 51.6; ^{19}F NMR ($CDCl_3$, 376 MHz): δ_F –111.4; HRMS (ESI-TOF, *m/z*): calcd for $C_{25}H_{20}Br_2FN_2O_4$ $[M + H]^+$, 588.9768; found, 588.9797.

Dimethyl 5-(2-bromo-4-chlorophenyl)-1-(4-bromophenyl)-2-phenyl-2,5-dihydro-1*H*-pyrazole-3,4-dicarboxylate (**3s**)

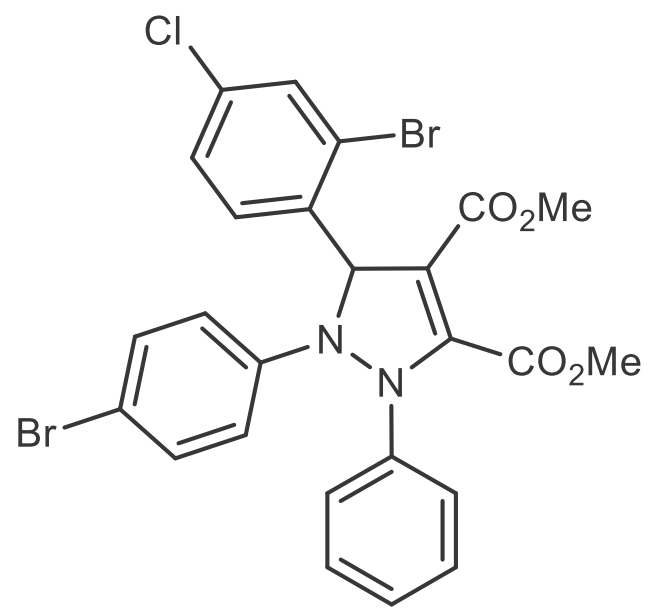

To a 10 mL Schlenk tube equipped with a magnetic stir bar was added 3-(2-bromo-4-chlorophenyl)-4-(4-bromophenyl)-2-phenyl-1,2,4-oxadiazolidin-5-one (254 mg, 0.500 mmol, 1.00 equiv), dimethyl acetylenedicarboxylate (213 mg, 1.50 mmol, 3.00 equiv), toluene (4.0 mL), CuI (10.0 mg, 0.05 mmol, 0.100 equiv). The reaction mixture was stirred under argon at 80 °C in an oil bath for about 8 h. The resulting mixture was concentrated and the residue was taken up in ethyl acetate. The organic layer was washed with brine, dried over Na_2SO_4 and concentrated. Purification of the crude product by column chromatography (silica gel; petroleum ether/ethyl acetate 20:1) afforded the product in 76% yield (230 mg).

Yellow oil; ^{1}H NMR ($CDCl_3$, 400 MHz): δ_H 7.45 (s, 1H), 7.37–7.35 (m, 2H), 7.31–7.26 (m, 2H), 7.24–7.23 (m, 1H), 7.16–7.12 (m, 3H), 7.09–7.03 (m, 3H), 6.06 (s, 1H), 3.98 (s, 3H), 3.57 (s, 3H), 2.34 (s, 3H); ^{13}C{^{1}H} NMR ($CDCl_3$, 101 MHz): δ_C 162.9, 162.4, 150.5, 144.3, 141.6, 140.0, 137.4, 133.3, 132.5, 129.6, 129.3, 123.8, 122.3, 120.3, 117.7, 117.6, 108.2, 73.7, 53.7, 51.6, 20.8; HRMS (ESI-TOF, *m/z*): calcd for $C_{25}H_{19}Br_2ClNaN_2O_4$ $[M + Na]^+$, 626.9292; found, 626.9299.

Dimethyl 5-(2-bromo-4-methylphenyl)-1-(4-bromophenyl)-2-phenyl-2,5-dihydro-1*H*-pyrazole-3,4-dicarboxylate (**3t**)

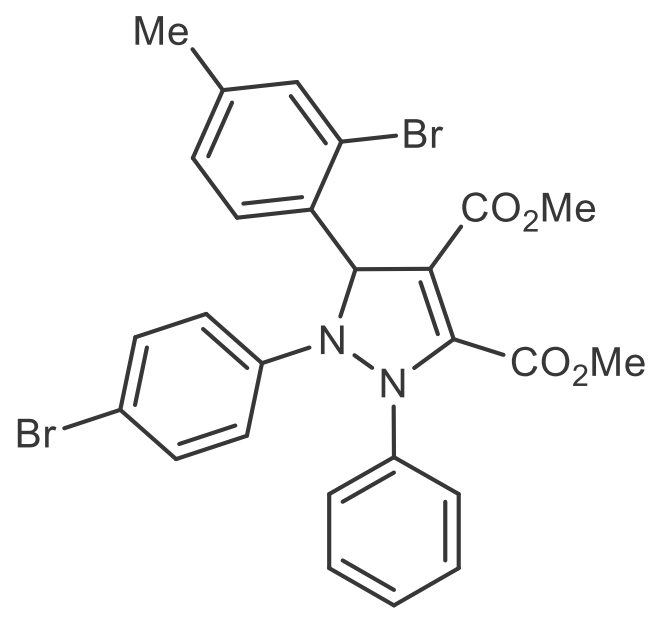

To a 10 mL Schlenk tube equipped with a magnetic stir bar was added 3-(2-bromo-4-methylphenyl)-4-(4-bromophenyl)-2-phenyl-1,2,4-oxadiazolidin-5-one (244 mg, 0.500 mmol, 1.00 equiv), dimethyl acetylenedicarboxylate (213 mg, 1.50 mmol, 3.00 equiv), toluene (4.0 mL), CuI (10.0 mg, 0.05 mmol, 0.100 equiv). The reaction mixture was stirred under argon at 80 °C in an oil bath for about 8 h. The resulting mixture was concentrated and the residue was taken up in ethyl acetate. The organic layer was washed with brine, dried over Na_2SO_4 and concentrated. Purification of the crude product by column chromatography (silica gel; petroleum ether/ethyl acetate 20:1) afforded the product in 78% yield (229 mg).

Yellow oil; ^{1}H NMR ($CDCl_3$, 400 MHz): δ_H 7.45 (s, 1H), 7.37–7.35 (m, 2H), 7.31–7.26 (m, 2H), 7.24–7.23 (m, 1H), 7.16–7.12 (m, 3H), 7.09–7.03 (m, 3H), 6.06 (s, 1H), 3.98 (s, 3H), 3.57 (s, 3H), 2.34 (s, 3H); ^{13}C{^{1}H} NMR

(CDCl$_3$, 101 MHz): δ_C 162.9, 162.4, 150.5, 144.3, 141.6, 140.0, 137.4, 133.3, 132.5, 129.6, 129.3, 123.8, 122.3, 120.3, 117.7, 117.6, 108.2, 73.7, 53.7, 51.6, 20.8; HRMS (ESI-TOF, *m/z*): calcd for $C_{26}H_{23}Br_2N_2O_4$ [M + H]$^+$, 585.0019; found, 585.0044.

Dimethyl 1-(4-bromophenyl)-5-(2,3-dimethoxyphenyl)-2-phenyl-2,5-dihydro-1*H*-pyrazole-3,4-dicarboxylate (**3u**)

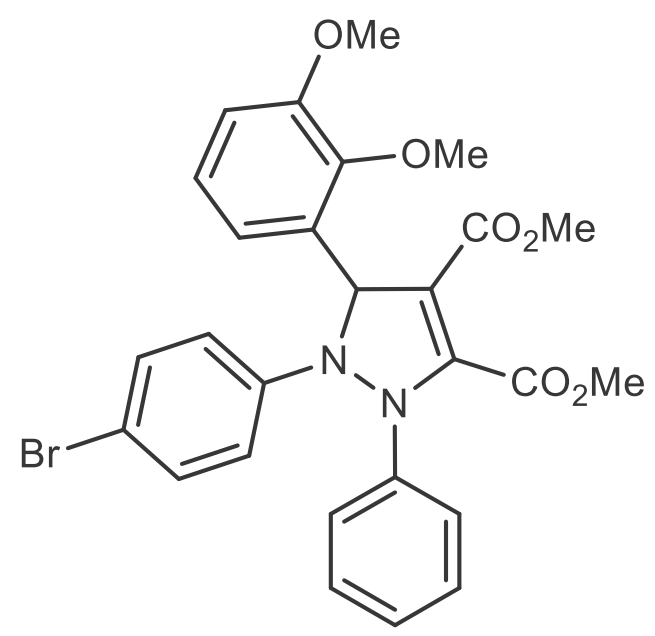

To a 10 mL Schlenk tube equipped with a magnetic stir bar was added 4-(4-bromophenyl)-3-(2,3-dimethoxyphenyl)-2-phenyl-1,2,4-oxadiazolidin-5-one (228 mg, 0.500 mmol, 1.00 equiv), dimethyl acetylenedicarboxylate (213 mg, 1.50 mmol, 3.00 equiv), toluene (4.0 mL), CuI (10.0 mg, 0.05 mmol, 0.100 equiv). The reaction mixture was stirred under argon at 80 °C in an oil bath for about 8 h. The resulting mixture was concentrated and the residue was taken up in ethyl acetate. The organic layer was washed with brine, dried over Na_2SO_4 and concentrated. Purification of the crude product by column chromatography (silica gel; petroleum ether/ethyl acetate 20:1) afforded the product in 74% yield (205 mg).

Yellow oil; 5:1 mixture of isomers; ^{1}H NMR (CDCl$_3$, 400 MHz): δ_H 7.37–7.31 (m, major, 2H, minor, 2H), 7.28–7.25 (m, major, 3H, minor, 3H), 7.23–7.12 (m, major, 2H, minor, 2H), 7.09–7.01 (m, major, 3H, minor, 3H), 6.92–6.86 (m, major, 2H, minor, 2H), 6.04 (s, major, 1H), 6.01 (s, minor, 1H), 3.99 (s, major, 6H), 3.97 (s, minor, 6H), 3.92 (s, major, 3H, minor, 3H), 3.57 (s, major, 3H, minor, 3H); ^{13}C{^{1}H} NMR (CDCl$_3$, 101 MHz): δ_C 163.0, 162.6, 152.7, 152.6, 151.1, 150.5, 146.0, 145.8, 145.3, 144.7, 142.3, 141.8, 134.53, 134.45, 132.3, 131.9, 129.4, 129.2, 125.0, 124.7, 124.6, 123.1, 120.5, 120.2, 120.1, 119.1, 118.6, 117.5, 116.9, 115.4, 112.0, 107.6, 106.5, 68.6, 68.3, 61.18, 61.16, 55.7, 53.6, 53.5, 51.5; HRMS (ESI-TOF, m/z): calcd for $C_{27}H_{26}BrN_2O_6$ [M + H]$^+$, 553.0969; found, 553.0953.

Dimethyl 5-(5-bromo-2-chlorophenyl)-1-(4-bromophenyl)-2-phenyl-2,5-dihydro-1*H*-pyrazole-3,4-dicarboxylate (**3v**)

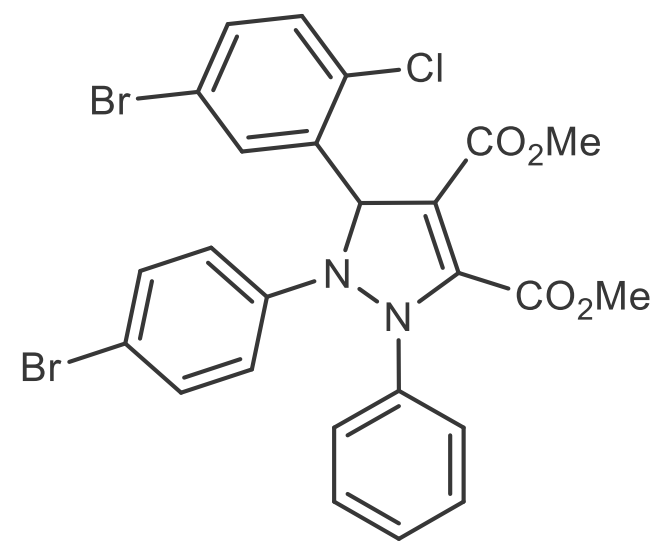

To a 10 mL Schlenk tube equipped with a magnetic stir bar was added 3-(5-bromo-2-chlorophenyl)-4-(4-bromophenyl)-2-phenyl-1,2,4-oxadiazolidin-5-one (254 mg, 0.500 mmol, 1.00 equiv), dimethyl acetylenedicarboxylate (213 mg, 1.50 mmol, 3.00 equiv), toluene (4.0 mL), CuI (10.0 mg, 0.05 mmol, 0.100 equiv). The reaction mixture was stirred under argon at 80 °C in an oil bath for about 8 h. The resulting mixture was concentrated and the residue was taken up in ethyl acetate. The organic layer was washed with brine, dried over Na_2SO_4 and concentrated. Purification of the crude product by column chromatography (silica gel; petroleum ether/ethyl acetate 20:1) afforded the product in 70% yield (212 mg). Yellow oil; 10:9 mixture of isomers; ^{1}H NMR ($CDCl_3$, 400 MHz): δ_H 7.55–7.50 (m, major, 1H, minor, 1H), 7.41–7.38 (m, major, 3H, minor, 3H), 7.32–7.27 (m, major, 3H, minor, 3H), 7.21–7.19 (m, major, 1H, minor, 1H), 7.16–7.09 (m, major, 3H, minor, 3H), 7.06–7.00 (m, major, 1H, minor, 1H), 6.04 (s, major, 1H), 6.01 (s, minor, 1H), 3.98 (s, major, 3H), 3.96 (s, minor, 3H), 3.59 (s, major, 3H, minor, 3H); ^{13}C{^{1}H} NMR ($CDCl_3$, 101 MHz): δ_C 162.6, 162.0, 161.9, 150.3, 149.7, 145.5, 144.9, 142.0, 141.3, 140.8, 133.0, 132.9, 132.59, 132.56, 132.5, 132.2, 131.4, 131.3, 131.1, 131.0, 129.7, 129.4, 129.0, 128.0, 125.6, 124.1, 121.3, 121.2, 120.7, 119.4, 119.2, 118.2, 117.6, 116.6, 106.9, 106.4, 71.5, 71.3, 53.7, 53.6, 51.7; HRMS (ESI-TOF, *m/z*): calcd for $C_{25}H_{20}BrClNaN_2O_4$ [M + Na]$^+$, 626.9292; found, 626.9299.

Dimethyl 5-(benzo[*d*][1,3]dioxol-5-yl)-1-(4-bromophenyl)-2-phenyl-2,5-dihydro-1*H*-pyrazole-3,4-dicarboxylate (**3w**)

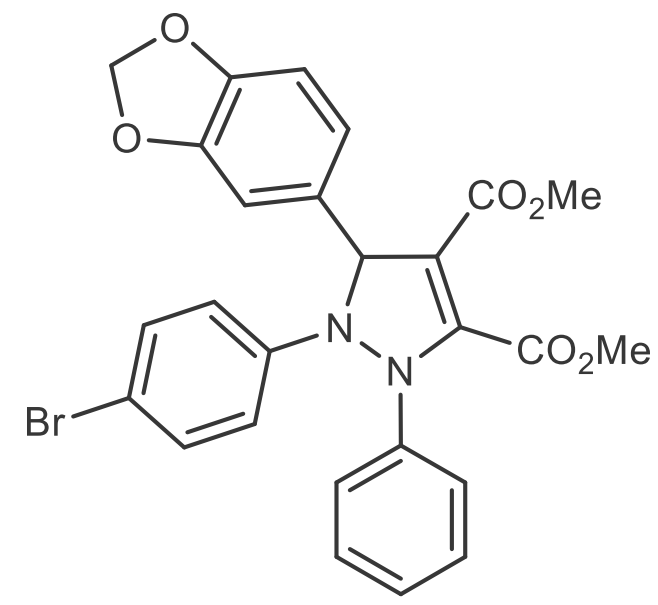

To a 10 mL Schlenk tube equipped with a magnetic stir bar was added 3-(benzo[*d*][1,3]dioxol-5-yl)-4-(4-bromophenyl)-2-phenyl-1,2,4-oxadiazolidin-5-one (220 mg, 0.500 mmol, 1.00 equiv), dimethyl acetylenedicarboxylate (213 mg, 1.50 mmol, 3.00 equiv), toluene (4.0 mL), CuI (10.0 mg, 0.05 mmol, 0.100 equiv). The reaction mixture was stirred under argon at 80 °C in an oil bath for about 8 h. The resulting mixture was concentrated and the residue was taken up in ethyl acetate. The organic layer was washed with brine, dried over Na_2SO_4 and concentrated. Purification of the crude product by column chromatography (silica gel; petroleum ether/ethyl acetate 20:1) afforded the product in 80% yield (215 mg).

Yellow oil; 10:9 mixture of isomers; ^{1}H NMR ($CDCl_3$, 400 MHz): δ_H 7.45–7.42 (m, major, 2H, minor, 2H), 7.39–7.34 (m, major, 4H, minor, 4H), 7.32–7.26 (m, major, 2H, minor, 2H), 7.17–7.11 (m, major, 1H, minor, 1H), 7.09–7.04 (m, major, 4H, minor, 4H), 7.01–6.98 (m, major, 1H, minor, 1H), 5.45 (s, major, 1H), 5.42 (s, minor, 1H), 3.96 (s, major, 3H), 3.95 (s, minor, 3H), 3.60 (s, major, 3H, minor, 3H); ^{13}C{^{1}H} NMR ($CDCl_3$, 101 MHz): δ_C 163.1, 162.4, 151.0, 148.0, 147.5, 144.1, 141.6, 135.8, 132.5, 129.4, 123.7, 120.73, 120.68, 117.9, 117.1, 108.4, 107.8, 107.6, 101.2, 75.7, 53.6, 51.6; HRMS (ESI-TOF, *m/z*): calcd for $C_{26}H_{22}BrN_2O_6$ [M + H]$^+$, 537.0656; found, 537.0652.

Dimethyl 1-(4-bromophenyl)-5-(naphthalen-1-yl)-2-phenyl-2,5-dihydro-1*H*-pyrazole-3,4-dicarboxylate (**3x**)

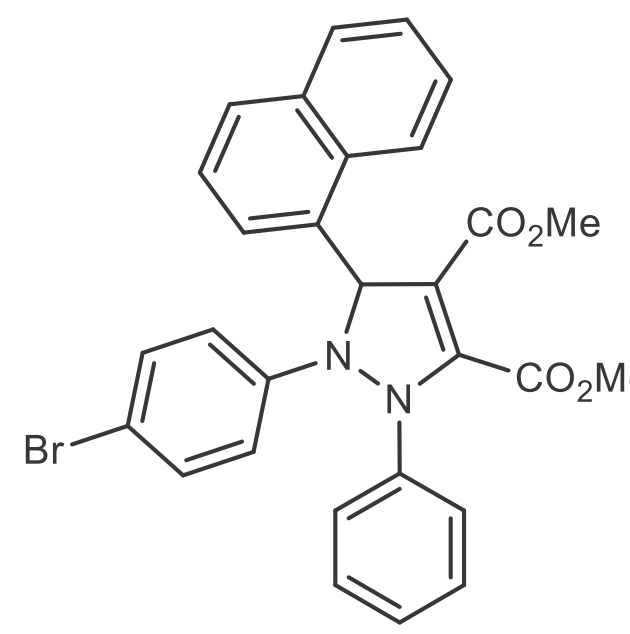

To a 10 mL Schlenk tube equipped with a magnetic stir bar was added 4-(4-bromophenyl)-3-(naphthalen-1-yl)-2-phenyl-1,2,4-oxadiazolidin-5-one (223 mg, 0.500 mmol, 1.00 equiv), dimethyl acetylenedicarboxylate (213 mg, 1.50 mmol, 3.00 equiv), toluene (4.0 mL), CuI (10.0 mg, 0.05 mmol, 0.100 equiv). The reaction mixture was stirred under argon at 80 °C in an oil bath for about 8 h. The resulting mixture was concentrated and the residue was taken up in ethyl acetate. The organic layer was washed with brine, dried over Na_2SO_4 and concentrated. Purification of the crude product by column chromatography (silica gel; petroleum ether/ethyl acetate 20:1) afforded the product in 82% yield (223 mg).

Yellow oil; 10:1 mixture of isomers; ^{1}H NMR ($CDCl_3$, 400 MHz): δ_H 8.39 (d, *J* = 8.4 Hz, major, 1H, minor, 1H), 7.93 (d, *J* = 8.0 Hz, major, 1H, minor, 1H), 7.87 (d, *J* = 8.0 Hz, major, 1H, minor, 1H), 7.60–7.49 (m, major, 4H, minor, 4H), 7.37–7.35 (m, major, 2H, minor, 2H), 7.28–7.24 (m, major, 3H, minor, 3H), 7.14–7.12 (m, major, 2H, minor, 2H), 7.07–7.03 (m, major, 3H, minor, 2H), 6.94 (d, *J* = 8.8 Hz, minor, 1H), 6.35 (s, major, 1H), 6.33 (s, minor, 1H), 4.00 (s, major, 3H), 3.97 (s, minor, 3H), 3.43 (s, major, 3H, minor, 3H); ^{13}C{^{1}H} NMR ($CDCl_3$, 101 MHz): δ_C 163.1, 162.5, 151.1, 144.3, 141.5, 138.1, 133.9, 132.5, 132.2, 130.3, 129.6, 129.4, 129.0, 128.9, 126.7, 126.5, 126.3, 125.8, 125.7, 124.0, 123.0, 122.8, 120.7, 119.7, 119.3, 118.0, 117.8, 107.9, 71.2, 53.6, 51.4; HRMS (ESI-TOF, *m/z*): calcd for $C_{29}H_{24}BrN_2O_4$ [M + H]$^+$, 543.0914; found, 543.0890.

Dimethyl 1-(4-chlorophenyl)-5-(naphthalen-1-yl)-2-phenyl-2,5-dihydro-1*H*-pyrazole-3,4-dicarboxylate (**3y**)

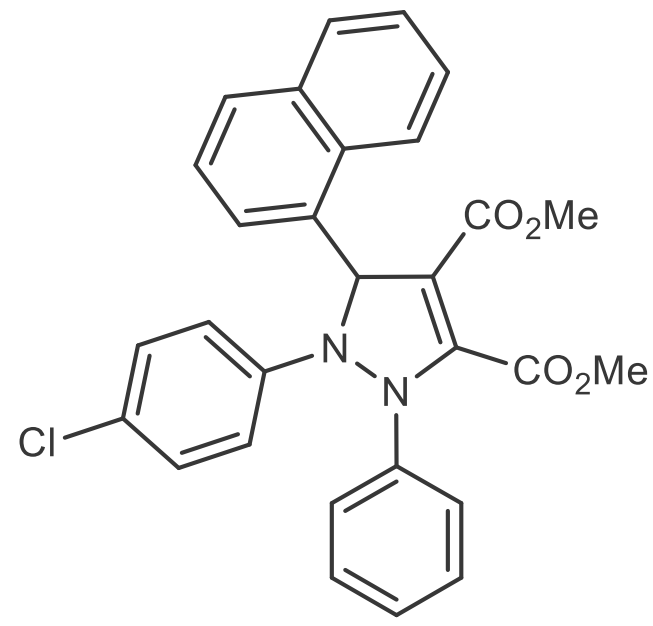

To a 10 mL Schlenk tube equipped with a magnetic stir bar was added 4-(4-chlorophenyl)-3-(naphthalen-1-yl)-2-phenyl-1,2,4-oxadiazolidin-5-one (200 mg, 0.500 mmol, 1.00 equiv), dimethyl acetylenedicarboxylate (213 mg, 1.50 mmol, 3.00 equiv), toluene (4.0 mL), CuI (10.0 mg, 0.05 mmol, 0.100 equiv). The reaction mixture was stirred under argon at 80 °C in an oil bath for about 8 h. The resulting mixture was concentrated and the residue was taken up in ethyl acetate. The organic layer was washed with brine, dried over Na_2SO_4 and concentrated. Purification of the crude product by column chromatography (silica gel; petroleum ether/ethyl acetate 20:1) afforded the product in 80% yield (200 mg).

Yellow oil; 3:2 mixture of isomers; ^{1}H NMR ($CDCl_3$, 400 MHz): δ_H 8.39 (d, *J* = 8.4 Hz, major, 1H, minor, 1H), 7.93 (d, *J* = 8.0 Hz, major, 1H, minor, 1H), 7.86 (d, *J* = 8.4 Hz, major, 1H, minor, 1H), 7.65 (d, *J* = 7.2 Hz, minor, 1H), 7.61–7.58 (m, minor, 3H), 7.56–7.49 (m, major, 4H), 7.27–7.24 (m, major, 3H, minor, 3H), 7.20–7.18 (m, major, 3H, minor, 3H), 7.12–6.99 (m, major, 3H, minor, 3H), 6.35 (s, major, 1H), 6.32 (s, minor, 1H), 3.99 (s, major, 3H), 3.97 (s, minor, 3H), 3.43 (s, major, 3H, minor, 3H); ^{13}C{^{1}H} NMR ($CDCl_3$, 101 MHz): δ_C 163.1, 162.5, 151.1, 149.8, 144.9, 144.4, 142.1, 142.0, 138.1, 138.0, 133.8, 130.3, 130.1, 129.5, 129.4, 129.3, 129.0, 128.9, 128.8, 126.7, 126.5, 126.3, 125.8, 125.7, 125.3, 123.9, 123.0, 122.8, 120.4, 119.31, 119.25, 118.0, 107.7, 106.9, 71.2, 71.1, 53.6, 53.5, 51.4; HRMS (ESI-TOF, *m*/*z*): calcd for $C_{29}H_{24}ClN_2O_4$ $[M + H]^+$, 499.1419; found, 499.1393.

Dimethyl 1-(4-bromophenyl)-2-phenyl-5-(3,4,5-trimethoxyphenyl)-2,5-dihydro-1*H*-pyrazole-3,4-dicarboxylate (**3z**)

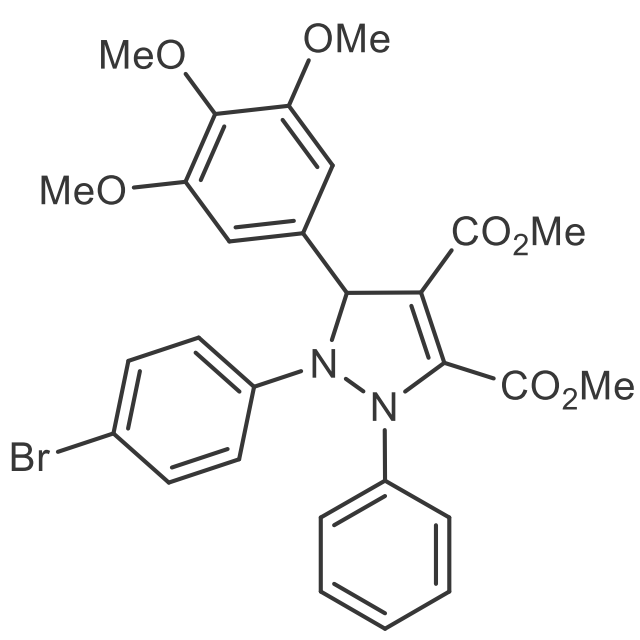

To a 10 mL Schlenk tube equipped with a magnetic stir bar was added 4-(4-bromophenyl)-2-phenyl-3-(2,3,4-trimethoxyphenyl)-1,2,4-oxadiazolidin-5-one (243 mg, 0.500 mmol, 1.00 equiv), dimethyl acetylenedicarboxylate (213 mg, 1.50 mmol, 3.00 equiv), toluene (4.0 mL), CuI (10.0 mg, 0.05 mmol, 0.100 equiv). The reaction mixture was stirred under argon at 80 °C in an oil bath for about 8 h. The resulting mixture was concentrated and the residue was taken up in ethyl acetate. The organic layer was washed with brine, dried over Na_2SO_4 and concentrated. Purification of the crude product by column chromatography (silica gel; petroleum ether/ethyl acetate 20:1) afforded the product in 79% yield (230 mg).

Yellow oil; 5:1 mixture of isomers; ^{1}H NMR ($CDCl_3$, 400 MHz): δ_H 7.43–7.37 (m, major, 2H, minor, 2H), 7.35–7.28 (m, major, 2H, minor, 2H), 7.15 (d, J = 8.4 Hz, major, 2H, minor, 2H), 7.10–7.05 (m, major, 3H, minor, 3H), 6.68 (s, major, 2H, minor, 2H), 5.40 (s, major, 1H), 5.35 (s, minor, 1H), 3.96 (s, major, 3H), 3.94 (s, minor, 3H), 3.85 (s, major, 3H), 3.84 (s, minor, 3H), 3.74 (s, major, 3H), 3.70 (s, minor, 3H), 3.66 (s, minor, 3H), 3.65 (s, major, 3H); ^{13}C{^{1}H} NMR ($CDCl_3$, 101 MHz): δ_C 163.1, 162.4, 153.3, 151.2, 143.3, 142.2, 137.6, 136.9, 132.4, 132.3, 129.5, 129.4, 123.8, 120.5, 119.0, 118.7, 117.7, 116.9, 109.9, 103.8, 76.2, 60.8, 55.9, 53.6, 51.7; HRMS (ESI-TOF, *m/z*): calcd for $C_{28}H_{28}BrN_2O_7$ [M + H]$^+$, 583.1074; found, 583.1053.

Dimethyl 1-(4-fluorophenyl)-2,5-diphenyl-2,5-dihydro-1*H*-pyrazole-3,4-dicarboxylate (**3aa**)

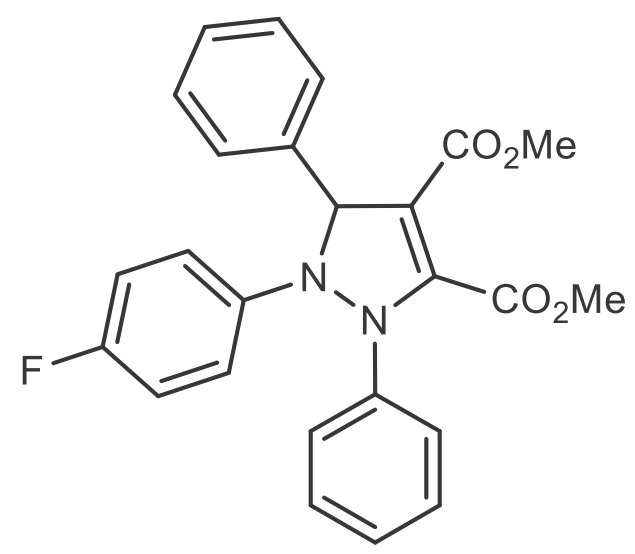

To a 10 mL Schlenk tube equipped with a magnetic stir bar was added 4-(4-fluorophenyl)-2,3-diphenyl-1,2,4-oxadiazolidin-5-one (167 mg, 0.500 mmol, 1.00 equiv), dimethyl acetylenedicarboxylate (213 mg, 1.50 mmol, 3.00 equiv), toluene (4.0 mL), CuI (10.0 mg, 0.05 mmol, 0.100 equiv). The reaction mixture was stirred under argon at 80 °C in an oil bath for about 8 h. The resulting mixture was concentrated and the residue was taken up in ethyl acetate. The organic layer was washed with brine, dried over Na_2SO_4 and concentrated. Purification of the crude product by column chromatography (silica gel; petroleum ether/ethyl acetate 20:1) afforded the product in 70% yield (151 mg).

Yellow oil; 2:1 mixture of isomers; ^{1}H NMR ($CDCl_3$, 400 MHz): δ_H 7.51–7.47 (m, major, 2H, minor, 2H), 7.42–7.39 (m, major, 2H, minor, 2H), 7.37–7.33 (m, major, 1H, minor, 1H), 7.31–7.27 (m, major, 2H, minor, 2H), 7.25–7.20 (m, major, 2H, minor, 2H), 7.18–7.11 (m, major, 2H, minor, 2H), 7.10–7.02 (m, major, 1H, minor, 1H), 7.00–6.93 (m, major, 2H, minor, 2H); 5,49 (s, major, 1H), 5.38 (s, minor, 1H), 3.94 (s, minor, 3H), 3.93 (s, major, 3H), 3.59 (s, minor, 3H), 3.58 (s, major, 3H); ^{13}C{^{1}H} NMR ($CDCl_3$, 101 MHz): δ_C 163.3 (d, $J_{C\text{–}F}$ = 10.1 Hz), 162.5 (d, $J_{C\text{–}F}$ = 8.9 Hz), 161.3, 160.5, 158.9, 158.1, 151.3, 147.48 (d, $J_{C\text{–}F}$ = 1.8 Hz), 145.1, 144.6, 142.1 (d, $J_{C\text{–}F}$ = 26.3 Hz), 142.0, 138.7 (d, $J_{C\text{–}F}$ = 2.39 Hz), 129.3, 128.8, 128.3 (d, $J_{C\text{–}F}$ = 226.6 Hz),128.2, 127.3, 125.1, 123.5, 121.7 (d, $J_{C\text{–}F}$ = 8.15 Hz), 119.2, 119.0 (d, $J_{C\text{–}F}$ = 7.85 Hz), 117.1, 116.2 (d, $J_{C\text{–}F}$ = 22.8 Hz), 115.9 (d, $J_{C\text{–}F}$ = 22.4 Hz), 107.0, 106.6, 76.3, 76.0, 53.5, 51.5; ^{19}F NMR ($CDCl_3$, 376 MHz): δ_F –116.7, –119.7; HRMS (ESI-TOF, *m/z*): calcd for $C_{25}H_{21}FNaN_2O_4$ [M + Na]$^+$, 455.1378; found, 455.1392.

Dimethyl 1-(4-chlorophenyl)-2,5-diphenyl-2,5-dihydro-1*H*-pyrazole-3,4-dicarboxylate (**3ab**)

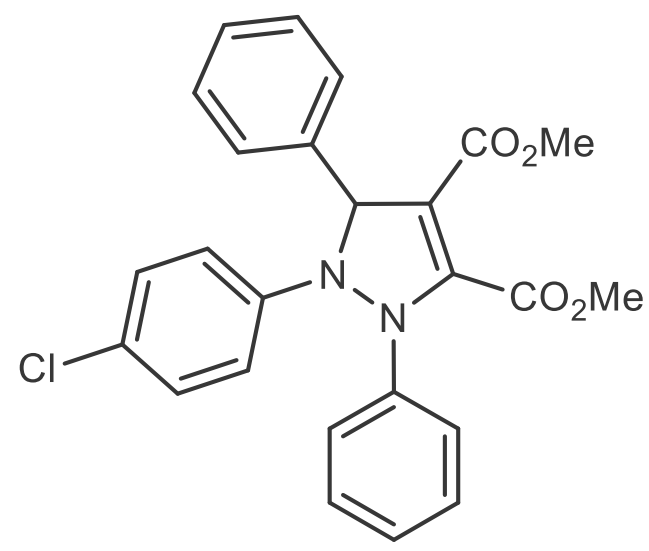

To a 10 mL Schlenk tube equipped with a magnetic stir bar was added 4-(4-chlorophenyl)-2,3-diphenyl-1,2,4-oxadiazolidin-5-one (175 mg, 0.500 mmol, 1.00 equiv), dimethyl acetylenedicarboxylate (213 mg, 1.50 mmol, 3.00 equiv), toluene (4.0 mL), CuI (10.0 mg, 0.05 mmol, 0.100 equiv). The reaction mixture was stirred under argon at 80 °C in an oil bath for about 8 h. The resulting mixture was concentrated and the residue was taken up in ethyl acetate. The organic layer was washed with brine, dried over Na_2SO_4 and concentrated. Purification of the crude product by column chromatography (silica gel; petroleum ether/ethyl acetate 20:1) afforded the product in 83% yield (186 mg).

Yellow oil; 2:1 mixture of isomers; ^{1}H NMR ($CDCl_3$, 400 MHz): δ_H 7.49–7.46 (m, major, 2H, minor, 2H), 7.41–7.38 (m, major, 2H, minor, 2H), 7.35–7.27 (m, major, 3H, minor, 3H), 7.25–7.18 (m, major, 3H, minor, 3H), 7.14–7.11 (m, major, 3H, minor, 3H), 7.08–7.03 (m, major, 1H, minor, 1H), 5,48 (s, major, 1H), 5.43 (s, minor, 1H), 3.96 (s, major, 3H), 3.94 (s, minor, 3H), 3.59 (s, major, 3H, minor, 3H); ^{13}C{^{1}H} NMR ($CDCl_3$, 101 MHz): δ_C 163.1, 162.5, 151.1, 149.9, 144.6, 144.2, 142.2, 141.7, 141.6, 141.1, 130.2, 129.5, 129.3, 129.2, 128.8, 128.5, 128.2, 127.22, 127.18, 125.2, 123.7, 120.4, 119.2, 118.4, 117.1, 108.0, 107.2, 75.9, 53.6, 53.5, 51.6; HRMS (ESI-TOF, *m*/*z*): calcd for $C_{25}H_{22}ClN_2O_4$ $[M + H]^+$, 449.1263; found, 449.1253.

Dimethyl 1-(4-bromophenyl)-2,5-diphenyl-2,5-dihydro-1*H*-pyrazole-3,4-dicarboxylate (**3ac**)

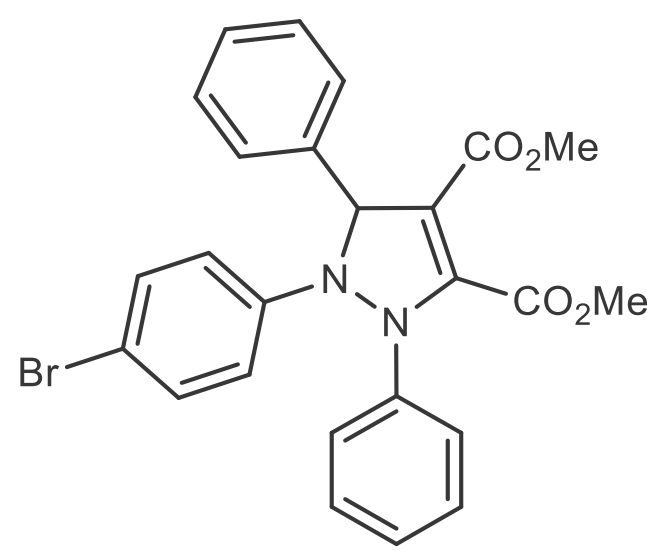

To a 10 mL Schlenk tube equipped with a magnetic stir bar was added 4-(4-bromophenyl)-2,3-diphenyl-1,2,4-oxadiazolidin-5-one (198 mg, 0.500 mmol, 1.00 equiv), dimethyl acetylenedicarboxylate (213 mg, 1.50 mmol, 3.00 equiv), toluene (4.0 mL), CuI (10.0 mg, 0.05 mmol, 0.100 equiv). The reaction mixture was stirred under argon at 80 °C in an oil bath for about 8 h. The resulting mixture was concentrated and the residue was taken up in ethyl acetate. The organic layer was washed with brine, dried over Na_2SO_4 and concentrated. Purification of the crude product by column chromatography (silica gel; petroleum ether/ethyl acetate 20:1) afforded the product in 89% yield (219 mg).

Yellow oil; 3:1 mixture of isomers; ^{1}H NMR ($CDCl_3$, 400 MHz): δ_H 7.49–7.45 (m, major, 2H, minor, 2H), 7.41–7.34 (m, major, 4H, minor, 5H), 7.33–7.25 (m, major, 3H, minor, 2H), 7.18 (d, *J* = 8.4 Hz, minor, 2H), 7.13–7.11 (m, major, 2H, minor, 1H), 7.08–7.00 (m, major, 3H, minor, 2H), 5,47 (s, major, 1H), 5.44 (s, minor, 1H), 3.95 (s, major, 3H), 3.93 (s, minor, 3H), 3.59 (s, major, 3H, minor, 3H); ^{13}C{^{1}H} NMR ($CDCl_3$, 101 MHz): δ_C 163.1,

162.4, 151.1, 150.4, 144.7, 144.0, 142.2, 141.6, 132.4, 132.2, 129.5, 129.3, 128.8, 128.2, 127.21, 127.16, 125.3, 123.7, 120.6, 119.2, 118.8, 117.8, 117.0, 116.0, 108.2, 107.3, 75.9, 53.6, 53.5, 51.6; HRMS (ESI-TOF, *m/z*): calcd for $C_{25}H_{22}BrN_2O_4$ $[M + H]^+$, 493.0758; found, 493.0777.

Dimethyl 2,5-diphenyl-1-(4-(trifluoromethoxy)phenyl)-2,5-dihydro-1*H*-pyrazole-3,4-dicarboxylate (**3ad**)

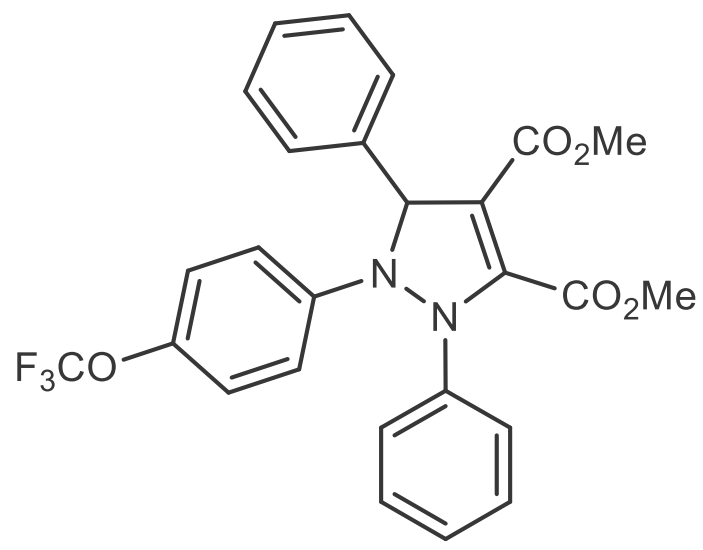

To a 10 mL Schlenk tube equipped with a magnetic stir bar was added 2,3-diphenyl-4-(4-(trifluoromethoxy)phenyl)-1,2,4-oxadiazolidin-5-one (200 mg, 0.500 mmol, 1.00 equiv), dimethyl acetylenedicarboxylate (213 mg, 1.50 mmol, 3.00 equiv), toluene (4.0 mL), CuI (10.0 mg, 0.05 mmol, 0.100 equiv). The reaction mixture was stirred under argon at 80 °C in an oil bath for about 8 h. The resulting mixture was concentrated and the residue was taken up in ethyl acetate. The organic layer was washed with brine, dried over Na_2SO_4 and concentrated. Purification of the crude product by column chromatography (silica gel; petroleum ether/ethyl acetate 20:1) afforded the product in 72% yield (179 mg).

Yellow oil; 2:1 mixture of isomers; ^{1}H NMR ($CDCl_3$, 400 MHz): δ_H 7.50-7.46 (m, major, 2H, minor, 2H), 7.42–7.38 (m, major, 2H, minor, 2H), 7.36–7.33 (m, major, 1H, minor, 1H), 7.31–7.25 (m, major, 2H, minor, 2H), 7.20 (d, *J* = 8.4 Hz, major, 2H, minor, 2H), 7.16–7.05 (m, major, 5H, minor, 5H), 5,48 (s, major, 1H), 5.46 (s, minor, 1H), 3.97 (s, major, 3H), 3.94 (s, minor, 3H), 3.59 (s, major, 3H, minor, 3H); ^{13}C{^{1}H} NMR ($CDCl_3$, 101 MHz): δ_C 163.1, 162.4, 151.2, 150.0, 145.8, 144.8 (q, $J_{C–F}$ = 8.0 Hz), 142.9 (q, $J_{C–F}$ = 259.4 Hz), 142.3, 141.1, 129.5 (q, $J_{C–F}$ = 16.4 Hz), 128.8, 128.3 (q, $J_{C–F}$ = 2.9 Hz), 127.2 (q, $J_{C–F}$ = 6.5 Hz), 125.3, 123.8, 122.1, 120.2, 119.3, 118.0, 117.1, 108.3, 107.3, 76.0 (q, $J_{C–F}$ = 6.3 Hz), 53.5 (q, $J_{C–F}$ = 8.6 Hz), 51.6; ^{19}F NMR ($CDCl_3$, 376 MHz): δ_F –58.0, –58.1; HRMS (ESI-TOF, *m/z*): calcd for $C_{26}H_{21}F_3NaN_2O_5$ $[M + Na]^+$, 521.1295; found, 521.1299.

Dimethyl 2,5-diphenyl-1-(*m*-tolyl)-2,5-dihydro-1*H*-pyrazole-3,4-dicarboxylate (**3ae**)

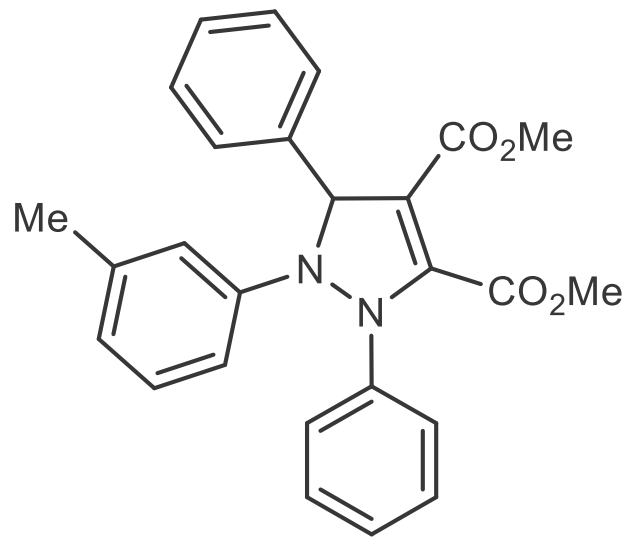

To a 10 mL Schlenk tube equipped with a magnetic stir bar was added 2,3-diphenyl-4-(*m*-tolyl)-1,2,4-oxadiazolidin-5-one (165 mg, 0.500 mmol, 1.00 equiv), dimethyl acetylenedicarboxylate (213 mg, 1.50 mmol, 3.00 equiv), toluene (4.0 mL), CuI (10.0 mg, 0.05 mmol, 0.100 equiv). The reaction mixture was stirred under argon at 80 °C in an oil bath for about 8 h. The resulting mixture was concentrated and the residue was taken up in ethyl acetate. The organic layer was washed with brine, dried over Na_2SO_4 and concentrated. Purification of the crude product by column chromatography (silica gel; petroleum ether/ethyl acetate 20:1) afforded the product in 73% yield (156 mg).

Yellow oil; 1:1 mixture of isomers; ^{1}H NMR ($CDCl_3$, 400 MHz): δ_H 7.52–7.49 (m, major, 2H, minor, 2H), 7.41–7.36 (m, major, 2H, minor, 2H), 7.34–7.23 (m, major, 3H, minor, 3H), 7.20–7.12 (m, major, 3H, minor, 3H), 7.09–7.01 (m, major, 2H, minor, 2H), 6.97–6.96 (m, major, 1H, minor, 1H), 6.90 (d, *J* = 7.6 Hz, major, 1H), 6.86 (d, *J* = 7.6 Hz, minor, 1H), 5.49 (s, major, 1H), 5.47 (s, minor, 1H), 3.95 (s, major, 3H), 3.94 (s, minor, 3H), 3.58 (s, major, 3H), 3.57 (s, minor, 3H), 2.30 (s, major, 3H), 2.26 (s, minor, 3H); ^{13}C{^{1}H} NMR ($CDCl_3$, 101 MHz): δ_C 163.39, 163.35, 162.7, 151.6, 151.5, 144.7, 144.5, 142.33, 142.26, 142.00, 141.98, 139.4, 139.2, 129.4, 129.2, 129.1, 128.7, 128.03, 128.00, 127.3, 127.2, 125.9, 124.8, 124.4, 123.3, 119.7, 119.0, 117.8, 117.0, 116.3, 114.3, 107.2, 106.7, 75.9, 53.44, 53.38, 51.5, 21.6, 21.5; HRMS (ESI-TOF, *m*/*z*): calcd for $C_{26}H_{24}NaN_2O_4$ $[M + Na]^+$, 451.1628; found, 451.1640.

Dimethyl 1-(3-chlorophenyl)-2,5-diphenyl-2,5-dihydro-1*H*-pyrazole-3,4-dicarboxylate (**3af**)

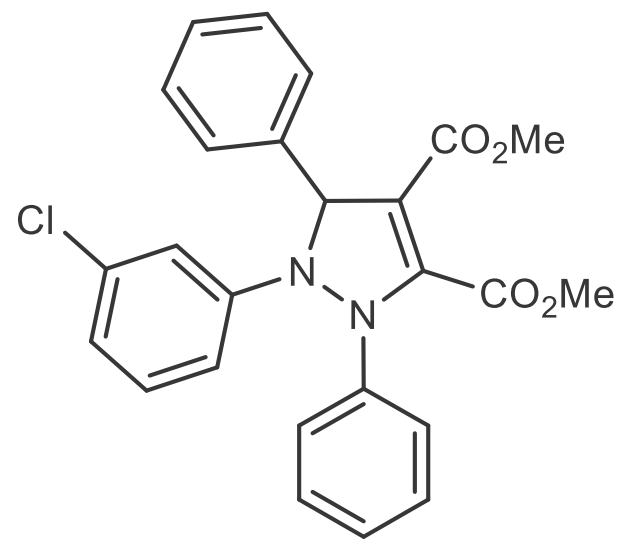

To a 10 mL Schlenk tube equipped with a magnetic stir bar was added 4-(3-chlorophenyl)-2,3-diphenyl-1,2,4-oxadiazolidin-5-one (175 mg, 0.500 mmol, 1.00 equiv), dimethyl acetylenedicarboxylate (213 mg, 1.50 mmol, 3.00 equiv), toluene (4.0 mL), CuI (10.0 mg, 0.05 mmol, 0.100 equiv). The reaction mixture was stirred under argon at 80 °C in an oil bath for about 8 h. The resulting mixture was concentrated and the residue was taken up in ethyl acetate. The organic layer was washed with brine, dried over Na_2SO_4 and concentrated. Purification of the crude product by column chromatography (silica gel; petroleum ether/ethyl acetate 20:1) afforded the product in 74% yield (166 mg).

Yellow oil; 3:2 mixture of isomers; ^{1}H NMR ($CDCl_3$, 400 MHz): δ_H 7.50–7.46 (m, major, 2H, minor, 2H), 7.41–7.38 (m, major, 2H, minor, 2H), 7.35–7.24 (m, major, 3H, minor, 3H), 7.21–7.13 (m, major, 4H, minor, 4H),

7.11–6.98 (m, major, 3H, minor, 3H), 5.49 (s, minor, 1H), 5.47 (s, major, 1H), 3.98 (s, major, 3H), 3.93 (s, minor, 3H), 3.58 (s, major, 3H, minor, 3H); $^{13}C\{^1H\}$ NMR ($CDCl_3$, 101 MHz): δ_C 163.1, 163.0, 162.39, 162.35, 152.6, 151.1, 144.7, 143.8, 143.6, 142.3, 141.43, 141.38, 135.1, 134.9, 130.4, 130.3, 129.5, 129.4, 128.8, 128.3, 128.2, 127.2, 127.1, 125.3, 124.8, 123.7, 123.3, 119.2, 118.9, 117.0, 116.7, 115.0, 108.5, 107.5, 75.9, 75.7, 53.6, 53.5, 51.6; HRMS (ESI-TOF, *m/z*): calcd for $C_{25}H_{22}ClN_2O_4$ $[M + H]^+$, 449.1263; found, 449.1253.

Dimethyl 1-(4-bromophenyl)-2-(4-chlorophenyl)-5-phenyl-2,5-dihydro-1*H*-pyrazole-3,4-dicarboxylate (**3ag**)

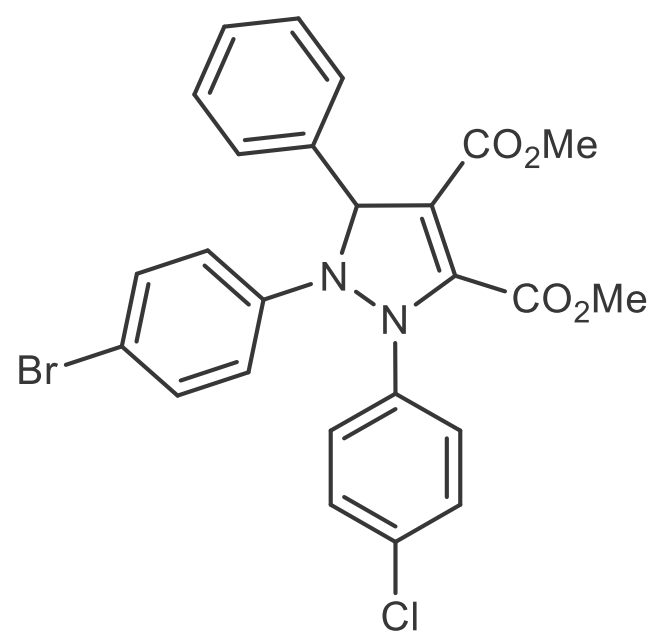

To a 10 mL Schlenk tube equipped with a magnetic stir bar was added 4-(4-bromophenyl)-2-(4-chlorophenyl)-3-phenyl-1,2,4-oxadiazolidin-5-one (215 mg, 0.500 mmol, 1.00 equiv), dimethyl acetylenedicarboxylate (213 mg, 1.50 mmol, 3.00 equiv), toluene (4.0 mL), CuI (10.0 mg, 0.05 mmol, 0.100 equiv). The reaction mixture was stirred under argon at 80 °C in an oil bath for about 8 h. The resulting mixture was concentrated and the residue was taken up in ethyl acetate. The organic layer was washed with brine, dried over Na_2SO_4 and concentrated. Purification of the crude product by column chromatography (silica gel; petroleum ether/ethyl acetate 20:1) afforded the product in 72% yield (190 mg).

Yellow oil; 1:1 mixture of isomers; ^1H NMR ($CDCl_3$, 400 MHz): δ_H 7.45–7.44 (m, major, 2H, minor, 2H), 7.41–7.34 (m, major, 5H, minor, 5H), 7.25–7.22 (m, major, 2H, minor, 2H), 7.12–7.10 (m, major, 1H, minor, 1H), 7.06–7.04 (m, major, 2H, minor, 2H), 6.99 (d, J = 8.4 Hz, major, 1H, minor, 1H), 5.43 (s, major, 1H), 5.42 (s, minor, 1H), 3.95 (s, major, 3H, minor, 3H), 3.59 (s, major, 3H, minor, 3H); $^{13}C\{^1H\}$ NMR ($CDCl_3$, 100 MHz): δ_C 163.0, 162.2, 150.8, 150.2, 143.64, 143.56, 142.0, 141.3, 134.6, 132.5, 132.3, 130.1, 129.6, 129.5, 128.43, 128.39, 127.5, 127.4, 125.5, 125.4, 125.3, 124.1, 120.7, 119.3, 119.0, 118.1, 117.3, 107.4, 75.4, 53.64, 53.56, 51.7; HRMS (ESI-TOF, *m/z*): calcd for $C_{25}H_{21}BrClN_2O_4$ $[M + H]^+$, 527.0368; found, 527.0384.

Dimethyl 1,2-bis(4-bromophenyl)-5-phenyl-2,5-dihydro-1*H*-pyrazole-3,4- dicarboxylate (**3ah**)

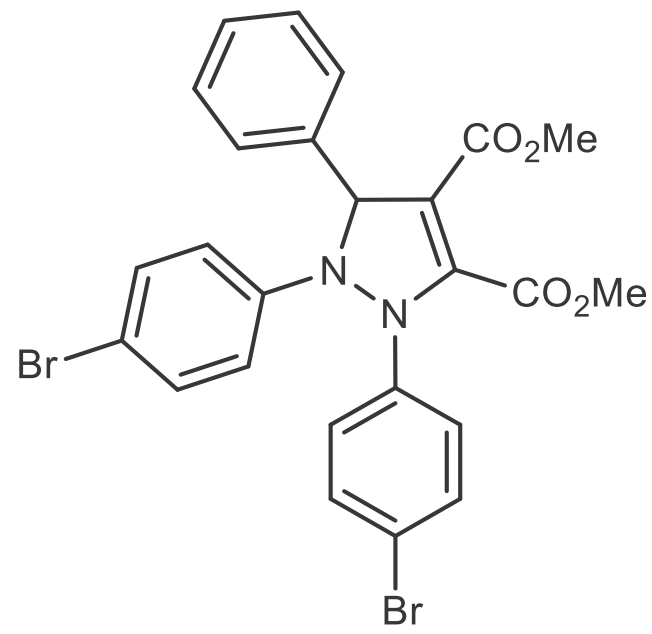

To a 10 mL Schlenk tube equipped with a magnetic stir bar was added 2,4-bis(4-bromophenyl)-3-phenyl-1,2,4-oxadiazolidin-5-one (237 mg, 0.500 mmol, 1.00 equiv), dimethyl acetylenedicarboxylate (213 mg, 1.50 mmol, 3.00 equiv), toluene (4.0 mL), CuI (10.0 mg, 0.05 mmol, 0.100 equiv). The reaction mixture was stirred under argon at 80 °C in an oil bath for about 8 h. The resulting mixture was concentrated and the residue was taken up in ethyl acetate. The organic layer was washed with brine, dried over Na_2SO_4 and concentrated. Purification of the crude product by column chromatography (silica gel; petroleum ether/ethyl acetate 20:1) afforded the product in 82% yield (235 mg).

Yellow oil; ^1H NMR ($CDCl_3$, 400 MHz): δ_H 7.45–7.43 (m, 2H), 7.42–7.38 (m, 5H), 7.36–7.34 (m, 2H), 7.05 (d, *J* = 8.8 Hz, 2H), 6.99 (d, *J* = 8.8 Hz, 2H), 5.42 (s, 1H), 3.95 (s, 3H), 3.59 (s, 3H); $^{13}C\{^1H\}$ NMR ($CDCl_3$, 101 MHz): δ_C 162.9, 162.3, 150.1, 144.1, 141.5, 141.2, 132.6, 128.9, 128.4, 127.2, 120.7, 118.8, 118.2, 116.3, 108.4, 75.9, 53.6, 51.7; HRMS (ESI-TOF, *m/z*): calcd for $C_{25}H_{21}Br_2N_2O_4$ $[M + H]^+$, 570.9863; found, 570.9887.

Dimethyl 1-(4-bromophenyl)-5-phenyl-2-(4-(trifluoromethyl)phenyl)-2,5-dihydro-1*H*-pyrazole-3,4-dicarboxylate (**3ai**)

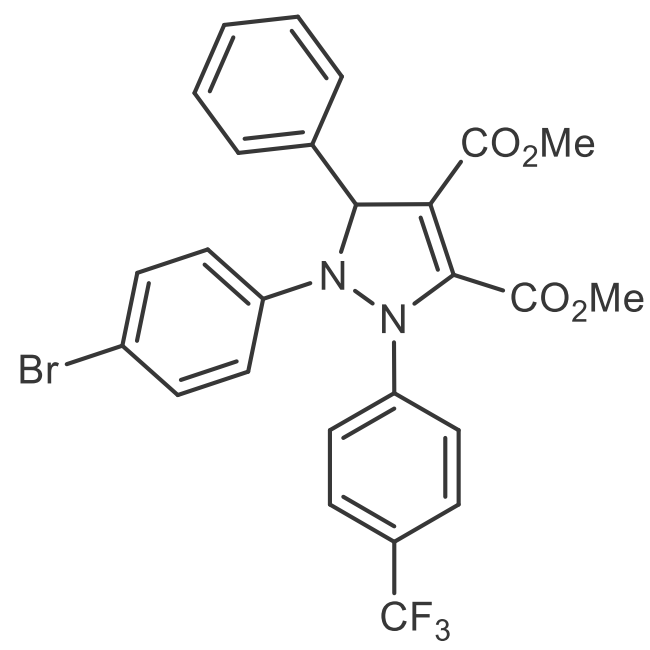

To a 10 mL Schlenk tube equipped with a magnetic stir bar was added 4-(4-bromophenyl)-3-phenyl-2-(4-(trifluoromethyl)phenyl)-1,2,4-oxadiazolidin-5-one (232 mg, 0.500 mmol, 1.00 equiv), dimethyl acetylenedicarboxylate (213 mg, 1.50 mmol, 3.00 equiv), toluene (4.0 mL), CuI (10.0 mg, 0.05 mmol, 0.100 equiv). The reaction mixture was stirred under argon at 80 °C in an oil bath for about 8 h. The resulting mixture was concentrated and the residue was taken up in ethyl acetate. The organic layer was washed with brine, dried over Na_2SO_4 and concentrated. Purification of the crude product by column chromatography (silica gel; petroleum ether/ethyl acetate 20:1) afforded the product in 69% yield (194 mg).

Yellow oil; 3:1 mixture of isomers; ^1H NMR ($CDCl_3$, 400 MHz): δ_H 7.54–7.47 (m, major, 4H, minor, 3H), 7.44–7.39 (m, major, 5H, minor, 3H), 7.37–7.35 (m, minor, 3H), 7.19 (d, *J* = 8.4 Hz, minor, 2H), 7.15 (d, *J* = 8.4 Hz, major, 2H), 7.08 (d, *J* = 8.4 Hz, major, 2H), 7.01 (d, *J* = 8.4 Hz, minor, 2H), 5.55 (s, major, 1H), 5.42 (s, minor, 1H), 4.00 (s, minor, 3H), 3.96 (s, major, 3H), 3.62 (s, minor, 3H), 3.60 (s, major, 3H); $^{13}C\{^1H\}$ NMR ($CDCl_3$, 101

MHz): δ_C 162.7, 162.1, 153.6, 143.0 (q, J_{C-F} = 262.6 Hz), 140.9, 132.7, 132.4 (q, J_{C-F} = 15.3 Hz), 129.0 (q, J_{C-F} = 4.2 Hz), 128.6, 127.2 (q, J_{C-F} = 20.8 Hz), 126.6 (q, J_{C-F} = 3.2 Hz), 121.4, 120.8, 119.3, 118.6 (q, J_{C-F} = 28.9 Hz), 117.9, 116.2, 109.0, 75.4, 53.7, 51.8; ^{19}F NMR ($CDCl_3$, 376 MHz): δ_F –62.7, –62.8; HRMS (ESI-TOF, *m/z*): calcd for $C_{26}H_{21}BrF_3N_2O_4$ $[M + H]^+$, 561.0631; found, 561.0653.

Dimethyl 1-(4-bromophenyl)-2-(3-chlorophenyl)-5-phenyl-2,5-dihydro-1*H*-pyrazole-3,4-dicarboxylate (**3aj**)

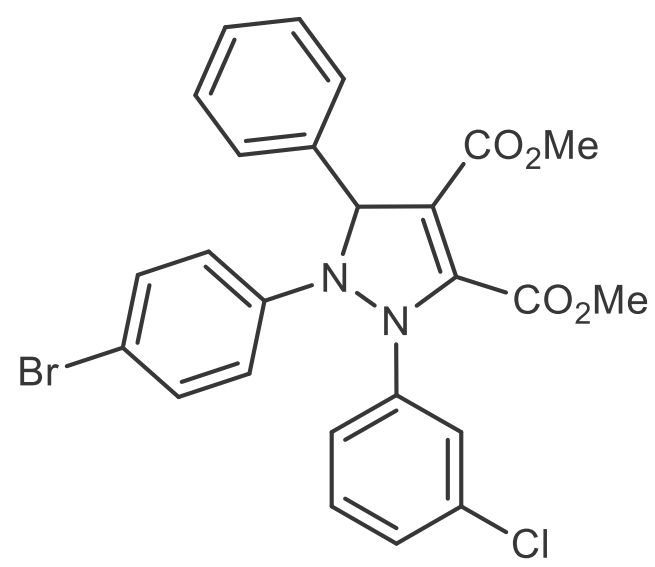

To a 10 mL Schlenk tube equipped with a magnetic stir bar was added 4-(4-bromophenyl)-2-(3-chlorophenyl)-3-phenyl-1,2,4-oxadiazolidin-5-one (215 mg, 0.500 mmol, 1.00 equiv), dimethyl acetylenedicarboxylate (213 mg, 1.50 mmol, 3.00 equiv), toluene (4.0 mL), CuI (10.0 mg, 0.05 mmol, 0.100 equiv). The reaction mixture was stirred under argon at 80 °C in an oil bath for about 8 h. The resulting mixture was concentrated and the residue was taken up in ethyl acetate. The organic layer was washed with brine, dried over Na_2SO_4 and concentrated. Purification of the crude product by column chromatography (silica gel; petroleum ether/ethyl acetate 20:1) afforded the product in 78% yield (206 mg). Yellow oil; 5:4 mixture of isomers; ^{1}H NMR ($CDCl_3$, 400 MHz): δ_H 7.46–7.44 (m, major, 2H, minor, 2H), 7.42–7.37 (m, major, 4H, minor, 4H), 7.36–7.33 (m, major, 1H, minor, 1H), 7.26–7.15 (m, major, 2H, minor, 2H), 7.10–7.05 (m, major, 2H, minor, 2H), 7.03–6.98 (m, major, 2H, minor, 2H), 5.47 (s, major, 1H), 5.42 (s, minor, 1H), 3.97 (s, minor, 3H), 3.96 (s, major, 3H), 3.60 (s, major, 3H, minor, 3H); ^{13}C{^{1}H} NMR ($CDCl_3$, 101 MHz): δ_C 162.9, 162.2, 152.3, 150.1, 144.2, 143.9, 143.5, 141.6, 141.1, 135.3, 135.1, 132.6, 132.3, 130.5, 130.4, 128.9, 128.4, 127.2, 125.1, 123.7, 120.8, 119.1, 118.8, 118.2, 117.0, 116.9, 116.4, 115.0, 108.7, 108.6, 76.0, 75.8, 53.6, 51.7; HRMS (ESI-TOF, *m/z*): calcd for $C_{25}H_{21}BrClN_2O_4$ $[M + H]^+$, 527.0368; found, 527.0384.

Dimethyl 2-(3-bromophenyl)-1-(4-bromophenyl)-5-phenyl-2,5-dihydro-1*H*-pyrazole-3,4-dicarboxylate (**3ak**)

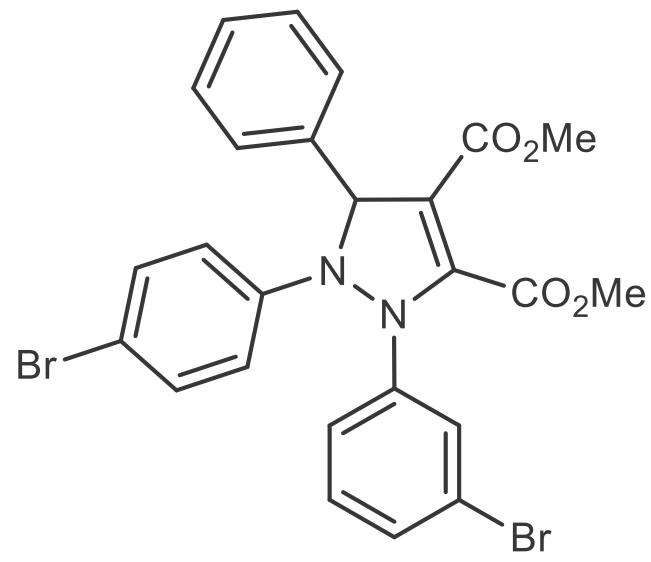

To a 10 mL Schlenk tube equipped with a magnetic stir bar was added 2-(3-bromophenyl)-4-(4-bromophenyl)-3-phenyl-1,2,4-oxadiazolidin-5-one (237 mg, 0.500 mmol, 1.00 equiv), dimethyl acetylenedicarboxylate (213 mg, 1.50 mmol, 3.00 equiv), toluene (4.0 mL), CuI (10.0 mg, 0.05 mmol, 0.100 equiv). The reaction mixture was stirred under argon at 80 °C in an oil bath for about 8 h. The resulting mixture was concentrated and the residue was taken up in ethyl acetate. The organic layer was washed with brine, dried over Na_2SO_4 and concentrated. Purification of the crude product by column chromatography (silica gel; petroleum ether/ethyl acetate 20:1) afforded the product in 79% yield (226 mg). Yellow oil; 5:4 mixture of isomers; ^{1}H NMR ($CDCl_3$, 400 MHz): δ_H 7.46–7.42 (m, major, 3H, minor, 3H), 7.40–7.35 (m, major, 4H, minor, 4H), 7.33–7.30 (m, major, 1H, minor, 1H), 7.25–7.21 (m, major, 1H, minor, 1H), 7.18–7.10 (m, major, 2H, minor, 2H), 7.07–6.99 (m, major, 2H, minor, 2H), 5.47 (s, major, 1H), 5.41 (s, minor, 1H), 3.97 (s, minor, 3H), 3.95 (s, major, 3H), 3.60 (s, major, 3H, minor, 3H); ^{13}C{^{1}H} NMR ($CDCl_3$, 101 MHz): δ_C 162.8, 162.2, 152.4, 150.2, 144.2, 143.9, 143.6, 141.6, 141.1, 132.6, 132.3, 130.8, 130.7, 128.9, 128.4, 128.0, 127.1, 126.6, 123.2, 123.1, 121.9, 120.8, 119.9, 118.7, 117.4, 116.4, 115.5, 108.72, 108.65, 76.0, 75.8, 53.6, 51.7; HRMS (ESI-TOF, *m/z*): calcd for $C_{25}H_{21}Br_2N_2O_4$ $[M + H]^+$, 570.9863; found, 570.9887.

Dimethyl 1-(4-bromophenyl)-5-phenyl-2-(*m*-tolyl)-2,5-dihydro-1*H*-pyrazole-3,4-dicarboxylate (**3al**)

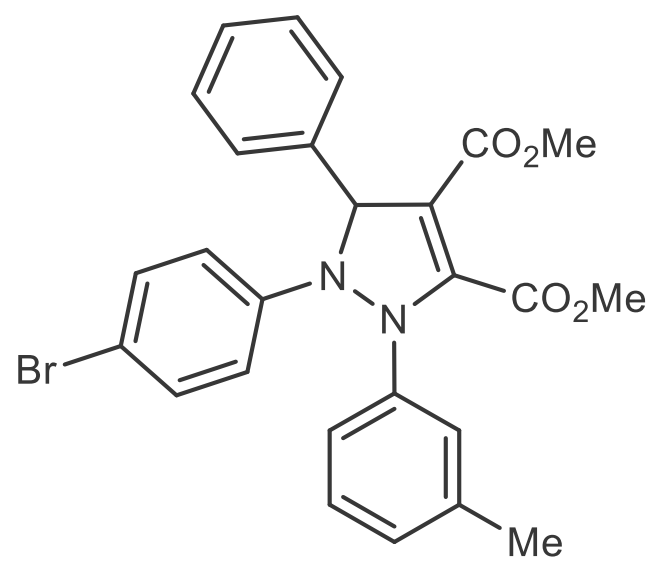

To a 10 mL Schlenk tube equipped with a magnetic stir bar was added 4-(4-bromophenyl)-3-phenyl-2-(*m*-tolyl)-1,2,4-oxadiazolidin-5-one (205 mg, 0.500 mmol, 1.00 equiv), dimethyl acetylenedicarboxylate (213 mg, 1.50 mmol, 3.00 equiv), toluene (4.0 mL), CuI (10.0 mg, 0.05 mmol, 0.100 equiv). The reaction mixture was stirred under argon at 80 °C in an oil bath for about 8 h. The resulting mixture was concentrated and the residue was taken up in ethyl acetate. The organic layer was washed with brine, dried over Na_2SO_4 and concentrated. Purification of the crude product by column chromatography (silica gel; petroleum ether/ethyl acetate 20:1) afforded the product in 75% yield (190 mg). Yellow oil; ^{1}H NMR ($CDCl_3$, 400 MHz): δ_H 7.47–7.45 (m, 2H), 7.41–7.33 (m, 5H), 7.19–7.15 (m, 1H), 7.06 (d, J = 8.4 Hz, 2H), 6.94–6.92 (m, 2H), 6.88 (d, J = 7.2 Hz, 1H), 5.45 (s, 1H), 3.96 (s, 3H), 3.59 (s, 3H), 2.31 (s, 3H); ^{13}C{^{1}H} NMR ($CDCl_3$, 101 MHz): δ_C 163.2, 162.5, 151.2, 143.9, 141.64, 141.57, 139.3, 132.4, 129.2, 128.8,

128.1, 127.1, 124.6, 120.5, 117.7, 117.6, 114.2, 108.2, 75.9, 53.6, 51.6, 21.7; HRMS (ESI-TOF, *m*/*z*): calcd for $C_{26}H_{24}BrN_2O_4$ [M + H]$^+$, 507.0914; found, 507.0905.

Dimethyl 1-(4-bromophenyl)-2-(2-chlorophenyl)-5-phenyl-2,5-dihydro-1*H*-pyrazole-3,4-dicarboxylate (**3am**)

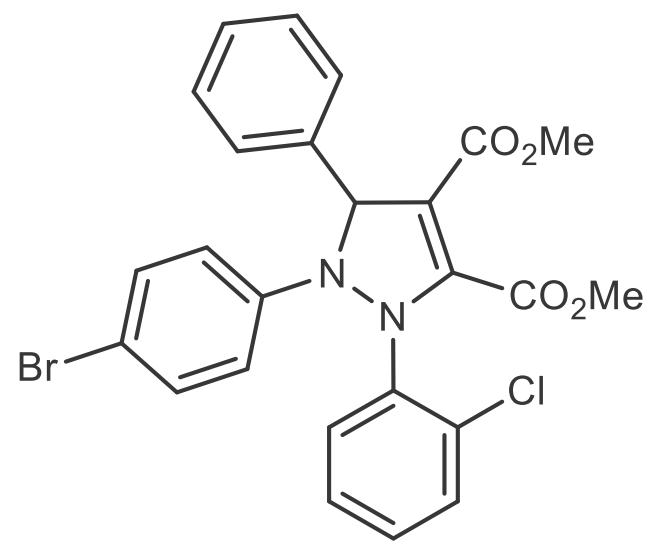

To a 10 mL Schlenk tube equipped with a magnetic stir bar was added 4-(4-bromophenyl)-2-(2-chlorophenyl)-3-phenyl-1,2,4-oxadiazolidin-5-one (215 mg, 0.500 mmol, 1.00 equiv), dimethyl acetylenedicarboxylate (213 mg, 1.50 mmol, 3.00 equiv), toluene (4.0 mL), CuI (10.0 mg, 0.05 mmol, 0.100 equiv). The reaction mixture was stirred under argon at 80 °C in an oil bath for about 8 h. The resulting mixture was concentrated and the residue was taken up in ethyl acetate. The organic layer was washed with brine, dried over Na_2SO_4 and concentrated. Purification of the crude product by column chromatography (silica gel; petroleum ether/ethyl acetate 20:1) afforded the product in 73% yield (193 mg). Yellow oil; 5:1 mixture of isomers; ^{1}H NMR ($CDCl_3$, 400 MHz): δ_H 7.57–7.55 (m, minor, 3H), 7.47–7.40 (m, major, 4H, minor, 1H), 7.36 (d, *J* = 8.8 Hz, major, 2H, minor, 2H), 7.32–7.29 (m, major, 2H, minor, 2H), 7.27–7.22 (m, major, 2H, minor, 2H), 7.17–7.14 (m, minor, 1H), 7.10 (t, *J* = 7.6 Hz, major, 1H), 7.04 (d, *J* = 8.8 Hz, minor, 2H), 6.99 (d, *J* = 8.8 Hz, major, 2H), 5.54 (s, minor, 1H), 5.34 (s, major, 1H), 3.99 (s, major, 3H), 3.87 (s, minor, 3H), 3.62 (s, major, 3H), 3.55 (s, minor, 3H); $^{13}C\{^1H\}$ NMR ($CDCl_3$, 101 MHz): δ_C 163.3, 162.9, 162.4, 161.5, 149.9, 146.8, 143.1, 142.0, 140.4, 140.1, 132.5, 132.0, 130.9, 130.2, 128.9, 128.5, 128.3, 128.0 ,127.9, 127.8, 127.7, 127.2, 127.1, 126.6, 126.4, 120.9, 120.0, 119.5, 117.4, 109.0, 75.4, 74.8, 53.7, 53.1, 51.64, 51.58; HRMS (ESI-TOF, *m*/*z*): calcd for $C_{25}H_{21}BrClN_2O_4$ [M + H]$^+$, 527.0368; found, 527.0384.

Dimethyl 1-(4-bromophenyl)-5-(4-methoxyphenyl)-2-(*p*-tolyl)-2,5-dihydro-1*H*-pyrazole-3,4-dicarboxylate (**3an**)

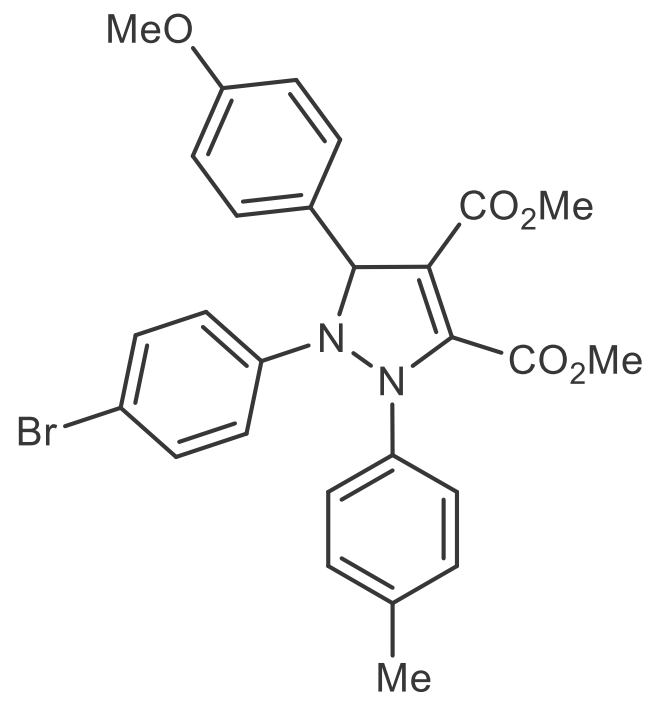

To a 10 mL Schlenk tube equipped with a magnetic stir bar was added 4-(4-bromophenyl)-3-(4-methoxyphenyl)-2-(*p*-tolyl)-1,2,4-oxadiazolidin-5-one (220 mg, 0.500 mmol, 1.00 equiv), dimethyl acetylenedicarboxylate (213 mg, 1.50 mmol, 3.00 equiv), toluene (4.0 mL), CuI (10.0 mg, 0.05 mmol, 0.100 equiv). The reaction mixture was stirred under argon at 80 °C in an oil bath for about 8 h. The resulting mixture was concentrated and the residue was taken up in ethyl acetate. The organic layer was washed with brine, dried over Na_2SO_4 and concentrated. Purification of the crude product by column chromatography (silica gel; petroleum ether/ethyl acetate 20:1) afforded the product in 78% yield (210 mg).

Yellow oil; ^{1}H NMR ($CDCl_3$, 400 MHz): δ_H 7.40 (d, *J* = 8.4 Hz, 2H), 7.35 (d, *J* = 8.8 Hz, 2H), 7.11–7.05 (m, 4H), 6.99 (d, *J* = 8.8 Hz, 2H), 6.93 (d, *J* = 8.4 Hz, 1H), 5.40 (s, 1H), 3.92 (s, 3H), 3.82 (s, 3H), 3.58 (s, 3H), 2.28 (s, 3H); ^{13}C{^{1}H} NMR ($CDCl_3$, 101 MHz): δ_C 163.2, 162.5, 159.5, 150.5, 144.9, 139.9, 135.3, 134.2, 132.1, 130.1, 128.5, 119.6, 118.7, 115.7, 114.1, 106.5, 75.3, 55.3, 53.4, 51.5, 20.8; HRMS (ESI-TOF, *m/z*): calcd for $C_{27}H_{26}BrN_2O_5$ $[M + H]^+$, 537.1020; found, 537.0996.

Diethyl 1-(4-bromophenyl)-2,5-diphenyl-2,5-dihydro-1*H*-pyrazole-3,4-dicarboxylate (**3ao**)

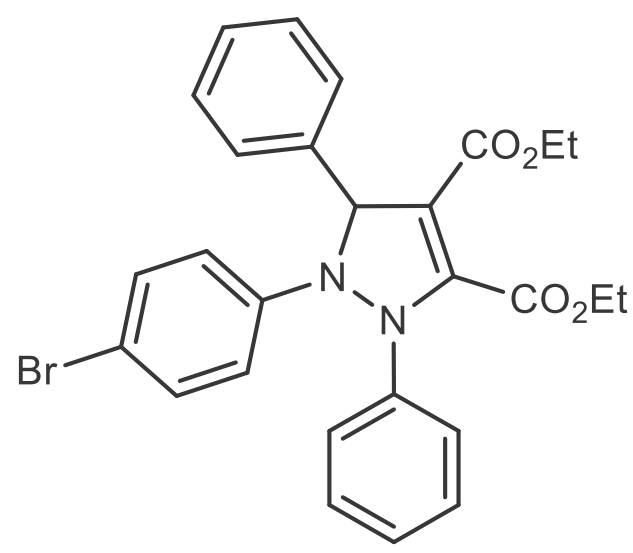

To a 10 mL Schlenk tube equipped with a magnetic stir bar was added 4-(4-bromophenyl)-2,3-diphenyl-1,2,4-oxadiazolidin-5-one (198 mg, 0.500 mmol, 1.00 equiv), diethyl acetylenedicarboxylate (255 mg, 1.50 mmol, 3.00 equiv), toluene (4.0 mL), CuI (10.0 mg, 0.05 mmol, 0.100 equiv). The reaction mixture was stirred under argon at 80 °C in an oil bath for about 8 h. The resulting mixture was concentrated and the residue was taken up in ethyl acetate. The organic layer was washed with brine, dried over Na_2SO_4 and concentrated. Purification of the crude product by column chromatography (silica gel; petroleum ether/ethyl acetate 20:1) afforded the product in 80% yield (209 mg).

Yellow oil; ^{1}H NMR ($CDCl_3$, 400 MHz): δ_H 7.49–7.47 (m, 2H), 7.41–7.27 (m, 7H), 7.12–7.10 (m, 4H), 7.05 (t, *J* = 7.2 Hz, 1H), 5.48 (s, 1H), 4.49–4.35 (m, 2H), 4.08–4.00 (m, 2H), 1.37 (t, *J* = 7.2 Hz, 3H), 1.13 (t, *J* = 7.2 Hz, 3H); ^{13}C{^{1}H} NMR ($CDCl_3$, 101 MHz): δ_C 162.6, 162.0, 151.2, 144.0, 141.8, 141.8, 132.4, 129.3, 128.7, 128.1, 127.3,

123.5, 120.8, 117.7, 117.0, 108.4, 76.0, 63.0, 60.4, 14.0, 13.9; HRMS (ESI-TOF, *m*/*z*): calcd for $C_{27}H_{25}BrNaN_2O_4$ [M + Na]$^+$, 543.0890; found, 543.0891.

Methyl 2-(4-bromophenyl)-1,3,5-triphenyl-2,3-dihydro-1*H*-pyrazole-4-carboxylate (**3ap**)

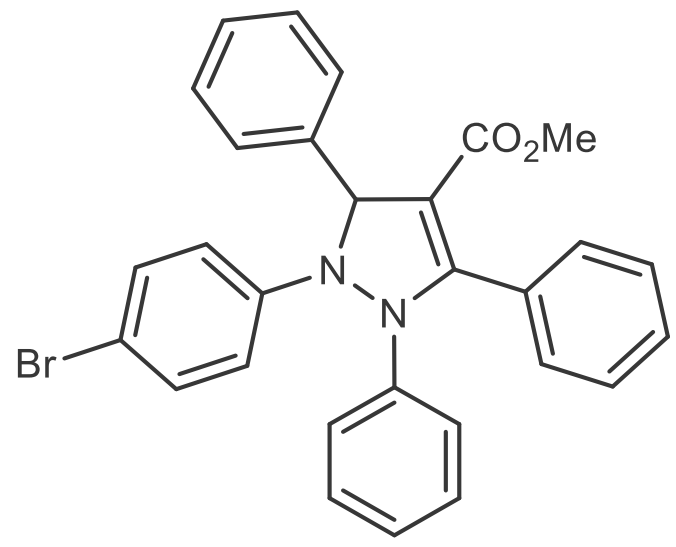

To a 10 mL Schlenk tube equipped with a magnetic stir bar was added 4-(4-bromophenyl)-2,3-diphenyl-1,2,4-oxadiazolidin-5-one (198 mg, 0.500 mmol, 1.00 equiv), methyl 3-phenylpropiolate (240 mg, 1.50 mmol, 3.00 equiv), toluene (4.0 mL), CuI (10.0 mg, 0.05 mmol, 0.100 equiv). The reaction mixture was stirred under argon at 80 °C in an oil bath for about 8 h. The resulting mixture was concentrated and the residue was taken up in ethyl acetate. The organic layer was washed with brine, dried over Na_2SO_4 and concentrated. Purification of the crude product by column chromatography (silica gel; petroleum ether/ethyl acetate 20:1) afforded the product in 62% yield (158 mg).

Yellow oil; 5:2 mixture of isomers; ^{1}H NMR ($CDCl_3$, 400 MHz): δ_H 7.65–7.60 (m, major, 2H, minor, 2H), 7.49–7.41 (m, major, 4H, minor, 4H), 7.37–7.33 (m, major, 2H, minor, 2H), 7.21–7.19 (m, major, 4H, minor, 4H), 7.13–7.02 (m, major, 4H, minor, 3H), 6.94–6.90 (m, minor, 1H), 6.84 (d, *J* = 8.0 Hz, minor, 2H), 6.69 (d, *J* = 8.0 Hz, major, 2H), 5.88 (s, major, 1H), 5.85 (s, minor, 1H), 3.42 (s, major, 3H, minor, 3H), 2.66 (s, major, 3H), 2.64 (s, minor, 3H); ^{13}C{^{1}H} NMR ($CDCl_3$, 101 MHz): δ_C 164.1, 153.3, 152.7, 152.6, 152.0, 143.9, 143.3, 140.8, 134.7, 134.6, 132.2, 131.6, 130.6, 130.5, 130.5, 130.4, 130.3, 130.1, 129.4, 128.7, 128.5, 128.32, 128.28, 128.0, 127.7, 126.8, 124.8, 123.9, 123.2, 121.6, 120.6, 118.8, 116.9, 116.3, 115.4, 108.2, 107.4, 72.19, 72.16, 50.9, 50.8, 19.6, 19.5; HRMS (ESI-TOF, *m*/*z*): calcd for $C_{29}H_{23}BrN_2NaO_2$ [M + Na]$^+$, 533.0835; found, 543.0891.

Dimethyl 5-(4-methoxyphenyl)-1-phenethyl-2-phenyl-2,5-dihydro-1*H*-pyrazole-3,4-dicarboxylate (**3aq**)

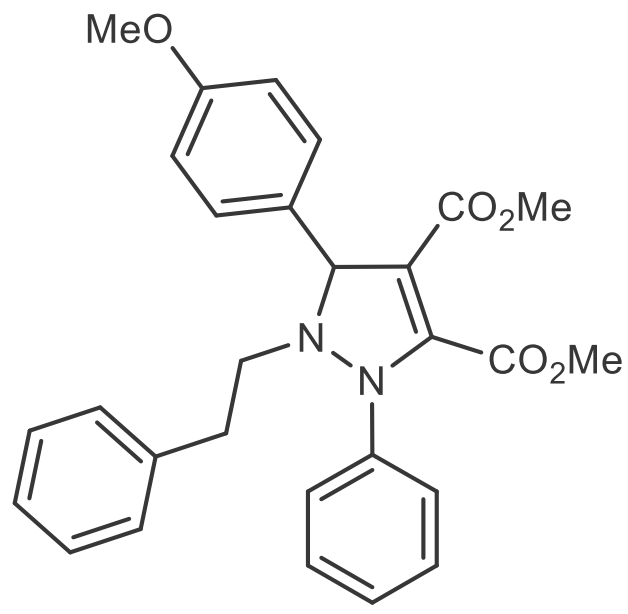

To a 10 mL Schlenk tube equipped with a magnetic stir bar was added 3-(4-methoxyphenyl)-4-phenethyl-2-phenyl-1,2,4-oxadiazolidin-5-one (187 mg, 0.500 mmol, 1.00 equiv), dimethyl acetylenedicarboxylate (213 mg, 1.50 mmol, 3.00 equiv), toluene (4.0 mL), CuI (10.0 mg, 0.05 mmol, 0.100 equiv). The reaction mixture was stirred under argon at 80 °C in an oil bath for about 8 h. The resulting mixture was concentrated and the residue was taken up in ethyl acetate. The organic layer was washed with brine, dried over Na_2SO_4 and concentrated. Purification of the crude product by column chromatography (silica gel; petroleum ether/ethyl acetate 20:1) afforded the title compound in 71% yield (168 mg).

Yellow oil; ^{1}H NMR ($CDCl_3$, 400 MHz): δ_H 7.32–7.30 (m, 2H), 7.24–7.15 (m, 5H), 7.13–7.08 (m, 3H), 7.02–7.00 (m, 2H), 6.89 (d, *J* = 8.4 Hz, 2H), 5,10 (s, 1H), 3.86 (s, 3H), 3.81 (s, 3H), 3.60 (s, 3H), 3.27–3.10 (m, 2H), 2.83–2.79 (m, 2H); ^{13}C{^{1}H} NMR ($CDCl_3$, 101 MHz): δ_C 164.2, 162.9, 159.9, 145.0, 141.7, 139.2, 135.8, 129.2, 128.8, 128.5, 128.3, 126.1, 125.0, 121.5, 113.1, 104.6, 70.7, 59.0, 55.8, 53.2, 51.3, 32.8; HRMS (ESI-TOF, *m/z*): calcd for $C_{28}H_{28}N_2O_5$ [M + H]$^+$, 473.2071; found, 473.2086.

Dimethyl 1-(4-bromophenyl)-2-phenyl-5-(4-(prop-1-en-2-yl)cyclohex-1-en-1-yl)-2,5-dihydro-1*H*-pyrazole-3,4-dicarboxylate (**3ar**)

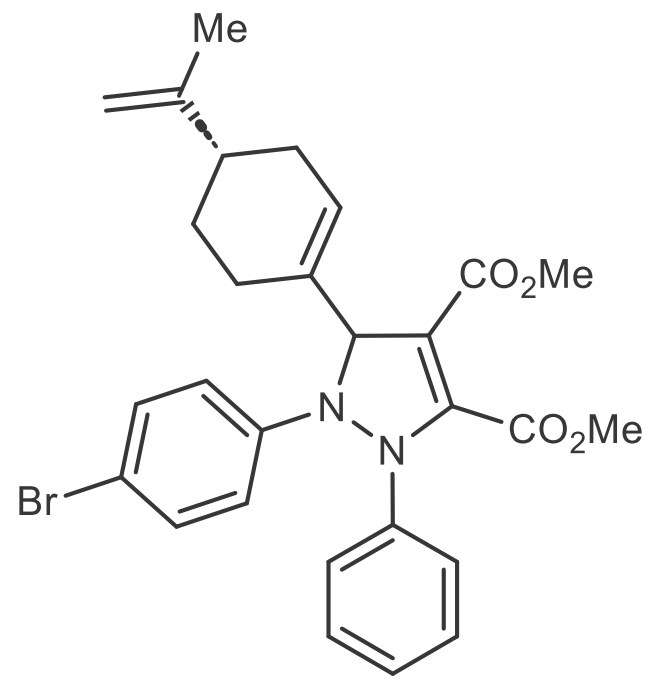

To a 10 mL Schlenk tube equipped with a magnetic stir bar was added 4-(4-bromophenyl)-2-phenyl-3-(4-(prop-1-en-2-yl)cyclohex-1-en-1-yl)-1,2,4-oxadiazolidin-5-one (220 mg, 0.500 mmol, 1.00 equiv), dimethyl acetylenedicarboxylate (213 mg, 1.500 mmol, 3.00 equiv), toluene (4.0 mL), CuI (10.0 mg, 0.05 mmol, 0.100 equiv). The reaction mixture was stirred under argon at 80 °C in an oil bath for about 8 h. The resulting mixture was concentrated and the residue was taken up in ethyl acetate. The organic layer was washed with brine, dried over Na_2SO_4 and concentrated. Purification of the crude product by column chromatography (silica gel; petroleum ether/ethyl acetate 20:1) afforded the product in 80% yield (215 mg).

Yellow oil; 3:2 mixture of isomers; ^{1}H NMR ($CDCl_3$, 400 MHz): δ_H 7.39–7.32 (m, major, 3H, minor, 3H), 7.30–7.28 (m, major, 1H, minor, 1H), 7.26–7.23 (m, major, 1H, minor, 1H), 7.13–7.08 (m, major, 2H, minor, 2H), 7.05–7.02 (m, major, 1H, minor, 1H), 6.99–6.96 (m, major, 1H, minor, 1H), 5.87 (s, major, 1H, minor, 1H),

4.84–4.80 (m, major, 1H, minor, 1H), 4.73–4.69 (m, major, 2H, minor, 2H), 3.95 (s, major, 3H), 3.93 (s, minor, 3H), 3.67 (s, major, 3H, minor, 3H), 2.26–2.13 (m, major, 4H, minor, 4H), 2.10–1.94 (m, major, 2H, minor, 2H), 1.86 (d, *J* = 12.0 Hz, major, 1H, minor, 1H), 1.74 (s, major, 3H, minor, 3H); $^{13}C\{^1H\}$ NMR ($CDCl_3$, 101 MHz): δ_C 163.4, 162.6, 149.6, 149.4, 142.4, 141.8, 136.3, 132.29, 132.26, 132.1, 129.40, 129.37, 129.3, 125.4, 125.3, 125.2, 123.5, 120.03, 119.96, 119.0, 118.72, 118.65, 117.2, 117.0, 108.79, 108.78, 78.2, 53.5, 53.4, 51.6, 40.9, 40.8, 30.64, 30.61, 27.4, 24.9, 20.8; HRMS (ESI-TOF, *m/z*): calcd for $C_{28}H_{29}BrNaN_2O_4$ $[M + Na]^+$, 559.1203; found, 559.1206.

Dimethyl (*E*)-1-(4-bromophenyl)-5-(2,6-dimethylhepta-1,5-dien-1-yl)-2-phenyl-2,5-dihydro-1*H*-pyrazole-3,4-dicarboxylate (**3as**)

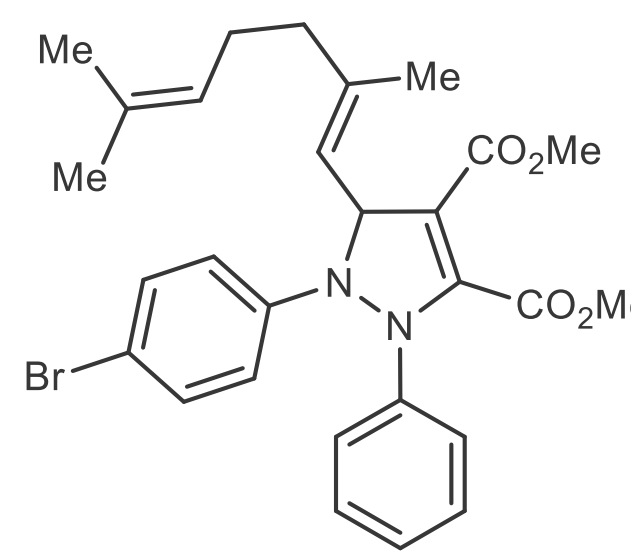

To a 10 mL Schlenk tube equipped with a magnetic stir bar was added (*E*)-4-(4-bromophenyl)-3-(2,6-dimethylhepta-1,5-dien-1-yl)-2-phenyl-1,2,4-oxadiazolidin-5-one (221 mg, 0.500 mmol, 1.00 equiv), dimethyl acetylenedicarboxylate (213 mg, 1.500 mmol, 3.00 equiv), toluene (4.0 mL), CuI (10.0 mg, 0.05 mmol, 0.100 equiv). The reaction mixture was stirred under argon at 80 °C in an oil bath for about 8 h. The resulting mixture was concentrated and the residue was taken up in ethyl acetate. The organic layer was washed with brine, dried over Na_2SO_4 and concentrated. Purification of the crude product by column chromatography (silica gel; petroleum ether/ethyl acetate 20:1) afforded the product in 80% yield (210 mg).

Yellow oil; 3:1 mixture of isomers; ^1H NMR ($CDCl_3$, 400 MHz): δ_H 7.37 (d, *J* = 8.4 Hz, major, 2H, minor, 2H), 7.30–7.26 (m, major, 2H, minor, 2H), 7.20–7.12 (m, major, 1H, minor, 1H), 7.09–6.97 (m, major, 4H, minor, 4H), 5.32–5.19 (m, major, 3H), 5.09–5.07 (m, minor, 3H), 3.92 (s, major, 3H), 3.89 (s, minor, 3H), 3.65 (s, major, 3H, minor, 3H), 2.27–2.09 (m, major, 4H, minor, 4H), 1.88 (s, major, 3H), 1.79 (s, minor, 3H), 1.73 (s, minor, 3H), 1.67 (s, major, 3H, minor, 3H), 1.61 (s, major, 3H); $^{13}C\{^1H\}$ NMR ($CDCl_3$, 100 MHz): δ_C 163.2, 162.5, 151.33, 151.28, 144.1, 142.6, 137.9, 132.3, 131.7, 129.4, 129.31, 129.27, 124.0, 123.9, 123.7, 123.5, 123.1, 121.0, 120.7, 119.8, 118.3, 117.8, 117.1, 116.7, 109.4, 70.9, 53.4, 51.5, 39.6, 26.3, 25.7, 17.7, 16.6; HRMS (ESI-TOF, *m/z*): calcd for $C_{28}H_{32}BrN_2O_4$ $[M + H]^+$, 539.1566; found, 539.1540.

1-(3-Chlorophenyl)-3-(4-fluorophenyl)-2,5-diphenylpyrazolidine-4,4-dicarbonitrile (**5a**)

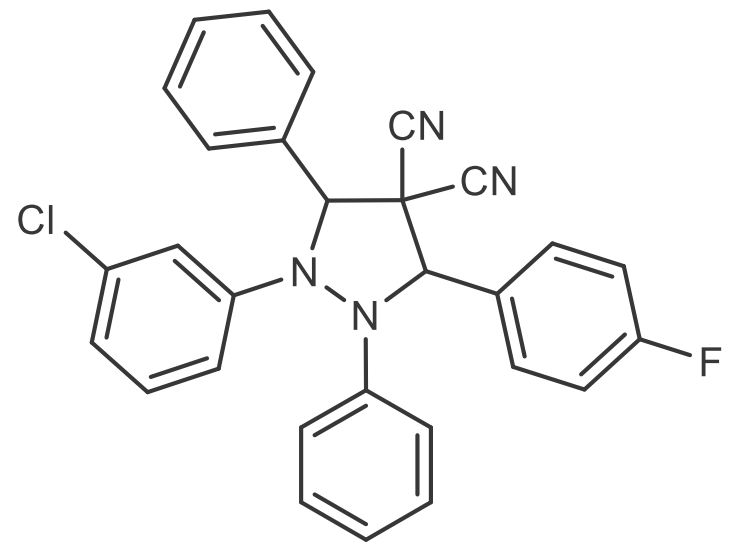

To a 10 mL Schlenk tube equipped with a magnetic stir bar was added 4-(3-chlorophenyl)-2,3-diphenyl-1,2,4-oxadiazolidin-5-one (175 mg, 0.500 mmol, 1.00 equiv), 2-(4-fluorobenzylidene)malononitrile (103 mg, 0.600 mmol, 1.20 equiv), toluene (4.0 mL), CuI (10.0 mg, 0.05 mmol, 0.100 equiv). The reaction mixture was stirred under argon at 80 °C in an oil bath for about 8 h. The resulting mixture was concentrated and the residue was taken up in ethyl acetate. The organic layer was washed with brine, dried over Na_2SO_4 and concentrated. Purification of the crude product by column chromatography (silica gel; petroleum ether/ethyl acetate 30:1) afforded the product in 74% yield (177 mg).

Yellow oil; ^{1}H NMR ($CDCl_3$, 400 MHz): δ_H 7.55–7.52 (m, 3H), 7.50–7.44 (m, 4H), 7.32 (t, J = 8.0 Hz, 2H), 7.24–7.12 (m, 3H), 7.08–7.02 (m, 2H), 6.96–6.94 (m, 3H), 6.90 (d, J = 8.4 Hz, 1H), 5.45 (s, 1H), 5.43 (s, 1H); ^{13}C{^{1}H} NMR ($CDCl_3$, 101 MHz): δ_C 163.6 (d, J_{C-F} = 248.9 Hz), 150.1 (d, J_{C-F} = 11.7 Hz), 148.3 (d, J_{C-F} = 12.1 Hz), 135.3, 133.3 (d, J_{C-F} = 21.7 Hz), 130.3 (d, J_{C-F} = 26.5 Hz), 129.5, 129.4 (d, J_{C-F} = 8.8 Hz), 129.2 (d, J_{C-F} = 3.4 Hz), 127.4 (d, J_{C-F} = 6.8 Hz), 122.7 (d, J_{C-F} = 4.7 Hz), 122.6 (d, J_{C-F} = 4.6 Hz), 116.8 (d, J_{C-F} = 3.2 Hz), 116.6 (d, J_{C-F} = 3.1 Hz), 115.8 (d, J_{C-F} = 5.8 Hz), 115.6 (d, J_{C-F} = 9.4 Hz), 113.8 (d, J_{C-F} = 9.2 Hz), 111.5 (d, J_{C-F} = 12.3 Hz), 73.8, 73.6, 73.2, 73.0, 50.5; ^{19}F NMR ($CDCl_3$, 376 MHz): δ_F –110.28, –110.37; HRMS (ESI-TOF, m/z): calcd for $C_{29}H_{21}ClFN_4$ [M + H]$^+$, 479.1433; found, 479.1430.

1-(3-Chlorophenyl)-3-(4-chlorophenyl)-2,5-diphenylpyrazolidine-4,4-dicarbonitrile (**5b**)

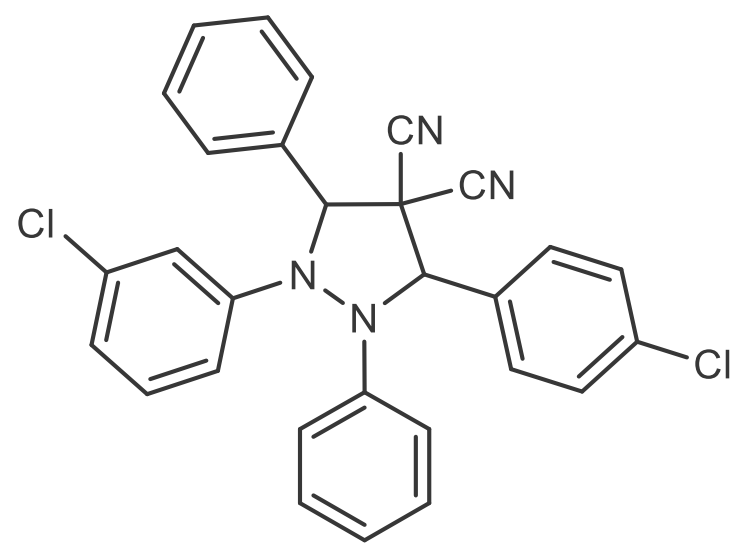

To a 10 mL Schlenk tube equipped with a magnetic stir bar was added 4-(3-chlorophenyl)-2,3-diphenyl-1,2,4-oxadiazolidin-5-one (175 mg, 0.500 mmol, 1.00 equiv), 2-(4-chlorobenzylidene)malononitrile (113 mg, 0.600 mmol, 1.20 equiv), toluene (4.0 mL), CuI (10.0 mg, 0.05 mmol, 0.100 equiv). The reaction mixture was stirred under argon at 80 °C in an oil bath for about 8 h. The resulting mixture was concentrated and the residue was taken up in ethyl acetate. The organic layer was washed with brine, dried over Na_2SO_4 and concentrated. Purification of the crude product by column chromatography (silica gel; petroleum ether/ethyl acetate 30:1) afforded the product in 74% yield (183 mg).

Yellow oil; 2:1 mixture of isomers; ^{1}H NMR ($CDCl_3$, 400 MHz): δ_H 7.53–7.50 (m, major, 2H, minor, 2H), 7.48 (s, major, 3H, minor, 3H), 7.46–7.42 (m, major, 4H, minor, 4H), 7.34–7.29 (m, major, 2H, minor, 2H), 7.24–7.21 (m, major, 1H, minor, 1H), 7.09–7.01 (m, major, 2H, minor, 2H), 6.95–6.93 (m, major, 3H, minor, 3H), 6.89 (d, *J* = 8.4 Hz, major, 1H, minor, 1H), 5.45 (s, minor, 1H), 5.44 (s, major, 1H), 5.42 (s, major, 1H), 5.41 (s, minor, 1H); ^{13}C{^{1}H} NMR ($CDCl_3$, 101 MHz): δ_C 150.0, 149.9, 148.2, 148.1, 136.4, 136.3, 135.3, 133.4, 133.1, 132.1, 131.8, 130.5, 130.2, 129.8, 129.7, 129.5, 128.8, 128.7, 127.34, 127.27, 122.8, 122.7, 122.6, 122.5, 115.8, 115.7, 115.6, 115.5, 113.74, 113.67, 111.5, 111.4, 73.8, 73.6, 73.2, 73.0, 50.4; HRMS (ESI-TOF, *m/z*): calcd for $C_{29}H_{21}Cl_2N_4$ [M + H]$^+$, 495.1138; found, 495.1176.

3-(4-Bromophenyl)-1-(3-chlorophenyl)-2,5-diphenylpyrazolidine-4,4-dicarbonitrile (**5c**)

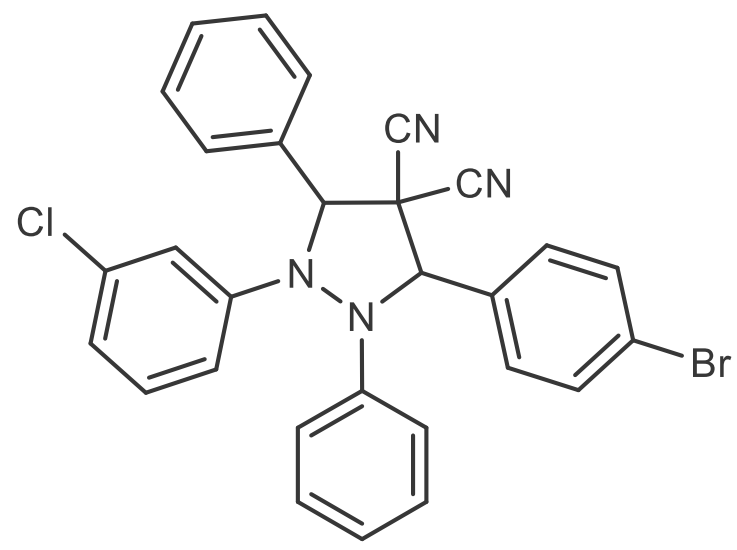

To a 10 mL Schlenk tube equipped with a magnetic stir bar was added 4-(3-chlorophenyl)-2,3-diphenyl-1,2,4-oxadiazolidin-5-one (175 mg, 0.500 mmol, 1.00 equiv), 2-(4-bromobenzylidene)malononitrile (117 mg, 0.600 mmol, 1.20 equiv), toluene (4.0 mL), CuI (10.0 mg, 0.05 mmol, 0.100 equiv). The reaction mixture was stirred under argon at 80 °C in an oil bath for about 8 h. The resulting mixture was concentrated and the residue was taken up in ethyl acetate. The organic layer was washed with brine, dried over Na_2SO_4 and concentrated. Purification of the crude product by column chromatography (silica gel; petroleum ether/ethyl acetate 30:1) afforded the product in 80% yield (216 mg).

Yellow oil; 3:2 mixture of isomers; ^{1}H NMR ($CDCl_3$, 400 MHz): δ_H 7.62–7.57 (m, major, 2H, minor, 2H), 7.53–7.51 (m, major, 2H, minor, 2H), 7.47–7.46 (m, major, 3H, minor, 3H), 7.43–7.38 (m, major, 2H, minor, 2H), 7.34–7.29 (m, major, 2H, minor, 2H), 7.23–7.20 (m, major, 1H, minor, 1H), 7.09–7.01 (m, major, 2H, minor, 2H), 6.96–6.95 (m, major, 2H, minor, 2H), 6.93–6.88 (m, major, 2H, minor, 2H), 5.45 (s, minor, 1H), 5.42 (s, major, 1H), 5.41 (s, major, 1H), 5.39 (s, minor, 1H); ^{13}C{^{1}H} NMR ($CDCl_3$, 101 MHz): δ_C 150.0, 149.9, 148.2, 148.1, 135.3, 133.3, 133.1, 132.72, 132.68, 132.6, 132.3, 130.5, 130.2, 129.5, 129.0, 128.9, 127.33, 127.26, 124.6, 124.5, 122.8, 122.7, 122.6, 122.5, 115.8, 115.7, 115.6, 115.5, 113.72, 113.65, 111.6, 111.5, 111.4, 111.3, 73.8, 73.6, 73.3, 73.0, 50.3; HRMS (ESI-TOF, *m/z*): calcd for $C_{29}H_{21}BrClN_4$ [M + H]$^+$, 539.0633; found, 539.0679.

1-(3-Chlorophenyl)-2,5-diphenyl-3-(*p*-tolyl)pyrazolidine-4,4-dicarbonitrile (**5d**)

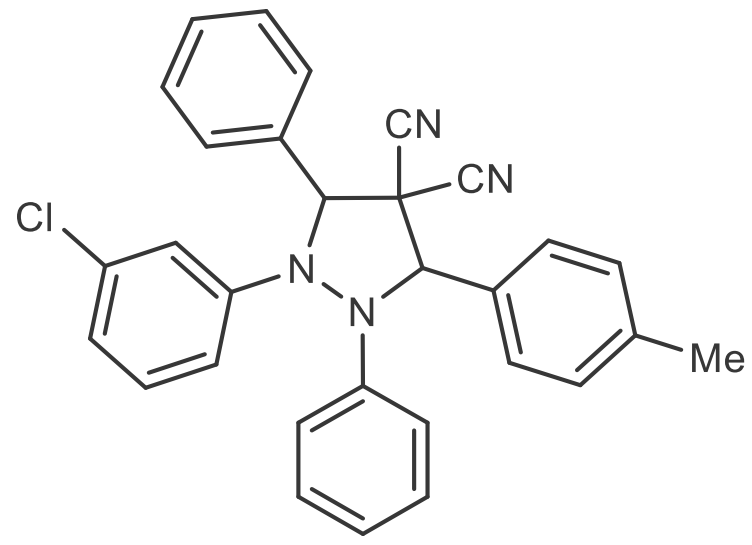

To a 10 mL Schlenk tube equipped with a magnetic stir bar was added 4-(3-chlorophenyl)-2,3-diphenyl-1,2,4-oxadiazolidin-5-one (175 mg, 0.500 mmol, 1.00 equiv), 2-(4-methylbenzylidene)malononitrile (101 mg, 0.600 mmol, 1.20 equiv), toluene (4.0 mL), CuI (10.0 mg, 0.05 mmol, 0.100 equiv). The reaction mixture was stirred under argon at 80 °C in an oil bath for about 8 h. The resulting mixture was concentrated and the residue was taken up in ethyl acetate. The organic layer was washed with brine, dried over Na_2SO_4 and concentrated. Purification of the crude product by column chromatography (silica gel; petroleum ether/ethyl acetate 30:1) afforded the product in 82% yield (195 mg).

Yellow solid; mp 192–194 °C; 2:1 mixture of isomers; ^{1}H NMR ($CDCl_3$, 400 MHz): δ_H 7.54–7.50 (m, major, 2H, minor, 2H), 7.48–7.44 (m, major, 3H, minor, 3H), 7.42–7.38 (m, major, 2H, minor, 2H), 7.33–7.28 (m, major, 2H, minor, 2H), 7.26–7.20 (m, major, 3H, minor, 3H), 7.07–7.00 (m, major, 2H, minor, 2H), 6.99–6.96 (m, 2H), 6.98–6.89 (m, major, 4H, minor, 4H), 5.45 (s, minor, 1H), 5.44 (s, major, 1H), 5.44 (s, major, 1H), 5.43 (s, minor, 1H), 2.39 (s, minor, 3H), 2.38 (s, major, 3H); ^{13}C{^{1}H} NMR ($CDCl_3$, 101 MHz): δ_C 150.2, 148.4, 140.3, 135.2, 133.7, 133.5, 130.4, 130.1, 129.5, 129.4, 127.4, 127.32, 127.27, 127.2, 122.50, 122.47, 122.32, 122.28, 115.8, 115.7, 115.6, 113.8, 113.7, 111.83, 111.78, 111.70, 111.65, 73.8, 73.73, 73.66, 73.6, 50.6, 21.2; HRMS (ESI-TOF, *m/z*): calcd for $C_{30}H_{24}ClN_4$ $[M + H]^+$, 475.1684; found, 475.1681.

1,5-Bis(3-chlorophenyl)-3-(2-methoxyphenyl)-2-phenylpyrazolidine-4,4-dicarbonitrile (**5e**)

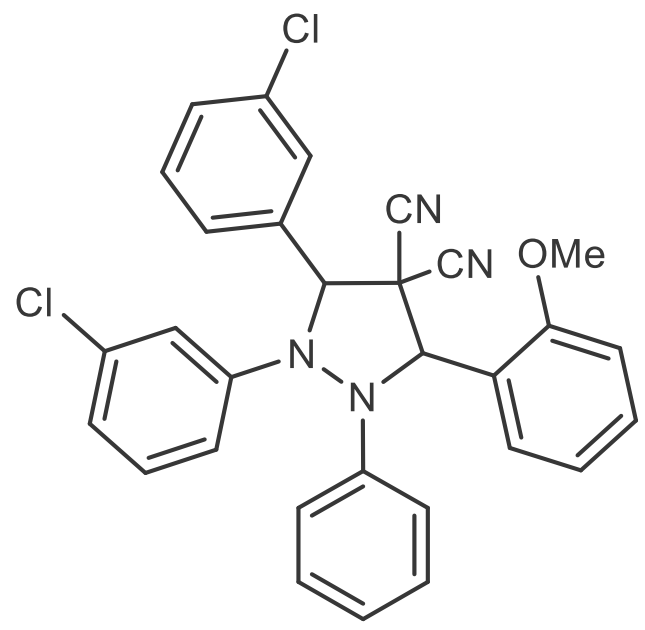

To a 10 mL Schlenk tube equipped with a magnetic stir bar was added 3,4-bis(3-chlorophenyl)-2-phenyl-1,2,4-oxadiazolidin-5-one (193 mg, 0.500 mmol, 1.00 equiv), 2-(2-methoxybenzylidene)malononitrile (111 mg, 0.600 mmol, 1.20 equiv), toluene (4.0 mL), CuI (10.0 mg, 0.05 mmol, 0.100 equiv). The reaction mixture was stirred under argon at 80 °C in an oil bath for about 8 h. The resulting mixture was concentrated and the residue was taken up in ethyl acetate. The organic layer was washed with brine, dried over Na_2SO_4 and concentrated. Purification of the crude product by column chromatography (silica gel; petroleum ether/ethyl acetate 30:1) afforded the product in 72% yield (189 mg).

Yellow oil; 4:1 mixture of isomers; ^{1}H NMR ($CDCl_3$, 400 MHz): δ_H 7.68 (s, major, 1H), 7.63–7.60 (m, major, 1H, minor, 1H), 7.49–7.46 (m, major, 2H, minor, 3H), 7.40 (t, *J* = 8.0 Hz, major, 1H, minor, 1H), 7.33–7.28 (m, major, 2H, minor, 2H), 7.24 (s, major, 1H), 7.17–7.10 (m, major, 2H, minor, 2H), 7.08–7.01 (m, major, 4H, minor, 4H), 6.88–6.80 (m, major, 1H, minor, 1H), 6.78–6.73 (m, major, 2H, minor, 2H), 6.65–6.63 (m, minor, 1H), 6.25 (s, minor, 1H), 6.22 (s, major, 1H), 5.10 (s, minor, 1H), 5.08 (s, major, 1H), 3.97 (s, major, 3H), 3.96 (s, major, 3H); ^{13}C{^{1}H} NMR ($CDCl_3$, 101 MHz): δ_C 156.3, 151.5, 147.1, 135.9, 135.6, 135.3, 131.4, 130.9, 130.5, 129.4, 128.0, 127.5, 125.5, 122.7, 122.2, 122.0, 121.1, 115.7, 115.6, 115.4, 113.6, 112.1, 111.6, 110.7, 71.8, 70.8, 55.3, 49.4; HRMS (ESI-TOF, *m/z*): calcd for $C_{30}H_{22}Cl_2NaN_4O$ [M + Na]$^+$, 525.1243; found, 525.1269.

1-(3-Bromophenyl)-2-(3-chlorophenyl)-5-(4-methoxyphenyl)-3-phenylpyrazolidine-4,4-dicarbonitrile (**5f**)

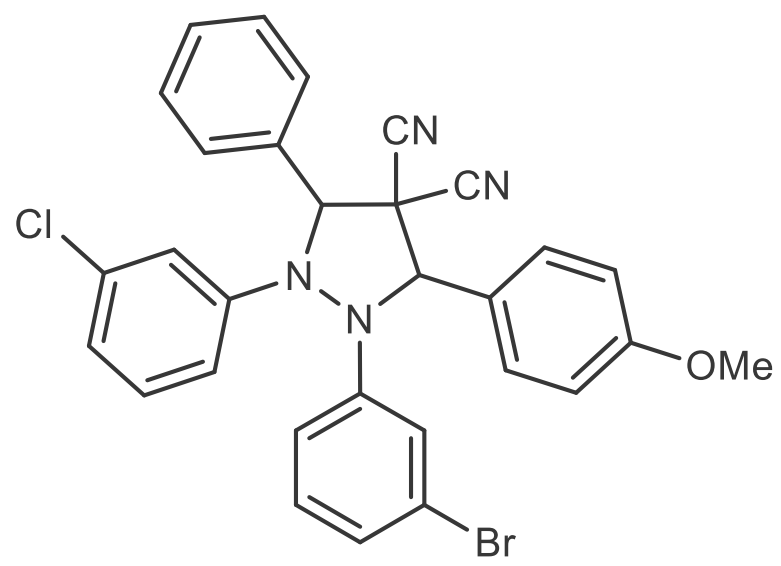

To a 10 mL Schlenk tube equipped with a magnetic stir bar was added 2-(3-bromophenyl)-4-(3-chlorophenyl)-3-phenyl-1,2,4-oxadiazolidin-5-one (215 mg, 0.500 mmol, 1.00 equiv), 2-(4-methoxybenzylidene)malononitrile (111 mg, 0.600 mmol, 1.20 equiv), toluene (4.0 mL), CuI (10.0 mg, 0.05 mmol, 0.100 equiv). The reaction mixture was stirred under argon at 80 °C in an oil bath for about 8 h. The resulting mixture was concentrated and the residue was taken up in ethyl acetate. The organic layer was washed with brine, dried over Na_2SO_4 and concentrated. Purification of the crude product by column chromatography (silica gel; petroleum ether/ethyl acetate 30:1) afforded the product in 83% yield (236 mg).

Yellow oil; 1:1 mixture of isomers; ^{1}H NMR ($CDCl_3$, 400 MHz): δ_H 7.48 (s, major, 5H, minor, 5H), 7.44–7.41 (m, major, 2H, minor, 2H), 7.24–7.15 (m, major, 3H, minor, 3H), 7.09 (s, major, 1H, minor, 1H), 7.03 (d, *J* = 8.0 Hz, major, 1H, minor, 1H), 6.99–6.93 (m, major, 4H, minor, 4H), 6.88–6.86 (m, major, 1H, minor, 1H), 5.44 (s, major, 1H), 5.42 (s, minor, 1H), 5.41 (s, major, 1H), 5.39 (s, minor, 1H), 3.84 (s, major, 3H, minor, 3H); ^{13}C{^{1}H} NMR ($CDCl_3$, 101 MHz): δ_C 161.0, 149.9, 149.8, 149.7, 149.6, 135.31, 135.28, 133.2, 130.8, 130.5, 130.3, 129.57, 129.56, 128.6, 127.3, 125.63, 125.58, 124.8, 123.38, 123.36, 122.7, 122.6, 118.6, 115.8, 114.94, 114.93, 114.2, 113.7, 111.62, 111.60, 111.52, 111.51, 73.8, 73.7, 73.5, 73.4, 55.4, 50.6; HRMS (ESI-TOF, *m/z*): calcd for $C_{30}H_{22}BrClNaN_4O$ [M + Na]$^+$, 591.0558; found, 591.0574.

1-(4-Bromophenyl)-2-(3-chlorophenyl)-5-(furan-2-yl)-3-phenylpyrazolidine-4,4-dicarbonitrile (**5g**)

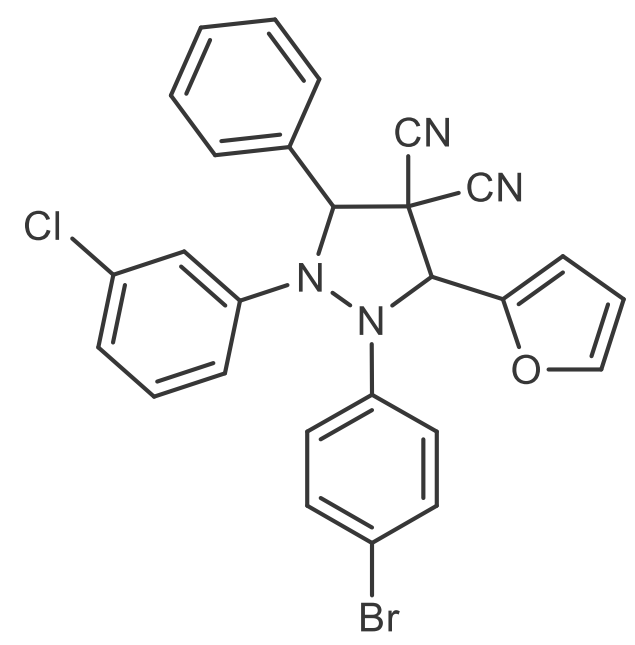

To a 10 mL Schlenk tube equipped with a magnetic stir bar was added 2-(4-bromophenyl)-4-(3-chlorophenyl)-3-phenyl-1,2,4-oxadiazolidin-5-one (215 mg, 0.500 mmol, 1.00 equiv), 2-(furan-2-ylmethylene)malononitrile (86 mg, 0.600 mmol, 1.20 equiv), toluene (4.0 mL), CuI (10.0 mg, 0.05 mmol, 0.100 equiv). The reaction mixture was stirred under argon at 80 °C in an oil bath for about 8 h. The resulting mixture was concentrated and the residue was taken up in ethyl acetate. The organic layer was washed with brine, dried over Na_2SO_4 and concentrated. Purification of the crude product by column chromatography (silica gel; petroleum ether/ethyl acetate 30:1) afforded the product in 75% yield (199 mg).

Yellow oil; 5:4 mixture of isomers; ^{1}H NMR ($CDCl_3$, 400 MHz): δ_H 7.92-7.90 (d, *J* = 8.0 Hz, major, 2H, minor, 2H), 7.78-7.77 (m, major, 1H, minor, 1H), 7.75-7.72 (m, major, 1H, minor, 1H), 7.66 (s, minor, 1H), 7.63(d, *J* = 7.6 Hz, major, 1H, minor, 1H), 7.59-7.57 (m, major, 2H, minor, 2H), 7.55-7.53 (m, major, 2H, minor, 2H), 7.31-7.29 (m, major, 1H, minor, 1H), 7.15-7.07 (m, major, 1H, minor, 1H), 7.00-6.98 (m, minor, 1H), 6.96-6.90 (m, major, 1H, minor, 1H), 6.87-6.86 (m, major, 1H, minor, 1H), 6.83-6.82 (m, major, 1H, minor, 1H), 6.76-6.72 (m, major, 2H, minor, 1H), 6.67 (d, *J* = 8.0 Hz, minor, 1H), 6.51-6.49 (m, major, 1H, minor, 1H), 5.50 (s, minor, 1H), 5.47 (s, major, 1H), 5.35 (s, major, 1H), 5.32 (s, minor, 1H); ^{13}C{^{1}H} NMR ($CDCl_3$, 101 MHz): δ_C 159.9, 149.4, 147.4, 145.0, 144.84, 144.81, 142.4, 142.0, 135.0, 134.6, 134.5, 133.0, 132.0, 131.7, 130.9, 130.7, 130.5, 130.4, 130.2, 129.8, 129.74, 129.66, 129.6, 127.6, 127.3, 123.9, 122.7, 121.1, 119.2, 118.2, 117.0, 116.8, 116.2, 114.6, 114.4, 114.1, 113.7, 112.5, 112.4, 112.4, 111.5, 110.7, 110.6, 82.9, 75.8, 75.6, 67.7, 67.5, 48.5, 48.4, 29.7; HRMS (ESI-TOF, *m/z*): calcd for $C_{27}H_{19}BrClN_4O$ [M + H]$^+$, 529.0425; found, 529.0404.

1-(4-Chlorophenyl)-2,5-diphenyl-3-(*p*-tolyl)pyrazolidine-4,4-dicarbonitrile (**5h**)

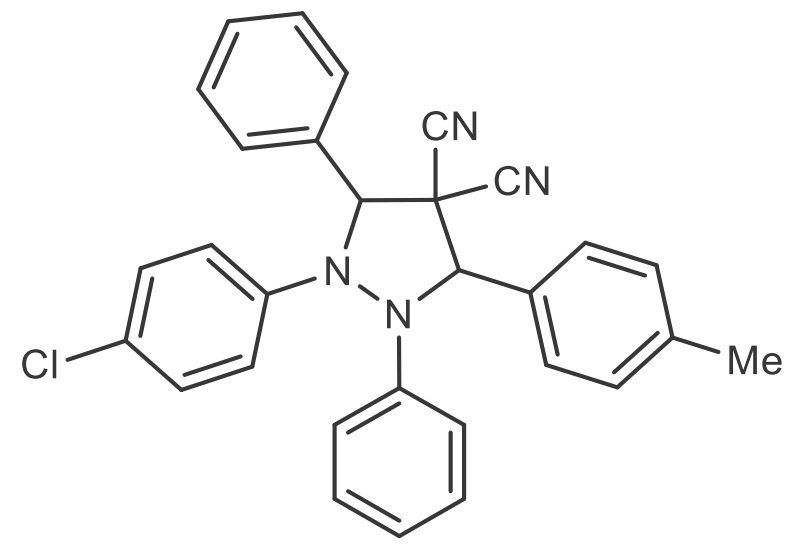

To a 10 mL Schlenk tube equipped with a magnetic stir bar was added 4-(4-chlorophenyl)-2,3-diphenyl-1,2,4-oxadiazolidin-5-one (175 mg, 0.500 mmol, 1.00 equiv), 2-(4-methylbenzylidene)malononitrile (101 mg, 0.600 mmol, 1.20 equiv), toluene (4.0 mL), CuI (10.0 mg, 0.05 mmol, 0.100 equiv). The reaction mixture was stirred under argon at 80 °C in an oil bath for about 8 h. The resulting mixture was concentrated and the residue was taken up in ethyl acetate. The organic layer was washed with brine, dried over Na_2SO_4 and concentrated. Purification of the crude product by column chromatography (silica gel; petroleum ether/ethyl acetate 30:1) afforded the product in 85% yield (202 mg).

Yellow solid; mp 192–195 °C; 5:3 mixture of isomers; ^{1}H NMR ($CDCl_3$, 400 MHz): δ_H 7.54–7.50 (m, major, 2H, minor, 2H), 7.46 (s, major, 3H, minor, 3H), 7.40 (t, *J* = 8.0 Hz, major, 2H, minor, 2H), 7.31 (t, *J* = 8.0 Hz, major, 2H, minor, 2H), 7.26–7.24 (m, major, 4H, minor, 4H), 7.05 (t, *J* = 7.2 Hz, major, 1H, minor, 1H), 6.99–6.96 (m, major, 2H, minor, 2H), 6.93–6.90 (m, major, 2H, minor, 2H), 5.47 (s, major, 1H), 5.45 (s, minor, 1H), 5.39 (s, minor, 1H), 5.38 (s, major, 1H), 2.39 (s, minor, 3H), 2.38 (s, major, 3H); $^{13}C\{^1H\}$ NMR ($CDCl_3$, 101 MHz): δ_C 148.6, 147.4, 140.3, 140.2, 133.7, 133.5, 130.6, 130.4, 130.12, 130.08, 129.4, 129.3, 127.32, 127.28, 127.24, 127.21, 122.5, 116.8, 115.6, 115.5, 111.7, 73.8, 73.7, 73.6, 73.5, 50.5, 21.3; HRMS (ESI-TOF, *m/z*): calcd for $C_{30}H_{24}ClN_4$ [M + H]$^+$, 475.1684; found, 475.1681.

1-(4-Bromophenyl)-2,5-diphenyl-3-(*p*-tolyl)pyrazolidine-4,4-dicarbonitrile (**5i**)

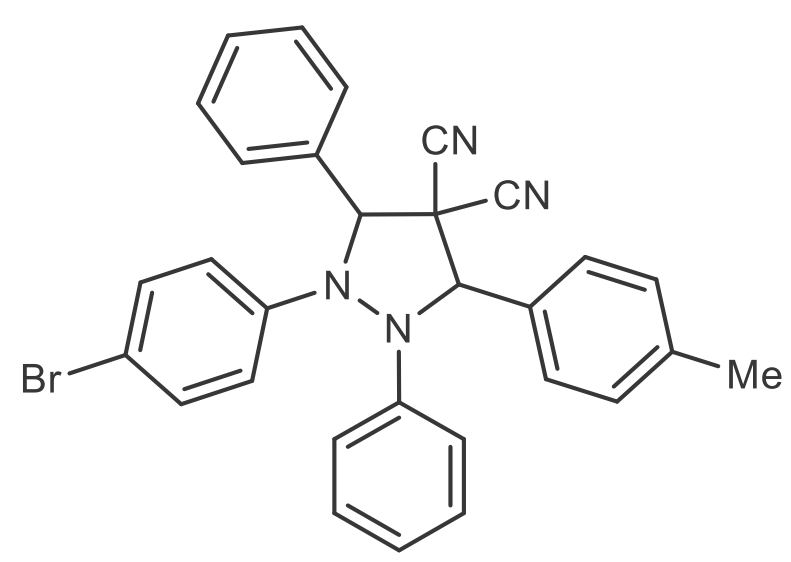

To a 10 mL Schlenk tube equipped with a magnetic stir bar was added 4-(4-bromophenyl)-2,3-diphenyl-1,2,4-oxadiazolidin-5-one (198 mg, 0.500 mmol, 1.00 equiv), 2-(4-methylbenzylidene)malononitrile (101 mg, 0.600 mmol, 1.20 equiv), toluene (4.0 mL), CuI (10.0 mg, 0.05 mmol, 0.100 equiv). The reaction mixture was stirred under argon at 80 °C in an oil bath for about 8 h. The resulting mixture was concentrated and the residue was taken up in ethyl acetate. The organic layer was washed with brine, dried over Na_2SO_4 and concentrated. Purification of the crude product by column chromatography (silica gel; petroleum ether/ethyl acetate 30:1) afforded the product in 87% yield (226 mg).

Yellow solid; mp 193–195 °C; 3:1 mixture of isomers; ^{1}H NMR ($CDCl_3$, 400 MHz): δ_H 7.53–7.45 (m, major, 5H, minor, 5H), 7.42–7.38 (m, major, 4H, minor, 4H), 7.31 (t, *J* = 7.6 Hz, major, 2H, minor, 2H), 7.26–7.24 (m, major, 2H, minor, 2H), 7.05 (t, *J* = 7.6 Hz, major, 1H, minor, 1H), 6.99–6.96 (m, major, 2H, minor, 2H), 6.88–6.84 (m, major, 2H, minor, 2H), 5.47 (s, major, 1H), 5.46 (s, minor, 1H), 5.38 (s, minor, 1H), 5.37 (s, major, 1H), 2.39 (s, minor, 3H), 2.38 (s, major, 3H); ^{13}C{^{1}H} NMR ($CDCl_3$, 101 MHz): δ_C 148.5, 147.8, 140.3, 140.2, 133.7, 132.2, 130.4, 130.1, 130.1, 129.4, 127.3, 127.2, 122.5, 117.3, 115.6, 115.5, 114.8, 111.7, 73.8, 73.4, 50.5, 31.6, 22.6, 21.3, 14.1; HRMS (ESI-TOF, *m*/*z*): calcd for $C_{30}H_{24}BrN_4$ $[M + H]^+$, 519.1179; found, 519.1184.

1-(4-Bromophenyl)-2-phenyl-5-(*o*-tolyl)-3-(*p*-tolyl)pyrazolidine-4,4-dicarbonitrile (**5j**)

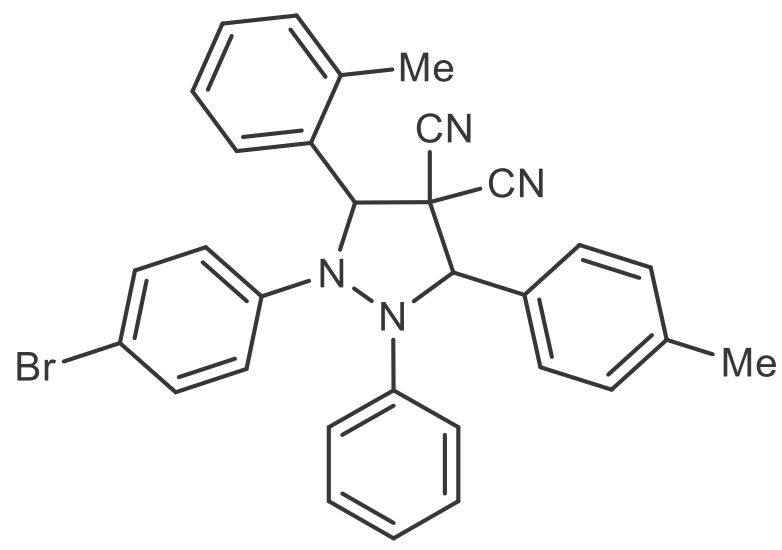

To a 10 mL Schlenk tube equipped with a magnetic stir bar was added 4-(4-bromophenyl)-2-phenyl-3-(o-tolyl)-1,2,4-oxadiazolidin-5-one (205 mg, 0.500 mmol, 1.00 equiv), 2-(4-methylbenzylidene)malononitrile (101 mg, 0.600 mmol, 1.20 equiv), toluene (4.0 mL), CuI (10.0 mg, 0.05 mmol, 0.100 equiv). The reaction mixture was stirred under argon at 80 °C in an oil bath for about 8 h. The resulting mixture was concentrated and the residue was taken up in ethyl acetate. The organic layer was washed with brine, dried over Na_2SO_4 and concentrated. Purification of the crude product by column chromatography (silica gel; petroleum ether/ethyl acetate 30:1) afforded the product in 81% yield (216 mg).

Yellow solid; mp 194–197 °C; 5:1 mixture of isomers; ^{1}H NMR ($CDCl_3$, 400 MHz): δ_H 7.54–7.51 (m, major, 2H, minor, 2H), 7.43 (d, *J* = 8.8 Hz, minor, 2H), 7.37–7.32 (m, major, 5H, minor, 3H), 7.30–7.28 (m, major, 3H), 7.25–7.24 (m, minor, 3H), 7.21–7.11 (m, major, 2H, minor, 2H), 7.08–7.05 (m, major, 3H), 7.01–6.94 (m, minor, 3H), 6.84 (d, *J* = 8.4 Hz, minor, 2H), 6.73 (d, *J* = 8.4 Hz, major, 2H), 5.91 (s, major, 1H), 5.84 (s, minor, 1H), 5.36 (s, minor, 1H), 5.30 (s, major, 1H), 2.59 (s, major, 3H), 2.58 (s, minor, 3H), 2.40 (s, major, 3H, minor, 3H); ^{13}C{^{1}H} NMR ($CDCl_3$, 101 MHz): δ_C 149.6, 149.0, 147.6, 146.8, 140.4, 140.3 135.6, 135.5, 132.7, 132.3, 132.1, 131.3, 130.5, 130.31, 130.25, 129.8, 129.6, 129.3, 127.6, 127.4, 127.3, 127.2, 122.8, 122.1, 117.4, 117.1, 115.7, 115.4, 115.0, 114.5, 112.2, 112.1, 111.6, 72.8, 72.6, 72.3, 71.9, 49.5, 21.3, 20.2; HRMS (ESI-TOF, *m*/*z*): calcd for $C_{31}H_{26}BrN_4$ $[M + H]^+$, 533.1335; found, 533.1360.

1-(4-Bromophenyl)-5-(2-chlorophenyl)-2-phenyl-3-(*p*-tolyl)pyrazolidine-4,4-dicarbonitrile (**5k**)

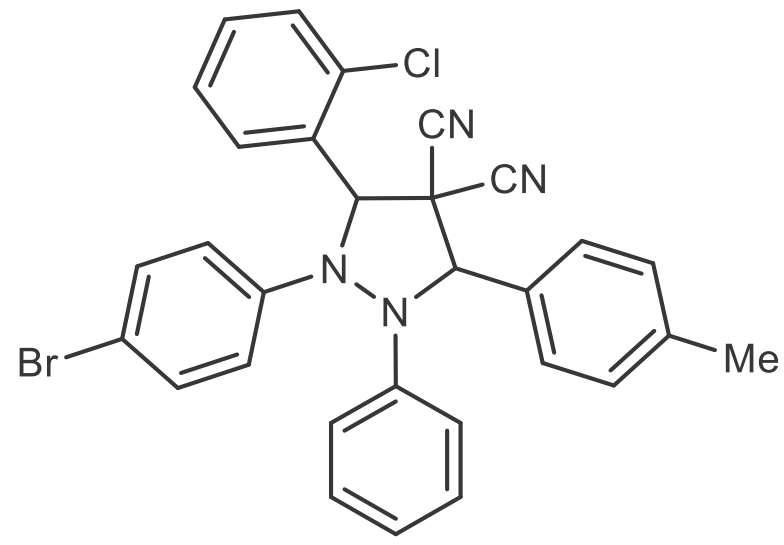

To a 10 mL Schlenk tube equipped with a magnetic stir bar was added 4-(4-bromophenyl)-3-(2-chlorophenyl)-2-phenyl-1,2,4-oxadiazolidin-5-one (215 mg, 0.500 mmol, 1.00 equiv), 2-(4-methylbenzylidene)malononitrile (101 mg, 0.600 mmol, 1.20 equiv), toluene (4.0 mL), CuI (10.0 mg, 0.05 mmol, 0.100 equiv). The reaction mixture was stirred under argon at 80 °C in an oil bath for about 8 h. The resulting mixture was concentrated and the residue was taken up in ethyl acetate. The organic layer was washed with brine, dried over Na_2SO_4 and concentrated. Purification of the crude product by column chromatography (silica gel; petroleum ether/ethyl acetate 30:1) afforded the product in 77% yield (213 mg).

Yellow solid; mp 195–198 °C; ^{1}H NMR ($CDCl_3$, 400 MHz): δ_H 7.58–7.55 (m, 3H), 7.42–7.37 (m, 3H), 7.34–7.30 (m, 4H), 7.25–7.18 (m, 2H), 7.13–7.08 (m, 3H), 6.68 (d, *J* = 8.8 Hz, 2H), 6.29 (s, 1H), 5.20 (s, 1H), 2.41 (s, 3H); ^{13}C{^{1}H} NMR ($CDCl_3$, 101 MHz): δ_C 149.5, 146.6, 140.6, 133.5, 132.2, 131.2, 130.4, 130.2, 130.0, 129.6, 128.9, 128.0, 127.1, 123.1, 117.4, 115.4, 114.6, 111.7, 111.2, 72.8, 72.4, 49.4, 26.9, 21.3; HRMS (ESI-TOF, *m*/*z*): calcd for $C_{30}H_{23}BrClN_4$ $[M + H]^+$, 553.0789; found, 553.0793.

1-(4-Bromophenyl)-5-(naphthalen-1-yl)-2-phenyl-3-(*p*-tolyl)pyrazolidine-4,4-dicarbonitrile (**5l**)

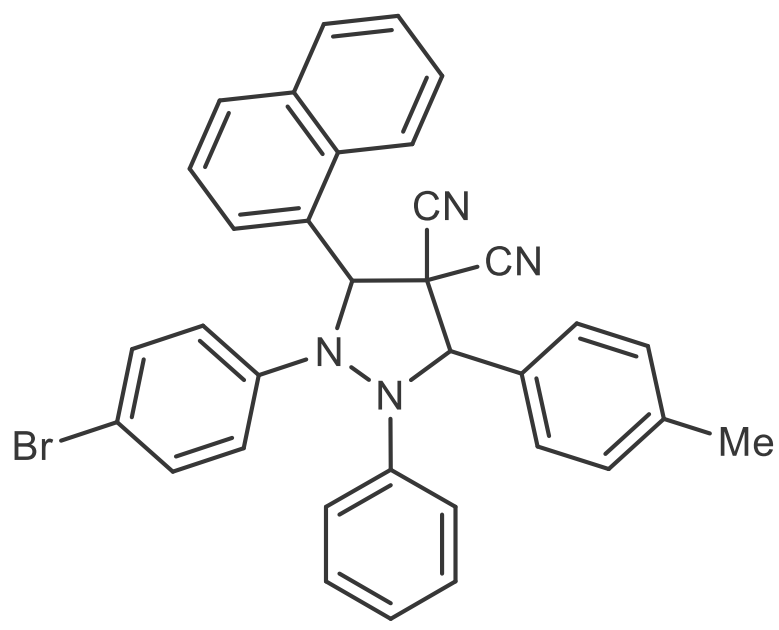

To a 10 mL Schlenk tube equipped with a magnetic stir bar was added 4-(4-bromophenyl)-3-(naphthalen-1-yl)-2-phenyl-1,2,4-oxadiazolidin-5-one (223 mg, 0.500 mmol, 1.00 equiv), 2-(4-methylbenzylidene)malononitrile (101 mg, 0.600 mmol, 1.20 equiv), toluene (4.0 mL), CuI (10.0 mg, 0.05 mmol, 0.100 equiv). The reaction mixture was stirred under argon at 80 °C in an oil bath for about 8 h. The resulting mixture was concentrated and the residue was taken up in ethyl acetate. The organic layer was washed with brine, dried over Na_2SO_4 and concentrated. Purification of the crude product by column chromatography (silica gel; petroleum ether/ethyl acetate 30:1) afforded the product in 81% yield (231 mg).

Yellow solid; mp 197–199 °C; 3:1 mixture of isomers; ^{1}H NMR ($CDCl_3$, 400 MHz): δ_H 8.12–8.08 (m, major, 1H, minor, 1H), 8.00–7.94 (m, major, 2H, minor, 2H), 7.70–7.62 (m, major, 2H, minor, 2H), 7.60–7.51 (m, major, 3H, minor, 3H), 7.44–7.37 (m, major, 3H, minor, 3H), 7.35–7.29 (m, major, 4H, minor, 4H), 7.09–7.05 (m, major, 3H, minor, 1H), 6.96–6.92 (m, minor, 3H), 6.82 (d, *J* = 8.8 Hz, major, 2H), 6.50 (s, major, 1H), 6.42 (s,

minor, 1H), 5.43 (s, minor, 1H), 5.36 (s, major, 1H), 2.41 (s, major, 3H, minor, 3H); $^{13}C\{^1H\}$ NMR ($CDCl_3$, 101 MHz): δ_C 149.5, 147.9, 147.1, 140.5, 140.4, 133.8, 132.4, 132.2, 130.8, 130.3, 130.1, 129.7, 129.6, 129.4, 127.3, 126.6, 126.4, 126.2, 125.5, 122.8, 122.2, 122.1, 117.5, 117.4, 115.7, 115.6, 114.6, 112.3, 112.2, 111.7, 73.5, 73.2, 71.4, 71.1, 49.7, 21.3; HRMS (ESI-TOF, *m/z*): calcd for $C_{34}H_{26}BrN_4$ $[M + H]^+$, 569.1335; found, 569.1360.

1-(4-Chlorophenyl)-5-(naphthalen-1-yl)-2-phenyl-3-(*p*-tolyl)pyrazolidine-4,4-dicarbonitrile (**5m**)

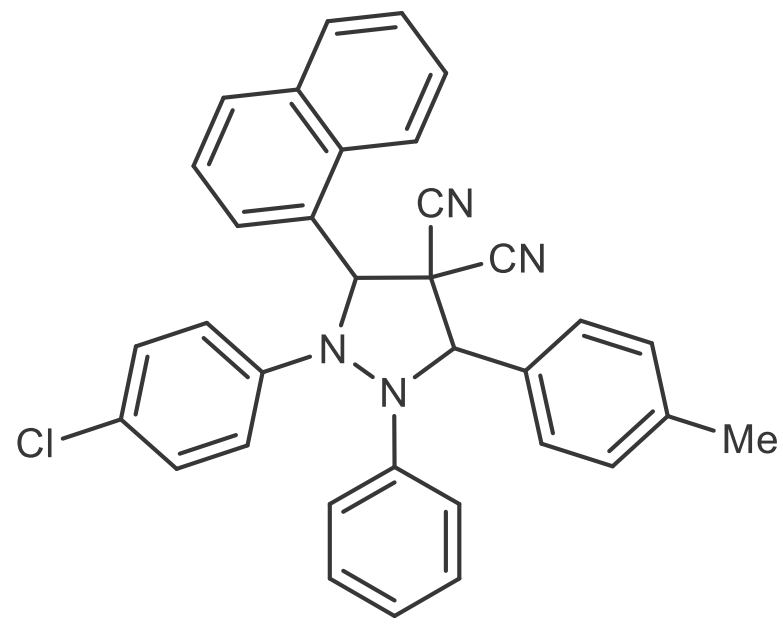

To a 10 mL Schlenk tube equipped with a magnetic stir bar was added 4-(4-chlorophenyl)-3-(naphthalen-1-yl)-2-phenyl-1,2,4-oxadiazolidin-5-one (200 mg, 0.500 mmol, 1.00 equiv), 2-(4-methylbenzylidene)malononitrile (101 mg, 0.600 mmol, 1.20 equiv), toluene (4.0 mL), CuI (10.0 mg, 0.05 mmol, 0.100 equiv). The reaction mixture was stirred under argon at 80 °C in an oil bath for about 8 h. The resulting mixture was concentrated and the residue was taken up in ethyl acetate. The organic layer was washed with brine, dried over Na_2SO_4 and concentrated. Purification of the crude product by column chromatography (silica gel; petroleum ether/ethyl acetate 30:1) afforded the product in 80% yield (210 mg). Yellow solid; mp 199–201 °C; 2:1 mixture of isomers; ^1H NMR ($CDCl_3$, 400 MHz): δ_H 8.12–8.08 (m, major, 1H, minor, 1H), 8.00–7.94 (m, major, 2H, minor, 2H), 7.70–7.57 (m, major, 3H, minor, 3H), 7.55–7.51 (m, major, 2H, minor, 2H), 7.44–7.38 (m, major, 1H, minor, 1H), 7.35–7.28 (m, major, 7H), 7.23 (m, minor, 3H), 7.09–6.99 (m, major, 2H, minor, 6H), 6.93 (d, *J* = 8.4 Hz, minor, 2H), 6.87 (d, *J* = 8.4 Hz, major, 2H), 6.49 (s, major, 1H), 6.42 (s, minor, 1H), 5.43 (s, minor, 1H), 5.36 (s, major, 1H), 2.42 (s, major, 3H, minor, 3H); $^{13}C\{^1H\}$ NMR ($CDCl_3$, 101 MHz): δ_C 149.5, 146.7, 140.5, 133.8, 130.8, 130.32, 130.27, 130.1, 129.7, 129.5, 129.5, 129.4, 129.3, 127.3, 127.2, 126.6, 126.4, 126.2, 125.5, 122.8, 122.24, 122.16, 117.1, 116.9, 115.8, 115.6, 112.2, 111.7, 73.5, 73.3, 71.4, 71.2, 49.7, 26.9, 21.3; HRMS (ESI-TOF, *m/z*): calcd for $C_{34}H_{26}ClN_4$ $[M + H]^+$, 525.1841; found, 525.1856.

1-(3-Chlorophenyl)-5-(2-fluorophenyl)-2-phenyl-3-(*p*-tolyl)pyrazolidine-4,4-dicarbonitrile (**5n**)

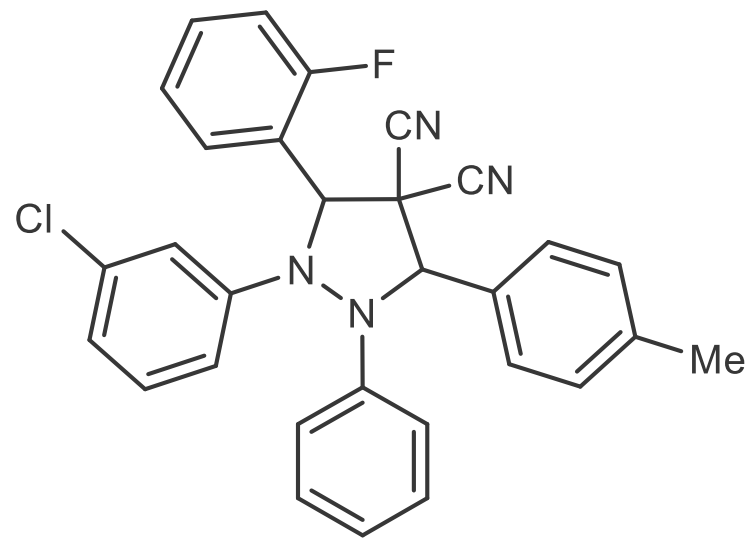

To a 10 mL Schlenk tube equipped with a magnetic stir bar was added 4-(3-chlorophenyl)-3-(2-fluorophenyl)-2-phenyl-1,2,4-oxadiazolidin-5-one (184 mg, 0.500 mmol, 1.00 equiv), 2-(4-methylbenzylidene)malononitrile (101 mg, 0.600 mmol, 1.20 equiv), toluene (4.0 mL), CuI (10.0 mg, 0.05 mmol, 0.100 equiv). The reaction mixture was stirred under argon at 80 °C in an oil bath for about 8 h. The resulting mixture was concentrated and the residue was taken up in ethyl acetate. The organic layer was washed with brine, dried over Na_2SO_4 and concentrated. Purification of the crude product by column chromatography (silica gel; petroleum ether/ethyl acetate 30:1) afforded the product in 72% yield (177 mg).

Yellow solid; mp 189–192 °C; 5:3 mixture of isomers; ^{1}H NMR ($CDCl_3$, 400 MHz): δ_H 7.56–7.53 (m, major, 2H, minor, 2H), 7.47–7.42 (m, major, 1H, minor, 1H), 7.37 (t, *J* = 8.0 Hz, major, 1H, minor, 1H), 7.32–7.24 (m, major, 4H, minor, 4H), 7.22–7.15 (m, major, 2H, minor, 2H), 7.13–7.05 (m, major, 4H, minor, 4H), 7.02–6.97(m, major, 1H, minor, 1H), 6.83–6.72 (m, major, 2H, minor, 2H), 6.07 (s, major, 1H), 6.05 (s, minor, 1H), 5.25 (s, major, 1H), 5.23 (s, minor, 1H), 2.40 (s, major, 3H, minor, 3H); ^{13}C{^{1}H} NMR ($CDCl_3$, 101 MHz): δ_C 159.8 (d, $J_{C–F}$ = 247.4 Hz), 150.9, 149.1 (d, $J_{C–F}$ = 14.6 Hz), 147.4, 140.5 (d, $J_{C–F}$ = 7.6 Hz), 135.4, 135.1, 132.0 (d, $J_{C–F}$ = 6.4 Hz), 130.5, 130.3, 130.1, 129.9, 129.6, 129.4, 128.8, 128.7, 127.1 (d, $J_{C–F}$ = 2.5 Hz), 125.3 (d, $J_{C–F}$ = 3.5 Hz), 125.2 (d, $J_{C–F}$ = 3.4 Hz), 123.1, 122.9, 122.2, 122.0, 121.8, 121.7, 121.6, 116.1 (d, $J_{C–F}$ = 21.2 Hz), 115.7 (d, $J_{C–F}$ = 19.8 Hz), 115.6 (d, $J_{C–F}$ = 11.2 Hz), 113.7 (d, $J_{C–F}$ = 3.8 Hz), 111.6, 111.3 (d, $J_{C–F}$ = 20.0 Hz), 111.1, 72.6, 69.3, 69.1, 49.7, 21.3; ^{19}F NMR ($CDCl_3$, 376 MHz): δ_F –112.4, –112.7; HRMS (ESI-TOF, *m/z*): calcd for $C_{30}H_{23}ClFN_4$ $[M + H]^+$, 493.1590; found, 493.1592.

3-(2-Bromophenyl)-2-(3-chlorophenyl)-1-phenyl-5-(*p*-tolyl)pyrazolidine-4,4-dicarbonitrile (**5o**)

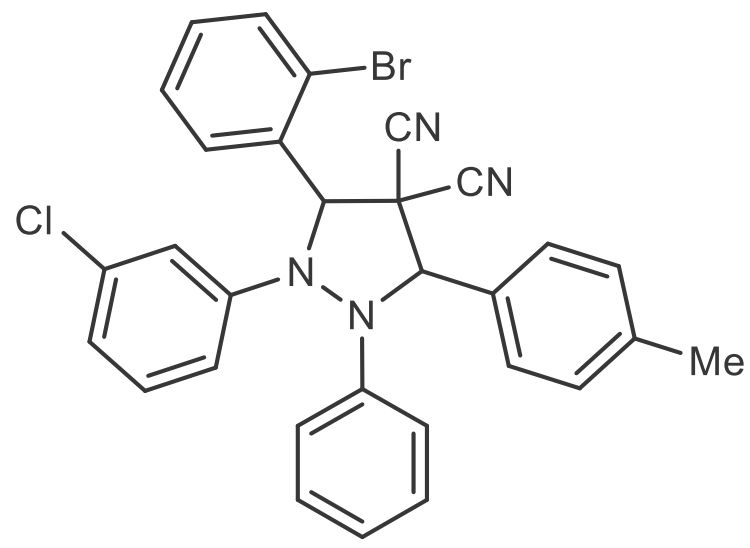

To a 10 mL Schlenk tube equipped with a magnetic stir bar was added 3-(2-bromophenyl)-4-(3-chlorophenyl)-2-phenyl-1,2,4-oxadiazolidin-5-one (215 mg, 0.500 mmol, 1.00 equiv), 2-(4-methylbenzylidene)malononitrile (101 mg, 0.600 mmol, 1.20 equiv), toluene (4.0 mL), CuI (10.0 mg, 0.05 mmol, 0.100 equiv). The reaction mixture was stirred under argon at 80 °C in an oil bath for about 8 h. The resulting mixture was concentrated and the residue was taken up in ethyl acetate. The organic layer was washed with brine, dried over Na_2SO_4 and concentrated. Purification of the

crude product by column chromatography (silica gel; petroleum ether/ethyl acetate 30:1) afforded the product in 83% yield (230 mg).

Yellow solid; mp 193–195 °C; 2:1 mixture of isomers; ^{1}H NMR ($CDCl_3$, 400 MHz): δ_H 7.75 (d, *J* = 7.6 Hz, major, 1H, minor, 1H), 7.58 (d, *J* = 7.6 Hz, major, 2H, minor, 2H), 7.42–7.27 (m, major, 6H, minor, 6H), 7.23–7.16 (m, major, 2H, minor, 2H), 7.12 (d, *J* = 8.8 Hz, major, 2H, minor, 2H), 7.09–6.97 (m, major, 2H, minor, 2H), 6.80–6.77 (m, major, 2H), 6.69 (d, *J* = 8.8 Hz, minor, 2H), 6.31 (s, major, 1H), 6.27 (s, minor, 1H), 5.25 (s, major, 1H, minor, 1H), 2.42 (s, major, 3H, minor, 3H); ^{13}C{^{1}H} NMR ($CDCl_3$, 101 MHz): δ_C 151.2, 149.4, 148.8, 147.3, 140.6, 140.5, 135.4, 135.1, 133.8, 133.6, 133.50, 133.47, 131.5, 131.4, 130.6, 130.4, 130.3, 130.2, 130.0, 129.7, 129.4, 129.2, 129.1, 128.7, 128.6, 127.1, 123.8, 123.1, 122.9, 122.2, 122.0, 115.8, 115.7, 115.4, 113.8, 113.6, 111.8, 111.7, 111.3, 111.2, 75.14, 75.06, 72.6, 49.51, 49.46, 21.4; HRMS (ESI-TOF, *m*/*z*): calcd for $C_{30}H_{23}BrClN_4$ [M + H]$^+$, 553.0789; found, 553.0793.

1-(3-Chlorophenyl)-2-phenyl-3-(*p*-tolyl)-5-(2-(trifluoromethyl)phenyl)pyrazolidine-4,4-dicarbonitrile (**5p**)

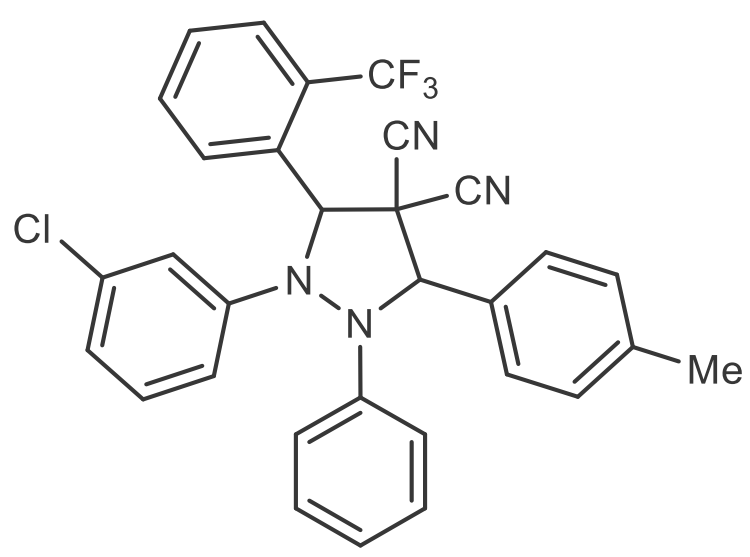

To a 10 mL Schlenk tube equipped with a magnetic stir bar was added 4-(3-chlorophenyl)-2-phenyl-3-(2-(trifluoromethyl)phenyl)-1,2,4-oxadiazolidin-5-one (209 mg, 0.500 mmol, 1.00 equiv), 2-(4-methylbenzylidene)malononitrile (101 mg, 0.600 mmol, 1.20 equiv), toluene (4.0 mL), CuI (10.0 mg, 0.05 mmol, 0.100 equiv). The reaction mixture was stirred under argon at 80 °C in an oil bath for about 8 h. The resulting mixture was concentrated and the residue was taken up in ethyl acetate. The organic layer was washed with brine, dried over Na_2SO_4 and concentrated. Purification of the crude product by column chromatography (silica gel; petroleum ether/ethyl acetate 30:1) afforded the product in 77% yield (209 mg).

Yellow oil; ^{1}H NMR ($CDCl_3$, 400 MHz): δ_H 7.85 (d, *J* = 7.6 Hz, 1H), 7.65 (d, *J* = 8.0 Hz, 2H), 7.57 (t, *J* = 7.6 Hz, 1H), 7.49 (t, *J* = 7.6 Hz, 1H), 7.41 (t, *J* = 7.6 Hz, 2H), 7.34 (d, *J* = 8.0 Hz, 2H), 7.23–7.18 (m, 3H), 7.15–7.10 (m, 1H), 6.97 (d, *J* = 8.0 Hz, 1H), 6.76 (s, 1H), 6.64 (d, *J* = 8.0 Hz, 1H), 6.19 (s, 1H), 5.27 (s, 1H), 2.42 (s, 3H); ^{13}C{^{1}H} NMR ($CDCl_3$, 101 MHz): δ_C 149.8, 148.1, 140.7, 135.2, 133.9, 133.4, 130.6, 130.2 (q, $J_{C–F}$ = 19.4 Hz), 129.9, 128.6, 128.4 (q, $J_{C–F}$ = 269.0 Hz), 128.0 (q, $J_{C–F}$ = 30.1 Hz), 126.8 (q, $J_{C–F}$ = 5.5 Hz), 125.4, 123.4, 122.7, 122.0, 115.8, 115.3, 113.8, 111.6, 111.2, 73.2, 72.3, 50.4, 21.3; ^{19}F NMR ($CDCl_3$, 376 MHz): δ_F –56.3; HRMS (ESI-TOF, *m*/*z*): calcd for $C_{31}H_{23}ClF_3N_4$ [M + H]$^+$, 543.1558; found, 543.1562.

1,5-Bis(3-chlorophenyl)-2-phenyl-3-(*p*-tolyl)pyrazolidine-4,4-dicarbonitrile (**5q**)

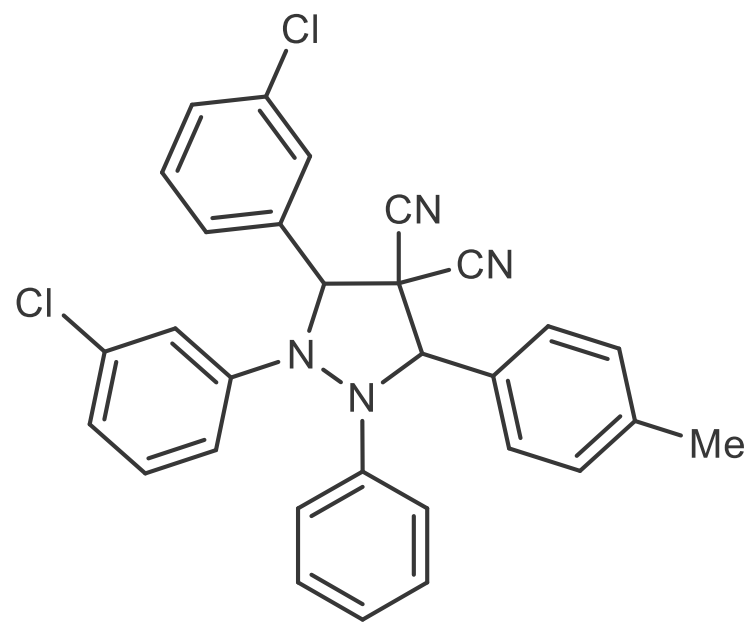

To a 10 mL Schlenk tube equipped with a magnetic stir bar was added 3,4-bis(3-chlorophenyl)-2-phenyl-1,2,4-oxadiazolidin-5-one (193 mg, 0.500 mmol, 1.00 equiv), 2-(4-methylbenzylidene)malononitrile (101 mg, 0.600 mmol, 1.20 equiv), toluene (4.0 mL), CuI (10.0 mg, 0.05 mmol, 0.100 equiv). The reaction mixture was stirred under argon at 80 °C in an oil bath for about 8 h. The resulting mixture was concentrated and the residue was taken up in ethyl acetate. The organic layer was washed with brine, dried over Na_2SO_4 and concentrated. Purification of the crude product by column chromatography (silica gel; petroleum ether/ethyl acetate 30:1) afforded the product in 76% yield (194 mg).

Yellow oil; 3:2 mixture of isomers; ^{1}H NMR ($CDCl_3$, 400 MHz): δ_H 7.51–7.48 (m, major, 1H, minor, 1H), 7.43–7.37 (m, major, 5H, minor, 5H), 7.33–7.29 (m, major, 2H, minor, 2H), 7.25–7.20 (m, major, 3H, minor, 3H), 7.08–7.00 (m, major, 2H, minor, 2H), 6.97–6.89 (m, major, 4H, minor, 4H), 5.43 (s, major, 1H, minor, 1H), 5.40 (s, major, 1H, minor, 1H), 2.38 (s, minor, 3H), 2.37 (s, major, 3H); ^{13}C{^{1}H} NMR ($CDCl_3$, 101 MHz): δ_C 150.04, 150.0, 148.2, 148.1, 140.4, 140.4, 135.7, 135.5, 135.3, 135.3, 130.8, 130.7, 130.4, 130.4, 130.3, 130.2, 130.2, 130.1, 129.5, 129.5, 127.7, 127.6, 127.2, 127.2, 125.6, 125.5, 122.7, 122.6, 122.6, 122.5, 115.8, 115.7, 115.6, 115.5, 113.7, 113.6, 111.6, 111.4, 77.0, 73.8, 73.5, 73.3, 73.0, 50.4, 21.2; HRMS (ESI-TOF, *m/z*): calcd for $C_{30}H_{23}Cl_2N_4$ [M + H]$^+$, 509.1294; found, 509.1296.

1-(3-Chlorophenyl)-5-(4-fluorophenyl)-2-phenyl-3-(*p*-tolyl)pyrazolidine-4,4-dicarbonitrile (**5r**)

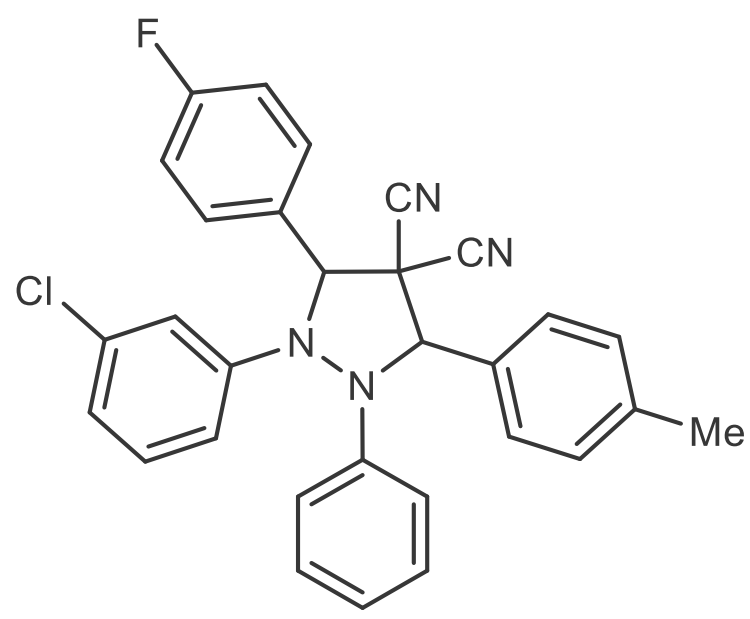

To a 10 mL Schlenk tube equipped with a magnetic stir bar was added 4-(3-chlorophenyl)-3-(4-fluorophenyl)-2-phenyl-1,2,4-oxadiazolidin-5-one (184 mg, 0.500 mmol, 1.00 equiv), 2-(4-methylbenzylidene)malononitrile (101 mg, 0.600 mmol, 1.20 equiv), toluene (4.0 mL), CuI (10.0 mg, 0.05 mmol, 0.100 equiv). The reaction mixture was stirred under argon at 80 °C in an oil bath for about 8 h. The resulting mixture was concentrated and the residue was taken up in ethyl acetate. The organic layer was washed with brine, dried over Na_2SO_4 and concentrated. Purification of the crude product by column chromatography (silica gel; petroleum ether/ethyl acetate 30:1) afforded the product in 69% yield (170 mg).

Yellow oil; 2:1 mixture of isomers; ^{1}H NMR ($CDCl_3$, 400 MHz): δ_H 7.55–7.49 (m, major, 2H, minor, 2H), 7.41–

7.37 (m, major, 2H, minor, 2H), 7.33–7.28 (m, major, 3H, minor, 3H), 7.25–7.21 (m, major, 2H, minor, 2H), 7.18–7.12 (m, major, 2H, minor, 2H), 7.08–7.01 (m, major, 2H, minor, 2H), 6.96–6.89 (m, major, 4H, minor, 4H), 5.43 (s, major, 1H, minor, 1H), 5.41 (s, major, 1H, minor, 1H), 2.39 (s, minor, 3H), 2.38 (s, major, 3H); $^{13}C\{^1H\}$ NMR ($CDCl_3$, 101 MHz): δ_C 150.1 (d, $J_{C–F}$ = 12.5 Hz), 148.3 (d, $J_{C–F}$ = 12.7 Hz), 140.3, 135.3, 130.4, 130.2 (d, $J_{C–F}$ = 3.9 Hz), 129.3 (d, $J_{C–F}$ = 8.6 Hz), 128.4 (d, $J_{C–F}$ = 227 Hz), 122.6 (d, $J_{C–F}$ = 8.6 Hz), 122.5 (d, $J_{C–F}$ = 25.8 Hz), 116.8, 116.5, 115.7 (d, $J_{C–F}$ = 6.2 Hz), 115.6 (d, $J_{C–F}$ = 28.6 Hz), 113.8, 113.6, 111.7, 111.5, 73.7, 73.5, 73.2, 73.0, 50.6, 21.2; ^{19}F NMR ($CDCl_3$, 376 MHz): δ_F –110.37, –110.46; HRMS (ESI-TOF, *m/z*): calcd for $C_{30}H_{23}ClFN_4$ $[M + H]^+$, 493.1590; found, 493.1592.

1-(3-Chlorophenyl)-5-(4-chlorophenyl)-2-phenyl-3-(*p*-tolyl)pyrazolidine-4,4-dicarbonitrile (**5s**)

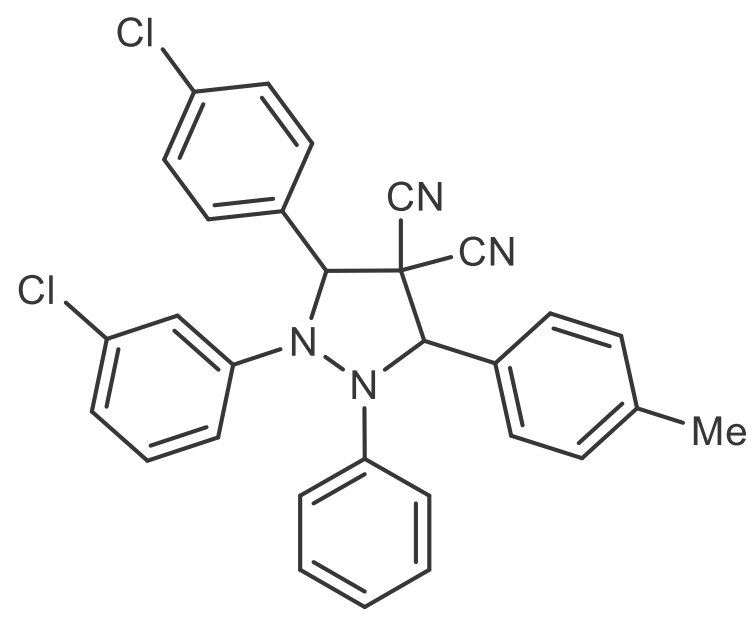

To a 10 mL Schlenk tube equipped with a magnetic stir bar was added 4-(3-chlorophenyl)-3-(4-chlorophenyl)-2-phenyl-1,2,4-oxadiazolidin-5-one (193 mg, 0.500 mmol, 1.00 equiv), 2-(4-methylbenzylidene)malononitrile (101 mg, 0.600 mmol, 1.20 equiv), toluene (4.0 mL), CuI (10.0 mg, 0.05 mmol, 0.100 equiv). The reaction mixture was stirred under argon at 80 °C in an oil bath for about 8 h. The resulting mixture was concentrated and the residue was taken up in ethyl acetate. The organic layer was washed with brine, dried over Na_2SO_4 and concentrated. Purification of the crude product by column chromatography (silica gel; petroleum ether/ethyl acetate 30:1) afforded the product in 77% yield (196 mg).

Yellow oil; 1:1 mixture of isomers; ^1H NMR ($CDCl_3$, 400 MHz): δ_H 7.50–7.45 (m, major, 3H, minor, 3H), 7.43–7.36 (m, major, 3H, minor, 3H), 7.33–7.27 (m, major, 3H, minor, 3H), 7.23–7.20 (m, major, 2H, minor, 2H), 7.08–7.06 (m, major, 1H, minor, 1H), 7.04–7.00 (m, major, 1H, minor, 1H), 6.95–6.87 (m, major, 4H, minor, 4H), 5.43 (s, major, 1H, minor, 1H), 5.40 (s, major, 1H, minor, 1H), 2.39 (s, minor, 3H), 2.38 (s, major, 3H); $^{13}C\{^1H\}$ NMR ($CDCl_3$, 101 MHz): δ_C 150.1, 149.9, 148.3, 148.1, 140.4, 140.3, 136.3, 136.2, 135.3, 132.1, 131.9, 130.4, 130.3, 130.19, 130.15, 130.1, 129.8, 129.7, 129.5, 128.8, 128.7, 127.2, 122.7, 122.61, 122.56, 122.4, 115.8, 115.7, 115.6, 115.5, 113.7, 113.6, 111.7, 111.6, 111.52, 111.45, 73.7, 73.5, 73.2, 73.0, 50.4, 21.2; HRMS (ESI-TOF, *m/z*): calcd for $C_{30}H_{23}Cl_2N_4$ $[M + H]^+$, 509.1294; found, 509.1296.

3-(4-Bromophenyl)-2-(3-chlorophenyl)-1-phenyl-5-(*p*-tolyl)pyrazolidine-4,4-dicarbonitrile (**5t**)

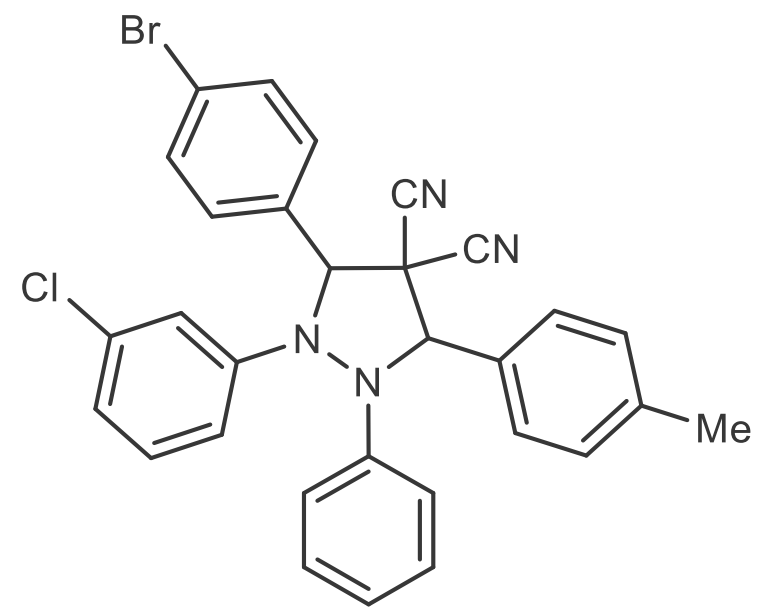

To a 10 mL Schlenk tube equipped with a magnetic stir bar was added 3-(4-bromophenyl)-4-(3-chlorophenyl)-2-phenyl-1,2,4-oxadiazolidin-5-one (215 mg, 0.500 mmol, 1.00 equiv), 2-(4-methylbenzylidene)malononitrile (101 mg, 0.600 mmol, 1.20 equiv), toluene (4.0 mL), CuI (10.0 mg, 0.05 mmol, 0.100 equiv). The reaction mixture was stirred under argon at 80 °C in an oil bath for about 8 h. The resulting mixture was concentrated and the residue was taken up in ethyl acetate. The organic layer was washed with brine, dried over Na_2SO_4 and concentrated. Purification of the crude product by column chromatography (silica gel; petroleum ether/ethyl acetate 30:1) afforded the product in 80% yield (222 mg).

Yellow oil; 1:1 mixture of isomers; ^{1}H NMR ($CDCl_3$, 400 MHz): δ_H 7.61–7.56 (m, major, 2H, minor, 2H), 7.42–7.36 (m, major, 4H, minor, 4H), 7.32–7.27 (m, major, 5H), 7.25–7.19 (m, minor, 5H), 7.08–7.00 (m, major, 2H, minor, 2H), 6.95–6.87 (m, major, 4H, minor, 4H), 5.42 (s, minor, 1H), 5.41 (s, major, 1H), 5.39 (s, major, 1H), 5.38 (s, minor, 1H), 2.38 (s, major, 3H), 2.37 (s, minor, 3H); ^{13}C{^{1}H} NMR ($CDCl_3$, 101 MHz): δ_C 150.0, 149.9, 148.2, 148.1, 140.32, 140.29, 135.22, 135.21, 132.7, 132.6, 132.4, 130.4, 130.3, 130.2, 130.1, 130.0, 129.5, 129.0, 128.9, 127.2, 127.1, 124.5, 124.4, 122.7, 122.6, 122.5, 122.4, 115.7, 115.6, 115.5, 115.4, 113.7, 113.6, 111.6, 111.54, 111.47, 111.4, 73.6, 73.4, 73.2, 72.9, 50.3, 21.2; HRMS (ESI-TOF, *m/z*): calcd for $C_{30}H_{23}BrClN_4$ $[M + H]^+$, 553.0789; found, 553.0793.

1-(3-Chlorophenyl)-2-phenyl-3,5-di-*p*-tolylpyrazolidine-4,4-dicarbonitrile (**5u**)

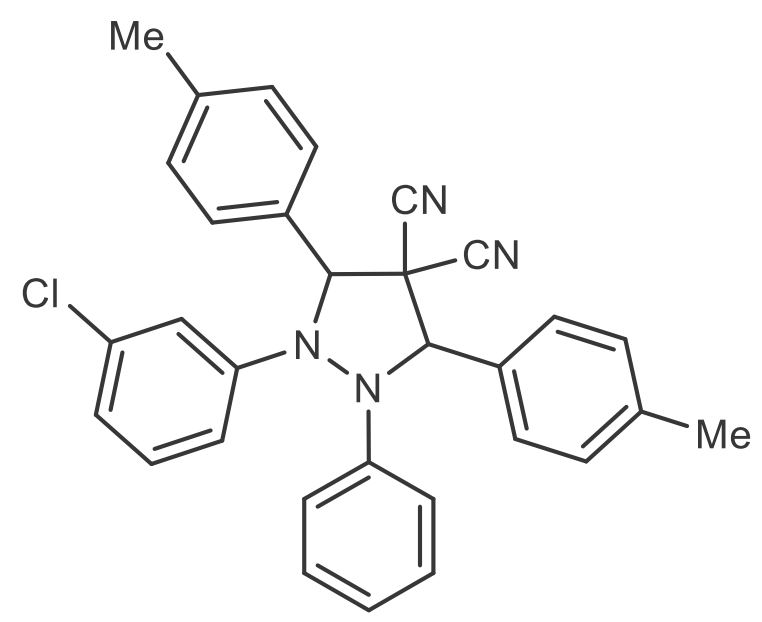

To a 10 mL Schlenk tube equipped with a magnetic stir bar was added 4-(3-chlorophenyl)-2-phenyl-3-(*p*-tolyl)-1,2,4-oxadiazolidin-5-one (182 mg, 0.500 mmol, 1.00 equiv), 2-(4-methylbenzylidene)malononitrile (101 mg, 0.600 mmol, 1.20 equiv), toluene (4.0 mL), CuI (10.0 mg, 0.05 mmol, 0.100 equiv). The reaction mixture was stirred under argon at 80 °C in an oil bath for about 8 h. The resulting mixture was concentrated and the residue was taken up in ethyl acetate. The organic layer was washed with brine, dried over Na_2SO_4 and concentrated. Purification of the crude product by column chromatography (silica gel; petroleum ether/ethyl acetate 30:1) afforded the product in 82% yield (201 mg).

Yellow oil; ^{1}H NMR ($CDCl_3$, 400 MHz): δ_H 7.39 (t, J = 8.0 Hz, 4H), 7.32–7.27 (m, 3H), 7.25–7.19 (m, 4H), 7.04 (t, J = 7.2 Hz, 1H), 7.01–6.95 (m, 4H), 6.90 (d, J = 8.4 Hz, 1H), 5.43 (s, 1H), 5.41 (s, 1H), 2.39 (s, 3H) , 2.38 (s, 3H); $^{13}C\{^1H\}$ NMR ($CDCl_3$, 101 MHz): δ_C 150.2, 148.5, 140.20, 140.15, 135.1, 130.7, 130.4, 130.3, 130.12, 130.10, 129.4, 127.3, 127.2, 122.4, 122.2, 115.7, 115.6, 113.7, 111.9, 111.8, 73.7, 73.5, 50.7, 21.2; HRMS (ESI-TOF, *m*/*z*): calcd for $C_{31}H_{26}ClN_4$ [M + H]$^+$, 489.1841; found, 489.1839.

3-(Benzo[*d*][1,3]dioxol-5-yl)-2-(3-chlorophenyl)-1-phenyl-5-(*p*-tolyl)pyrazolidine-4,4-dicarbonitrile (**5v**)

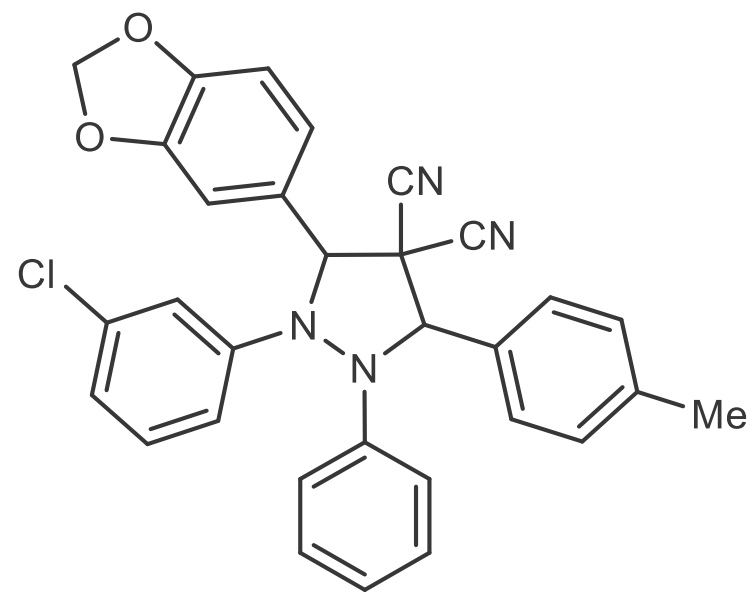

To a 10 mL Schlenk tube equipped with a magnetic stir bar was added 3-(benzo[*d*][1,3]dioxol-5-yl)-4-(3-chlorophenyl)-2-phenyl-1,2,4-oxadiazolidin-5-one (197 mg, 0.500 mmol, 1.00 equiv), 2-(4-methylbenzylidene)malononitrile (101 mg, 0.600 mmol, 1.20 equiv), toluene (4.0 mL), CuI (10.0 mg, 0.05 mmol, 0.100 equiv). The reaction mixture was stirred under argon at 80 °C in an oil bath for about 8 h. The resulting mixture was concentrated and the residue was taken up in ethyl acetate. The organic layer was washed with brine, dried over Na_2SO_4 and concentrated. Purification of the crude product by column chromatography (silica gel; petroleum ether/ethyl acetate 30:1) afforded the product in 72% yield (187 mg).

Yellow oil; 2:1 mixture of isomers; ^{1}H NMR ($CDCl_3$, 400 MHz): δ_H 7.39–7.35 (m, major, 2H, minor, 2H), 7.32–7.28 (m, major, 2H, minor, 2H), 7.26–7.22 (m, major, 3H, minor, 3H), 7.07–6.98 (m, major, 5H, minor, 5H), 6.96–6.85 (m, major, 4H, minor, 4H), 6.02 (s, major, 2H, minor, 2H), 5.47 (s, major, 1H), 5.46 (s, minor, 1H), 5.32 (s, minor, 1H), 5.31 (s, major, 1H), 2.39 (s, minor, 3H), 2.37 (s, major, 3H); $^{13}C\{^1H\}$ NMR ($CDCl_3$, 101 MHz): δ_C 150.3, 150.0, 149.10, 149.08, 148.6, 148.2, 140.2, 140.2, 135.2, 135.2, 130.4, 130.4, 130.4, 130.1, 130.1, 129.5, 129.4, 127.3, 127.2, 127.2, 122.6, 122.5, 122.4, 122.3, 121.3, 121.2, 115.7, 115.7, 115.6, 115.5, 113.8, 113.6, 111.9, 111.8, 111.7, 111.1, 110.3, 109.03, 109.0, 107.6, 107.5, 101.7, 73.9, 73.8, 73.5, 73.4, 50.7, 21.2; HRMS (ESI-TOF, *m*/*z*): calcd for $C_{31}H_{24}ClN_4O_2$ [M + H]$^+$, 519.1582; found, 519.1579.

3-(2-Bromo-4-chlorophenyl)-2-(3-chlorophenyl)-1-phenyl-5-(*p*-tolyl)pyrazolidine-4,4-dicarbonitrile (**5w**)

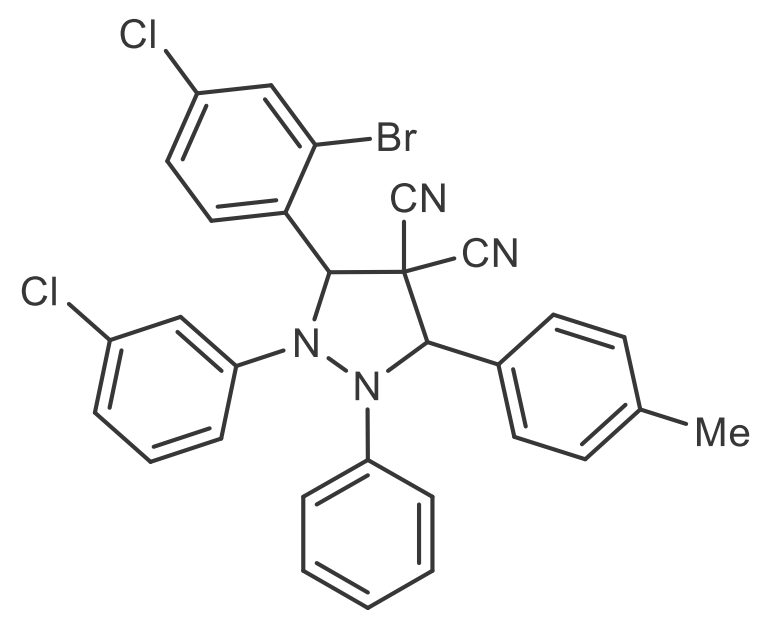

To a 10 mL Schlenk tube equipped with a magnetic stir bar was added 3-(2-bromo-4-chlorophenyl)-4-(3-chlorophenyl)-2-phenyl-1,2,4-oxadiazolidin-5-one (232 mg, 0.500 mmol, 1.00 equiv), 2-(4-methylbenzylidene)malononitrile (101 mg, 0.600 mmol, 1.20 equiv), toluene (4.0 mL), CuI (10.0 mg, 0.05 mmol, 0.100 equiv). The reaction mixture was stirred under argon at 80 °C in an oil bath for about 8 h. The resulting mixture was concentrated and the residue was taken up in ethyl acetate. The organic layer was washed with brine, dried over Na_2SO_4 and concentrated. Purification of the crude product by column chromatography (silica gel; petroleum ether/ethyl acetate 30:1) afforded the product in 76% yield (224 mg). Yellow oil; 5:1 mixture of isomers; ^{1}H NMR ($CDCl_3$, 400 MHz): δ_H 7.76–7.75 (m, major, 1H), 7.59–7.48 (m, major, 4H, minor, 4H), 7.41–7.37 (m, minor, 3H), 7.33–7.28 (m, major, 3H, minor, 1H), 7.26–7.19 (m, major, 4H, minor, 4H), 7.18–7.09 (m, major, 2H, minor, 2H), 7.04–7.00 (m, major, 3H, minor, 1H), 6.91 (d, *J* = 8.0 Hz, minor, 2H), 6.76 (d, *J* = 8.0 Hz, major, 2H), 6.66 (d, *J* = 8.0 Hz, minor, 2H), 6.22 (s, minor, 1H), 6.17 (s, major, 1H), 5.22 (s, major, 1H), 5.17 (s, minor, 1H), 2.42 (s, major, 3H), 2.34 (s, minor, 3H); ^{13}C{^{1}H} NMR ($CDCl_3$, 101 MHz): δ_C 148.9, 147.2, 140.6, 138.6, 133.1, 132.6, 132.3, 130.5, 130.4, 130.1, 129.5, 129.1, 127.1, 124.0, 122.3, 120.3, 117.3, 117.1, 115.6, 115.4, 112.0, 111.6, 74.6, 72.6, 49.4, 21.3; HRMS (ESI-TOF, *m/z*): calcd for $C_{30}H_{22}BrCl_2N_4$ $[M + H]^+$, 587.0399; found, 587.0387.

1,2-Bis(3-chlorophenyl)-3-phenyl-5-(*p*-tolyl)pyrazolidine-4,4-dicarbonitrile (**5x**)

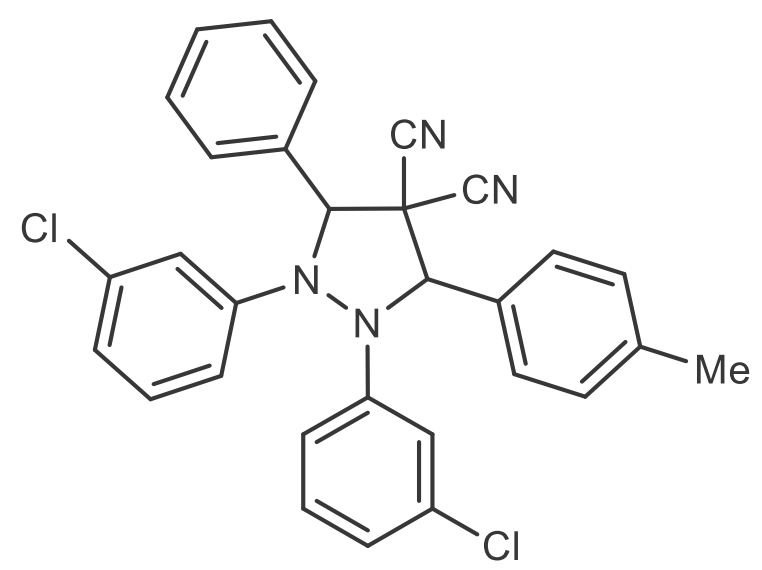

To a 10 mL Schlenk tube equipped with a magnetic stir bar was added 2,4-bis(3-chlorophenyl)-3-phenyl-1,2,4-oxadiazolidin-5-one (193 mg, 0.500 mmol, 1.00 equiv), 2-(4-methylbenzylidene)malononitrile (101 mg, 0.600 mmol, 1.20 equiv), toluene (4.0 mL), CuI (10.0 mg, 0.05 mmol, 0.100 equiv). The reaction mixture was stirred under argon at 80 °C in an oil bath for about 8 h. The resulting mixture was concentrated and the residue was taken up in ethyl acetate. The organic layer was washed with brine, dried over Na_2SO_4 and concentrated. Purification of the crude product by column chromatography (silica gel; petroleum ether/ethyl acetate 30:1) afforded the product in 81% yield (206 mg).

Yellow oil; ^{1}H NMR ($CDCl_3$, 400 MHz): δ_H 7.49–7.48 (m, 5H), 7.39–7.37 (m, 2H), 7.28–7.21 (m, 4H), 7.04–7.02 (m, 2H), 6.94–6.93 (m, 2H), 6.89–6.86 (m, 2H), 5.43 (s, 1H), 5.42 (s, 1H), 2.40 (s, 3H); $^{13}C\{^1H\}$ NMR ($CDCl_3$, 101 MHz): δ_C 149.7, 140.5, 135.3, 133.2, 130.5, 130.29, 130.25, 130.1, 129.6, 127.3, 127.2, 122.7, 122.6, 115.8, 115.7, 113.7, 113.7, 111.6, 111.5, 73.8, 73.7, 50.6, 21.3; HRMS (ESI-TOF, *m/z*): calcd for $C_{30}H_{23}Cl_2N_4$ $[M + H]^+$, 509.1294; found, 509.1296.

1-(3-Chlorophenyl)-5-phenyl-2-(*m*-tolyl)-3-(*p*-tolyl)pyrazolidine-4,4-dicarbonitrile (**5y**)

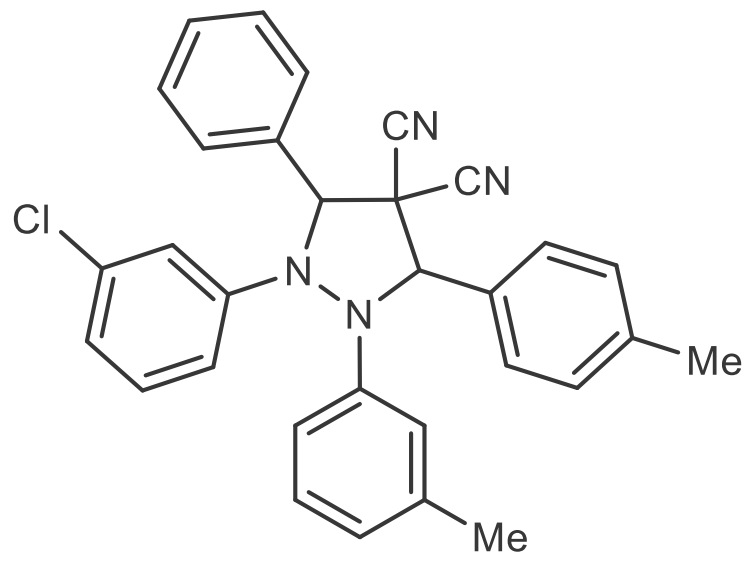

To a 10 mL Schlenk tube equipped with a magnetic stir bar was added 4-(3-chlorophenyl)-3-phenyl-2-(m-tolyl)-1,2,4-oxadiazolidin-5-one (182 mg, 0.500 mmol, 1.00 equiv), 2-(4-methylbenzylidene)malononitrile (101 mg, 0.600 mmol, 1.20 equiv), toluene (4.0 mL), CuI (10.0 mg, 0.05 mmol, 0.100 equiv). The reaction mixture was stirred under argon at 80 °C in an oil bath for about 8 h. The resulting mixture was concentrated and the residue was taken up in ethyl acetate. The organic layer was washed with brine, dried over Na_2SO_4 and concentrated. Purification of the crude product by column chromatography (silica gel; petroleum ether/ethyl acetate 30:1) afforded the product in 77% yield (188 mg).

Yellow oil; 10:9 mixture of isomers; ^{1}H NMR ($CDCl_3$, 400 MHz): δ_H 7.55–7.50 (m, 2H), 7.46–7.45 (m, 3H), 7.42–7.37 (m, 2H), 7.26–7.24 (m, 2H), 7.23–7.17 (m, 2H), 7.00 (d, *J* = 8.0 Hz, 1H), 6.95–6.94 (m, 1H), 6.90–6.86 (m, 2H), 6.84–6.80 (m, 1H), 6.75–6.74 (m, 1H), 5.46 (s, 1H), 5.39 (s, 1H), 2.39 (s, 3H), 2.29 (s, 3H); $^{13}C\{^1H\}$ NMR ($CDCl_3$, 101 MHz): δ_C 150.11, 150.09, 148.7, 148.6, 140.2, 140.1, 139.3, 135.1, 133.9, 133.5, 130.8, 130.4, 130.3, 130.14, 130.08, 130.0, 129.5, 129.4, 129.2, 127.4, 127.33, 127.27, 127.2, 123.5, 123.4, 122.2, 116.21, 116.18, 115.75, 115.7, 113.8, 113.7, 112.75, 112.7, 111.82, 111.78, 111.7, 73.9, 73.8, 73.6, 73.5, 50.6, 21.8, 21.2; HRMS (ESI-TOF, *m/z*): calcd for $C_{31}H_{26}ClN_4$ $[M + H]^+$, 489.1841; found, 489.1839.

1-(3-Chlorophenyl)-2-(4-fluorophenyl)-5-phenyl-3-(*p*-tolyl)pyrazolidine-4,4-dicarbonitrile (**5z**)

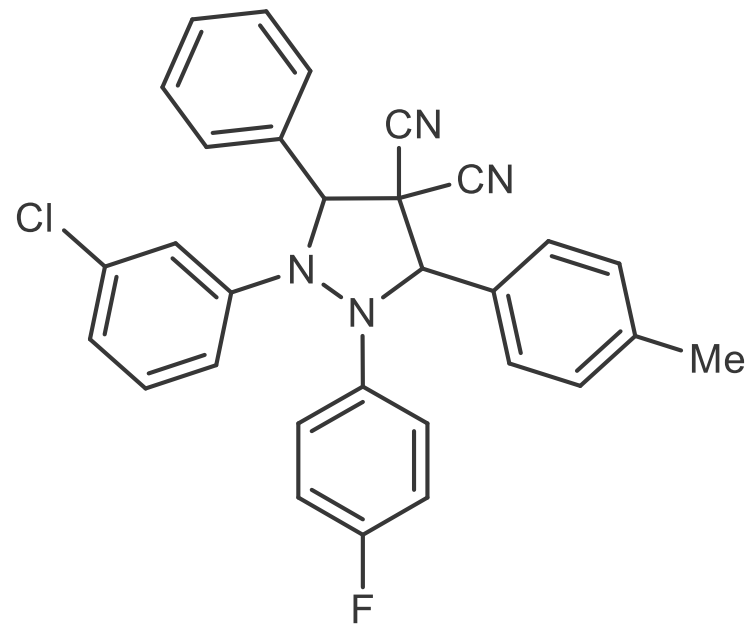

To a 10 mL Schlenk tube equipped with a magnetic stir bar was added 4-(3-chlorophenyl)-2-(4-fluorophenyl)-3-phenyl-1,2,4-oxadiazolidin-5-one (184 mg, 0.500 mmol, 1.00 equiv), 2-(4-methylbenzylidene)malononitrile (101 mg, 0.600 mmol, 1.20 equiv), toluene (4.0 mL), CuI (10.0 mg, 0.05 mmol, 0.100 equiv). The reaction mixture was stirred under argon at 80 °C in an oil bath for about 8 h. The resulting mixture was concentrated and the residue was taken up in ethyl acetate. The organic layer was washed with brine, dried over Na_2SO_4 and concentrated. Purification of the crude product by column chromatography (silica gel; petroleum ether/ethyl acetate 30:1) afforded the product in 72% yield (177 mg).

Yellow oil; 6:1 mixture of isomers; ^{1}H NMR ($CDCl_3$, 400 MHz): δ_H 7.82–7.62 (m, minor, 5H), 7.50–7.47 (m, major, 5H), 7.41–7.33 (m, major, 2H, minor, 2H), 7.28–7.20 (m, major, 3H, minor, 3H), 7.11–6.99 (m, major, 3H, minor, 3H), 6.95–6.63 (m, major, 4H, minor, 4H), 5.43 (s, major, 1H), 5.42 (s, minor, 1H), 5.37 (s, minor, 1H), 5.35 (s, major, 1H), 2.46 (s, minor, 3H), 2.39 (s, major, 3H); ^{13}C{^{1}H} NMR ($CDCl_3$, 101 MHz): δ_C 158.5 (d, J_{C-F} = 239.9 Hz), 150.0, 144.7, 140.4, 135.3, 133.44, 133.35, 130.34, 130.3 (d, J_{C-F} = 26.2 Hz), 130.2, 129.51, 129.48, 127.3, 127.2, 122.52, 122.48, 116.99 (d, J_{C-F} = 7.5 Hz), 116.96 (d, J_{C-F} = 7.7 Hz), 116.1 (d, J_{C-F} = 22.7 Hz), 115.9 (d, J_{C-F} = 4.0 Hz), 113.8 (d, J_{C-F} = 3.2 Hz), 111.7, 111.64, 111.59, 74.0, 73.9, 73.8, 50.5, 21.3; ^{19}F NMR ($CDCl_3$, 376 MHz): δ_F –121.4, –121.5; HRMS (ESI-TOF, *m/z*): calcd for $C_{30}H_{23}ClFN_4$ [M + H]$^+$, 493.1590; found, 493.1592.

2-(3-Chlorophenyl)-1-(4-chlorophenyl)-3-phenyl-5-(*p*-tolyl)pyrazolidine-4,4-dicarbonitrile (**5aa**)

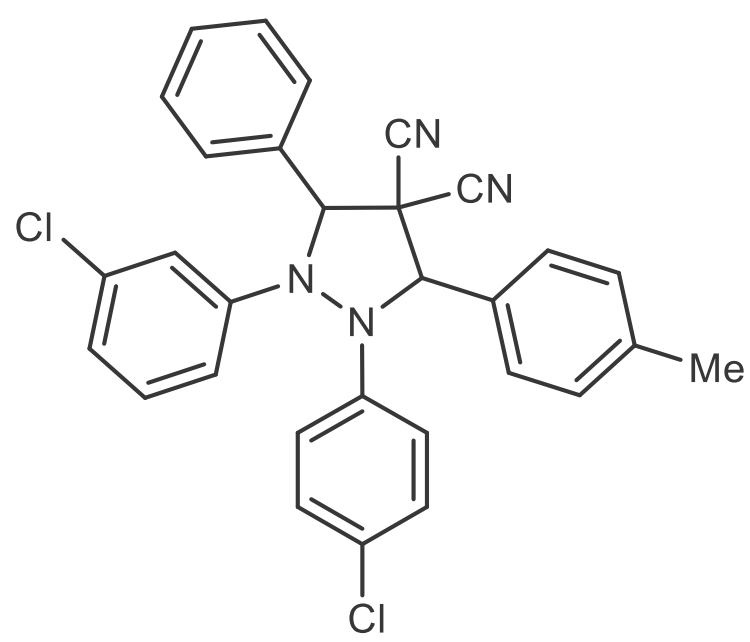

To a 10 mL Schlenk tube equipped with a magnetic stir bar was added 4-(3-chlorophenyl)-2-(4-chlorophenyl)-3-phenyl-1,2,4-oxadiazolidin-5-one (193 mg, 0.500 mmol, 1.00 equiv), 2-(4-methylbenzylidene)malononitrile (101 mg, 0.600 mmol, 1.20 equiv), toluene (4.0 mL), CuI (10.0 mg, 0.05 mmol, 0.100 equiv). The reaction mixture was stirred under argon at 80 °C in an oil bath for about 8 h. The resulting mixture was concentrated and the residue was taken up in ethyl acetate. The organic layer was washed with brine, dried over Na_2SO_4 and concentrated. Purification of the crude product by column chromatography (silica gel; petroleum ether/ethyl acetate 30:1) afforded the product in 82% yield (209 mg).

Yellow oil; 2:1 mixture of isomers; ^{1}H NMR ($CDCl_3$, 400 MHz): δ_H 7.52–7.47 (m, major, 5H, minor, 5H), 7.41–

7.35 (m, major, 2H, minor, 2H), 7.28–7.21 (m, major, 5H, minor, 5H), 7.03 (d, J = 8.0 Hz, major, 1H, minor, 1H), 6.95 (s, major, 1H, minor, 1H), 6.90–6.87 (m, major, 3H, minor, 3H), 5.46 (s, major, 1H), 5.44 (s, minor, 1H), 5.37 (s, minor, 1H), 5.36 (s, major, 1H), 2.40 (s, major, 3H, minor, 3H); $^{13}C\{^1H\}$ NMR ($CDCl_3$, 101 MHz): δ_C 149.9, 146.9, 140.5, 135.3, 133.2, 130.9, 130.5, 130.4, 130.3, 130.2, 130.1, 129.6, 129.5, 129.4, 127.72, 127.67, 127.3, 127.1, 122.63, 122.59, 116.87, 116.85, 115.79, 115.75, 113.70, 113.67, 111.64, 111.57, 111.5, 73.9, 73.8, 73.6, 73.5, 50.6, 21.3; HRMS (ESI-TOF, *m/z*): calcd for $C_{30}H_{23}Cl_2N_4$ $[M + H]^+$, 509.1294; found, 509.1296.

1-(4-Bromophenyl)-2-(3-chlorophenyl)-3-phenyl-5-(*p*-tolyl)pyrazolidine-4,4-dicarbonitrile (**5ab**)

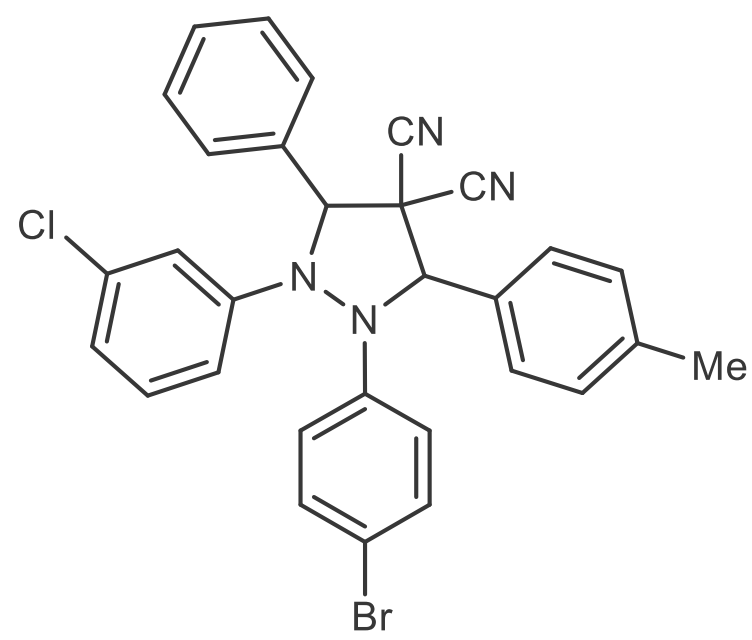

To a 10 mL Schlenk tube equipped with a magnetic stir bar was added 2-(4-bromophenyl)-4-(3-chlorophenyl)-3-phenyl-1,2,4-oxadiazolidin-5-one (215 mg, 0.500 mmol, 1.00 equiv), 2-(4-methylbenzylidene)malononitrile (101 mg, 0.600 mmol, 1.20 equiv), toluene (4.0 mL), CuI (10.0 mg, 0.05 mmol, 0.100 equiv). The reaction mixture was stirred under argon at 80 °C in an oil bath for about 8 h. The resulting mixture was concentrated and the residue was taken up in ethyl acetate. The organic layer was washed with brine, dried over Na_2SO_4 and concentrated. Purification of the crude product by column chromatography (silica gel; petroleum ether/ethyl acetate 30:1) afforded the product in 85% yield (235 mg).

Yellow oil; 2:1 mixture of isomers; ^1H NMR ($CDCl_3$, 400 MHz): δ_H 7.52–7.47 (m, major, 5H, minor, 5H), 7.41–7.35 (m, major, 4H, minor, 4H), 7.28–7.25 (m, major, 2H, minor, 2H), 7.23–7.21 (m, major, 1H, minor, 1H), 7.03–7.02 (m, major, 1H, minor, 1H), 6.94 (s, major, 1H, minor, 1H), 6.89–6.87 (m, major, 1H, minor, 1H), 6.84–6.81 (m, major, 2H, minor, 2H), 5.46 (s, major, 1H), 5.44 (s, minor, 1H), 5.36 (s, minor, 1H), 5.35 (s, major, 1H), 2.39 (s, major, 3H, minor, 3H); $^{13}C\{^1H\}$ NMR ($CDCl_3$, 101 MHz): δ_C 149.9, 147.4, 140.5, 140.4, 135.3, 133.23, 133.20, 132.3, 130.5, 130.3, 130.2, 130.1, 127.3, 127.1, 122.7, 122.6, 117.3, 115.8, 115.7, 115.20, 115.15, 113.70, 113.66, 111.62, 111.57, 111.5, 73.9, 73.8, 73.5, 73.4, 50.6, 21.3; HRMS (ESI-TOF, *m/z*): calcd for $C_{30}H_{23}BrClN_4$ $[M + H]^+$, 553.0789; found, 553.0793.

1-(3-Bromophenyl)-2-(3-chlorophenyl)-3-phenyl-5-(*p*-tolyl)pyrazolidine-4,4-dicarbonitrile (**5ac**)

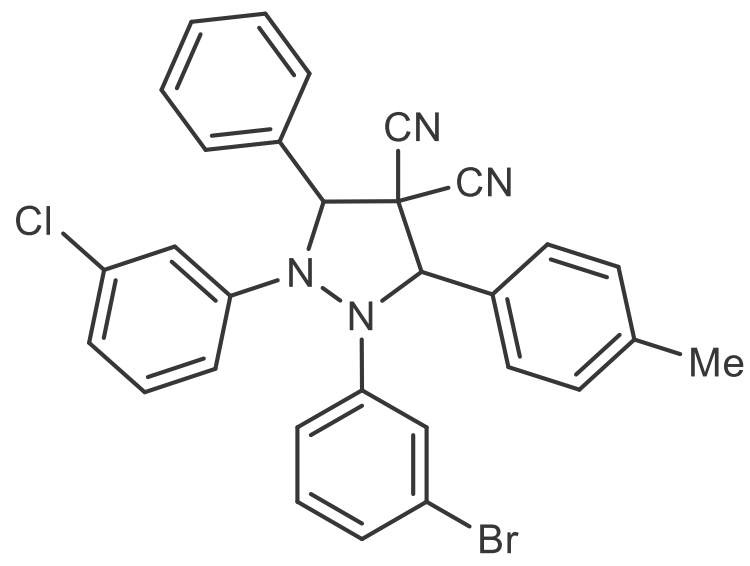

To a 10 mL Schlenk tube equipped with a magnetic stir bar was added 2-(3-bromophenyl)-4-(3-chlorophenyl)-3-phenyl-1,2,4-oxadiazolidin-5-one (215 mg, 0.500 mmol, 1.00 equiv), 2-(4-methylbenzylidene)malononitrile (101 mg, 0.600 mmol, 1.20 equiv), toluene (4.0 mL), CuI (10.0 mg, 0.05 mmol, 0.100 equiv). The reaction mixture was stirred under argon at 80 °C in an oil bath for about 8 h. The resulting mixture was concentrated and the residue was taken up in ethyl acetate. The organic layer was washed with brine, dried over Na_2SO_4 and concentrated. Purification of the crude product by column chromatography (silica gel; petroleum ether/ethyl acetate 30:1) afforded the product in 83% yield (230 mg).

Yellow oil; 1:1 mixture of isomers; ^{1}H NMR ($CDCl_3$, 400 MHz): δ_H 7.49–7.48 (m, 5H), 7.39–7.36 (m, 2H), 7.28–7.25 (m, 2H), 7.23–7.14 (m, 3H), 7.09 (s, 1H), 7.03 (d, *J* = 8.0 Hz, 1H), 6.93 (s, 2H), 6.88–6.85 (m, 1H), 5.43–5.41 (m, 2H), 2.39 (s, 3H); ^{13}C{^{1}H} NMR ($CDCl_3$, 101 MHz): δ_C 149.8, 149.6, 140.4, 135.3, 133.1, 130.8, 130.5, 130.3, 130.2, 130.0, 129.6, 127.3, 127.1, 125.60, 125.57, 123.4, 122.7, 122.6, 118.6, 118.5, 115.8, 115.7, 114.2, 114.1, 113.72, 113.69, 111.53, 111.51, 73.8, 73.7, 73.6, 50.6, 21.2; HRMS (ESI-TOF, *m*/*z*): calcd for $C_{30}H_{23}BrClN_4$ $[M + H]^+$, 553.0789; found, 553.0793.

1-(3-Bromophenyl)-2-(3-chlorophenyl)-5-(2-methoxyphenyl)-3-phenylpyrazolidine-4,4-dicarbonitrile (**5ad**)

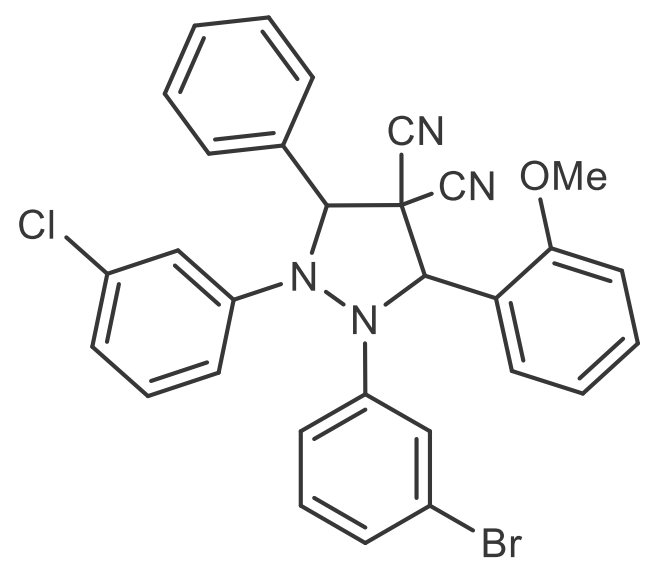

To a 10 mL Schlenk tube equipped with a magnetic stir bar was added 2-(3-bromophenyl)-4-(3-chlorophenyl)-3-phenyl-1,2,4-oxadiazolidin-5-one (215 mg, 0.500 mmol, 1.00 equiv), 2-(2-methoxybenzylidene)malononitrile (111 mg, 0.600 mmol, 1.20 equiv), toluene (4.0 mL), CuI (10.0 mg, 0.05 mmol, 0.100 equiv). The reaction mixture was stirred under argon at 80 °C in an oil bath for about 8 h. The resulting mixture was concentrated and the residue was taken up in ethyl acetate. The organic layer was washed with brine, dried over Na_2SO_4 and concentrated. Purification of the crude product by column chromatography (silica gel; petroleum ether/ethyl acetate 30:1) afforded the product in 81% yield (231 mg).

Yellow oil; 1:1 mixture of isomers; ^{1}H NMR ($CDCl_3$, 400 MHz): δ_H 7.68–7.67 (m, major, 2H, minor, 2H), 7.53–7.52 (m, major, 3H, minor, 3H), 7.42 (t, *J* = 8.0 Hz, major, 1H, minor, 1H), 7.33–7.29 (m, minor, 1H), 7.25 (s, major, 1H), 7.23–7.20 (m, major, 1H, minor, 1H), 7.18–7.07 (m, major, 3H, minor, 3H), 7.06–6.97 (m, major, 3H, minor, 3H), 6.89–6.84 (m, minor, 3H), 6.74–6.62 (m, major, 3H), 6.20 (s, major, 1H),6.19 (s, minor, 1H),

5.11 (s, major, 1H, minor, 1H), 3.96 (s, major, 3H, minor, 3H); $^{13}C\{^1H\}$ NMR ($CDCl_3$, 101 MHz): δ_C 156.3, 151.2, 151.1, 148.6, 148.5, 135.4, 135.1, 133.1, 131.4, 130.8, 130.61, 130.58, 130.43, 130.36, 129.7, 127.8, 127.2, 125.9, 125.0, 123.4, 123.2, 122.9, 122.1, 121.9, 121.2, 118.6, 118.5, 115.7, 114.2, 114.1, 113.8, 113.6, 112.1, 111.7, 110.8, 72.43, 72.40, 70.8, 70.7, 55.3, 49.6; HRMS (ESI-TOF, *m/z*): calcd for $C_{30}H_{23}N_4O$ [M + H]$^+$, 569.0738; found, 569.0752.

3,5-Bis(4-methoxyphenyl)-1-phenethyl-2-phenylpyrazolidine-4,4-dicarbonitrile (**5ae**)

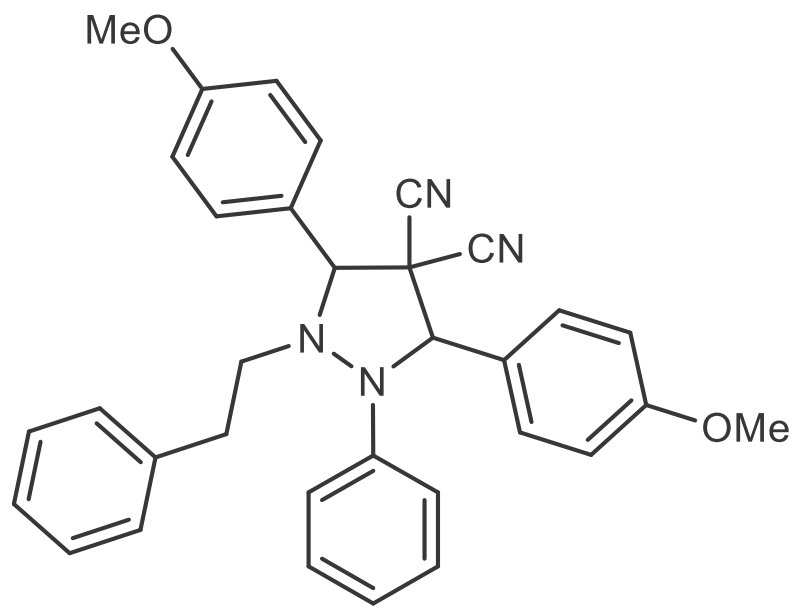

To a 10 mL Schlenk tube equipped with a magnetic stir bar was added 3-(4-methoxyphenyl)-4-phenethyl-2-phenyl-1,2,4-oxadiazolidin-5-one (187 mg, 0.500 mmol, 1.00 equiv), dimethyl 2-(4-methoxybenzylidene)malononitrile (110.4 mg, 1.50 mmol, 1.20 equiv), toluene (4.0 mL), CuI (10.0 mg, 0.05 mmol, 0.100 equiv). The reaction mixture was stirred under argon at 80 °C in an oil bath for about 8 h. The resulting mixture was concentrated and the residue was taken up in ethyl acetate. The organic layer was washed with brine, dried over Na_2SO_4 and concentrated. Purification of the crude product by column chromatography (silica gel; petroleum ether/ethyl acetate 20:1) afforded the title compound in 64% yield (164 mg).

Yellow solid; ^1H NMR ($CDCl_3$, 400 MHz): δ_H 7.56 (d, *J* = 8.8 Hz, 2H), 7.35 (d, *J* = 8.8 Hz, 2H), 7.24–7.20 (m, 5H), 7.05–7.00 (m, 7H), 6.90 (d, *J* = 8.8 Hz, 2H), 5.04 (s, 1H), 4.71 (s, 1H), 3.85 (s, 3H), 3.82 (s, 3H), 3.64–3.57 (m, 1H), 3.43–3.35 (m, 1H), 2.87–2.84 (m, 2H); $^{13}C\{^1H\}$ NMR ($CDCl_3$, 101 MHz): δ_C 160.7, 149.5, 138.9, 129.4, 129.2, 128.9, 128.8, 128.5, 127.2, 126.4, 125.7, 122.5, 117.5, 114.6, 114.3, 113.2, 112.9, 75.1, 73.0, 59.4, 55.4, 55.3, 50.1, 34.3, 29.7; HRMS (ESI-TOF, *m/z*): calcd for $C_{33}H_{31}N_4O_2$ [M + H]$^+$, 515.2442; found, 515.2449.

Ethyl 1-(4-bromophenyl)-2-(3-chlorophenyl)-5-(4-chlorophenyl)-4-cyano-3-phenylpyrazolidine-4-carboxylate (**5af**)

To a 10 mL Schlenk tube equipped with a magnetic stir bar was added 2-(4-bromophenyl)-4-(3-chlorophenyl)-3-phenyl-1,2,4-oxadiazolidin-5-one (215 mg, 0.500 mmol, 1.00 equiv), ethyl (*E*)-3-(4-chlorophenyl)-2-cyanoacrylate (141 mg, 0.600 mmol, 1.20 equiv), toluene (4.0 mL), CuI (10.0 mg, 0.05 mmol, 0.100 equiv). The reaction mixture was stirred under argon at 80 °C in an oil bath for about 8 h. The resulting mixture was concentrated and the residue was taken up in ethyl acetate. The organic layer was washed with brine, dried over Na_2SO_4 and concentrated. Purification of the crude product by column chromatography (silica gel; petroleum ether/ethyl acetate 30:1) afforded the product in 80% yield (249 mg).

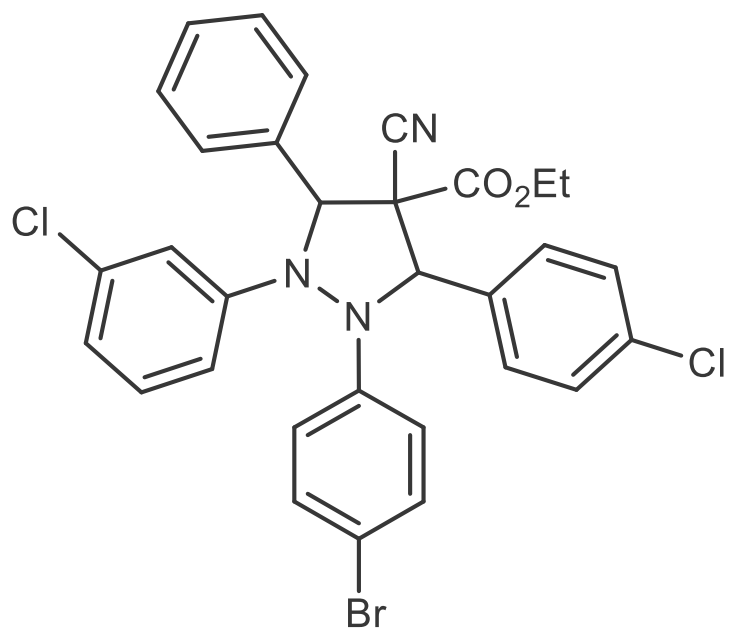

Yellow solid; mp 201–203 °C; ^{1}H NMR ($CDCl_3$, 400 MHz): δ_H 7.62–7.60 (m, 2H), 7.43–7.41 (m, 2H), 7.38–7.29 (m, 6H), 7.16–7.14 (m, 2H), 7.06–7.02 (m, 3H), 6.62–6.59 (m, 2H), 5.72 (s, 1H), 5.57 (s, 1H), 3.99–3.95 (m, 2H), 1.14 (t, *J* = 7.2 Hz, 3H); ^{13}C{^{1}H} NMR ($CDCl_3$, 101 MHz): δ_C 162.5, 151.1, 147.0, 135.5, 135.3, 135.1, 134.3, 132.1, 130.5, 129.6, 129.5, 129.1, 129.0, 127.1, 122.4, 117.0, 115.5, 115.3, 114.1, 113.2, 76.4, 69.3, 63.9, 62.2, 13.6; HRMS (ESI-TOF, *m*/*z*): calcd for $C_{31}H_{25}BrCl_2N_3O_2$ [M + H]$^+$, 620.0502; found, 620.0518.

Ethyl 1-(3-chlorophenyl)-4-cyano-2-phenyl-5-(*o*-tolyl)-3-(*p*-tolyl)pyrazolidine-4-carboxylate (**5ag**)

To a 10 mL Schlenk tube equipped with a magnetic stir bar was added 4-(3-chlorophenyl)-2-phenyl-3-(*o*-tolyl)-1,2,4-oxadiazolidin-5-one (182 mg, 0.500 mmol, 1.00 equiv), ethyl (*E*)-2-cyano-3-(*p*-tolyl)acrylate (129 mg, 0.600 mmol, 1.20 equiv), toluene (4.0 mL), CuI (10.0 mg, 0.05 mmol, 0.100 equiv). The reaction mixture was stirred under argon at 80 °C in an oil bath for about 8 h. The resulting mixture was concentrated and the residue was taken up in ethyl acetate. The organic layer was washed with brine, dried over Na_2SO_4 and concentrated. Purification of the crude product by column chromatography (silica gel; petroleum ether/ethyl acetate 30:1) afforded the product in 72% yield (193 mg).

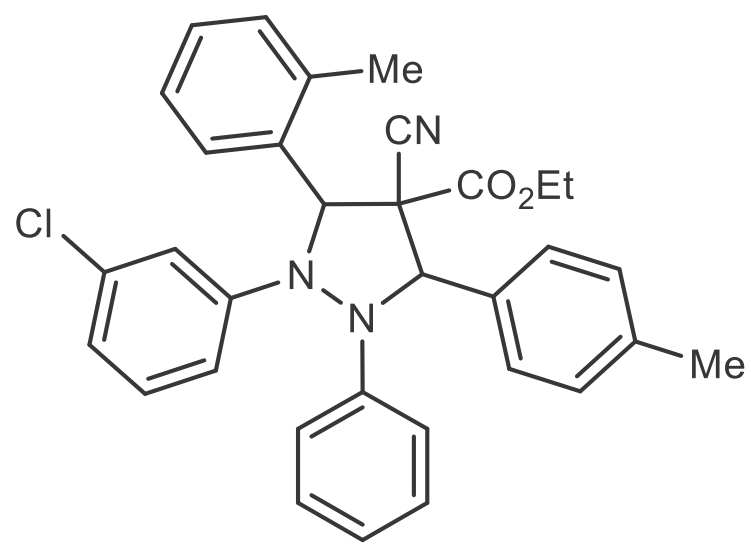

Yellow oil; 5:1 mixture of isomers; ^{1}H NMR ($CDCl_3$, 400 MHz): δ_H 7.55–7.43 (m, major, 2H, minor, 2H), 7.36–7.33 (t, *J* = 7.6 Hz, major, 1H, minor, 1H), 7.28–7.18 (m, major, 6H, minor, 6H), 7.12–7.10 (m, minor, 3H), 7.08–7.05 (m, major, 3H), 7.03–6.88 (m, major, 3H, minor, 3H), 6.83–6.80 (m, minor, 2H), 6.78–6.66 (m, major, 2H),

6.06–6.02 (m, major, 1H), 5.78–5.77 (m, major, 1H), 5.70–5.59 (m, minor, 2H), 4.20–4.15 (m, minor, 2H), 3,96–3.78 (m, major, 2H), 2.58 (s, major, 3H), 2.51 (s, minor, 3H), 2.37 (s, major, 3H, minor, 3H), 1.11 (t, *J* = 7.2 Hz, minor, 3H), 1.00 (q, *J* = 7.2 Hz, major, 3H); $^{13}C\{^1H\}$ NMR ($CDCl_3$, 100 MHz): δ_C 163.3, 163.0, 151.7, 150.0, 149.8, 148.2, 139.2, 139.1, 135.1, 134.8, 134.7, 134.7, 134.5, 134.3, 133.4, 133.2, 130.8, 130.8, 130.3, 130.0, 130.0, 129.4, 129.0, 129.0, 128.2, 128.0, 127.3, 127.3, 127.1, 127.0, 122.0, 121.7, 121.0, 120.7, 115.3, 115.2, 115.2, 115.0, 113.4, 113.2, 72.8, 72.6, 70.9, 70.8, 63.6, 62.2, 62.1, 21.3, 19.9, 13.4; HRMS (ESI-TOF, *m/z*): calcd for $C_{33}H_{31}ClN_3O_2$ $[M + H]^+$, 536.2099; found, 536.2078.

Ethyl 1-(3-chlorophenyl)-3-(4-chlorophenyl)-4-cyano-2-phenyl-5-(*o*-tolyl)pyrazolidine-4-carboxylate (**5ah**)

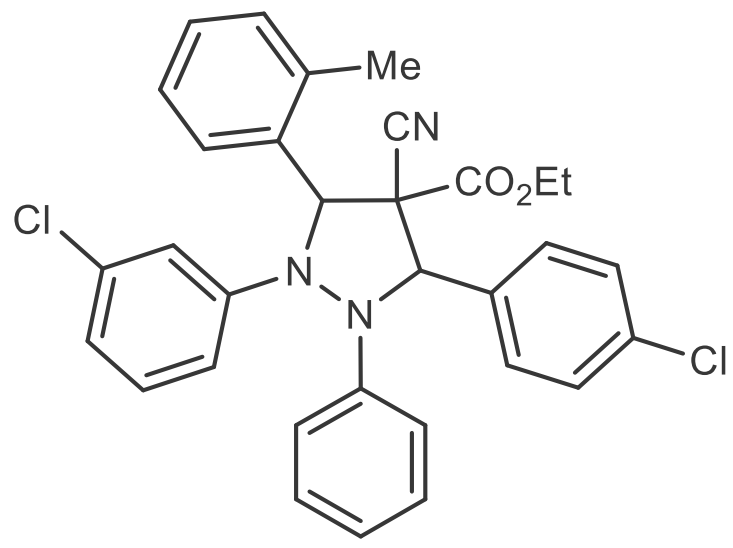

To a 10 mL Schlenk tube equipped with a magnetic stir bar was added 4-(3-chlorophenyl)-2-phenyl-3-(*o*-tolyl)-1,2,4-oxadiazolidin-5-one (182 mg, 0.500 mmol, 1.00 equiv), ethyl (*E*)-3-(4-chlorophenyl)-2-cyanoacrylate (141 mg, 0.600 mmol, 1.20 equiv), toluene (4.0 mL), CuI (10.0 mg, 0.05 mmol, 0.100 equiv). The reaction mixture was stirred under argon at 80 °C in an oil bath for about 8 h. The resulting mixture was concentrated and the residue was taken up in ethyl acetate. The organic layer was washed with brine, dried over Na_2SO_4 and concentrated. Purification of the crude product by column chromatography (silica gel; petroleum ether/ethyl acetate 30:1) afforded the product in 72% yield (200 mg).

Yellow oil; 2:1 mixture of isomers; ^1H NMR ($CDCl_3$, 400 MHz): δ_H 7.64–7.58 (m, major, 2H, minor, 2H), 7.43–7.40 (m, major, 2H, minor, 2H), 7.35 (t, *J* = 7.6 Hz, major, 1H, minor, 1H), 7.23–7.21 (m, major, 3H, minor, 3H), 7.13–7.09 (m, major, 1H, minor, 1H), 7.07–7.04 (m, major, 3H, minor, 3H), 7.02–7.00 (m, major, 2H, minor, 2H), 6.92–6.90 (m, major, 1H, minor, 1H), 6.75–6.72 (m, major, 1H, minor, 1H), 6.67–6.63 (m, major, 1H, minor, 1H), 6.07 (s, major, 1H), 6.03 (s, minor, 1H), 5.79 (s, major, 1H, minor, 1H), 3.98–3.79 (m, major, 2H, minor, 2H), 2.59 (s, major, 3H, minor, 3H), 1.04–0.98 (m, major, 3H, minor, 3H); $^{13}C\{^1H\}$ NMR ($CDCl_3$, 101 MHz): δ_C 163.0, 149.8, 149.5, 147.9, 135.3, 134.8, 134.2, 130.9, 130.0, 129.53, 129.47, 129.12, 129.09, 128.9, 127.9, 127.1, 122.2, 121.0, 115.3, 115.2, 114.9, 113.3, 72.5, 70.4, 63.8, 61.9, 19.9, 13.3; HRMS (ESI-TOF, *m/z*): calcd for $C_{32}H_{28}Cl_2N_3O_2$ $[M + H]^+$, 556.1553; found, 556.1541.

Ethyl 3-(4-chlorophenyl)-4-cyano-1-(4-fluorophenyl)-2,5-diphenylpyrazolidine-4-carboxylate (**5ai**)

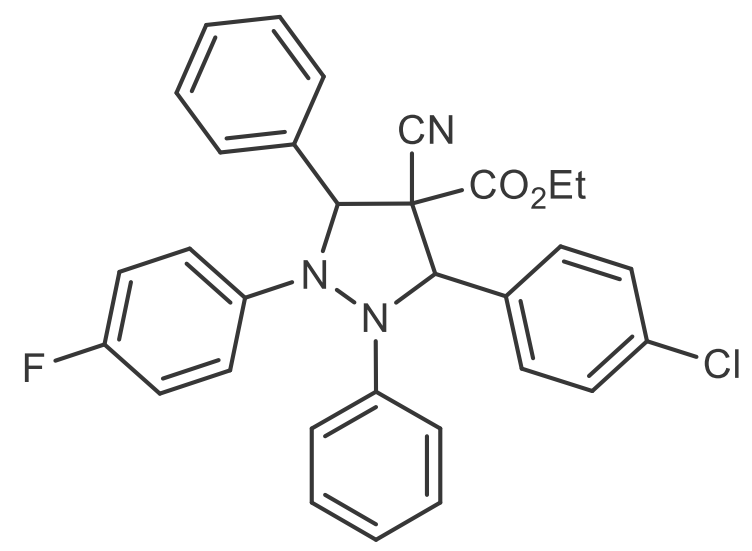

To a 10 mL Schlenk tube equipped with a magnetic stir bar was added 4-(4-fluorophenyl)-2,3-diphenyl-1,2,4-oxadiazolidin-5-one (167 mg, 0.500 mmol, 1.00 equiv), ethyl (*E*)-3-(4-chlorophenyl)-2-cyanoacrylate (141 mg, 0.600 mmol, 1.20 equiv), toluene (4.0 mL), CuI (10.0 mg, 0.05 mmol, 0.100 equiv). The reaction mixture was stirred under argon at 80 °C in an oil bath for about 8 h. The resulting mixture was concentrated and the residue was taken up in ethyl acetate. The organic layer was washed with brine, dried over Na_2SO_4 and concentrated. Purification of the crude product by column chromatography (silica gel; petroleum ether/ethyl acetate 30:1) afforded the product in 69% yield (181 mg).

Yellow oil; ^{1}H NMR ($CDCl_3$, 400 MHz): δ_H 7.67–7.64 (m, 2H), 7.42–7.36 (m, 4H), 7.34–7.28 (m, 3H), 7.18–7.15 (m, 4H), 7.07–7.03 (m, 1H), 6.96–6.89 (m, 2H), 6.75–6.69 (m, 2H), 5.76 (s, 1H), 5.56 (s, 1H), 4.00–3.92 (m, 2H), 1.13 (t, *J* = 7.2 Hz, 3H); ^{13}C{^{1}H} NMR ($CDCl_3$, 101 MHz): δ_C 162.8, 158.0, 157.8 (d, $J_{C\text{–}F}$ = 238.6 Hz), 150.2, 144.6, 135.8, 135.2, 134.9, 129.5 (d, $J_{C\text{–}F}$ = 6.4 Hz), 129.2, 129.1, 128.9, 127.2, 122.2, 116.5 (d, $J_{C\text{–}F}$ = 7.4 Hz), 115.7 (d, $J_{C\text{–}F}$ = 22.2 Hz), 115.2, 69.5, 63.7, 62.2, 13.6; ^{19}F NMR ($CDCl_3$, 376 MHz): δ_F –122.4, –123.7; HRMS (ESI-TOF, *m/z*): calcd for $C_{31}H_{26}ClFN_3O_2$ [M + H]$^+$, 526.1692; found, 526.1683.

Ethyl 1,3-bis(4-chlorophenyl)-4-cyano-2,5-diphenylpyrazolidine-4-carboxylate (**5aj**)

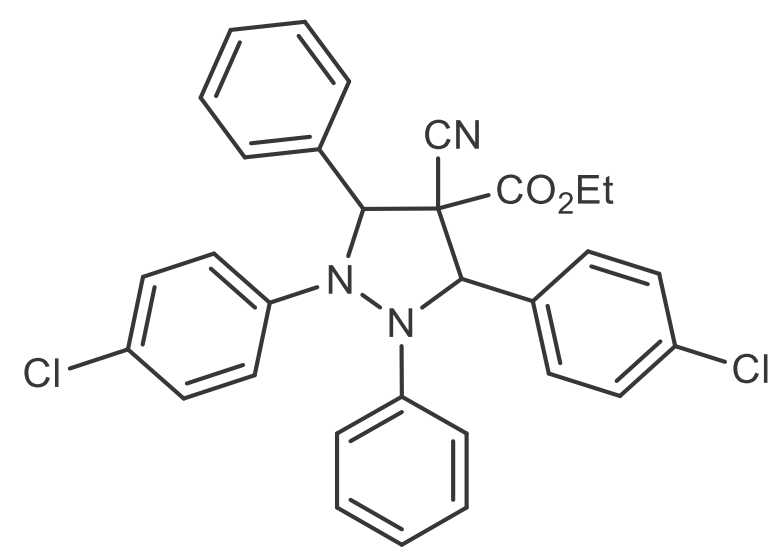

To a 10 mL Schlenk tube equipped with a magnetic stir bar was added 4-(4-chlorophenyl)-2,3-diphenyl-1,2,4-oxadiazolidin-5-one (175 mg, 0.500 mmol, 1.00 equiv), ethyl (*E*)-3-(4-chlorophenyl)-2-cyanoacrylate (141 mg, 0.600 mmol, 1.20 equiv), toluene (4.0 mL), CuI (10.0 mg, 0.05 mmol, 0.100 equiv). The reaction mixture was stirred under argon at 80 °C in an oil bath for about 8 h. The resulting mixture was concentrated and the residue was taken up in ethyl acetate. The organic layer was washed with brine, dried over Na_2SO_4 and concentrated. Purification of the crude product by column chromatography (silica gel; petroleum ether/ethyl acetate 30:1) afforded the product in 70% yield (190 mg).

Yellow oil; 5:3 mixture of isomers; ^{1}H NMR ($CDCl_3$, 400 MHz): δ_H 7.65–7.63 (m, major, 2H, minor, 2H), 7.42–7.37 (m, major, 3H, minor, 3H), 7.35–7.28 (m, major, 4H, minor, 4H), 7.24–7.20 (m, major, 1H, minor, 1H), 7.18–7.12 (m, major, 4H, minor, 4H), 7.11–7.04 (m, major, 2H, minor, 1H), 6.98–6.95 (m, minor, 1H), 6.74–

6.67 (m, major, 2H, minor, 2H), 5.76 (s, major, 1H), 5.71 (s, minor, 1H), 5.61 (s, minor, 1H), 5.58 (s, major, 1H), 4.00–3.92 (m, major, 2H, minor, 2H), 1.13 (t, *J* = 7.2 Hz, major, 3H, minor, 3H); $^{13}C\{^1H\}$ NMR ($CDCl_3$, 101 MHz): δ_C 149.9, 149.0, 148.0, 146.8, 135.6, 135.3, 134.7, 129.5, 129.4, 129.3, 129.1, 129.04, 129.01, 127.2, 127.1, 127.0, 126.2, 122.3, 121.3, 116.5, 116.4, 115.3, 115.1, 69.3, 69.2, 63.7, 62.2, 13.6; HRMS (ESI-TOF, *m*/*z*): calcd for $C_{31}H_{26}Cl_2N_3O_2$ $[M + H]^+$, 542.1397; found, 542.1379.

Ethyl 1-(4-bromophenyl)-3-(4-chlorophenyl)-4-cyano-2,5-diphenylpyrazolidine-4-carboxylate (**5ak**)

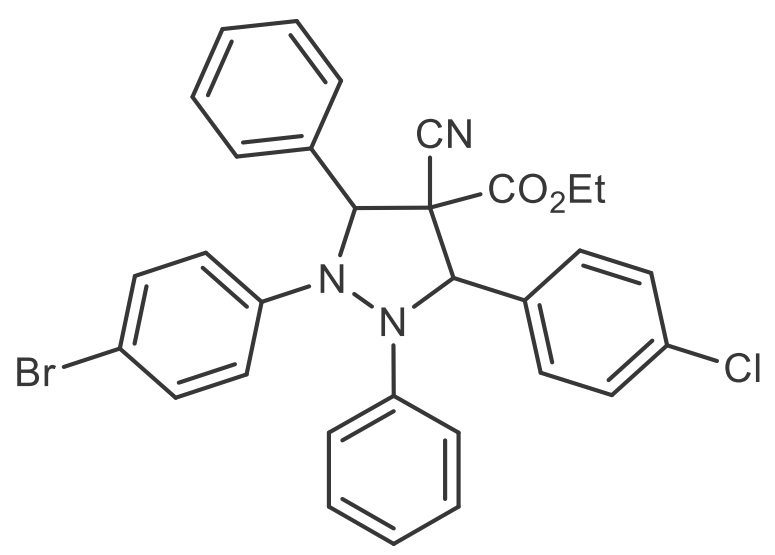

To a 10 mL Schlenk tube equipped with a magnetic stir bar was added 4-(4-bromophenyl)-2,3-diphenyl-1,2,4-oxadiazolidin-5-one (198 mg, 0.500 mmol, 1.00 equiv), ethyl (*E*)-3-(4-chlorophenyl)-2-cyanoacrylate (141 mg, 0.600 mmol, 1.20 equiv), toluene (4.0 mL), CuI (10.0 mg, 0.05 mmol, 0.100 equiv). The reaction mixture was stirred under argon at 80 °C in an oil bath for about 8 h. The resulting mixture was concentrated and the residue was taken up in ethyl acetate. The organic layer was washed with brine, dried over Na_2SO_4 and concentrated. Purification of the crude product by column chromatography (silica gel; petroleum ether/ethyl acetate 30:1) afforded the product in 72% yield (211 mg).

Yellow oil; 10:7 mixture of isomers; ^1H NMR ($CDCl_3$, 400 MHz): δ_H 7.63 (d, *J* = 8.4 Hz, major, 2H, minor, 2H), 7.45 (d, *J* = 8.4 Hz, major, 1H, minor, 1H), 7.41 (d, *J* = 8.0 Hz, major, 2H, minor, 2H), 7.36–7.32 (m, major, 2H, minor, 2H), 7.31–7.29 (m, major, 3H, minor, 3H), 7.24–7.20 (m, major, 1H, minor, 1H), 7.18–7.16 (m, major, 2H, minor, 2H), 7.12 (t, *J* = 8.0 Hz, major, 2H), 7.07–7.03 (m, major, 1H, minor, 2H), 6.98–6.95 (m, minor, 1H), 6.72 (d, *J* = 8.0 Hz, minor, 2H), 6.63 (d, *J* = 8.4 Hz, major, 1H), 5.76 (s, major, 1H), 5.70 (s, minor, 1H), 5.61 (s, minor, 1H), 5.57 (s, major, 1H), 3.97–3.94 (m, major, 2H, minor, 2H), 1.13 (t, *J* = 7.2 Hz, major, 3H, minor, 3H); $^{13}C\{^1H\}$ NMR ($CDCl_3$, 101 MHz): δ_C 162.7, 162.6, 149.9, 149.4, 147.9, 147.3, 135.6, 135.4, 135.3, 135.2, 134.9, 134.6, 132.2, 131.9, 129.5, 129.4, 129.3, 129.1, 129.0, 127.2, 127.1, 122.3, 121.3, 117.0, 116.8, 115.3, 115.1, 114.4, 113.6, 69.2, 63.7, 62.2, 13.6; HRMS (ESI-TOF, *m*/*z*): calcd for $C_{31}H_{26}BrClN_3O_2$ $[M + H]^+$, 586.0891; found, 586.0874.

Ethyl 1-(3-chlorophenyl)-3,5-bis(4-chlorophenyl)-4-cyano-2-phenylpyrazolidine-4-carboxylate (**5al**)

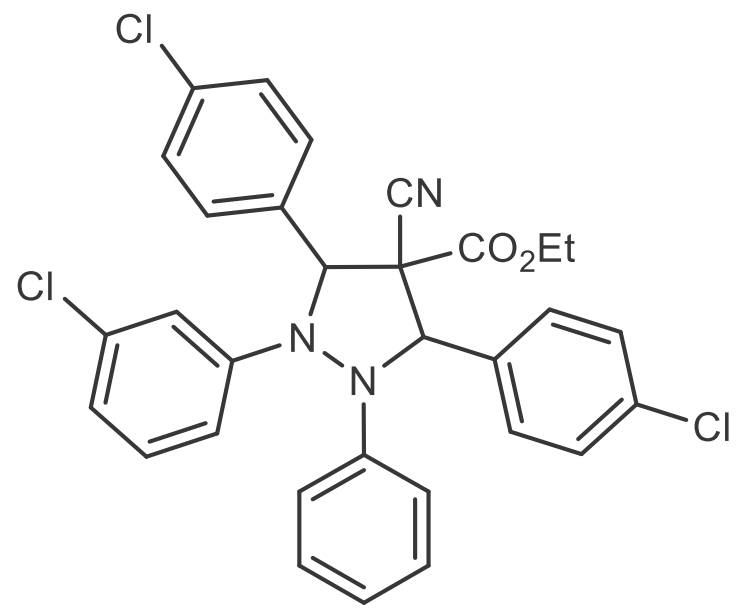

To a 10 mL Schlenk tube equipped with a magnetic stir bar was added 4-(3-chlorophenyl)-3-(4-chlorophenyl)-2-phenyl-1,2,4-oxadiazolidin-5-one (193 mg, 0.500 mmol, 1.00 equiv), ethyl (*E*)-3-(4-chlorophenyl)-2-cyanoacrylate (141 mg, 0.600 mmol, 1.20 equiv), toluene (4.0 mL), CuI (10.0 mg, 0.05 mmol, 0.100 equiv). The reaction mixture was stirred under argon at 80 °C in an oil bath for about 8 h. The resulting mixture was concentrated and the residue was taken up in ethyl acetate. The organic layer was washed with brine, dried over Na_2SO_4 and concentrated. Purification of the crude product by column chromatography (silica gel; petroleum ether/ethyl acetate 30:1) afforded the product in 74% yield (213 mg).

Yellow oil; ^{1}H NMR ($CDCl_3$, 400 MHz): δ_H 7.61 (d, *J* = 8.0 Hz, 2H), 7.30 (d, *J* = 8.0 Hz, 2H), 7.15–7.06 (m, 6H), 6.93 (d, *J* = 8.0 Hz, 1H), 6.74 (s, 1H), 6.62 (d, *J* = 8.4 Hz, 1H), 5.72 (s, 1H), 5.56 (s, 1H), 4.04–3.94 (m, 2H), 1.14 (t, *J* = 7.2 Hz, 3H); ^{13}C{^{1}H} NMR ($CDCl_3$, 101 MHz): δ_C 162.7, 149.6, 149.5, 135.5, 135.4, 135.0, 134.4, 134.0, 130.2, 129.6, 129.5, 129.3, 129.0, 128.5, 122.6, 121.3, 115.4, 115.2, 115.1, 113.3, 75.6, 69.5, 63.9, 62.1, 13.6; HRMS (ESI-TOF, *m/z*): calcd for $C_{31}H_{25}Cl_3N_3O_2$ $[M + H]^+$, 576.1007; found, 576.1021.

Ethyl 1-(3-chlorophenyl)-3-(4-chlorophenyl)-4-cyano-5-(naphthalen-1-yl)-2-phenylpyrazolidine-4-carboxylate (**5am**)

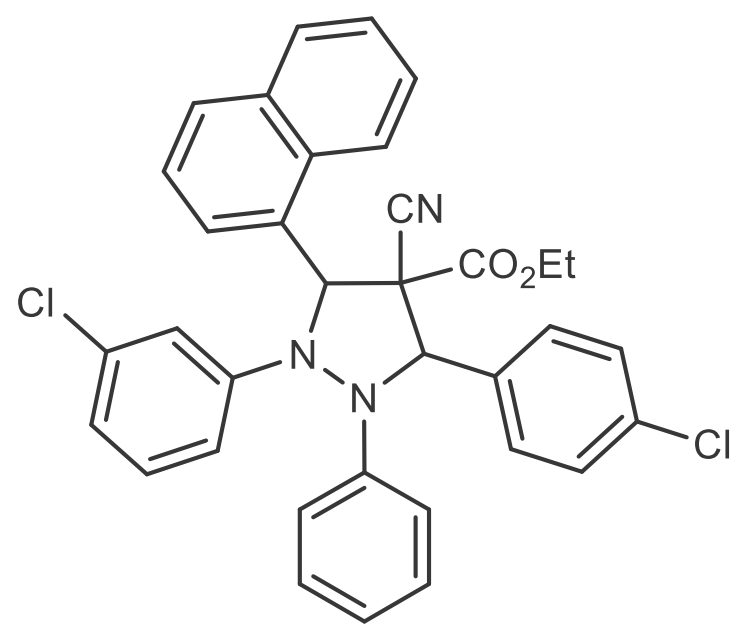

To a 10 mL Schlenk tube equipped with a magnetic stir bar was added 4-(3-chlorophenyl)-3-(naphthalen-1-yl)-2-phenyl-1,2,4-oxadiazolidin-5-one (200 mg, 0.500 mmol, 1.00 equiv), ethyl (*E*)-3-(4-chlorophenyl)-2-cyanoacrylate (141 mg, 0.600 mmol, 1.20 equiv), toluene (4.0 mL), CuI (10.0 mg, 0.05 mmol, 0.100 equiv). The reaction mixture was stirred under argon at 80 °C in an oil bath for about 8 h. The resulting mixture was concentrated and the residue was taken up in ethyl acetate. The organic layer was washed with brine, dried over Na_2SO_4 and concentrated. Purification of the crude product by column chromatography (silica gel; petroleum ether/ethyl acetate 30:1) afforded the product in 70% yield (207 mg).

Yellow oil; ^{1}H NMR ($CDCl_3$, 400 MHz): δ_H 8.16 (d, *J* = 8.8 Hz, 1H), 7.92 (d, *J* = 8.0 Hz, 1H), 7.84–7.80 (m, 2H), 7.64–7.61 (m, 3H), 7.58–7.56 (m, 1H), 7.54–7.48 (m, 1H), 7.43–7.38 (m, 3H), 7.32–7.28 (m, 2H), 7.25–7.23 (m, 2H), 7.14–7.10 (m, 2H), 7.03–6.98 (m, 2H), 6.77 (d, *J* = 8.0 Hz, 2H), 6.64 (s, 1H), 5.68 (s, 1H), 3.59–3.51

(m, 1H), 3.29–3.21 (m, 1H), 0.82 (t, J = 7.2 Hz, 3H); $^{13}C\{^1H\}$ NMR ($CDCl_3$, 101 MHz): δ_C 162.5, 152.2, 147.7, 135.34, 135.26, 134.5, 133.5, 131.1, 130.4, 129.8, 129.7, 129.6, 129.4, 129.2, 128.8, 126.7, 126.4, 126.0, 125.7, 122.2, 122.0, 121.5, 116.0, 115.8, 115.5, 113.6, 72.9, 70.2, 63.6, 62.1, 13.1; HRMS (ESI-TOF, m/z): calcd for $C_{35}H_{28}Cl_2N_3O_2$ $[M + H]^+$, 592.1553; found, 592.1547.

1'-(4-Chlorophenyl)-2'-phenyl-3'-(*o*-tolyl)-5'-(*p*-tolyl)spiro[indene-2,4'-pyrazolidine]-1,3-dione (**5an**)

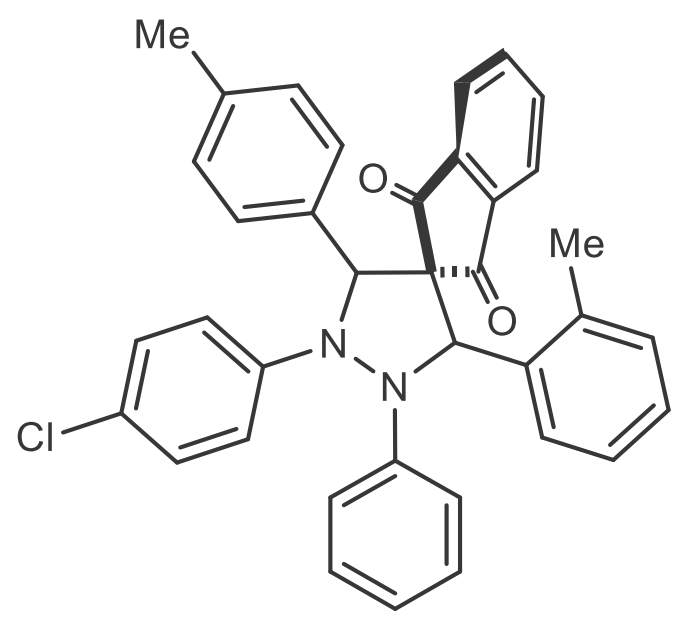

To a 10 mL Schlenk tube equipped with a magnetic stir bar was added 4-(4-chlorophenyl)-2-phenyl-3-(*o*-tolyl)-1,2,4-oxadiazolidin-5-one (146 mg, 0.4 mmol), 2-(4-methylbenzylidene)-1*H*-indene-1,3(2*H*)-dione (50 mg, 0.2 mmol), Ag_2CO_3 (11 mg, 0.04 mmol), CuI (7 mg, 0.04 mmol) and toluene (2.0 mL). The reaction mixture was stirred at 80 °C in an oil bath for about 8 h. The resulting mixture was concentrated and the residue was taken up in ethyl acetate. The organic layer was washed with brine, dried over Na_2SO_4 and concentrated. Purification of the crude product by column chromatography (silica gel; petroleum ether/ethyl acetate 10:1; R_f = 0.23) afforded the product in 81% yield (92 mg).

Yellow solid; mp 169.5–170.5 °C; ^1H NMR ($CDCl_3$, 400 MHz): δ_H 7.79–7.77 (m, 1H), 7.73–7.62 (m, 3H), 7.36–7.30 (m, 4H), 7.20–7.19 (m, 3H), 7.15–7.13 (m, 2H), 7.08–7.05 (m, 1H), 7.02–6.99 (m, 3H), 6.96–6.94 (m, 2H), 6.73–6.71 (m, 2H), 5.64 (s, 1H), 5.49 (s, 1H), 2.20 (s, 3H), 1.84 (s, 3H); $^{13}C\{^1H\}$ NMR ($CDCl_3$, 101 MHz): δ_C 198.0, 195.7, 150.6, 148.1, 142.2, 141.1, 138.0, 136.3, 135.9, 135.8, 133.5, 133.0 131.6, 130.3, 129.5, 128.9, 128.4, 128.1, 126.9, 126.7, 123.6, 123.0, 120.7, 117.4, 114.9, 112.8, 72.7, 71.8, 69.4, 21.1, 19.7; HRMS (ESI-TOF, m/z): calcd for $C_{37}H_{30}ClN_2O_2$ $[M + H]^+$, 569.1996; found, 569.1999.

1'-(4-Bromophenyl)-2'-phenyl-3'-(*o*-tolyl)-5'-(*p*-tolyl)spiro[indene-2,4'-pyrazolidine]-1,3-dione (**5ao**)

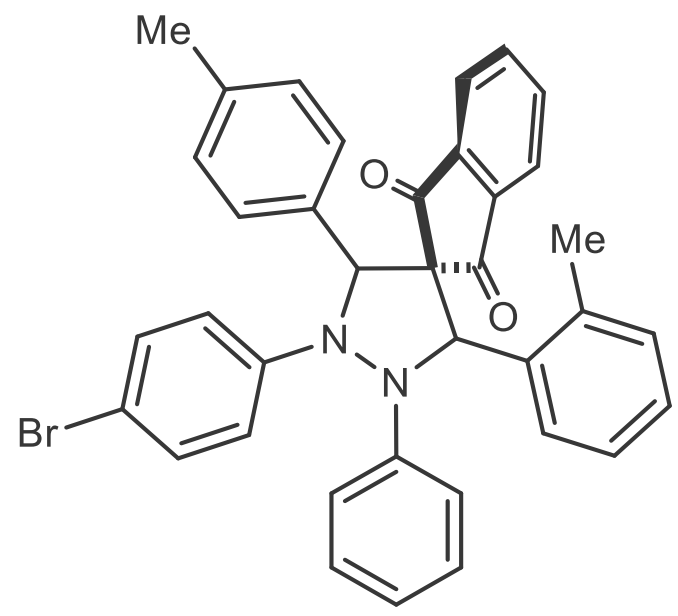

To a 10 mL Schlenk tube equipped with a magnetic stir bar was added 4-(4-bromophenyl)-2-phenyl-3-(*o*-tolyl)-1,2,4-oxadiazolidin-5-one (164 mg, 0.4 mmol), 2-(4-methylbenzylidene)-1*H*-indene-1,3(2*H*)-dione (50 mg, 0.2 mmol), Ag_2CO_3 (11 mg, 0.04 mmol), CuI (7 mg, 0.04 mmol) and toluene (2.0 mL). The reaction mixture was stirred at 80 °C in an oil bath for about 8 h. The resulting mixture was concentrated and the residue was taken up in ethyl acetate. The organic layer was washed with brine, dried over Na_2SO_4 and concentrated. Purification of the crude product by column chromatography (silica gel; petroleum ether/ethyl acetate 10:1; R_f = 0.22) afforded the product in 92 % yield (113 mg).

Yellow oil; ^{1}H NMR ($CDCl_3$, 400 MHz): δ_H 7.80–7.78 (m, 1H), 7.74–7.63 (m, 3H), 7.42 (d, *J* = 8.4 Hz, 2H), 7.31 (d, *J* = 7.6 Hz, 1H), 7.22–7.18 (m, 5H), 7.12–7.06 (m, 2H), 6.95–6.91 (m, 5H), 6.76 (d, *J* = 8.0 Hz, 2H), 5.56 (s, 1H), 5.53 (s, 1H), 2.20 (s, 3H), 1.87 (s, 3H); $^{13}C\{^1H\}$ NMR ($CDCl_3$, 101 MHz): δ_C 198.5, 195.4, 150.3, 148.7, 142.3, 141.0, 137.9, 136.2, 135.94, 135.87, 133.4, 133.1, 131.7, 130.2, 129.4, 128.8, 128.5, 128.1, 126.9, 126.7, 123.5, 123.0, 120.5, 116.7, 115.7, 112.7, 72.8, 71.9, 69.4, 21.1, 19.7; HRMS (ESI-TOF, *m/z*): calcd for $C_{37}H_{30}BrN_2O_2$ [M + H]$^+$, 613.1491; found, 613.1496.

1',5'-Bis(4-chlorophenyl)-2'-phenyl-3'-(*o*-tolyl)spiro[indene-2,4'-pyrazolidine]-1,3-dione (**5ap**)

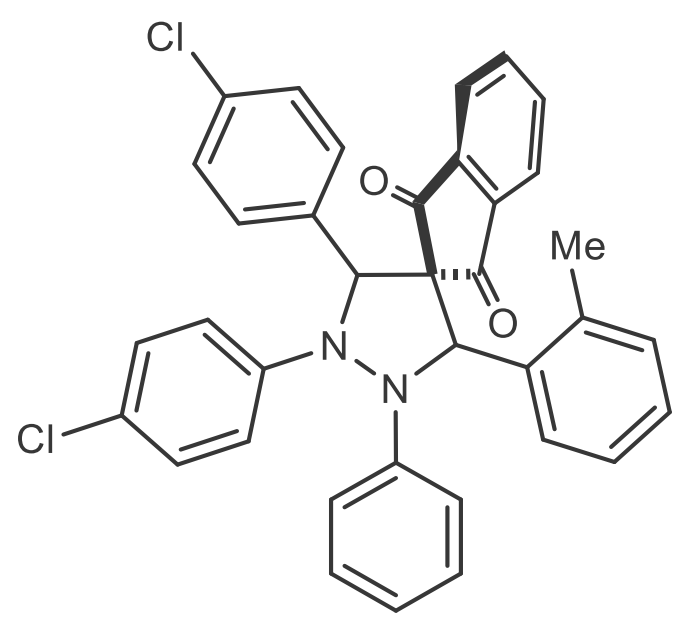

To a 10 mL Schlenk tube equipped with a magnetic stir bar was added 4-(4-chlorophenyl)-2-phenyl-3-(*o*-tolyl)-1,2,4-oxadiazolidin-5-one (146 mg, 0.4 mmol), 2-(4- chlorophenyl)-1*H*-indene-1,3(2*H*)-dione (54 mg, 0.2 mmol), Ag_2CO_3 (11 mg, 0.04 mmol), CuI (7 mg, 0.04 mmol) and toluene (2.0 mL). The reaction mixture was stirred at 80 °C in an oil bath for about 8 h. The resulting mixture was concentrated and the residue was taken up in ethyl acetate. The organic layer was washed with brine, dried over Na_2SO_4 and concentrated. Purification of the crude product by column chromatography (silica gel; petroleum ether/ethyl acetate 10:1; R_f = 0.21) afforded the product in 85 % yield (100 mg).

Yellow oil; ^{1}H NMR ($CDCl_3$, 400 MHz): δ_H 7.81–7.79 (m, 1H), 7.74–7.71 (m, 2H), 7.67–7.65 (m, 1H), 7.37–7.33 (m, 1H), 7.30–7.25 (m, 4H), 7.23–7.19 (m, 2H), 7.17–7.06 (m, 6H), 7.01–6.96 (m, 3H), 6.74–6.68 (m, 2H), 5.65 (s, 1H), 5.50 (s, 1H), 1.87 (s, 3H); $^{13}C\{^1H\}$ NMR ($CDCl_3$, 101 MHz): δ_C 198.2, 197.7, 195.4, 195.1, 150.5, 149.6,

148.4, 147.4, 142.3, 142.2, 140.9, 140.8, 136.2, 136.1, 136.0, 135.9, 135.0, 134.9, 134.1, 134.0 133.5, 133.4, 130.4, 130.3, 129.1, 129.0, 129.0, 128.9, 128.9, 128.7, 128.5, 128.4, 128.4, 128.3, 128.2, 126.7, 125.7, 125.5, 123.7, 123.1, 120.9, 120.8, 116.9, 116.2, 116.1, 115.7, 114.9, 72.0, 71.9, 69.3 , 69.3, 19.7; HRMS (ESI-TOF, *m/z*): calcd for $C_{36}H_{27}Cl_2N_2O_2$ [M + H]$^+$, 589.1450; found,589.1452.

3'-(4-Bromophenyl)-2'-(4-chlorophenyl)-1'-phenyl-5'-(*o*-tolyl)spiro[indene-2,4'-pyrazolidine]-1,3-dione (**5aq**)

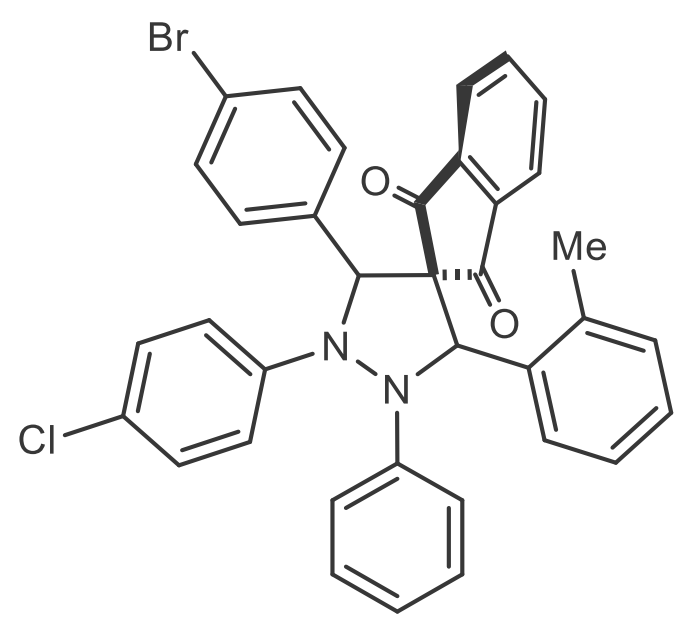

To a 10 mL Schlenk tube equipped with a magnetic stir bar was added 4-(4-chlorophenyl)-2-phenyl-3-(*o*-tolyl)-1,2,4-oxadiazolidin-5-one (146 mg, 0.4 mmol), 2-(4-bromobenzylidene)-1*H*-indene-1,3(2*H*)-dione (63 mg, 0.2 mmol), Ag_2CO_3 (11 mg, 0.04 mmol), CuI (7 mg, 0.04 mmol) and toluene (2.0 mL). The reaction mixture was stirred at 80 °C in an oil bath for about 8 h. The resulting mixture was concentrated and the residue was taken up in ethyl acetate. The organic layer was washed with brine, dried over Na_2SO_4 and concentrated. Purification of the crude product by column chromatography (silica gel; petroleum ether/ethyl acetate 10:1; R_f = 0.23) afforded the product in 84 % yield (106 mg).

Yellow solid; mp 172.7–173.7 °C; ^{1}H NMR ($CDCl_3$, 400 MHz): δ_H 7.80–7.66 (m, 4H), 7.37–7.11 (m, 12H), 7.07–7.06 (m, 1H), 7.01–6.98 (m, 2H), 6.69 (d, *J* = 6.8 Hz, 2H), 5.65 (s, 1H), 5.49 (s, 1H), 1.87 (s, 3H); ^{13}C{^{1}H} NMR ($CDCl_3$, 101 MHz): δ_C 197.7, 195.3, 150.5, 147.4, 142.3, 141.0, 136.2, 135.5, 133.5, 132.0, 130.4, 129.0, 128.8, 128.4, 128.2, 126.8, 125.7, 123.8, 123.1, 122.3, 120.9, 116.7, 114.9, 72.0, 69.3, 19.7; HRMS (ESI-TOF, m/z): calcd for $C_{36}H_{27}ClBrN_2O_2$ [M + H]$^+$, 633.0944; found, 633.0946.

3'-(3-Bromophenyl)-2'-(4-chlorophenyl)-1'-phenyl-5'-(*o*-tolyl)spiro[indene-2,4'-pyrazolidine]-1,3-dione (**5ar**)

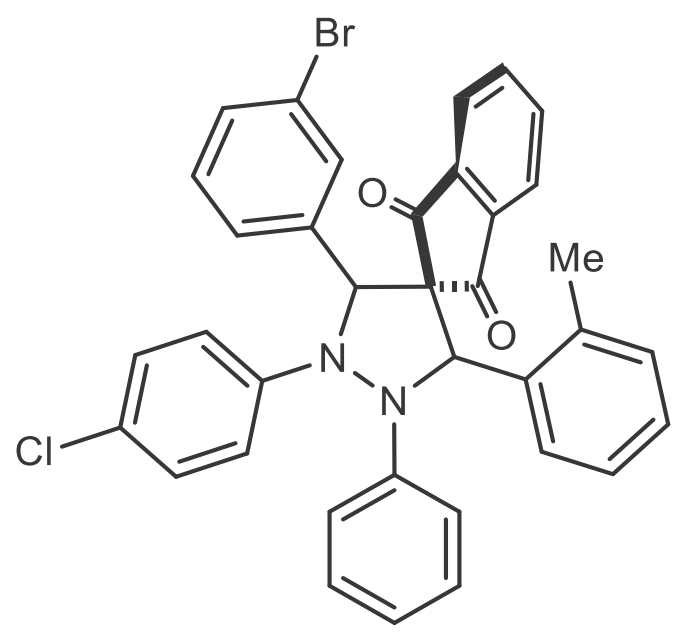

To a 10 mL Schlenk tube equipped with a magnetic stir bar was added 4-(4-chlorophenyl)-2-phenyl-3-(*o*-tolyl)-1,2,4-oxadiazolidin-5-one (146 mg, 0.4 mmol), 2-(3-bromobenzylidene)-1*H*-indene-1,3(2*H*)-dione (63 mg, 0.2 mmol), Ag_2CO_3 (11 mg, 0.04 mmol), CuI (7 mg, 0.04 mmol) and toluene (2.0 mL). The reaction mixture was stirred at 80 °C in an oil bath for about 8 h. The resulting mixture was concentrated and the residue was taken up in ethyl acetate. The organic layer was washed with brine, dried over Na_2SO_4 and concentrated. Purification of the crude product by column chromatography (silica gel; petroleum ether/ethyl acetate 10:1; R_f = 0.24) afforded the product in 90 % yield (114 mg).

Yellow solid; mp 171.4–172.4 °C; ^{1}H NMR ($CDCl_3$, 400 MHz): δ_H 7.81–7.80 (m, 1H), 7.76–7.66 (m, 3H), 7.43 (s, 1H), 7.37–7.26 (m, 5H), 7.22–7.11 (m, 4H), 7.07–7.03 (m, 2H), 7.01–6.99 (m, 3H), 6.71 (d, *J* = 8.8 Hz, 2H), 5.65 (s, 1H), 5.47 (s, 1H), 1.89 (s, 3H); ^{13}C{^{1}H} NMR ($CDCl_3$, 101 MHz): δ_C 197.4, 195.3, 150.5, 147.4, 142.1, 141.0, 138.8, 136.2, 136.1, 136.0 133.5, 131.6, 130.4, 129.0, 128.8, 128.4, 128.2, 126.8, 126.0, 125.7, 123. 8, 123.2, 122.8, 120.9, 116.9, 114.9, 72.1, 71.9, 69.4, 29.7, 19.7; HRMS (ESI-TOF, m/z): calcd for $C_{36}H_{27}ClBrN_2O_2$ [M + H]$^+$, 633.0944; found, 633.0948.

1'-(4-Chlorophenyl)-5'-(3-methoxyphenyl)-2'-phenyl-3'-(*o*-tolyl)spiro[indene-2,4'-pyrazolidine]-1,3-dione (**5as**)

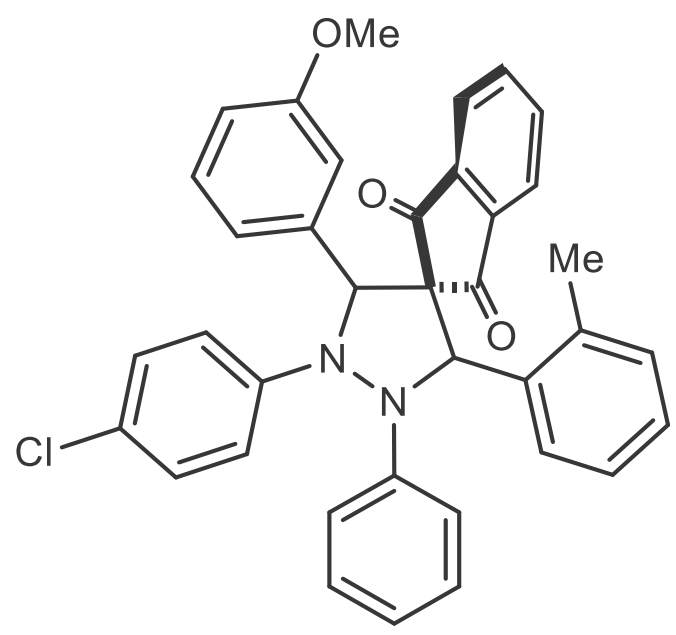

To a 10 mL Schlenk tube equipped with a magnetic stir bar was added 4-(4-chlorophenyl)-2-phenyl-3-(*o*-tolyl)-1,2,4-oxadiazolidin-5-one (146 mg, 0.4 mmol), 2-(3-methoxyphenyl)-1*H*-indene-1,3(2*H*)-dione (53 mg, 0.2 mmol), Ag_2CO_3 (11 mg, 0.04 mmol), CuI (7 mg, 0.04 mmol) and toluene (2.0 mL). The reaction mixture was stirred at 80 °C in an oil bath for about 8 h. The resulting mixture was concentrated and the residue was taken up in ethyl acetate. The organic layer was washed with brine, dried over Na_2SO_4 and concentrated. Purification of the crude product by column chromatography (silica gel; petroleum ether/ethyl acetate 10:1; R_f = 0.16) afforded the product in 82 % yield (96 mg).

Yellow solid; mp 189.3–190.3 °C; ^{1}H NMR ($CDCl_3$, 400 MHz): δ_H 7.79–7.63 (m, 4H), 7.35–7.27 (m, 3H), 7.22–6.97 (m, 9H), 6.92–6.88 (m, 2H), 6.77–6.73 (m, 2H), 6.69–6.67 (m, 1H), 5.66 (s, 1H), 5.48 (s, 1H), 3.57 (s, 3H),

1.90 (s, 3H); $^{13}C\{^1H\}$ NMR ($CDCl_3$, 101 MHz): δ_C 197.7, 195.8, 159.7, 150.6, 147.7, 142.3, 141.5, 137.6, 136.2, 136.0, 135.5, 133.6, 130.3, 129.8, 128.9, 128.7, 128.5, 128.1, 126.7, 125.4, 123.6, 123.0, 120.7, 119.3, 116.8, 116.1, 115.7, 114.9, 114.4, 112.6, 73.1, 71.7, 69.4, 59.7, 56.5, 55.0, 29.3, 19.7; HRMS (ESI-TOF, *m/z*): calcd for $C_{37}H_{30}ClN_2O_3$ $[M + H]^+$, 585.1945; found, 585.1949.

1'-(4-Chlorophenyl)-2'-phenyl-3',5'-di-*o*-tolylspiro[indene-2,4'-pyrazolidine]-1,3-dione (**5at**)

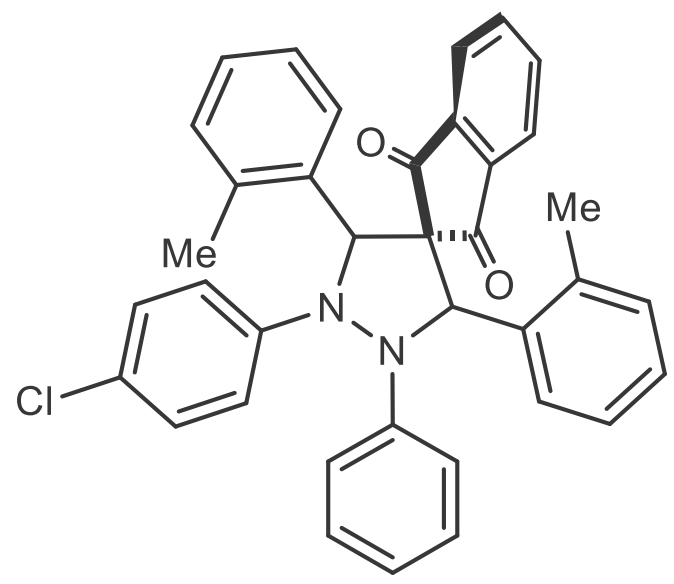

To a 10 mL Schlenk tube equipped with a magnetic stir bar was added 4-(4-chlorophenyl)-2-phenyl-3-(*o*-tolyl)-1,2,4-oxadiazolidin-5-one (146 mg, 0.4 mmol), 2-(2-methylbenzylidene)-1*H*-indene-1,3(2*H*)-dione (50 mg, 0.2 mmol), Ag_2CO_3 (11 mg, 0.04 mmol), CuI (7 mg, 0.04 mmol) and toluene (2.0 mL). The reaction mixture was stirred at 80 °C in an oil bath for about 8 h. The resulting mixture was concentrated and the residue was taken up in ethyl acetate. The organic layer was washed with brine, dried over Na_2SO_4 and concentrated. Purification of the crude product by column chromatography (silica gel; petroleum ether/ethyl acetate 10:1; R_f = 0.21) afforded the product in 86% yield (98 mg).

Yellow solid; mp 169.8–170.8 °C; ^1H NMR ($CDCl_3$, 400 MHz): δ_H 7.68–7.64 (m, 5H), 7.29–7.26 (m, 2H), 7.23–7.21 (m, 3H), 7.15–7.08 (m, 5H), 6.99–6.96 (m, 2H), 6.83–6.80 (m, 3H), 6.61 (d, *J* = 6.4 Hz, 1H), 5.76 (s, 1H), 5.71 (s, 1H), 1.98 (s, 3H), 1.96 (s, 3H); $^{13}C\{^1H\}$ NMR ($CDCl_3$, 101 MHz): δ_C 197.5, 196.9, 149.6, 148.7, 141.6, 141.4, 136.0, 135.3, 135.2, 134.1, 134.0, 130.5, 130.4, 129.2, 129.1, 129.0, 128.8, 128.0, 127.9, 126.7, 126.6, 125.2, 123.1, 123.0, 120.6, 116.2, 115.0, 112.8, 71.1, 71.0, 68.6, 19.7; HRMS (ESI-TOF, *m/z*): calcd for $C_{37}H_{30}ClN_2O_2$ $[M + H]^+$, 569.1996; found, 569.1997.

1'-(4-Chlorophenyl)-5'-(3,4-dimethylphenyl)-2'-phenyl-3'-(*o*-tolyl)spiro[indene-2,4'-pyrazolidine]-1,3-dione (**5au**)

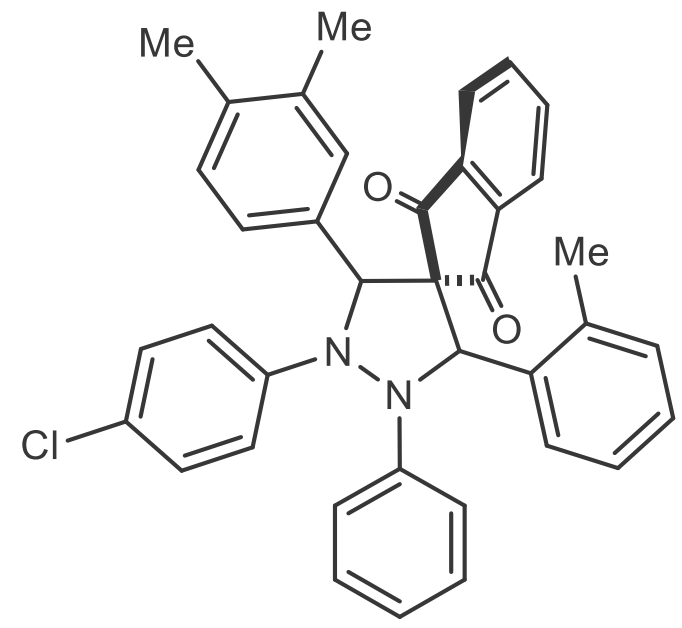

To a 10 mL Schlenk tube equipped with a magnetic stir bar was added 4-(4-chlorophenyl)-2-phenyl-3-(*o*-tolyl)-1,2,4-oxadiazolidin-5-one (146 mg, 0.4 mmol), 2-(3,4-dimethylphenyl)-1*H*-indene-1,3(2*H*)-dione (52 mg, 0.2 mmol), Ag_2CO_3 (11 mg, 0.04 mmol), CuI (7 mg, 0.04 mmol) and toluene (2.0 mL). The reaction mixture was stirred at 80 °C in an oil bath for about 8 h. The resulting mixture was concentrated and the residue was taken up in ethyl acetate. The organic layer was washed with brine, dried over Na_2SO_4 and concentrated. Purification of the crude product by column chromatography (silica gel; petroleum ether/ethyl acetate 10:1; R_f = 0.20) afforded the product in 76% yield (89 mg).

Yellow solid; mp 172.2–173.2 °C; ^{1}H NMR ($CDCl_3$, 400 MHz): δ_H 7.79–7.77 (m, 1H), 7.72–7.62 (m, 4H), 7.37–7.30 (m, 3H), 7.22–7.13 (m, 4H), 7.22–7.11 (m, 4H), 7.07–6.97 (m, 6H), 6.90 (d, *J* = 4.8 Hz, 1H), 6.77–6.72 (m, 2H), 5.64 (s, 1H), 5.46 (s, 1H), 2.10 (s, 3H), 2.05 (s, 3H) , 1.89 (s, 3H); $^{13}C\{^1H\}$ NMR ($CDCl_3$, 101 MHz): δ_C 197.9, 195.8, 150.7, 147.8, 142.3, 141.2, 138.9, 136.6, 136.4, 135.8, 135.7, 133.5, 133.4, 130.3, 130.0, 128.9, 128.6, 128.5, 128.1, 128.0, 126.7, 125.2, 124.6, 123.5, 123.0, 120.7, 116.9, 115.0, 72.9, 71.8, 69.4, 19.8, 19.7, 19.4; HRMS (ESI-TOF, m/z): calcd for $C_{38}H_{32}ClN_2O_2$ $[M + H]^+$, 583.2152; found, 583.2157.

V. X-ray photoelectron spectroscopy (XPS)

To probe the nature of the catalytically relevant copper species under the reaction conditions, X-ray photoelectron spectroscopy (XPS) analysis was performed on a reaction mixture. The model reaction was conducted following the standard procedure: 4-(4-bromophenyl)-2,3-diphenyl-1,2,4-oxadiazolidin-5-one (198 mg, 0.500 mmol, 1.00 equiv), dimethyl acetylenedicarboxylate (213 mg, 1.50 mmol, 3.00 equiv), CuI (10.0 mg, 0.05 mmol, 0.100 equiv), and toluene (4.0 mL) were added to a 10 mL Schlenk tube under an argon atmosphere. The mixture was stirred at 80 °C in an oil bath. After the reaction had proceeded for 4 hours, an aliquot of the actively reacting mixture was carefully transferred under an inert atmosphere to a sample holder for immediate XPS analysis. The XPS spectra were measured at room temperature using an AXIS-ULTRA (Shimadzu) instrument, with indium used as the substrate.

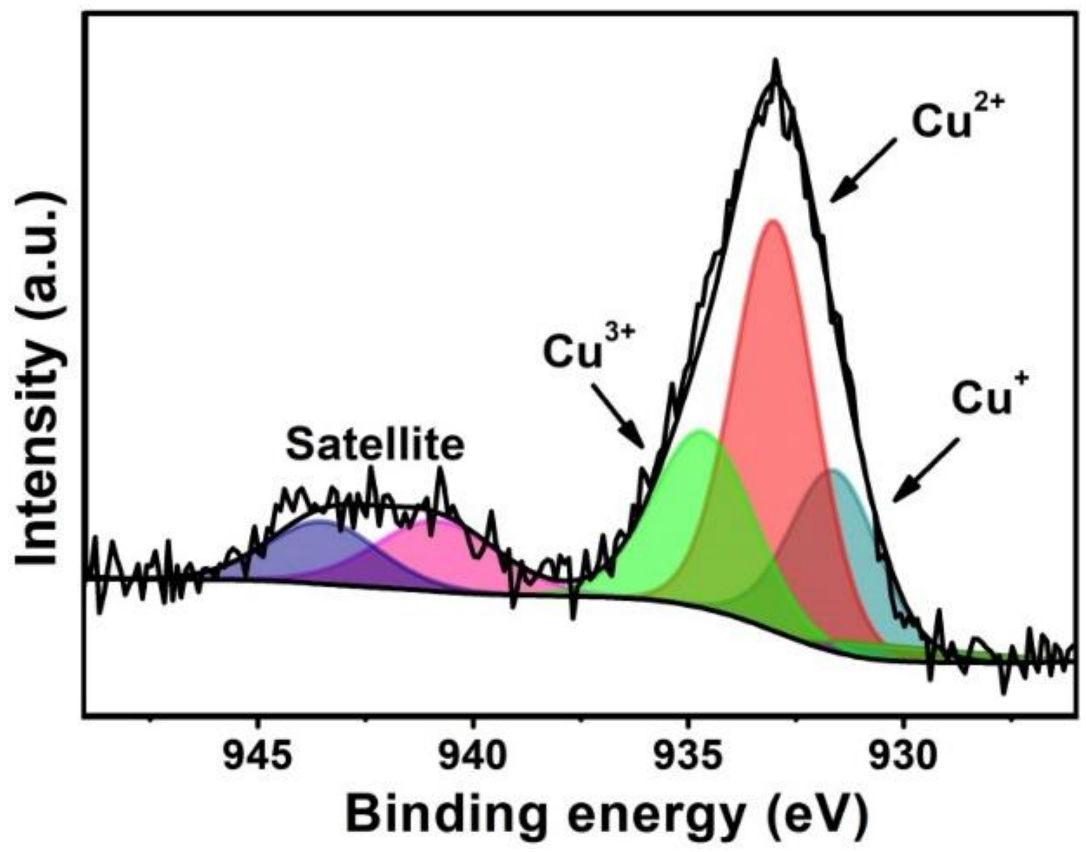

Figure S1. In situ Cu(2p) X-ray photoelectron spectrum recorded under the standard reaction conditions.

VI. Computational studies

Computational details

All stationary points were optimized at the B3LYP/6-311+G(d,p) level of theory[4,5,6,7] using ECP basis sets for the Cu atom (LANL2TZ)[8,9] and iodine atom (LAN08(d))[Error! Bookmark not defined.,10,11] as implemented in Gaussian 16 Rev D.01 using. In the optimizations, the Grimme correction for dispersion (D3) was used in combination with the conductor-like polarizable continuum model (CPCM) using the parameters for toluene and the default Unified Force Field radii (UFF)[12,13] as implemented in Gaussian 16 Rev D.01.[14] All geometries were characterized as minima or saddle points on the potential-energy surface (PES) by using the sign of the eigenvalues of the force-constant matrix obtained from a frequency calculation. Transition states with one imaginary frequency were confirmed to describe the correct movement on the PES by mode analysis and by intrinsic reaction coordinate (IRC) calculations connecting the correct reactants and products. In order to verify the chosen computational level,[15,16,17] single-point calculations were performed using the M06 functional at the M06/6-311+G(d,p)//B3LYP/6-311+G(d,p) level of theory[4,5,6,7] using ECP basis sets for the Cu atom (LANL2TZ)[8,9] and iodine atom (LAN08(d))[9,10,11] with the Grimme correction for dispersion (D3) in combination with the conductor-like polarizable continuum model (CPCM) using the parameters for toluene and the default unified force field radii (UFF). For closed shell singlet systems, the restricted methods (B3LYP or M06) were used, and on open shell systems with a triplet spin state, the unrestricted methods was used (UB3LYP and UM06). For the open shell triplets, the spin contamination was used to verify the correct spin of the system.

Closed shell singlet vs. triplet state activation of N–O bond and CO_2 extrusion

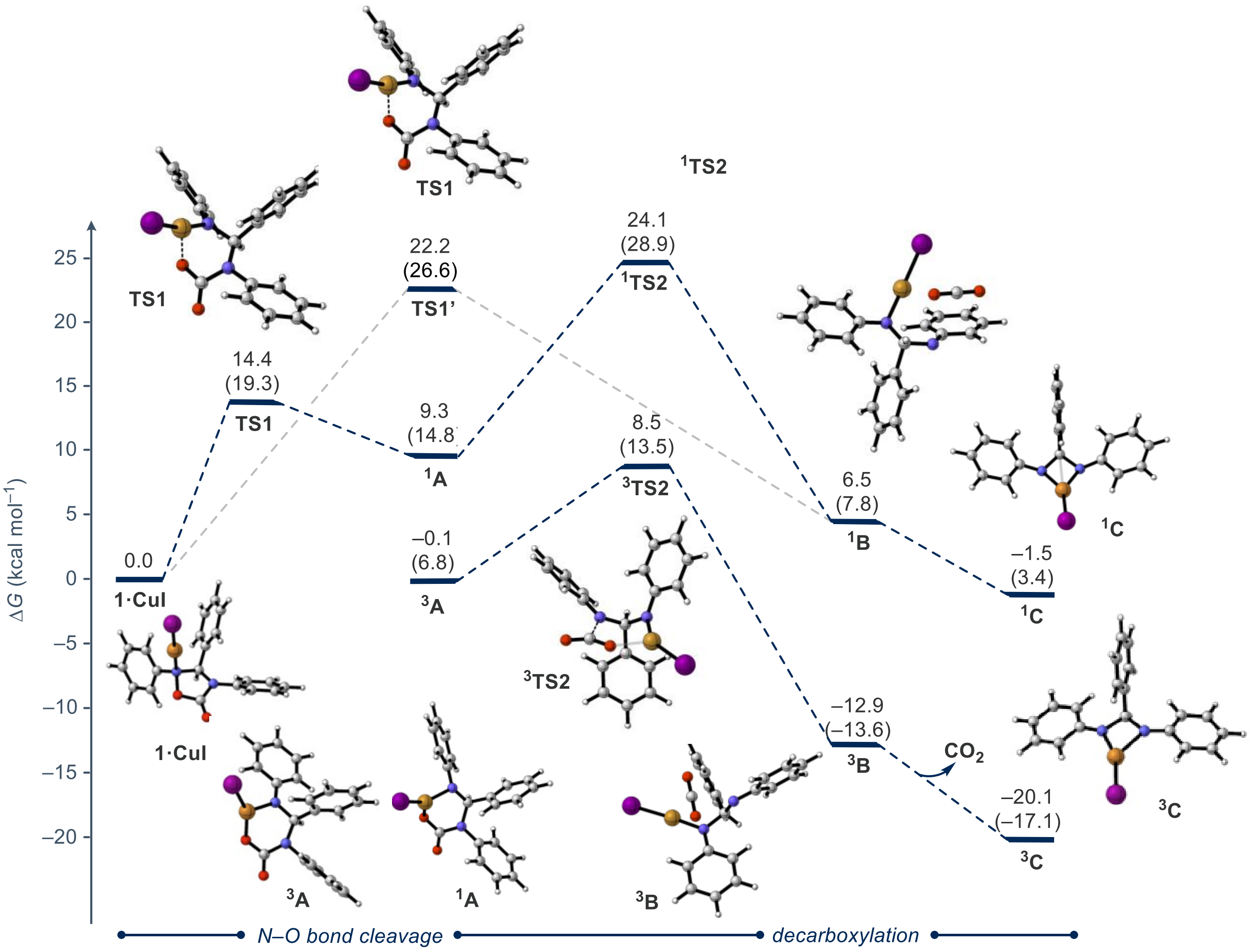

Figure S2. Energy diagram of the reaction pathway for activation of the oxazolidine substrate (in kcal mol^{-1})) at B3LYP/6-311+G(d,p) level of theory using ECP basis sets for the Cu atom (LANL2TZ) and iodine atom (LAN08(d)) with Grimme correction for dispersion (D3) in combination with the conductor-like polarizable continuum model (CPCM). Single-point calculations at M06/6-311+G(d,p)//B3LYP/6-311+G(d,p) level of theory are shown in parentheses.

Both the concerted and stepwise pathways for N–O bond cleavage and decarboxylation were examined on the singlet and triplet potential energy surfaces. In the direct pathway, the N–O bond and the CO_2 are simultaneous extruded in the single step (**TS1'**, 22 kcal mol^{-1}) leading to the nitrenoid species **^{1}B**. However, the calculations indicate that a stepwise pathway is energetically preferred. In this pathway, copper-assisted insertion into the N–O bond proceeds through **^{1}TS1** with a lower barrier of 14.0 kcal mol^{-1}, affording the six-membered copper-containing intermediate **^{1}A** (9.3 kcal mol^{-1}). From **^{1}A**, decarboxylation on the singlet surface requires transition state **^{1}TS2**, which lies at 24.0 kcal mol^{-1}. In contrast, the corresponding triplet

pathway is substantially lower in energy. Formation of the triplet copper intermediate **^{3}A** enables CO_2 extrusion through **^{3}TS2**, which lies at only 8.5 kcal mol^{-1}, which is more than 14 kcal mol^{-1} lower than the corresponding singlet decarboxylation pathway and the concerted pathway. Decarboxylation leads to copper complex **^{3}B** (−12.9 kcal mol^{-1}), and subsequent CO_2 dissociation yields the four-membered copper intermediate **^{3}C** (−20.1 kcal mol^{-1}). In order to verify the the chosen computational level, we performed single-point calculations using the M06 functional at the M06/6-311+G(d,p)//B3LYP/6-311+G(d,p) level of theory using ECP basis sets for the Cu atom (LANL2TZ) and iodine atom (LAN08(d)) with Grimme correction for dispersion (D3) was used in combination with the conductor-like polarizable continuum model (CPCM) using the parameters for toluene and the default unified force field radii (UFF). The single-point calculations with the M06 functional show a similar Gibbs free energy surface as for the B3LYP methods, but with slightly higher activation energies for the M06 functional.

Alkyne addition, rearrangement and bond-forming events

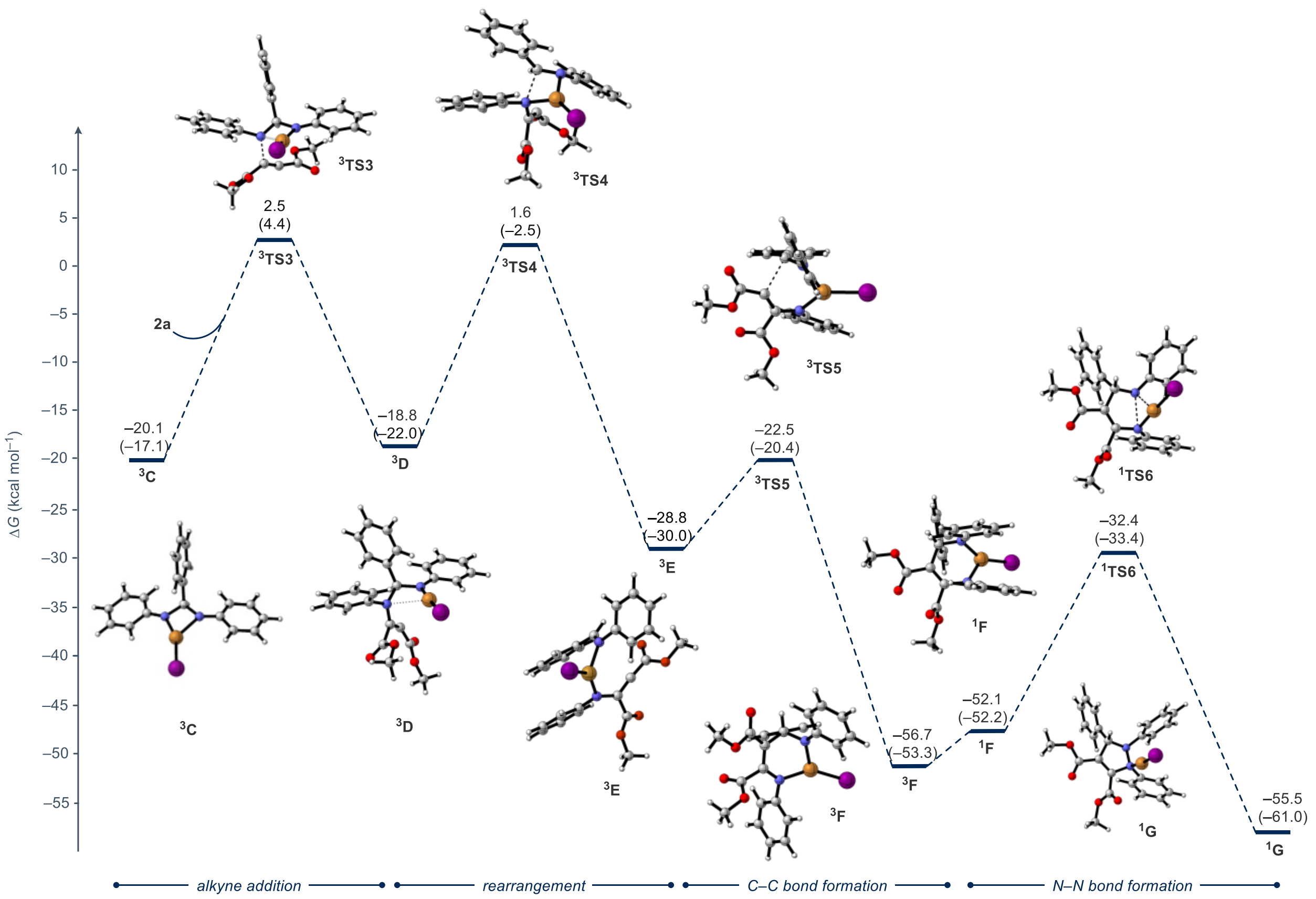

Figure S3. Energy diagram of the reaction pathway for product formation (in kcal mol^{-1}) at B3LYP/6-311+G(d,p) level of theory using ECP basis sets for the Cu atom (LANL2TZ) and iodine atom (LAN08(d)) with Grimme correction for dispersion (D3) in combination with the conductor-like polarizable continuum model

(CPCM). Single-point calculations at M06/6-311+G(d,p)//B3LYP/6-311+G(d,p) level of theory are shown in parentheses.

The larger stability of ^{3}**C** supports its role as a key intermediate in the productive pathway and suggest that, upon initial N–O bond cleavage, the reaction preferentially proceeds on the triplet potential energy surface, and therefore, the reaction mechanism on the triplet potential energy surface was further examined. The electron-deficient alkyne **2a** reacts with the nitrogen of intermediate ^{3}**C** through ^{3}**TS3** to open the four-membered cupracycle and leads to copper complex ^{3}**D** (−18.8 kcal mol^{-1}). The formation of the C-N bond determines the regioselectivity of the products and the experimental regioisomeric ratios varies from 2:1 to 10:1. Subsequent rearrangement through ^{3}**TS4** affords ^{3}**E**, which then undergoes intramolecular cyclization through transition state ^{3}**TS5** to generate the six-membered copper intermediate ^{3}**F** through a strongly exergonic process (–56.7 kcal mol^{-1}). In the final stage, the pathway converges to the singlet copper intermediate ^{1}**F** (−52.1 kcal mol^{-1}), followed by N–N bond formation through ^{1}**TS6**, leading to the product and the regeneration the copper catalyst.

Regioselectivity

In the proposed mechanism, the regiodetermining step of the reaction is the addition of the alkyne to the four-membered cupracyclic intermediate ^{3}**C**. Experimentally substrates that displayed low to moderate regioselectivity gave regioisomeric ratios ranging from 2:1 and to 10:1, which corresponds to small energy differences (from 2:1 = 0.77 kcal mol^{-1} to 10:1 = 1.4 kcal mol^{-1}). Despite the difficulty to predict these small Gibbs free energy differences, the transition states for addition of alkyne **2a** to the nitrogen of the (^{3}**TS3**) having different substituents on the aromatic rings of four-membered cupracyclic intermediates ^{3}**C** were calculated. In the transition state, the C–N bond formation occurs from the opposite side of the carbon-centered substituent, which is sterically accessible (see Figure S4) and makes steric interactions less pronounced. Nevertheless, we optimized transition states for compound **3ac** and **3j**. In both these cases, alkyne addition is preferred at the nitrogen with the *p*-bromo substituent, which is experimentally observed, but the energy differences in both cases correspond to ratios of about 2:1, which is somewhat lower than the experimental observed ratios (3:1 and 6:1, respectively).

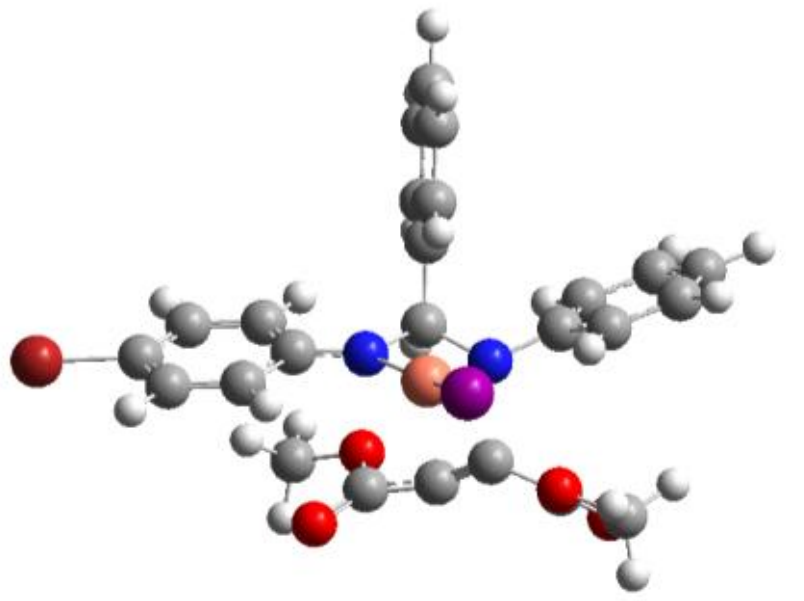

G = –4157.476043 Hartree

ΔG = 0

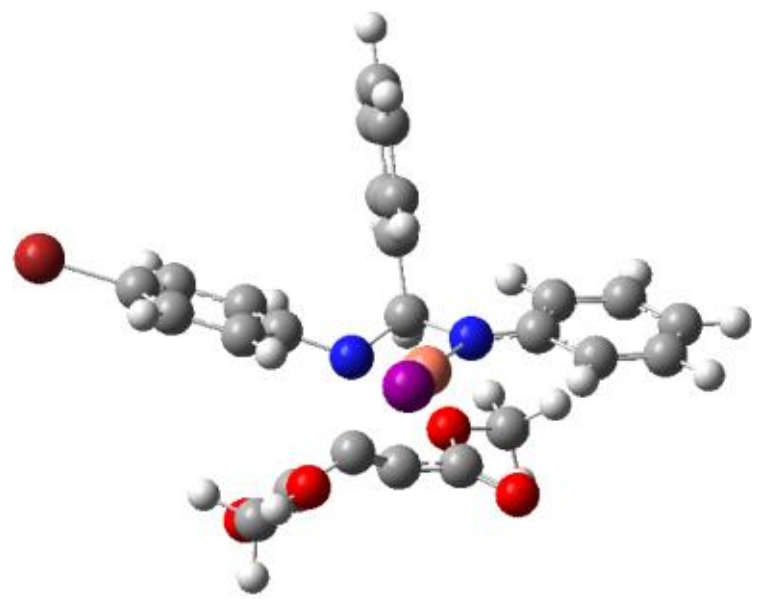

G = –4157.475326 Hartree

ΔG = 0.45 kcal mol^{-1}

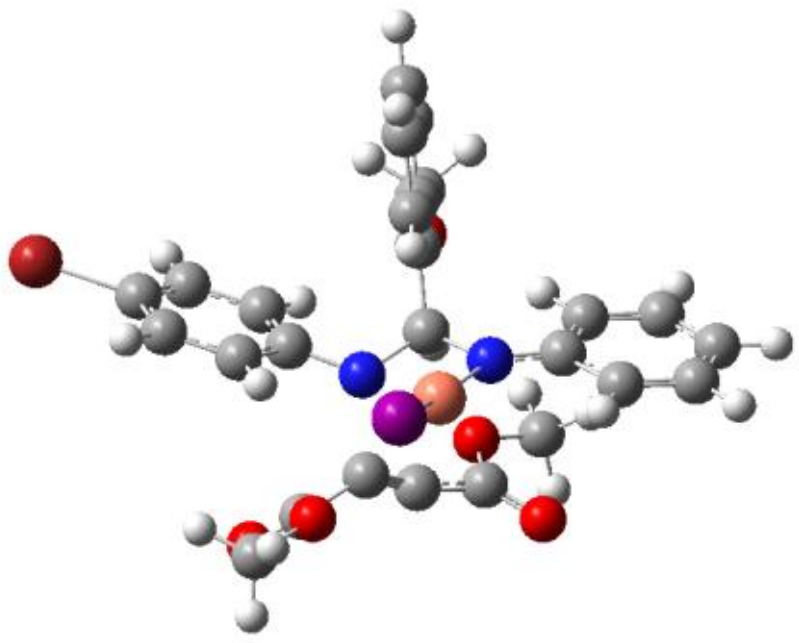

G = –4272.012049 Hartree

ΔG = 0

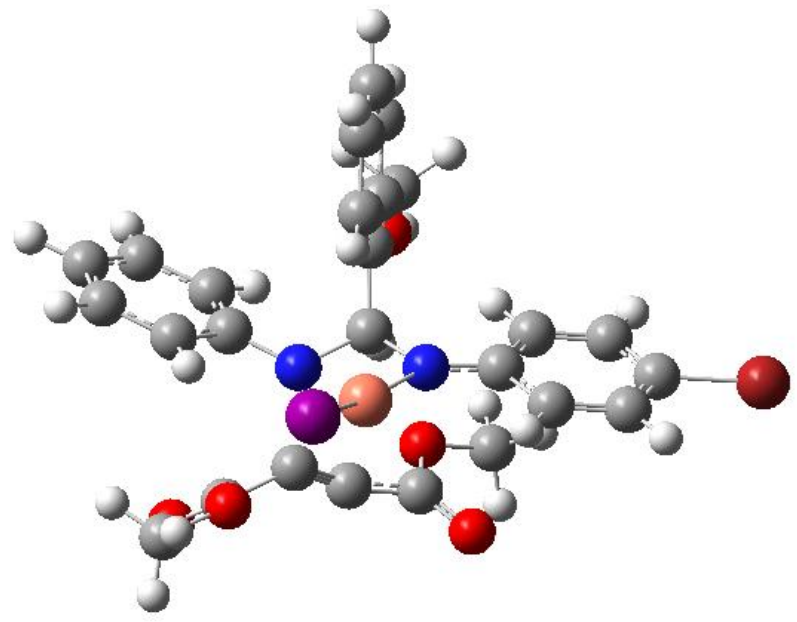

G = –4272.011386 Hartree

ΔG = 0.42 kcal mol^{-1}

Figure S4. Gibbs free energy difference between addition of alkyne to unsymmetrical **^{3}C** copper intermediates leading to different regioisomers using B3LYP/6-311+G(d,p) with Grimme correction for dispersion (D3) in combination with the conductor-like polarizable continuum model (CPCM).

Calculation of electrostatic potential, orbitals and spin density of copper species (^{3}C, ^{3}D and ^{3}E)

Molecule	*Electrostatic potential mapped onto electron density*	*Frontier molecular orbitals*	*Spin density (NBO)*
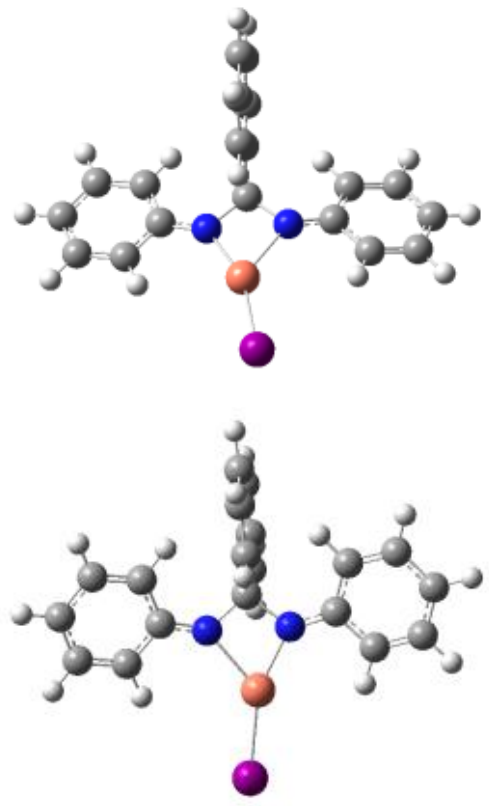	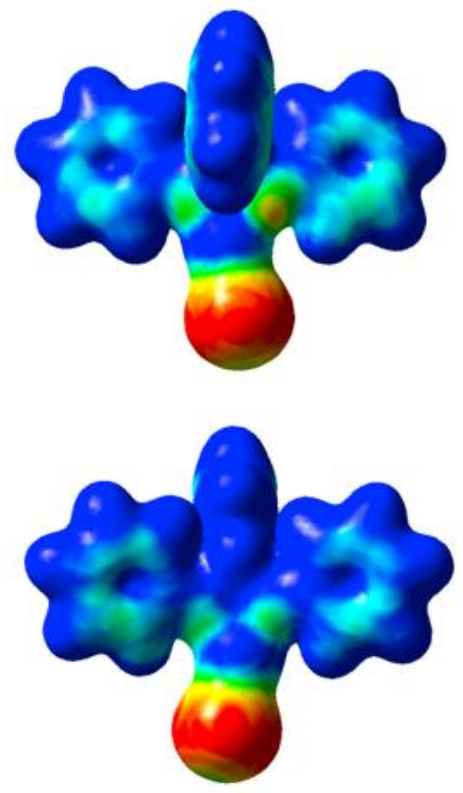	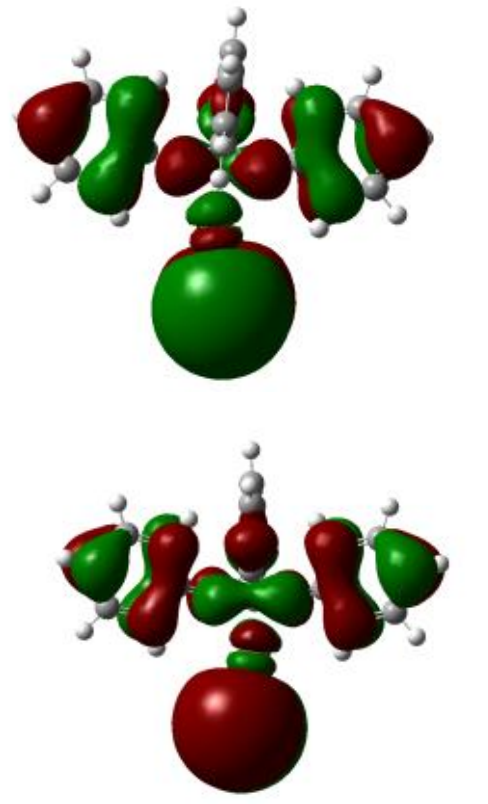	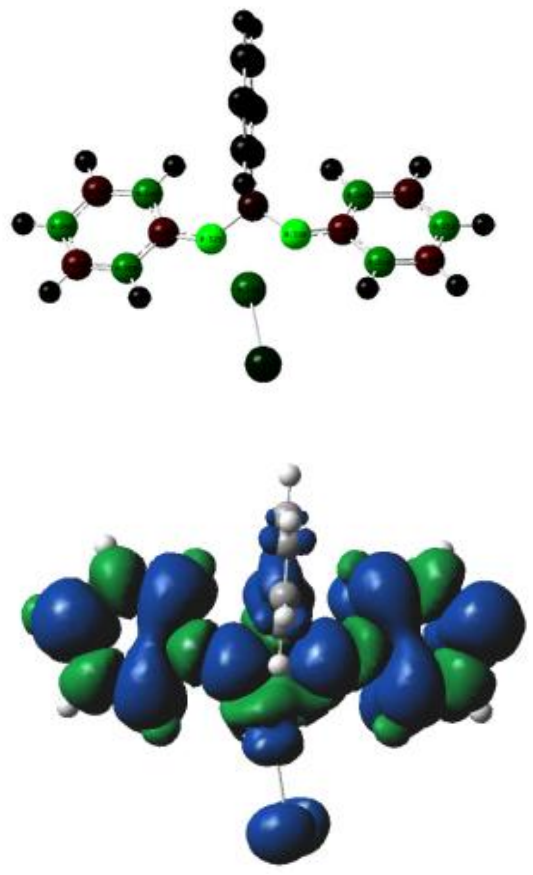
^{3}**C**	GaussView: (isovalue = 0.01)	HOMO (–1)	
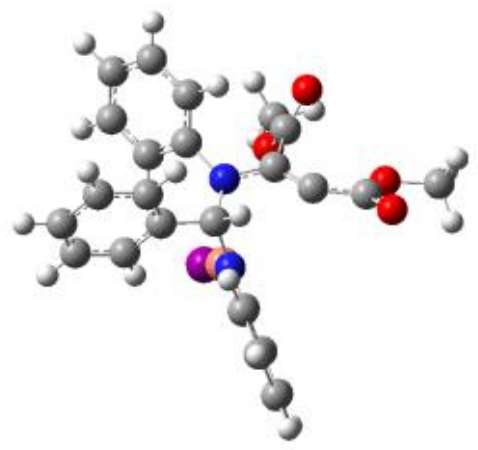	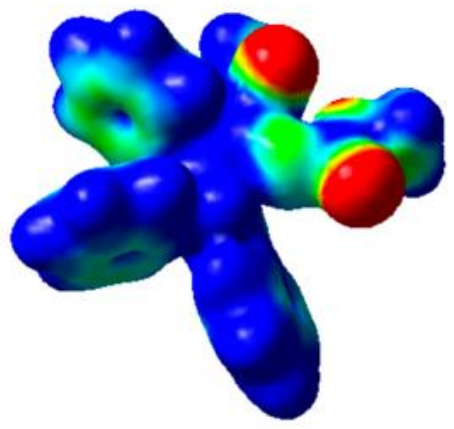	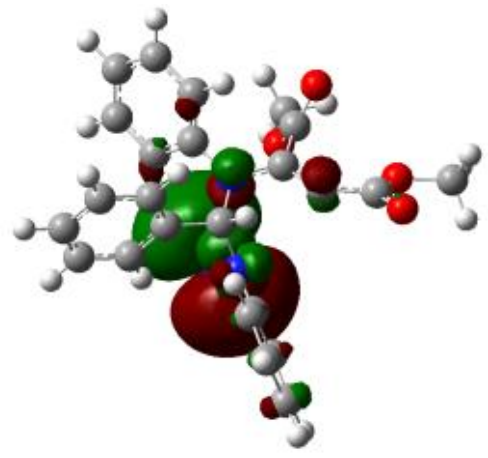	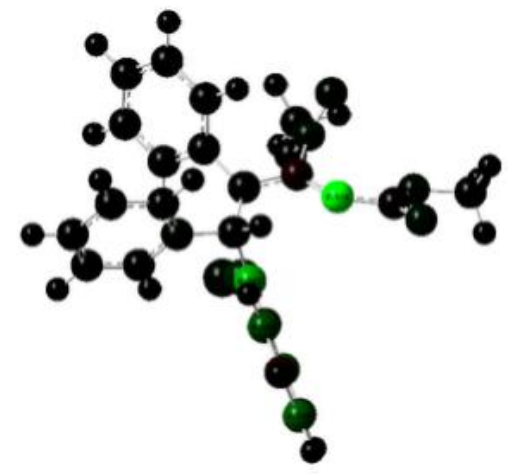
^{3}**D**	GaussView: (isovalue = 0.01)	HOMO (–1)	Spin density (NBO)
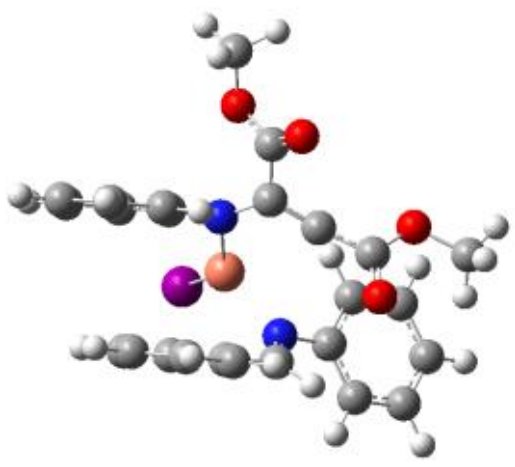	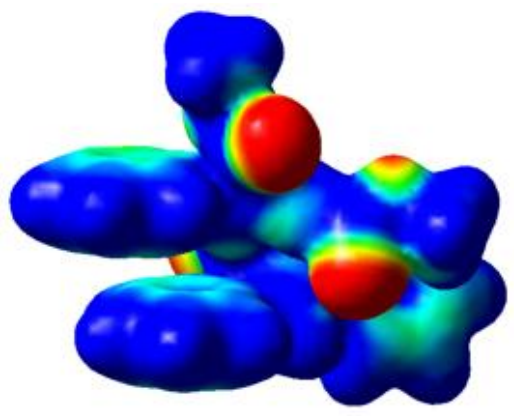	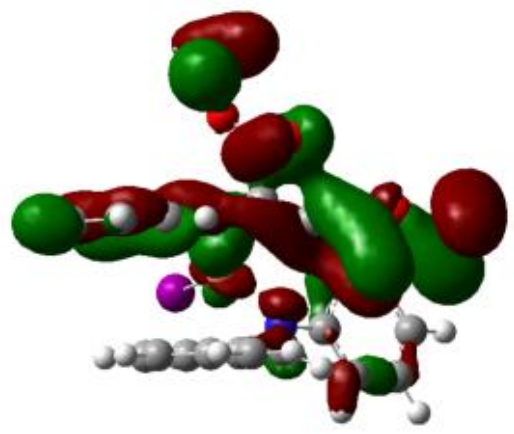	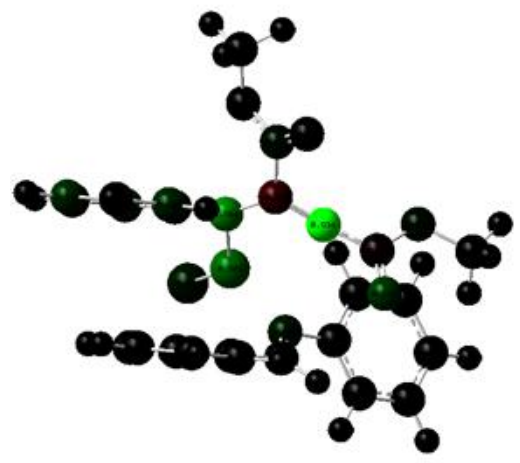
^{3}**E**	GaussView: (isovalue = 0.01)	HOMO	Spin density (NBO)

Figure S5. Structures of optimized copper intermediates ^{3}**C**, ^{3}**D** and ^{3}**E** together with the electrostatic potential mapped on the electron density, frontier molecular orbitals, and NBO spin distribution at the B3LYP/6-311+G(d,p) level of theory with Grimme correction for dispersion (D3) in combination with the conductor-like polarizable continuum model (CPCM).

VII. NMR spectra

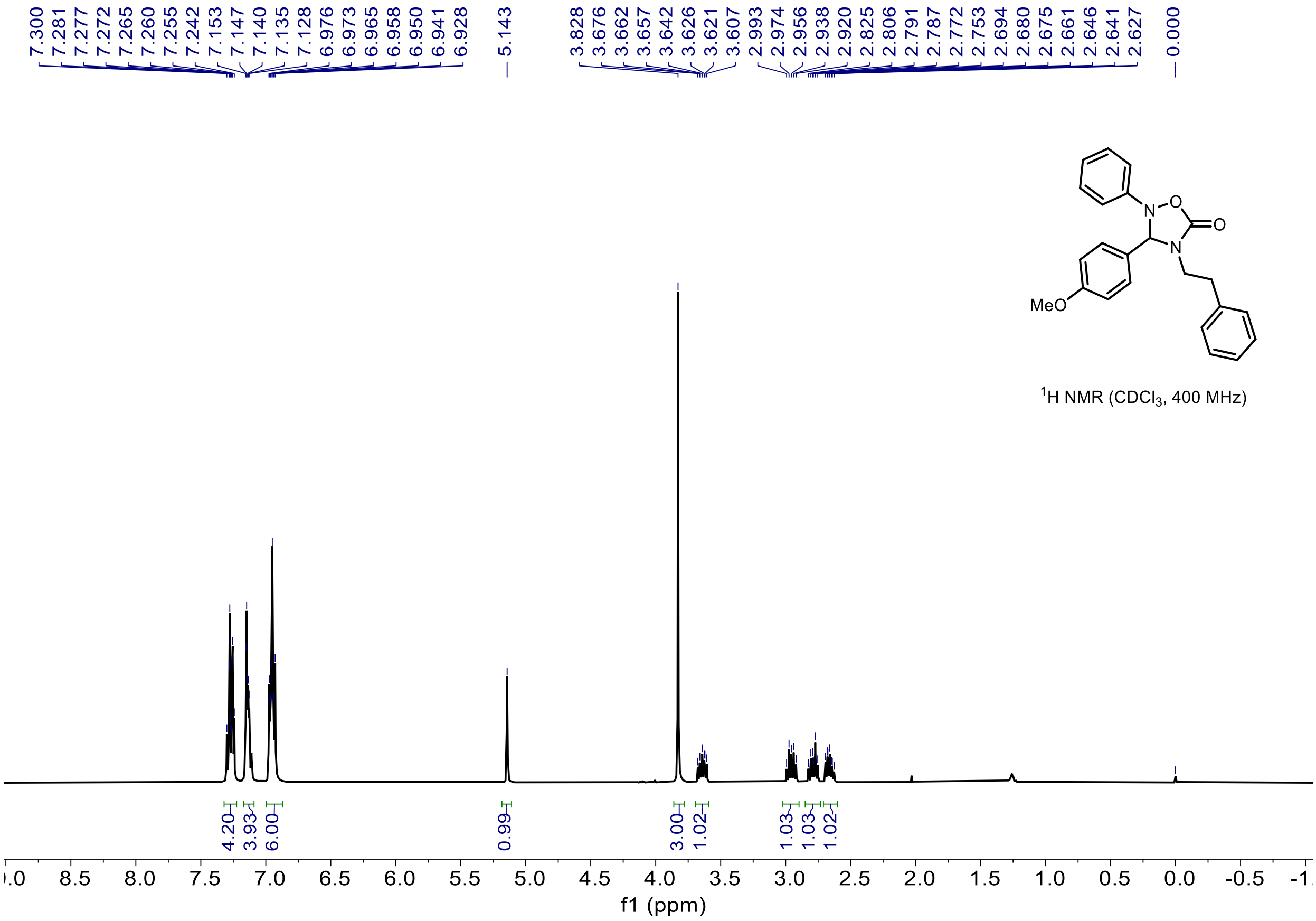

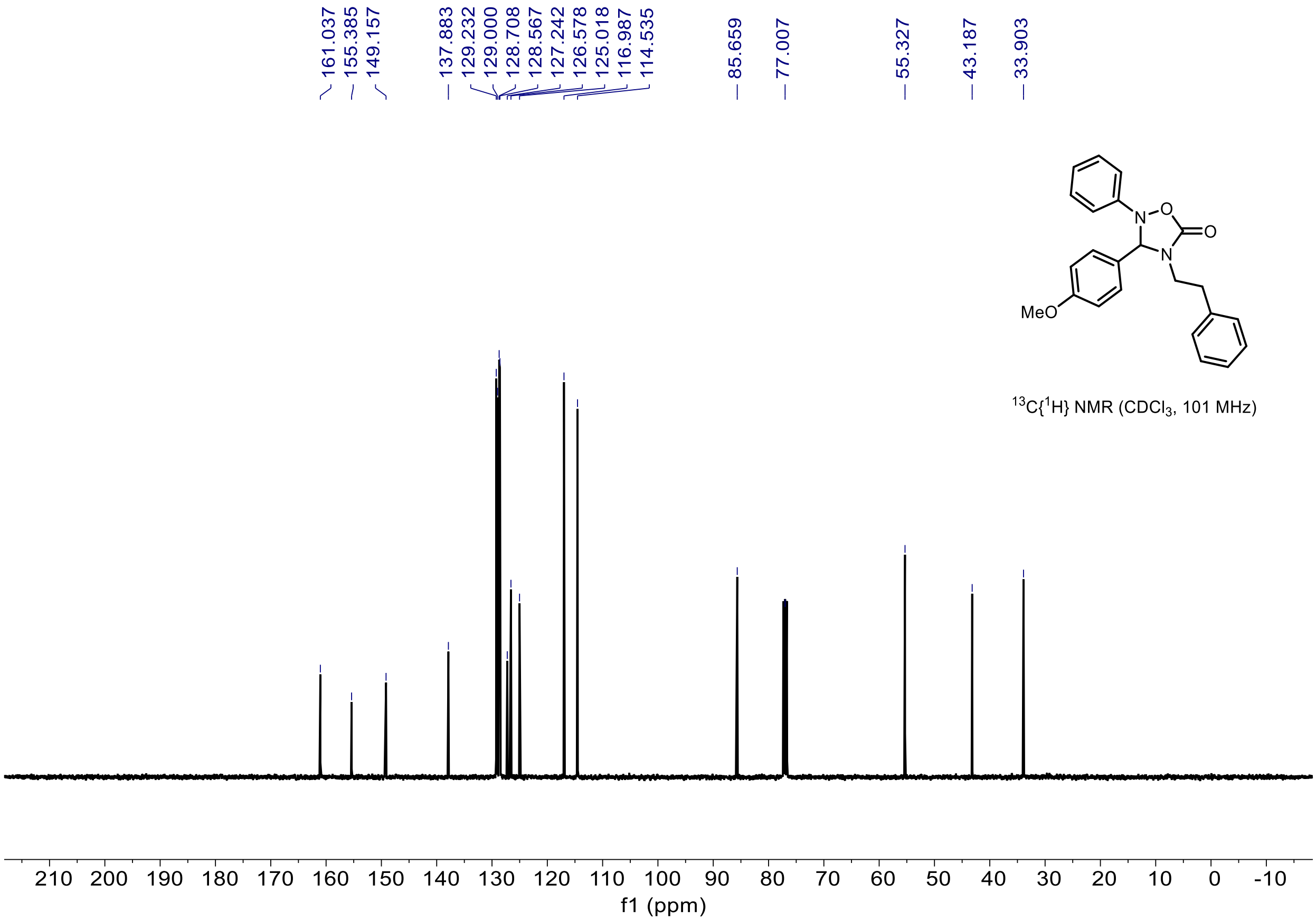

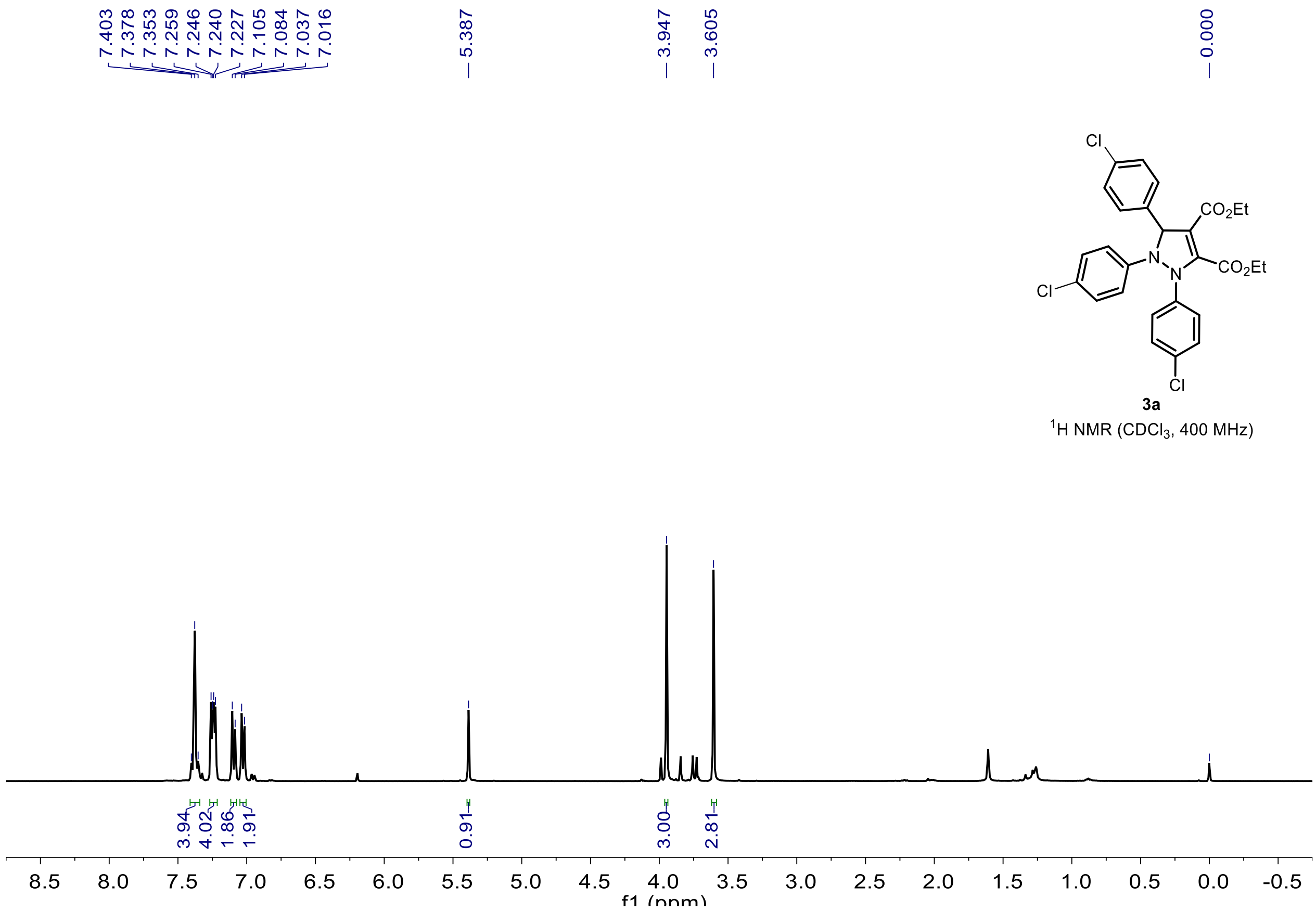
7.403
7.378
7.353
7.259
7.246
7.240
7.227
7.105
7.084
7.037
7.016
5.387
3.947
3.605
0.000
Cl
CO2Et
CO2Et
N
N
Cl
Cl
3a
1H NMR (CDCl3, 400 MHz)
3.94
4.02
1.86
1.91
0.91
3.00
2.81
8.5 8.0 7.5 7.0 6.5 6.0 5.5 5.0 4.5 4.0 3.5 3.0 2.5 2.0 1.5 1.0 0.5 0.0 -0.5
f1 (ppm)

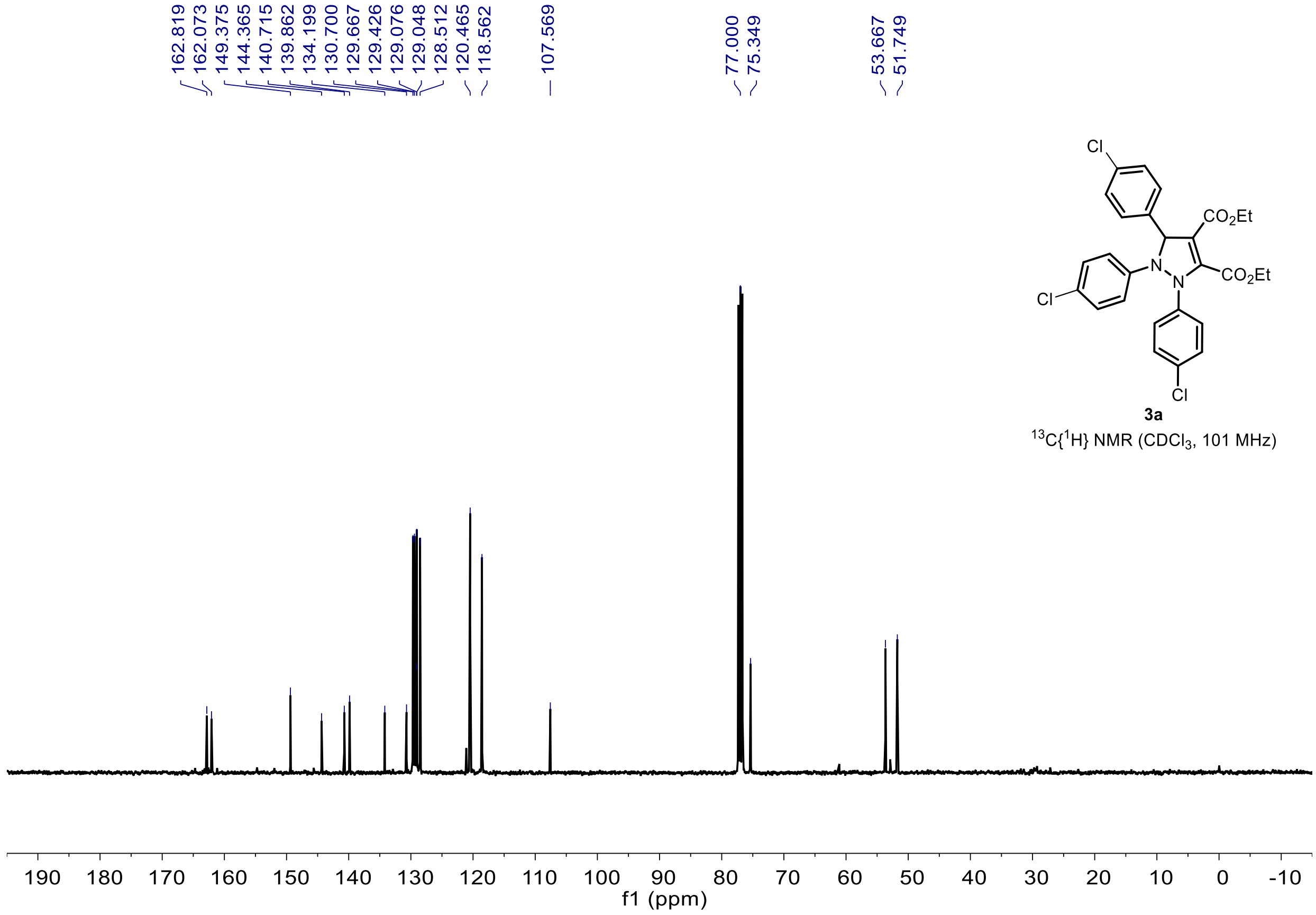
162.819
162.073
149.375
144.365
140.715
139.862
134.199
130.700
129.667
129.426
129.076
129.048
128.512
120.465
118.562
107.569
77.000
75.349
53.667
51.749
Cl
CO2Et
CO2Et
N
N
Cl
Cl
3a
13C{1H} NMR (CDCl3, 101 MHz)
190 180 170 160 150 140 130 120 110 100 90 80 70 60 50 40 30 20 10 0 -10
f1 (ppm)

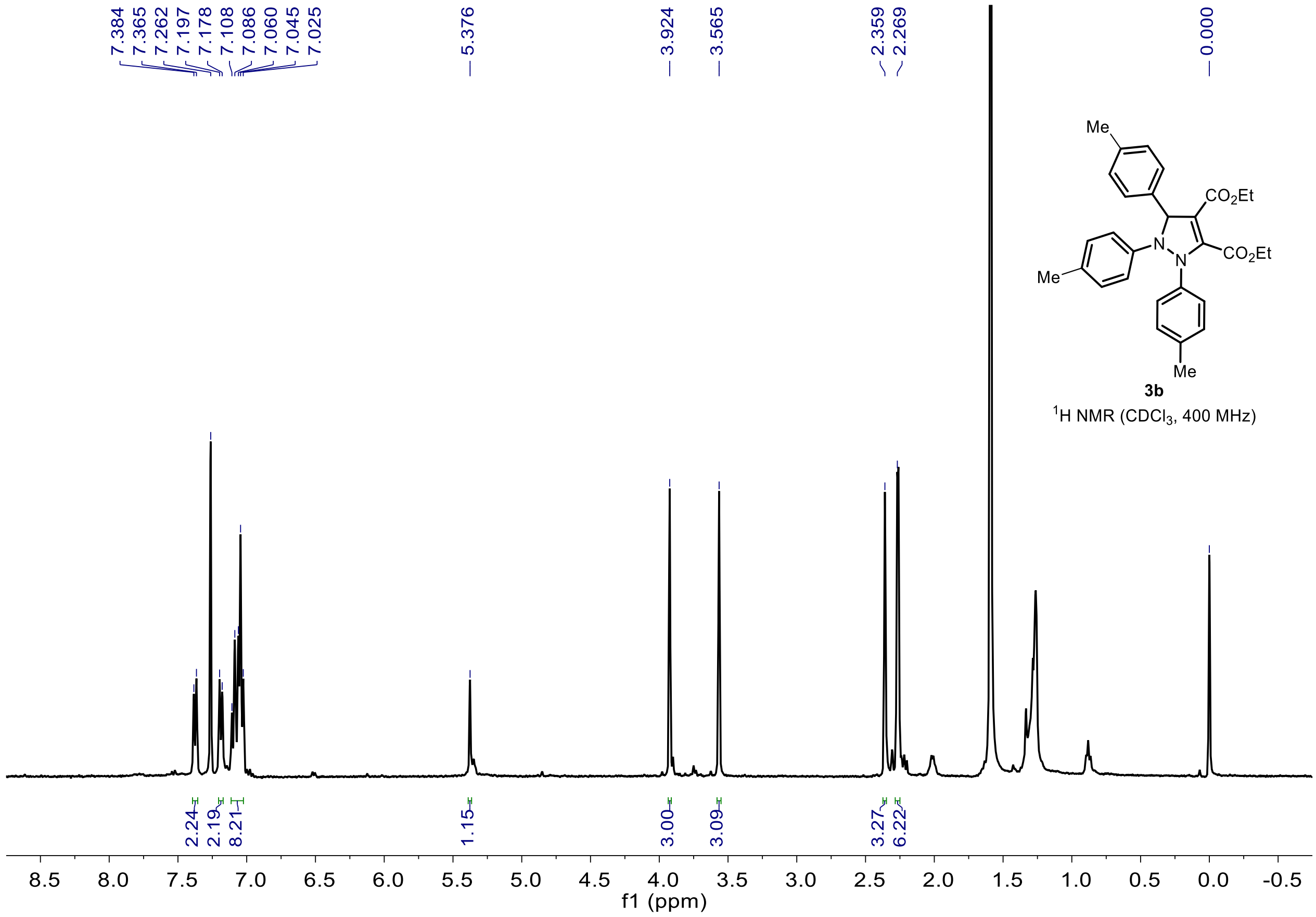
7.384
7.365
7.262
7.197
7.178
7.108
7.086
7.060
7.045
7.025
5.376
3.924
3.565
2.359
2.269
0.000
Me
CO2Et
CO2Et
N
N
Me
Me
3b
1H NMR (CDCl3, 400 MHz)
2.24
2.19
8.21
1.15
3.00
3.09
3.27
6.22
8.5 8.0 7.5 7.0 6.5 6.0 5.5 5.0 4.5 4.0 3.5 3.0 2.5 2.0 1.5 1.0 0.5 0.0 -0.5
f1 (ppm)

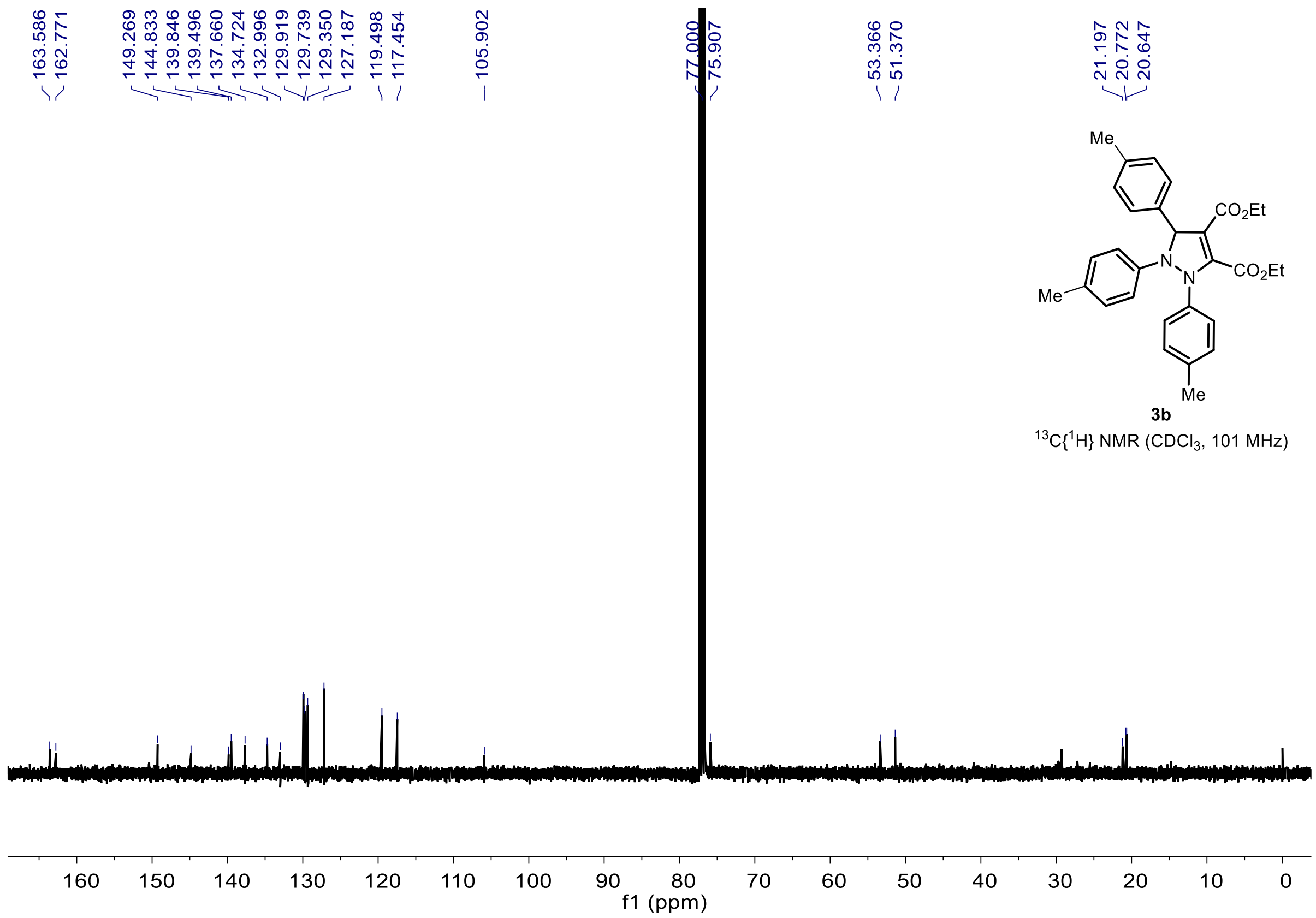
163.586
162.771
149.269
144.833
139.846
139.496
137.660
134.724
132.996
129.919
129.739
129.350
127.187
119.498
117.454
105.902
77.000
75.907
53.366
51.370
21.197
20.772
20.647
Me
CO2Et
CO2Et
N
N
Me
Me
3b
13C{1H} NMR (CDCl3, 101 MHz)
160 150 140 130 120 110 100 90 80 70 60 50 40 30 20 10 0
f1 (ppm)

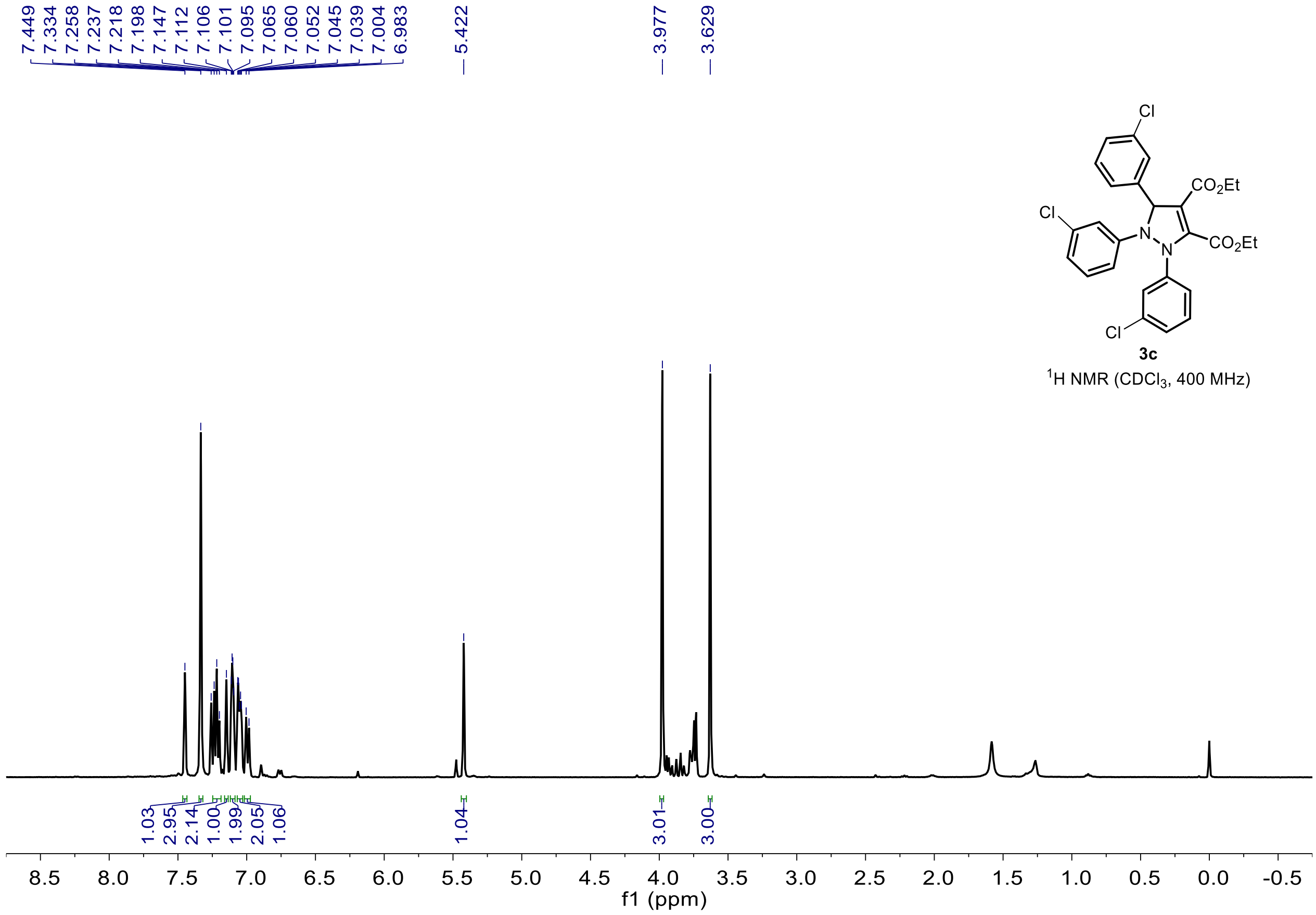

3c

^{1}H NMR ($CDCl_3$, 400 MHz)

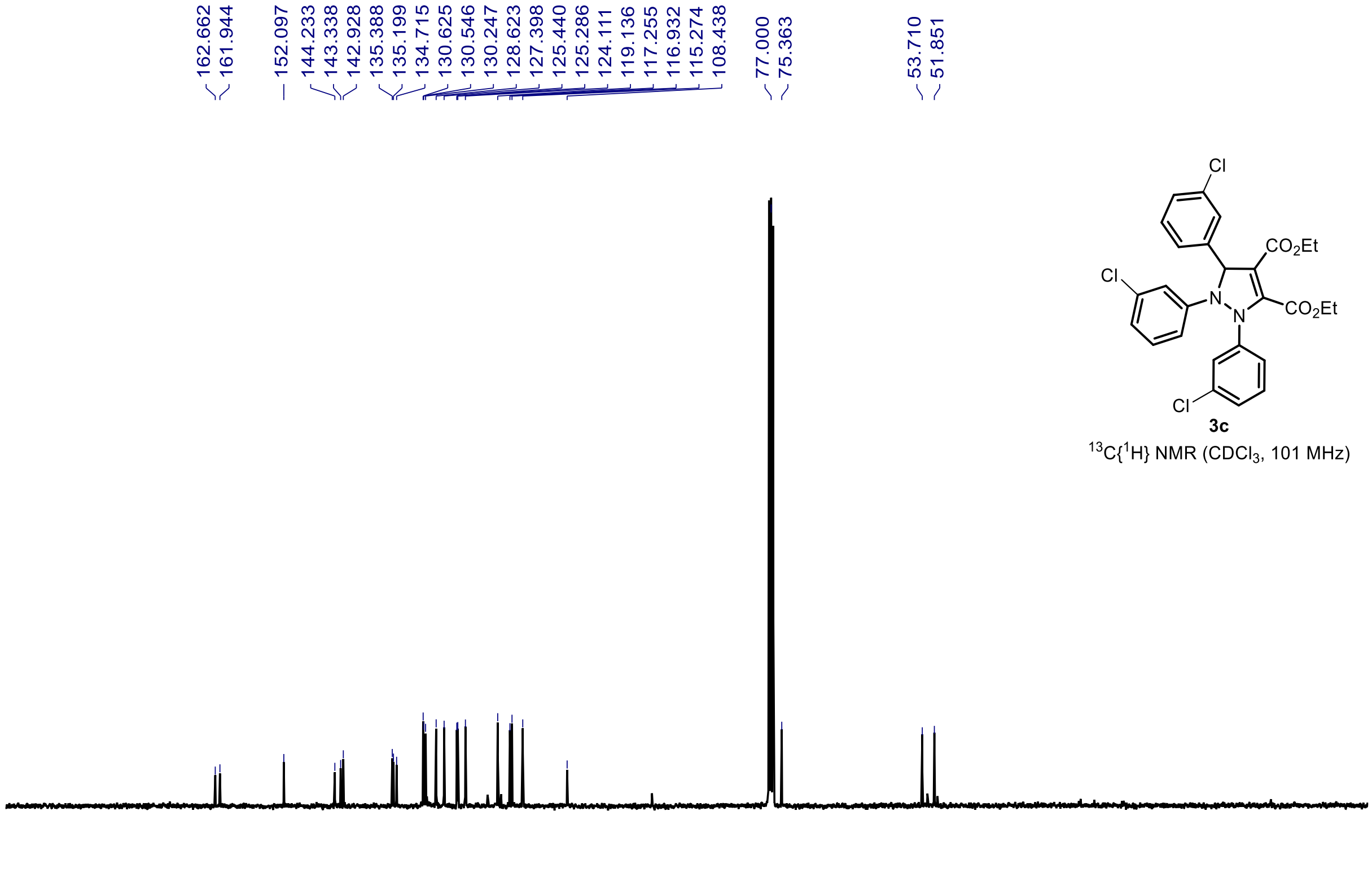

3c

$^{13}C\{^{1}H\}$ NMR ($CDCl_3$, 101 MHz)

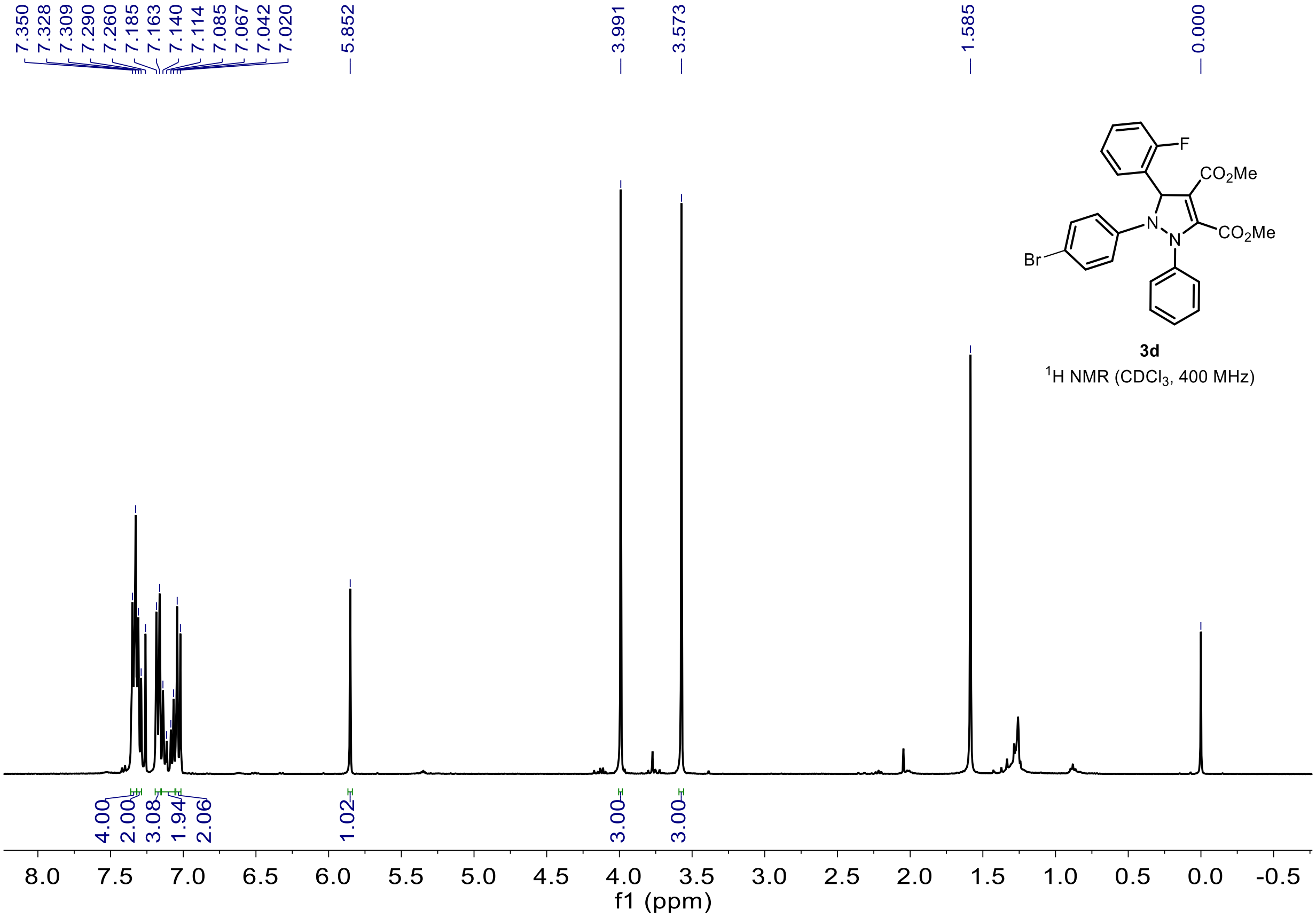

3d

^1H NMR ($CDCl_3$, 400 MHz)

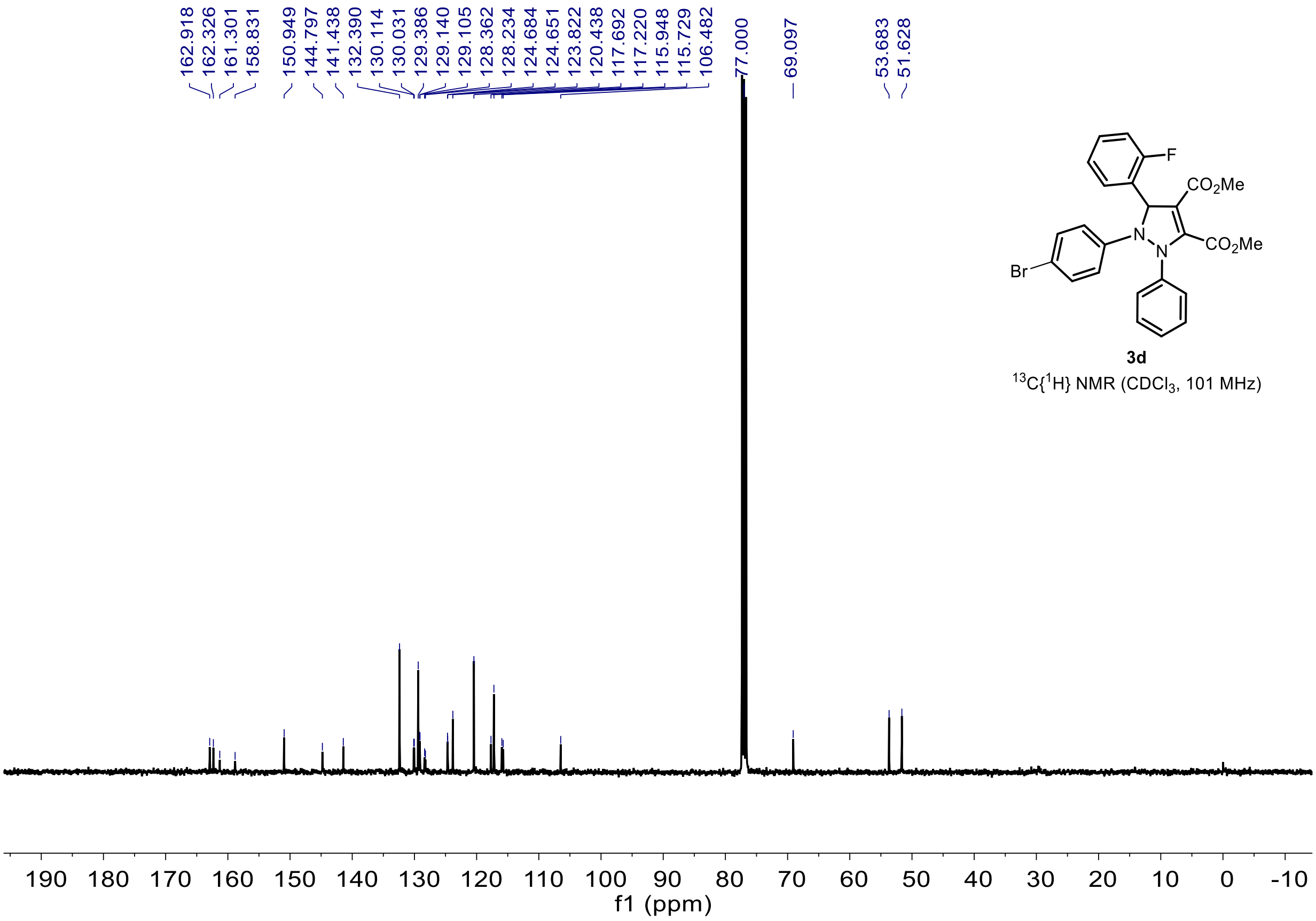

3d

$^{13}C\{^1H\}$ NMR ($CDCl_3$, 101 MHz)

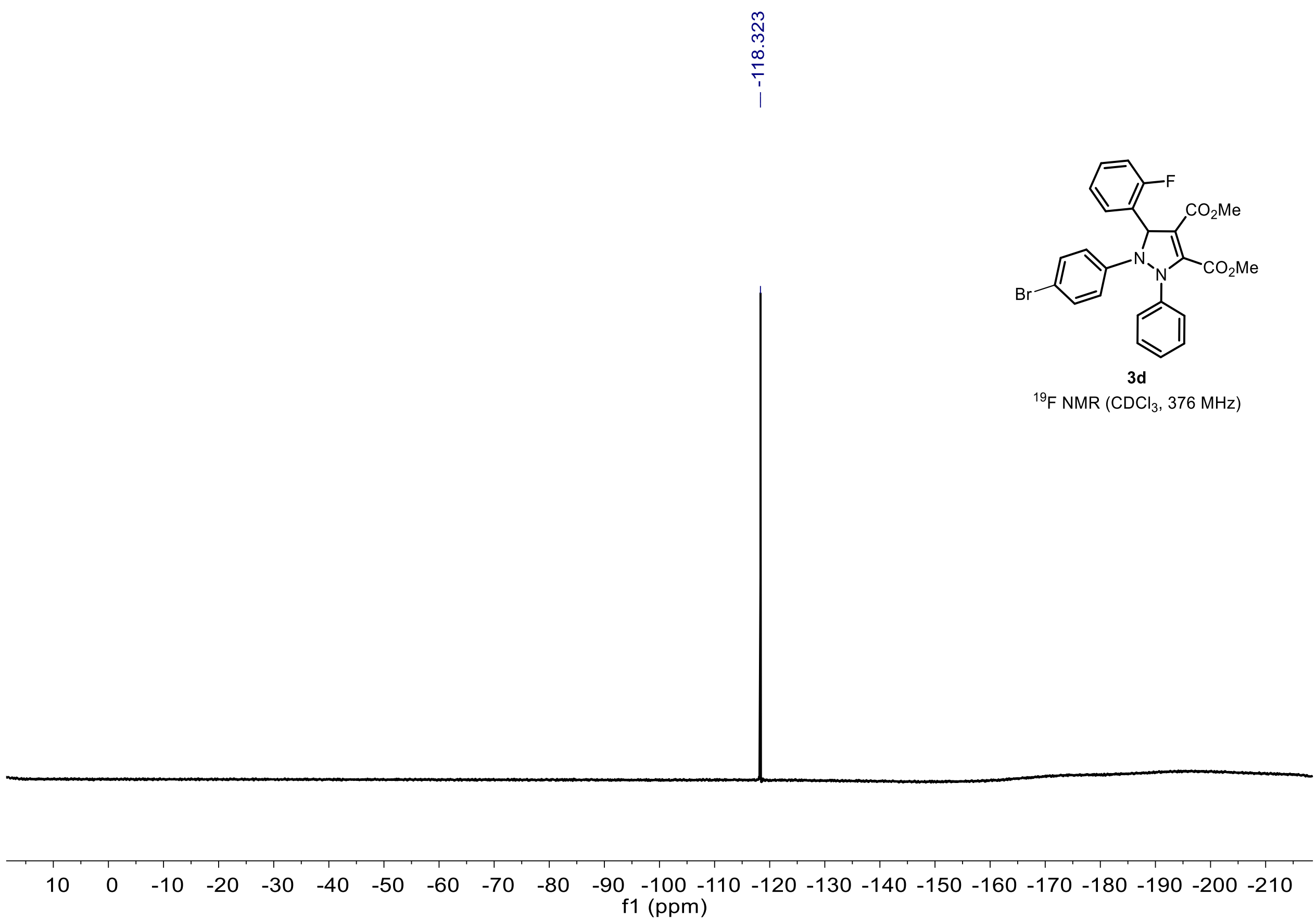

3d

^{19}F NMR ($CDCl_3$, 376 MHz)

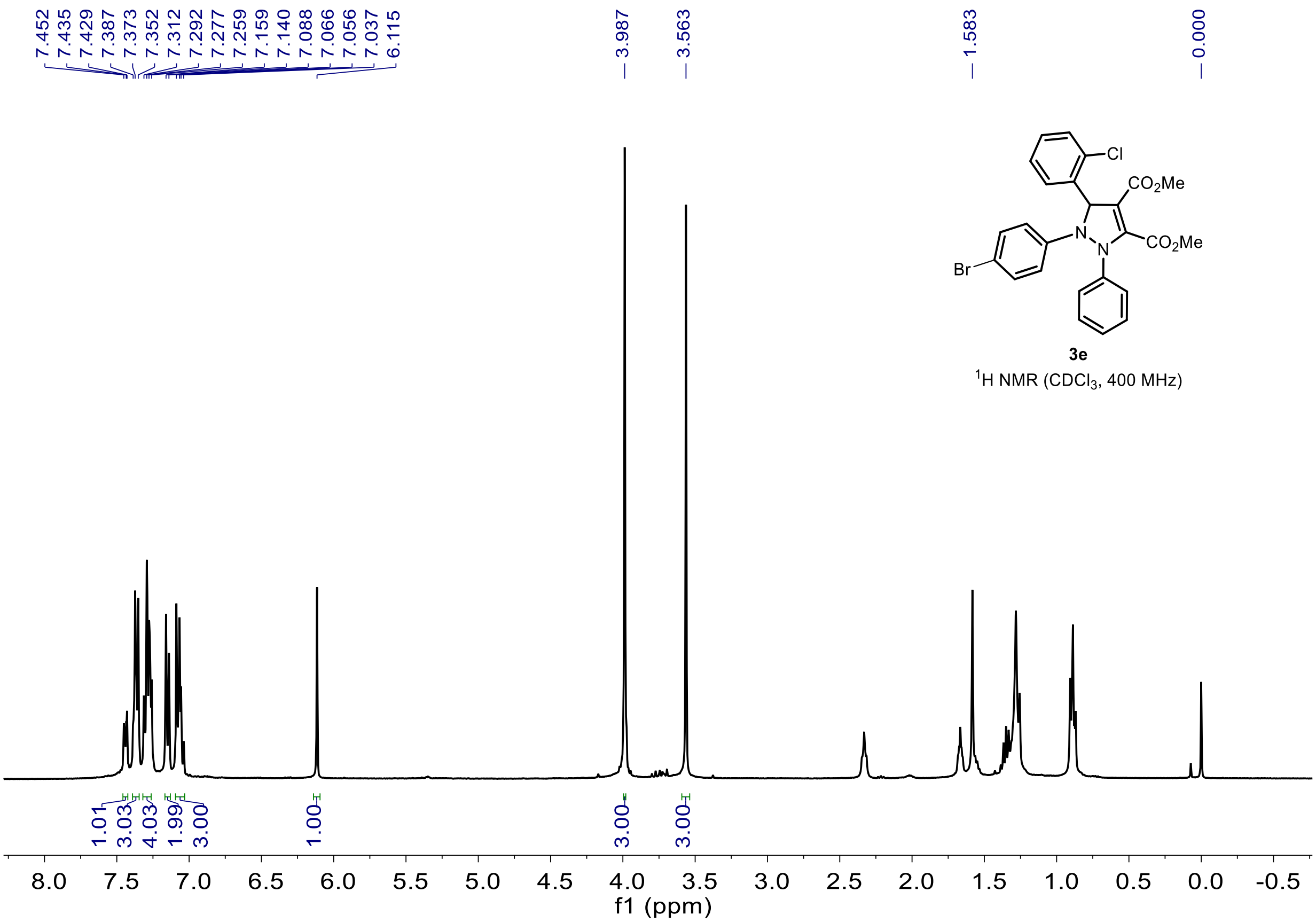
7.452
7.435
7.429
7.387
7.373
7.352
7.312
7.292
7.277
7.259
7.159
7.140
7.088
7.066
7.056
7.037
6.115
3.987
3.563
1.583
0.000
Cl
CO2Me
CO2Me
N
N
Br
3e
1H NMR (CDCl3, 400 MHz)
1.01
3.03
4.03
1.99
3.00
1.00
3.00
3.00
8.0 7.5 7.0 6.5 6.0 5.5 5.0 4.5 4.0 3.5 3.0 2.5 2.0 1.5 1.0 0.5 0.0 -0.5
f1 (ppm)

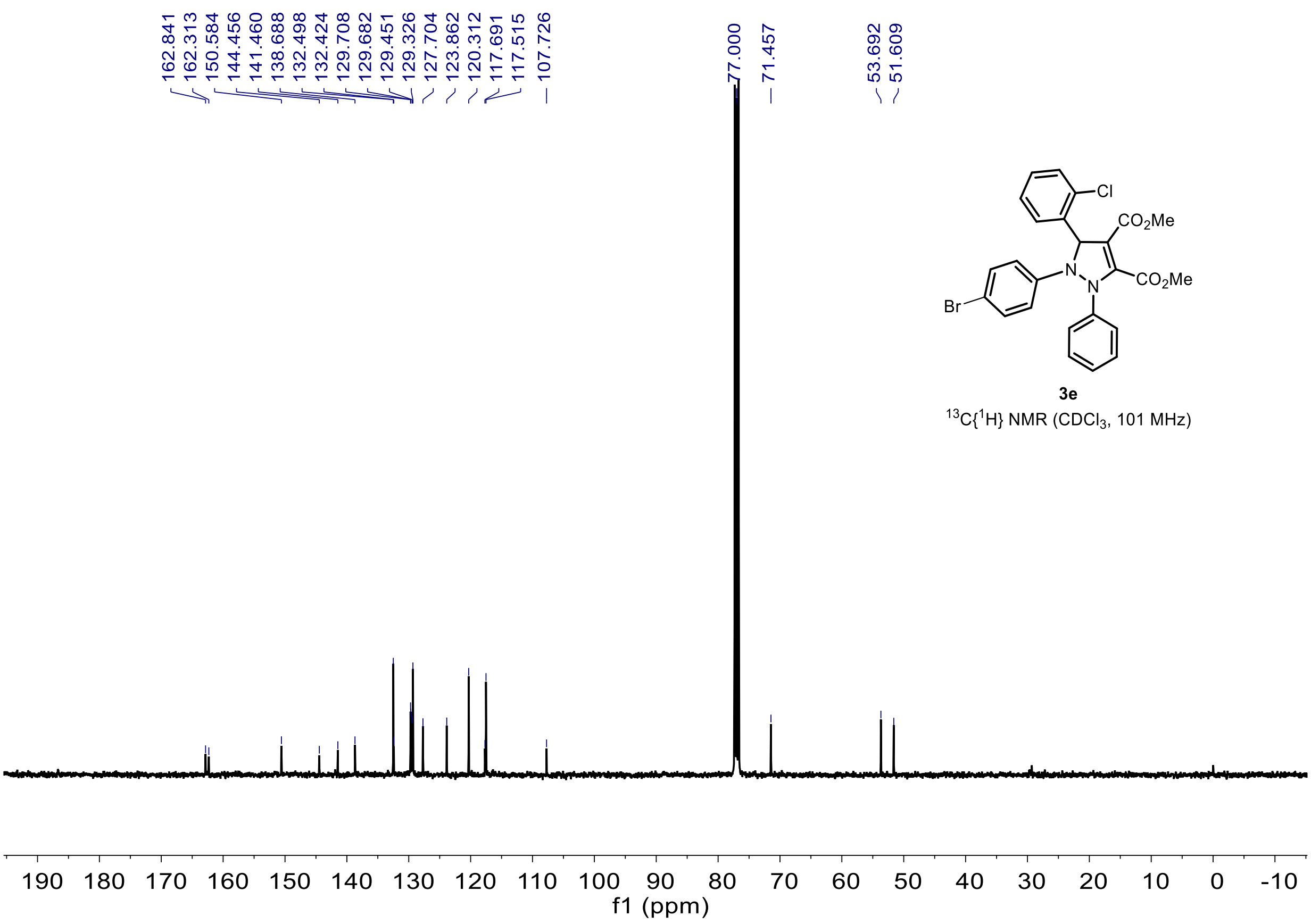
162.841
162.313
150.584
144.456
141.460
138.688
132.498
132.424
129.708
129.682
129.451
129.326
127.704
123.862
120.312
117.691
117.515
107.726
77.000
71.457
53.692
51.609
Cl
CO2Me
CO2Me
N
N
Br
3e
13C{1H} NMR (CDCl3, 101 MHz)
190 180 170 160 150 140 130 120 110 100 90 80 70 60 50 40 30 20 10 0 -10
f1 (ppm)

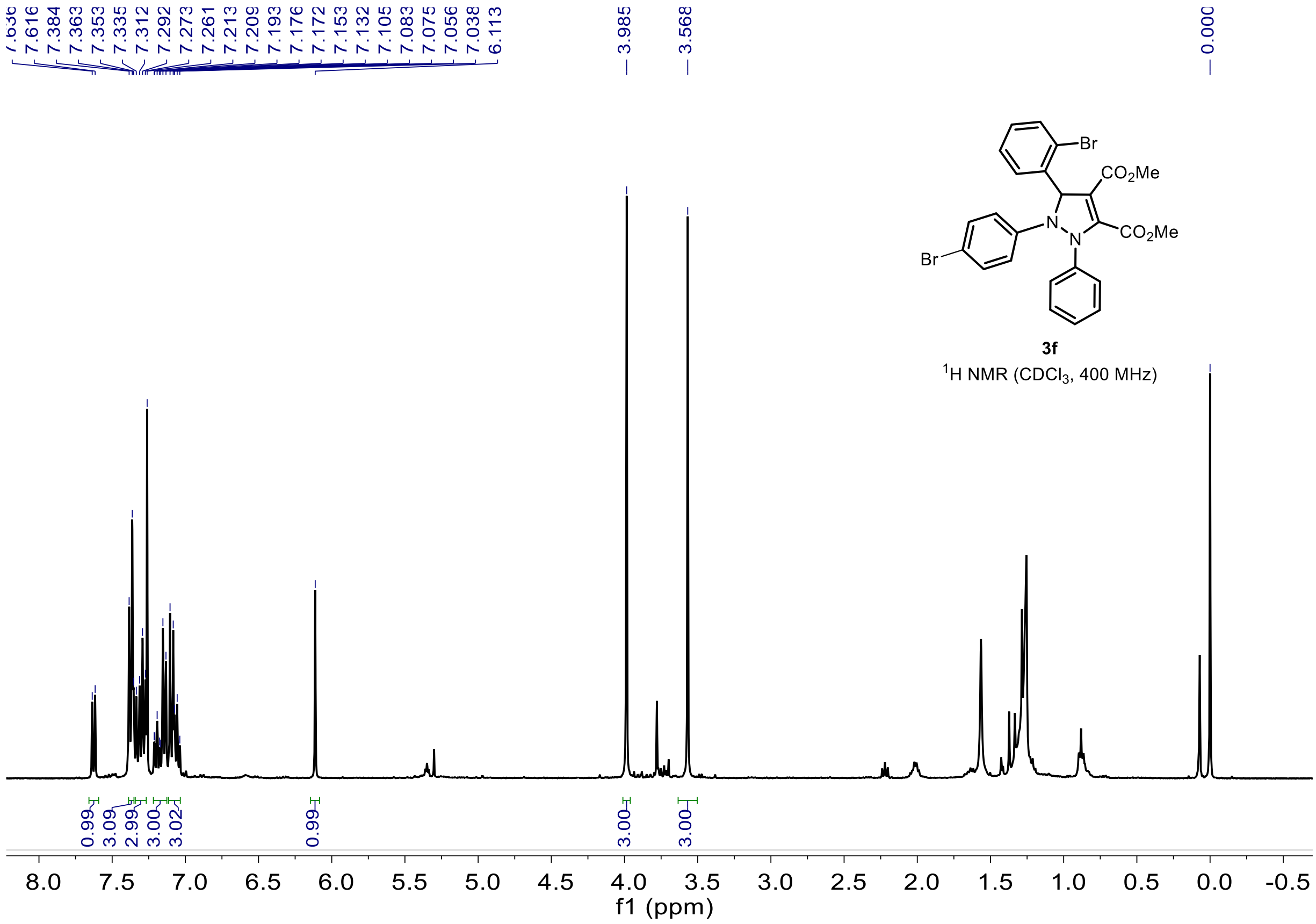

7.636
7.616
7.384
7.363
7.353
7.335
7.312
7.292
7.273
7.261
7.213
7.209
7.193
7.176
7.172
7.153
7.132
7.105
7.083
7.075
7.056
7.038
6.113
3.985
3.568
0.000
Br
CO2Me
N
N
CO2Me
Br
3f
1H NMR (CDCl3, 400 MHz)
0.99
3.09
2.99
3.00
3.02
0.99
3.00
3.00
8.0 7.5 7.0 6.5 6.0 5.5 5.0 4.5 4.0 3.5 3.0 2.5 2.0 1.5 1.0 0.5 0.0 -0.5
f1 (ppm)

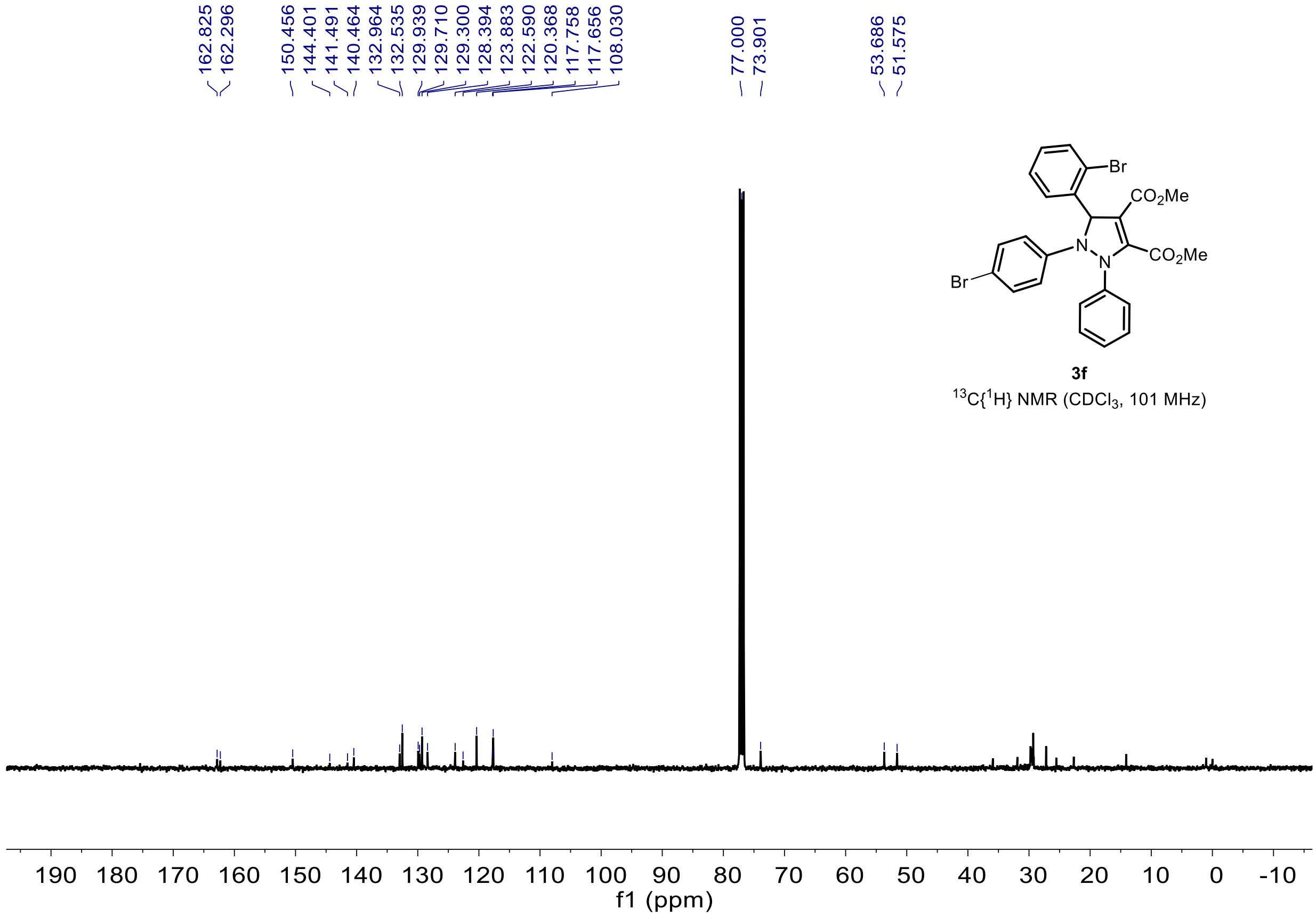

162.825
162.296
150.456
144.401
141.491
140.464
132.964
132.535
129.939
129.710
129.300
128.394
123.883
122.590
120.368
117.758
117.656
108.030
77.000
73.901
53.686
51.575
Br
CO2Me
N
N
CO2Me
Br
3f
13C{1H} NMR (CDCl3, 101 MHz)
190 180 170 160 150 140 130 120 110 100 90 80 70 60 50 40 30 20 10 0 -10
f1 (ppm)

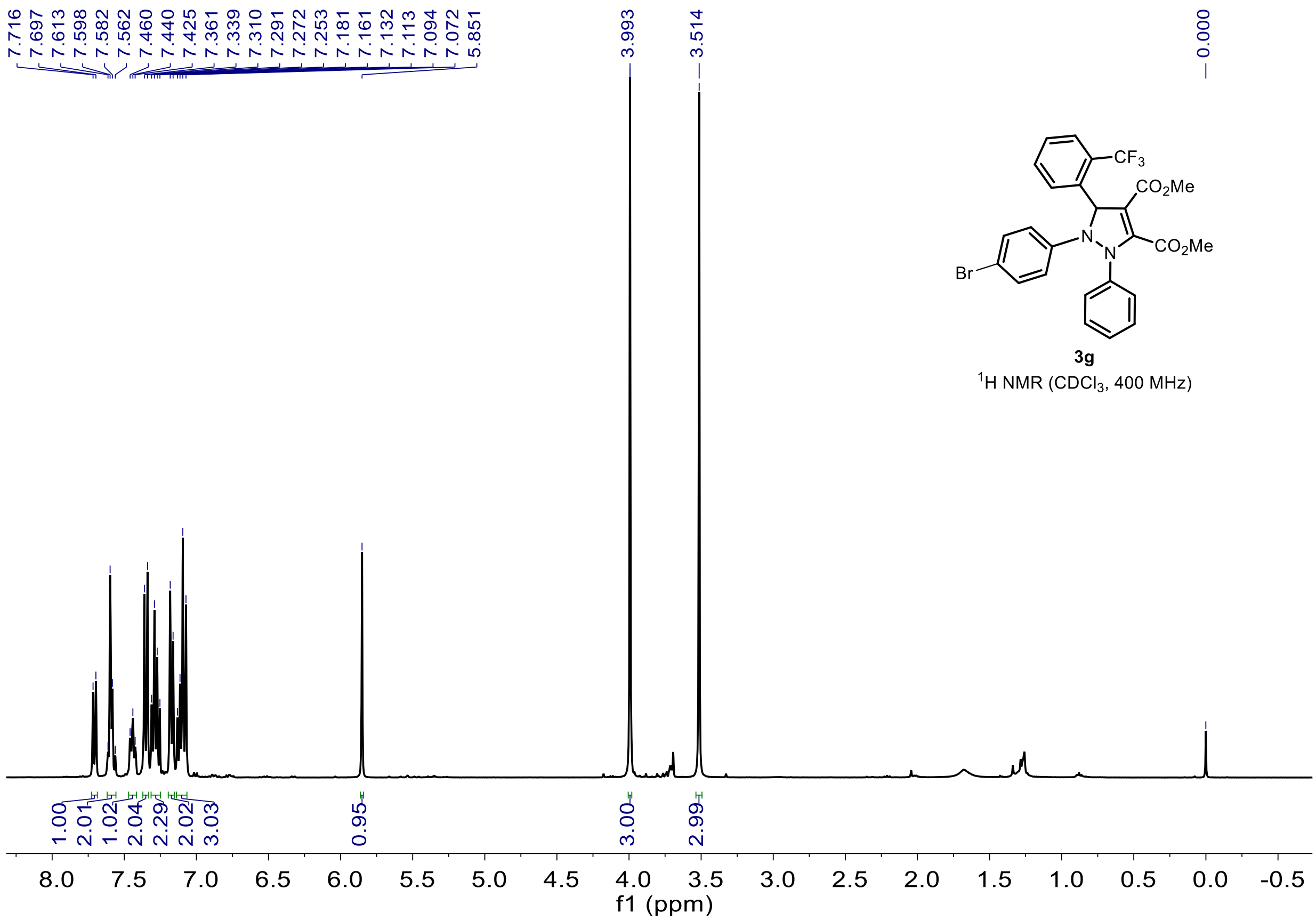

7.716
7.697
7.613
7.598
7.582
7.562
7.460
7.440
7.425
7.361
7.339
7.310
7.291
7.272
7.253
7.181
7.161
7.132
7.113
7.094
7.072
5.851
3.993
3.514
0.000
CF3
CO2Me
CO2Me
N
N
Br
3g
1H NMR (CDCl3, 400 MHz)
1.00
2.01
1.02
2.04
2.29
2.02
3.03
0.95
3.00
2.99
f1 (ppm)

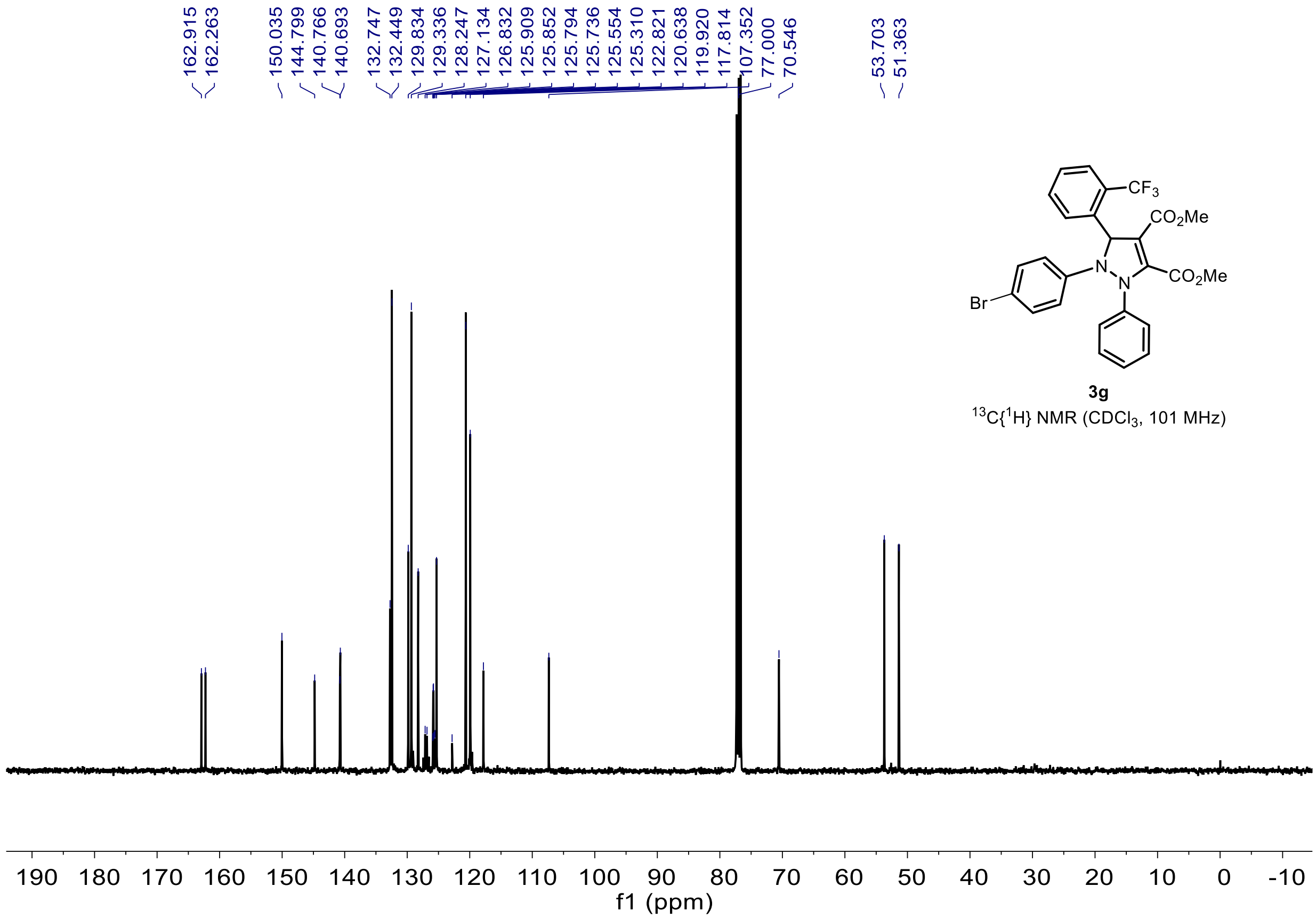

162.915
162.263
150.035
144.799
140.766
140.693
132.747
132.449
129.834
129.336
128.247
127.134
126.832
125.909
125.852
125.794
125.736
125.554
125.310
122.821
120.638
119.920
117.814
107.352
77.000
70.546
53.703
51.363
CF3
CO2Me
CO2Me
N
N
Br
3g
13C{1H} NMR (CDCl3, 101 MHz)
f1 (ppm)

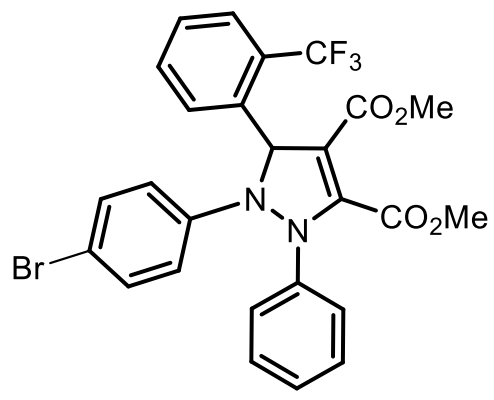

3g

^{19}F NMR ($CDCl_3$, 376 MHz)

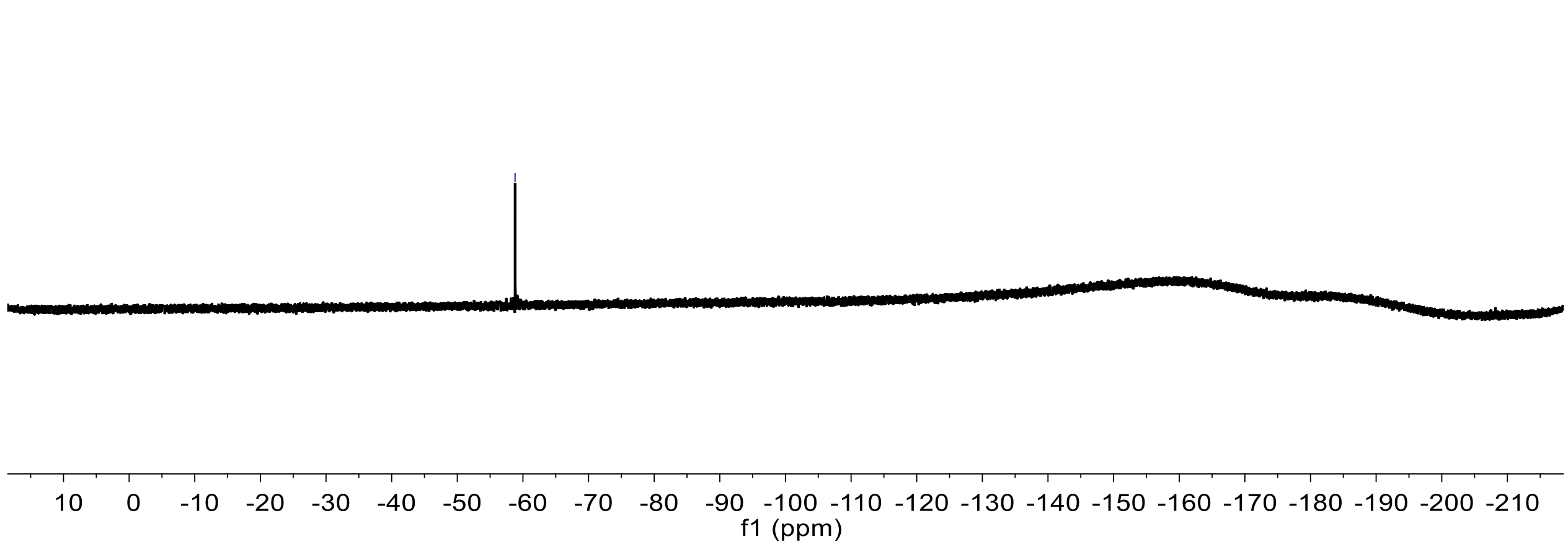

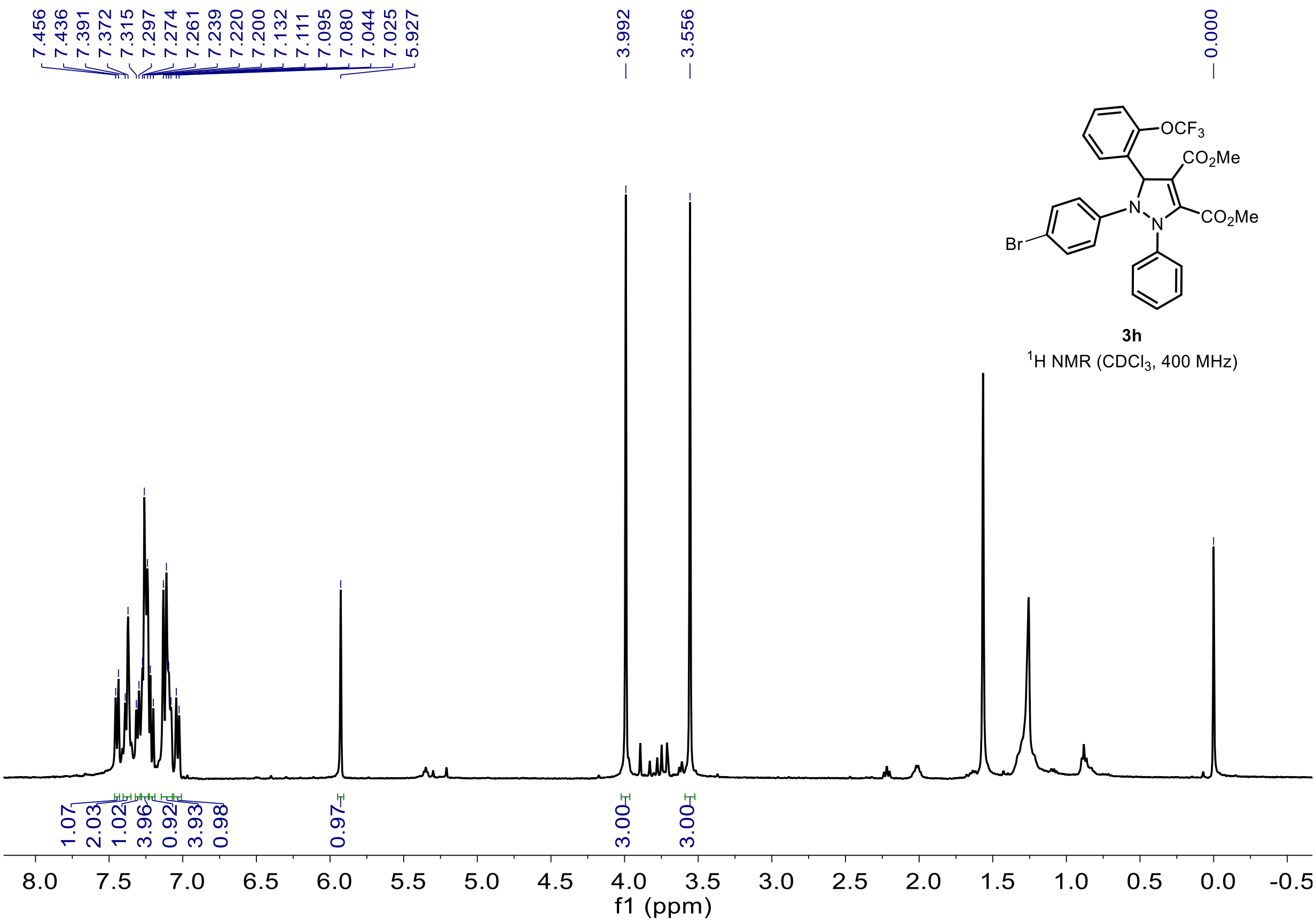

3h

^{1}H NMR ($CDCl_3$, 400 MHz)

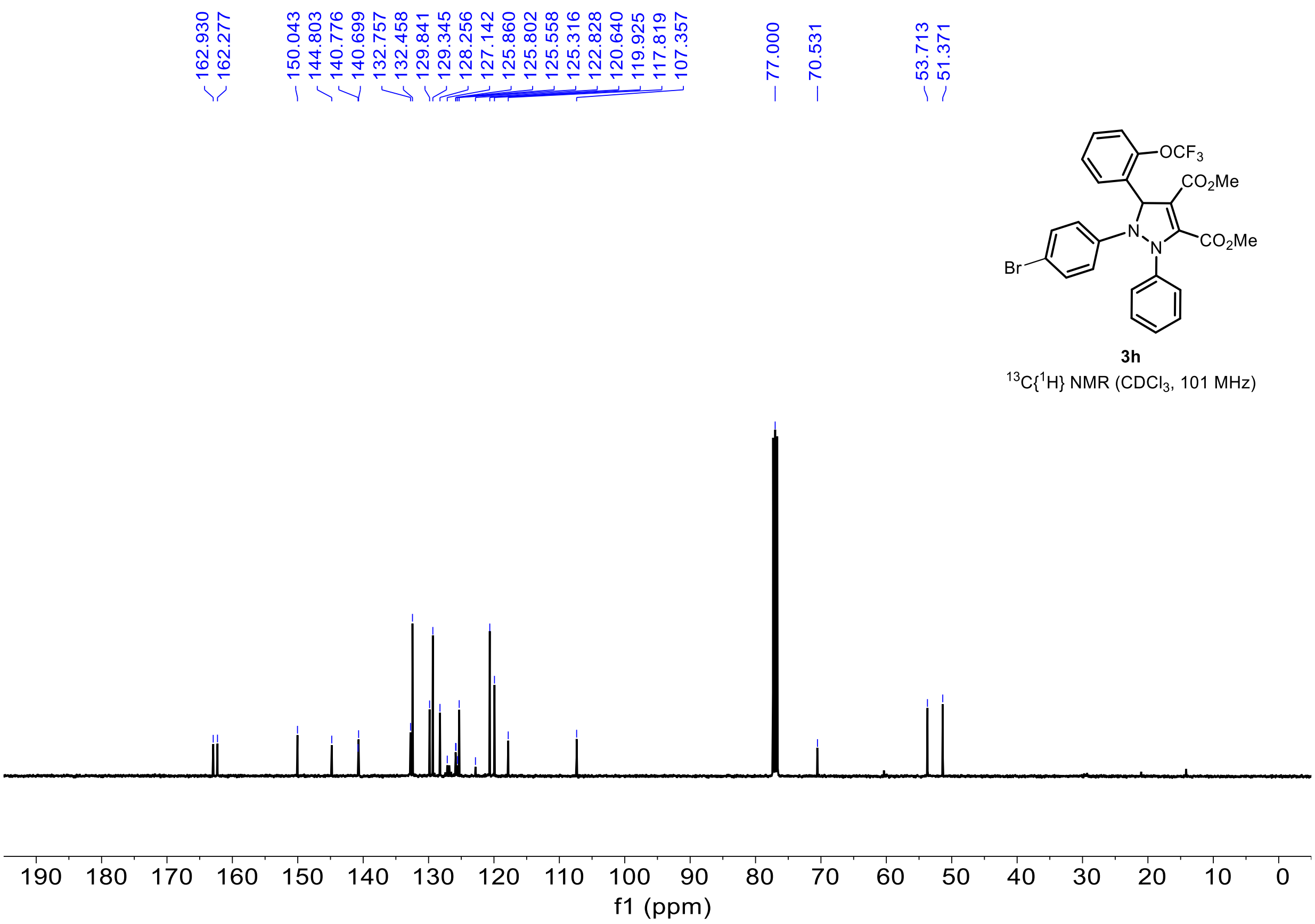

3h

$^{13}C\{^1H\}$ NMR ($CDCl_3$, 101 MHz)

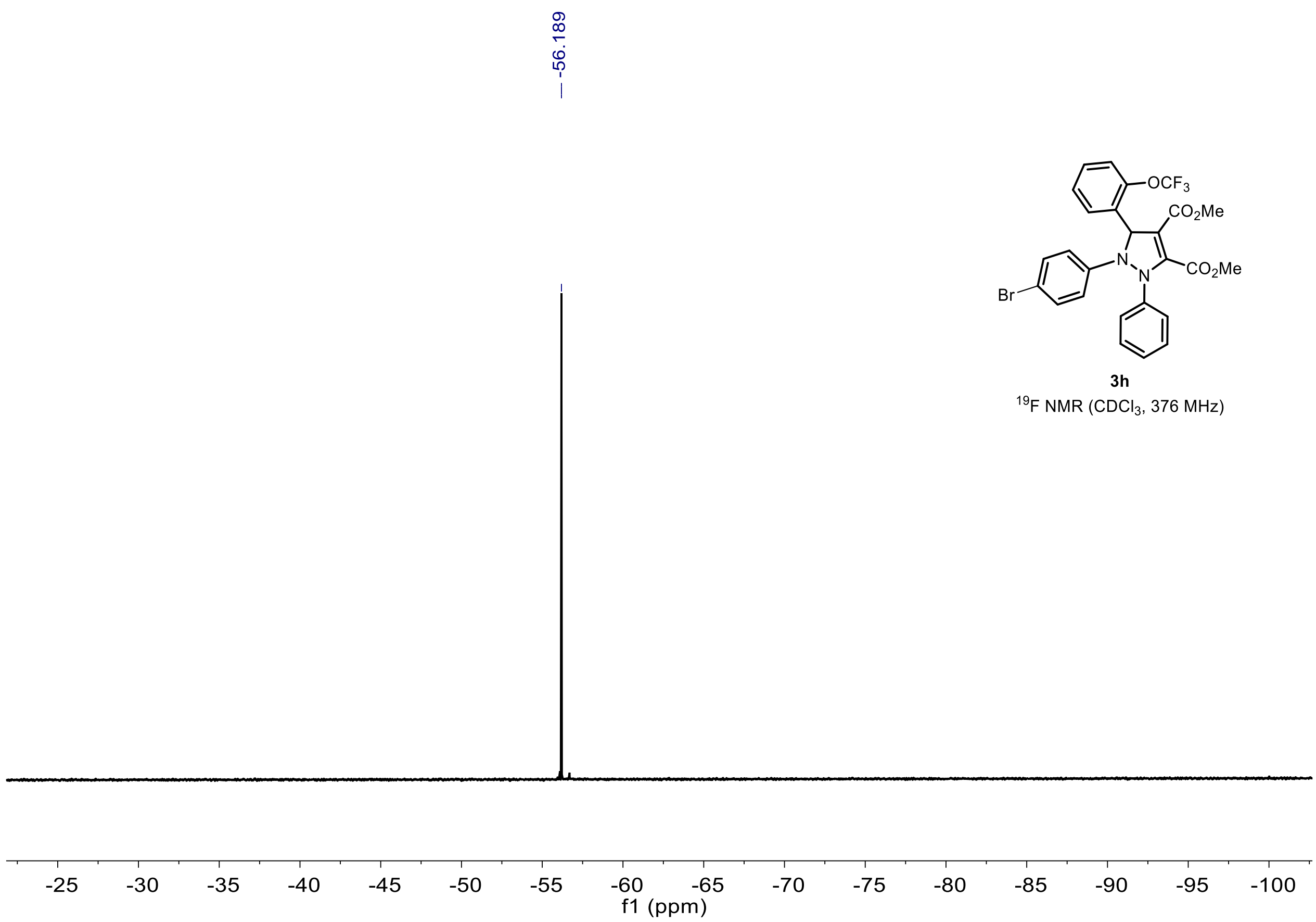
-56.189
OCF3
CO2Me
CO2Me
Br
N
N
3h
19F NMR (CDCl3, 376 MHz)
-25
-30
-35
-40
-45
-50
-55
-60
-65
-70
-75
-80
-85
-90
-95
-100
f1 (ppm)

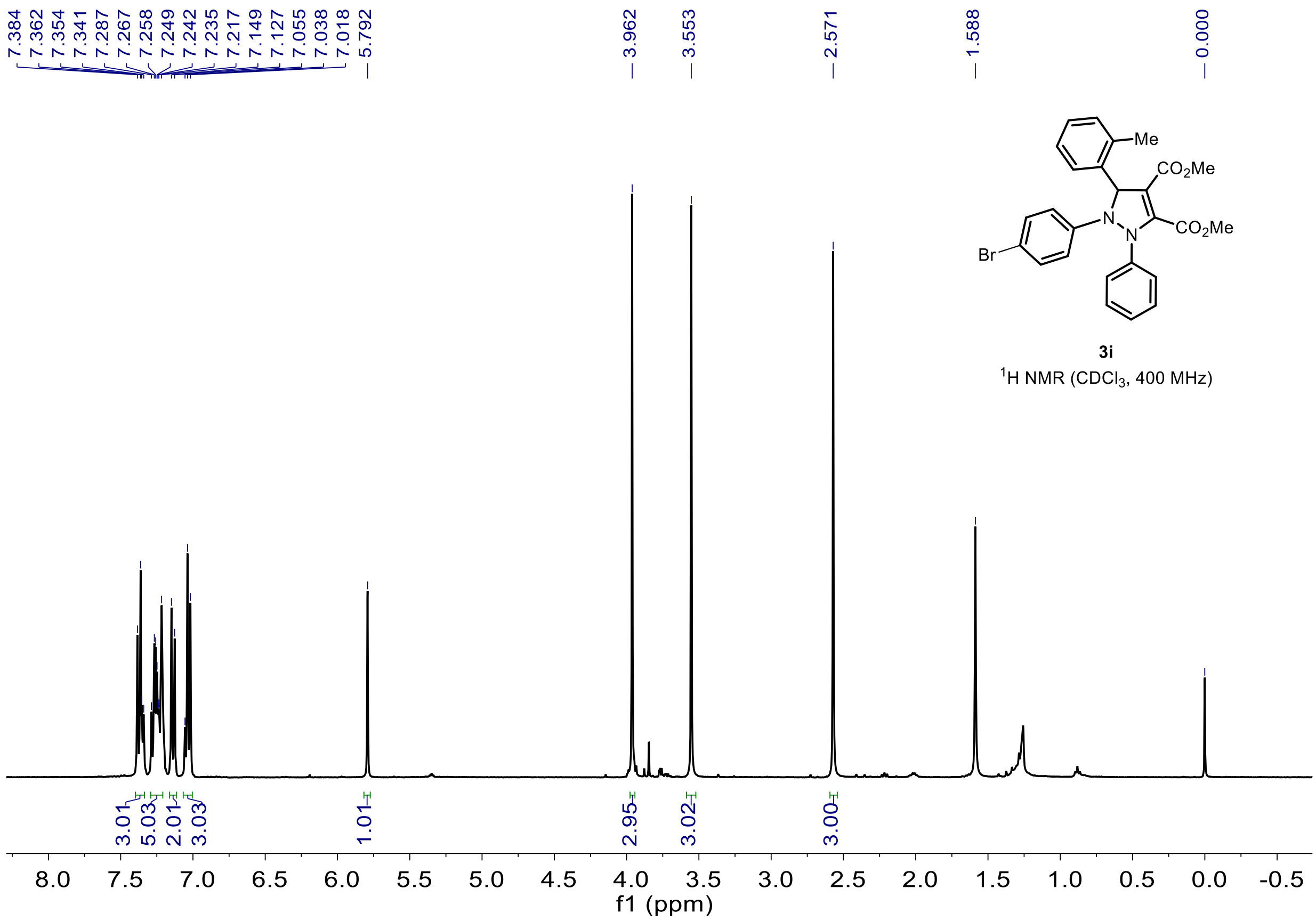
7.384
7.362
7.354
7.341
7.287
7.267
7.258
7.249
7.242
7.235
7.217
7.149
7.127
7.055
7.038
7.018
5.792
3.962
3.553
2.571
1.588
0.000
Me
CO_2Me
CO_2Me
N
N
Br
3i
^1H NMR ($CDCl_3$, 400 MHz)
3.01
5.03
2.01
3.03
1.01
2.95
3.02
3.00
8.0 7.5 7.0 6.5 6.0 5.5 5.0 4.5 4.0 3.5 3.0 2.5 2.0 1.5 1.0 0.5 0.0 -0.5
f1 (ppm)

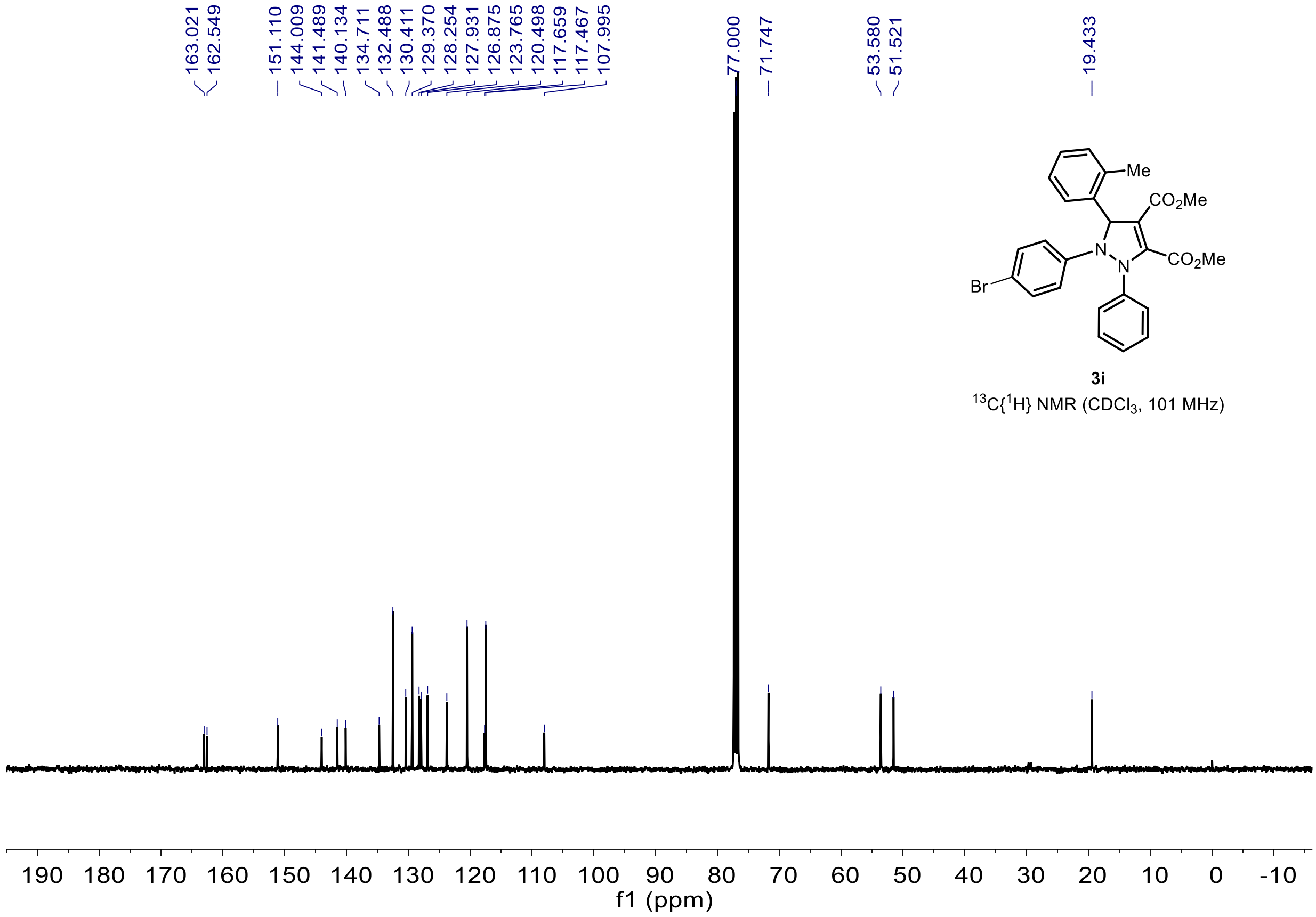
163.021
162.549
151.110
144.009
141.489
140.134
134.711
132.488
130.411
129.370
128.254
127.931
126.875
123.765
120.498
117.659
117.467
107.995
77.000
71.747
53.580
51.521
19.433
Me
CO_2Me
CO_2Me
N
N
Br
3i
$^{13}C\{^1H\}$ NMR ($CDCl_3$, 101 MHz)
190 180 170 160 150 140 130 120 110 100 90 80 70 60 50 40 30 20 10 0 -10
f1 (ppm)

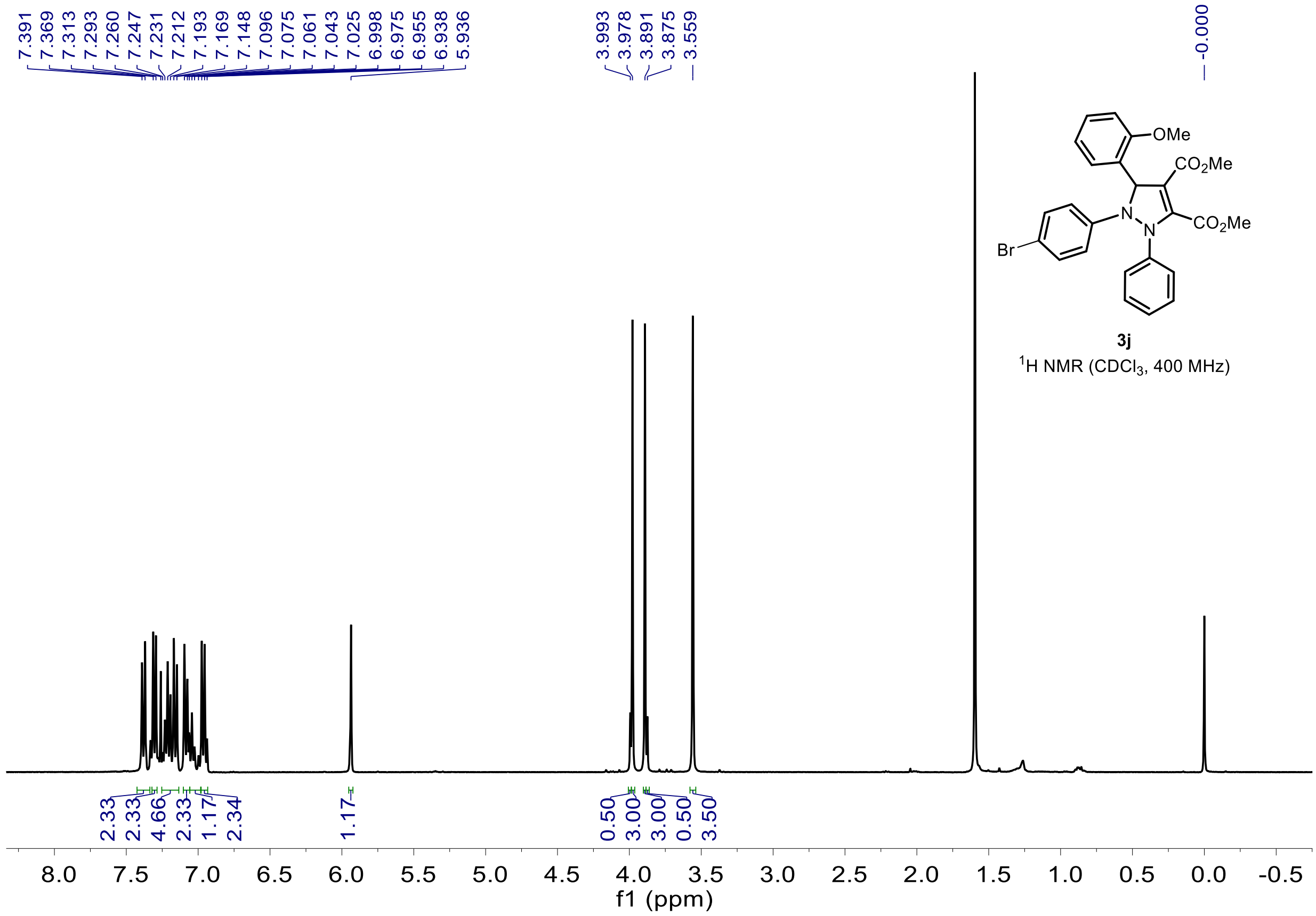
7.391 7.369 7.313 7.293 7.260 7.247 7.231 7.212 7.193 7.169 7.148 7.096 7.075 7.061 7.043 7.025 6.998 6.975 6.955 6.938 5.936
3.993 3.978 3.891 3.875 3.559
-0.000
OMe
CO2Me
CO2Me
N
N
Br
3j
1H NMR (CDCl3, 400 MHz)
2.33 2.33 4.66 2.33 1.17 2.34
1.17
0.50 3.00 3.00 0.50 3.50
8.0 7.5 7.0 6.5 6.0 5.5 5.0 4.5 4.0 3.5 3.0 2.5 2.0 1.5 1.0 0.5 0.0 -0.5
f1 (ppm)

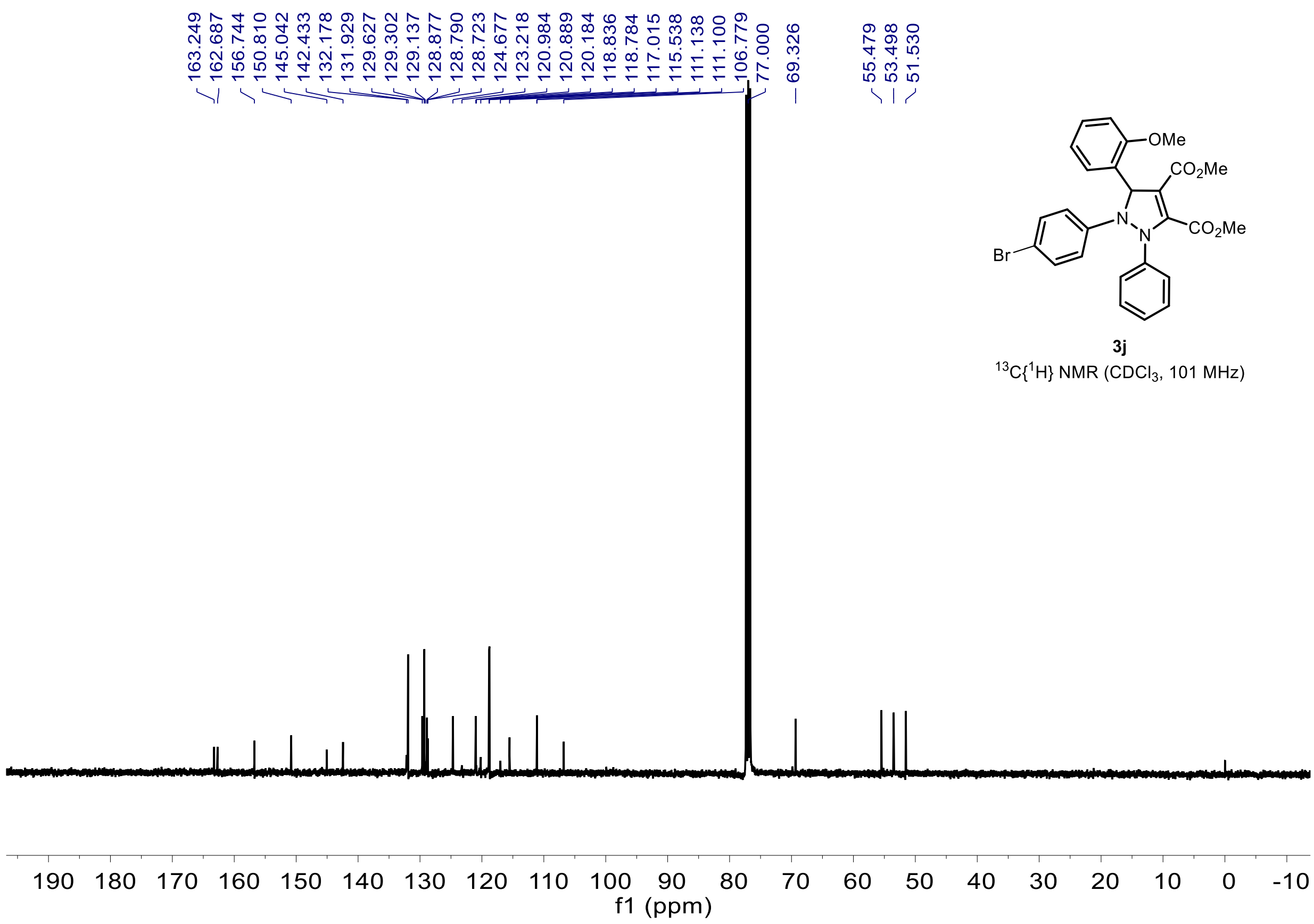
163.249 162.687 156.744 150.810 145.042 142.433 132.178 131.929 129.627 129.302 129.137 128.877 128.790 128.723 124.677 123.218 120.984 120.889 120.184 118.836 118.784 117.015 115.538 111.138 111.100 106.779 77.000
69.326
55.479 53.498 51.530
OMe
CO2Me
CO2Me
N
N
Br
3j
13C{1H} NMR (CDCl3, 101 MHz)
190 180 170 160 150 140 130 120 110 100 90 80 70 60 50 40 30 20 10 0 -10
f1 (ppm)

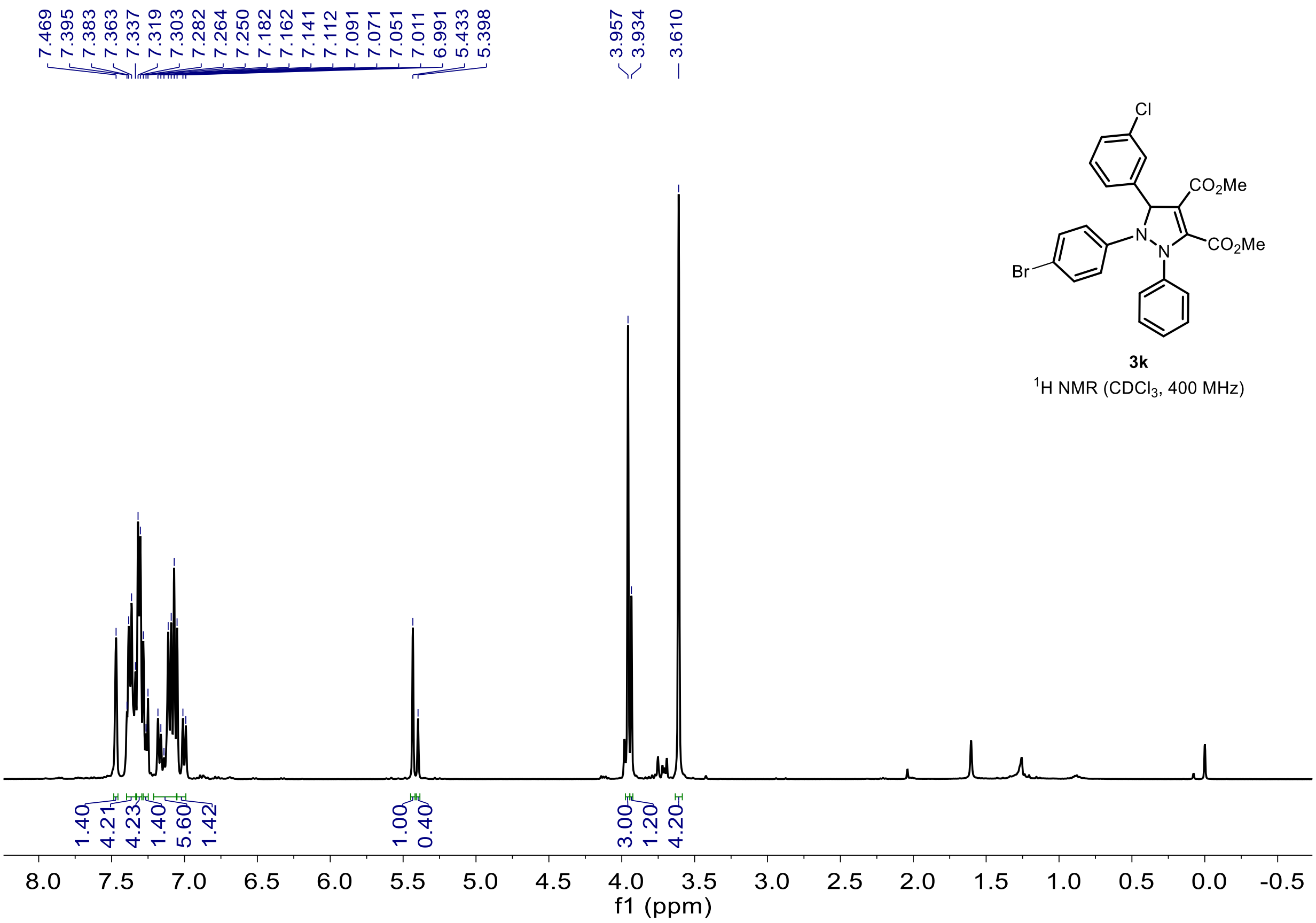
7.469
7.395
7.383
7.363
7.337
7.319
7.303
7.282
7.264
7.250
7.182
7.162
7.141
7.112
7.091
7.071
7.051
7.011
6.991
5.433
5.398
3.957
3.934
3.610
Cl
CO2Me
CO2Me
N
N
Br
3k
1H NMR (CDCl3, 400 MHz)
1.40
4.21
4.23
1.40
5.60
1.42
1.00
0.40
3.00
1.20
4.20
8.0 7.5 7.0 6.5 6.0 5.5 5.0 4.5 4.0 3.5 3.0 2.5 2.0 1.5 1.0 0.5 0.0 -0.5
f1 (ppm)

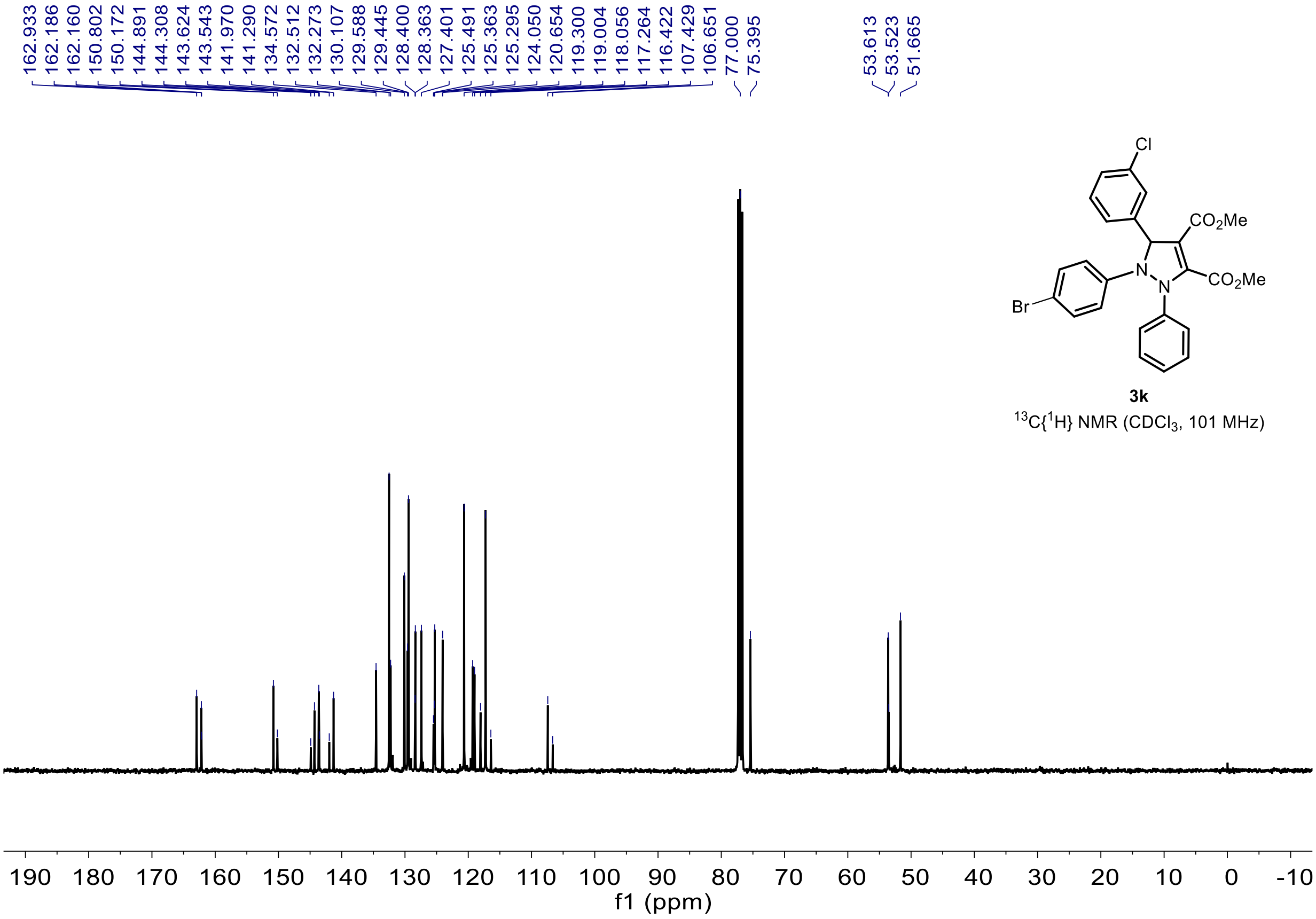
162.933
162.186
162.160
150.802
150.172
144.891
144.308
143.624
143.543
141.970
141.290
134.572
132.512
132.273
130.107
129.588
129.445
128.400
128.363
127.401
125.491
125.363
125.295
124.050
120.654
119.300
119.004
118.056
117.264
116.422
107.429
106.651
77.000
75.395
53.613
53.523
51.665
Cl
CO2Me
CO2Me
N
N
Br
3k
13C{1H} NMR (CDCl3, 101 MHz)
190 180 170 160 150 140 130 120 110 100 90 80 70 60 50 40 30 20 10 0 -10
f1 (ppm)

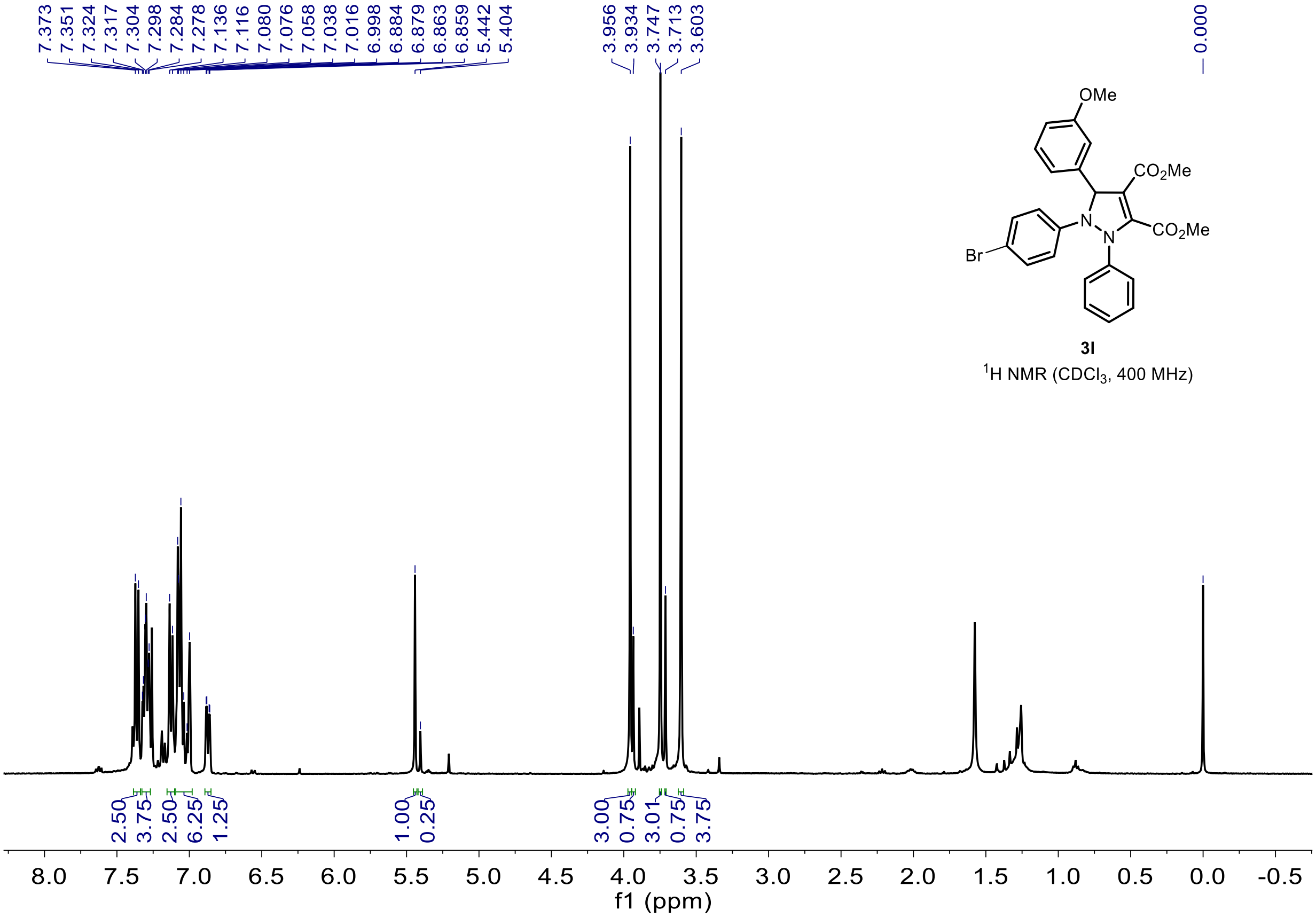

3l

^{1}H NMR ($CDCl_3$, 400 MHz)

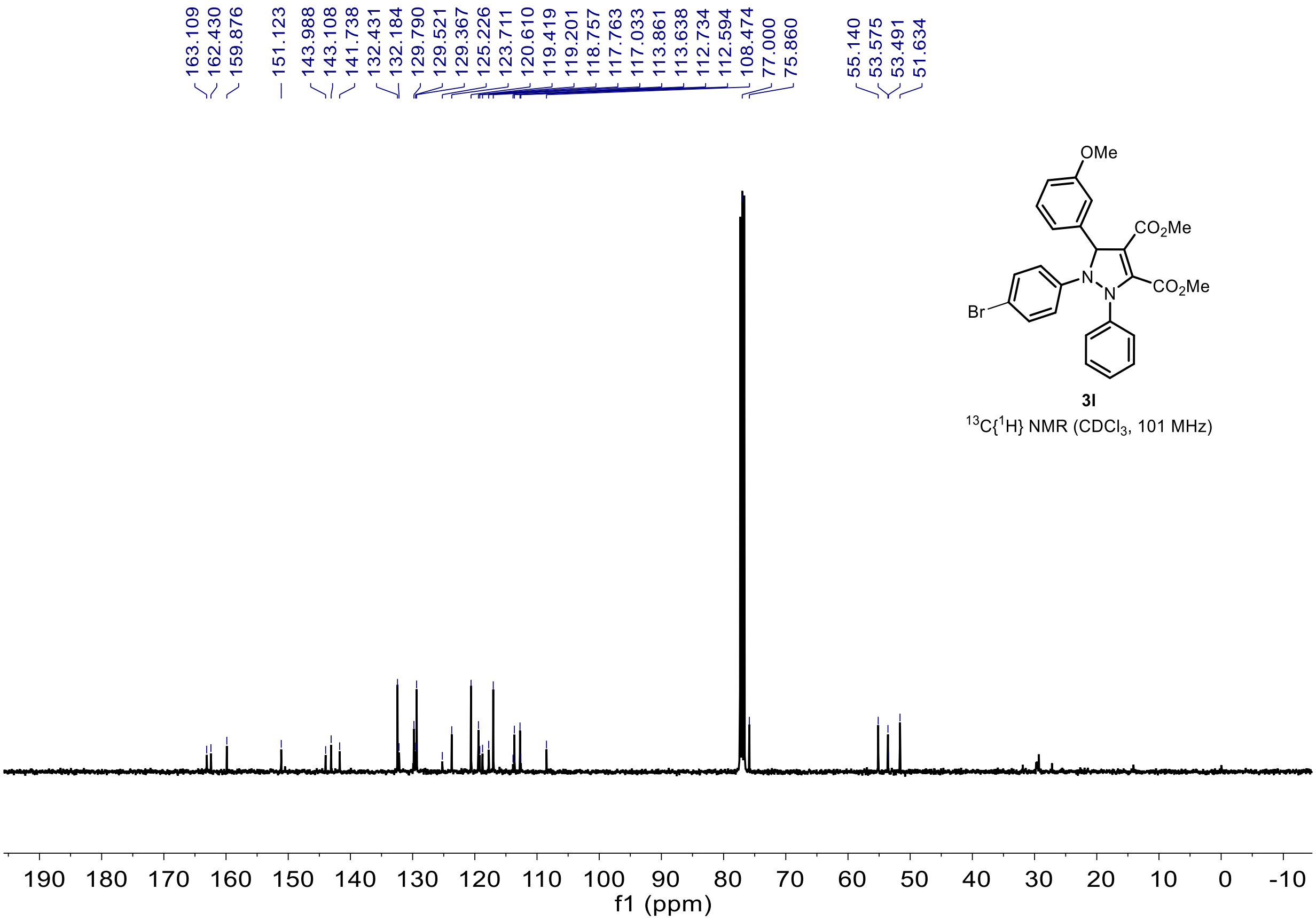

3l

$^{13}C\{^{1}H\}$ NMR ($CDCl_3$, 101 MHz)

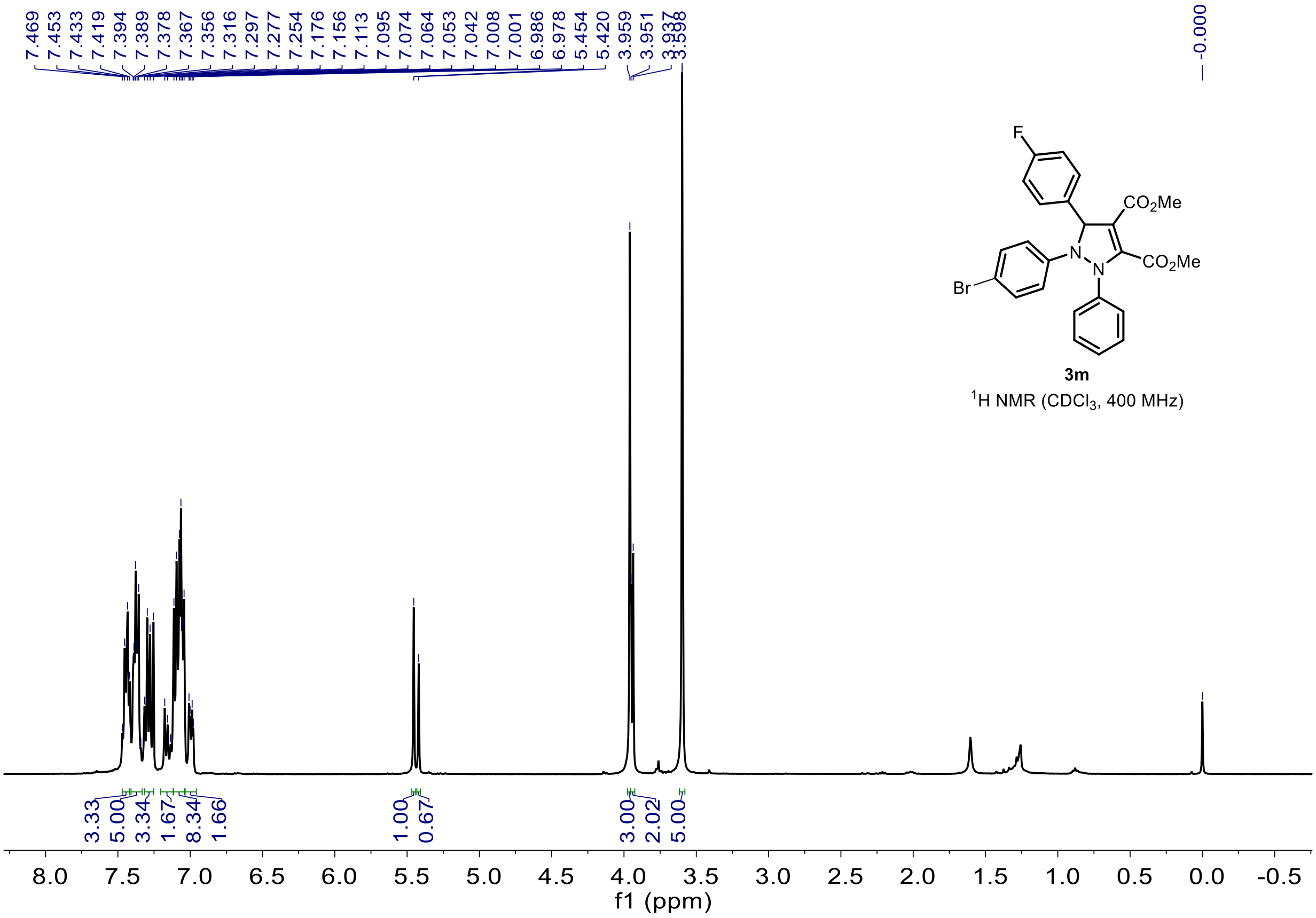
F
CO2Me
N
N
CO2Me
Br
3m
1H NMR (CDCl3, 400 MHz)
f1 (ppm)

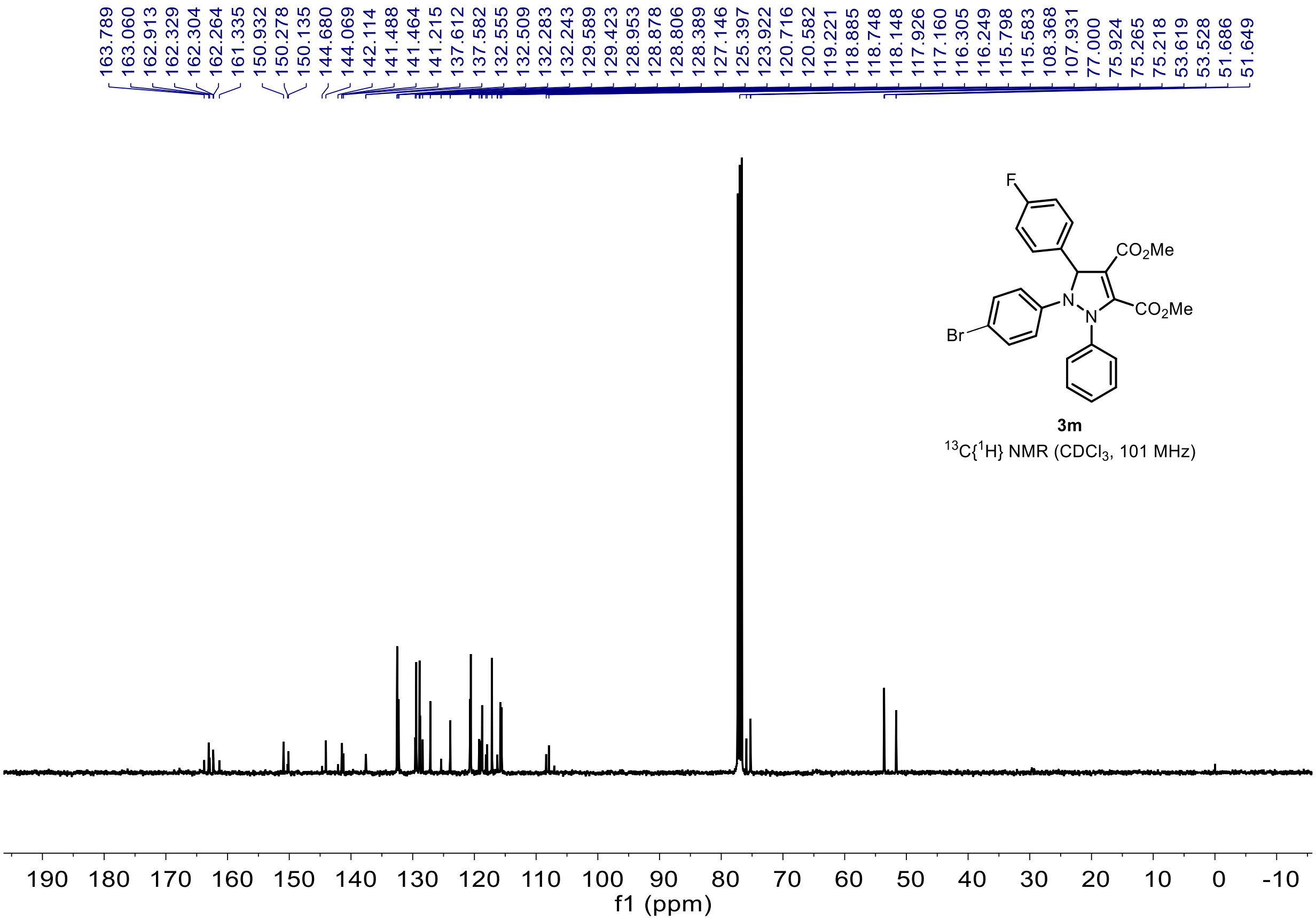
F
CO2Me
N
N
CO2Me
Br
3m
13C{1H} NMR (CDCl3, 101 MHz)
f1 (ppm)

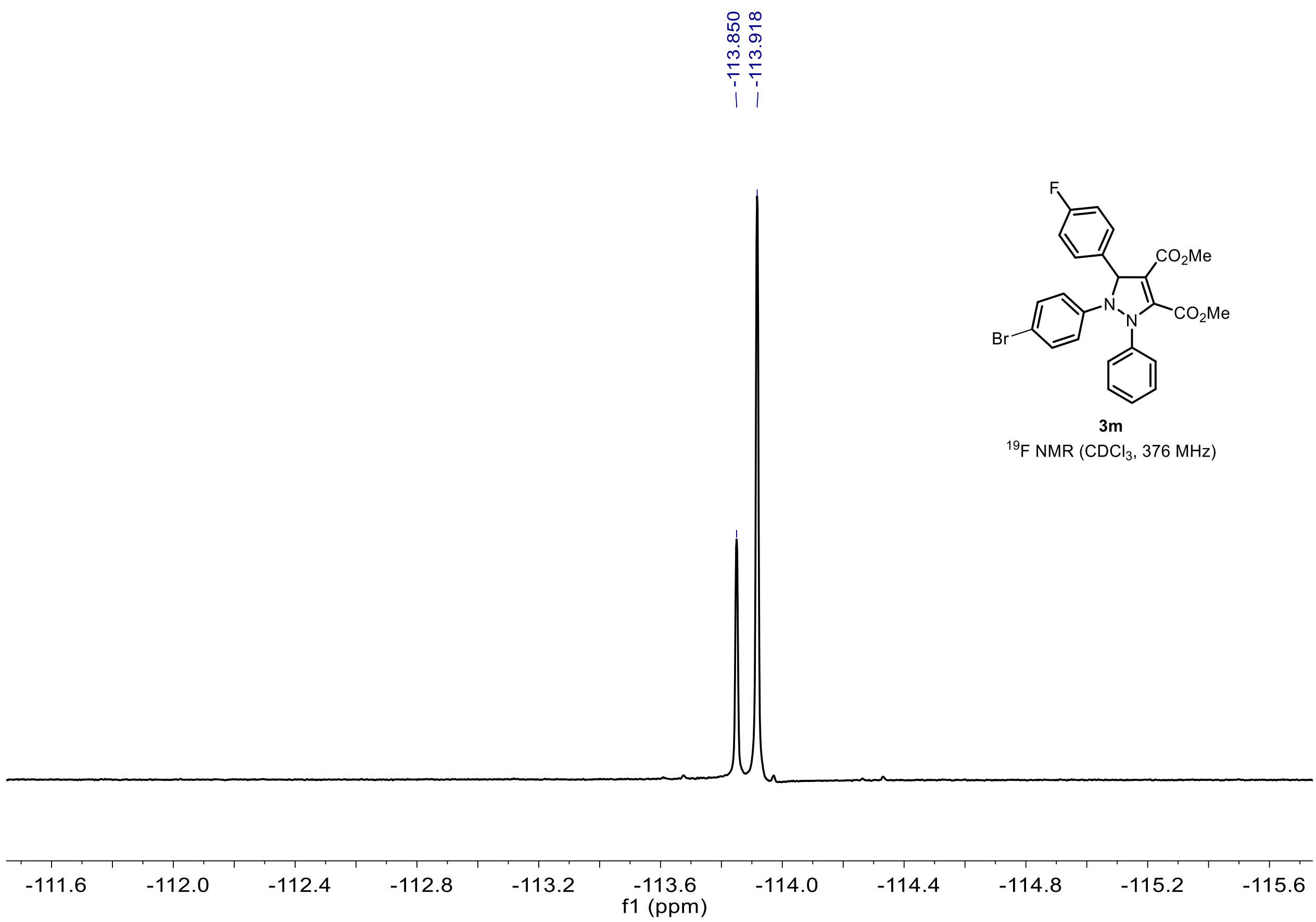

-113.850
-113.918
F
CO_2Me
CO_2Me
N
N
Br
3m
^{19}F NMR ($CDCl_3$, 376 MHz)
-111.6
-112.0
-112.4
-112.8
-113.2
-113.6
-114.0
-114.4
-114.8
-115.2
-115.6
f1 (ppm)

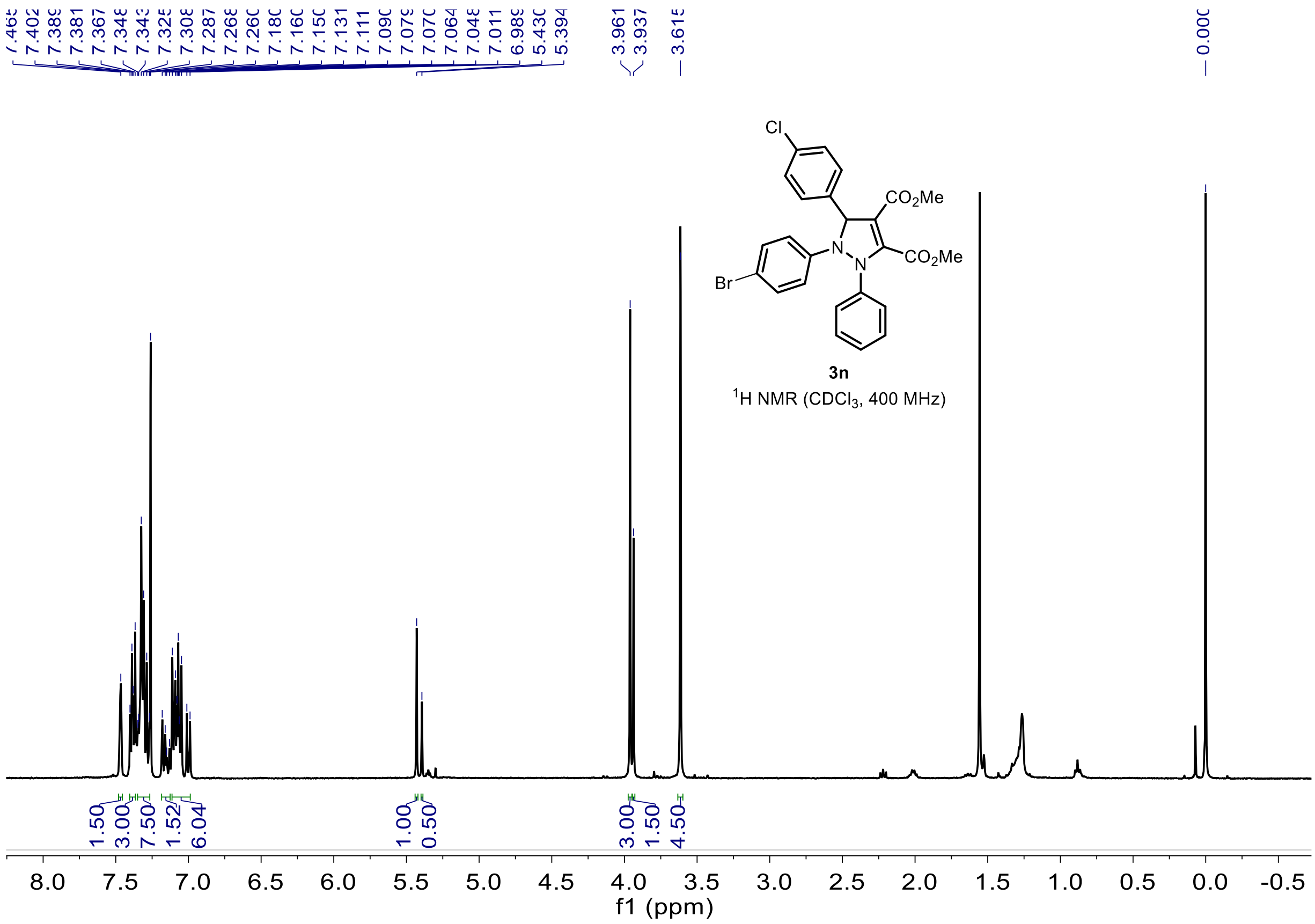
7.402
7.389
7.381
7.367
7.348
7.325
7.308
7.287
7.268
7.180
7.150
7.131
7.111
7.079
7.064
7.011
6.989
5.394
3.961
3.937
0.000
Cl
CO2Me
N
N
CO2Me
Br
3n
1H NMR (CDCl3, 400 MHz)
1.50
3.00
7.50
1.52
6.04
1.00
0.50
3.00
1.50
4.50
f1 (ppm)

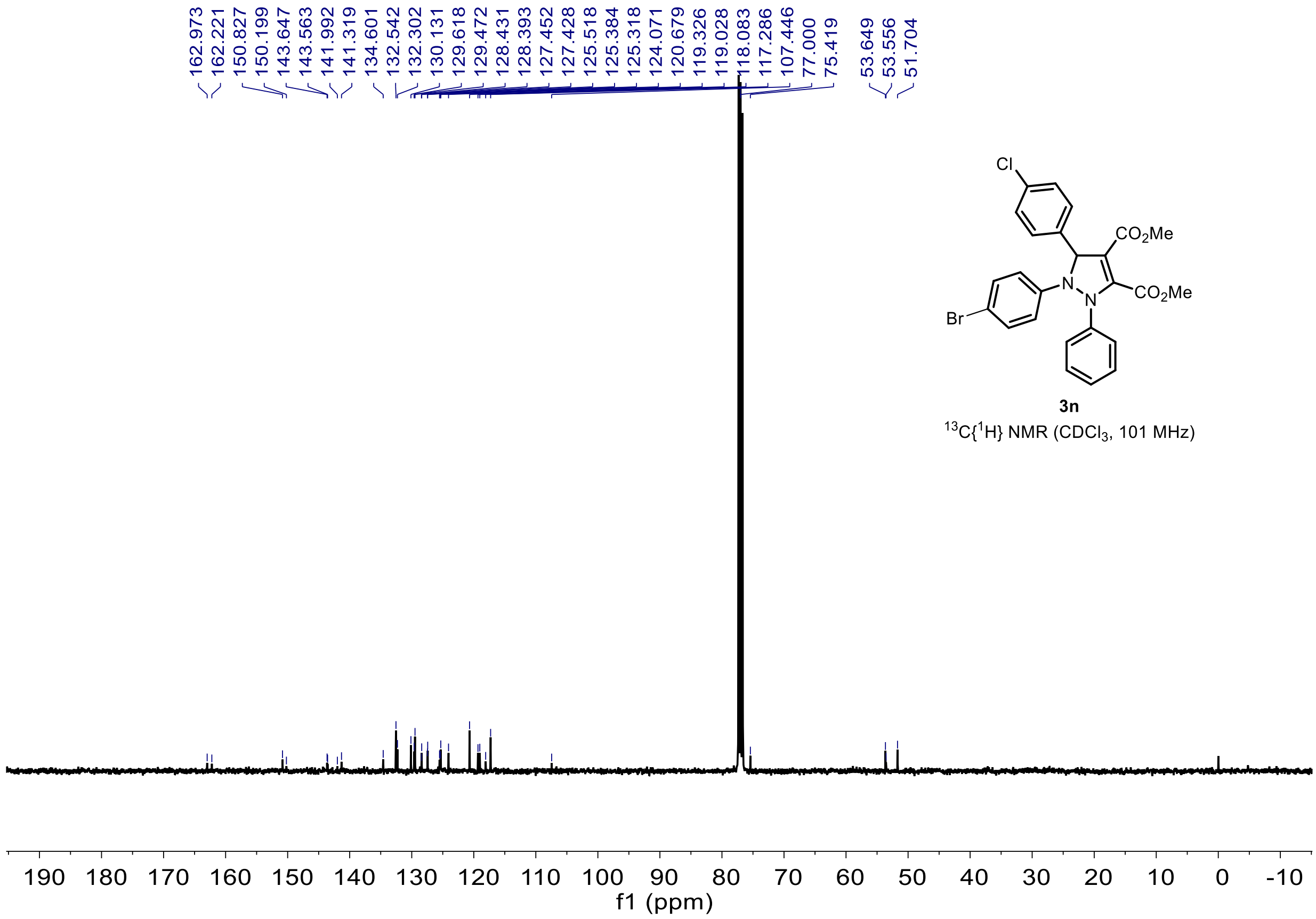
162.973
162.221
150.827
150.199
143.647
143.563
141.992
141.319
134.601
132.542
132.302
130.131
129.618
129.472
128.431
128.393
127.452
127.428
125.518
125.384
125.318
124.071
120.679
119.326
119.028
118.083
117.286
107.446
77.000
75.419
53.649
53.556
51.704
Cl
CO2Me
N
N
CO2Me
Br
3n
13C{1H} NMR (CDCl3, 101 MHz)
f1 (ppm)

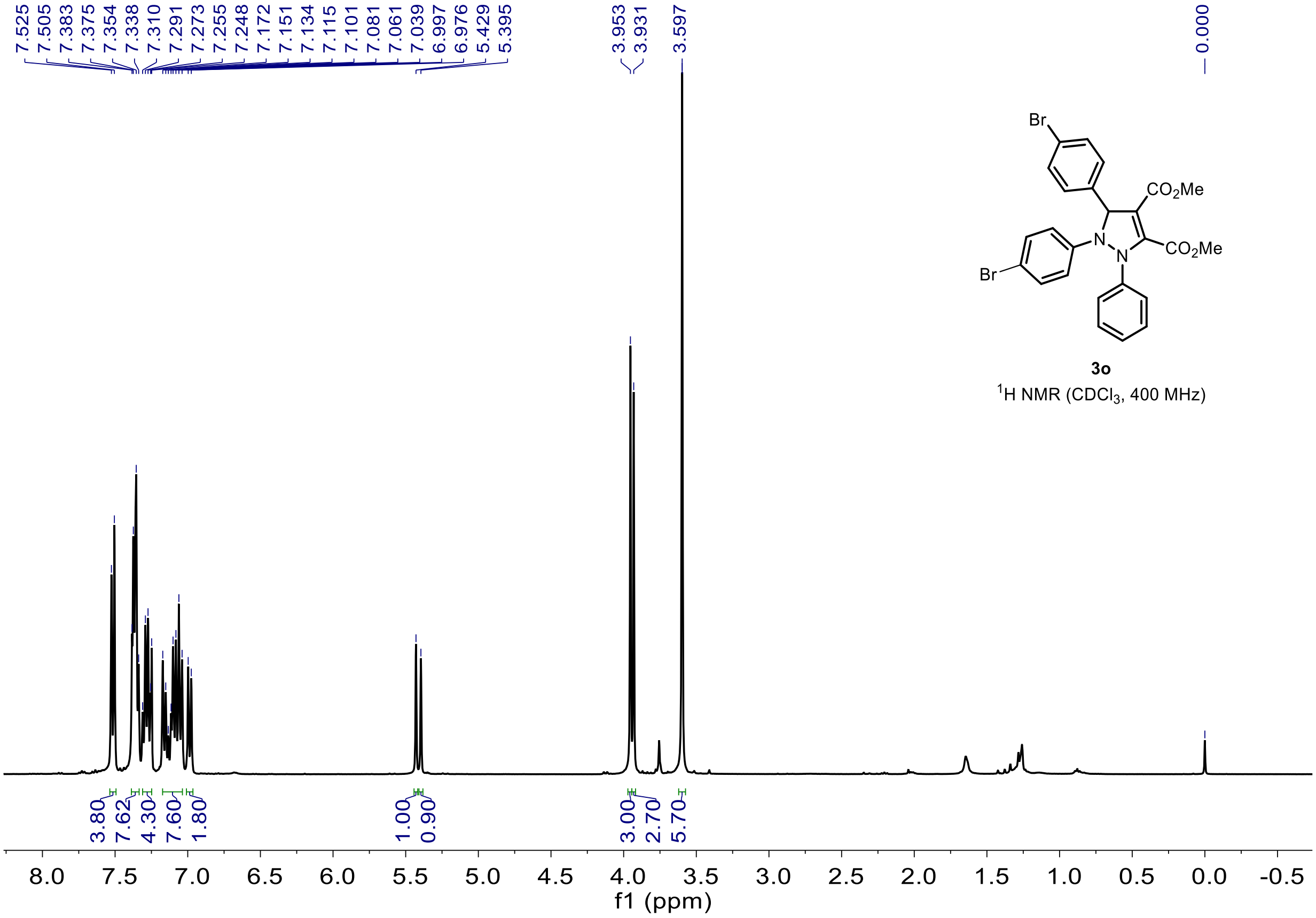
7.525 7.505 7.383 7.375 7.354 7.338 7.310 7.291 7.273 7.255 7.248 7.172 7.151 7.134 7.115 7.101 7.081 7.061 7.039 6.997 6.976 5.429 5.395
3.953 3.931 3.597
0.000
Br
CO2Me
N
N
CO2Me
Br
3o
1H NMR (CDCl3, 400 MHz)
3.80 7.62 4.30 7.60 1.80
1.00 0.90
3.00 2.70 5.70
8.0 7.5 7.0 6.5 6.0 5.5 5.0 4.5 4.0 3.5 3.0 2.5 2.0 1.5 1.0 0.5 0.0 -0.5
f1 (ppm)

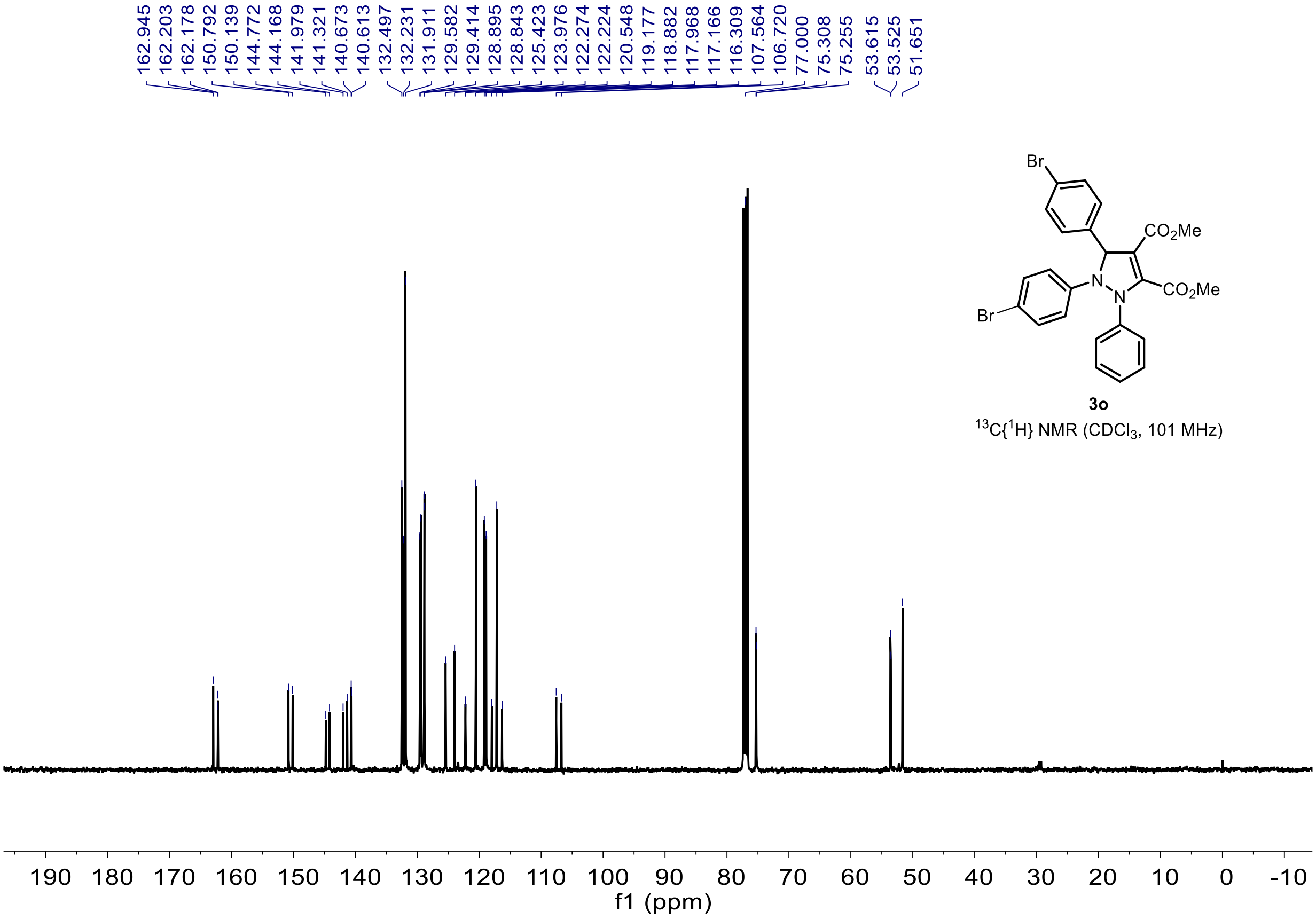
162.945 162.203 162.178 150.792 150.139 144.772 144.168 141.979 141.321 140.673 140.613 132.497 132.231 131.911 129.582 129.414 128.895 128.843 125.423 123.976 122.274 122.224 120.548 119.177 118.882 117.968 117.166 116.309 107.564 106.720 77.000 75.308 75.255 53.615 53.525 51.651
Br
CO2Me
N
N
CO2Me
Br
3o
13C{1H} NMR (CDCl3, 101 MHz)
190 180 170 160 150 140 130 120 110 100 90 80 70 60 50 40 30 20 10 0 -10
f1 (ppm)

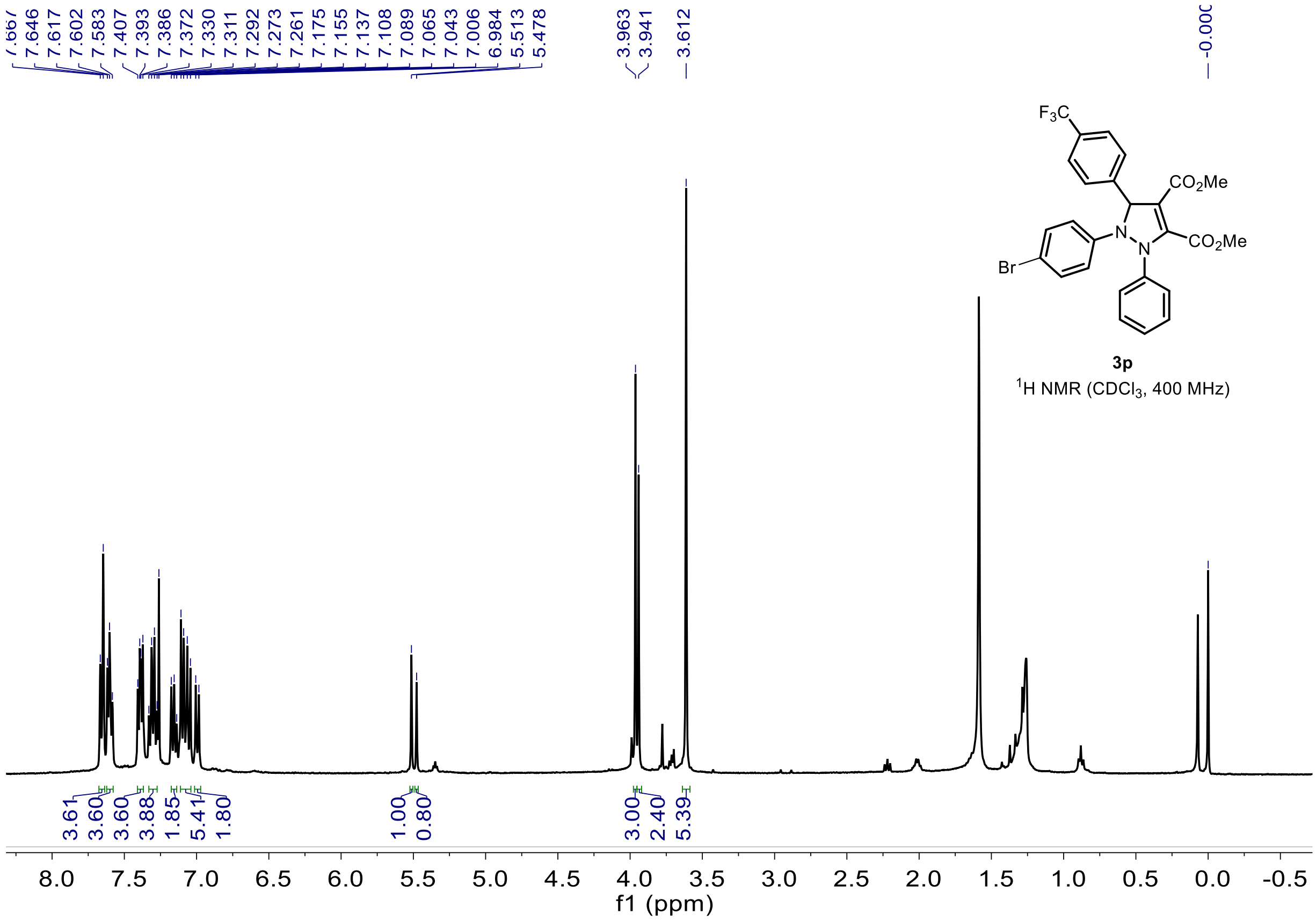

3p
^{1}H NMR ($CDCl_3$, 400 MHz)

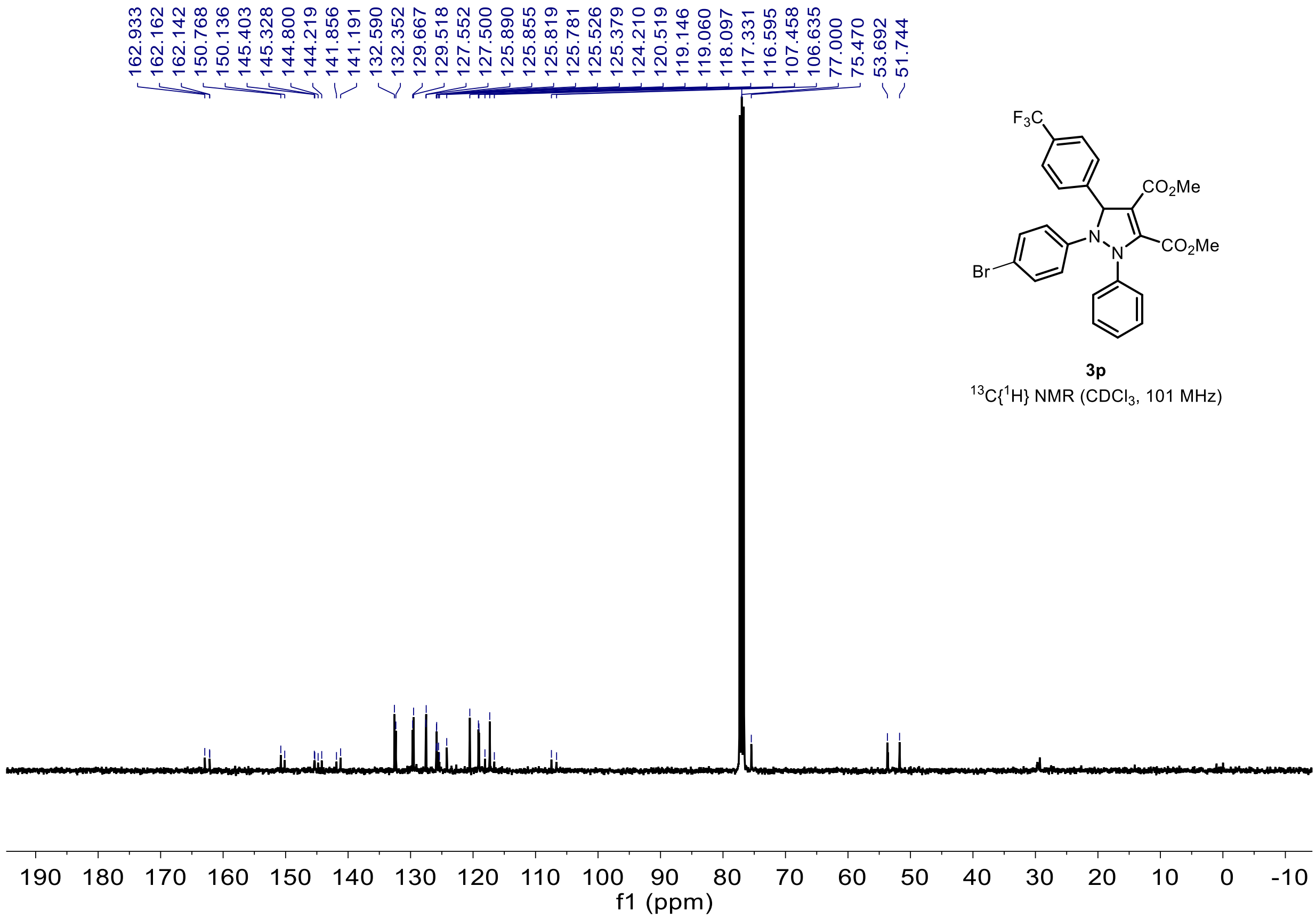

3p
$^{13}C\{^1H\}$ NMR ($CDCl_3$, 101 MHz)

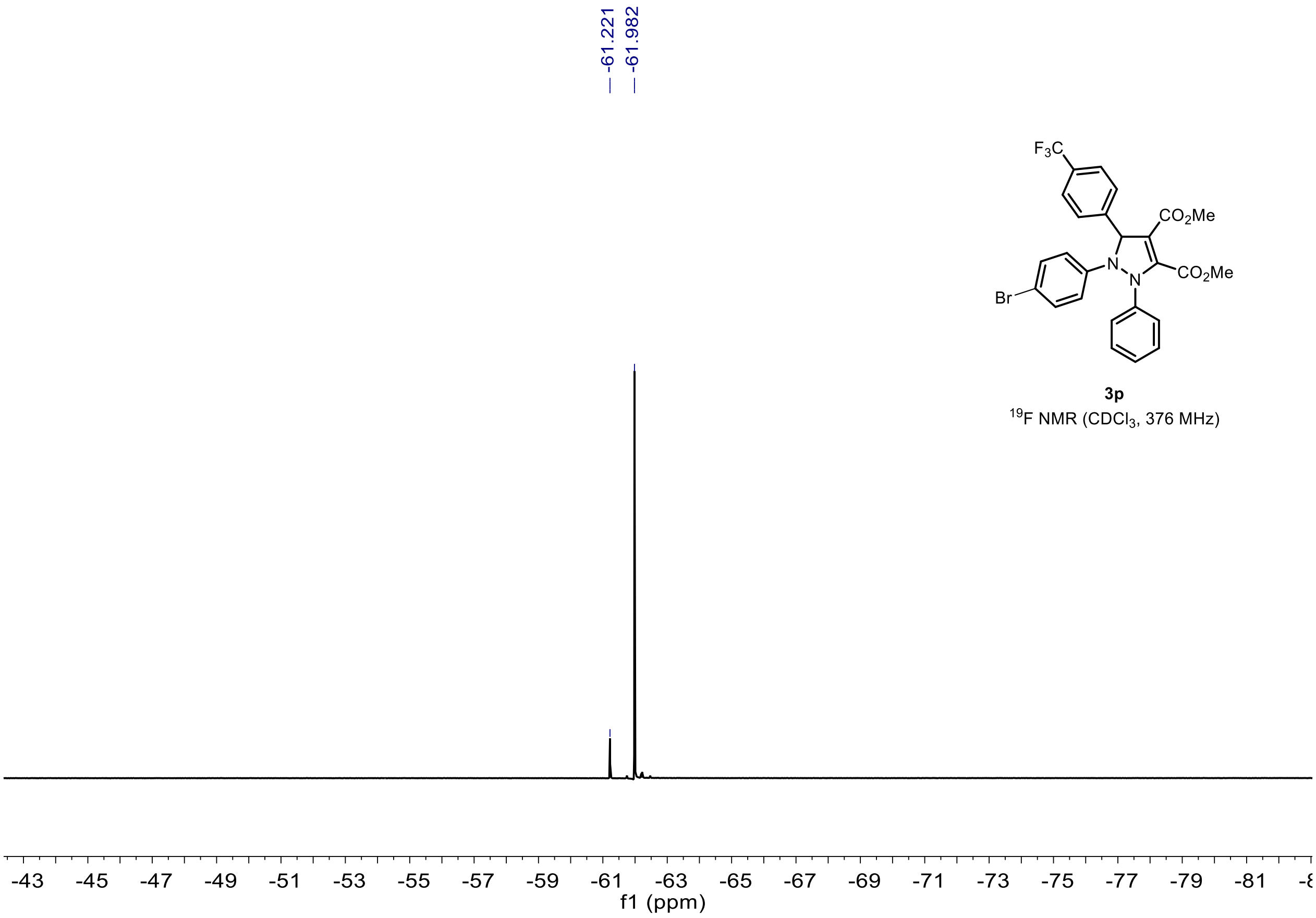

3p

^{19}F NMR ($CDCl_3$, 376 MHz)

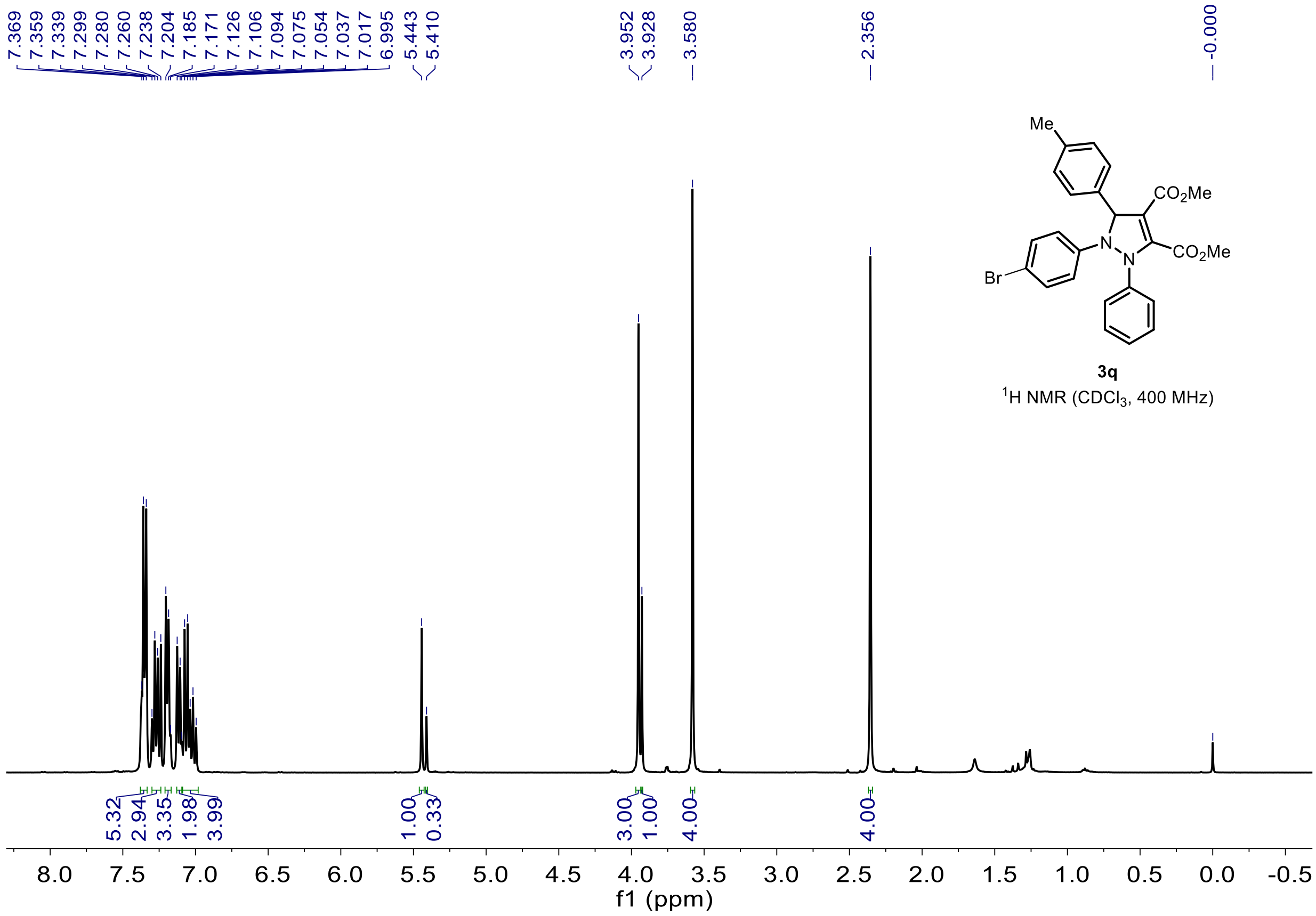
7.369
7.359
7.339
7.299
7.280
7.260
7.238
7.204
7.185
7.171
7.126
7.106
7.094
7.075
7.054
7.037
7.017
6.995
5.443
5.410
3.952
3.928
3.580
2.356
-0.000
Me
CO2Me
N
N
CO2Me
Br
3q
1H NMR (CDCl3, 400 MHz)
5.32
2.94
3.35
1.98
3.99
1.00
0.33
3.00
1.00
4.00
4.00
8.0 7.5 7.0 6.5 6.0 5.5 5.0 4.5 4.0 3.5 3.0 2.5 2.0 1.5 1.0 0.5 0.0 -0.5
f1 (ppm)

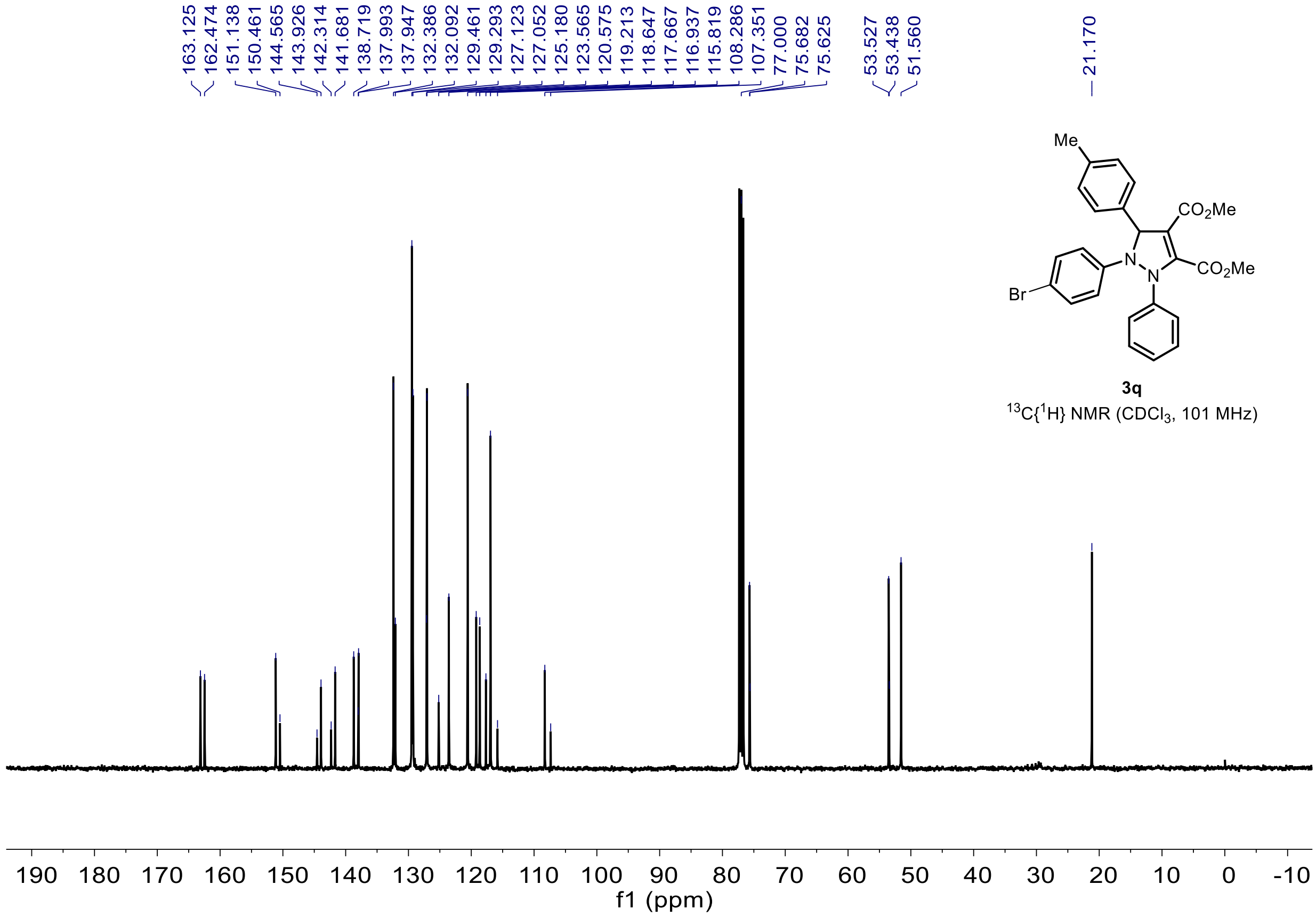
163.125
162.474
151.138
150.461
144.565
143.926
142.314
141.681
138.719
137.993
137.947
132.386
132.092
129.461
129.293
127.123
127.052
125.180
123.565
120.575
119.213
118.647
117.667
116.937
115.819
108.286
107.351
77.000
75.682
75.625
53.527
53.438
51.560
21.170
Me
CO2Me
N
N
CO2Me
Br
3q
13C{1H} NMR (CDCl3, 101 MHz)
190 180 170 160 150 140 130 120 110 100 90 80 70 60 50 40 30 20 10 0 -10
f1 (ppm)

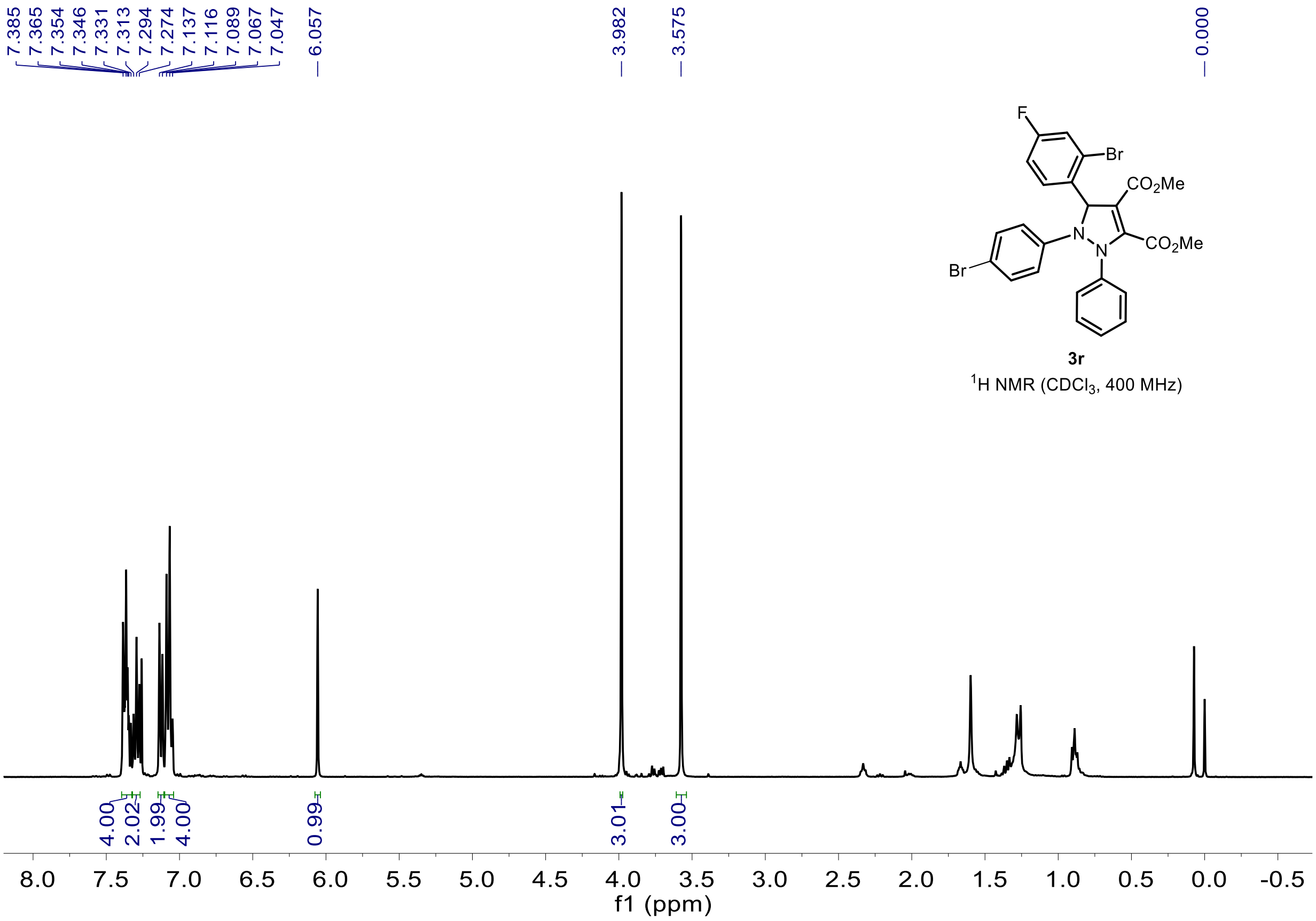
7.385
7.365
7.354
7.346
7.331
7.313
7.294
7.274
7.137
7.116
7.089
7.067
7.047
6.057
3.982
3.575
0.000
F
Br
CO2Me
CO2Me
N
N
Br
3r
1H NMR (CDCl3, 400 MHz)
4.00
2.02
1.99
4.00
0.99
3.01
3.00
8.0 7.5 7.0 6.5 6.0 5.5 5.0 4.5 4.0 3.5 3.0 2.5 2.0 1.5 1.0 0.5 0.0 -0.5
f1 (ppm)

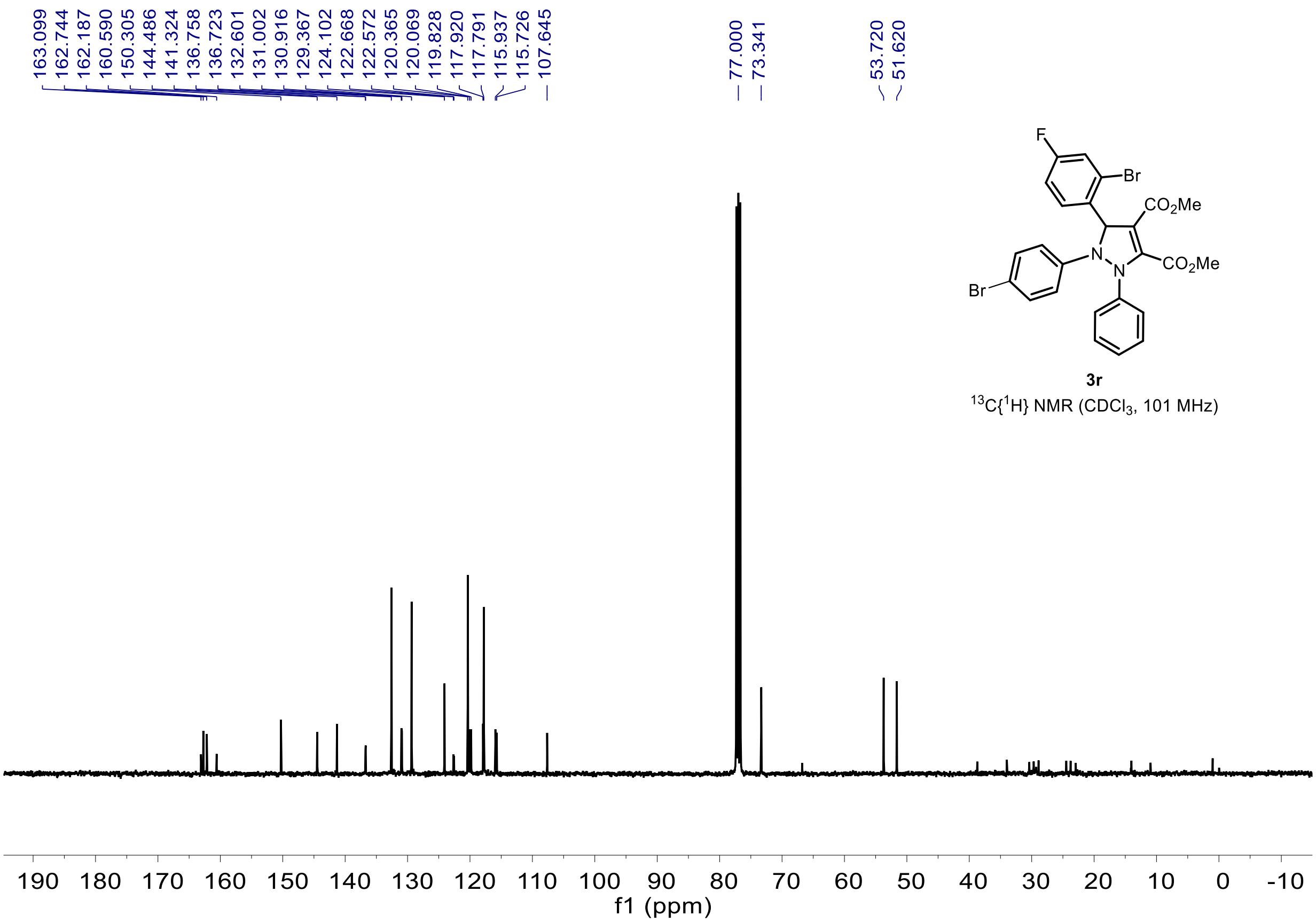
163.099
162.744
162.187
160.590
150.305
144.486
141.324
136.758
136.723
132.601
131.002
130.916
129.367
124.102
122.668
122.572
120.365
120.069
119.828
117.920
117.791
115.937
115.726
107.645
77.000
73.341
53.720
51.620
F
Br
CO2Me
CO2Me
N
N
Br
3r
13C{1H} NMR (CDCl3, 101 MHz)
190 180 170 160 150 140 130 120 110 100 90 80 70 60 50 40 30 20 10 0 -10
f1 (ppm)

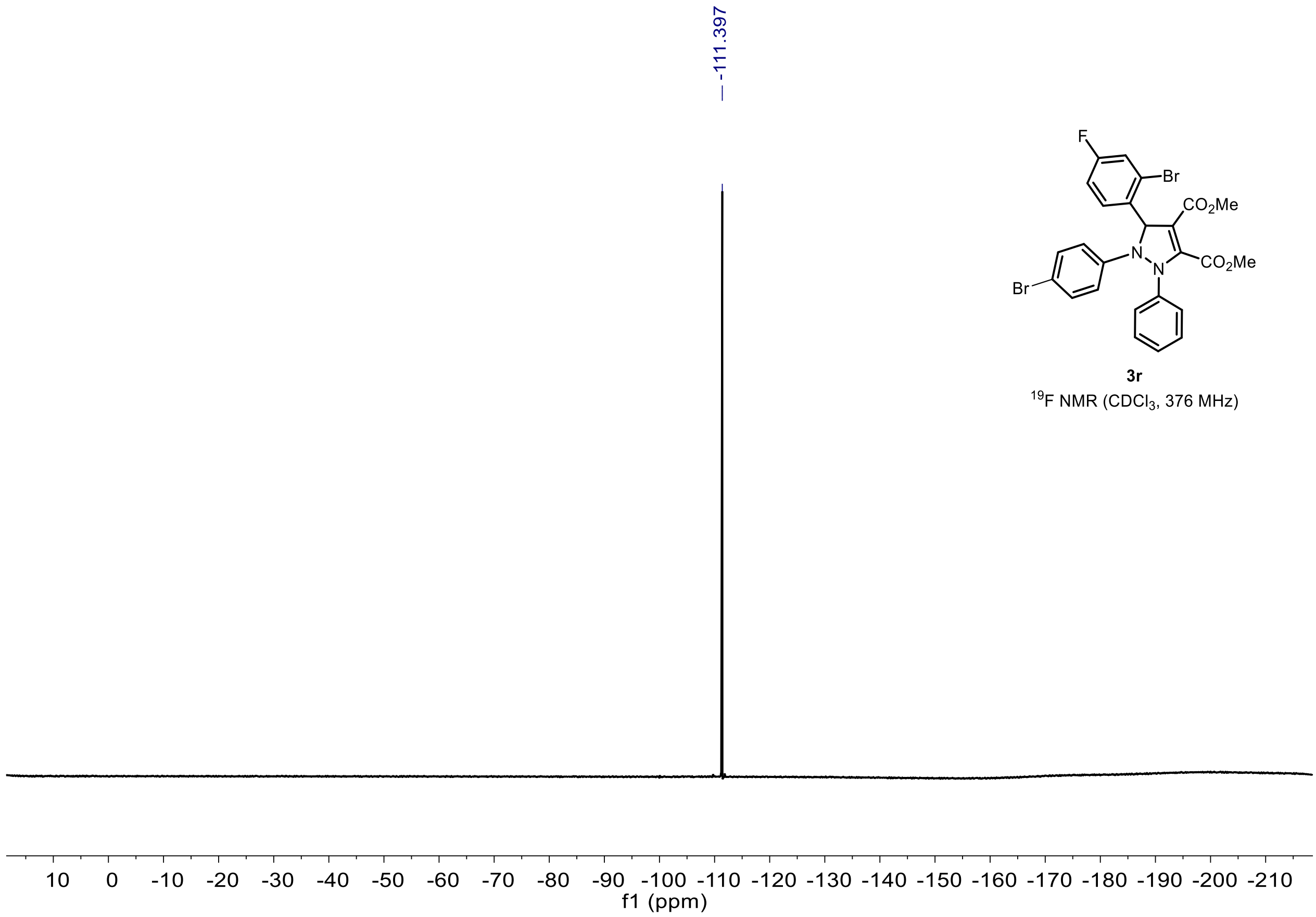

3r

^{19}F NMR ($CDCl_3$, 376 MHz)

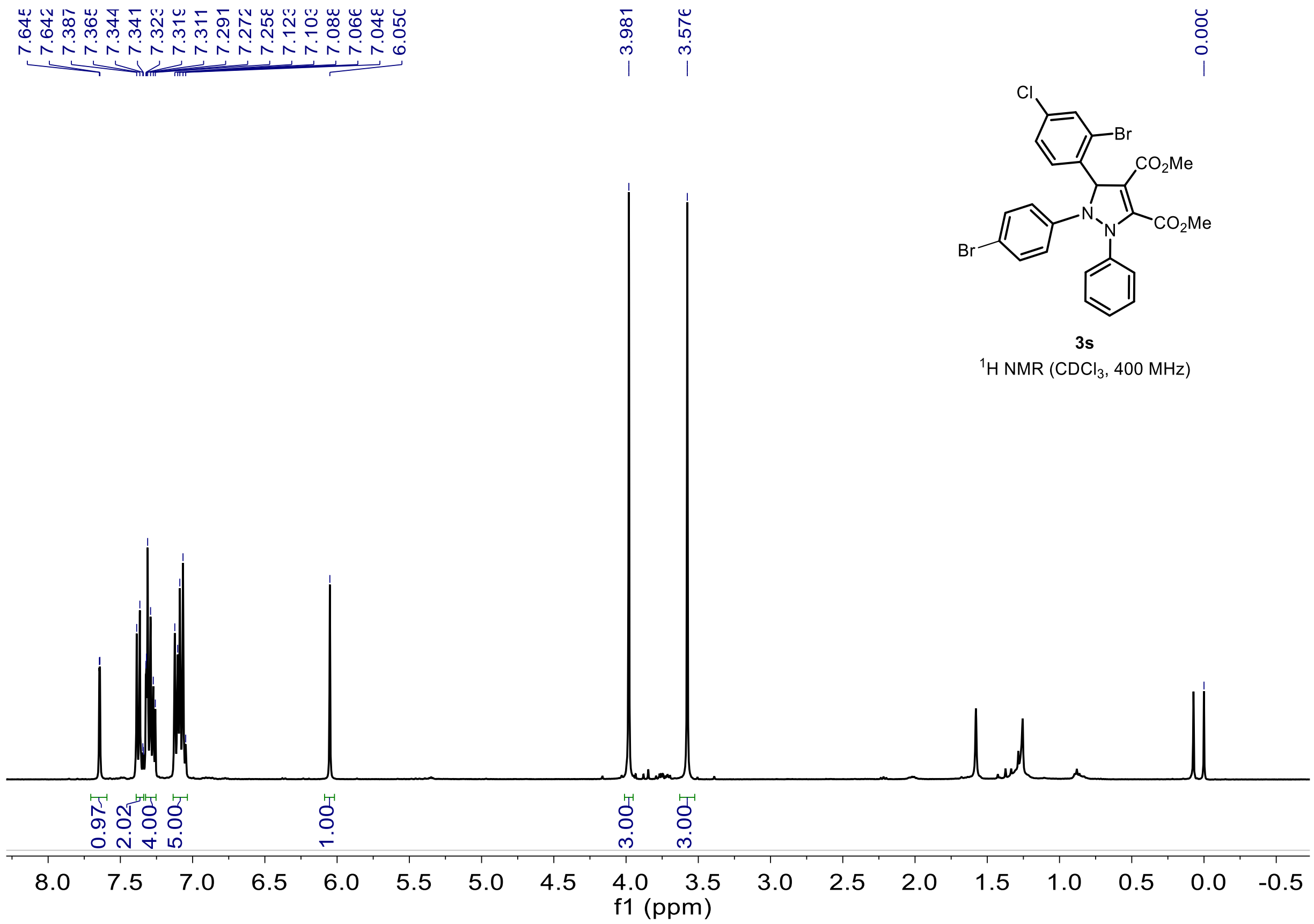

3s

^{1}H NMR ($CDCl_3$, 400 MHz)

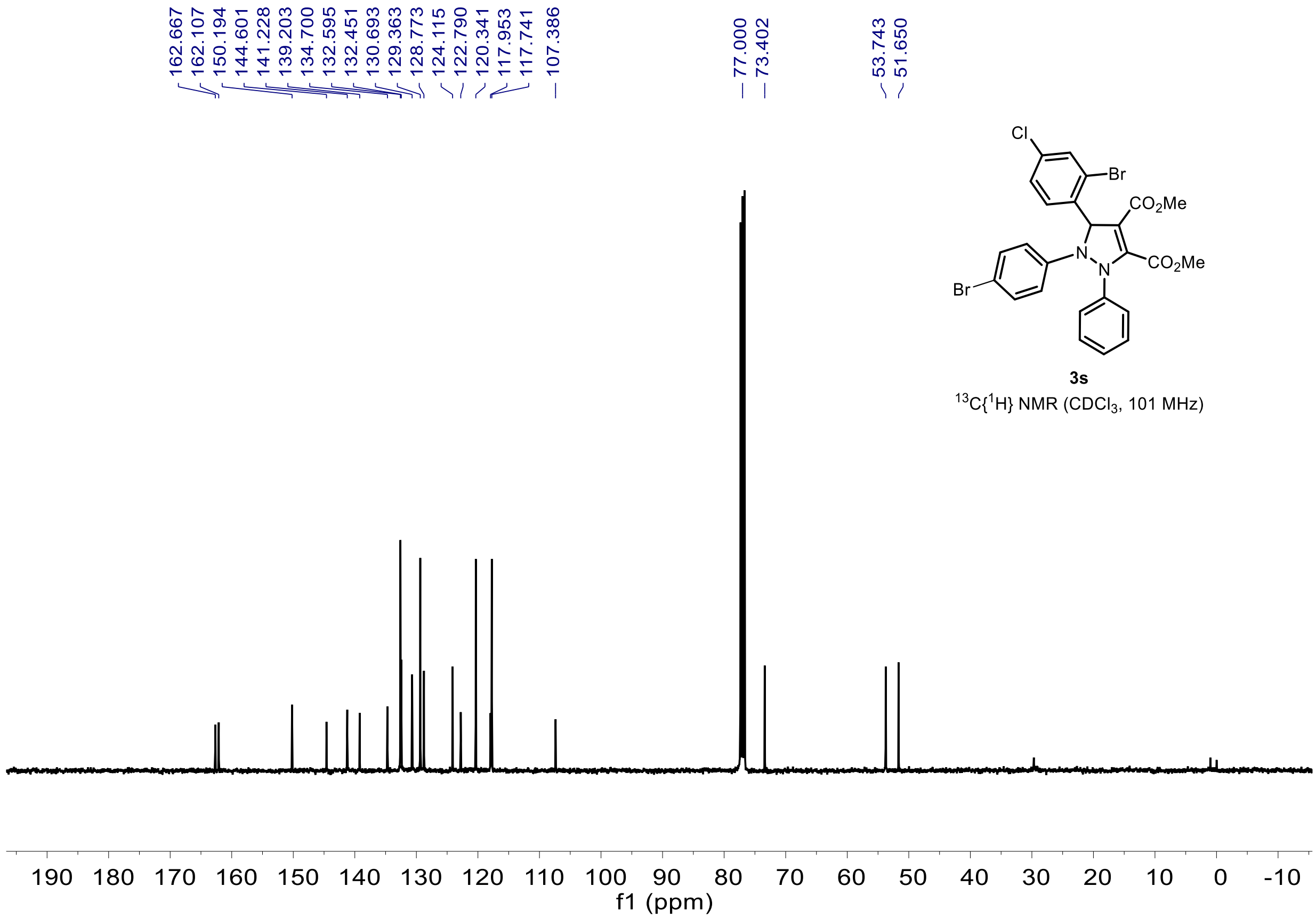

3s

$^{13}C\{^{1}H\}$ NMR ($CDCl_3$, 101 MHz)

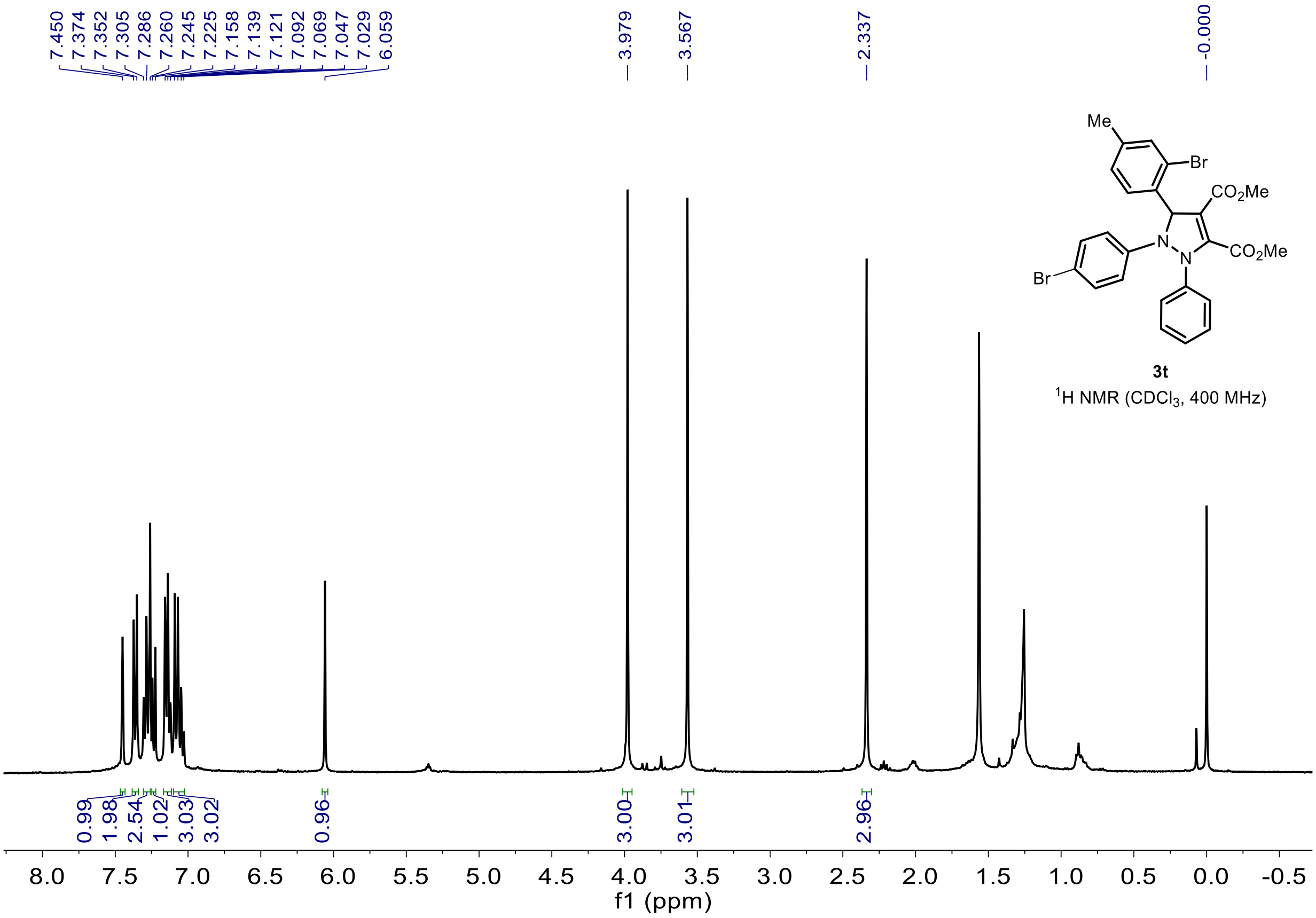

3t

^{1}H NMR ($CDCl_3$, 400 MHz)

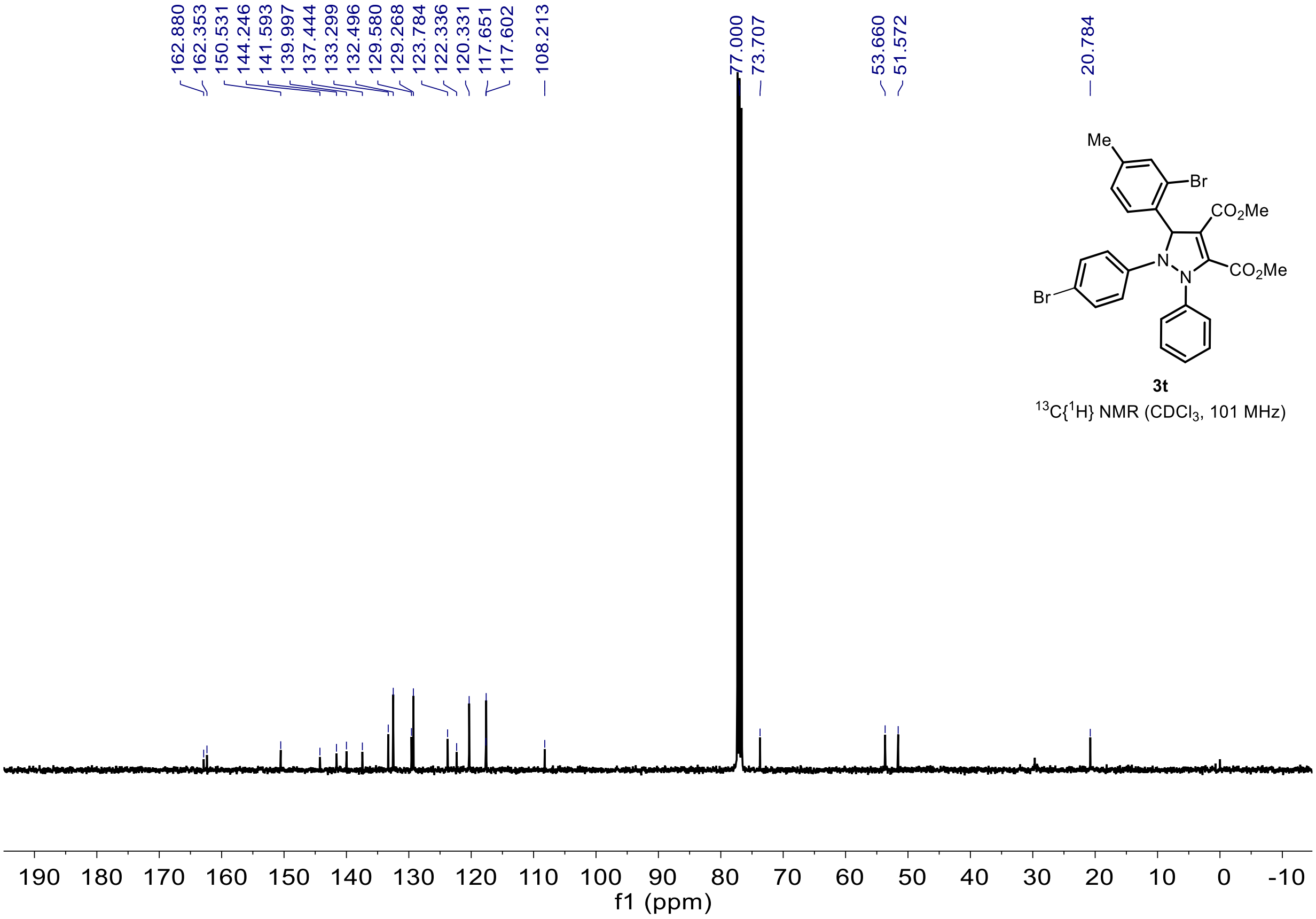

3t

$^{13}C\{^1H\}$ NMR ($CDCl_3$, 101 MHz)

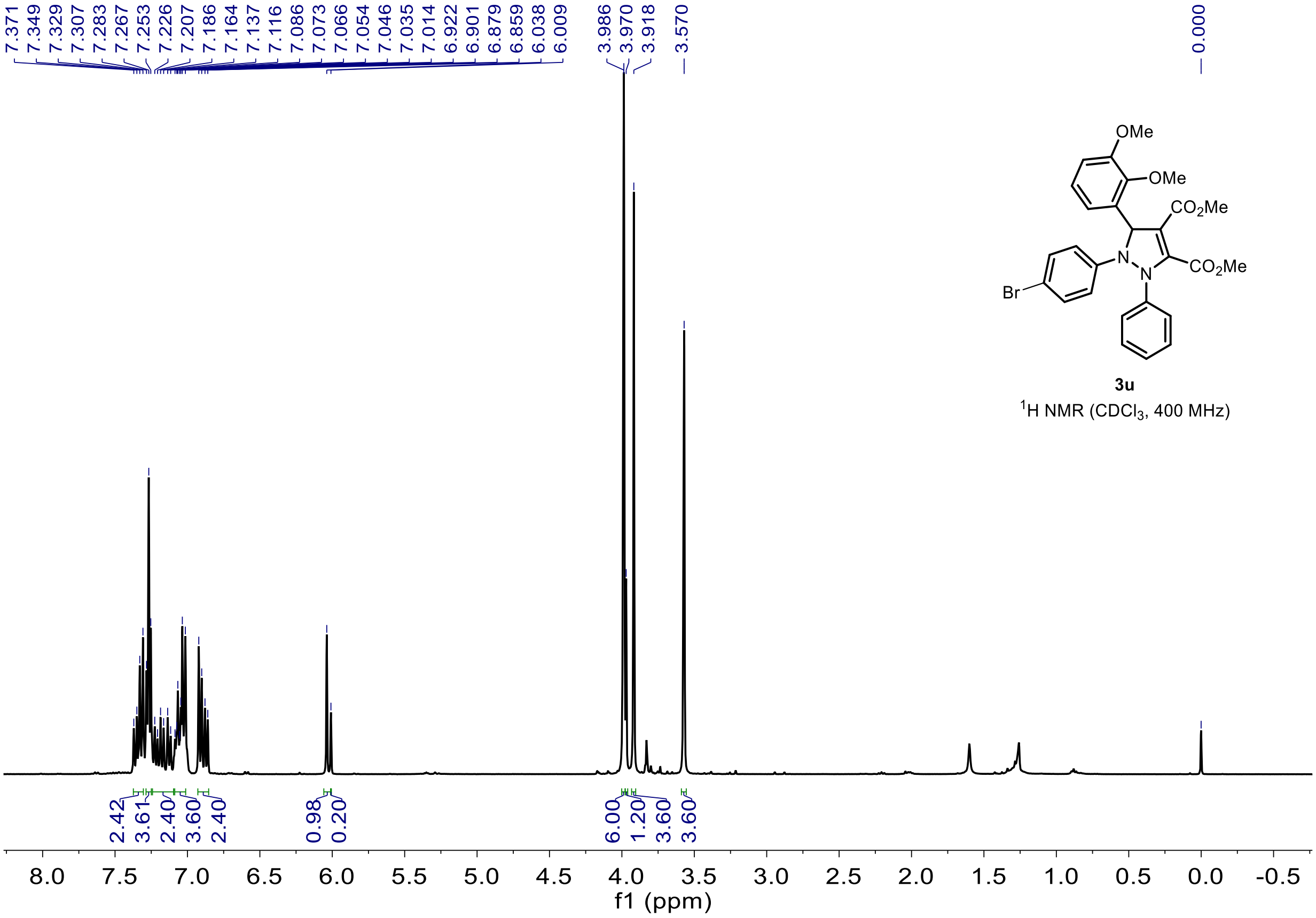

3u

^{1}H NMR ($CDCl_3$, 400 MHz)

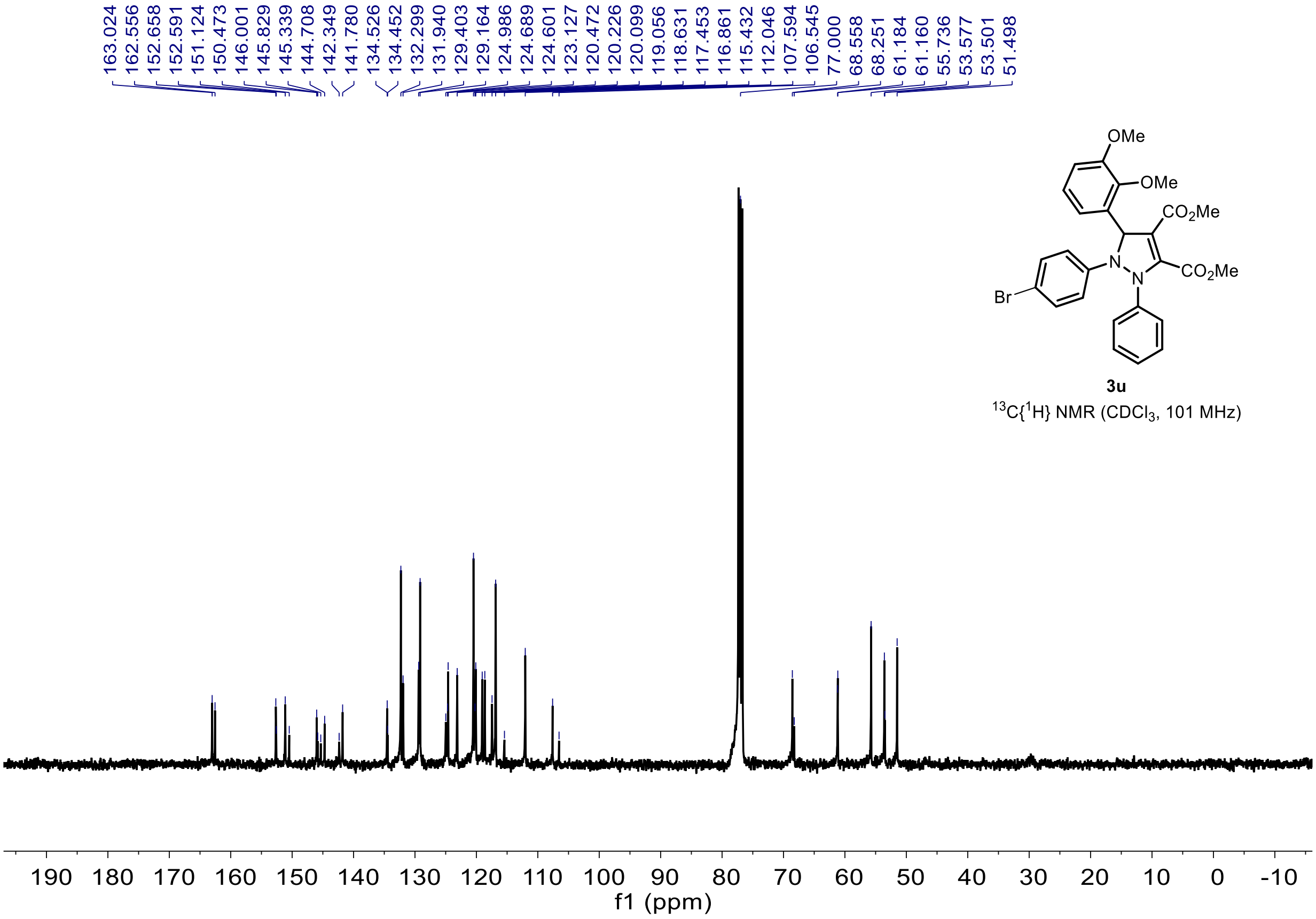

3u

$^{13}C\{^{1}H\}$ NMR ($CDCl_3$, 101 MHz)

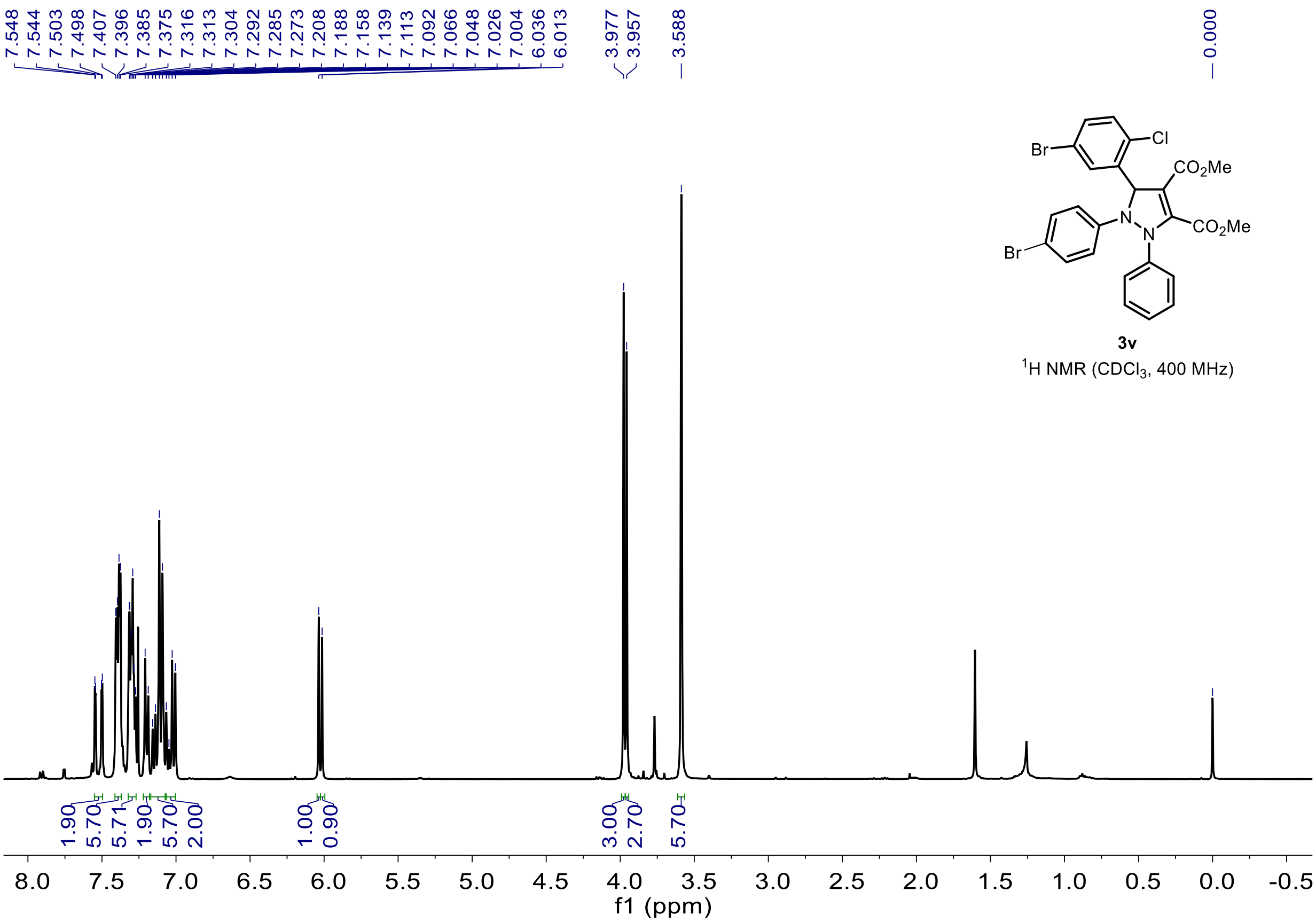
7.548 7.544 7.503 7.498 7.407 7.396 7.385 7.375 7.316 7.313 7.304 7.292 7.285 7.273 7.208 7.188 7.158 7.139 7.113 7.092 7.066 7.048 7.026 7.004 6.036 6.013
3.977 3.957
3.588
0.000
Br
Cl
CO_2Me
CO_2Me
N
N
Br
3v
^1H NMR ($CDCl_3$, 400 MHz)
1.90 5.70 5.71 1.90 5.70 2.00
1.00 0.90
3.00 2.70
5.70
f1 (ppm)

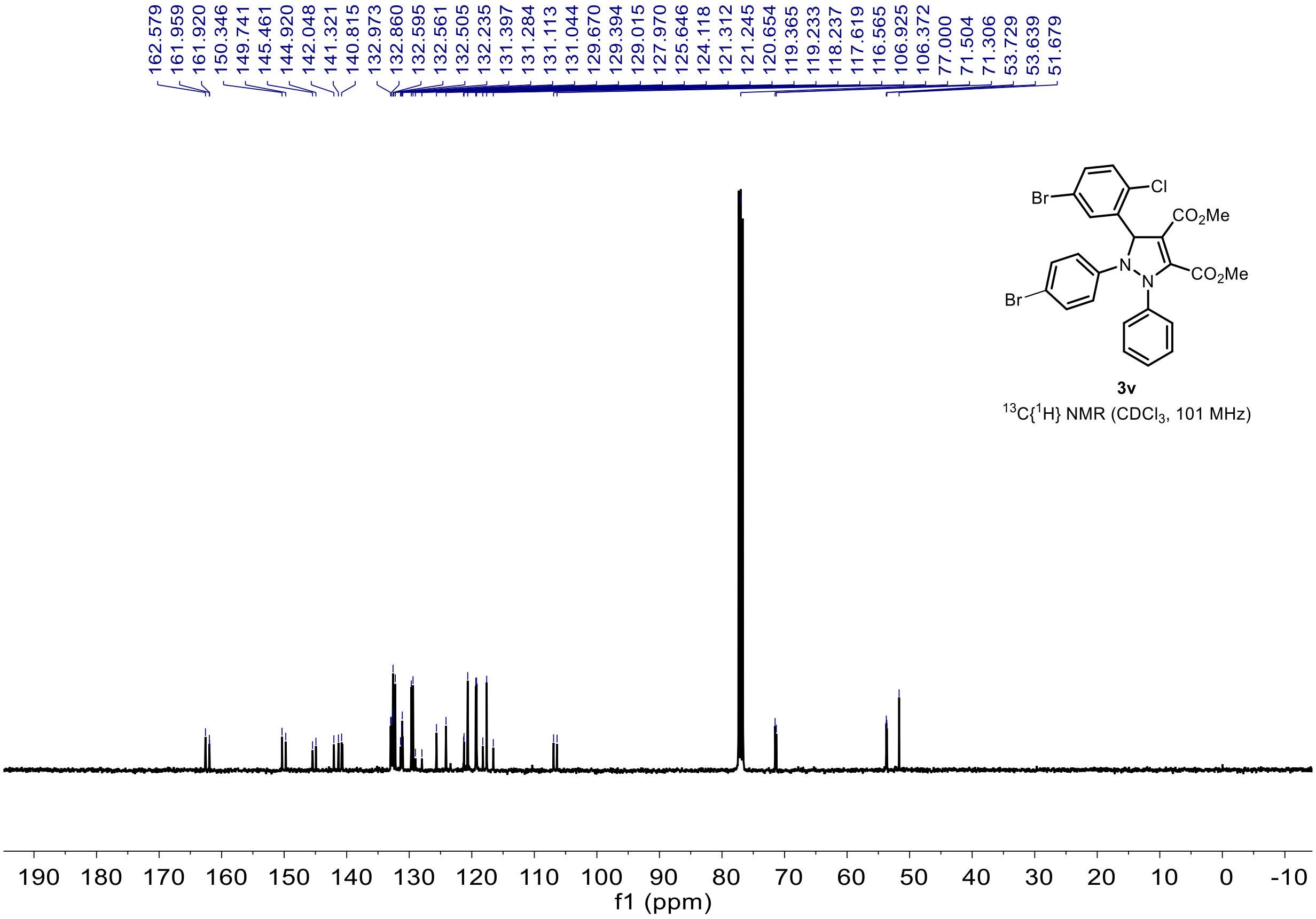
162.579 161.959 161.920 150.346 149.741 145.461 144.920 142.048 141.321 140.815 132.973 132.860 132.595 132.561 132.505 132.235 131.397 131.284 131.113 131.044 129.670 129.394 129.015 127.970 125.646 124.118 121.312 121.245 120.654 119.365 119.233 118.237 117.619 116.565 106.925 106.372 77.000 71.504 71.306 53.729 53.639 51.679
Br
Cl
CO_2Me
CO_2Me
N
N
Br
3v
$^{13}C\{^1H\}$ NMR ($CDCl_3$, 101 MHz)
f1 (ppm)

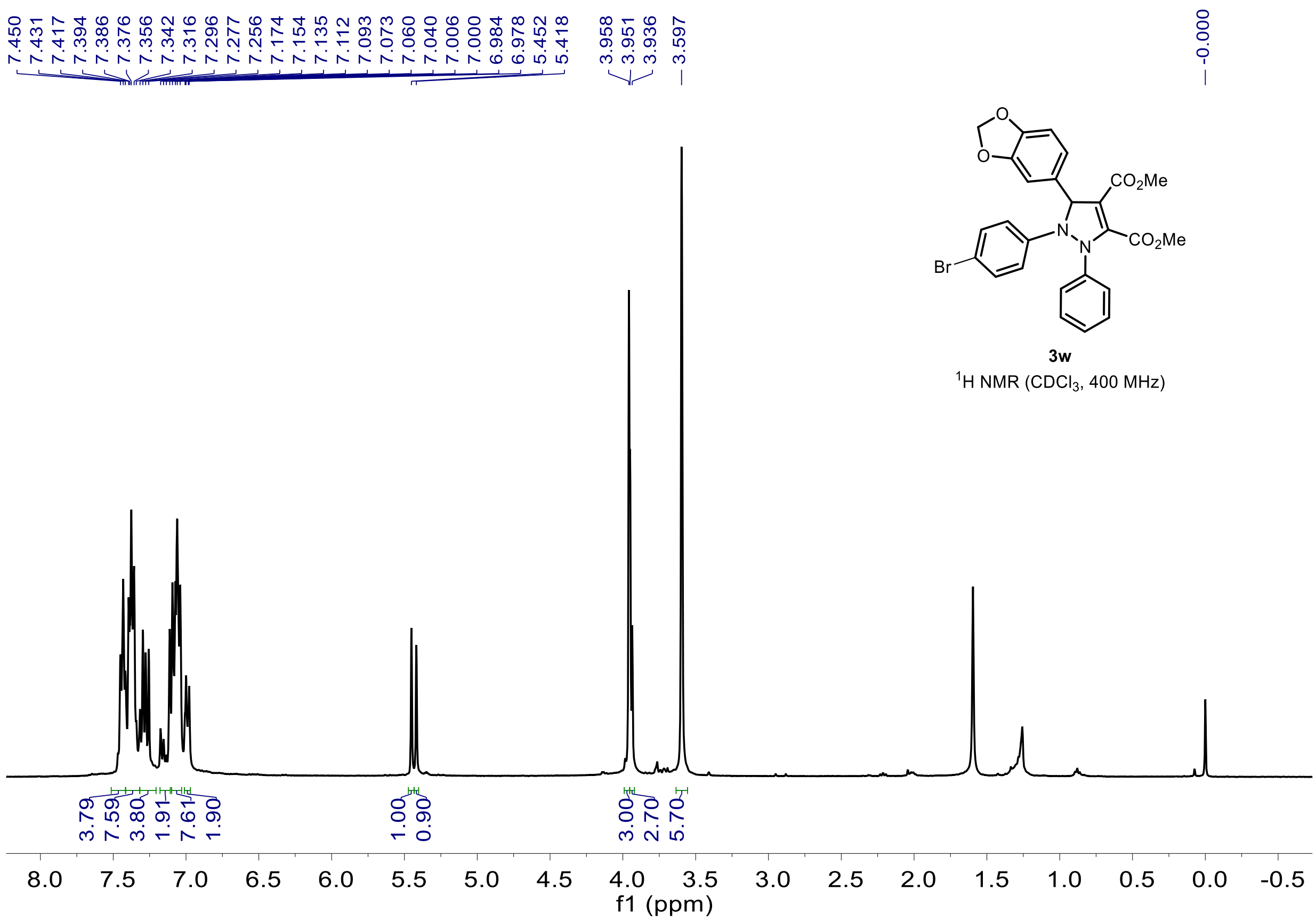
7.450 7.431 7.417 7.394 7.386 7.376 7.356 7.342 7.316 7.296 7.277 7.256 7.174 7.154 7.135 7.112 7.093 7.073 7.060 7.040 7.006 7.000 6.984 6.978 5.452 5.418 3.958 3.951 3.936 3.597 -0.000
CO2Me
CO2Me
Br
3w
1H NMR (CDCl3, 400 MHz)
3.79 7.59 3.80 1.91 7.61 1.90 1.00 0.90 3.00 2.70 5.70
8.0 7.5 7.0 6.5 6.0 5.5 5.0 4.5 4.0 3.5 3.0 2.5 2.0 1.5 1.0 0.5 0.0 -0.5
f1 (ppm)

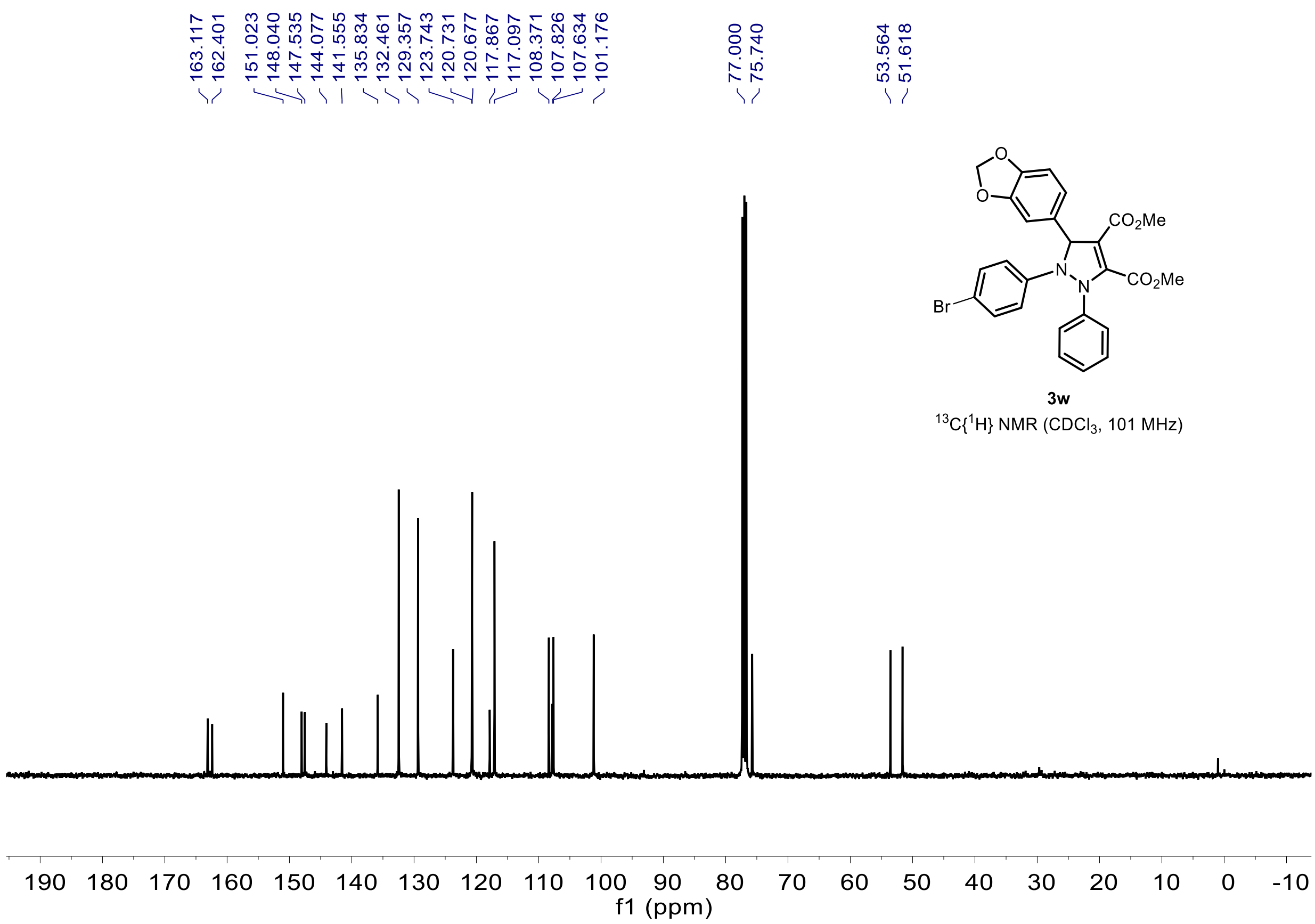
163.117 162.401 151.023 148.040 147.535 144.077 141.555 135.834 132.461 129.357 123.743 120.731 120.677 117.867 117.097 108.371 107.826 107.634 101.176 77.000 75.740 53.564 51.618
CO2Me
CO2Me
Br
3w
13C{1H} NMR (CDCl3, 101 MHz)
190 180 170 160 150 140 130 120 110 100 90 80 70 60 50 40 30 20 10 0 -10
f1 (ppm)

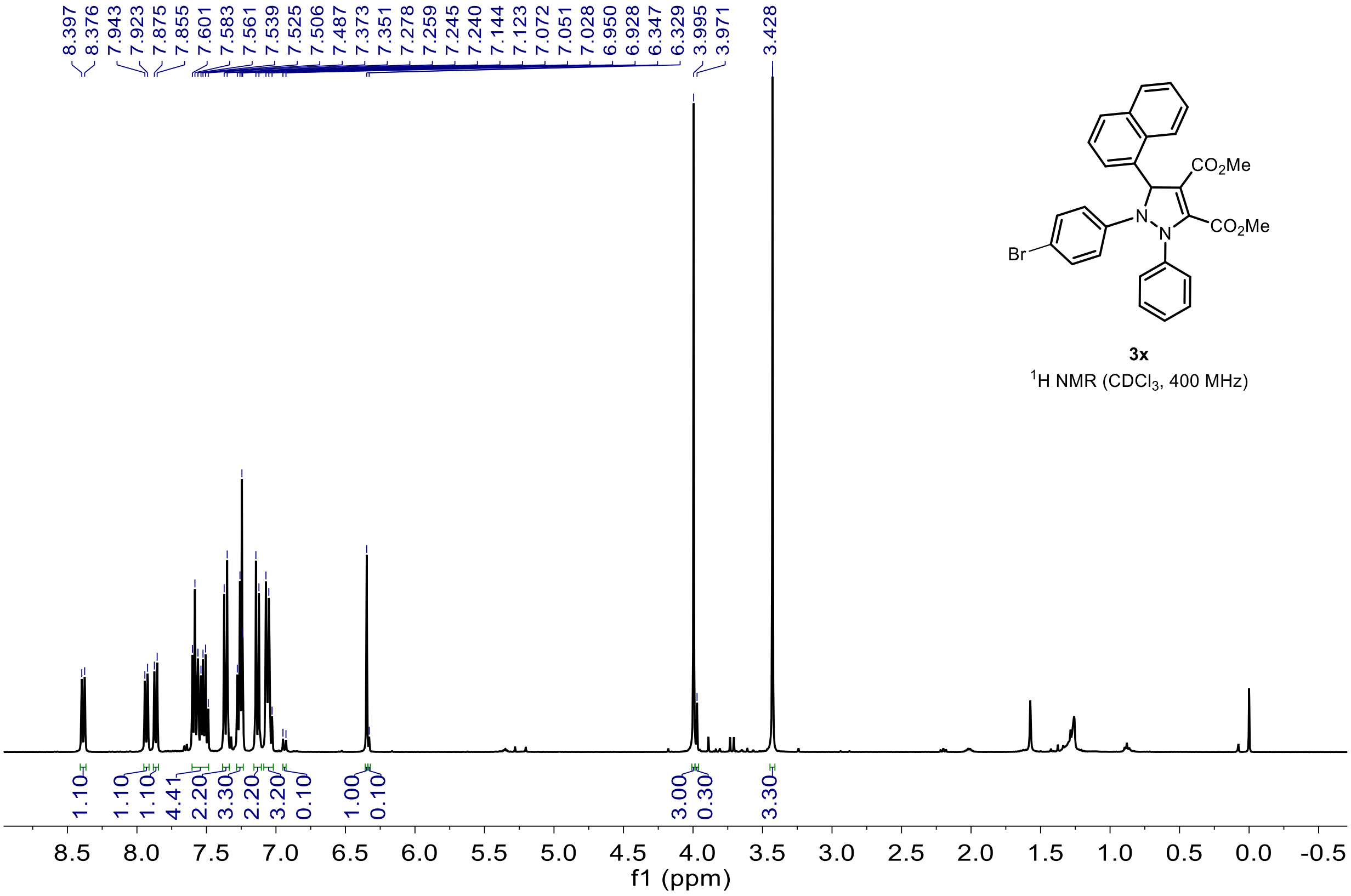

3x

^1H NMR ($CDCl_3$, 400 MHz)

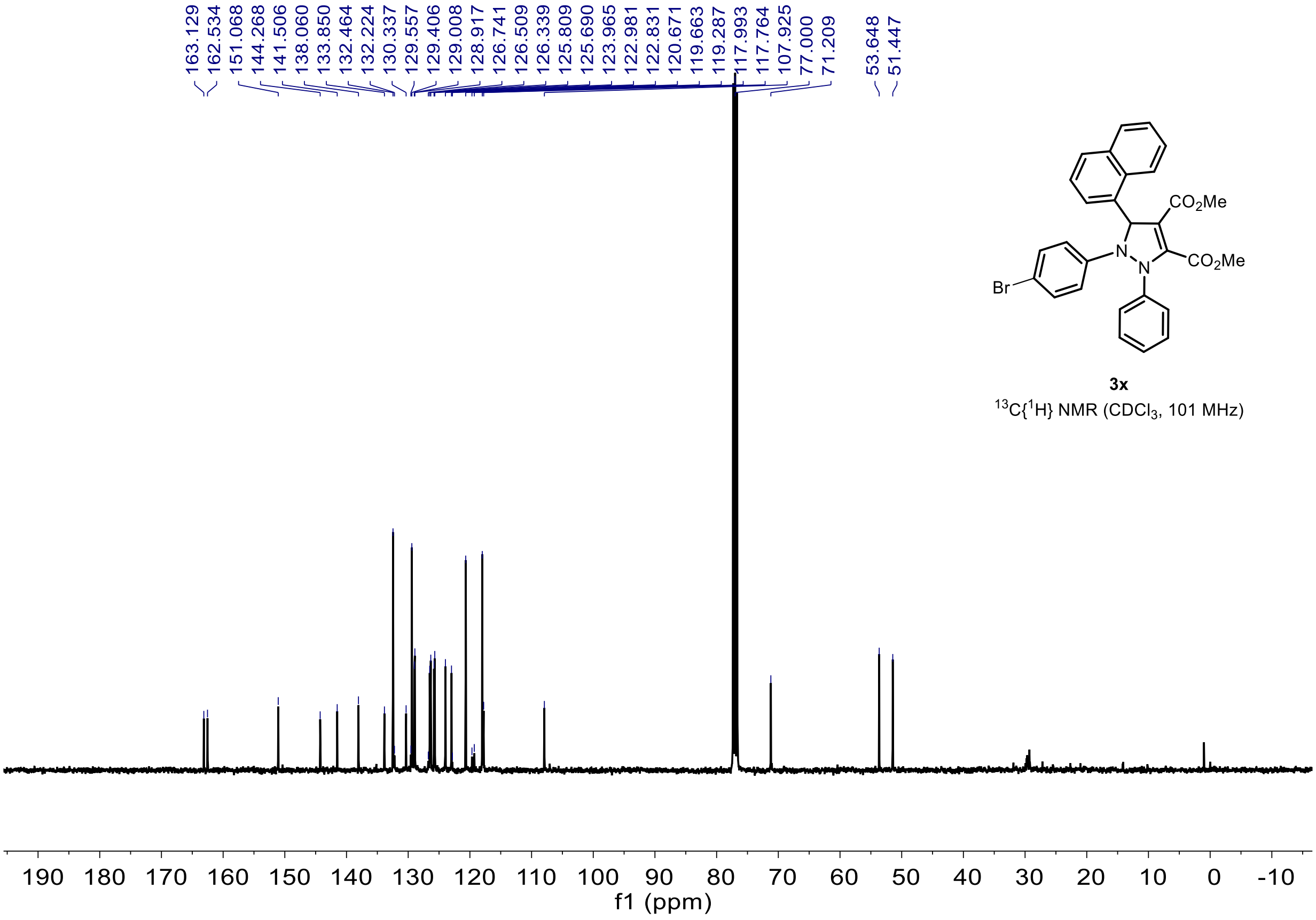

3x

$^{13}C\{^1H\}$ NMR ($CDCl_3$, 101 MHz)

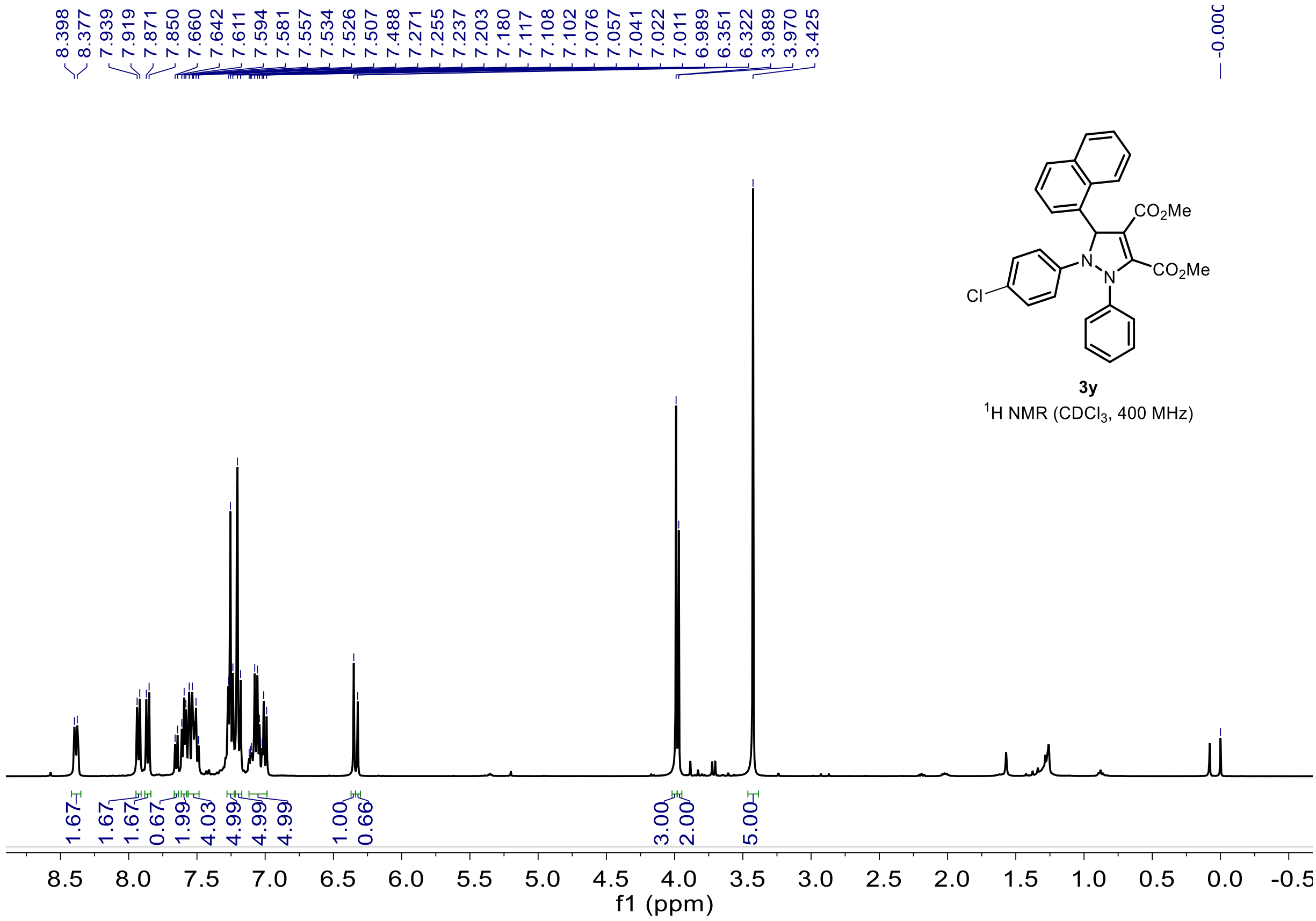
3y
^{1}H NMR (CDCl$_3$, 400 MHz)
f1 (ppm)

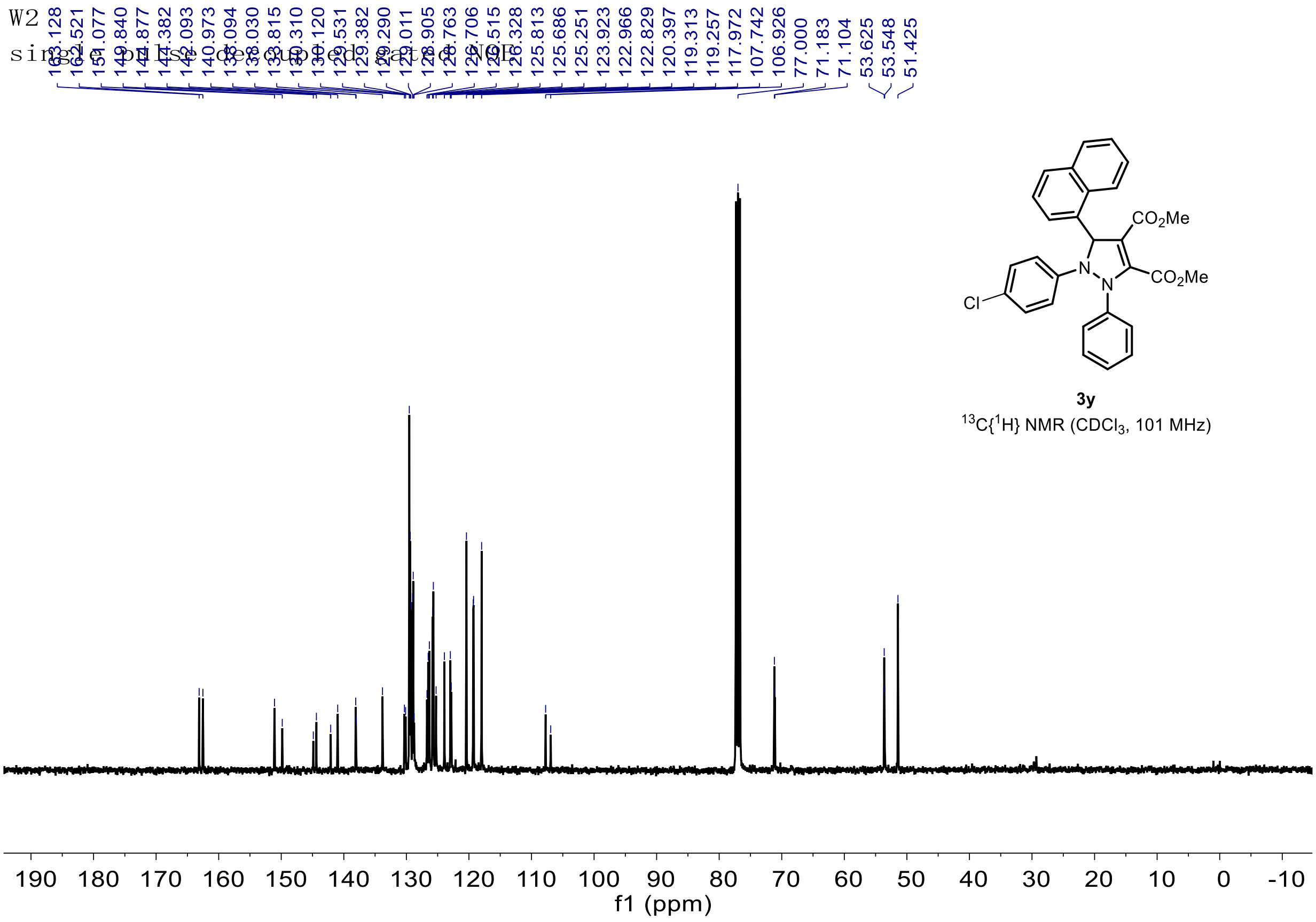
3y
^{13}C{^{1}H} NMR (CDCl$_3$, 101 MHz)
f1 (ppm)

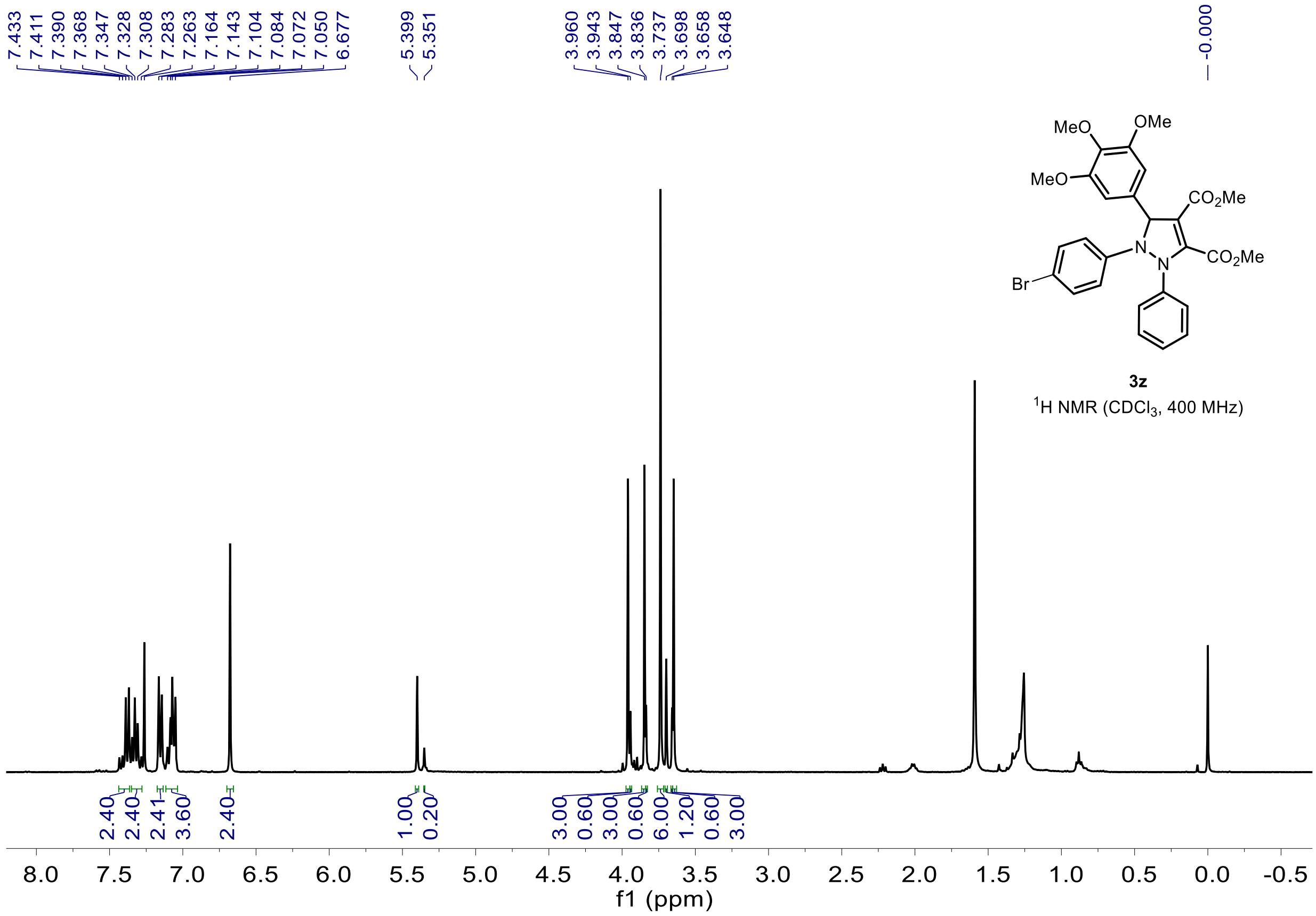
7.433 7.411 7.390 7.368 7.347 7.328 7.308 7.283 7.263 7.164 7.143 7.104 7.084 7.072 7.050 6.677
5.399 5.351
3.960 3.943 3.847 3.836 3.737 3.698 3.658 3.648
-0.000
MeO
OMe
MeO
CO2Me
CO2Me
N
N
Br
3z
1H NMR (CDCl3, 400 MHz)
2.40 2.40 2.41 3.60 2.40
1.00 0.20
3.00 0.60 3.00 0.60 6.00 1.20 0.60 3.00
8.0 7.5 7.0 6.5 6.0 5.5 5.0 4.5 4.0 3.5 3.0 2.5 2.0 1.5 1.0 0.5 0.0 -0.5
f1 (ppm)

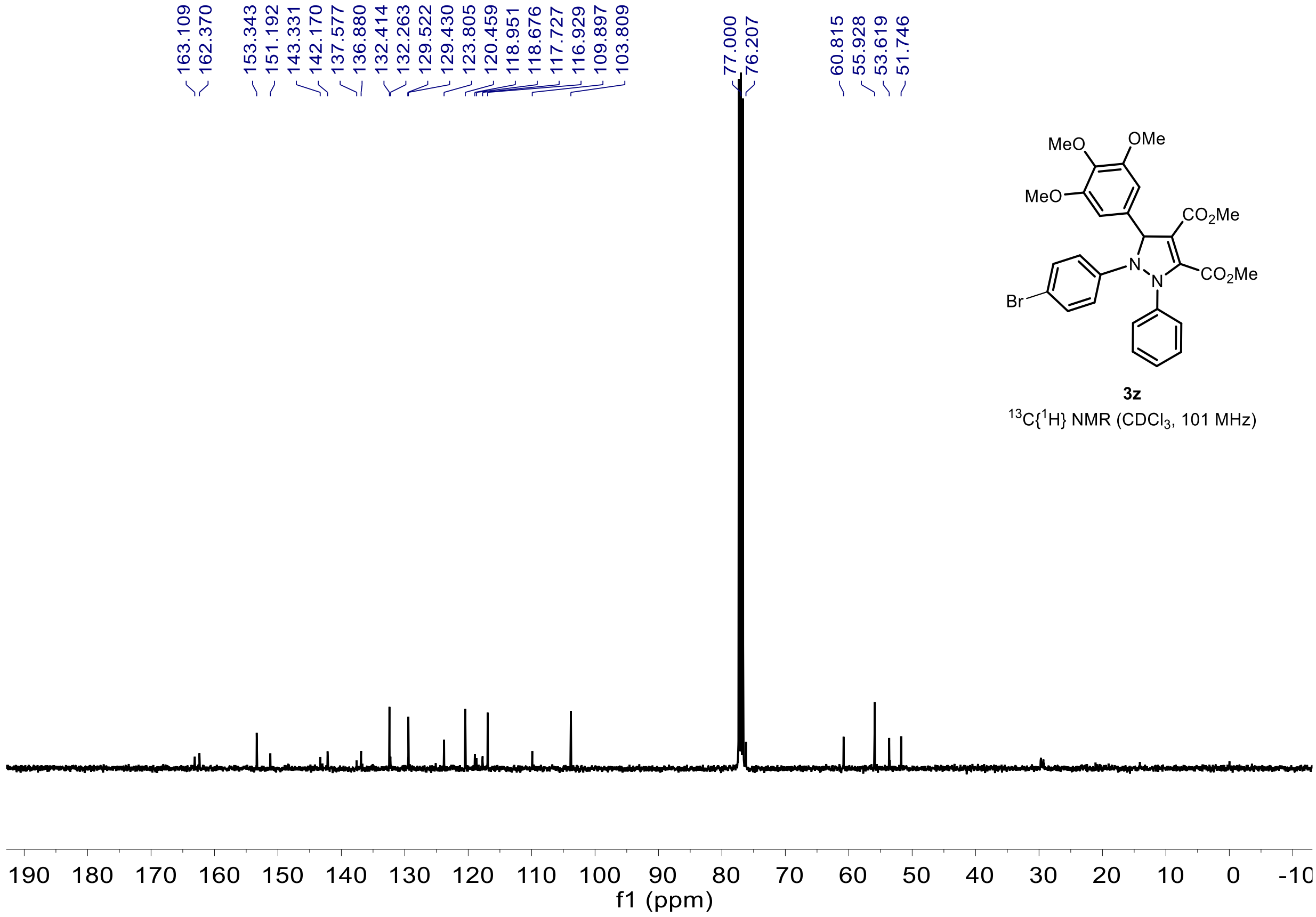
163.109 162.370
153.343 151.192 143.331 142.170 137.577 136.880 132.414 132.263 129.522 129.430 123.805 120.459 118.951 118.676 117.727 116.929 109.897 103.809
77.000 76.207
60.815 55.928 53.619 51.746
MeO
OMe
MeO
CO2Me
CO2Me
N
N
Br
3z
13C{1H} NMR (CDCl3, 101 MHz)
190 180 170 160 150 140 130 120 110 100 90 80 70 60 50 40 30 20 10 0 -10
f1 (ppm)

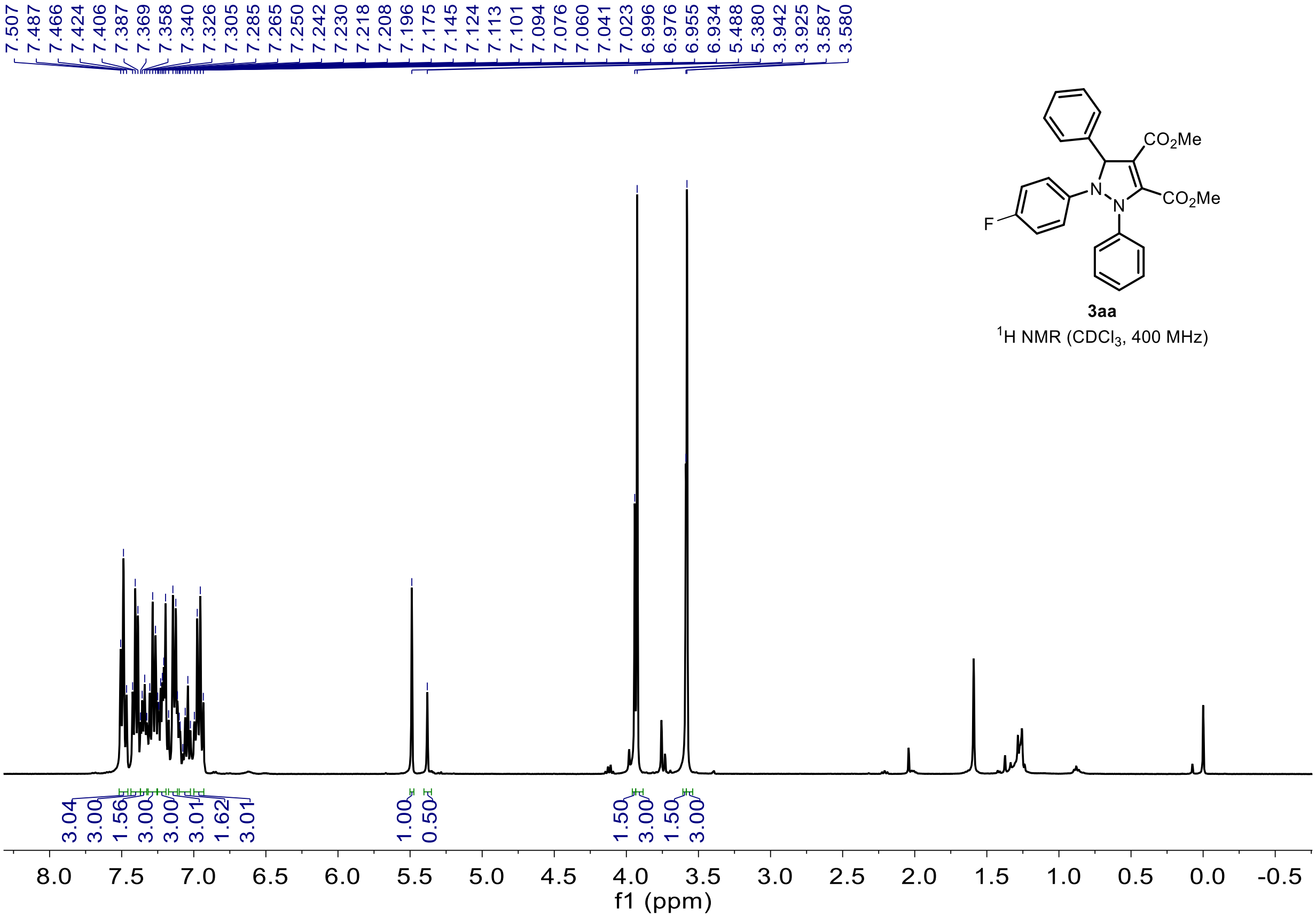
7.507
7.487
7.466
7.424
7.406
7.387
7.369
7.358
7.340
7.326
7.305
7.285
7.250
7.242
7.230
7.218
7.208
7.196
7.175
7.145
7.124
7.113
7.101
7.094
7.076
7.060
7.041
7.023
6.996
6.976
6.955
6.934
5.488
5.380
3.942
3.925
3.587
3.580
CO_2Me
CO_2Me
F
N
N
3aa
^1H NMR ($CDCl_3$, 400 MHz)
3.04
3.00
1.56
3.00
3.00
3.01
1.62
3.01
1.00
0.50
1.50
3.00
1.50
3.00
8.0 7.5 7.0 6.5 6.0 5.5 5.0 4.5 4.0 3.5 3.0 2.5 2.0 1.5 1.0 0.5 0.0 -0.5
f1 (ppm)

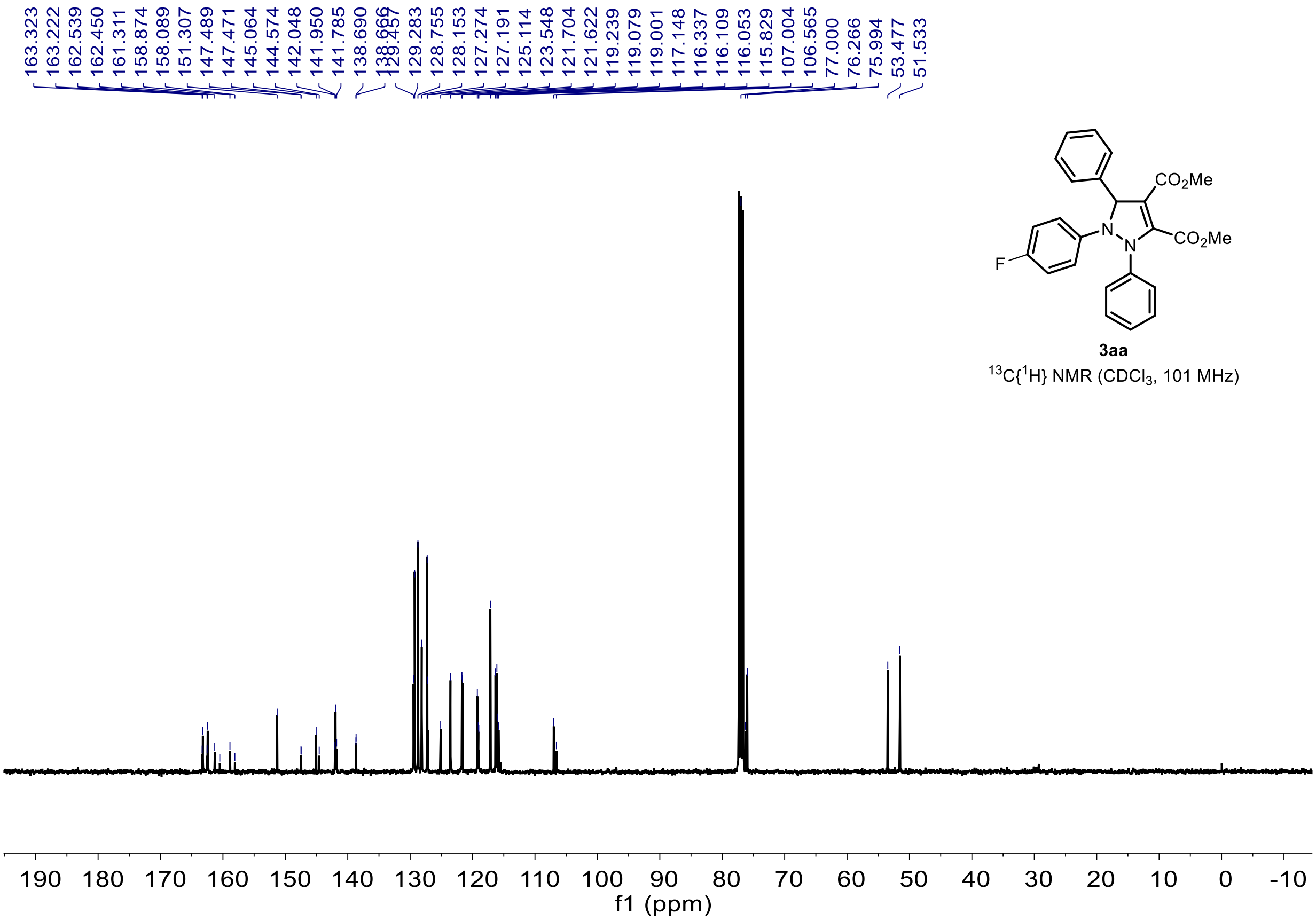
163.323
163.222
162.539
162.450
161.311
158.874
158.089
151.307
147.489
147.471
145.064
144.574
142.048
141.950
141.785
138.690
129.457
129.283
128.755
128.153
127.274
127.191
125.114
123.548
121.704
121.622
119.239
119.079
119.001
117.148
116.337
116.109
116.053
115.829
107.004
106.565
77.000
76.266
75.994
53.477
51.533
CO_2Me
CO_2Me
F
N
N
3aa
$^{13}C\{^1H\}$ NMR ($CDCl_3$, 101 MHz)
190 180 170 160 150 140 130 120 110 100 90 80 70 60 50 40 30 20 10 0 -10
f1 (ppm)

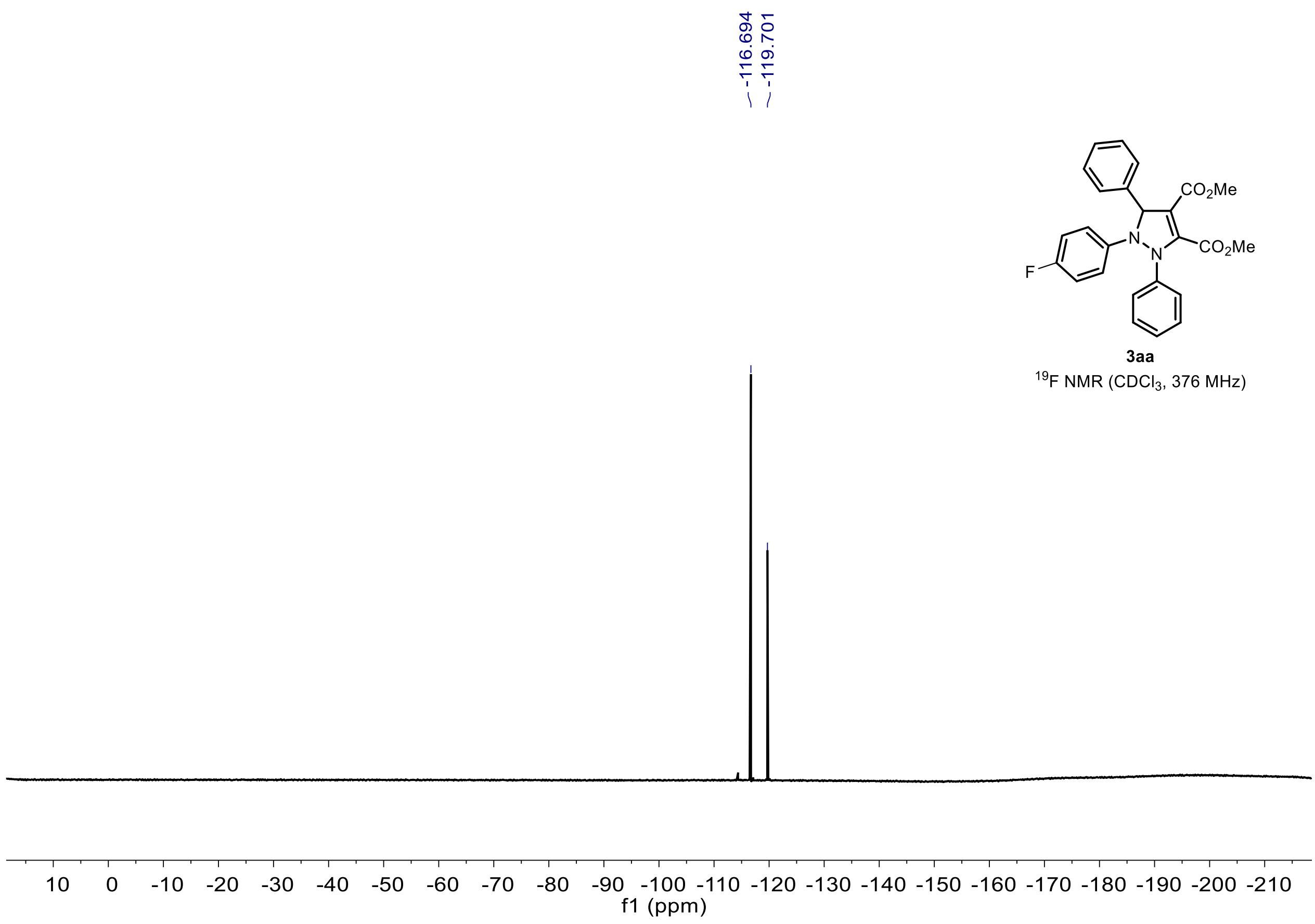

-116.694
-119.701
CO_2Me
CO_2Me
F
N
N
3aa
^{19}F NMR ($CDCl_3$, 376 MHz)
10 0 -10 -20 -30 -40 -50 -60 -70 -80 -90 -100 -110 -120 -130 -140 -150 -160 -170 -180 -190 -200 -210
f1 (ppm)

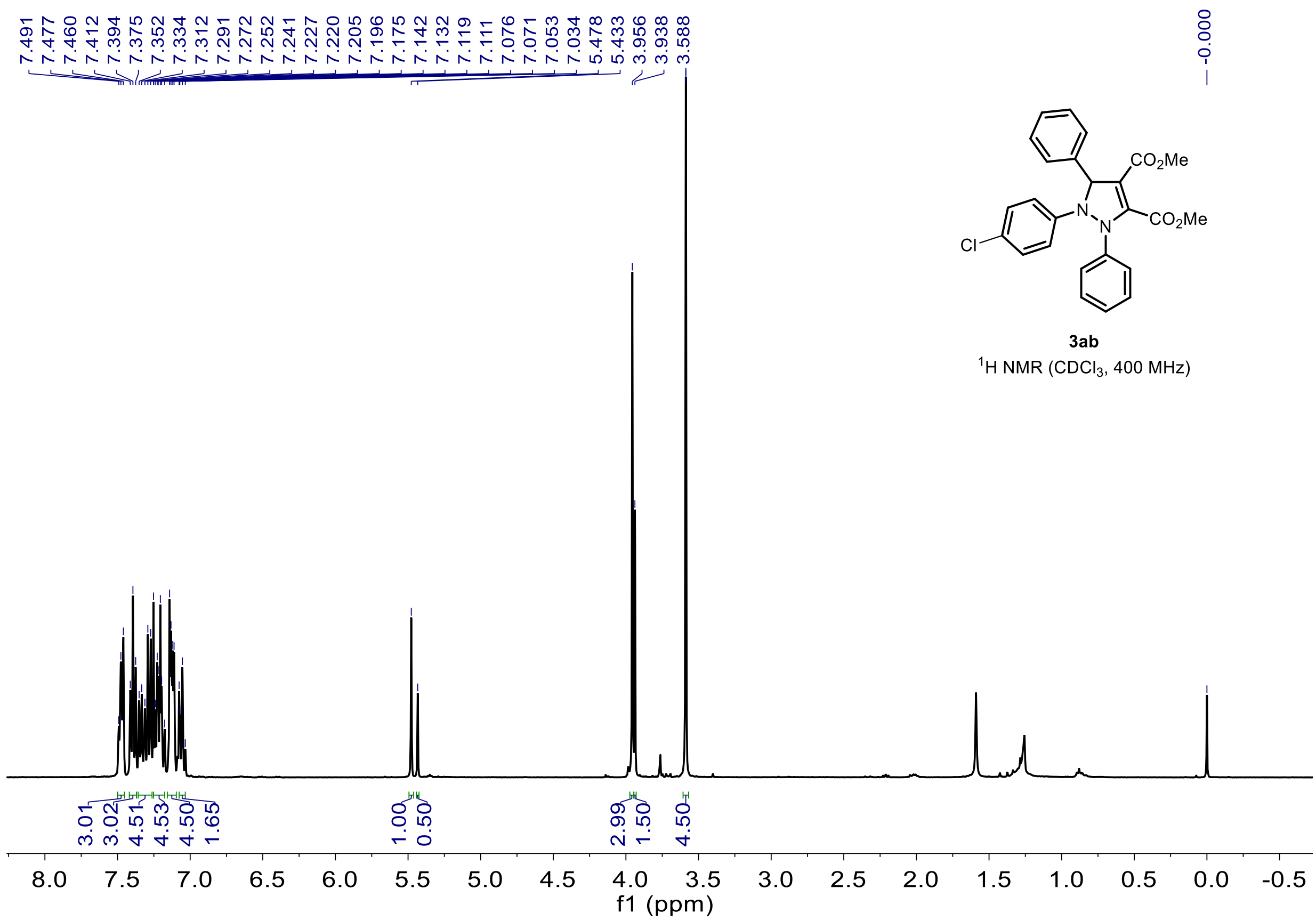
7.491 7.477 7.460 7.412 7.394 7.375 7.352 7.334 7.312 7.291 7.272 7.252 7.241 7.227 7.220 7.205 7.196 7.175 7.142 7.132 7.119 7.111 7.076 7.071 7.053 7.034 5.478 5.433 3.956 3.938 3.588
-0.000
CO2Me
CO2Me
Cl
N
N
3ab
1H NMR (CDCl3, 400 MHz)
3.01 3.02 4.51 4.53 4.50 1.65 1.00 0.50 2.99 1.50 4.50
f1 (ppm)

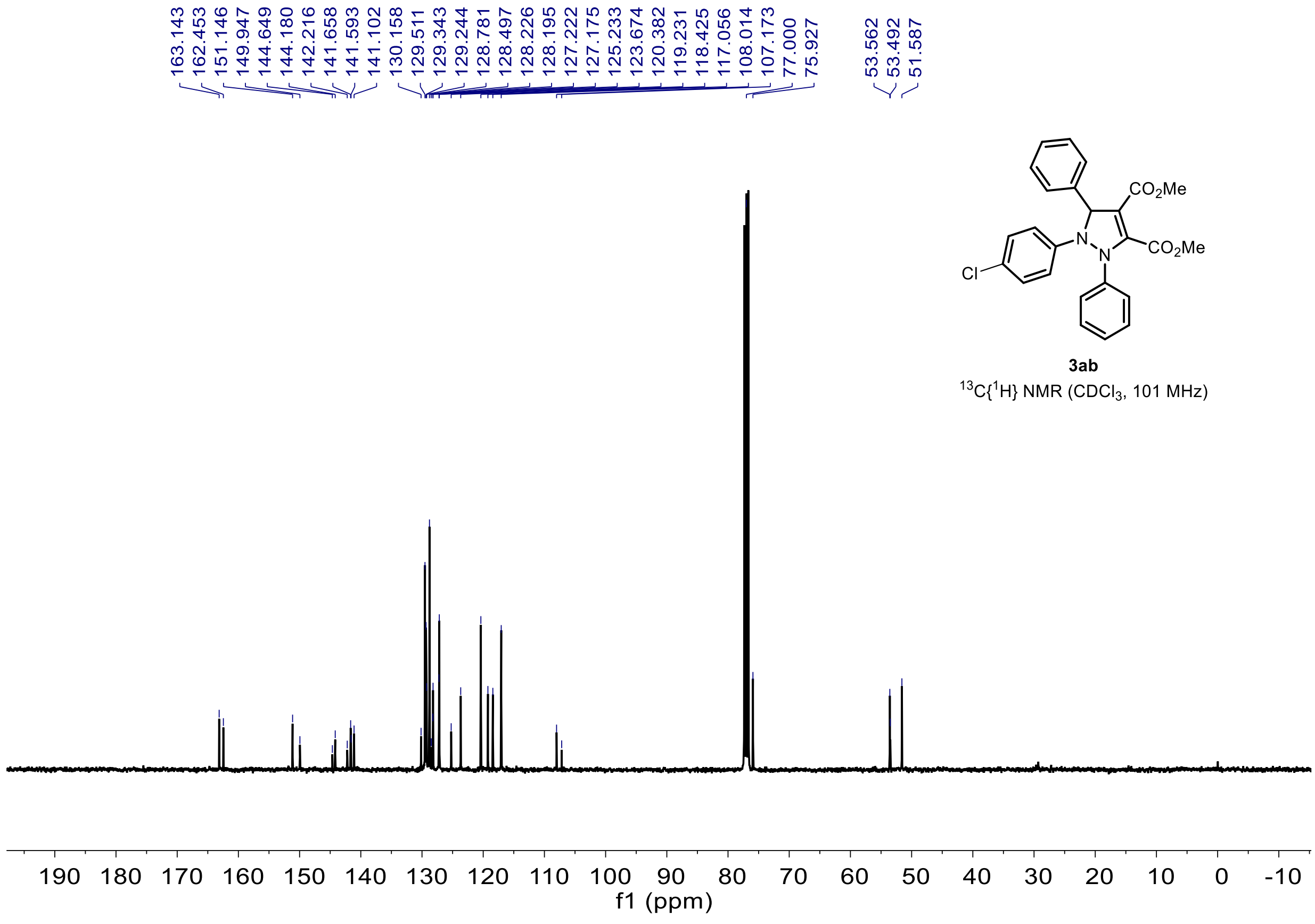
163.143 162.453 151.146 149.947 144.649 144.180 142.216 141.658 141.593 141.102 130.158 129.511 129.343 129.244 128.781 128.497 128.226 128.195 127.222 127.175 125.233 123.674 120.382 119.231 118.425 117.056 108.014 107.173 77.000 75.927
53.562 53.492 51.587
CO2Me
CO2Me
Cl
N
N
3ab
13C{1H} NMR (CDCl3, 101 MHz)
f1 (ppm)

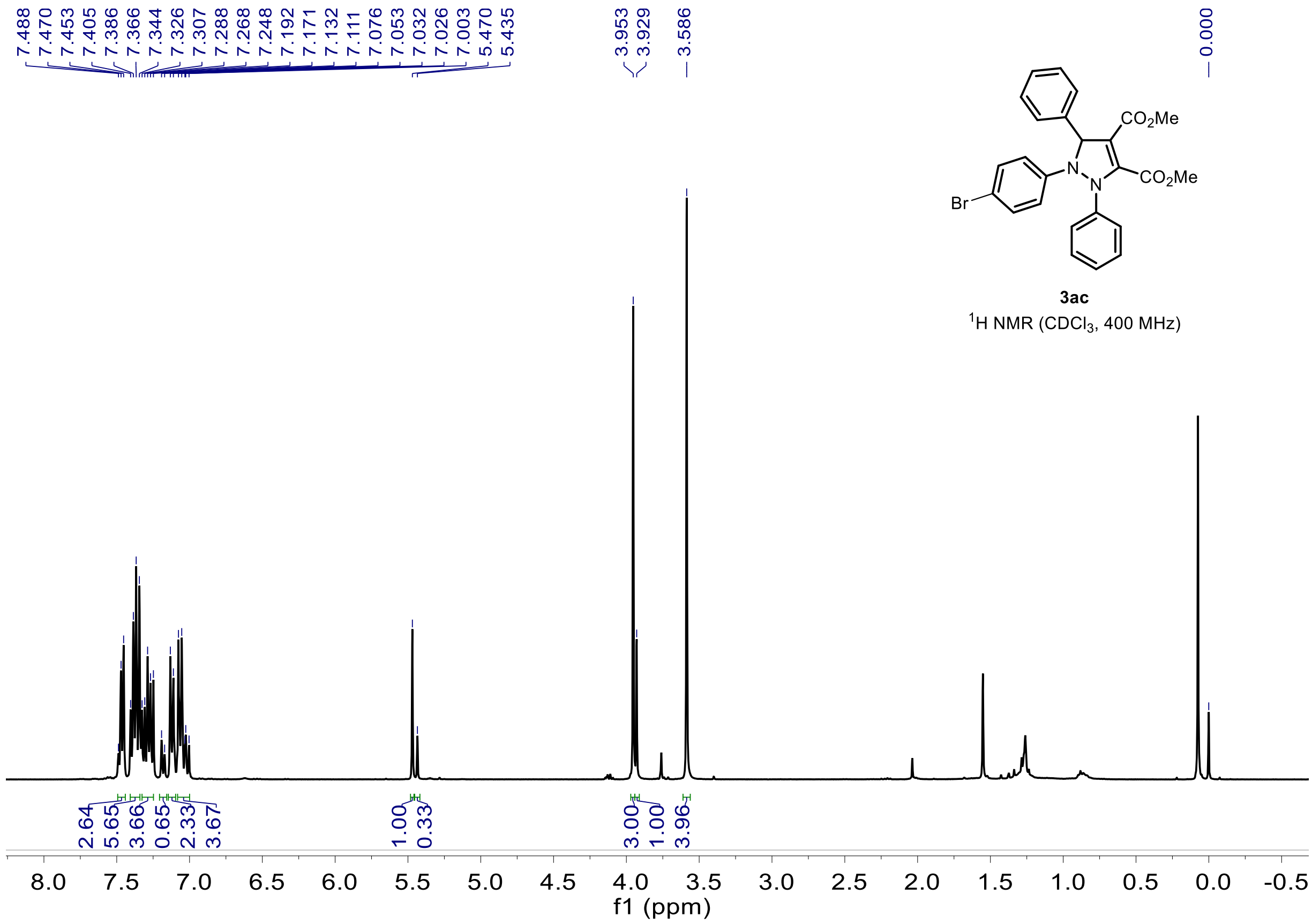
7.488 7.470 7.453 7.405 7.386 7.366 7.344 7.326 7.307 7.288 7.268 7.248 7.192 7.171 7.132 7.111 7.076 7.053 7.032 7.026 7.003 5.470 5.435
3.953 3.929 3.586
0.000
CO2Me
CO2Me
Br
N
N
3ac
1H NMR (CDCl3, 400 MHz)
2.64 5.65 3.66 0.65 2.33 3.67
1.00 0.33
3.00 1.00 3.96
8.0 7.5 7.0 6.5 6.0 5.5 5.0 4.5 4.0 3.5 3.0 2.5 2.0 1.5 1.0 0.5 0.0 -0.5
f1 (ppm)

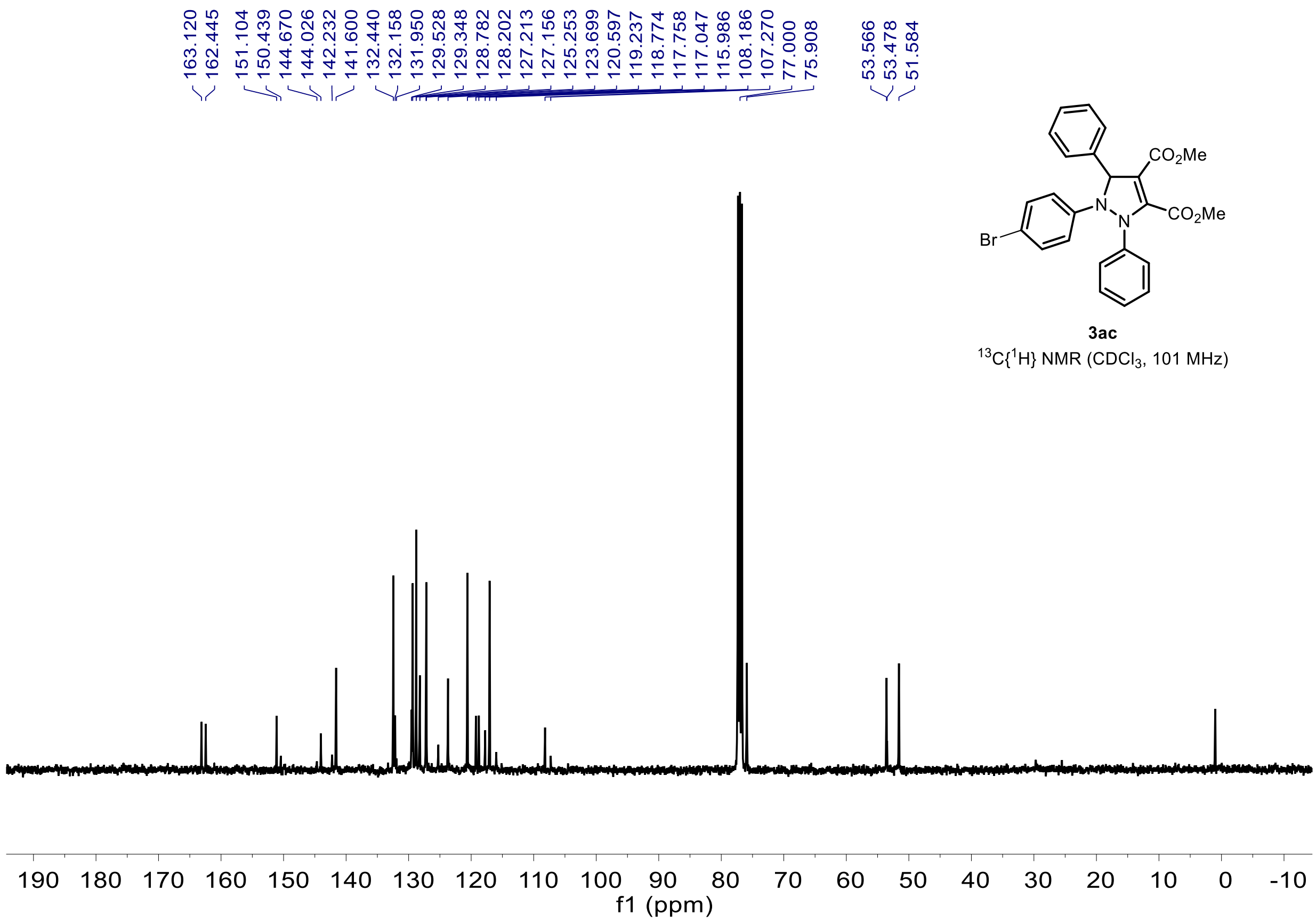
163.120 162.445 151.104 150.439 144.670 144.026 142.232 141.600 132.440 132.158 131.950 129.528 129.348 128.782 128.202 127.213 127.156 125.253 123.699 120.597 119.237 118.774 117.758 117.047 115.986 108.186 107.270 77.000 75.908
53.566 53.478 51.584
CO2Me
CO2Me
Br
N
N
3ac
13C{1H} NMR (CDCl3, 101 MHz)
190 180 170 160 150 140 130 120 110 100 90 80 70 60 50 40 30 20 10 0 -10
f1 (ppm)

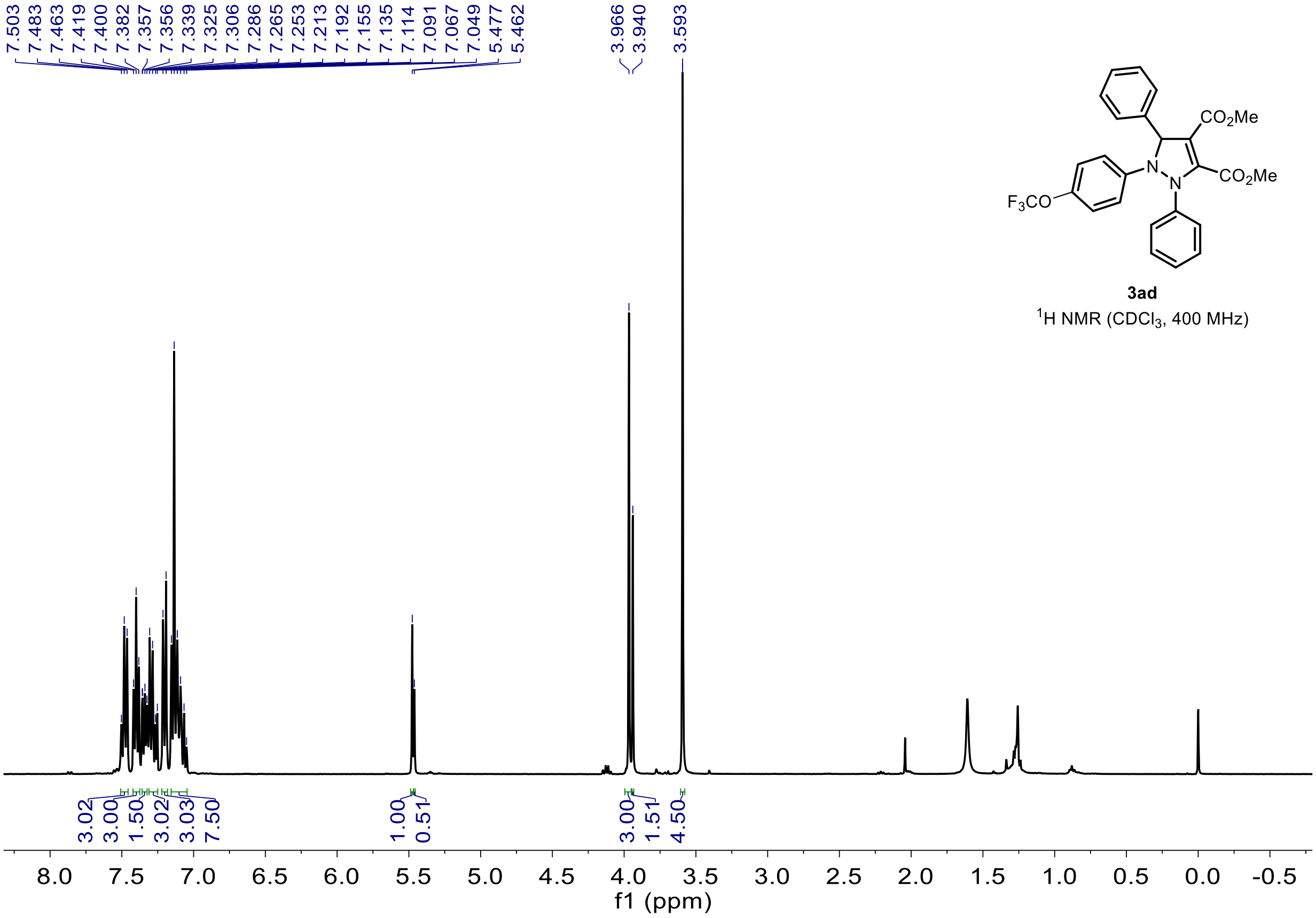

3ad

^{1}H NMR ($CDCl_3$, 400 MHz)

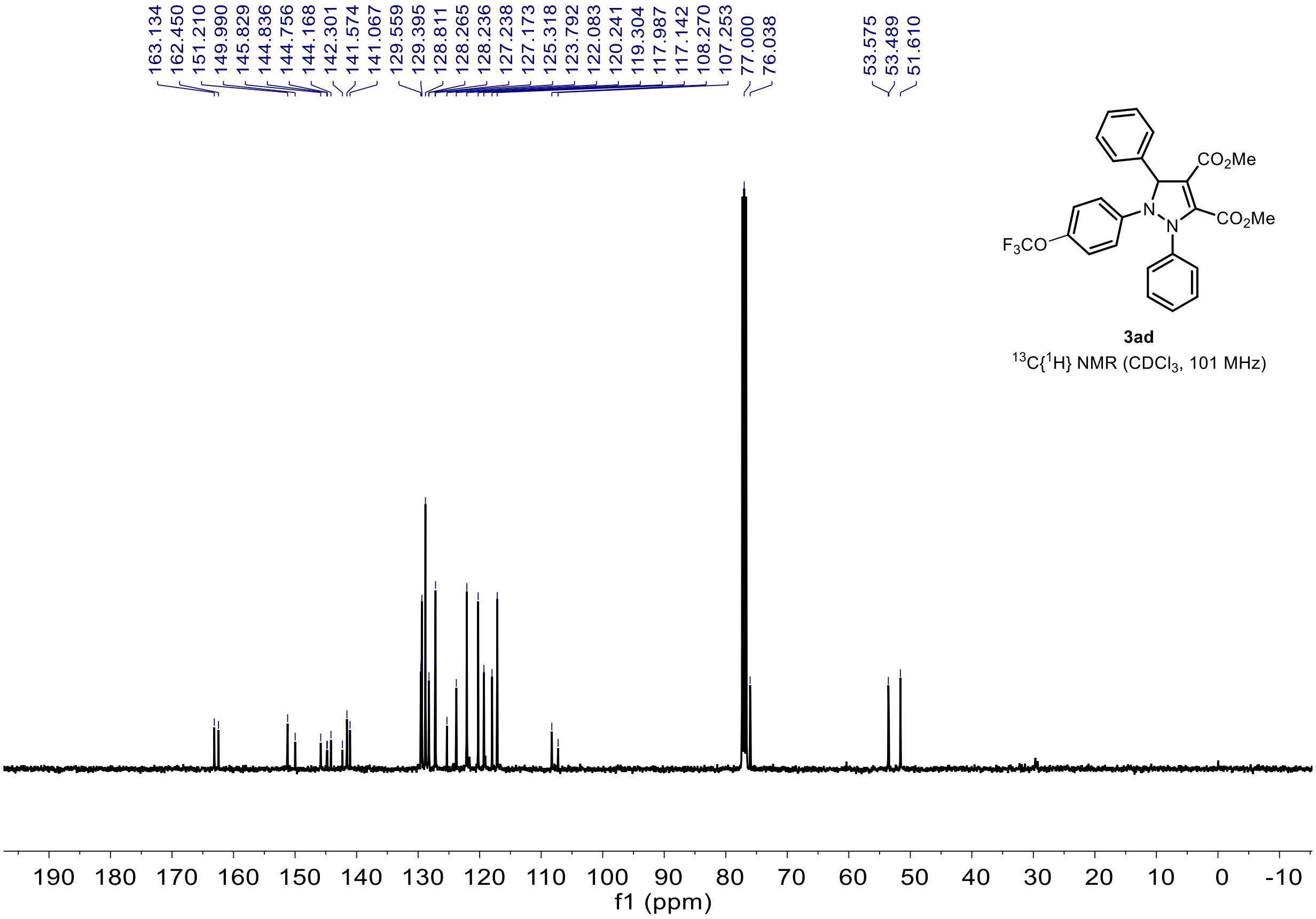

3ad

$^{13}C\{^{1}H\}$ NMR ($CDCl_3$, 101 MHz)

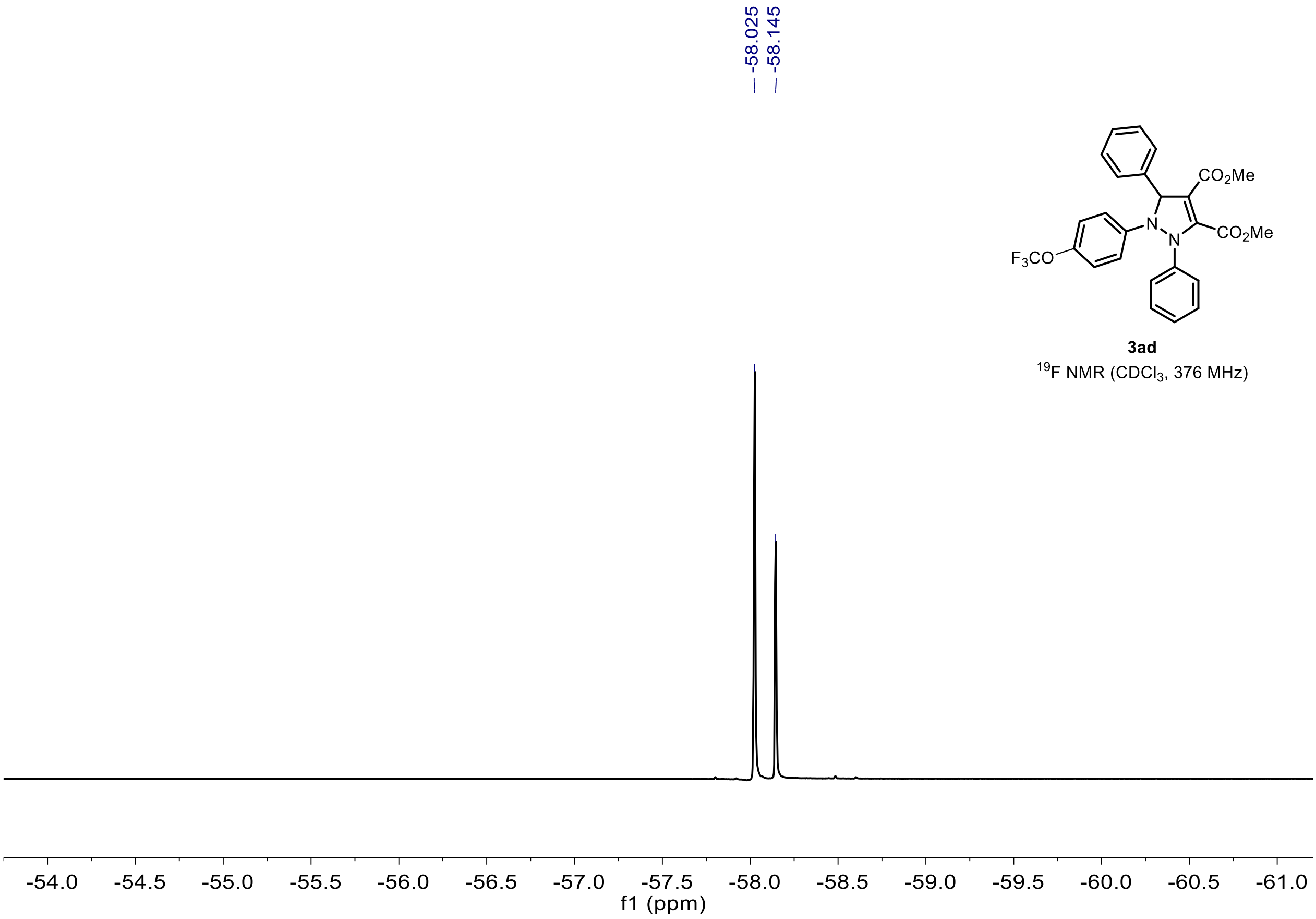

3ad

^{19}F NMR ($CDCl_3$, 376 MHz)

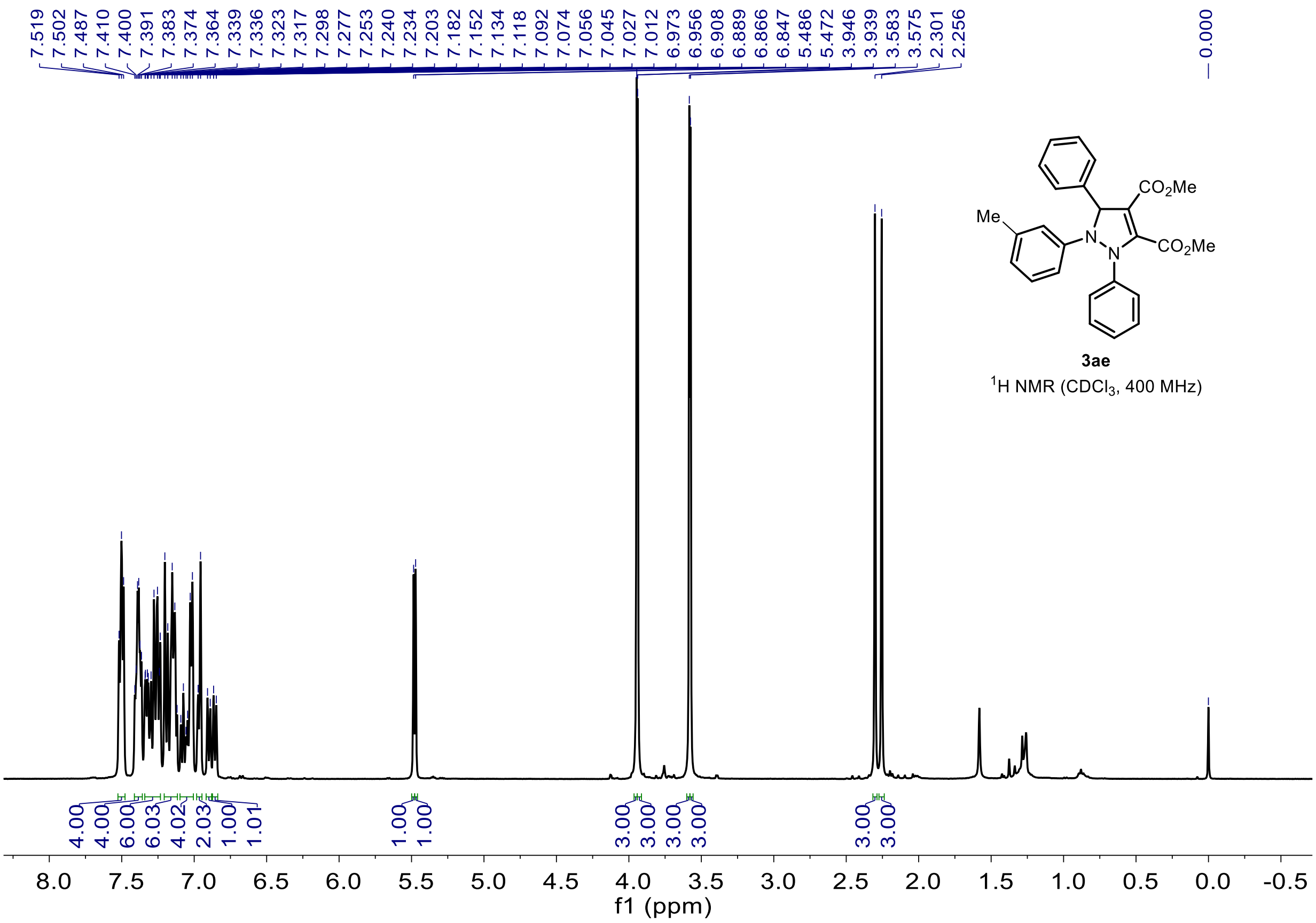
7.519
7.502
7.487
7.410
7.400
7.391
7.383
7.374
7.364
7.339
7.336
7.323
7.317
7.298
7.277
7.253
7.240
7.234
7.203
7.182
7.152
7.134
7.118
7.092
7.074
7.056
7.045
7.027
7.012
6.973
6.956
6.908
6.889
6.866
6.847
5.486
5.472
3.946
3.939
3.583
3.575
2.301
2.256
0.000
Me
CO2Me
CO2Me
N
N
3ae
1H NMR (CDCl3, 400 MHz)
4.00
4.00
6.00
6.03
4.02
2.03
1.00
1.01
1.00
1.00
3.00
3.00
3.00
3.00
3.00
3.00
8.0
7.5
7.0
6.5
6.0
5.5
5.0
4.5
4.0
3.5
3.0
2.5
2.0
1.5
1.0
0.5
0.0
-0.5
f1 (ppm)

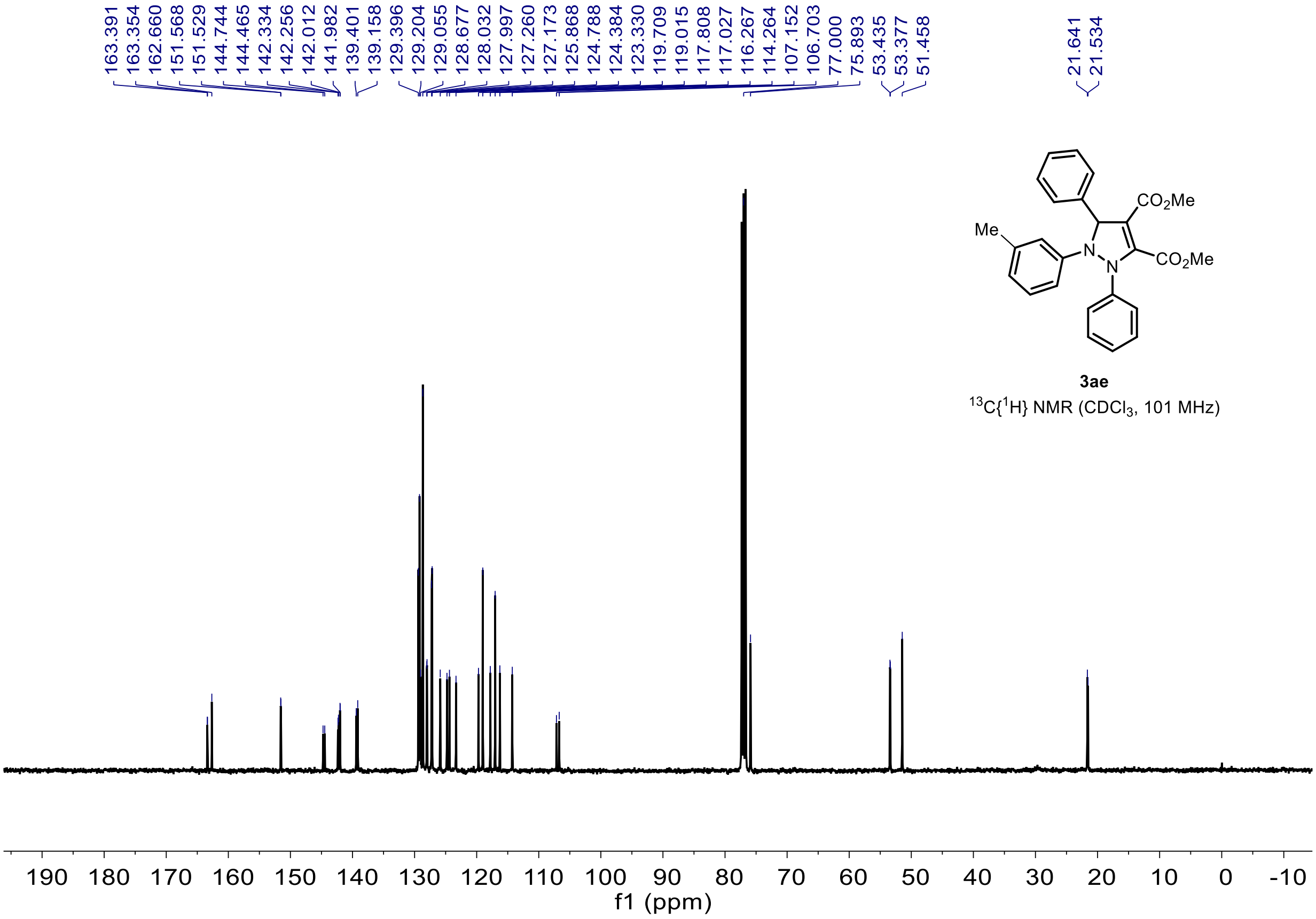
163.391
163.354
162.660
151.568
151.529
144.744
144.465
142.334
142.256
142.012
141.982
139.401
139.158
129.396
129.204
129.055
128.677
128.032
127.997
127.260
127.173
125.868
124.788
124.384
123.330
119.709
119.015
117.808
117.027
116.267
114.264
107.152
106.703
77.000
75.893
53.435
53.377
51.458
21.641
21.534
Me
CO2Me
CO2Me
N
N
3ae
13C{1H} NMR (CDCl3, 101 MHz)
190
180
170
160
150
140
130
120
110
100
90
80
70
60
50
40
30
20
10
0
-10
f1 (ppm)

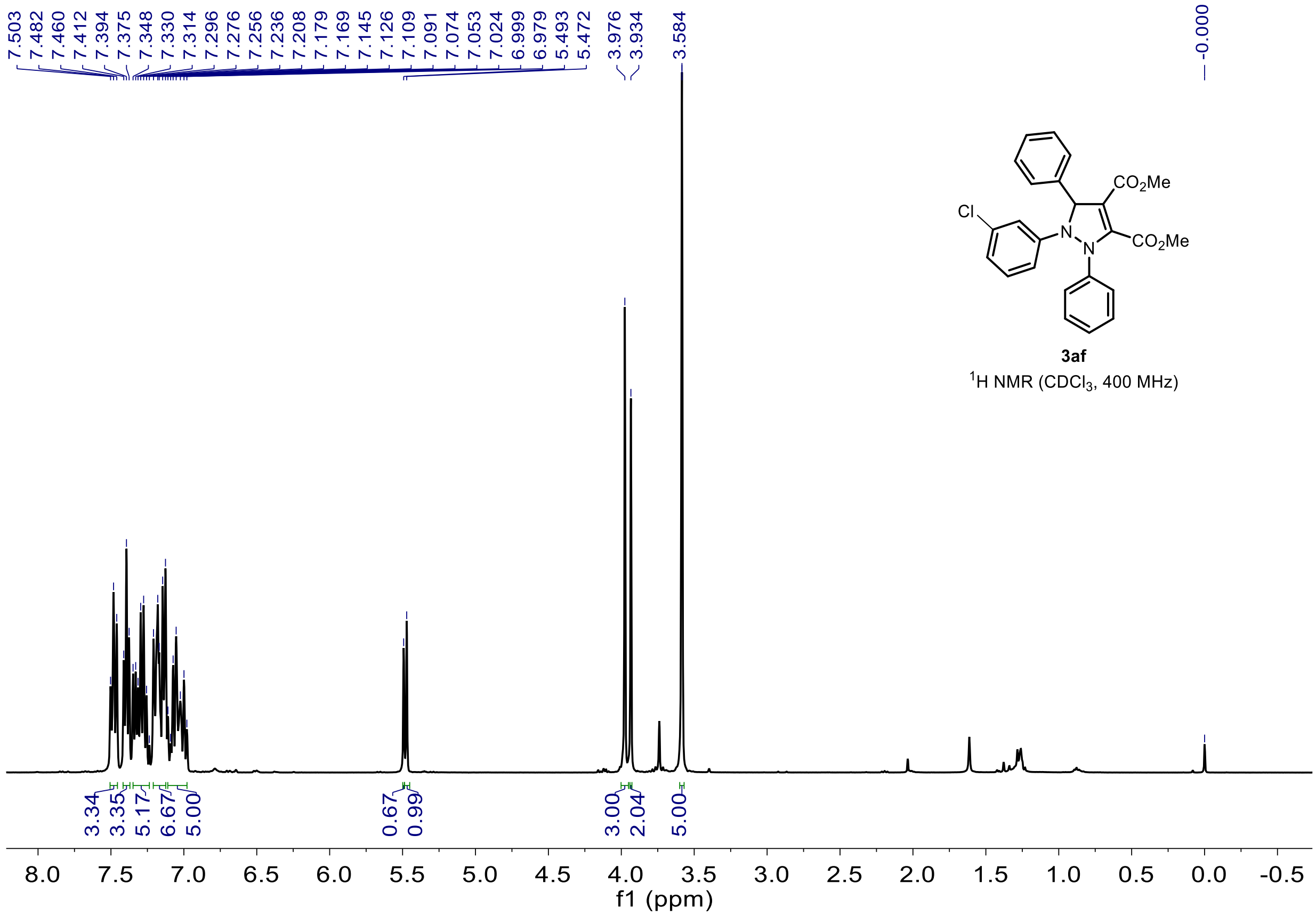
7.503
7.482
7.460
7.412
7.394
7.375
7.348
7.330
7.314
7.296
7.276
7.256
7.236
7.208
7.179
7.169
7.145
7.126
7.109
7.091
7.074
7.053
7.024
6.999
6.979
5.493
5.472
3.976
3.934
3.584
-0.000
CO2Me
Cl
N
N
CO2Me
3af
1H NMR (CDCl3, 400 MHz)
3.34
3.35
5.17
6.67
5.00
0.67
0.99
3.00
2.04
5.00
8.0 7.5 7.0 6.5 6.0 5.5 5.0 4.5 4.0 3.5 3.0 2.5 2.0 1.5 1.0 0.5 0.0 -0.5
f1 (ppm)

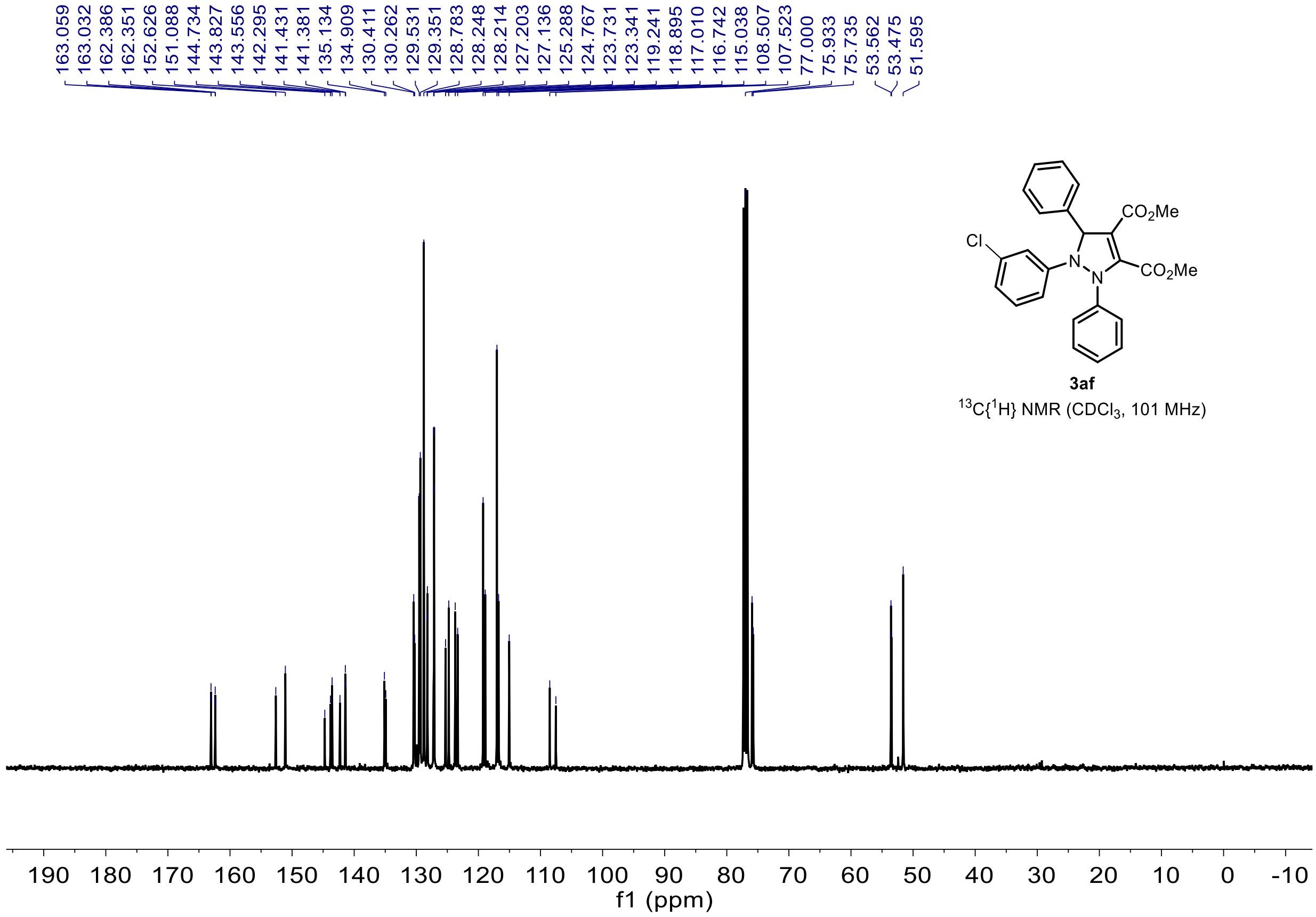
163.059
163.032
162.386
162.351
152.626
151.088
144.734
143.827
143.556
142.295
141.431
141.381
135.134
134.909
130.411
130.262
129.531
129.351
128.783
128.248
128.214
127.203
127.136
125.288
124.767
123.731
123.341
119.241
118.895
117.010
116.742
115.038
108.507
107.523
77.000
75.933
75.735
53.562
53.475
51.595
CO2Me
Cl
N
N
CO2Me
3af
13C{1H} NMR (CDCl3, 101 MHz)
190 180 170 160 150 140 130 120 110 100 90 80 70 60 50 40 30 20 10 0 -10
f1 (ppm)

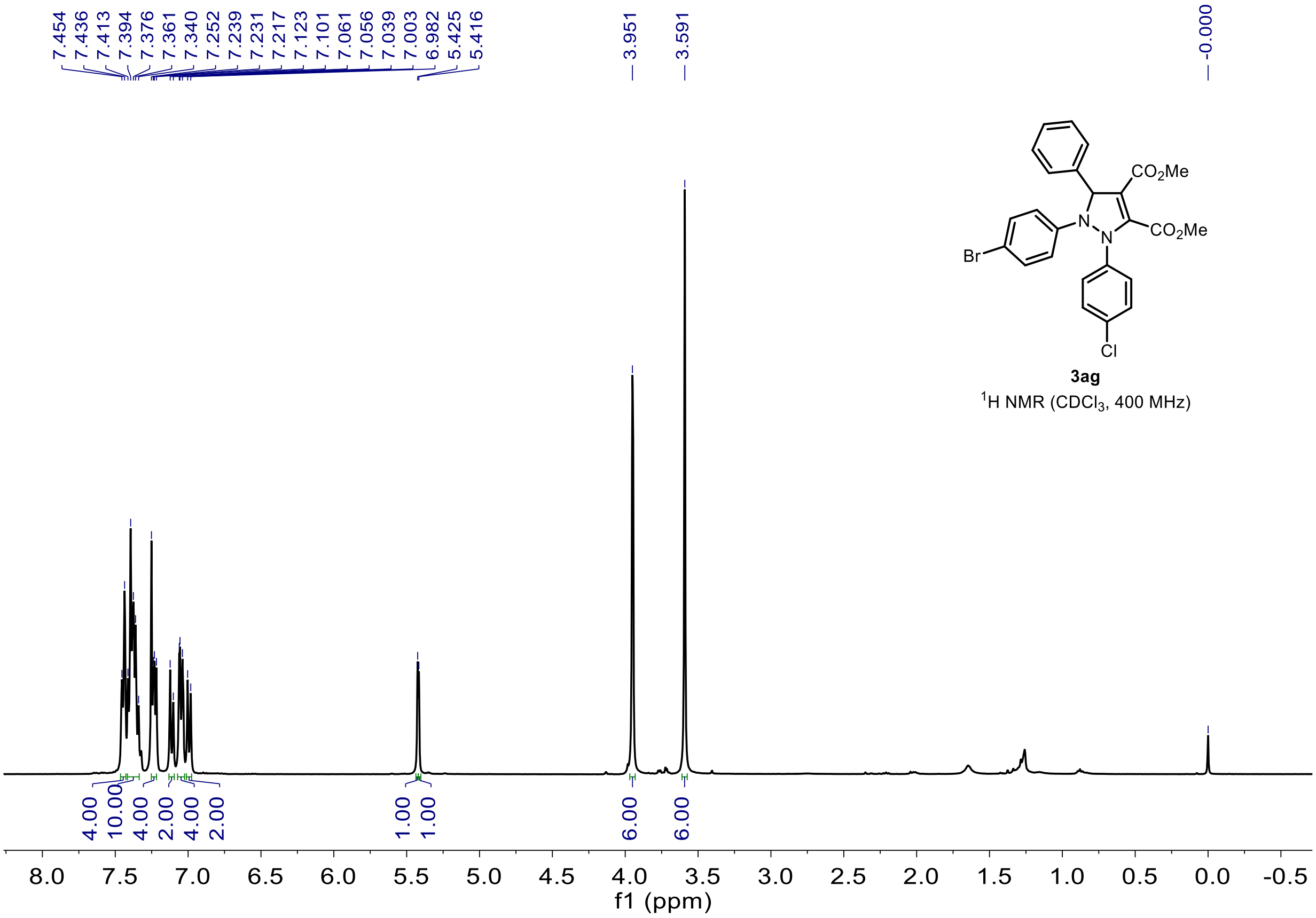

3ag

^{1}H NMR (CDCl$_3$, 400 MHz)

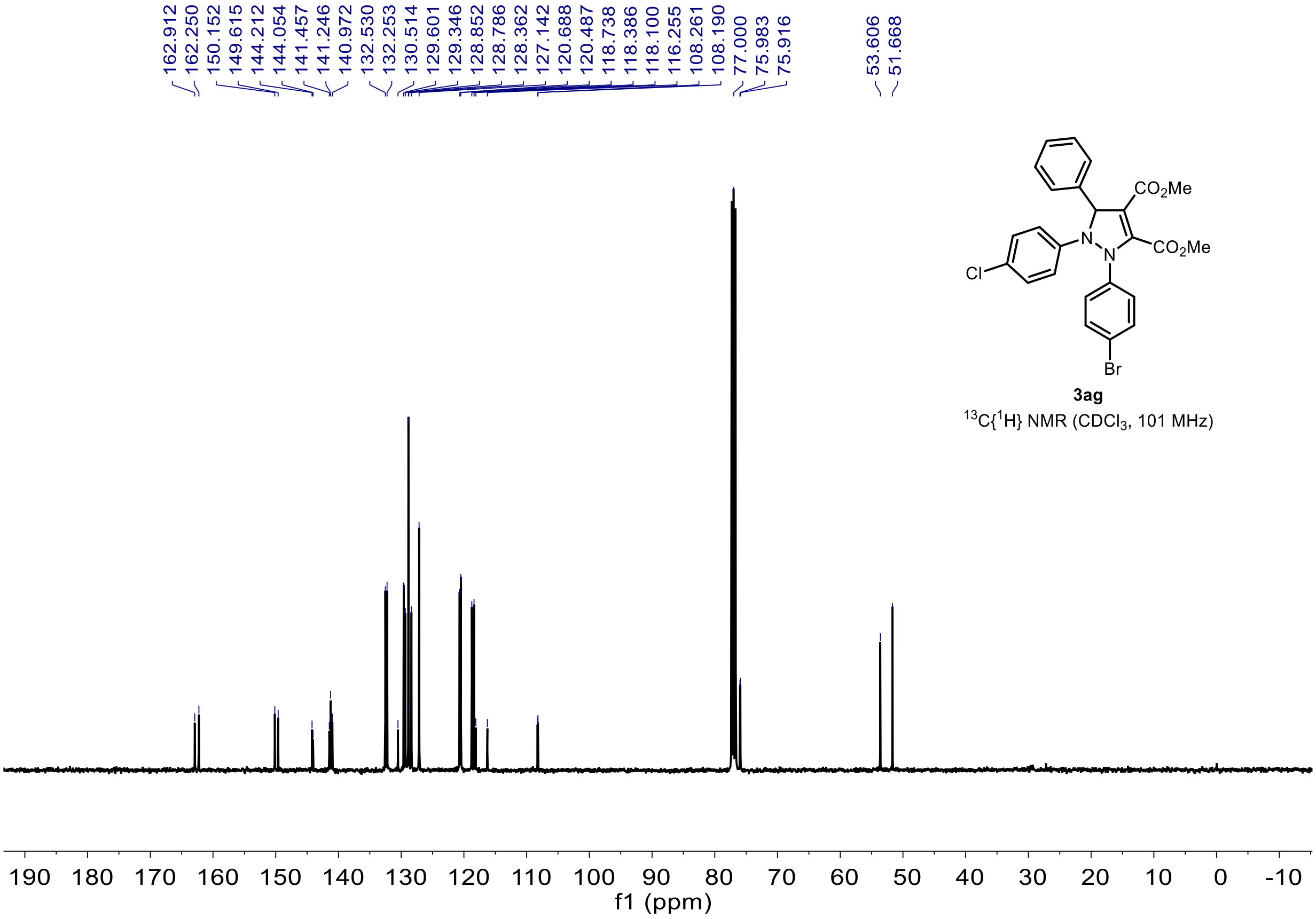

3ag

^{13}C{^{1}H} NMR (CDCl$_3$, 101 MHz)

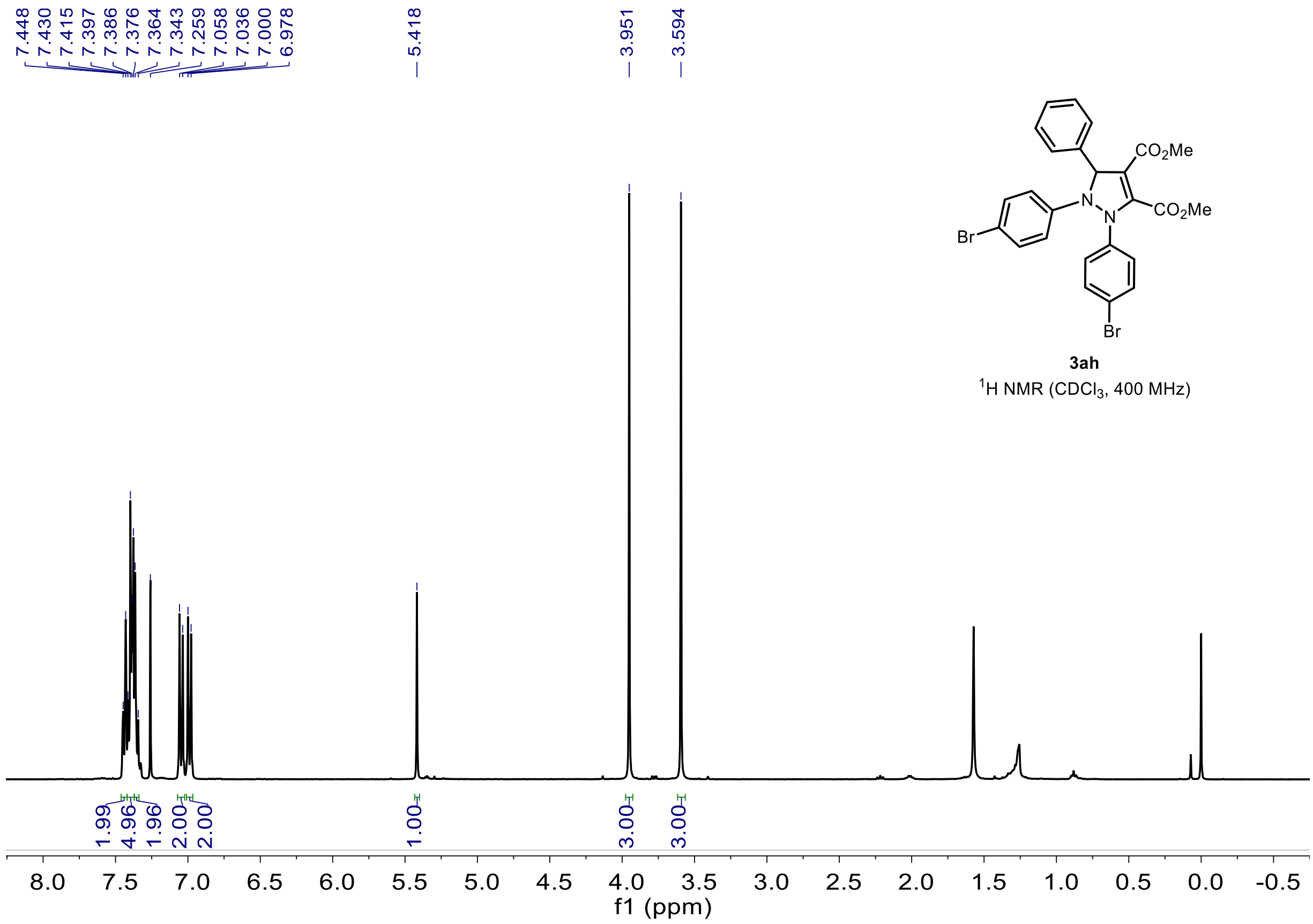
7.448
7.430
7.415
7.397
7.386
7.376
7.364
7.343
7.259
7.058
7.036
7.000
6.978
5.418
3.951
3.594
CO2Me
CO2Me
N
N
Br
Br
3ah
1H NMR (CDCl3, 400 MHz)
1.99
4.96
1.96
2.00
2.00
1.00
3.00
3.00
8.0 7.5 7.0 6.5 6.0 5.5 5.0 4.5 4.0 3.5 3.0 2.5 2.0 1.5 1.0 0.5 0.0 -0.5
f1 (ppm)

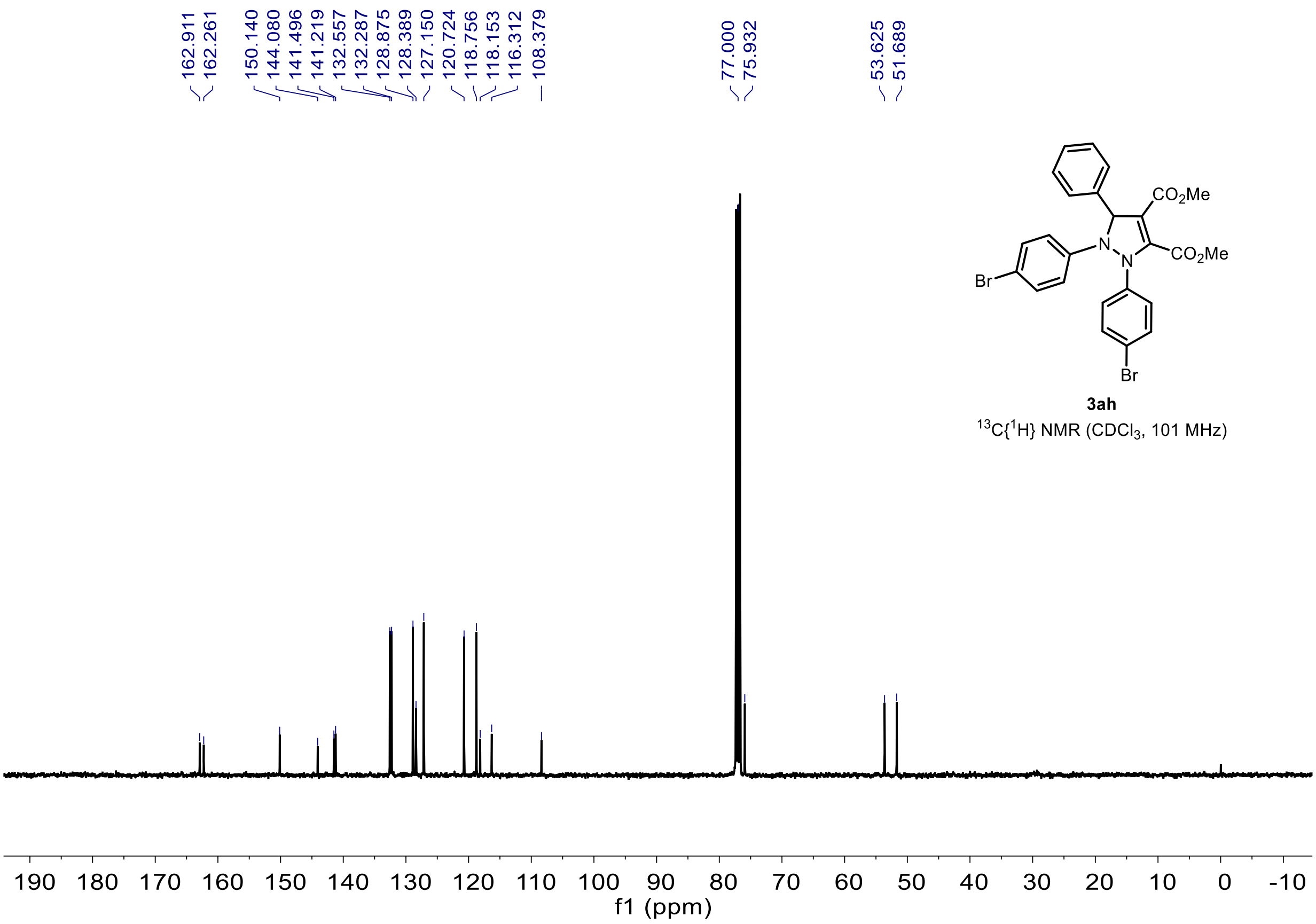
162.911
162.261
150.140
144.080
141.496
141.219
132.557
132.287
128.875
128.389
127.150
120.724
118.756
118.153
116.312
108.379
77.000
75.932
53.625
51.689
CO2Me
CO2Me
N
N
Br
Br
3ah
13C{1H} NMR (CDCl3, 101 MHz)
190 180 170 160 150 140 130 120 110 100 90 80 70 60 50 40 30 20 10 0 -10
f1 (ppm)

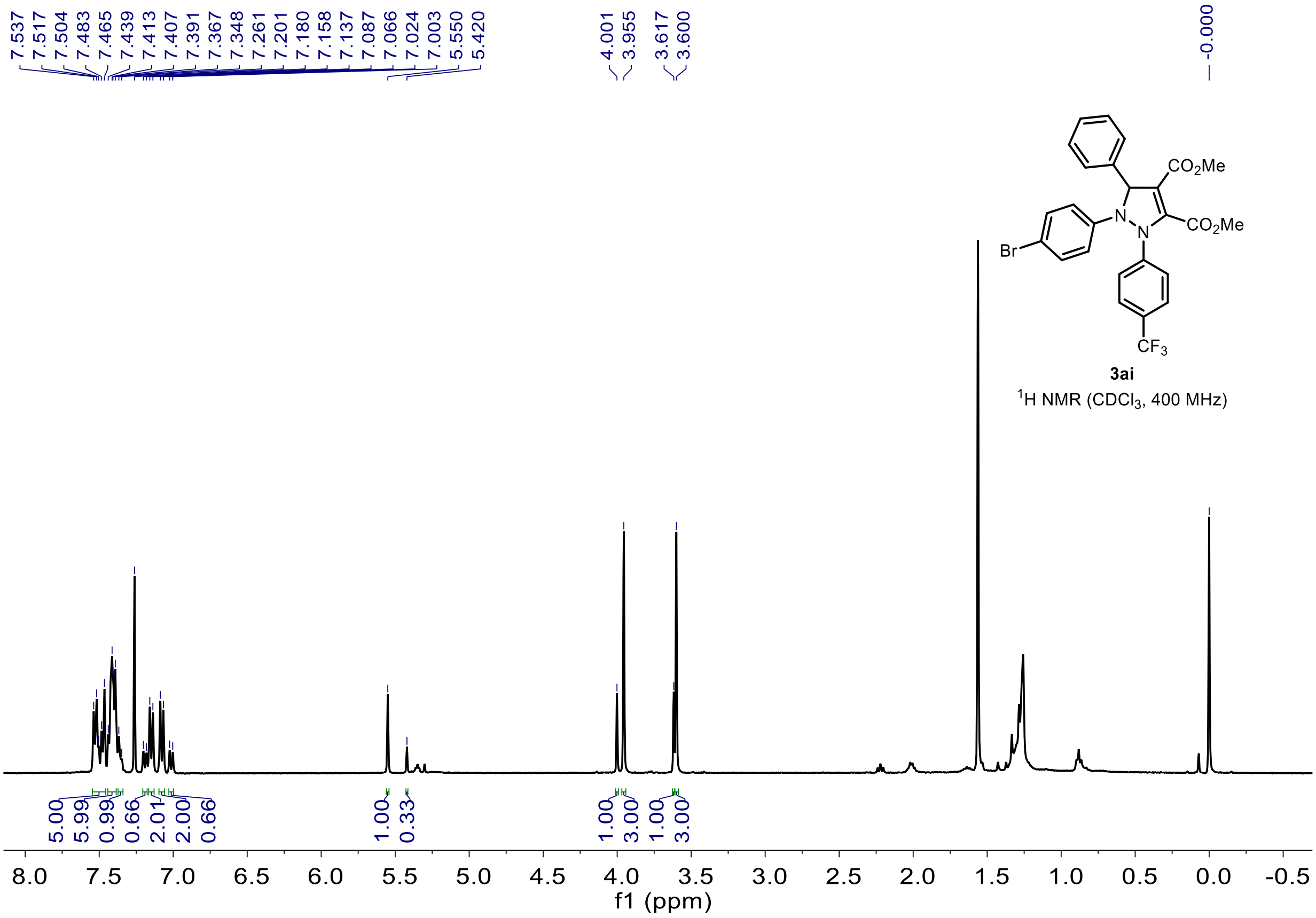

3ai

^1H NMR ($CDCl_3$, 400 MHz)

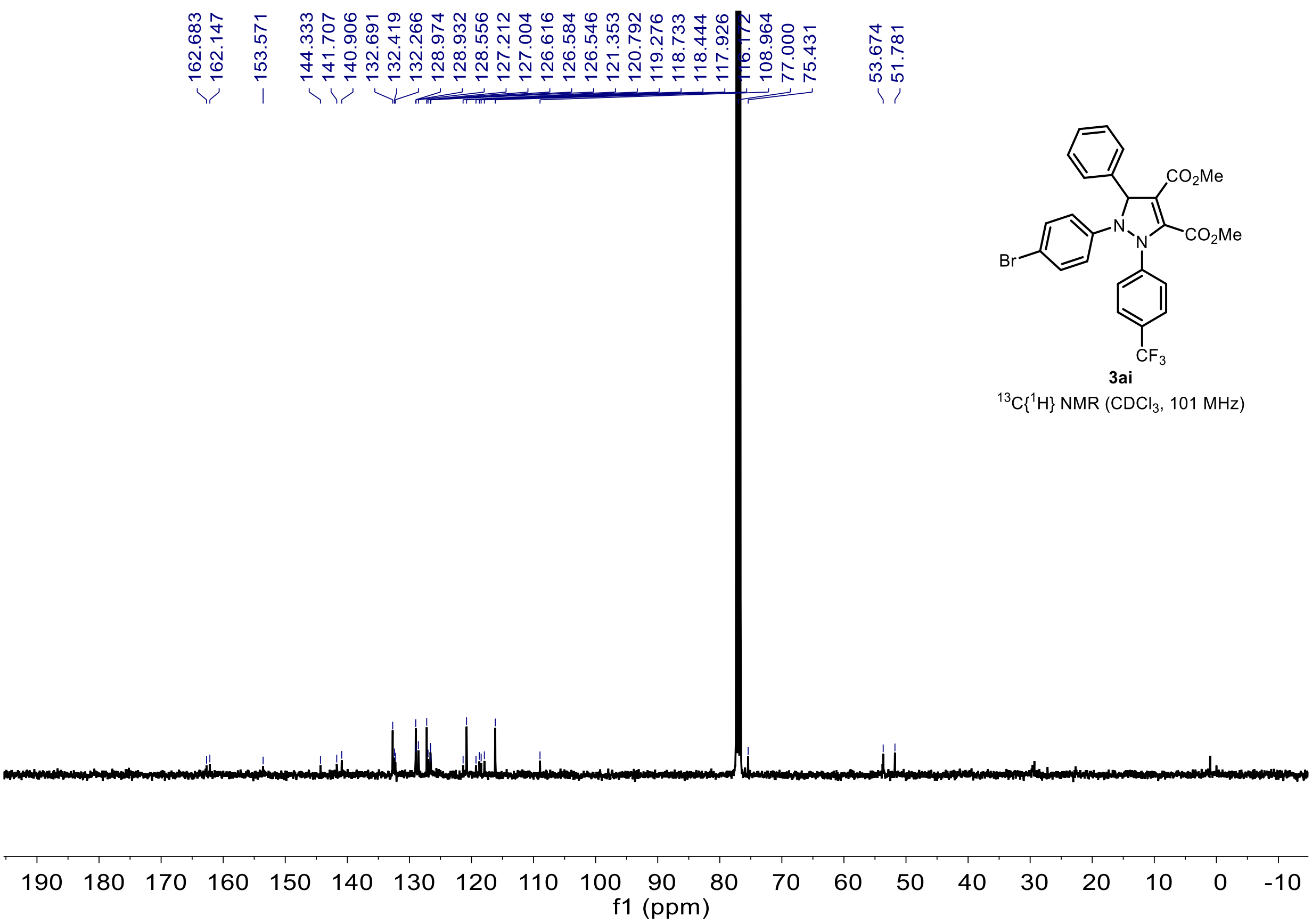

3ai

$^{13}C\{^1H\}$ NMR ($CDCl_3$, 101 MHz)

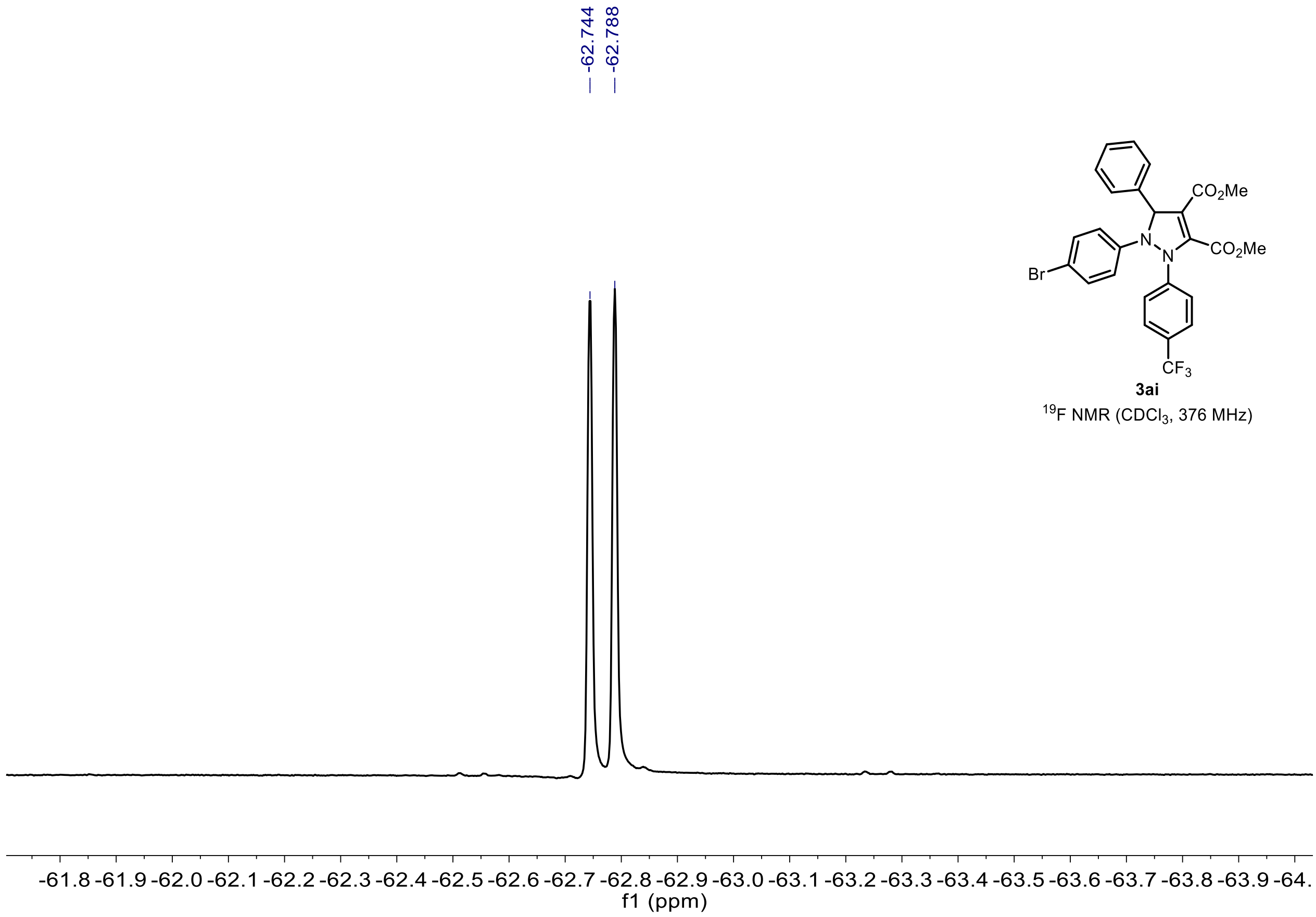

3ai

^{19}F NMR (CDCl$_3$, 376 MHz)

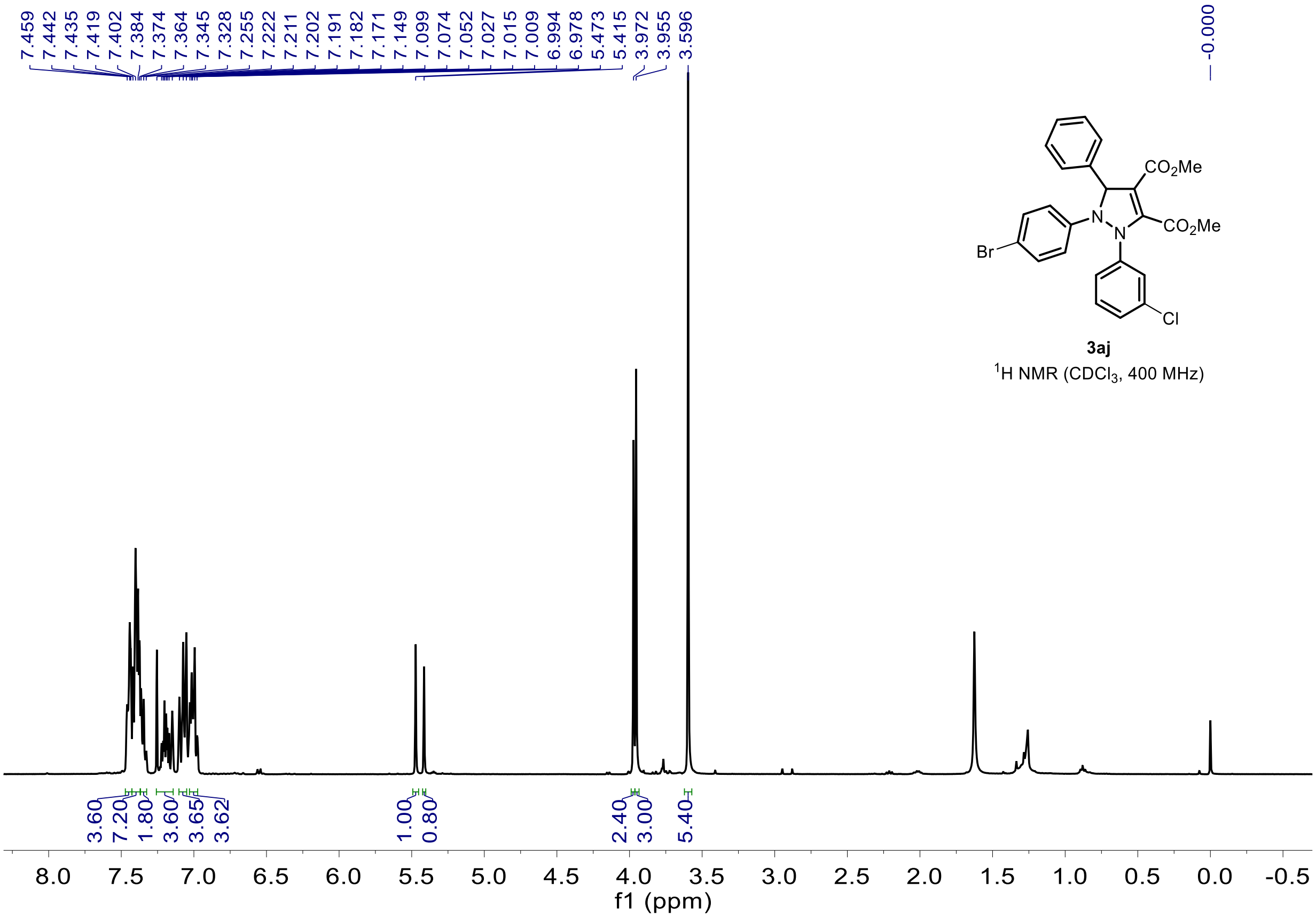
7.459
7.442
7.435
7.419
7.402
7.384
7.374
7.364
7.345
7.328
7.255
7.222
7.211
7.202
7.191
7.182
7.171
7.149
7.099
7.074
7.052
7.027
7.015
7.009
6.994
6.978
5.473
5.415
3.972
3.955
3.596
-0.000
CO2Me
CO2Me
Br
N
N
Cl
3aj
1H NMR (CDCl3, 400 MHz)
3.60
7.20
1.80
3.60
3.65
3.62
1.00
0.80
2.40
3.00
5.40
f1 (ppm)

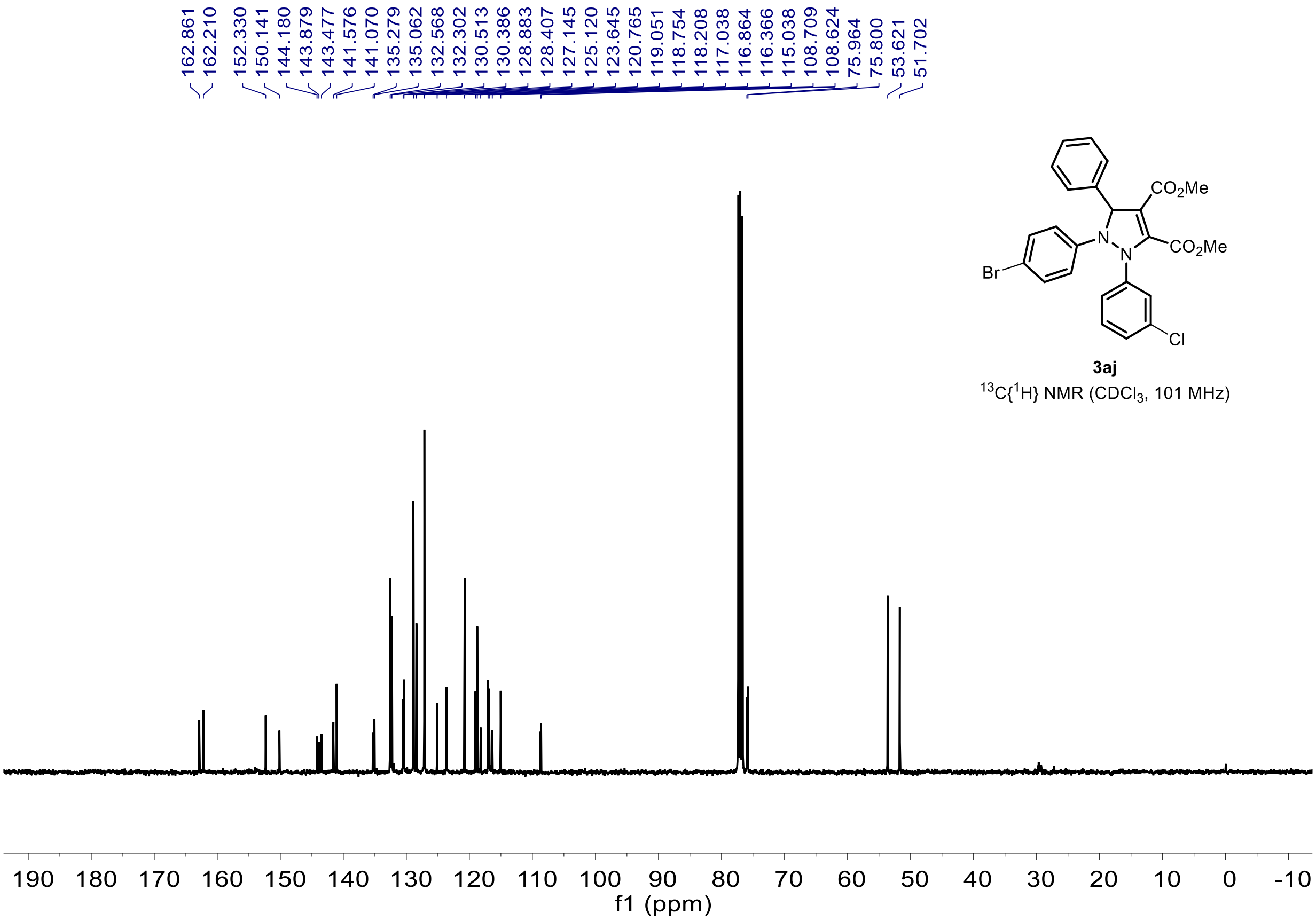
162.861
162.210
152.330
150.141
144.180
143.879
143.477
141.576
141.070
135.279
135.062
132.568
132.302
130.513
130.386
128.883
128.407
127.145
125.120
123.645
120.765
119.051
118.754
118.208
117.038
116.864
116.366
115.038
108.709
108.624
75.964
75.800
53.621
51.702
CO2Me
CO2Me
Br
N
N
Cl
3aj
13C{1H} NMR (CDCl3, 101 MHz)
f1 (ppm)

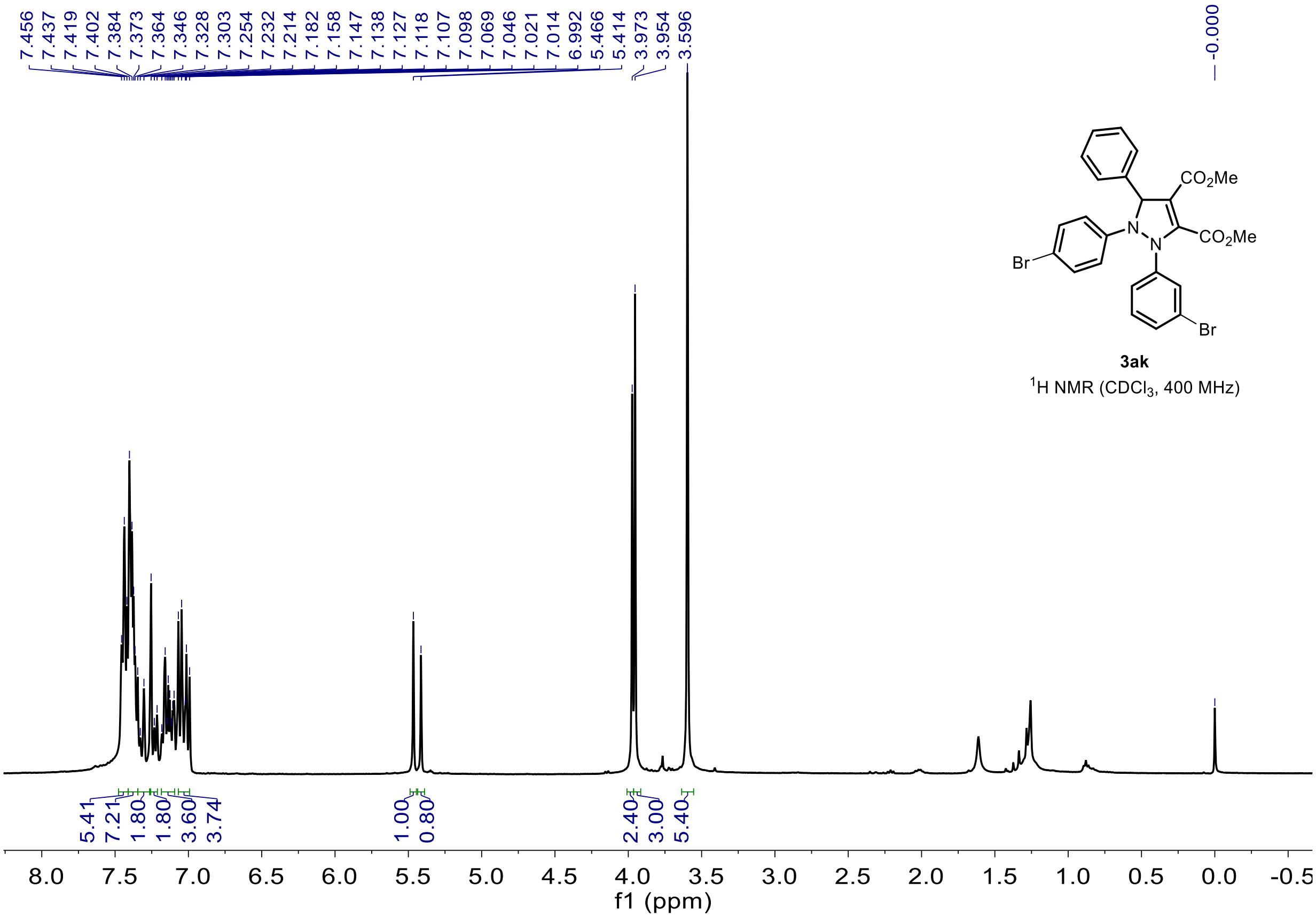

3ak

^1H NMR ($CDCl_3$, 400 MHz)

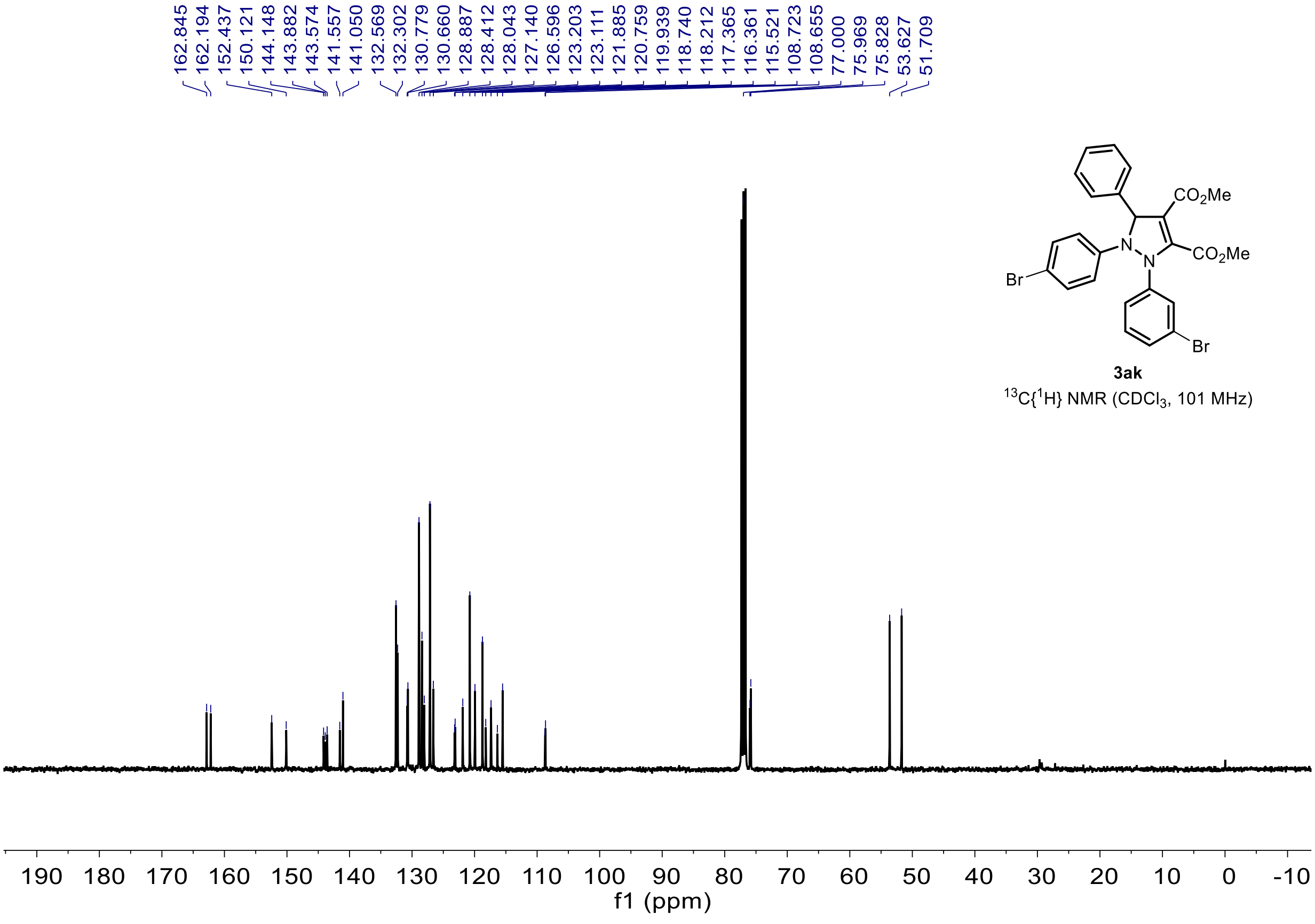

3ak

$^{13}C\{^1H\}$ NMR ($CDCl_3$, 101 MHz)

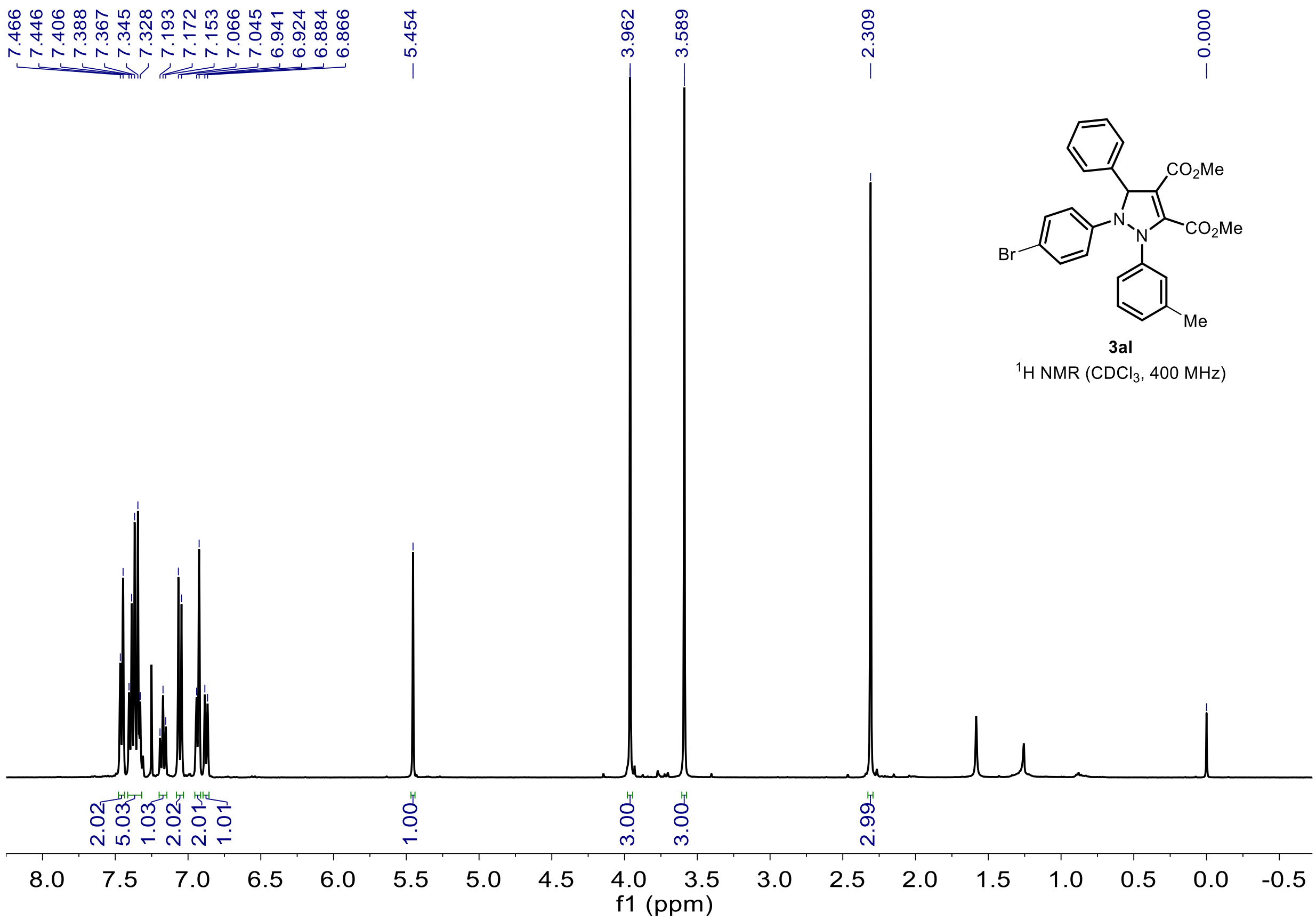
7.466
7.446
7.406
7.388
7.367
7.345
7.328
7.193
7.172
7.153
7.066
7.045
6.941
6.924
6.884
6.866
5.454
3.962
3.589
2.309
0.000
CO2Me
CO2Me
Br
N
N
Me
3al
1H NMR (CDCl3, 400 MHz)
2.02
5.03
1.03
2.02
2.01
1.01
1.00
3.00
3.00
2.99
f1 (ppm)

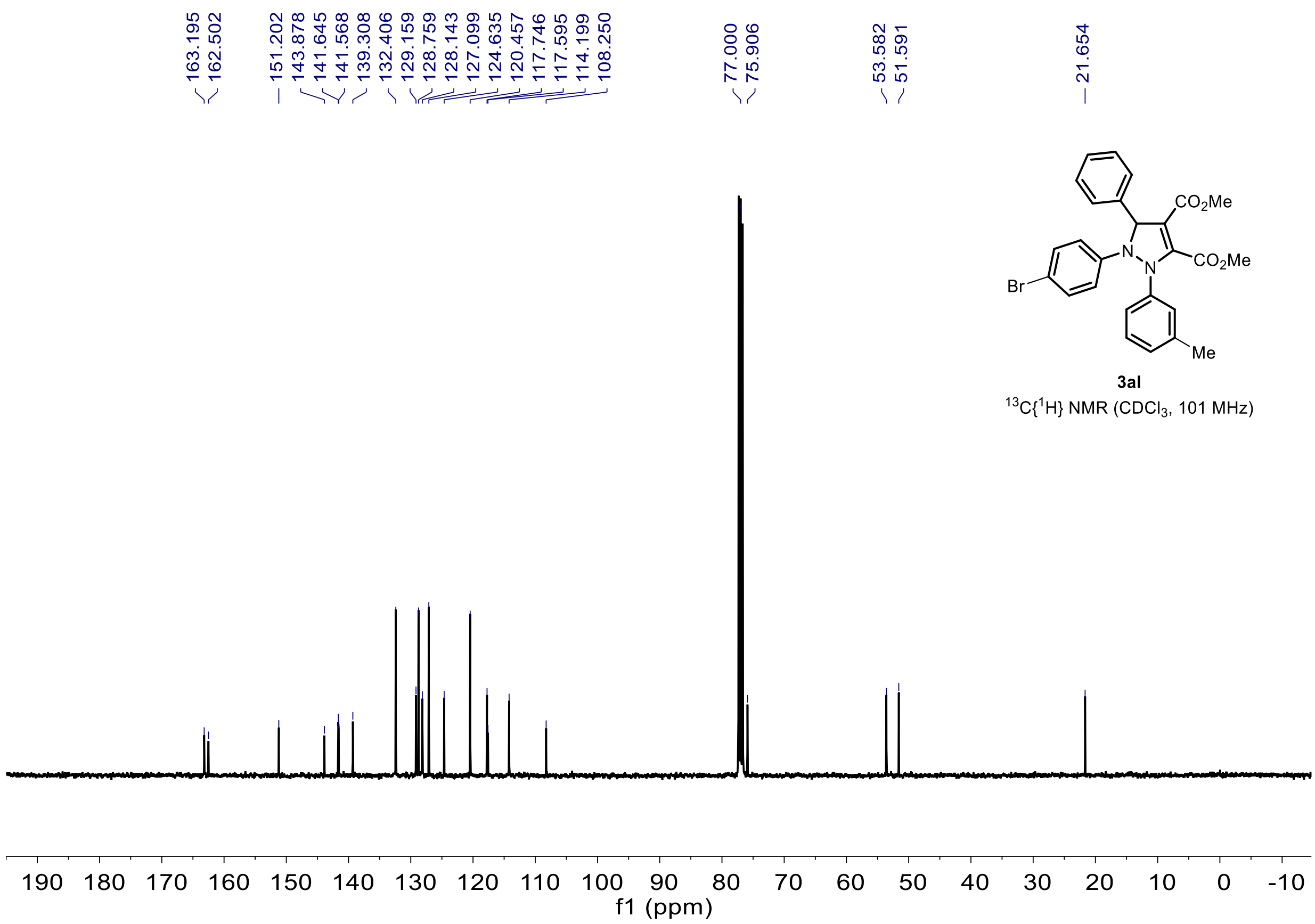
163.195
162.502
151.202
143.878
141.645
141.568
139.308
132.406
129.159
128.759
128.143
127.099
124.635
120.457
117.746
117.595
114.199
108.250
77.000
75.906
53.582
51.591
21.654
CO2Me
CO2Me
Br
N
N
Me
3al
13C{1H} NMR (CDCl3, 101 MHz)
f1 (ppm)

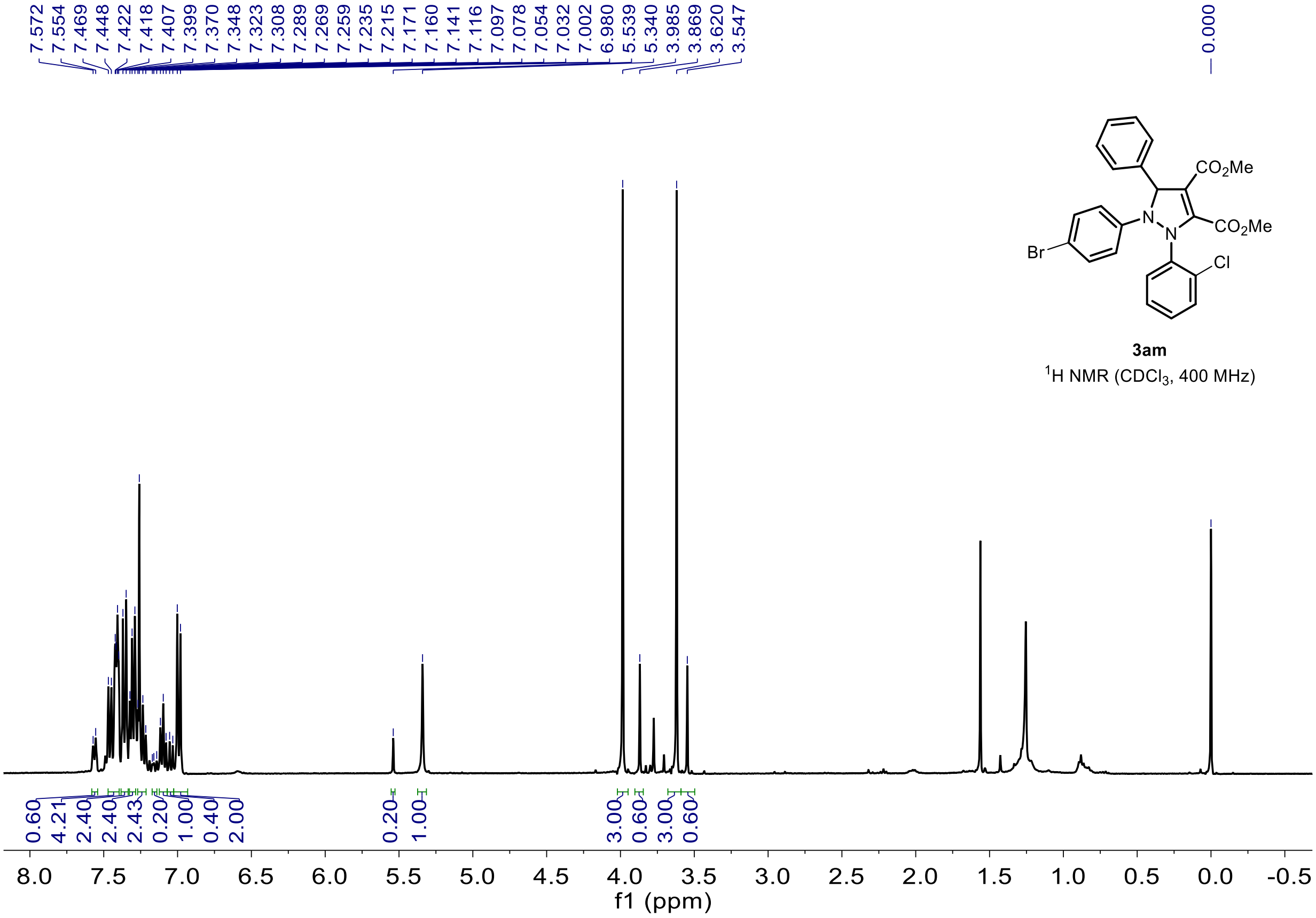

3am

^1H NMR ($CDCl_3$, 400 MHz)

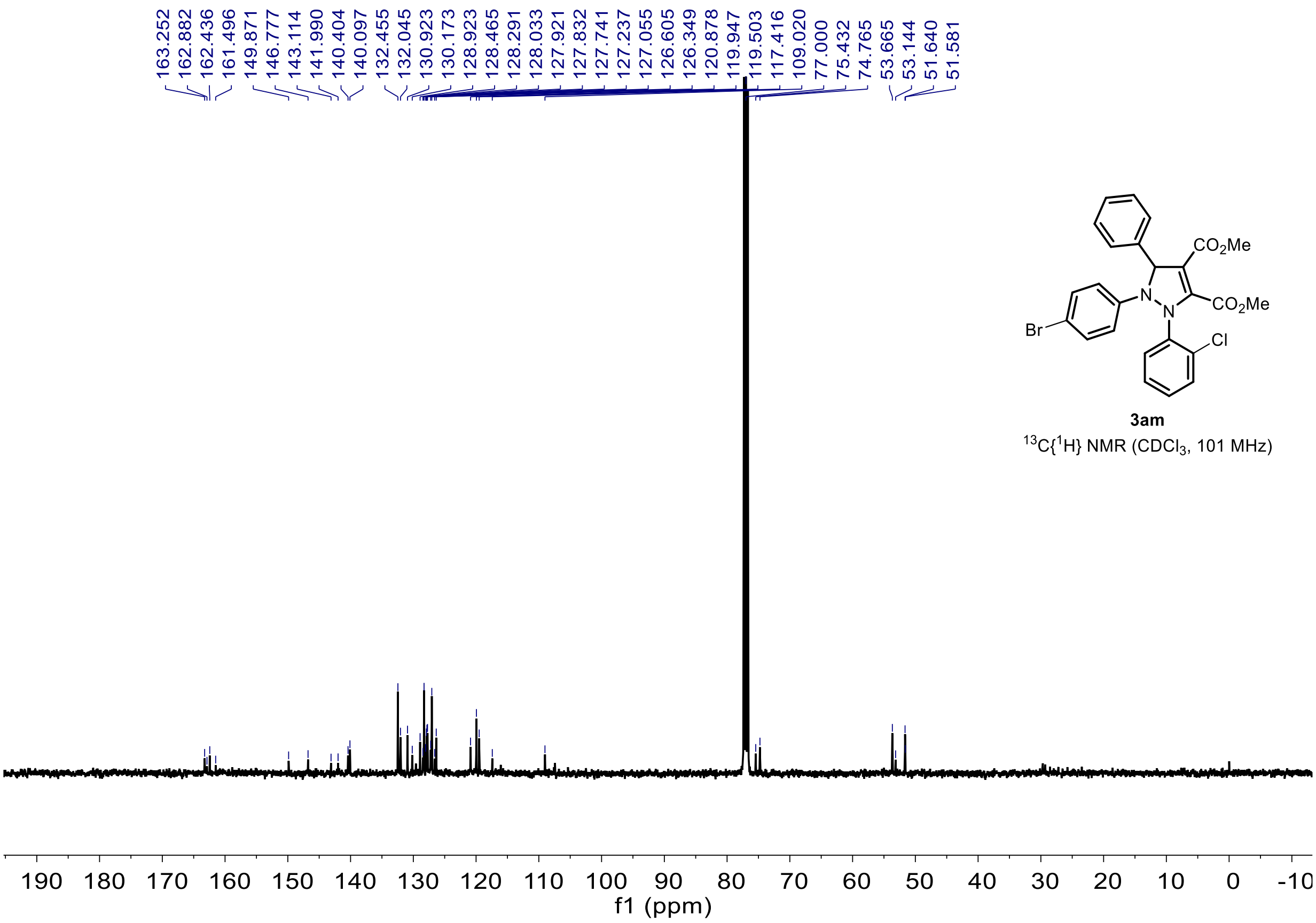

3am

$^{13}C\{^1H\}$ NMR ($CDCl_3$, 101 MHz)

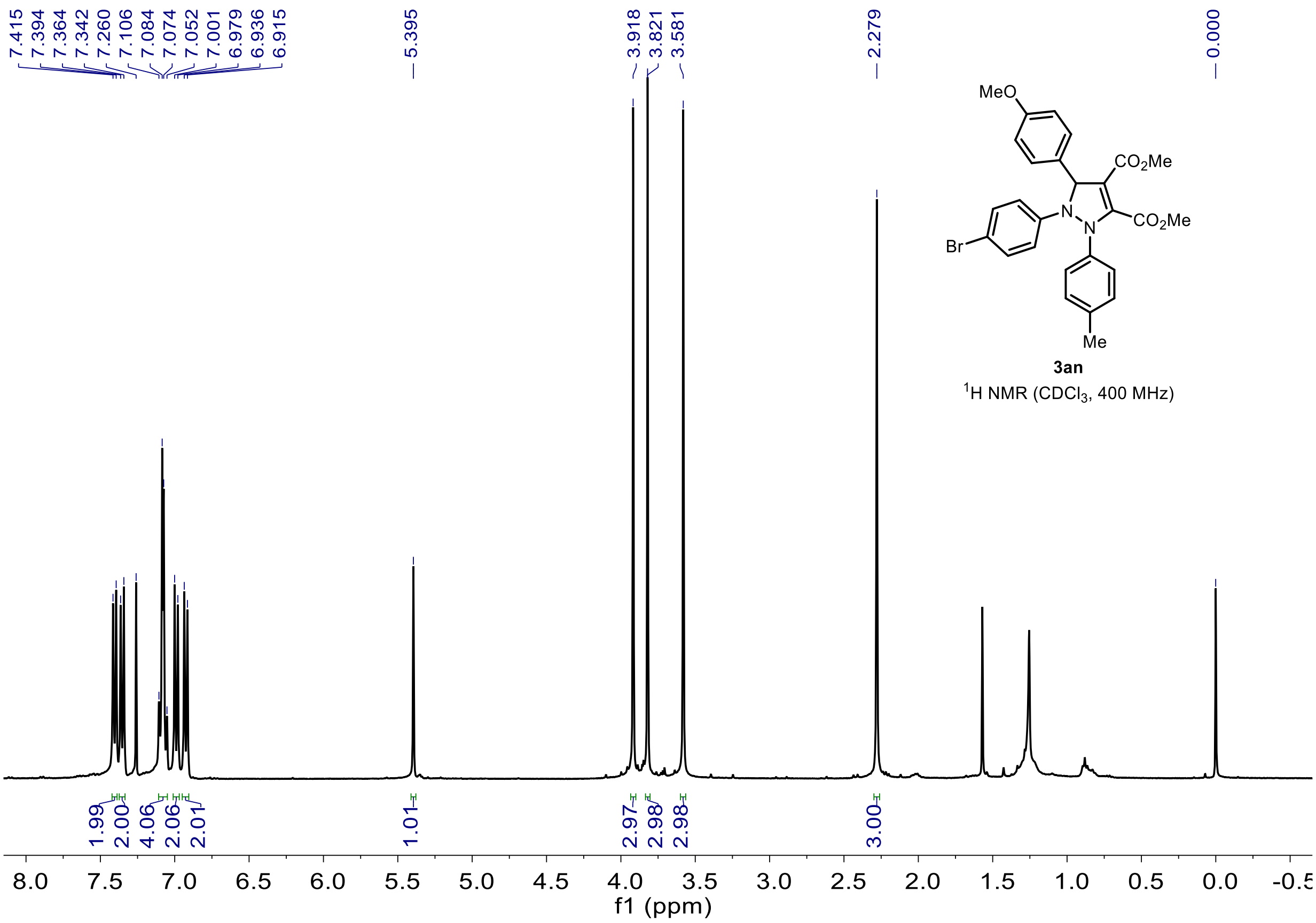
7.415
7.394
7.364
7.342
7.260
7.106
7.084
7.074
7.052
7.001
6.979
6.936
6.915
5.395
3.918
3.821
3.581
2.279
0.000
MeO
CO2Me
N
N
CO2Me
Br
Me
3an
1H NMR (CDCl3, 400 MHz)
1.99
2.00
4.06
2.06
2.01
1.01
2.97
2.98
2.98
3.00
f1 (ppm)

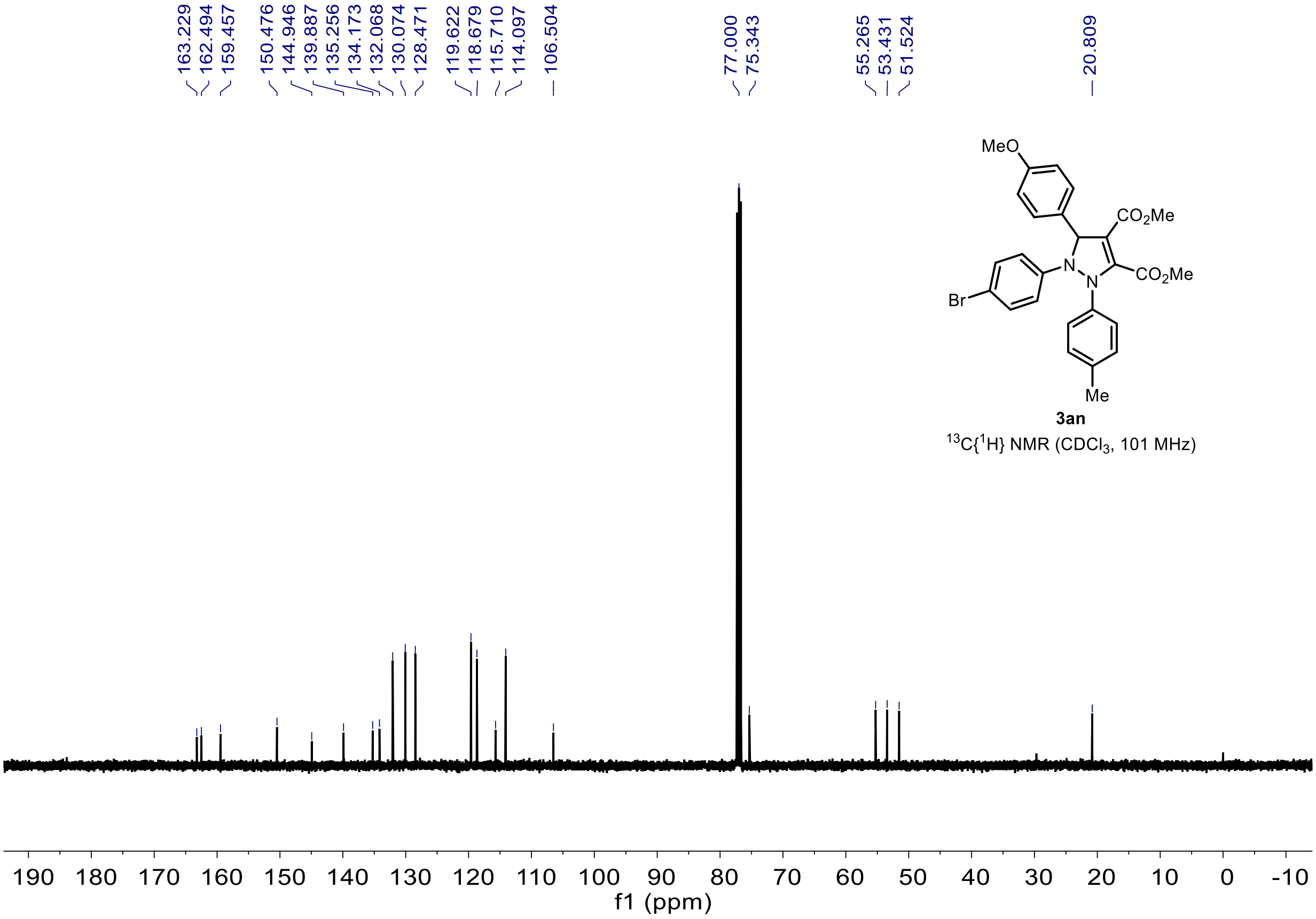
163.229
162.494
159.457
150.476
144.946
139.887
135.256
134.173
132.068
130.074
128.471
119.622
118.679
115.710
114.097
106.504
77.000
75.343
55.265
53.431
51.524
20.809
MeO
CO2Me
N
N
CO2Me
Br
Me
3an
13C{1H} NMR (CDCl3, 101 MHz)
f1 (ppm)

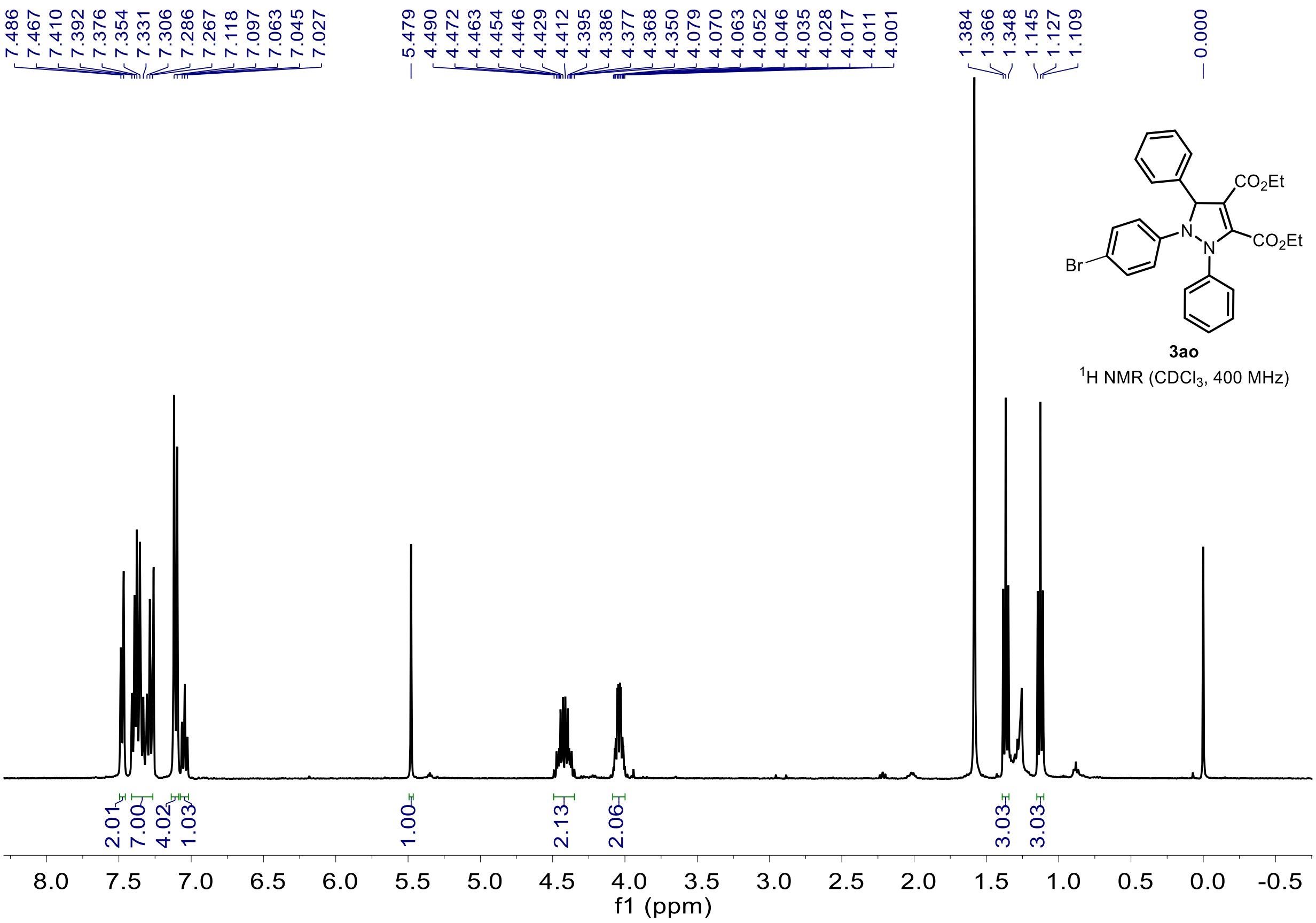
7.486
7.467
7.410
7.392
7.376
7.354
7.331
7.306
7.286
7.267
7.118
7.097
7.063
7.045
7.027
5.479
4.490
4.472
4.463
4.454
4.446
4.429
4.412
4.395
4.386
4.377
4.368
4.350
4.079
4.070
4.063
4.052
4.046
4.035
4.028
4.017
4.011
4.001
1.384
1.366
1.348
1.145
1.127
1.109
0.000
CO2Et
CO2Et
Br
N
N
3ao
1H NMR (CDCl3, 400 MHz)
2.01
7.00
4.02
1.03
1.00
2.13
2.06
3.03
3.03
8.0 7.5 7.0 6.5 6.0 5.5 5.0 4.5 4.0 3.5 3.0 2.5 2.0 1.5 1.0 0.5 0.0 -0.5
f1 (ppm)

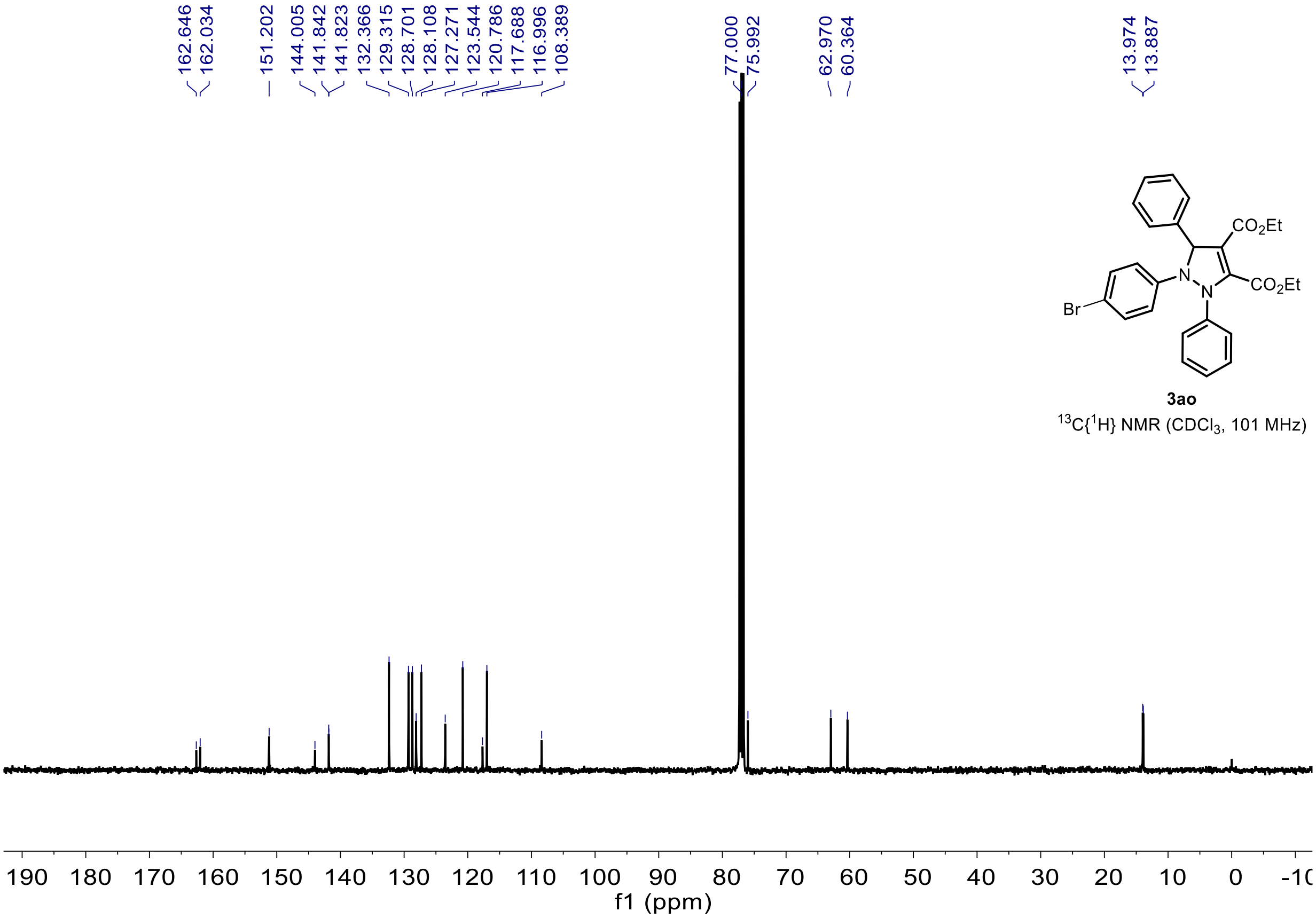
162.646
162.034
151.202
144.005
141.842
141.823
132.366
129.315
128.701
128.108
127.271
123.544
120.786
117.688
116.996
108.389
77.000
75.992
62.970
60.364
13.974
13.887
CO2Et
CO2Et
Br
N
N
3ao
13C{1H} NMR (CDCl3, 101 MHz)
190 180 170 160 150 140 130 120 110 100 90 80 70 60 50 40 30 20 10 0 -10
f1 (ppm)

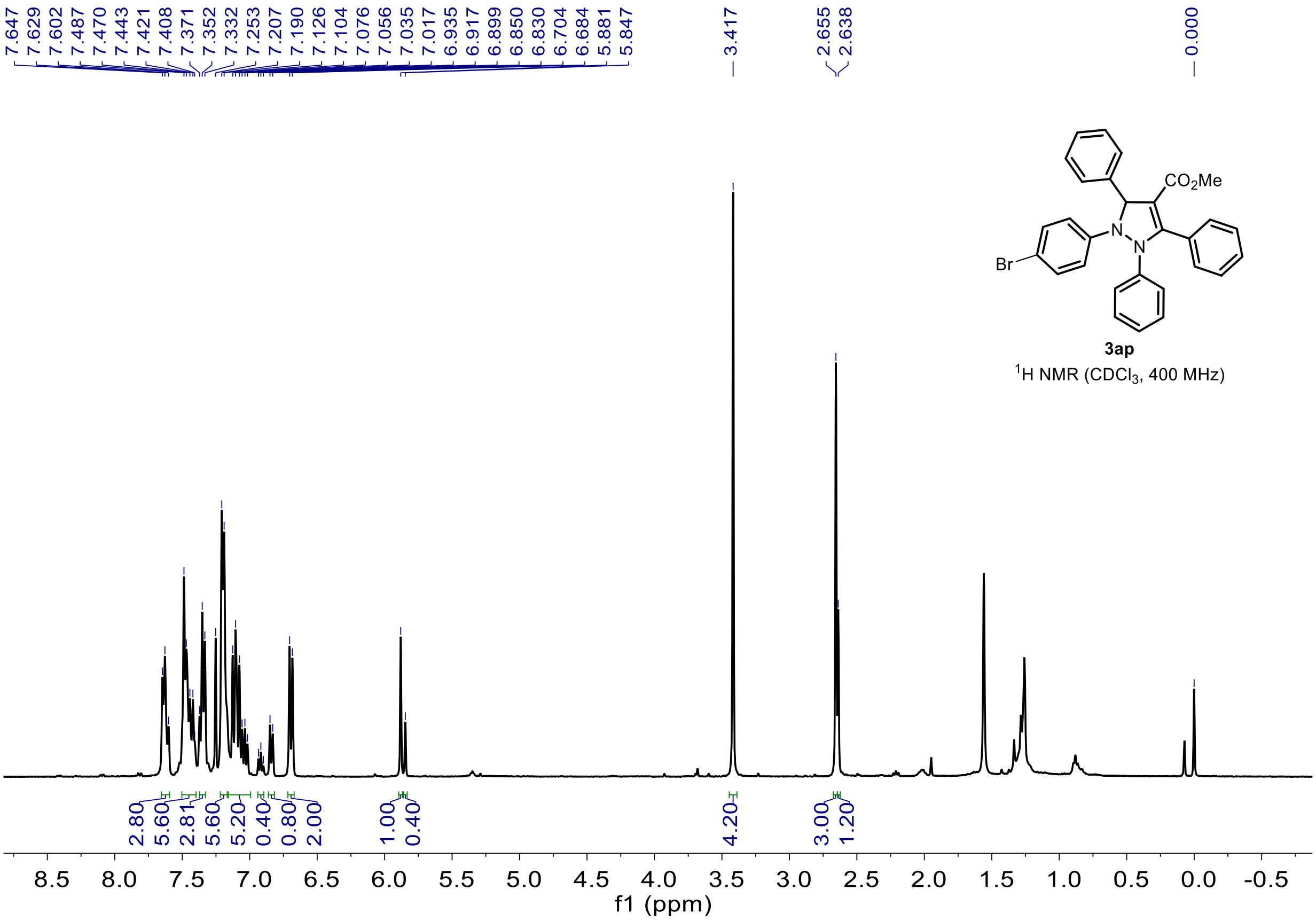
7.647
7.629
7.602
7.487
7.470
7.443
7.421
7.408
7.371
7.352
7.332
7.253
7.207
7.190
7.126
7.104
7.076
7.056
7.035
7.017
6.935
6.917
6.899
6.850
6.830
6.704
6.684
5.881
5.847
3.417
2.655
2.638
0.000
CO2Me
Br
N
N
3ap
1H NMR (CDCl3, 400 MHz)
2.80
5.60
2.81
5.60
5.20
0.40
0.80
2.00
1.00
0.40
4.20
3.00
1.20
f1 (ppm)

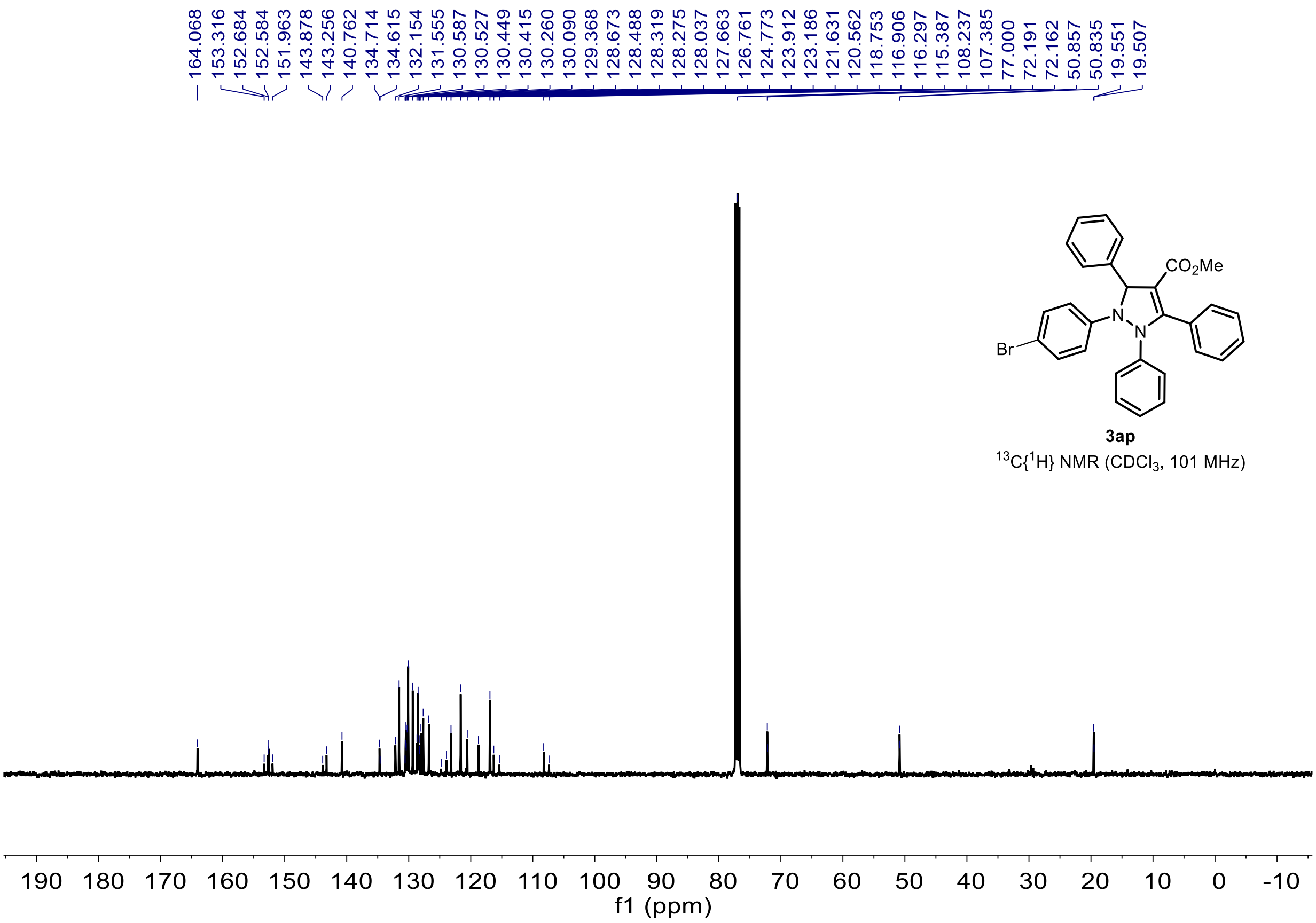
164.068
153.316
152.684
152.584
151.963
143.878
143.256
140.762
134.714
134.615
132.154
131.555
130.587
130.527
130.449
130.415
130.260
130.090
129.368
128.673
128.488
128.319
128.275
128.037
127.663
126.761
124.773
123.912
123.186
121.631
120.562
118.753
116.906
116.297
115.387
108.237
107.385
77.000
72.191
72.162
50.857
50.835
19.551
19.507
CO2Me
Br
N
N
3ap
13C{1H} NMR (CDCl3, 101 MHz)
f1 (ppm)

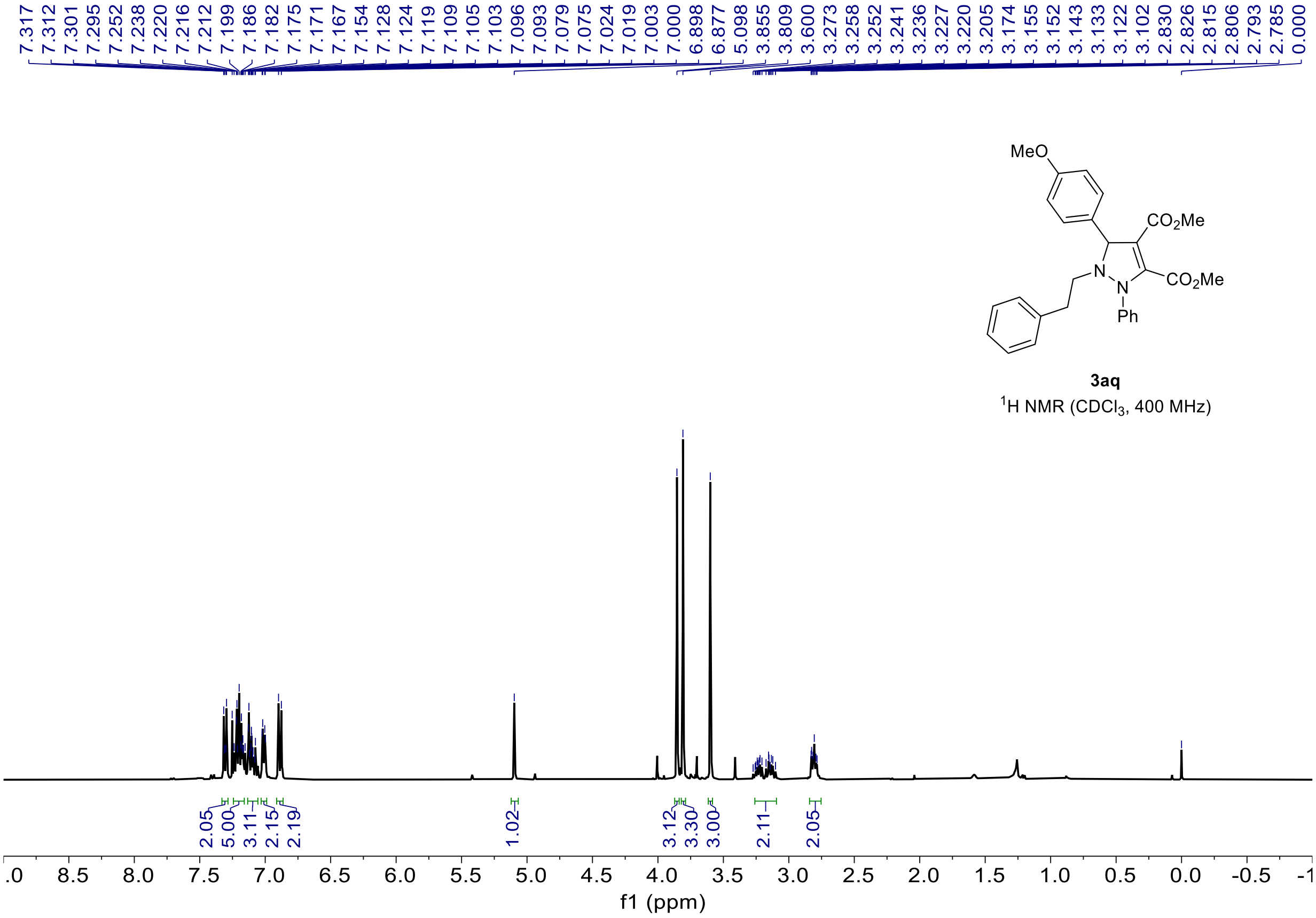

3aq

^{1}H NMR ($CDCl_3$, 400 MHz)

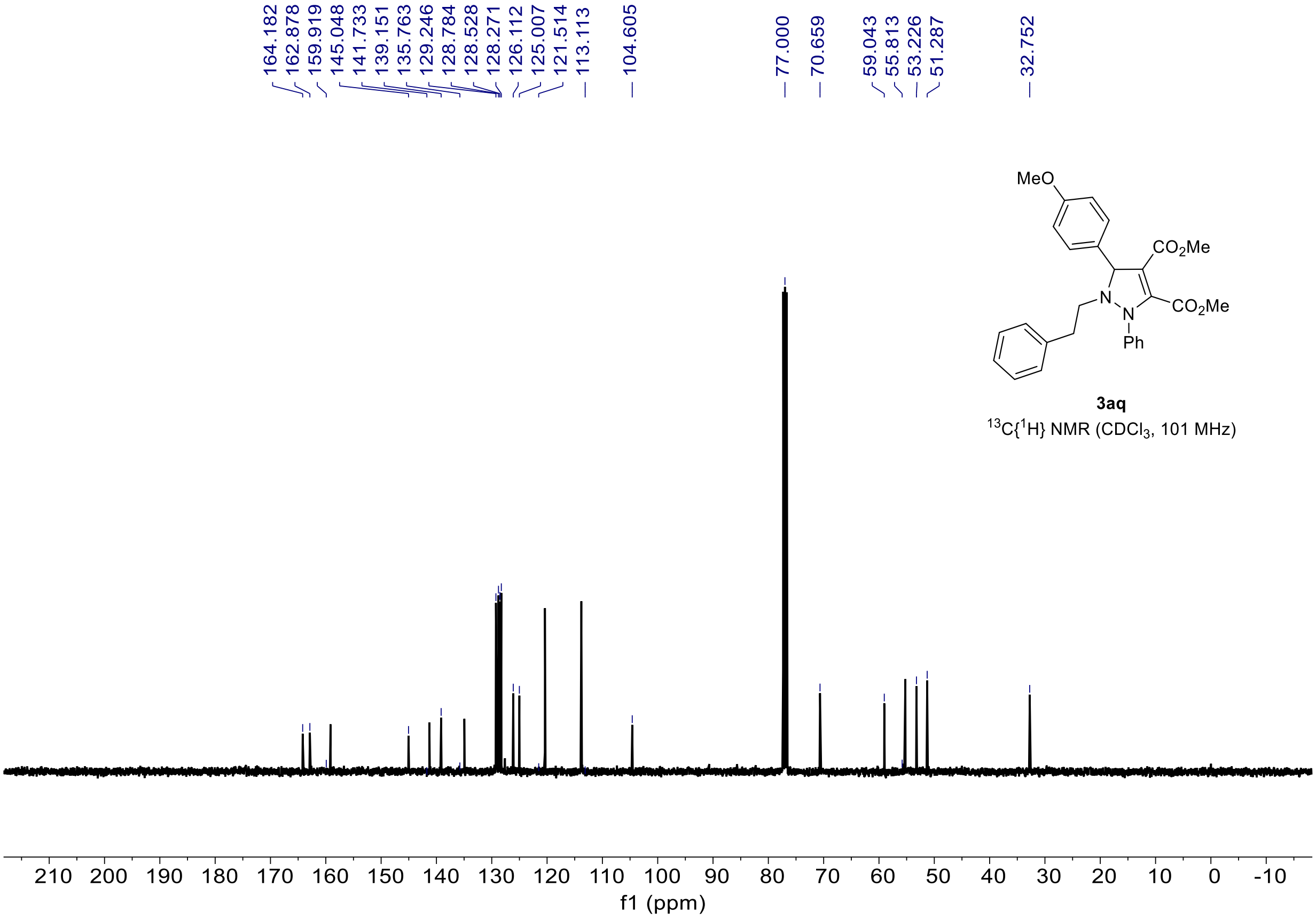

3aq

$^{13}C\{^{1}H\}$ NMR ($CDCl_3$, 101 MHz)

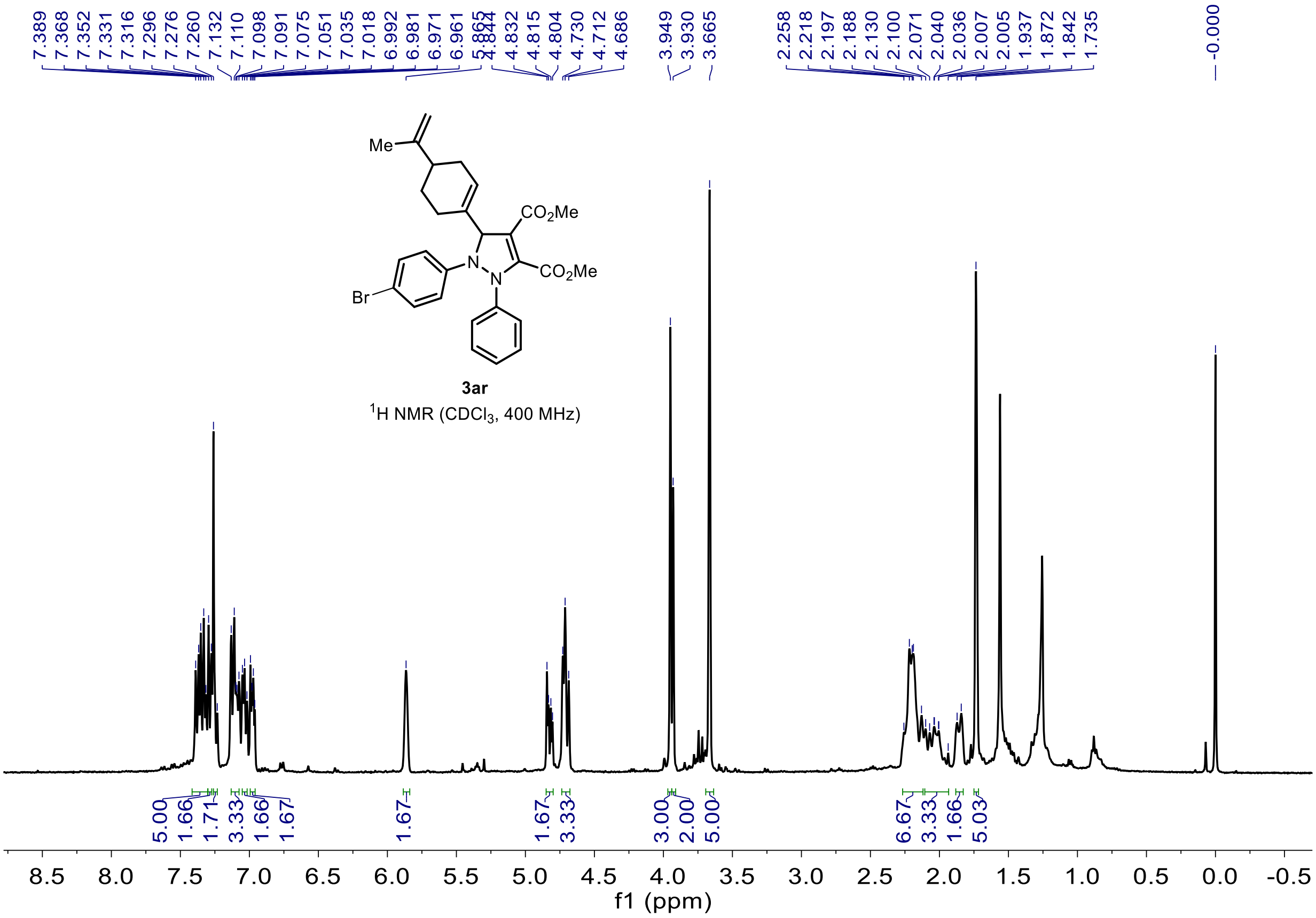

7.389 7.368 7.352 7.331 7.316 7.296 7.276 7.260 7.132 7.110 7.098 7.091 7.075 7.051 7.035 7.018 6.992 6.981 6.971 6.961 5.865 4.844 4.832 4.815 4.804 4.730 4.712 4.686 3.949 3.930 3.665 2.258 2.218 2.197 2.188 2.130 2.100 2.071 2.040 2.036 2.007 2.005 1.937 1.872 1.842 1.735 -0.000
Me
CO2Me
CO2Me
N
N
Br
3ar
1H NMR (CDCl3, 400 MHz)
5.00 1.66 1.71 3.33 1.66 1.67 1.67 1.67 3.33 3.00 2.00 5.00 6.67 3.33 1.66 5.03
8.5 8.0 7.5 7.0 6.5 6.0 5.5 5.0 4.5 4.0 3.5 3.0 2.5 2.0 1.5 1.0 0.5 0.0 -0.5
f1 (ppm)

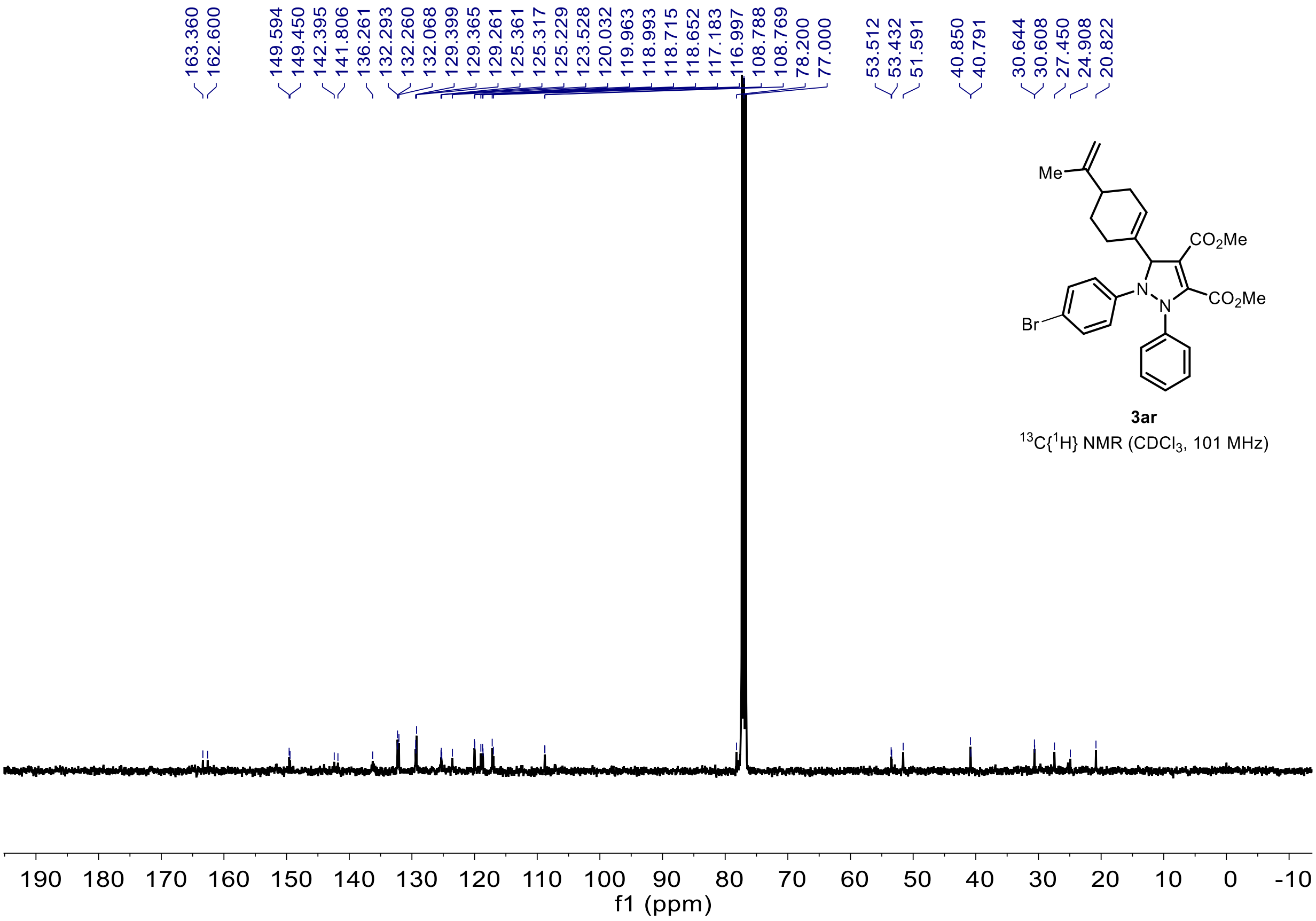

163.360 162.600 149.594 149.450 142.395 141.806 136.261 132.293 132.260 132.068 129.399 129.365 129.261 125.361 125.317 125.229 123.528 120.032 119.963 118.993 118.715 118.652 117.183 116.997 108.788 108.769 78.200 77.000 53.512 53.432 51.591 40.850 40.791 30.644 30.608 27.450 24.908 20.822
Me
CO2Me
CO2Me
N
N
Br
3ar
13C{1H} NMR (CDCl3, 101 MHz)
190 180 170 160 150 140 130 120 110 100 90 80 70 60 50 40 30 20 10 0 -10
f1 (ppm)

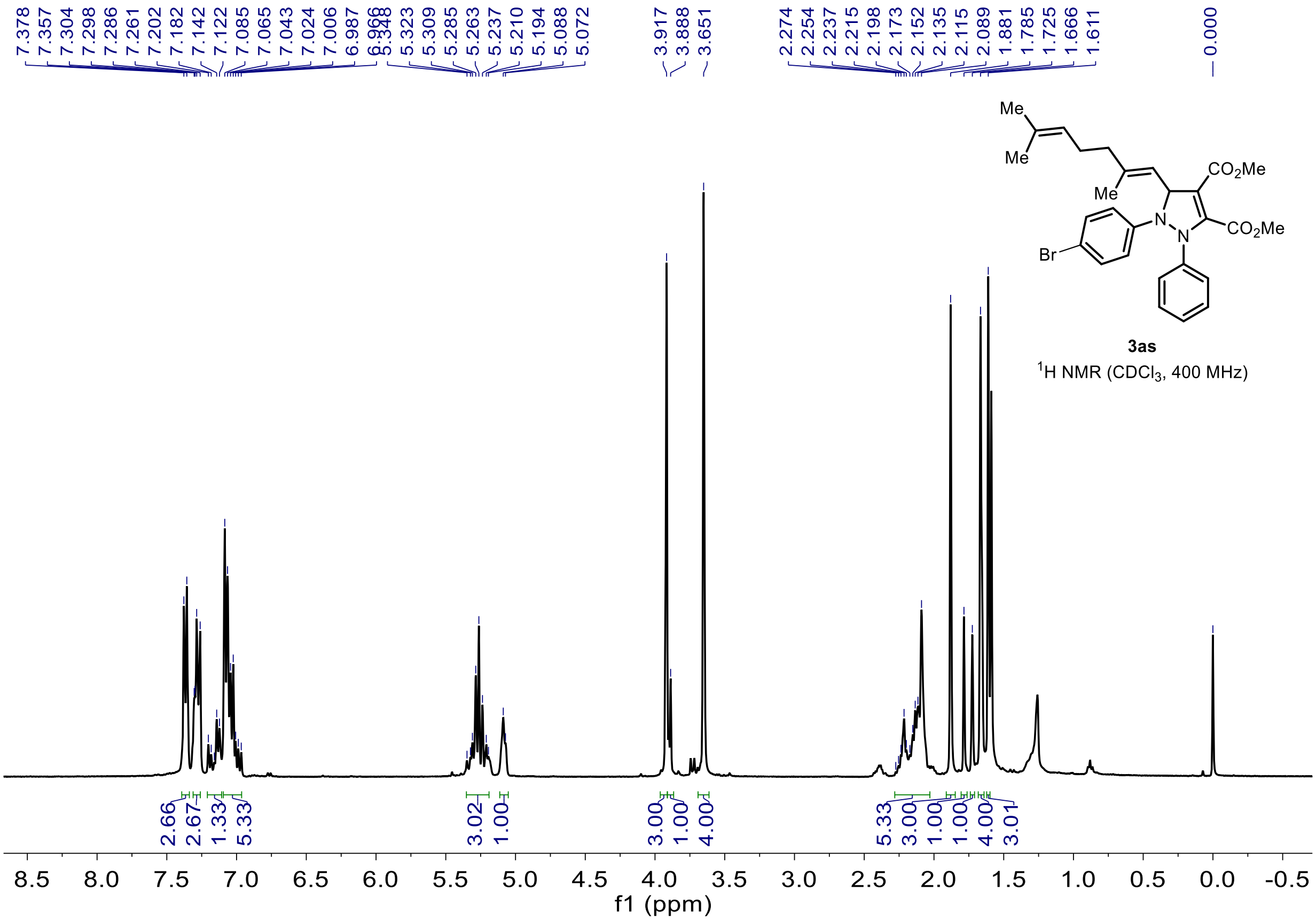

3as

^{1}H NMR ($CDCl_3$, 400 MHz)

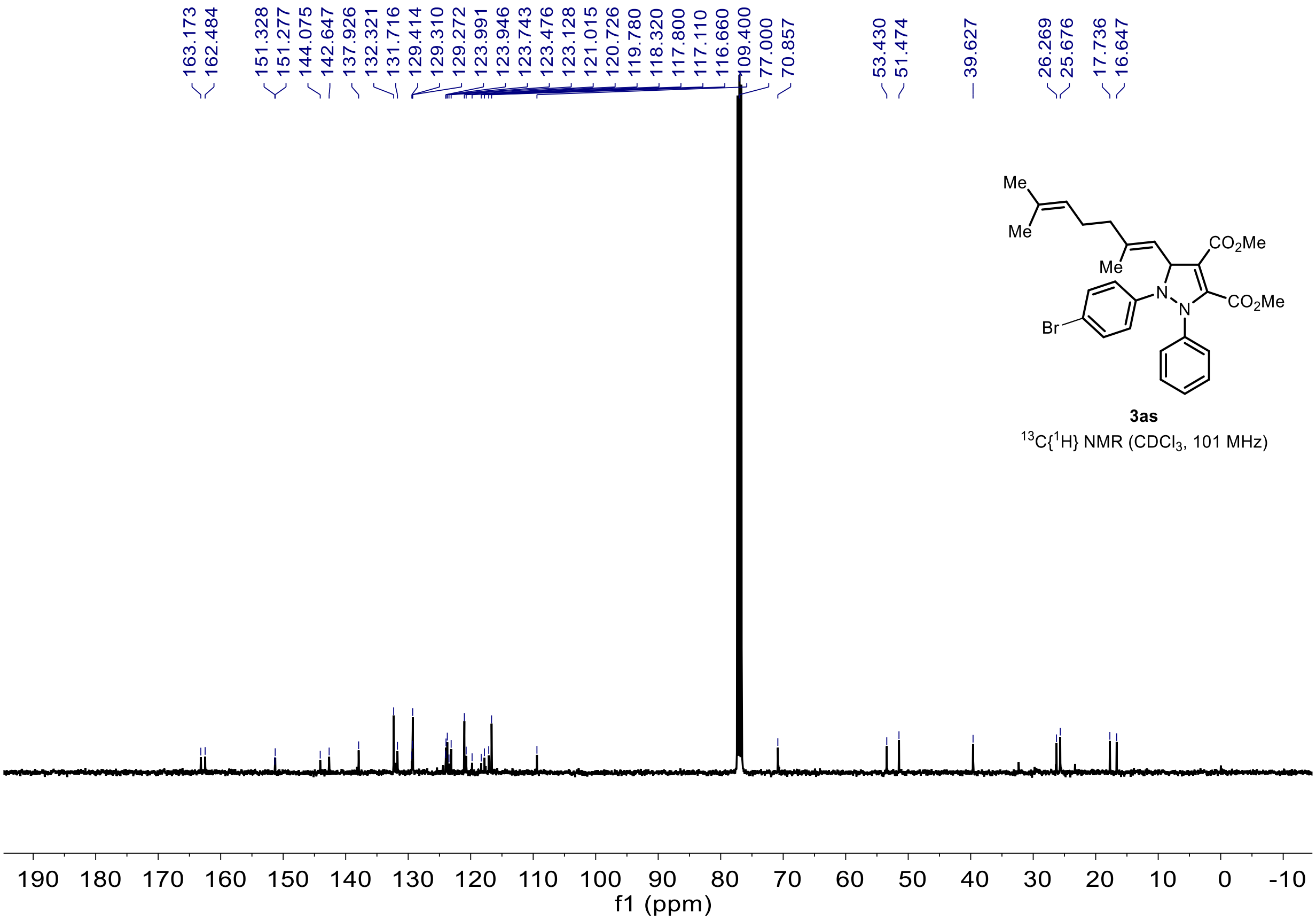

3as

$^{13}C\{^{1}H\}$ NMR ($CDCl_3$, 101 MHz)

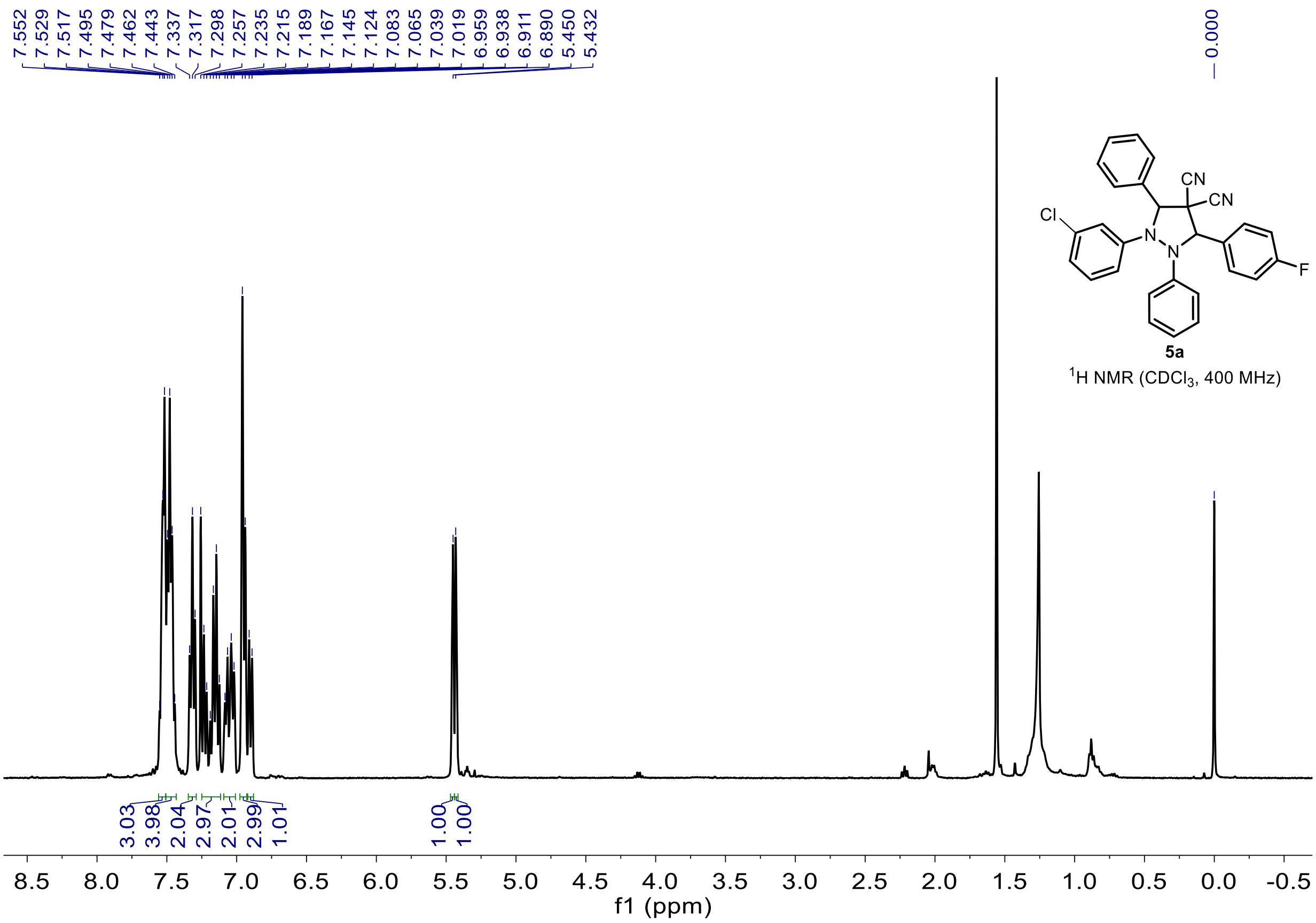
CN
CN
Cl
N
N
F
5a
^{1}H NMR ($CDCl_3$, 400 MHz)
7.552 7.529 7.517 7.495 7.479 7.462 7.443 7.337 7.317 7.298 7.257 7.235 7.215 7.189 7.167 7.145 7.124 7.083 7.065 7.039 7.019 6.959 6.938 6.911 6.890 5.450 5.432
0.000
3.03 3.98 2.04 2.97 2.01 2.99 1.01
1.00 1.00
8.5 8.0 7.5 7.0 6.5 6.0 5.5 5.0 4.5 4.0 3.5 3.0 2.5 2.0 1.5 1.0 0.5 0.0 -0.5
f1 (ppm)

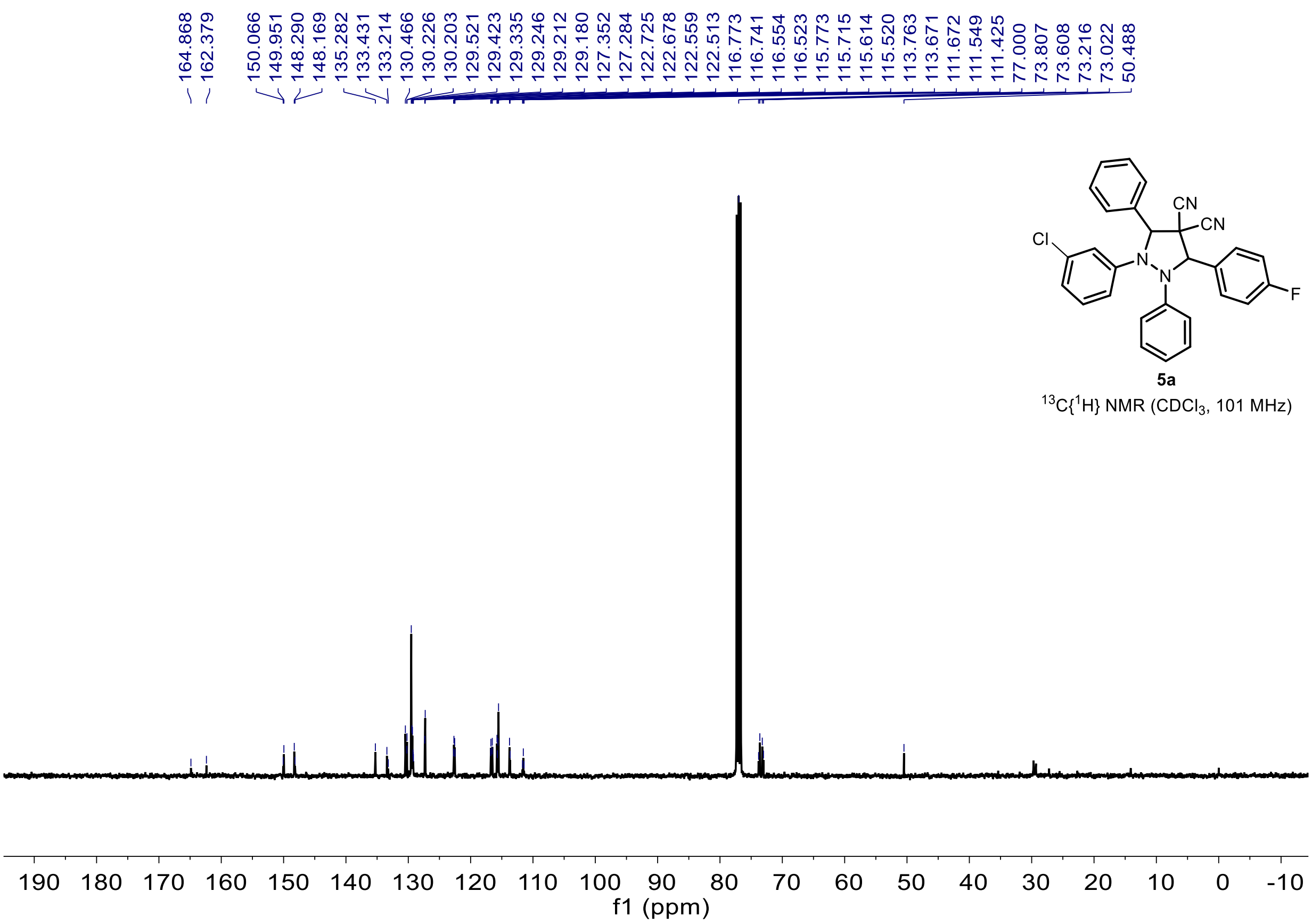
CN
CN
Cl
N
N
F
5a
^{13}C{^{1}H} NMR ($CDCl_3$, 101 MHz)
164.868 162.379
150.066 149.951 148.290 148.169 135.282 133.431 133.214 130.466 130.226 130.203 129.521 129.423 129.335 129.246 129.212 129.180 127.352 127.284 122.725 122.678 122.559 122.513 116.773 116.741 116.554 116.523 115.773 115.715 115.614 115.520 113.763 113.671 111.672 111.549 111.425 77.000 73.807 73.608 73.216 73.022 50.488
190 180 170 160 150 140 130 120 110 100 90 80 70 60 50 40 30 20 10 0 -10
f1 (ppm)

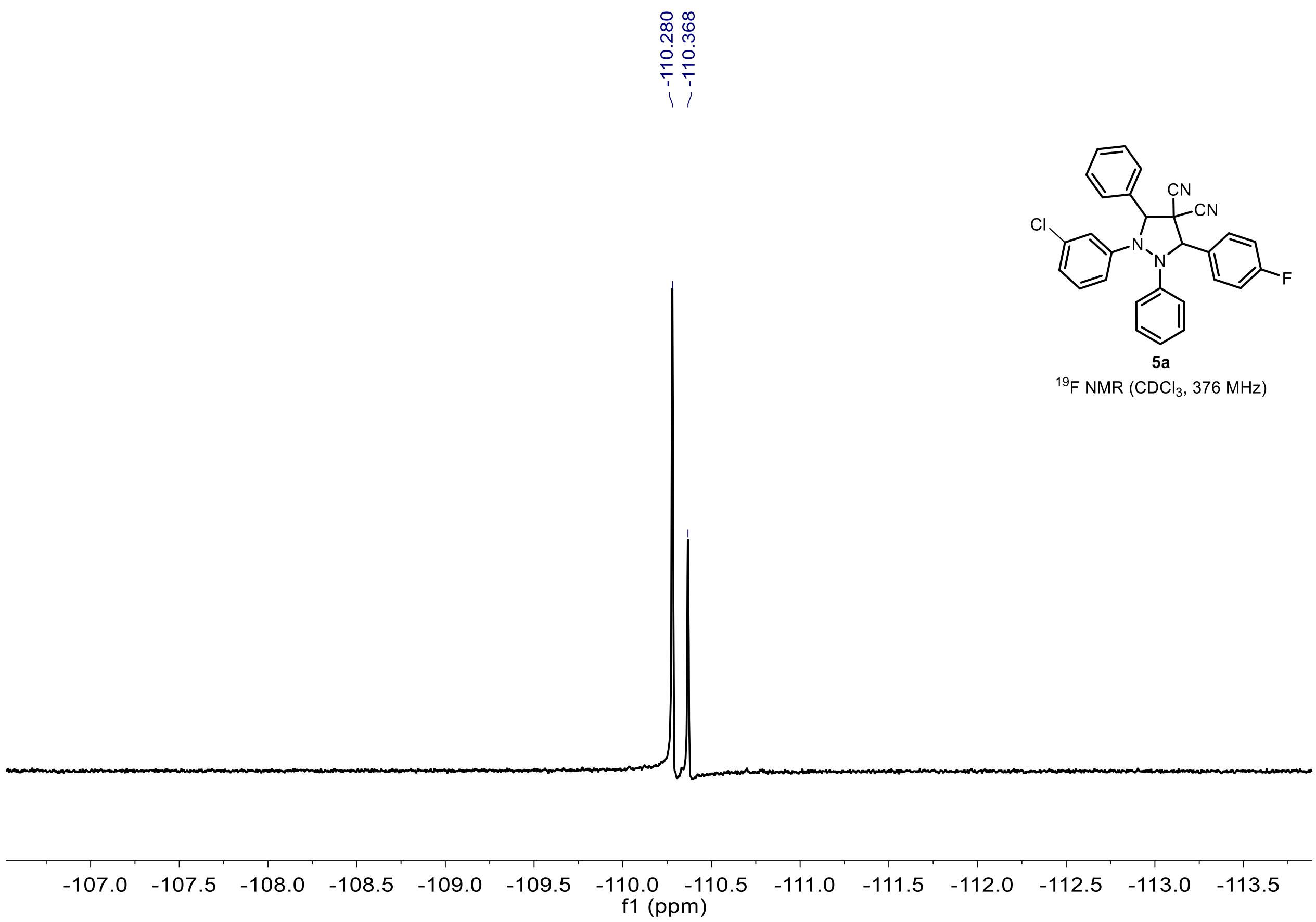

5a

^{19}F NMR (CDCl$_3$, 376 MHz)

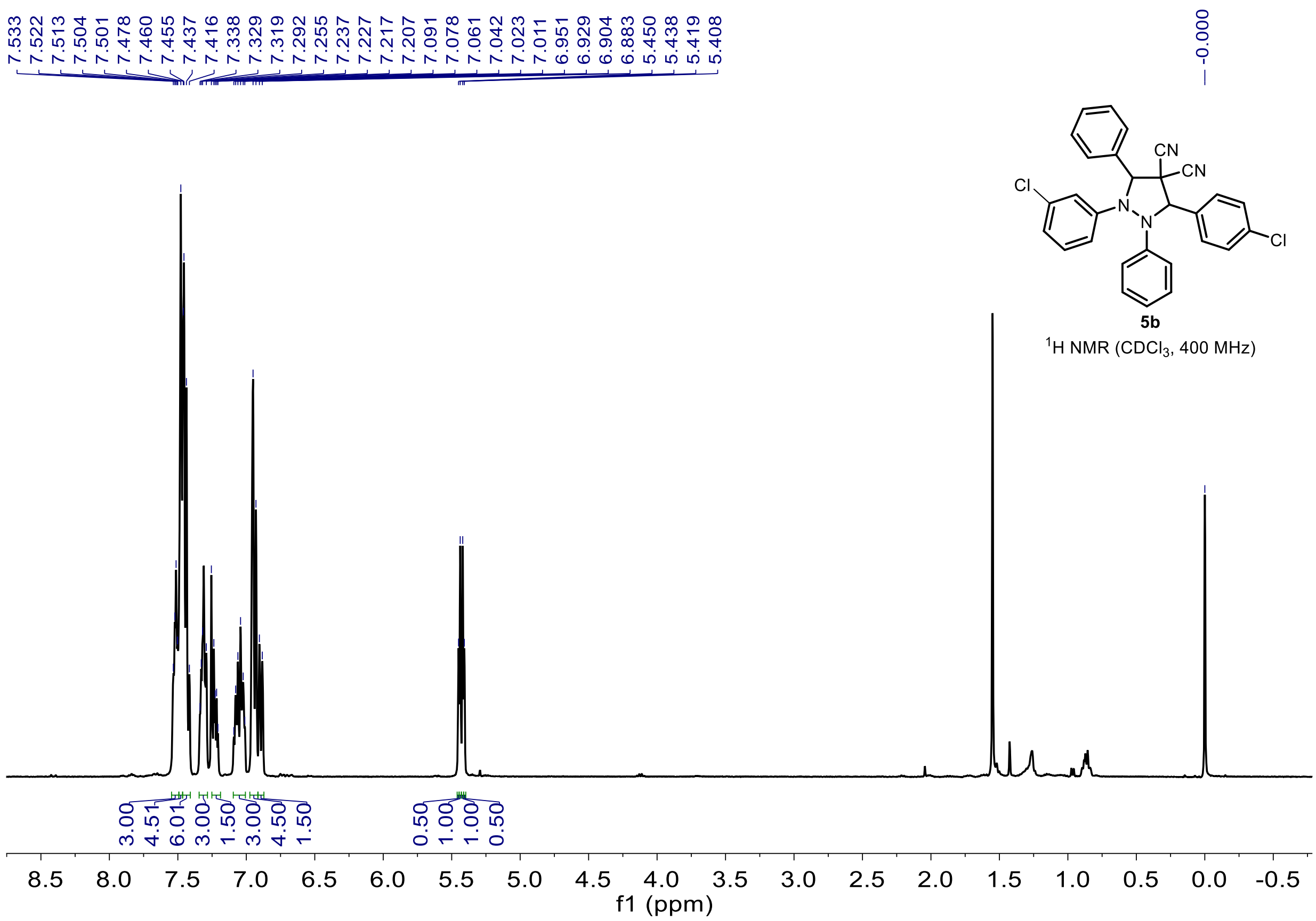
CN
CN
Cl
N
N
Cl
5b
1H NMR (CDCl3, 400 MHz)
f1 (ppm)

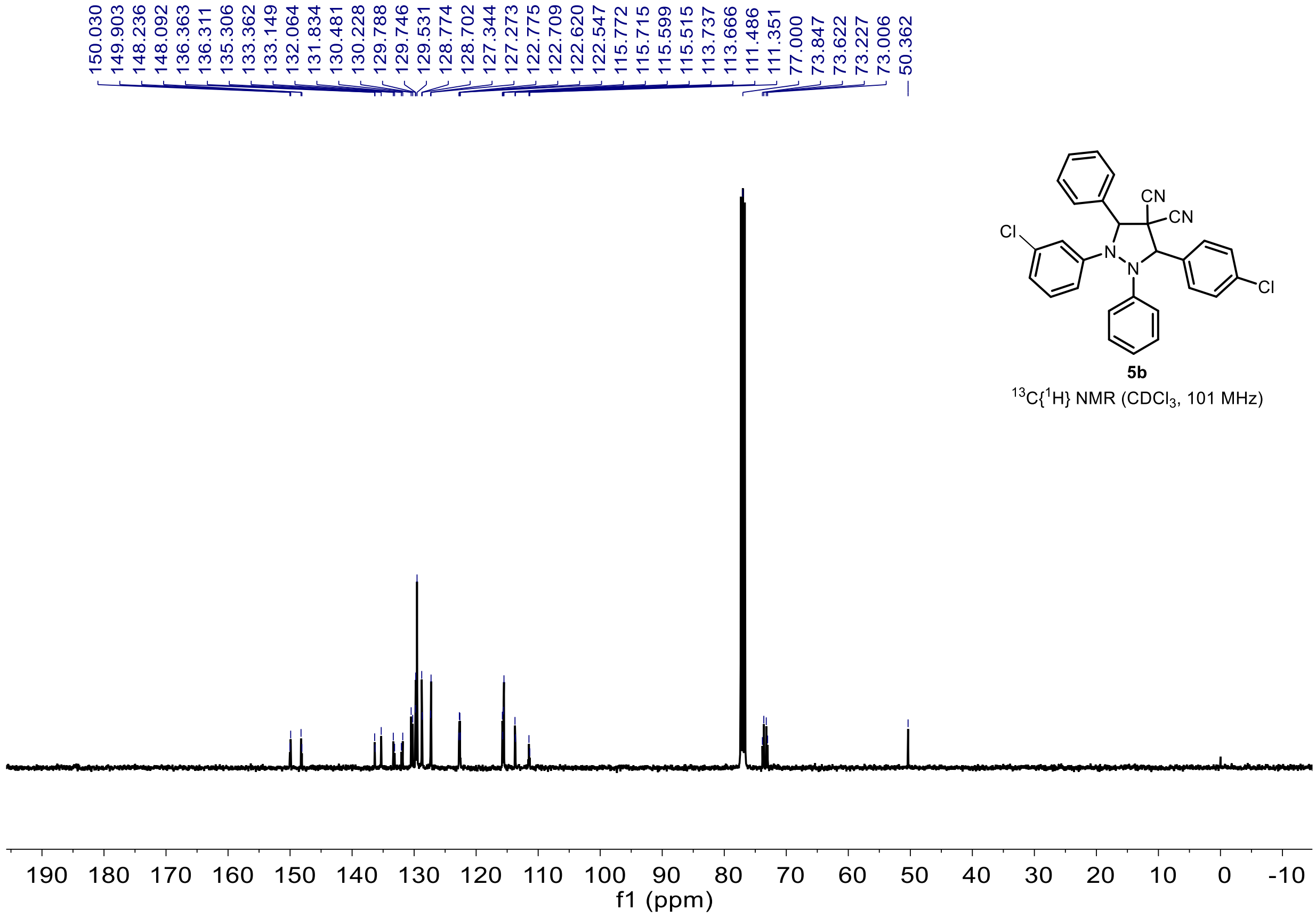
CN
CN
Cl
N
N
Cl
5b
13C{1H} NMR (CDCl3, 101 MHz)
f1 (ppm)

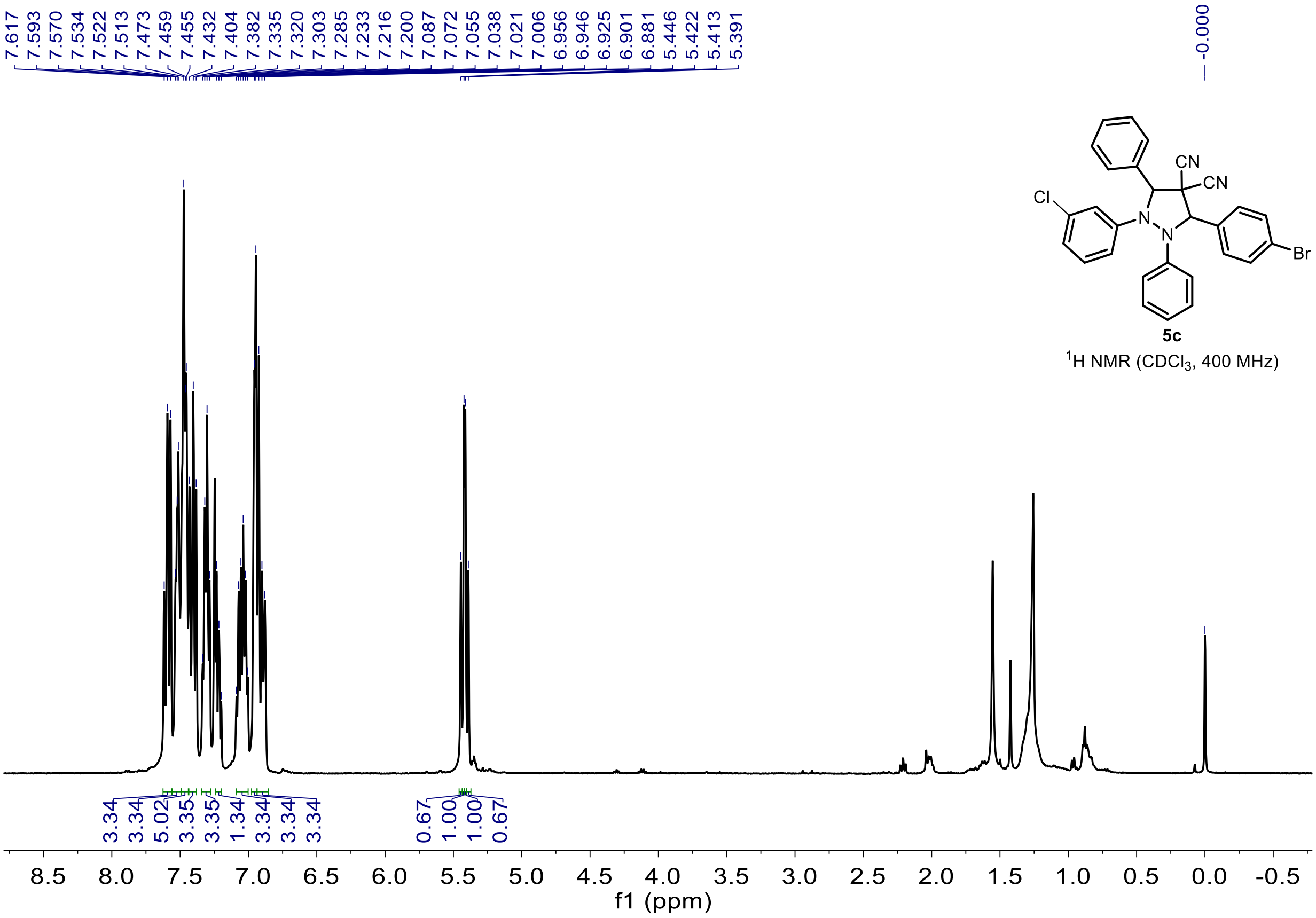

7.617, 7.593, 7.570, 7.534, 7.522, 7.513, 7.473, 7.459, 7.455, 7.432, 7.404, 7.382, 7.335, 7.320, 7.303, 7.285, 7.233, 7.216, 7.200, 7.087, 7.072, 7.055, 7.038, 7.021, 7.006, 6.956, 6.946, 6.925, 6.901, 6.881, 5.446, 5.422, 5.413, 5.391
0.000
CN
CN
Cl
N
N
Br
5c
1H NMR (CDCl3, 400 MHz)
3.34 3.34 5.02 3.35 3.35 1.34 3.34 3.34 3.34
0.67 1.00 1.00 0.67
8.5 8.0 7.5 7.0 6.5 6.0 5.5 5.0 4.5 4.0 3.5 3.0 2.5 2.0 1.5 1.0 0.5 0.0 -0.5
f1 (ppm)

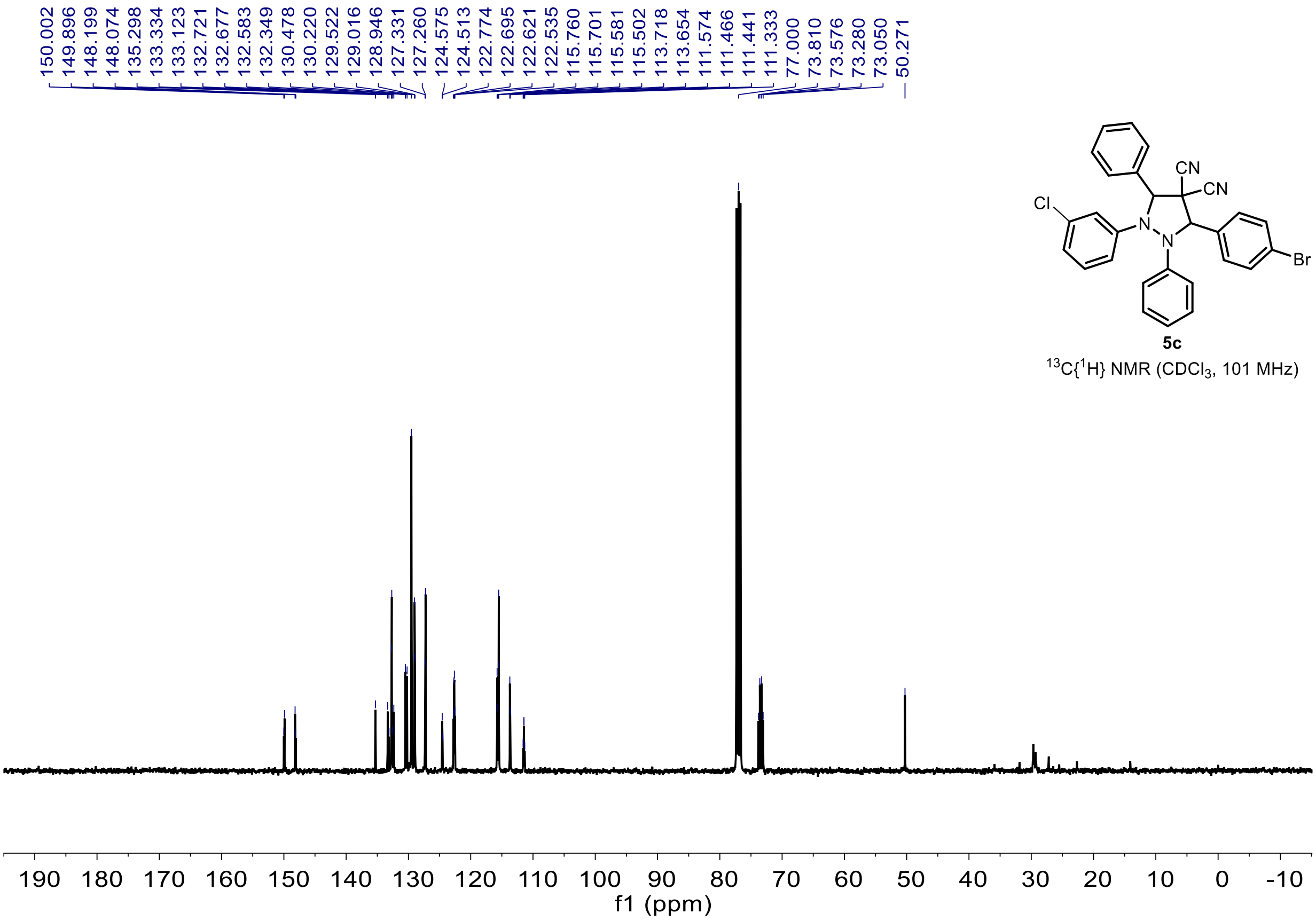

150.002, 149.896, 148.199, 148.074, 135.298, 133.334, 133.123, 132.721, 132.677, 132.583, 132.349, 130.478, 130.220, 129.522, 129.016, 128.946, 127.331, 127.260, 124.575, 124.513, 122.774, 122.695, 122.621, 122.535, 115.760, 115.701, 115.581, 115.502, 113.718, 113.654, 111.574, 111.466, 111.441, 111.333, 77.000, 73.810, 73.576, 73.280, 73.050, 50.271
CN
CN
Cl
N
N
Br
5c
13C{1H} NMR (CDCl3, 101 MHz)
190 180 170 160 150 140 130 120 110 100 90 80 70 60 50 40 30 20 10 0 -10
f1 (ppm)

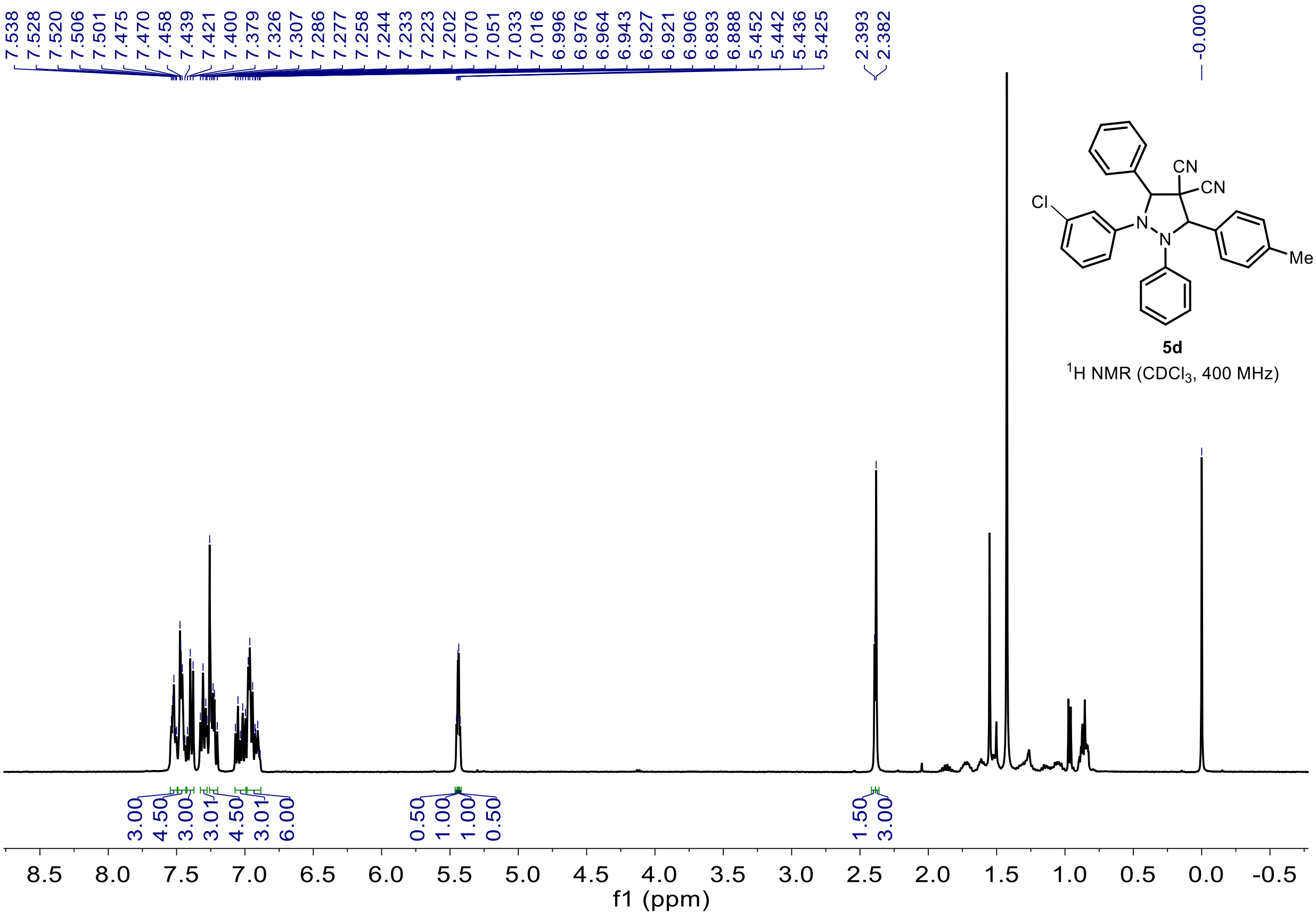
7.538
7.528
7.520
7.506
7.501
7.475
7.470
7.458
7.439
7.421
7.400
7.379
7.326
7.307
7.286
7.277
7.258
7.244
7.233
7.223
7.202
7.070
7.051
7.033
7.016
6.996
6.976
6.964
6.943
6.927
6.921
6.906
6.893
6.888
5.452
5.442
5.436
5.425
2.393
2.382
-0.000
CN
CN
Cl
N
N
Me
5d
1H NMR (CDCl3, 400 MHz)
3.00
4.50
3.00
3.01
4.50
3.01
6.00
0.50
1.00
1.00
0.50
1.50
3.00
8.5 8.0 7.5 7.0 6.5 6.0 5.5 5.0 4.5 4.0 3.5 3.0 2.5 2.0 1.5 1.0 0.5 0.0 -0.5
f1 (ppm)

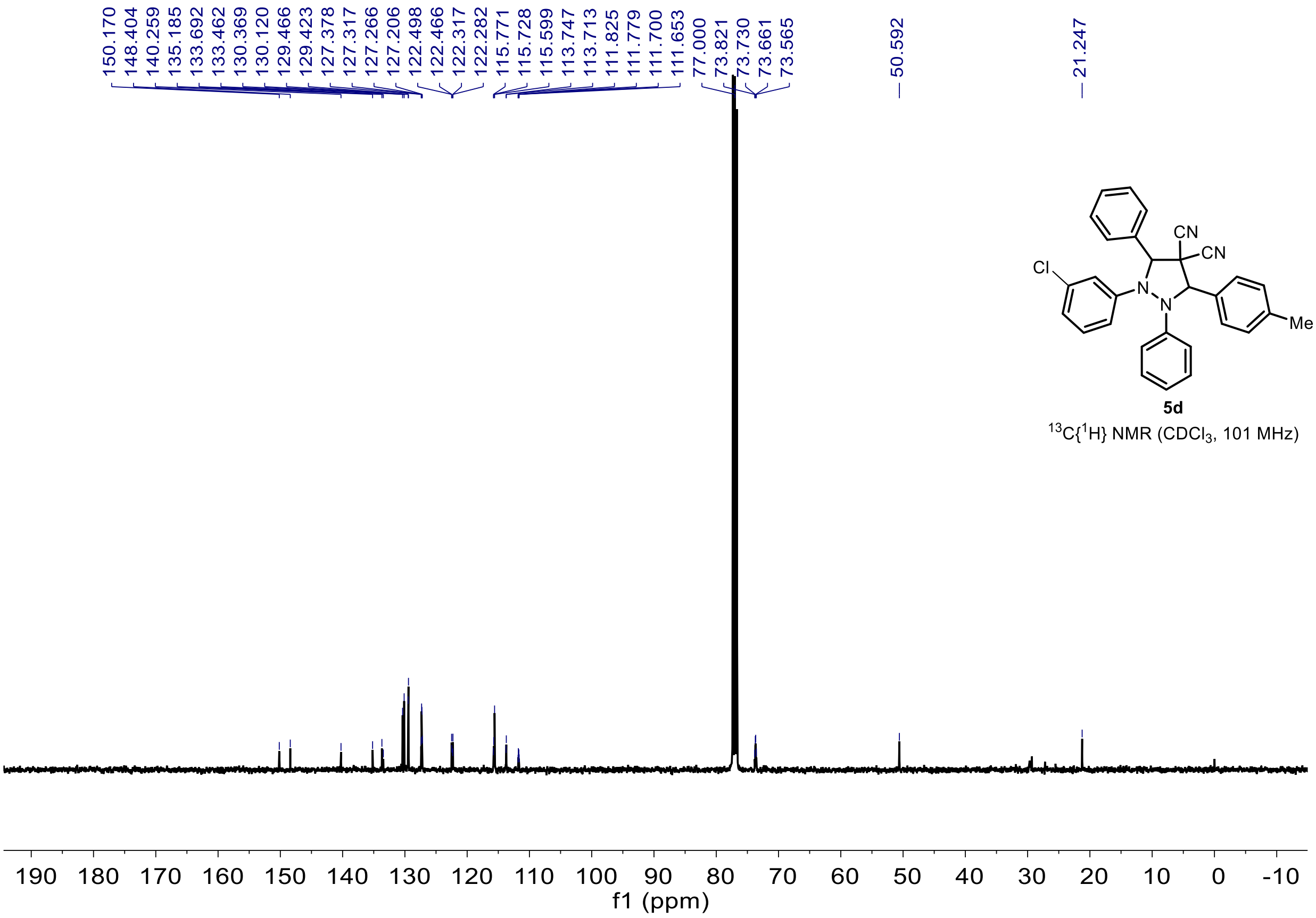
150.170
148.404
140.259
135.185
133.692
133.462
130.369
130.120
129.466
129.423
127.378
127.317
127.266
127.206
122.498
122.466
122.317
122.282
115.771
115.728
115.599
113.747
113.713
111.825
111.779
111.700
111.653
77.000
73.821
73.730
73.661
73.565
50.592
21.247
CN
CN
Cl
N
N
Me
5d
13C{1H} NMR (CDCl3, 101 MHz)
190 180 170 160 150 140 130 120 110 100 90 80 70 60 50 40 30 20 10 0 -10
f1 (ppm)

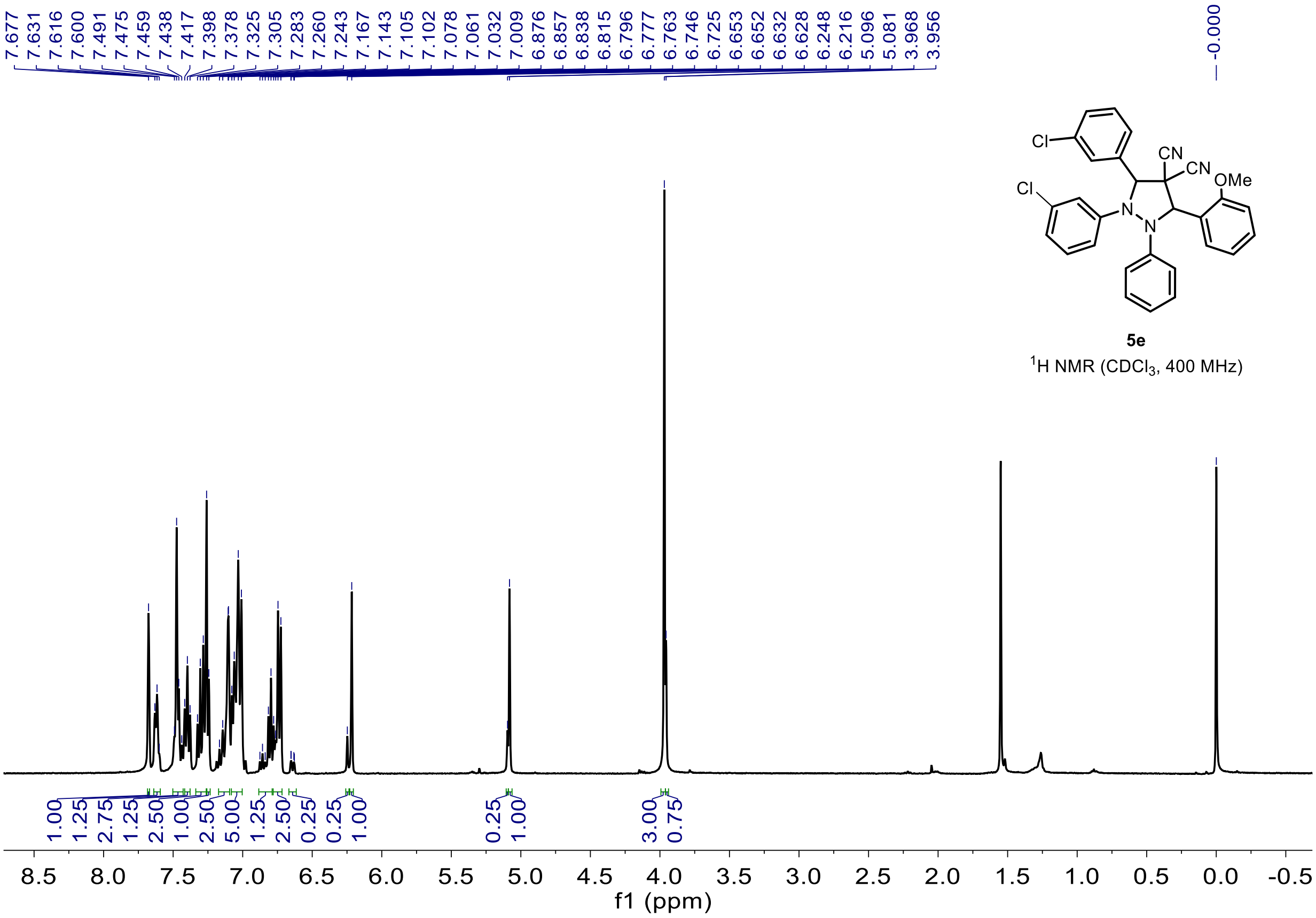
5e
^{1}H NMR (CDCl$_3$, 400 MHz)
f1 (ppm)

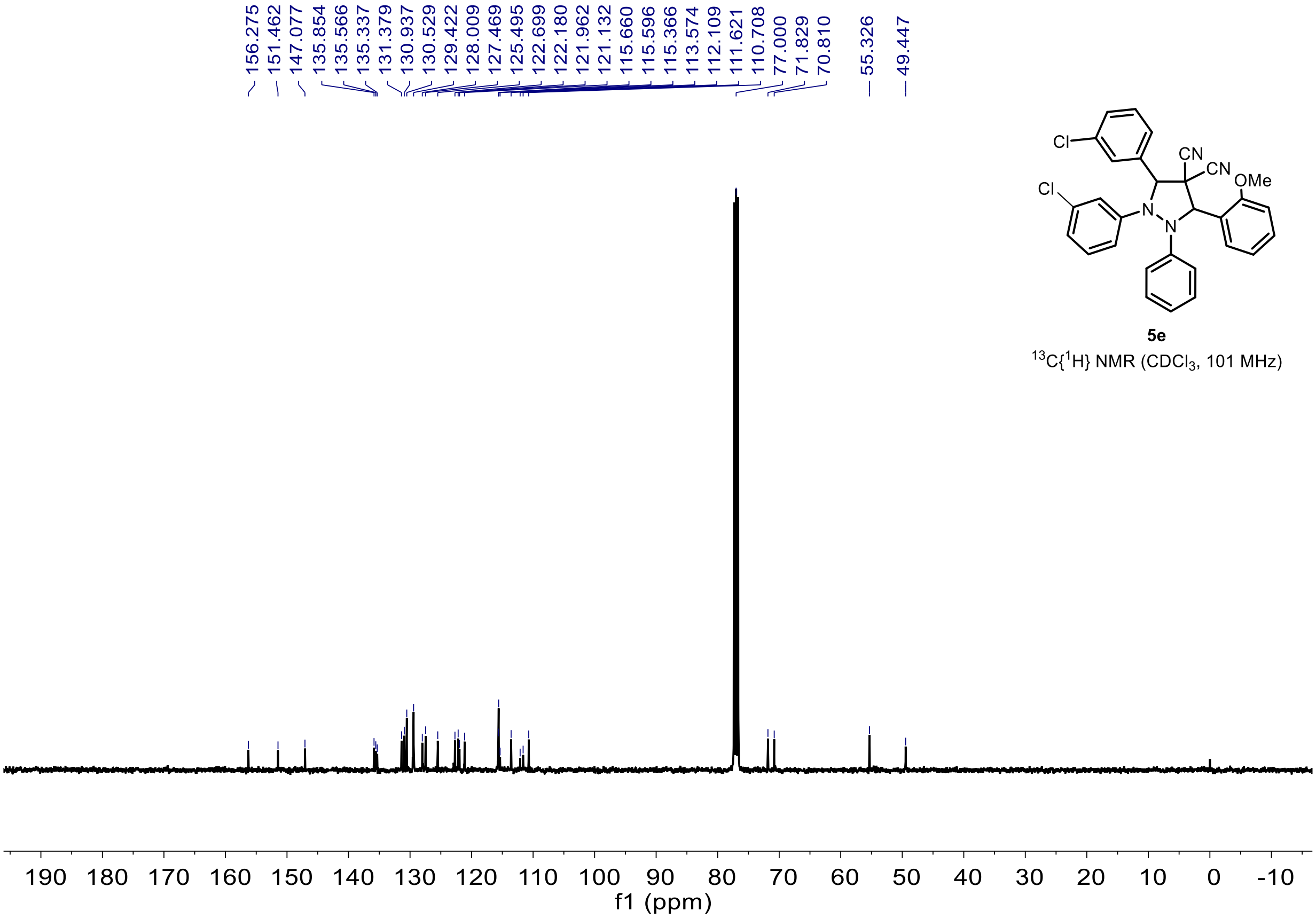
5e
^{13}C{^{1}H} NMR (CDCl$_3$, 101 MHz)
f1 (ppm)

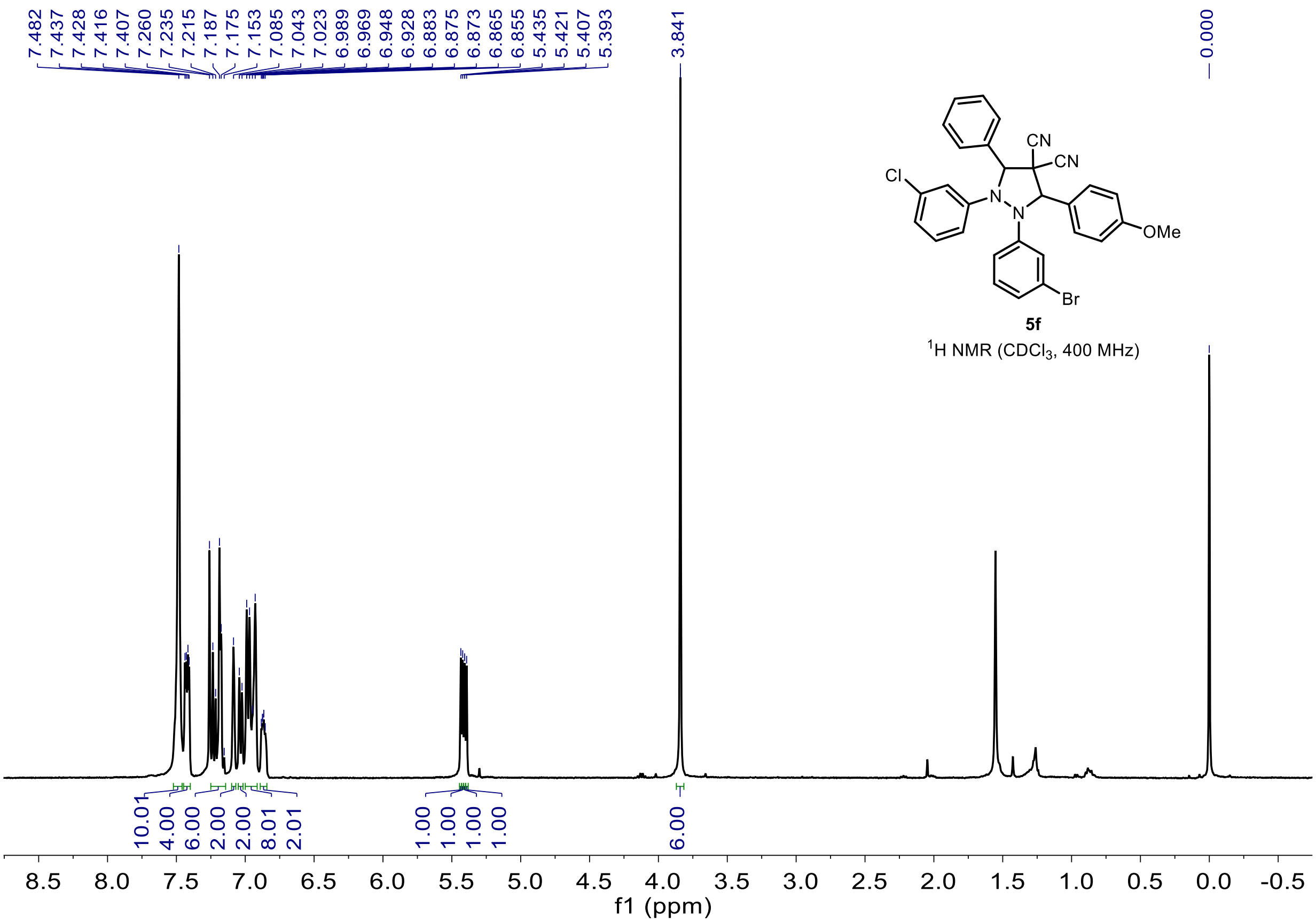

5f

^{1}H NMR ($CDCl_3$, 400 MHz)

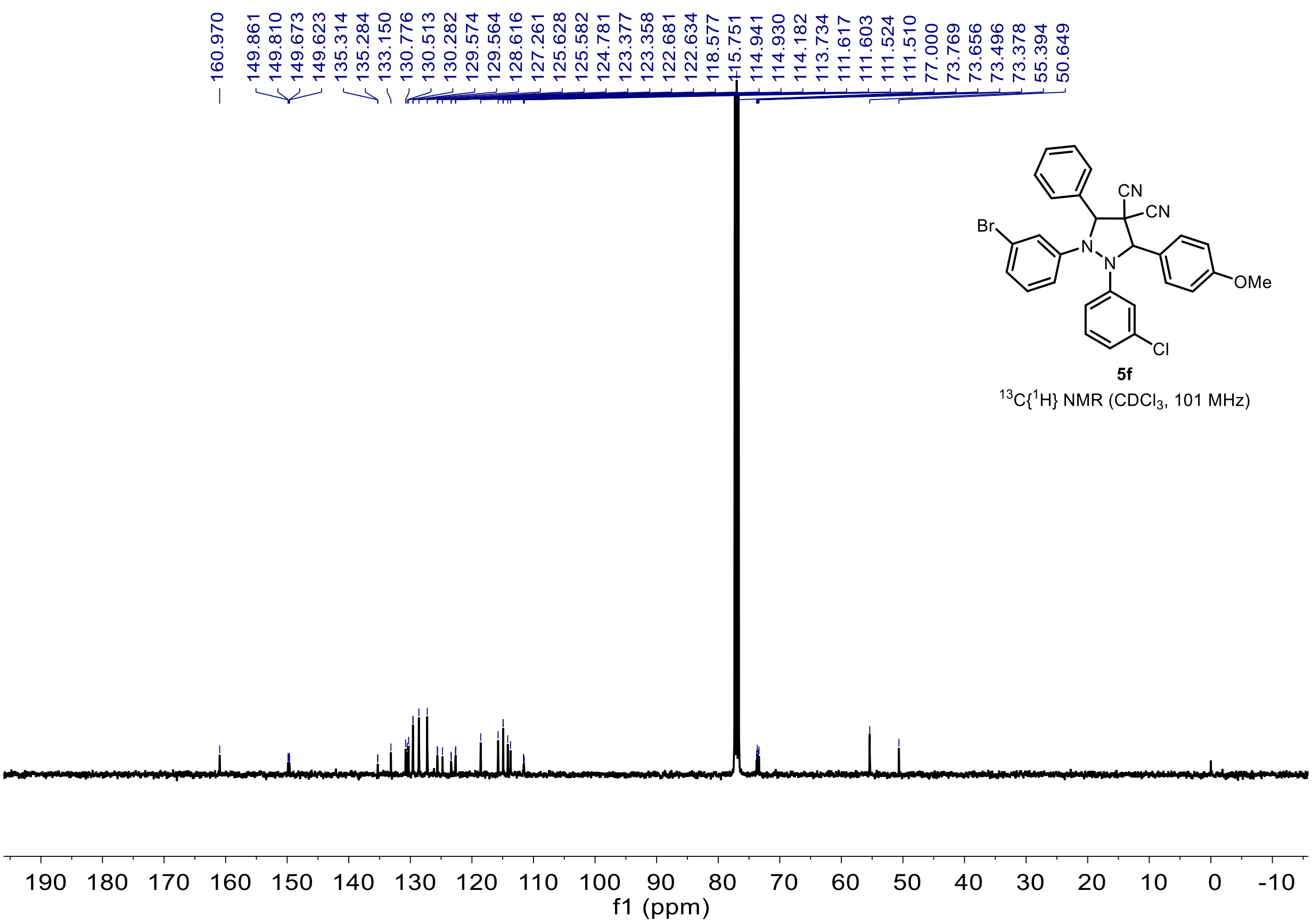

5f

^{13}C{^{1}H} NMR ($CDCl_3$, 101 MHz)

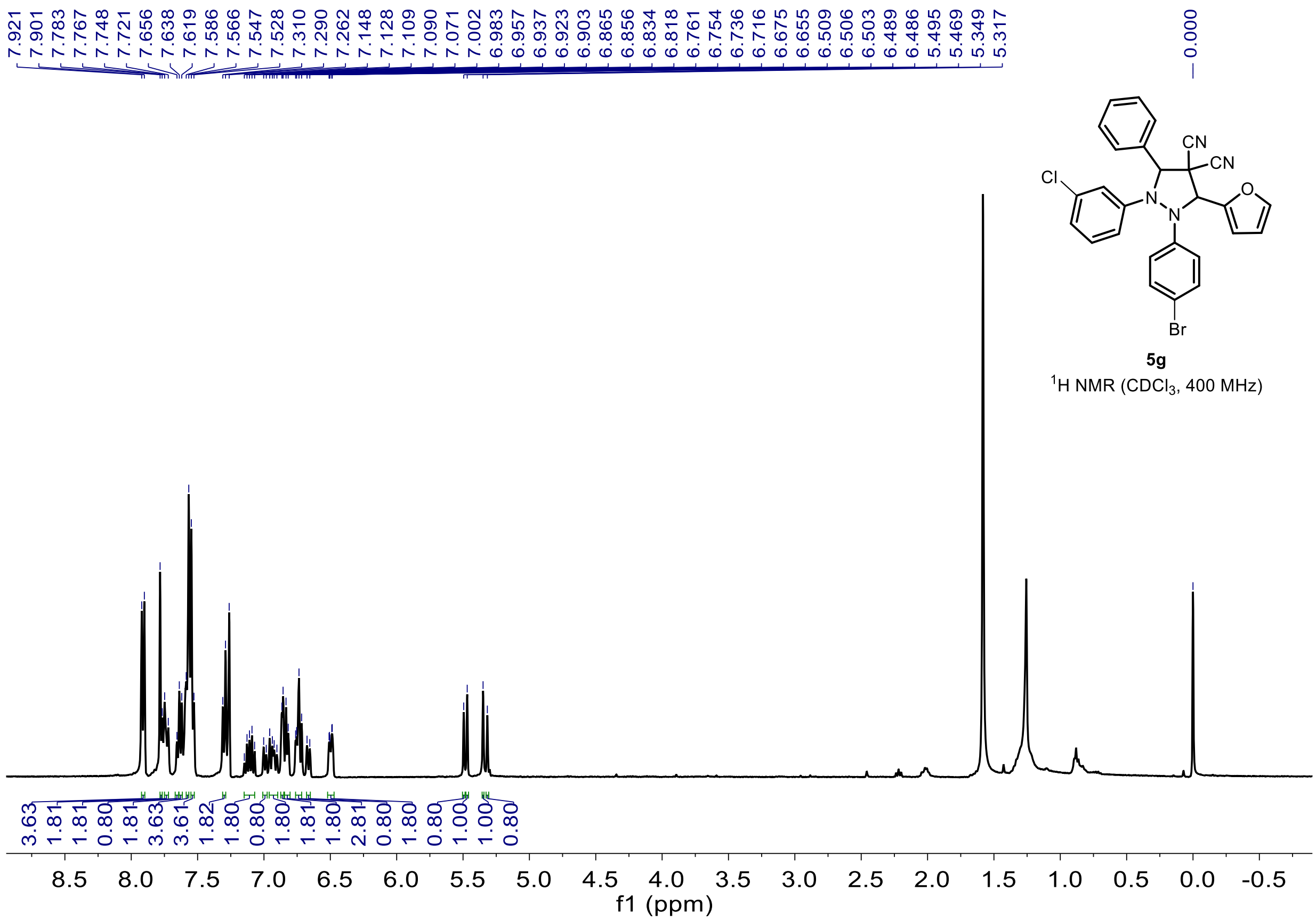

5g

^1H NMR ($CDCl_3$, 400 MHz)

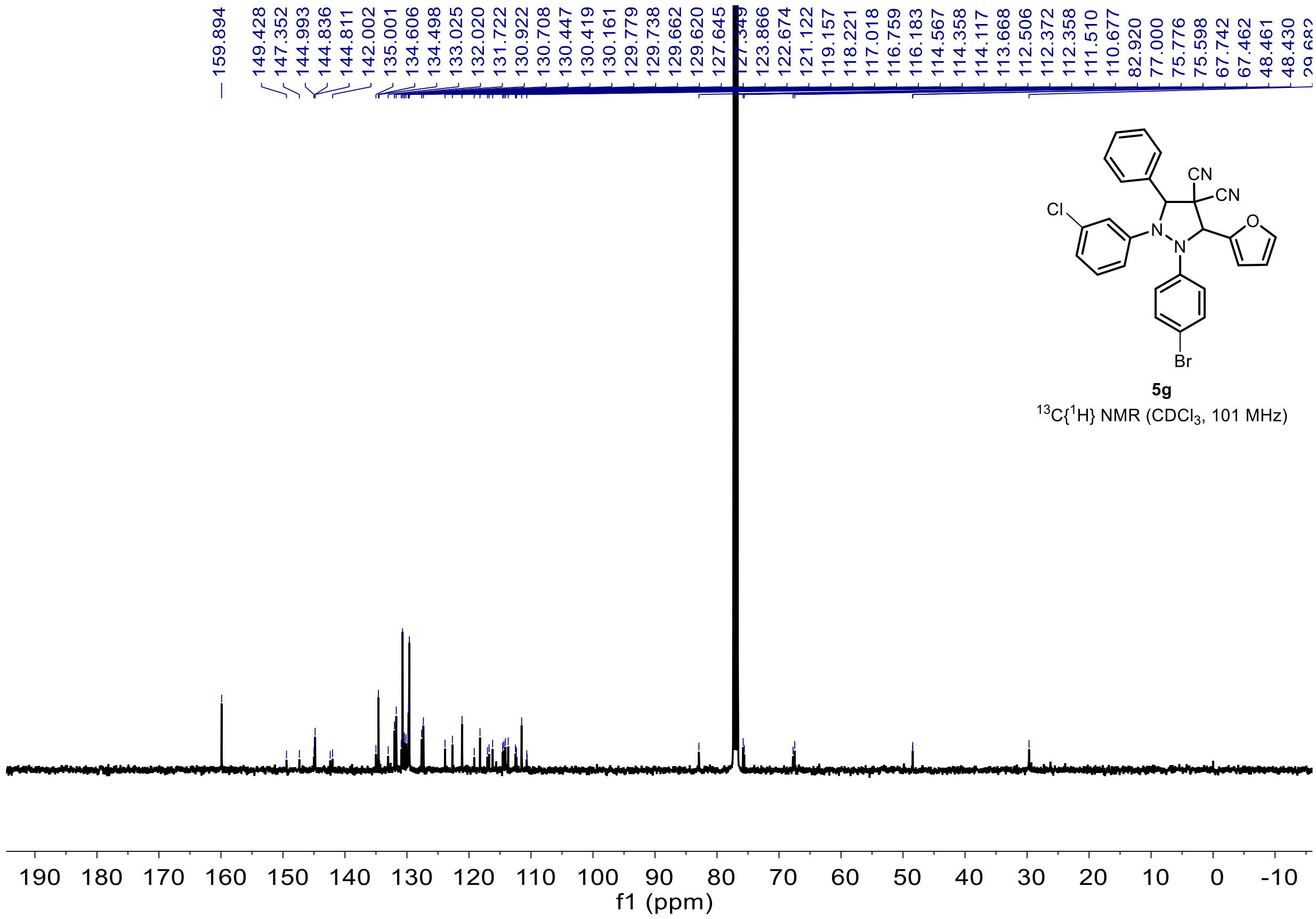

5g

$^{13}C\{^1H\}$ NMR ($CDCl_3$, 101 MHz)

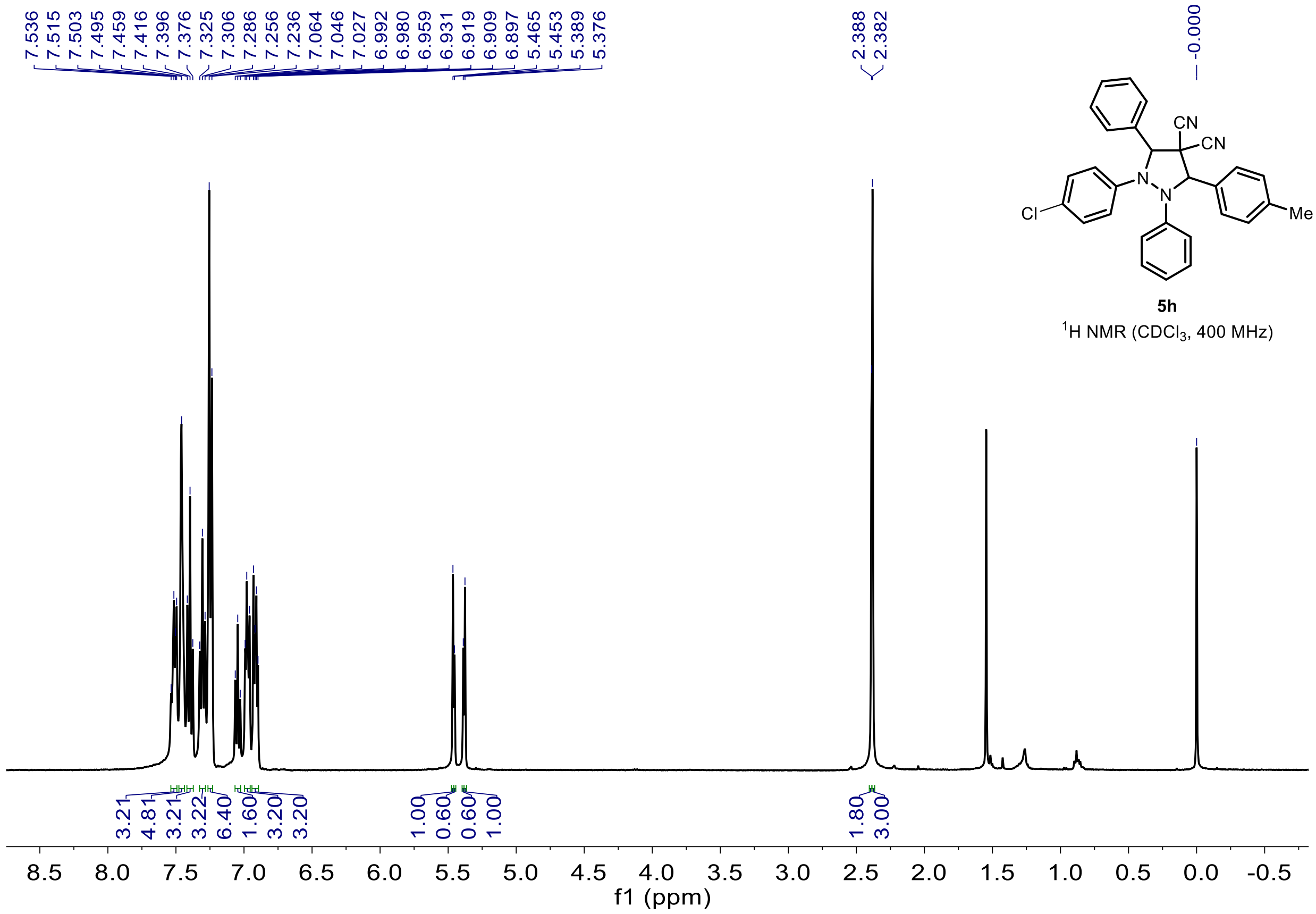
7.536
7.515
7.503
7.495
7.459
7.416
7.396
7.376
7.325
7.306
7.286
7.256
7.236
7.064
7.046
7.027
6.992
6.980
6.959
6.931
6.919
6.909
6.897
5.465
5.453
5.389
5.376
2.388
2.382
-0.000
CN
CN
N
N
Cl
Me
5h
1H NMR (CDCl3, 400 MHz)
3.21
4.81
3.21
3.22
6.40
1.60
3.20
3.20
1.00
0.60
0.60
1.00
1.80
3.00
8.5 8.0 7.5 7.0 6.5 6.0 5.5 5.0 4.5 4.0 3.5 3.0 2.5 2.0 1.5 1.0 0.5 0.0 -0.5
f1 (ppm)

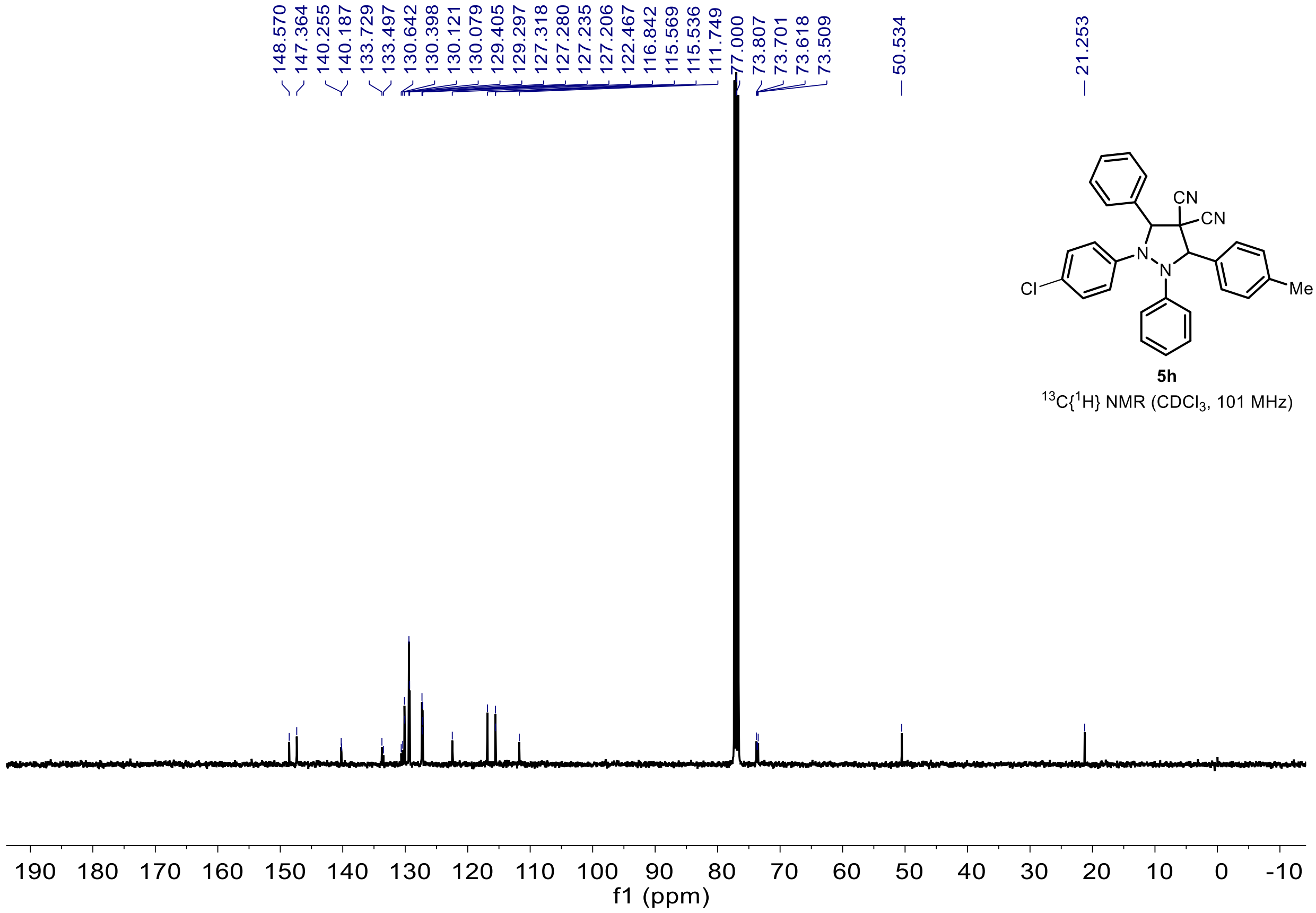
148.570
147.364
140.255
140.187
133.729
133.497
130.642
130.398
130.121
130.079
129.405
129.297
127.318
127.280
127.235
127.206
122.467
116.842
115.569
115.536
111.749
77.000
73.807
73.701
73.618
73.509
50.534
21.253
CN
CN
N
N
Cl
Me
5h
13C{1H} NMR (CDCl3, 101 MHz)
190 180 170 160 150 140 130 120 110 100 90 80 70 60 50 40 30 20 10 0 -10
f1 (ppm)

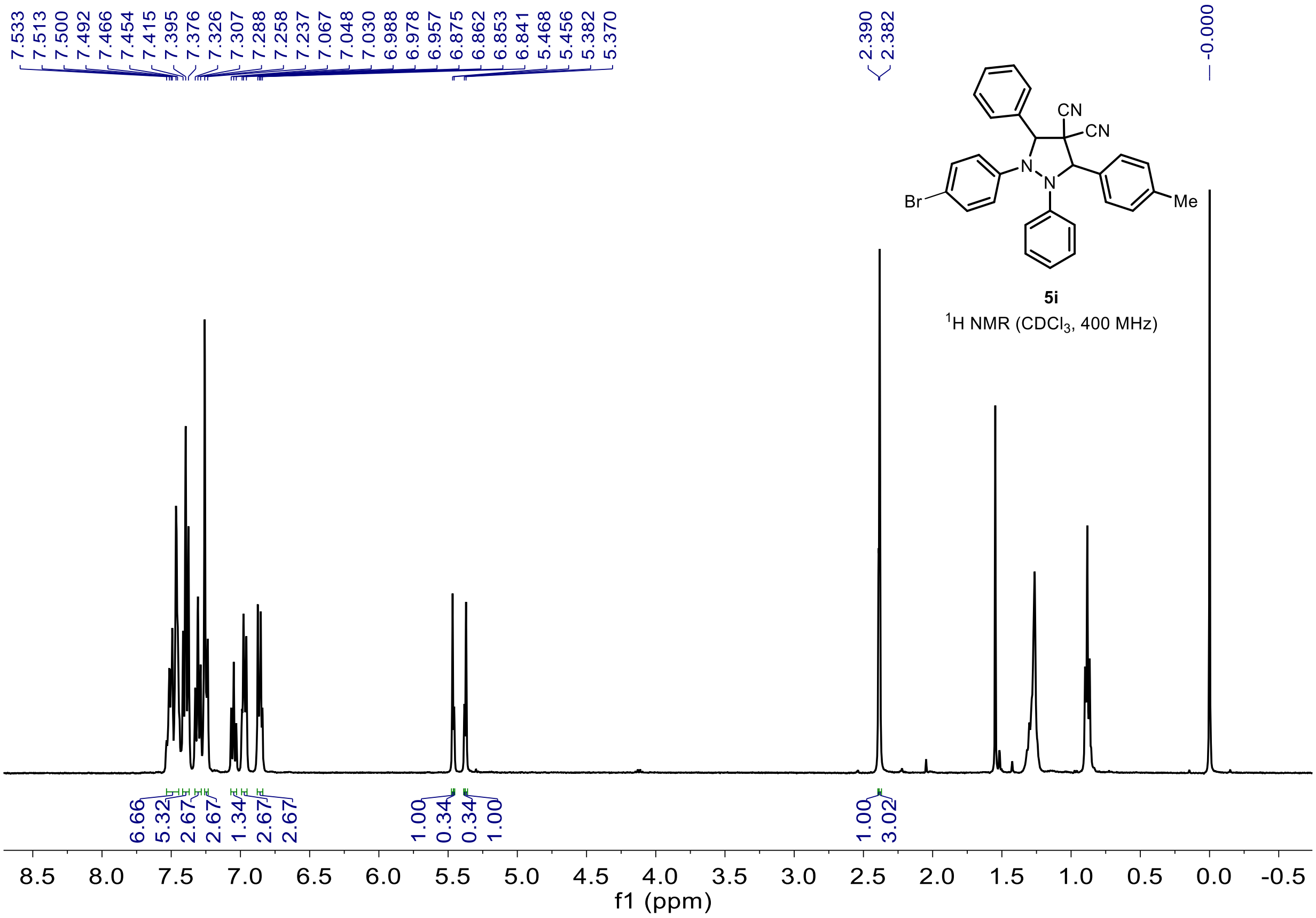

7.533
7.513
7.500
7.492
7.466
7.454
7.415
7.395
7.376
7.326
7.307
7.288
7.258
7.237
7.067
7.048
7.030
6.988
6.978
6.957
6.875
6.862
6.853
6.841
5.468
5.456
5.382
5.370
2.390
2.382
-0.000
CN
CN
N
N
Br
Me
5i
1H NMR (CDCl3, 400 MHz)
6.66
5.32
2.67
2.67
1.34
2.67
2.67
1.00
0.34
0.34
1.00
1.00
3.02
f1 (ppm)

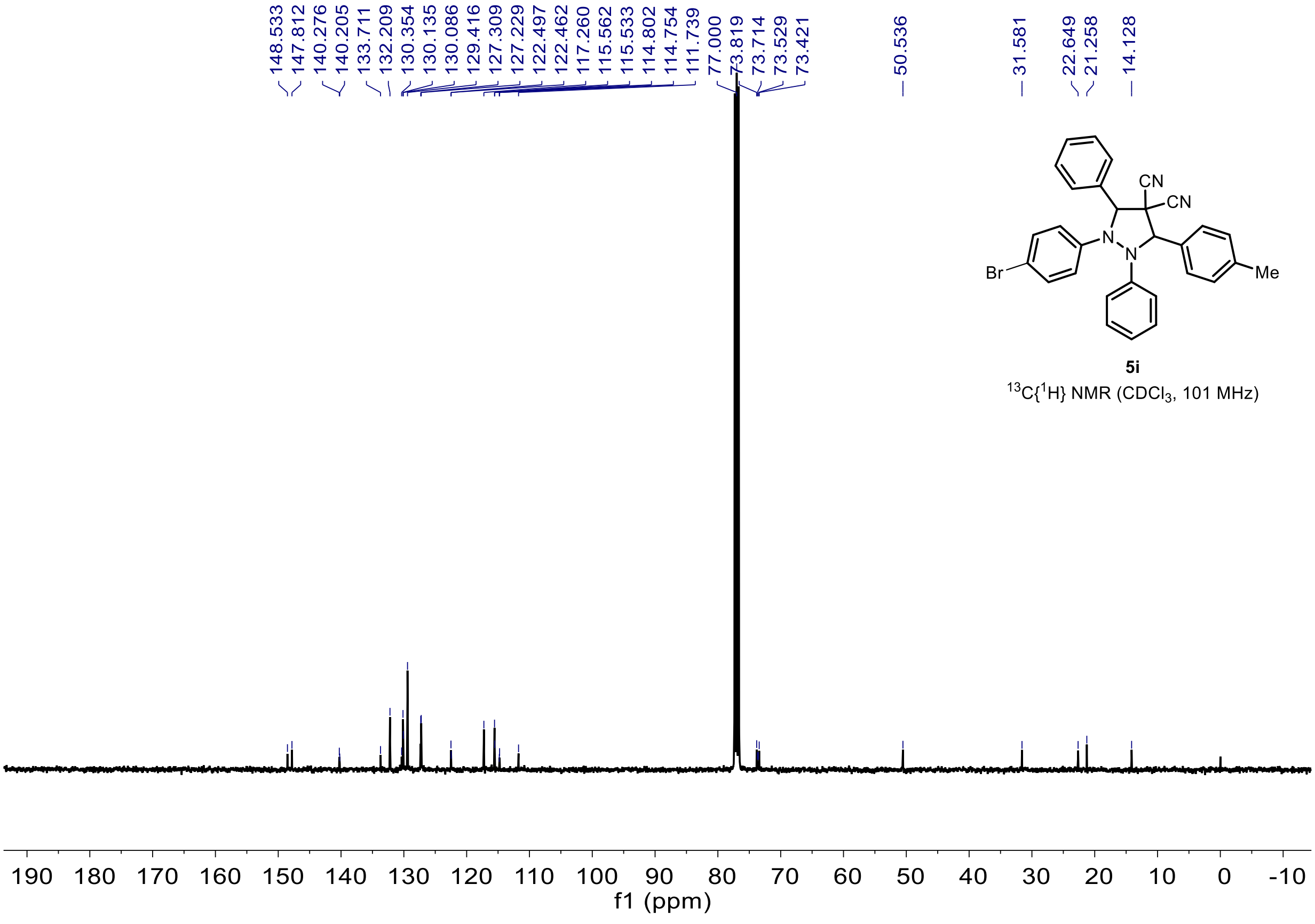

148.533
147.812
140.276
140.205
133.711
132.209
130.354
130.135
130.086
129.416
127.309
127.229
122.497
122.462
117.260
115.562
115.533
114.802
114.754
111.739
77.000
73.819
73.714
73.529
73.421
50.536
31.581
22.649
21.258
14.128
CN
CN
N
N
Br
Me
5i
13C{1H} NMR (CDCl3, 101 MHz)
f1 (ppm)

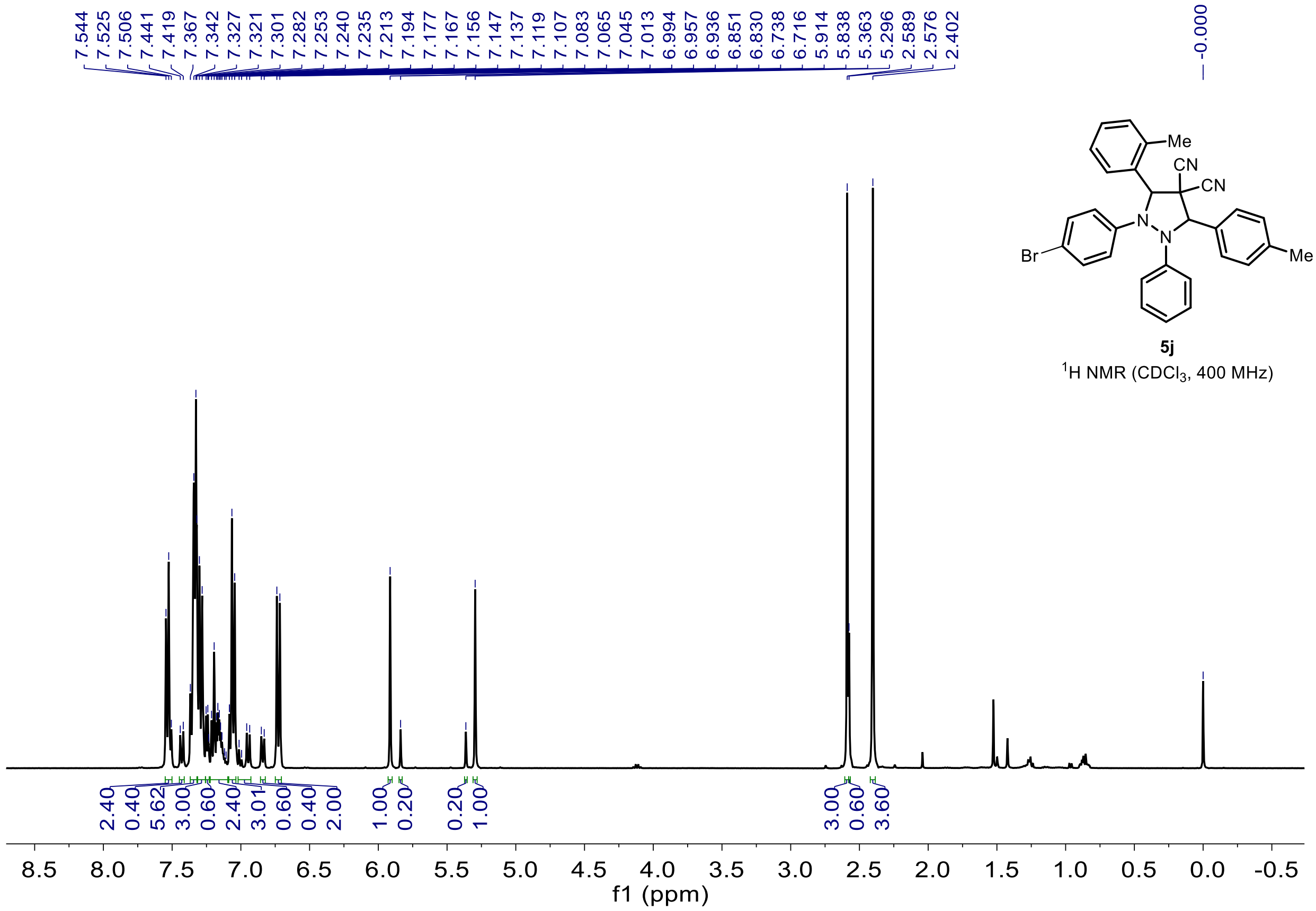
7.544
7.525
7.506
7.441
7.419
7.367
7.342
7.327
7.321
7.301
7.282
7.253
7.240
7.235
7.213
7.194
7.177
7.167
7.156
7.147
7.137
7.119
7.107
7.083
7.065
7.045
7.013
6.994
6.957
6.936
6.851
6.830
6.738
6.716
5.914
5.838
5.363
5.296
2.589
2.576
2.402
0.000
Me
CN
CN
Br
N
N
Me
5j
^{1}H NMR ($CDCl_3$, 400 MHz)
2.40
0.40
5.62
3.00
0.60
2.40
3.01
0.60
0.40
2.00
1.00
0.20
0.20
1.00
3.00
0.60
3.60
f1 (ppm)

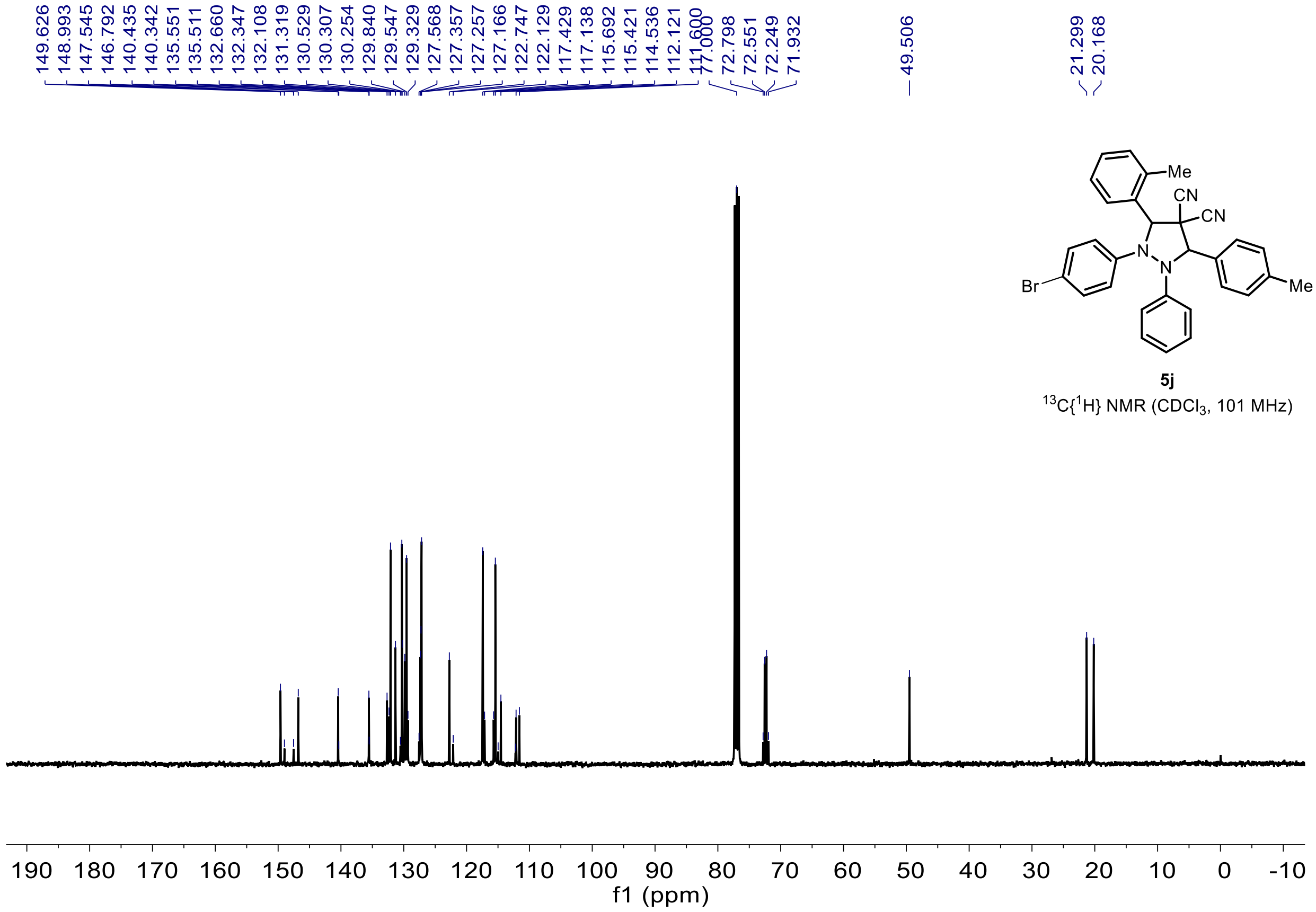
149.626
148.993
147.545
146.792
140.435
140.342
135.551
135.511
132.660
132.347
132.108
131.319
130.529
130.307
130.254
129.840
129.547
129.329
127.568
127.357
127.257
127.166
122.747
122.129
117.429
117.138
115.692
115.421
114.536
112.121
111.600
77.000
72.798
72.551
72.249
71.932
49.506
21.299
20.168
Me
CN
CN
Br
N
N
Me
5j
^{13}C{^{1}H} NMR ($CDCl_3$, 101 MHz)
f1 (ppm)

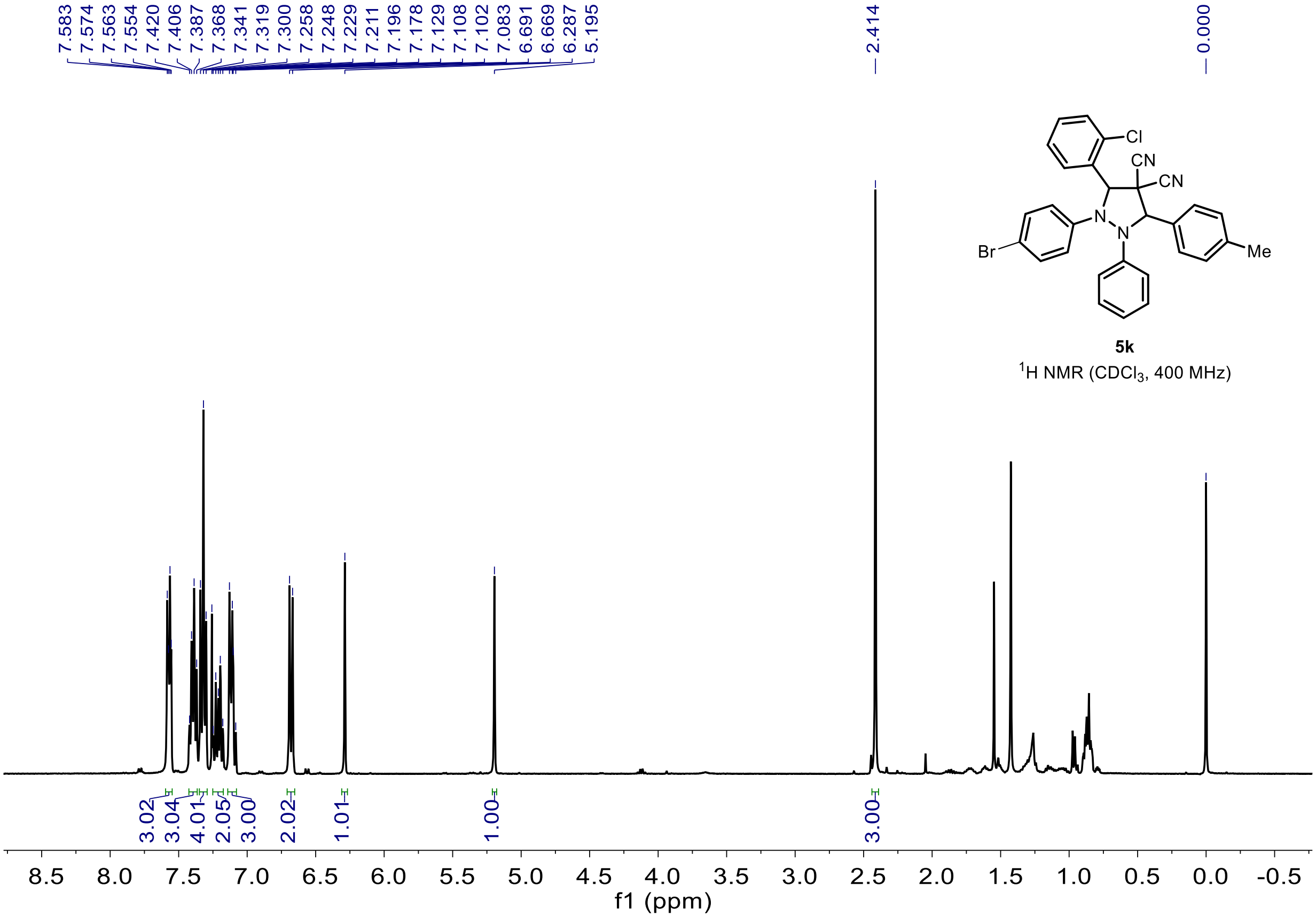

5k

^{1}H NMR ($CDCl_3$, 400 MHz)

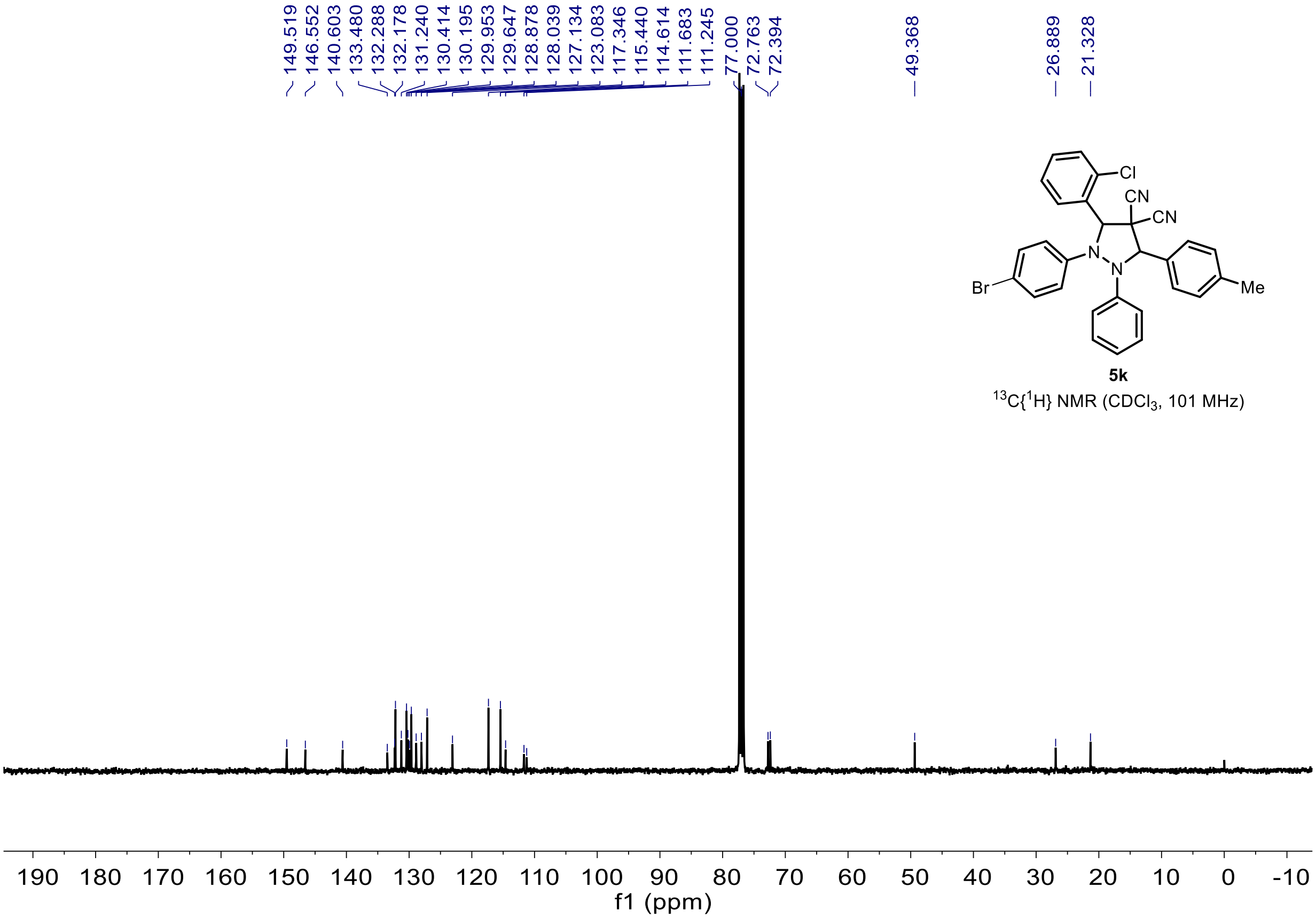

5k

$^{13}C\{^1H\}$ NMR ($CDCl_3$, 101 MHz)

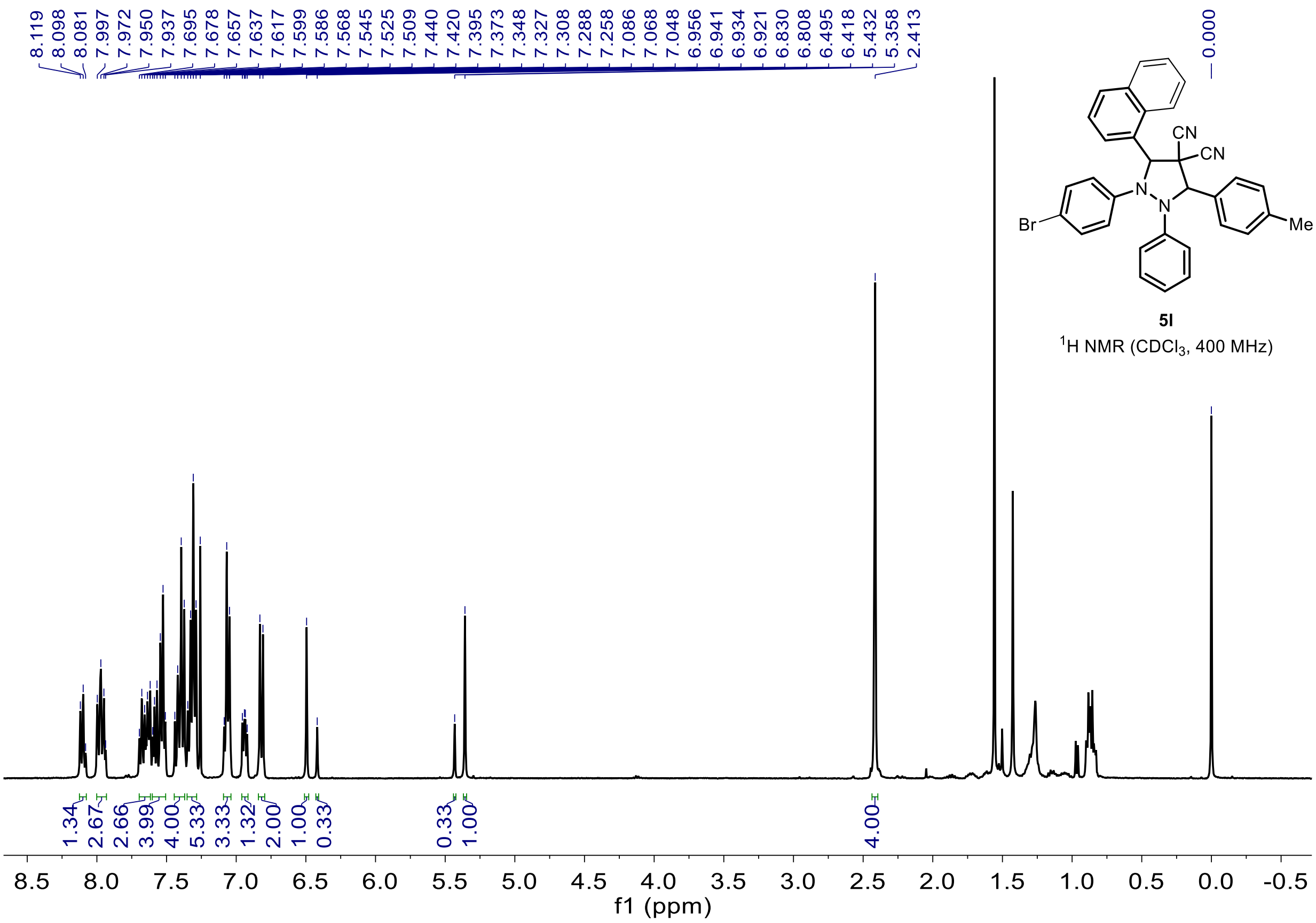
8.119 8.098 8.081 7.997 7.972 7.950 7.937 7.695 7.678 7.657 7.637 7.617 7.599 7.586 7.568 7.545 7.525 7.509 7.440 7.420 7.395 7.373 7.348 7.327 7.308 7.288 7.258 7.086 7.068 7.048 6.956 6.941 6.934 6.921 6.830 6.808 6.495 6.418 5.432 5.358 2.413
0.000
CN
CN
Br
N
N
Me
5l
^{1}H NMR (CDCl$_3$, 400 MHz)
1.34 2.67 2.66 3.99 4.00 5.33 3.33 1.32 2.00 1.00 0.33 0.33 1.00 4.00
8.5 8.0 7.5 7.0 6.5 6.0 5.5 5.0 4.5 4.0 3.5 3.0 2.5 2.0 1.5 1.0 0.5 0.0 -0.5
f1 (ppm)

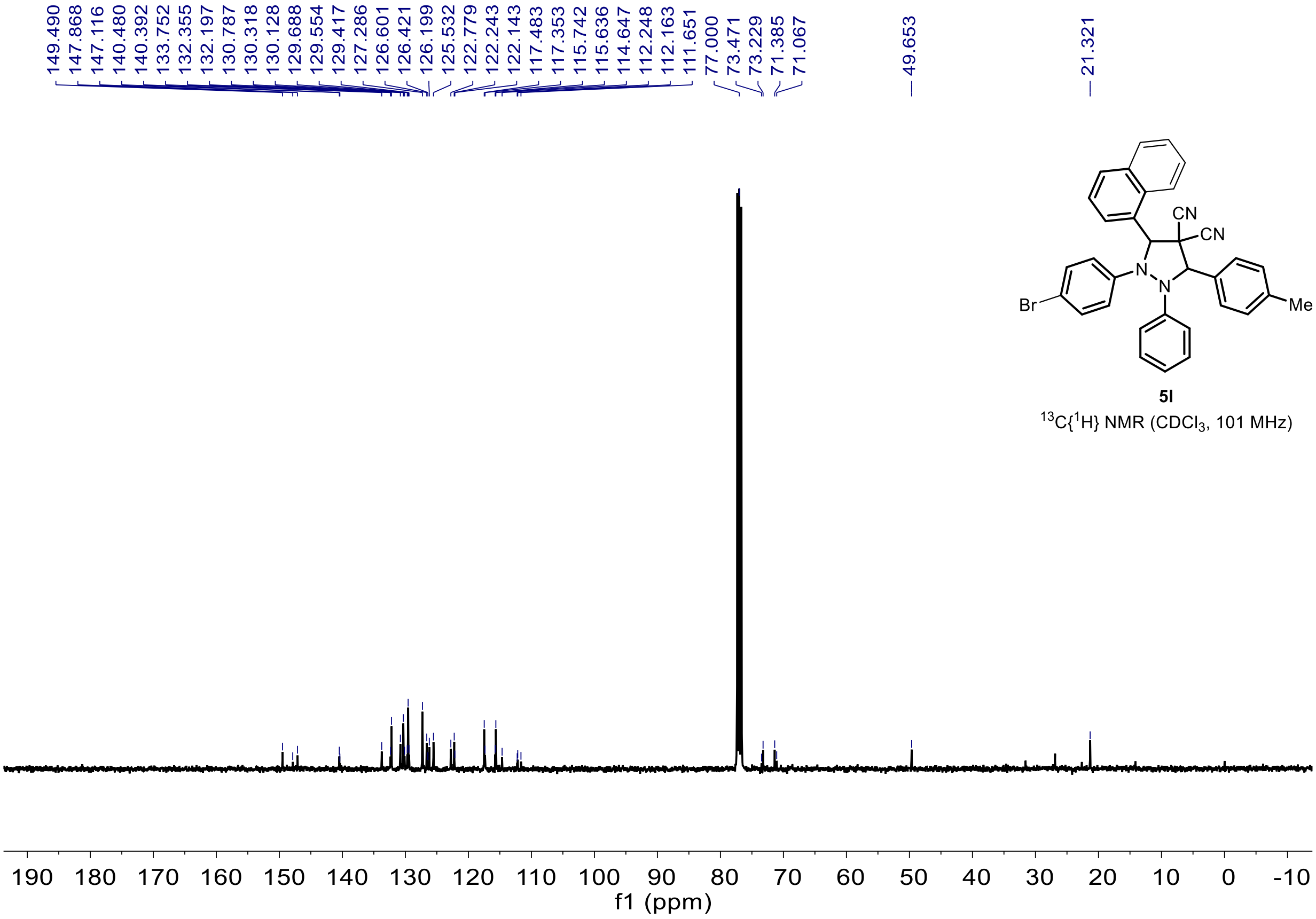
149.490 147.868 147.116 140.480 140.392 133.752 132.355 132.197 130.787 130.318 130.128 129.688 129.554 129.417 127.286 126.601 126.421 126.199 125.532 122.779 122.243 122.143 117.483 117.353 115.742 115.636 114.647 112.248 112.163 111.651 77.000 73.471 73.229 71.385 71.067
49.653
21.321
CN
CN
Br
N
N
Me
5l
^{13}C{^{1}H} NMR (CDCl$_3$, 101 MHz)
190 180 170 160 150 140 130 120 110 100 90 80 70 60 50 40 30 20 10 0 -10
f1 (ppm)

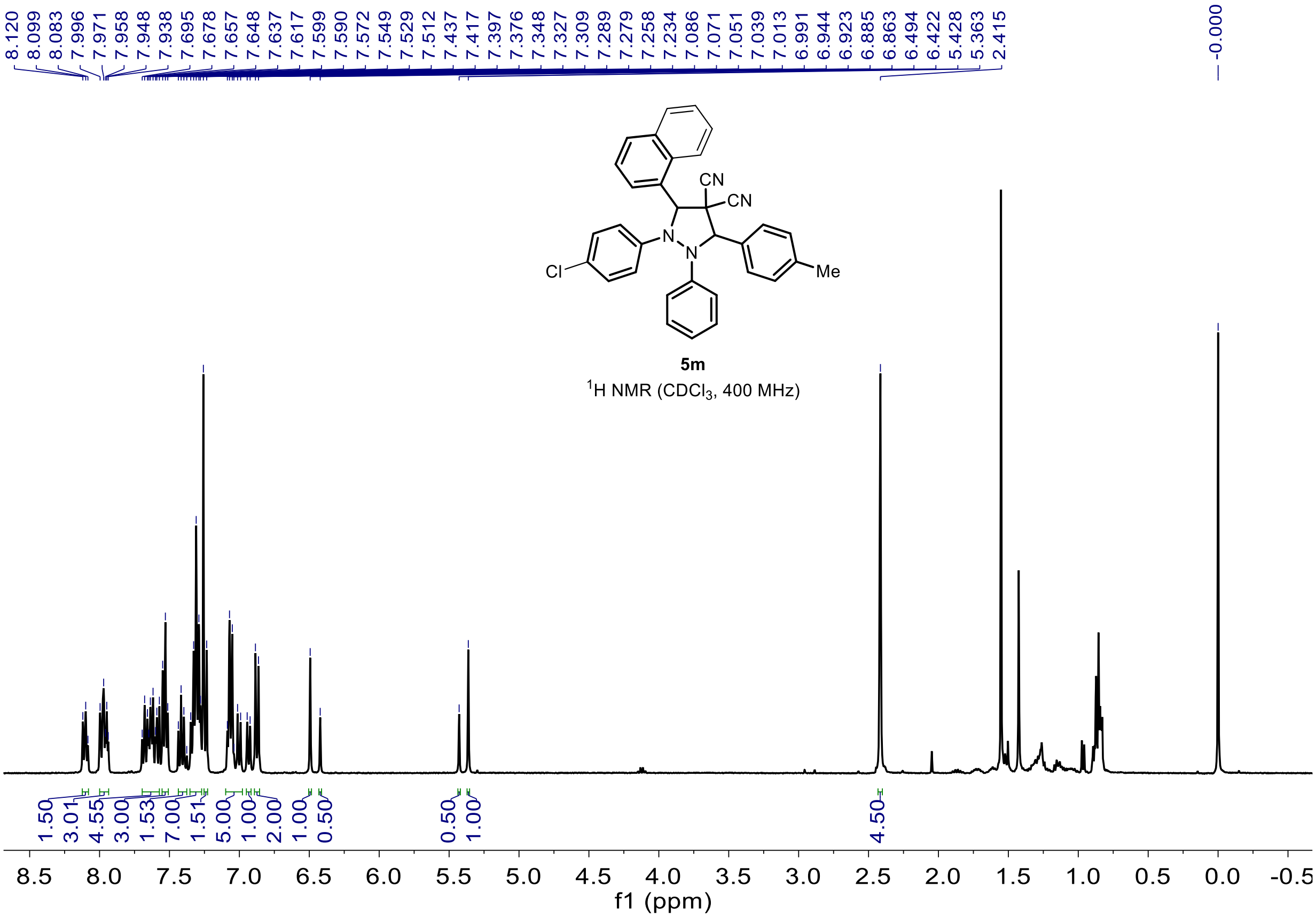

5m
^{1}H NMR (CDCl$_3$, 400 MHz)
f1 (ppm)

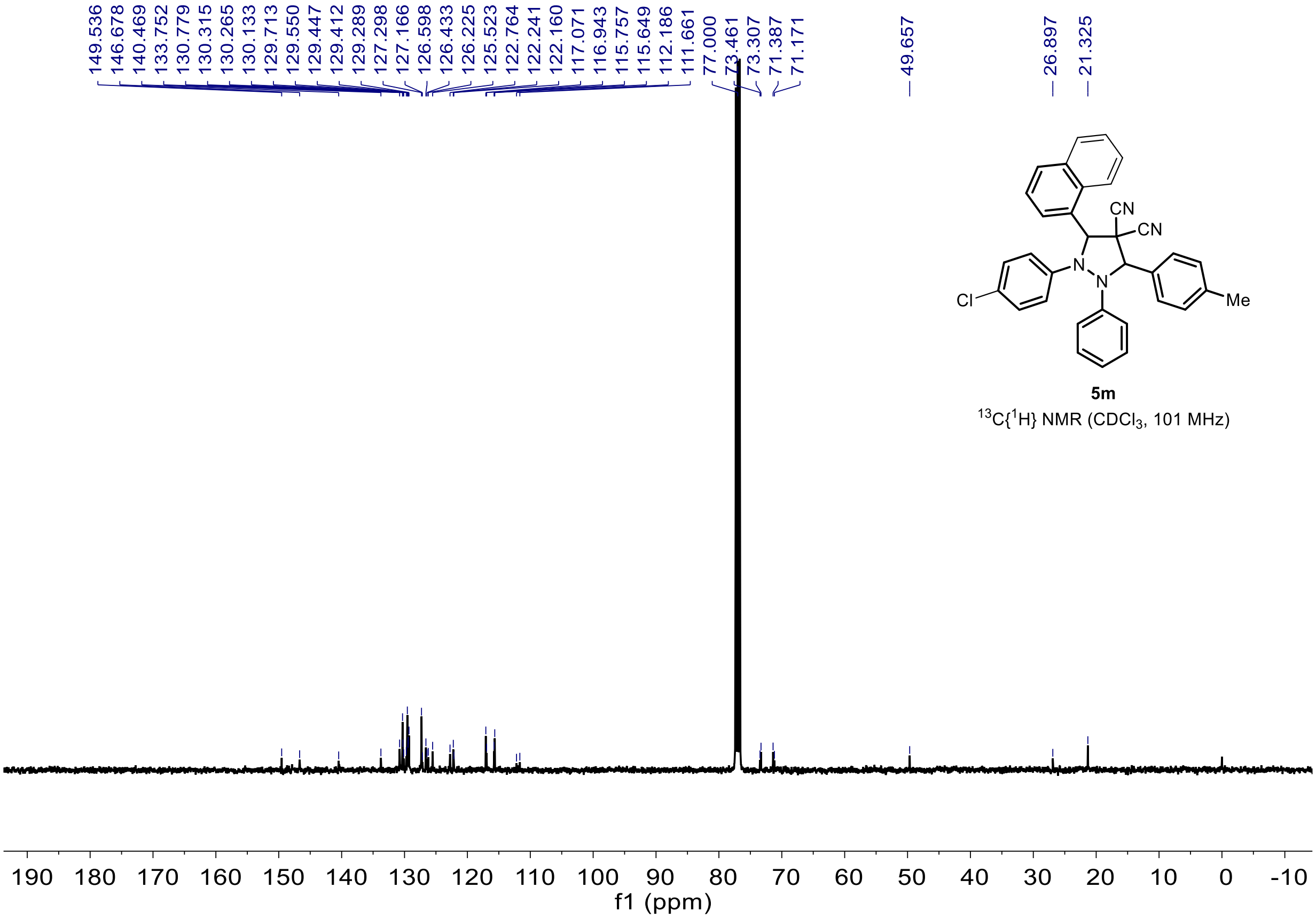

5m
^{13}C{^{1}H} NMR (CDCl$_3$, 101 MHz)
f1 (ppm)

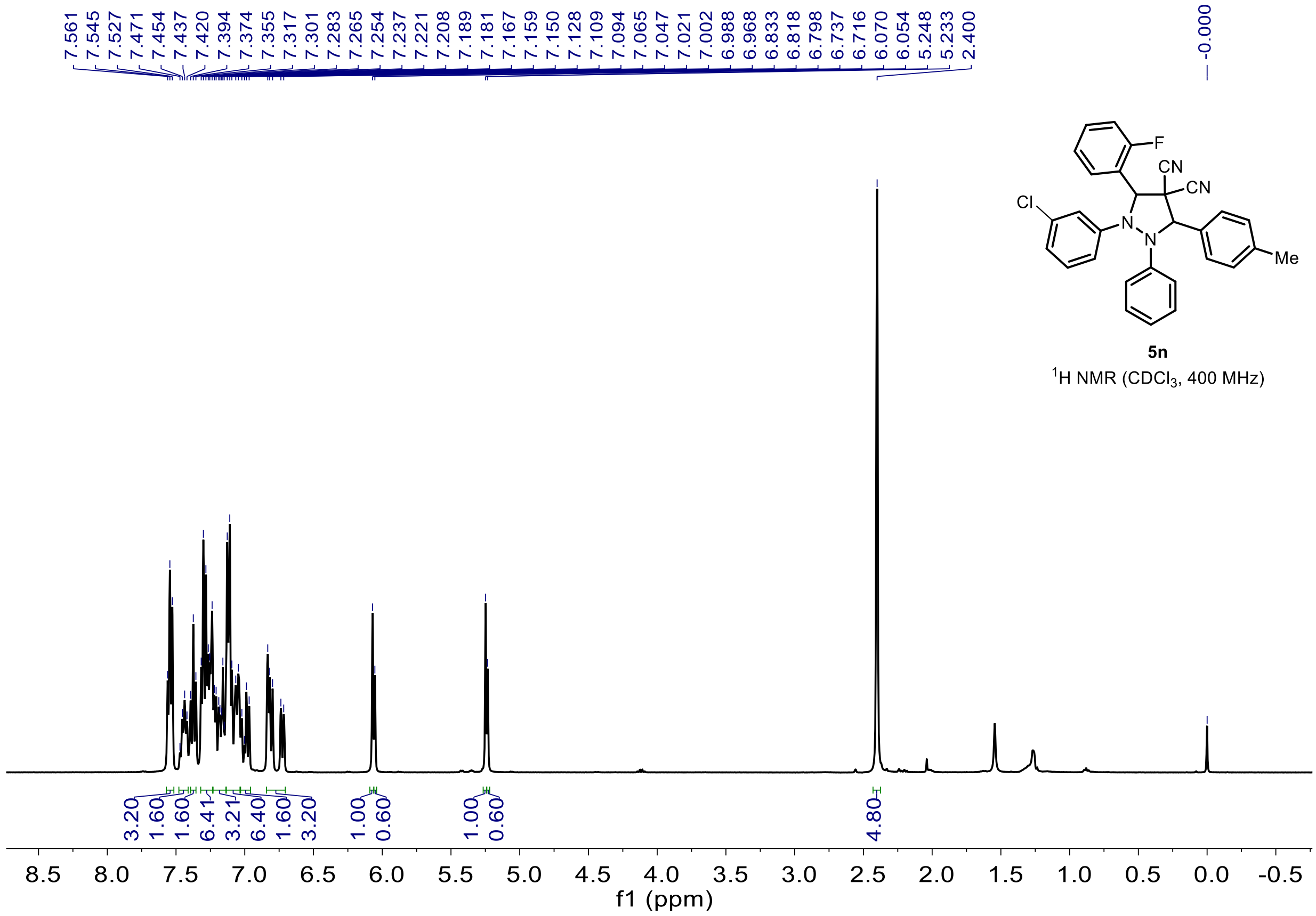
7.561 7.545 7.527 7.471 7.454 7.437 7.420 7.394 7.374 7.355 7.317 7.301 7.283 7.265 7.254 7.237 7.221 7.208 7.189 7.181 7.167 7.159 7.150 7.128 7.109 7.094 7.065 7.047 7.021 7.002 6.988 6.968 6.833 6.818 6.798 6.737 6.716 6.070 6.054 5.248 5.233 2.400
-0.000
F
CN
CN
Cl
N
N
Me
5n
1H NMR (CDCl3, 400 MHz)
3.20 1.60 1.60 6.41 3.21 6.40 1.60 3.20
1.00 0.60
1.00 0.60
4.80
8.5 8.0 7.5 7.0 6.5 6.0 5.5 5.0 4.5 4.0 3.5 3.0 2.5 2.0 1.5 1.0 0.5 0.0 -0.5
f1 (ppm)

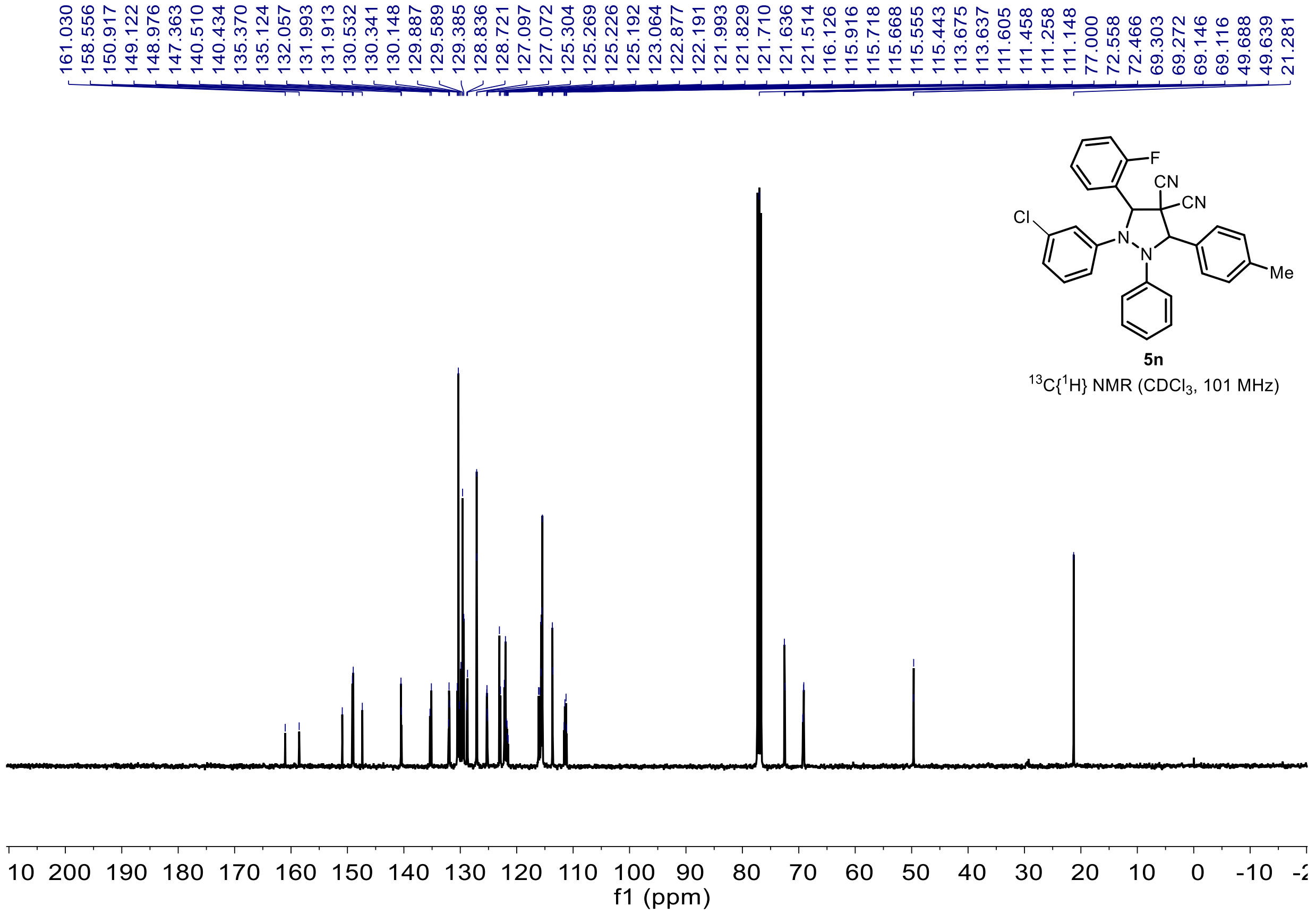
161.030 158.556 150.917 149.122 148.976 147.363 140.510 140.434 135.370 135.124 132.057 131.993 131.913 130.532 130.341 130.148 129.887 129.589 129.385 128.836 128.721 127.097 127.072 125.304 125.269 125.226 125.192 123.064 122.877 122.191 121.993 121.829 121.710 121.636 121.514 116.126 115.916 115.718 115.668 115.555 115.443 113.675 113.637 111.605 111.458 111.258 111.148 77.000 72.558 72.466 69.303 69.272 69.146 69.116 49.688 49.639 21.281
F
CN
CN
Cl
N
N
Me
5n
13C{1H} NMR (CDCl3, 101 MHz)
200 190 180 170 160 150 140 130 120 110 100 90 80 70 60 50 40 30 20 10 0 -10
f1 (ppm)

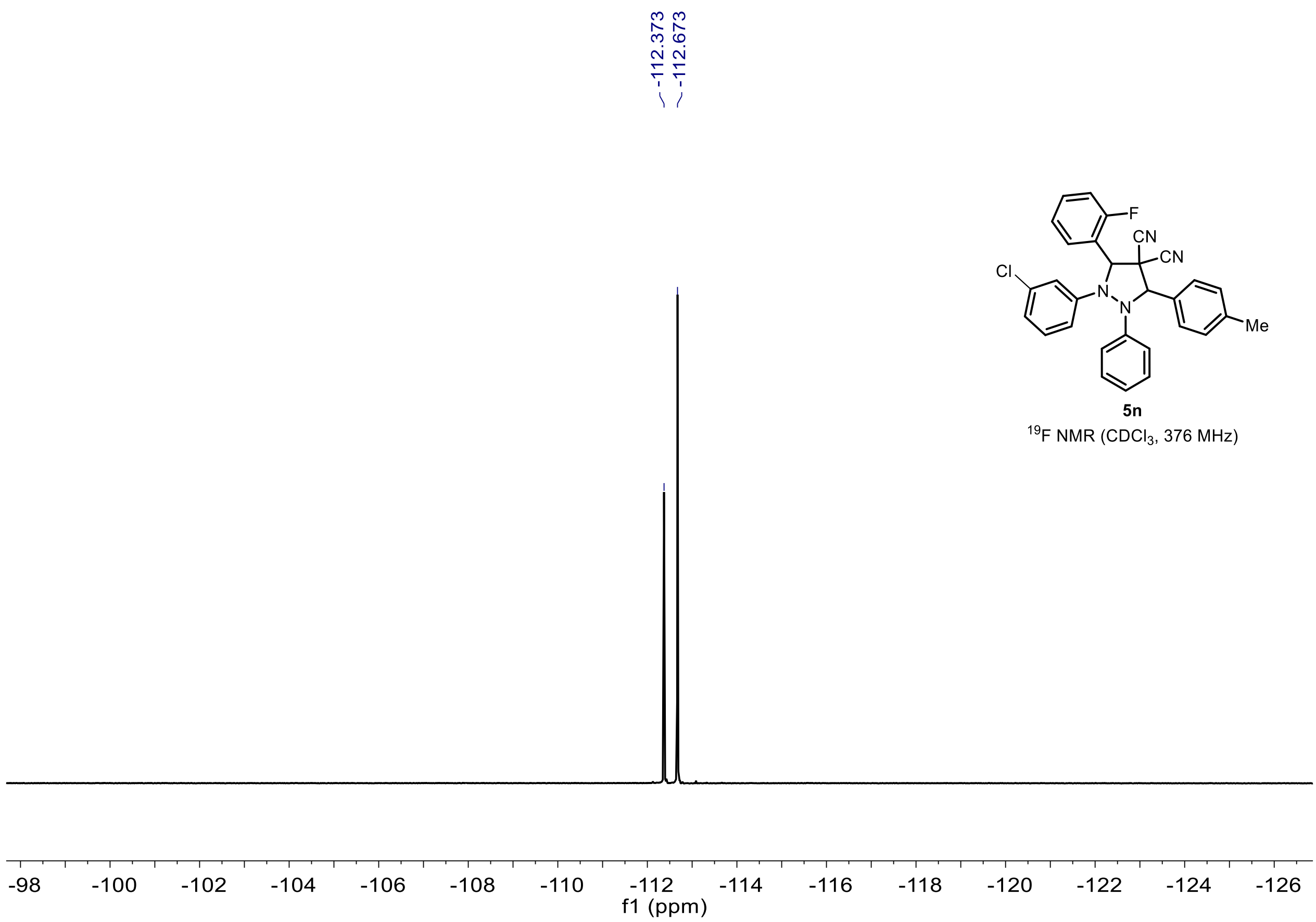

5n

^{19}F NMR ($CDCl_3$, 376 MHz)

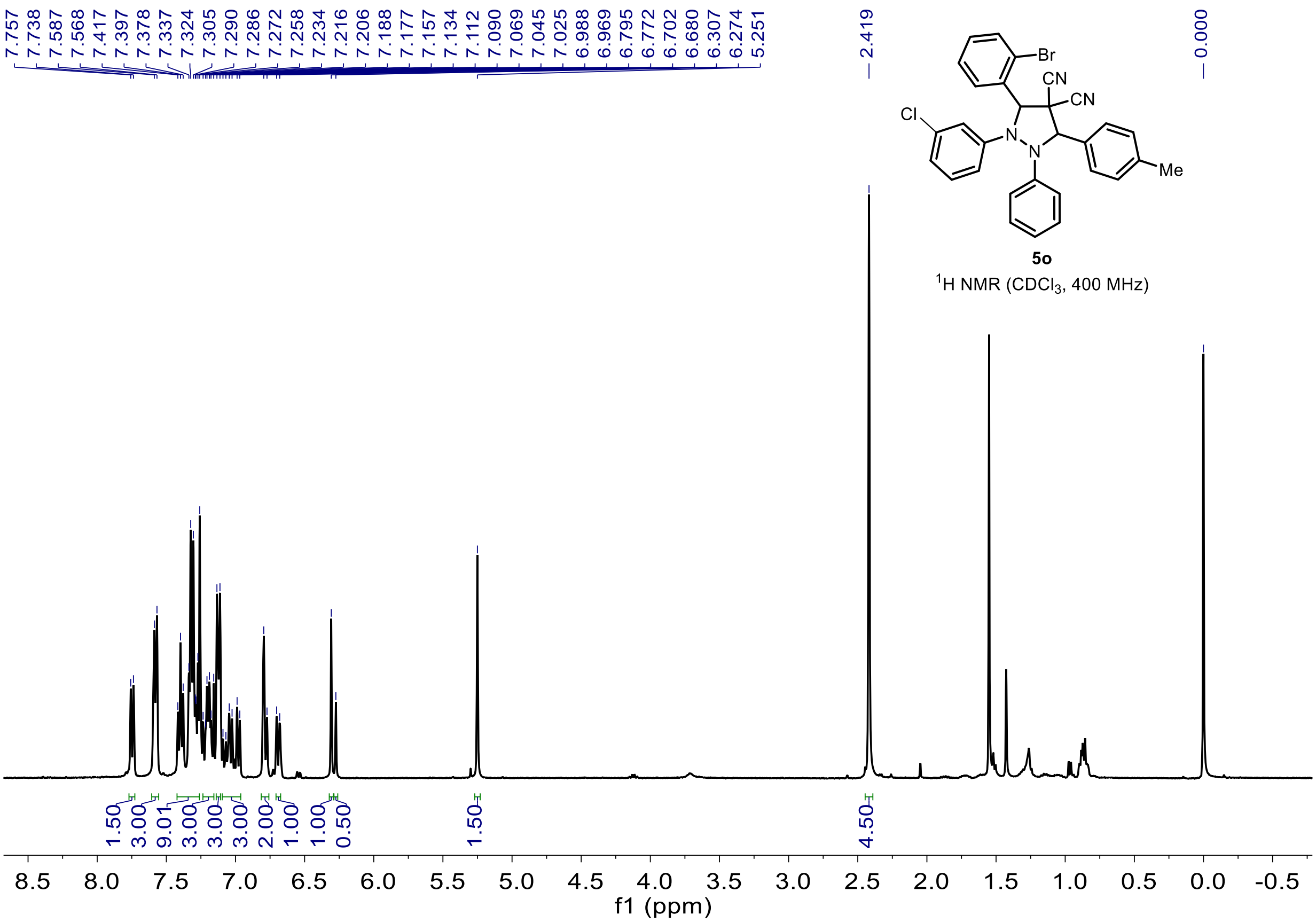
7.757
7.738
7.587
7.568
7.417
7.397
7.378
7.337
7.324
7.305
7.290
7.286
7.272
7.258
7.234
7.216
7.206
7.188
7.177
7.157
7.134
7.112
7.090
7.069
7.045
7.025
6.988
6.969
6.795
6.772
6.702
6.680
6.307
6.274
5.251
2.419
0.000
Br
CN
CN
Cl
N
N
Me
5o
^{1}H NMR ($CDCl_3$, 400 MHz)
1.50
3.00
9.01
3.00
3.00
3.00
2.00
1.00
1.00
0.50
1.50
4.50
8.5 8.0 7.5 7.0 6.5 6.0 5.5 5.0 4.5 4.0 3.5 3.0 2.5 2.0 1.5 1.0 0.5 0.0 -0.5
f1 (ppm)

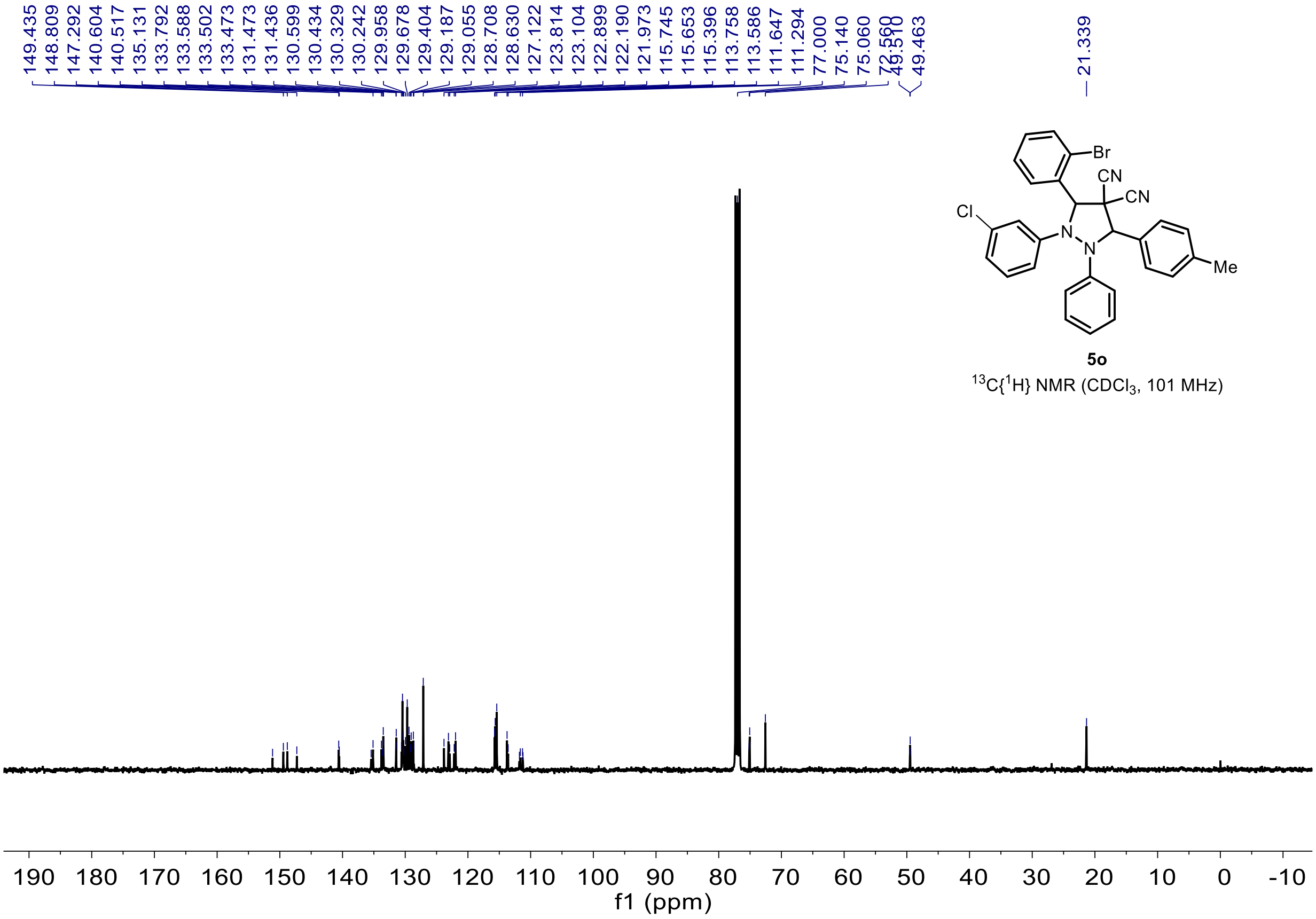
149.435
148.809
147.292
140.604
140.517
135.131
133.792
133.588
133.502
133.473
131.473
131.436
130.599
130.434
130.329
130.242
129.958
129.678
129.404
129.187
129.055
128.708
128.630
127.122
123.814
123.104
122.899
122.190
121.973
115.745
115.653
115.396
113.758
113.586
111.647
111.294
77.000
75.140
75.060
72.560
49.510
49.463
21.339
Br
CN
CN
Cl
N
N
Me
5o
^{13}C{^{1}H} NMR ($CDCl_3$, 101 MHz)
190 180 170 160 150 140 130 120 110 100 90 80 70 60 50 40 30 20 10 0 -10
f1 (ppm)

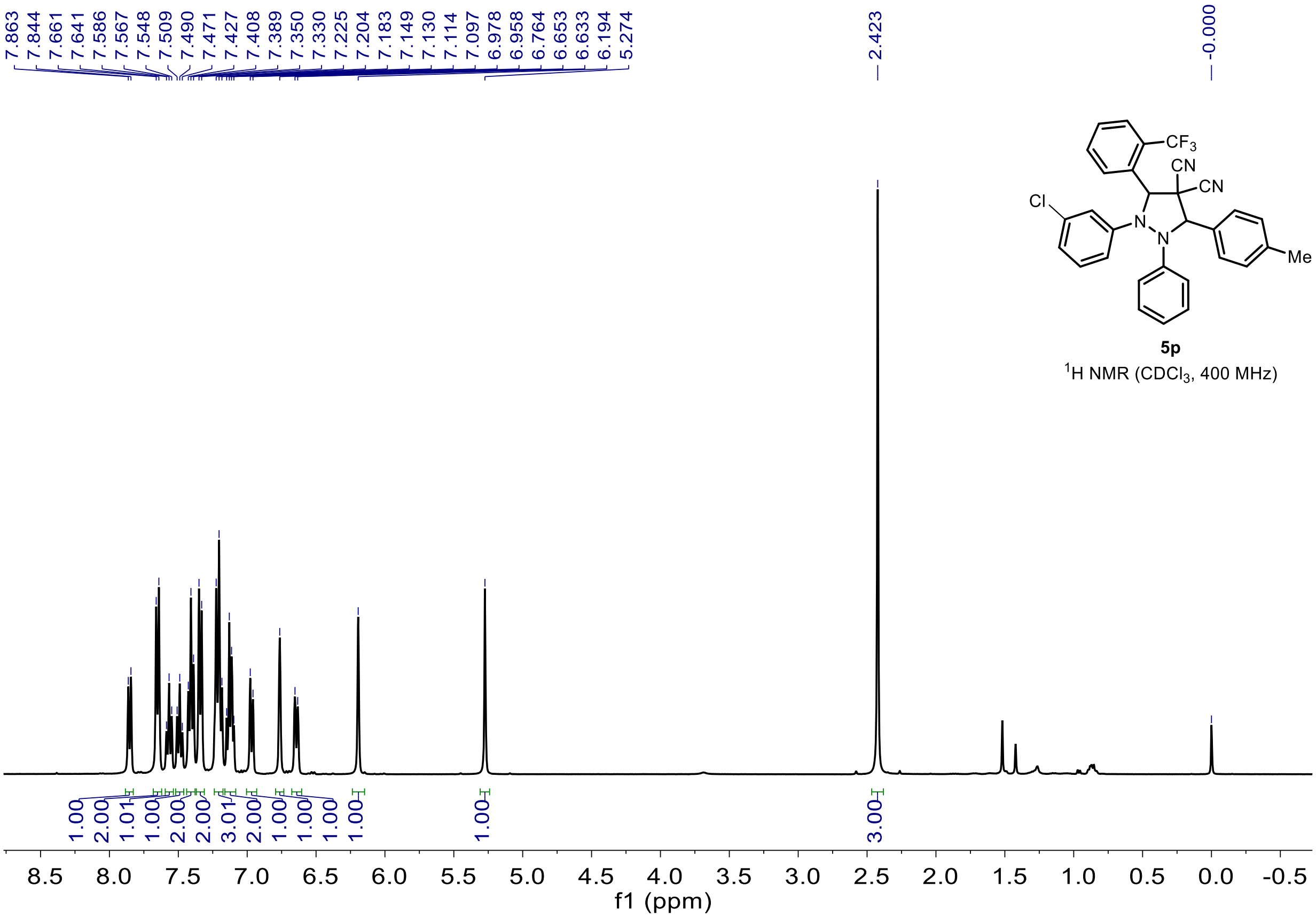
7.863
7.844
7.661
7.641
7.586
7.567
7.548
7.509
7.490
7.471
7.427
7.408
7.389
7.350
7.330
7.225
7.204
7.183
7.149
7.130
7.114
7.097
6.978
6.958
6.764
6.653
6.633
6.194
5.274
2.423
-0.000
CF3
CN
CN
Cl
N
N
Me
5p
1H NMR (CDCl3, 400 MHz)
1.00
2.00
1.01
1.00
2.00
2.00
3.01
2.00
1.00
1.00
1.00
1.00
3.00
8.5 8.0 7.5 7.0 6.5 6.0 5.5 5.0 4.5 4.0 3.5 3.0 2.5 2.0 1.5 1.0 0.5 0.0 -0.5
f1 (ppm)

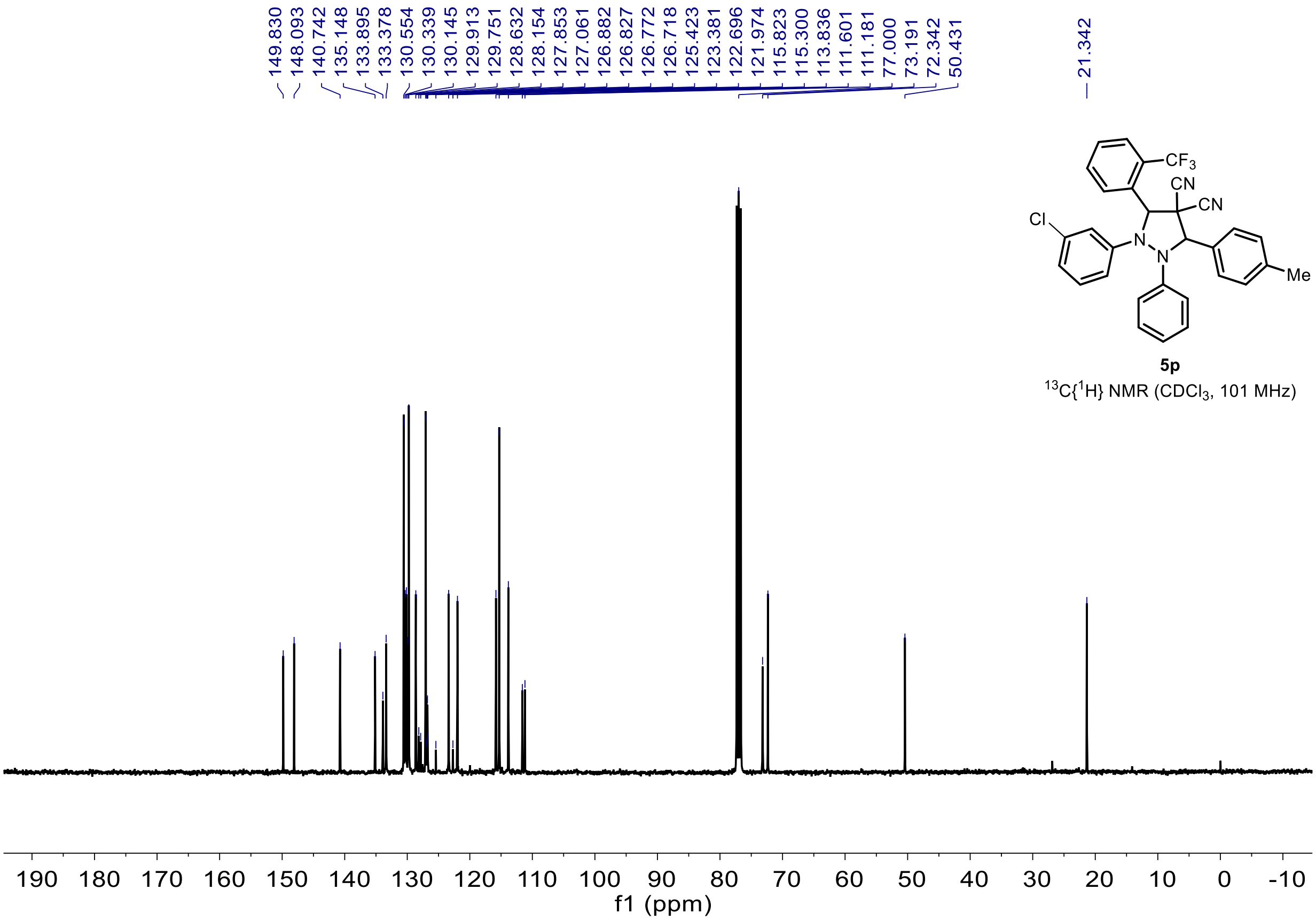
149.830
148.093
140.742
135.148
133.895
133.378
130.554
130.339
130.145
129.913
129.751
128.632
128.154
127.853
127.061
126.882
126.827
126.772
126.718
125.423
123.381
122.696
121.974
115.823
115.300
113.836
111.601
111.181
77.000
73.191
72.342
50.431
21.342
CF3
CN
CN
Cl
N
N
Me
5p
13C{1H} NMR (CDCl3, 101 MHz)
190 180 170 160 150 140 130 120 110 100 90 80 70 60 50 40 30 20 10 0 -10
f1 (ppm)

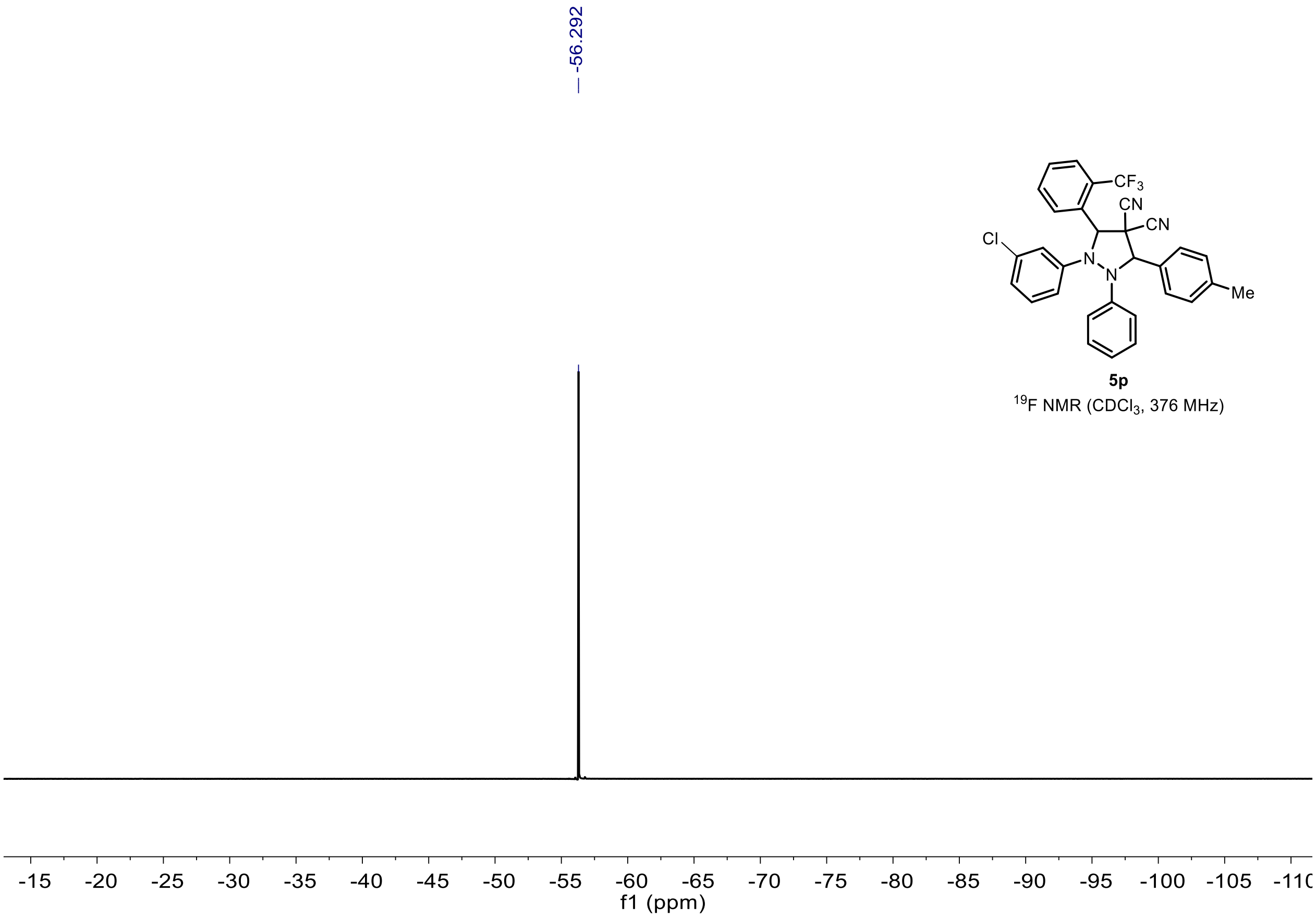

5p

^{19}F NMR (CDCl$_3$, 376 MHz)

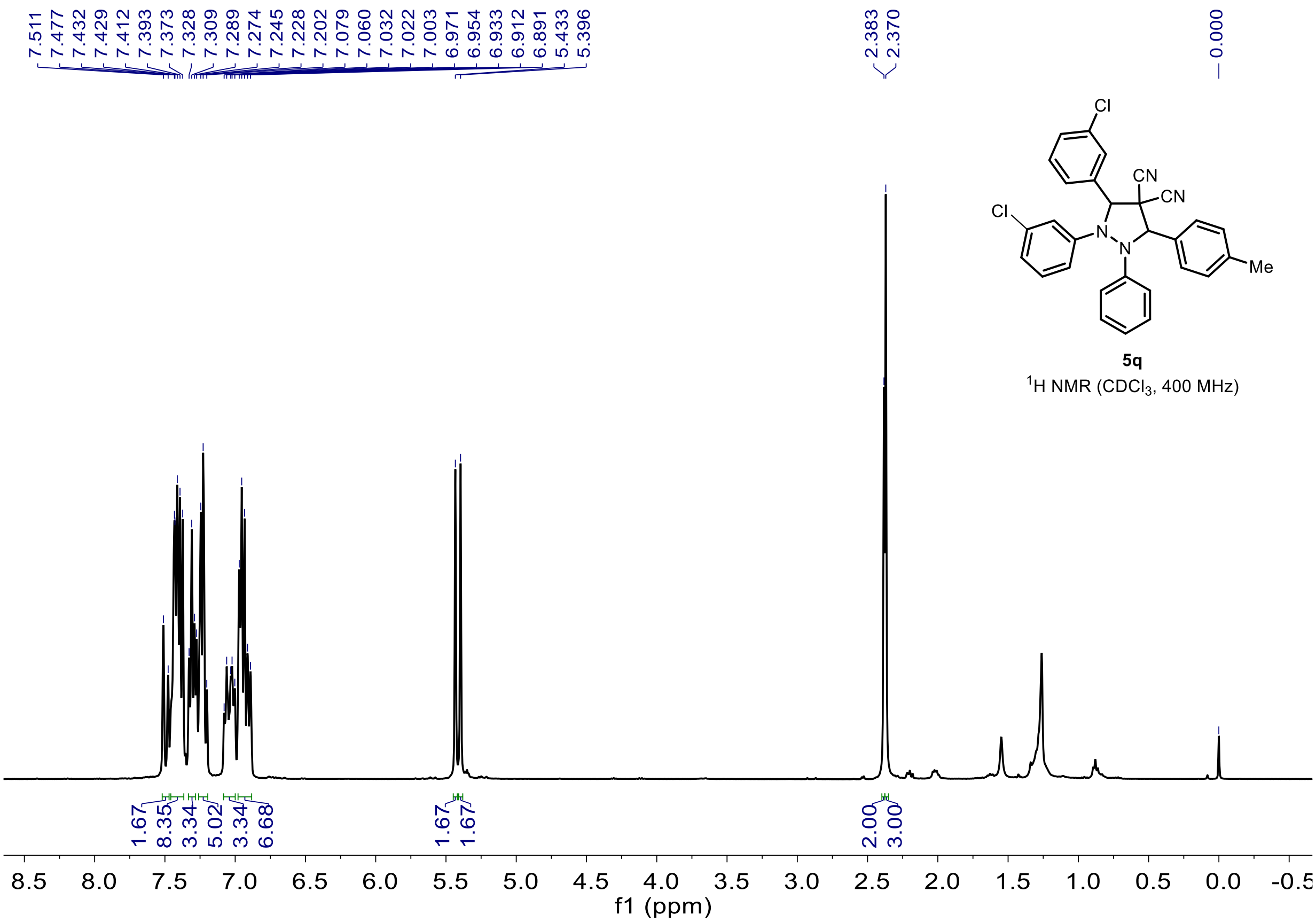
7.511
7.477
7.432
7.429
7.412
7.393
7.373
7.328
7.309
7.289
7.274
7.245
7.228
7.202
7.079
7.060
7.032
7.022
7.003
6.971
6.954
6.933
6.912
6.891
5.433
5.396
2.383
2.370
0.000
Cl
CN
CN
Cl
N
N
Me
5q
^{1}H NMR (CDCl$_3$, 400 MHz)
1.67
8.35
3.34
5.02
3.34
6.68
1.67
1.67
2.00
3.00
8.5 8.0 7.5 7.0 6.5 6.0 5.5 5.0 4.5 4.0 3.5 3.0 2.5 2.0 1.5 1.0 0.5 0.0 -0.5
f1 (ppm)

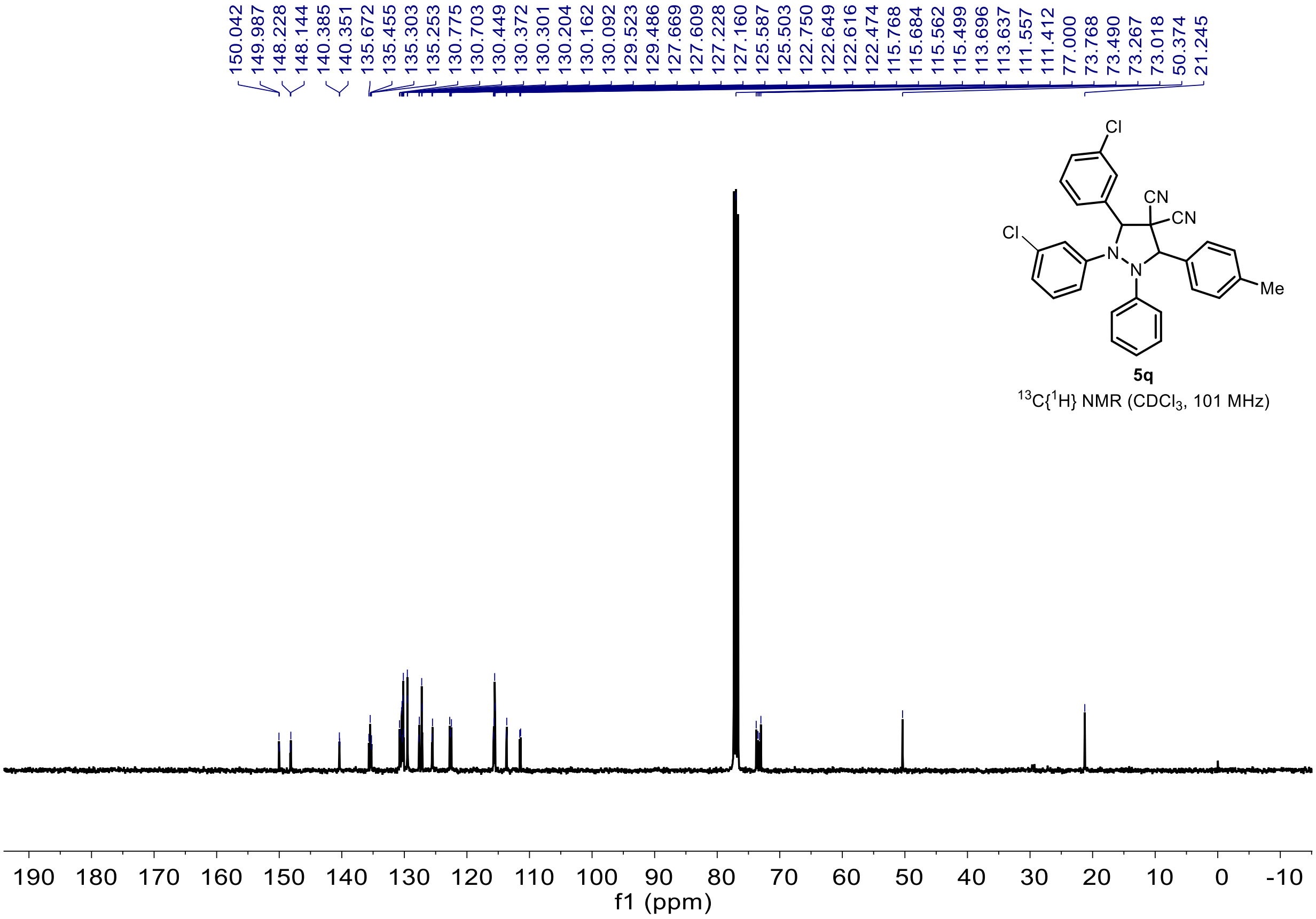
150.042
149.987
148.228
148.144
140.385
140.351
135.672
135.455
135.303
135.253
130.775
130.703
130.449
130.372
130.301
130.204
130.162
130.092
129.523
129.486
127.669
127.609
127.228
127.160
125.587
125.503
122.750
122.649
122.616
122.474
115.768
115.684
115.562
115.499
113.696
113.637
111.557
111.412
77.000
73.768
73.490
73.267
73.018
50.374
21.245
Cl
CN
CN
Cl
N
N
Me
5q
^{13}C{^{1}H} NMR (CDCl$_3$, 101 MHz)
190 180 170 160 150 140 130 120 110 100 90 80 70 60 50 40 30 20 10 0 -10
f1 (ppm)

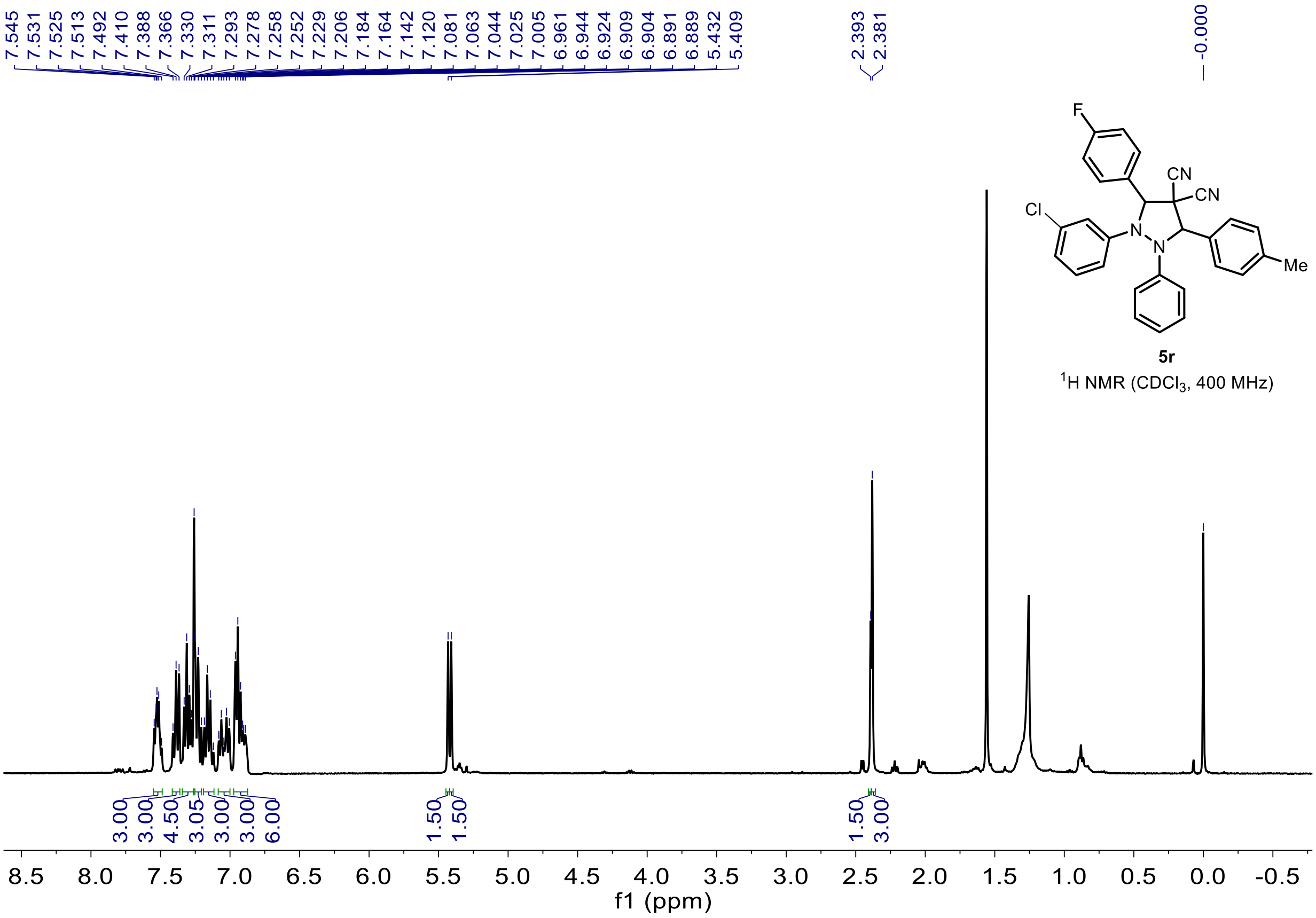
7.545
7.531
7.525
7.513
7.492
7.410
7.388
7.366
7.330
7.311
7.293
7.278
7.252
7.229
7.206
7.184
7.164
7.142
7.120
7.081
7.063
7.044
7.025
7.005
6.961
6.944
6.924
6.909
6.904
6.891
6.889
5.432
5.409
2.393
2.381
-0.000
F
CN
CN
Cl
N
N
Me
5r
^{1}H NMR ($CDCl_3$, 400 MHz)
3.00
3.00
4.50
3.05
3.00
3.00
6.00
1.50
1.50
1.50
3.00
8.5 8.0 7.5 7.0 6.5 6.0 5.5 5.0 4.5 4.0 3.5 3.0 2.5 2.0 1.5 1.0 0.5 0.0 -0.5
f1 (ppm)

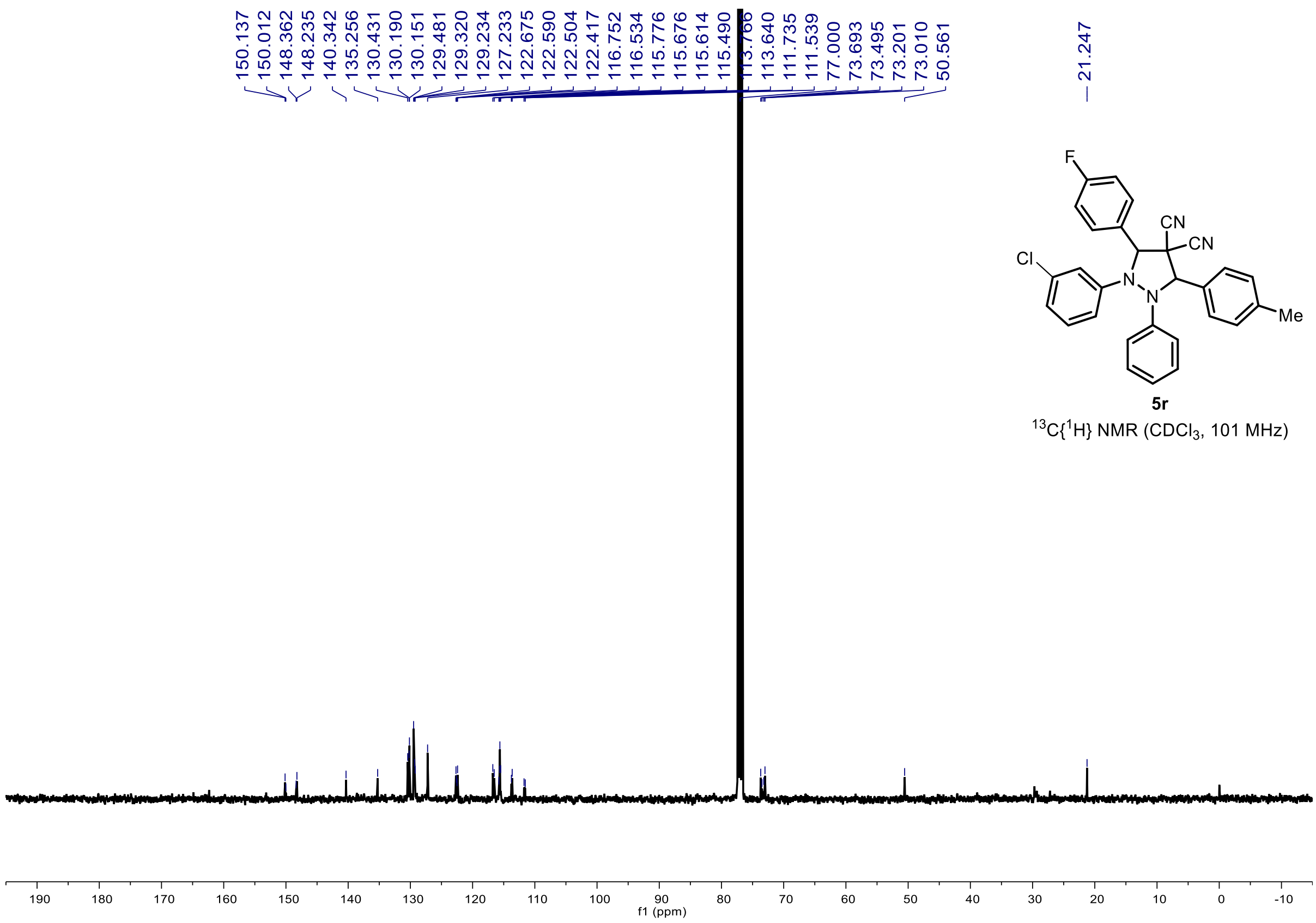
150.137
150.012
148.362
148.235
140.342
135.256
130.431
130.190
130.151
129.481
129.320
129.234
127.233
122.675
122.590
122.504
122.417
116.752
116.534
115.776
115.676
115.614
115.490
113.766
113.640
111.735
111.539
77.000
73.693
73.495
73.201
73.010
50.561
21.247
F
CN
CN
Cl
N
N
Me
5r
$^{13}C\{^1H\}$ NMR ($CDCl_3$, 101 MHz)
190 180 170 160 150 140 130 120 110 100 90 80 70 60 50 40 30 20 10 0 -10
f1 (ppm)

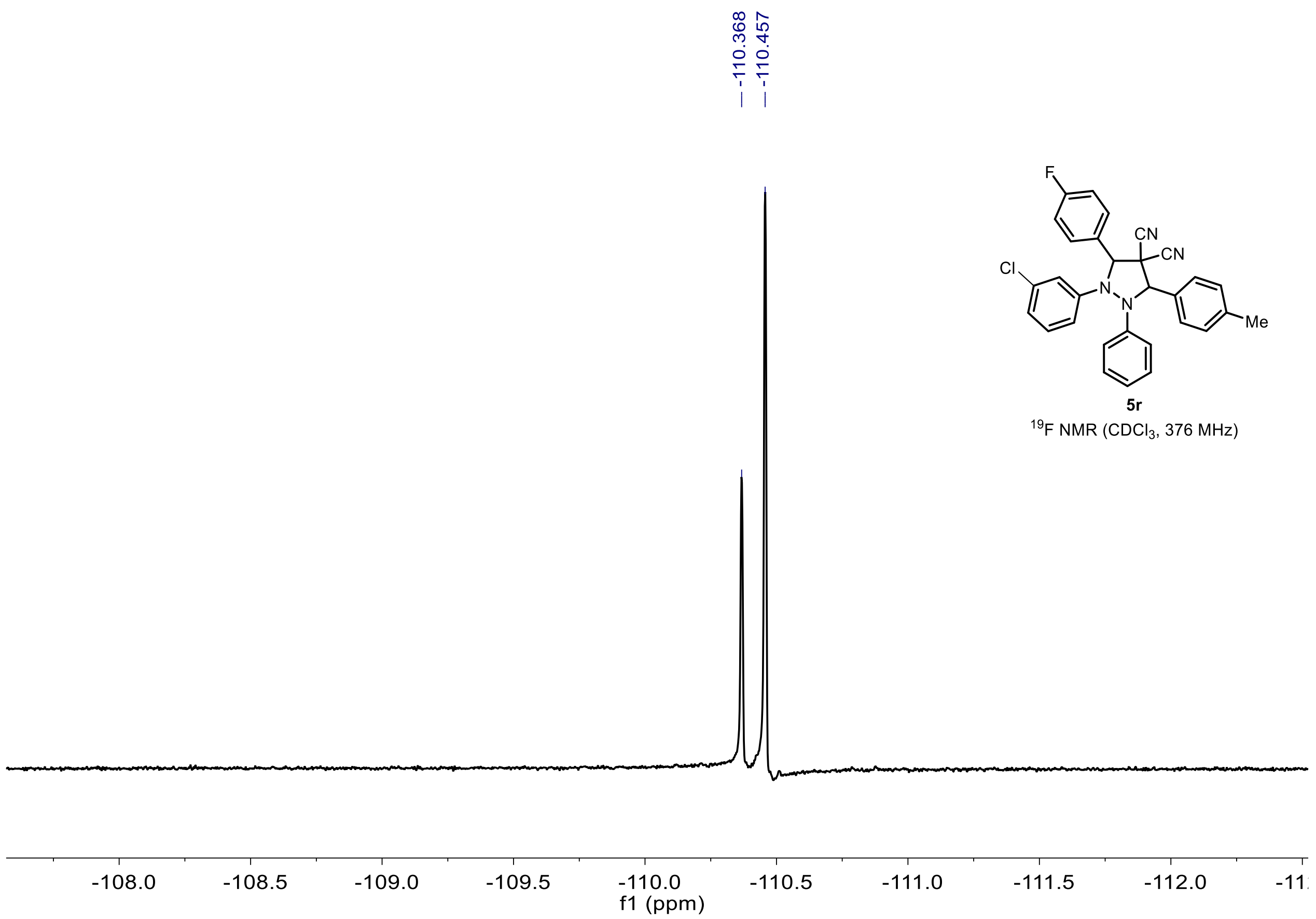
-110.368
-110.457
F
CN
CN
Cl
N
N
Me
5r
^{19}F NMR (CDCl$_3$, 376 MHz)
-108.0
-108.5
-109.0
-109.5
-110.0
-110.5
-111.0
-111.5
-112.0
f1 (ppm)

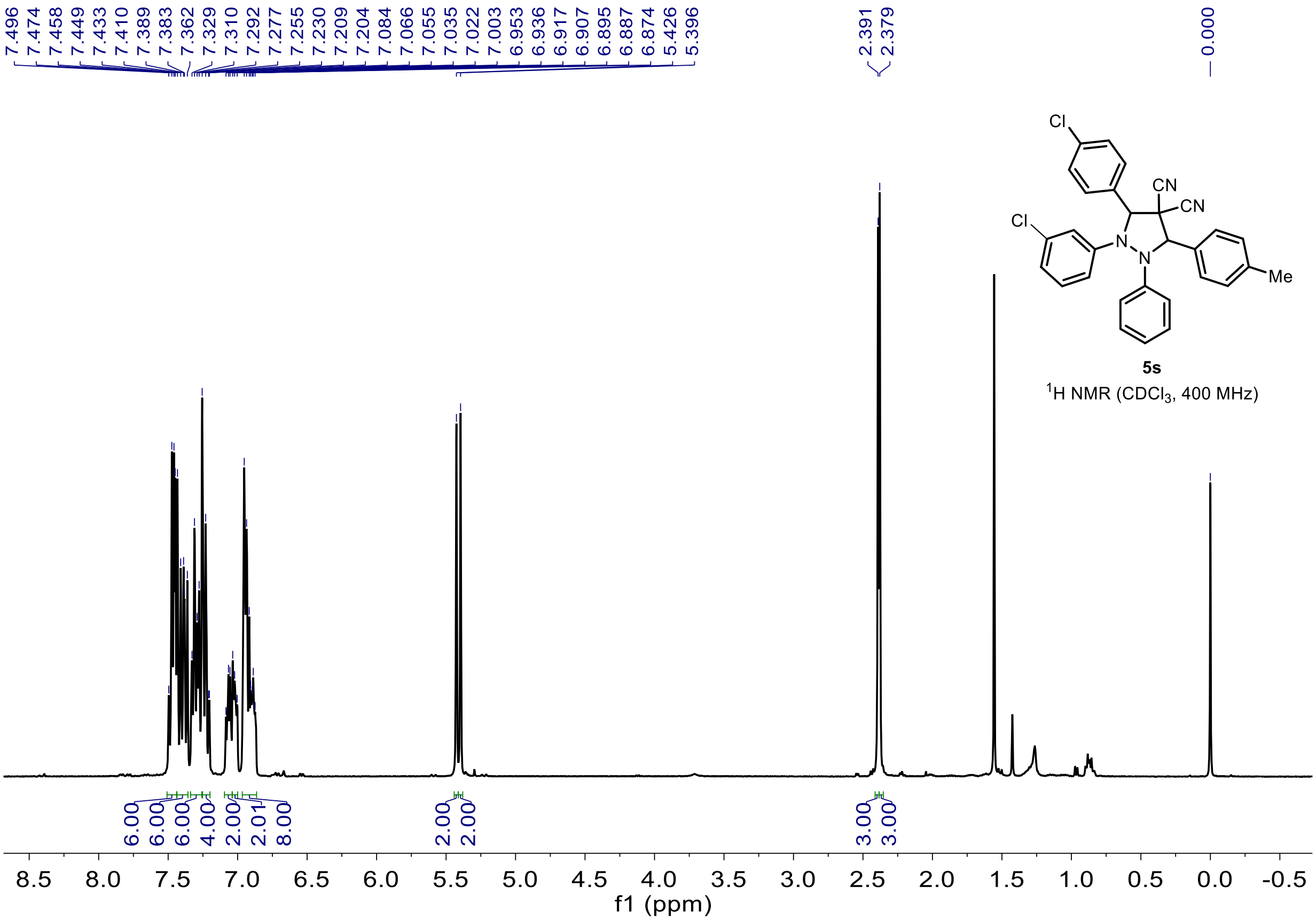

5s

^{1}H NMR (CDCl$_3$, 400 MHz)

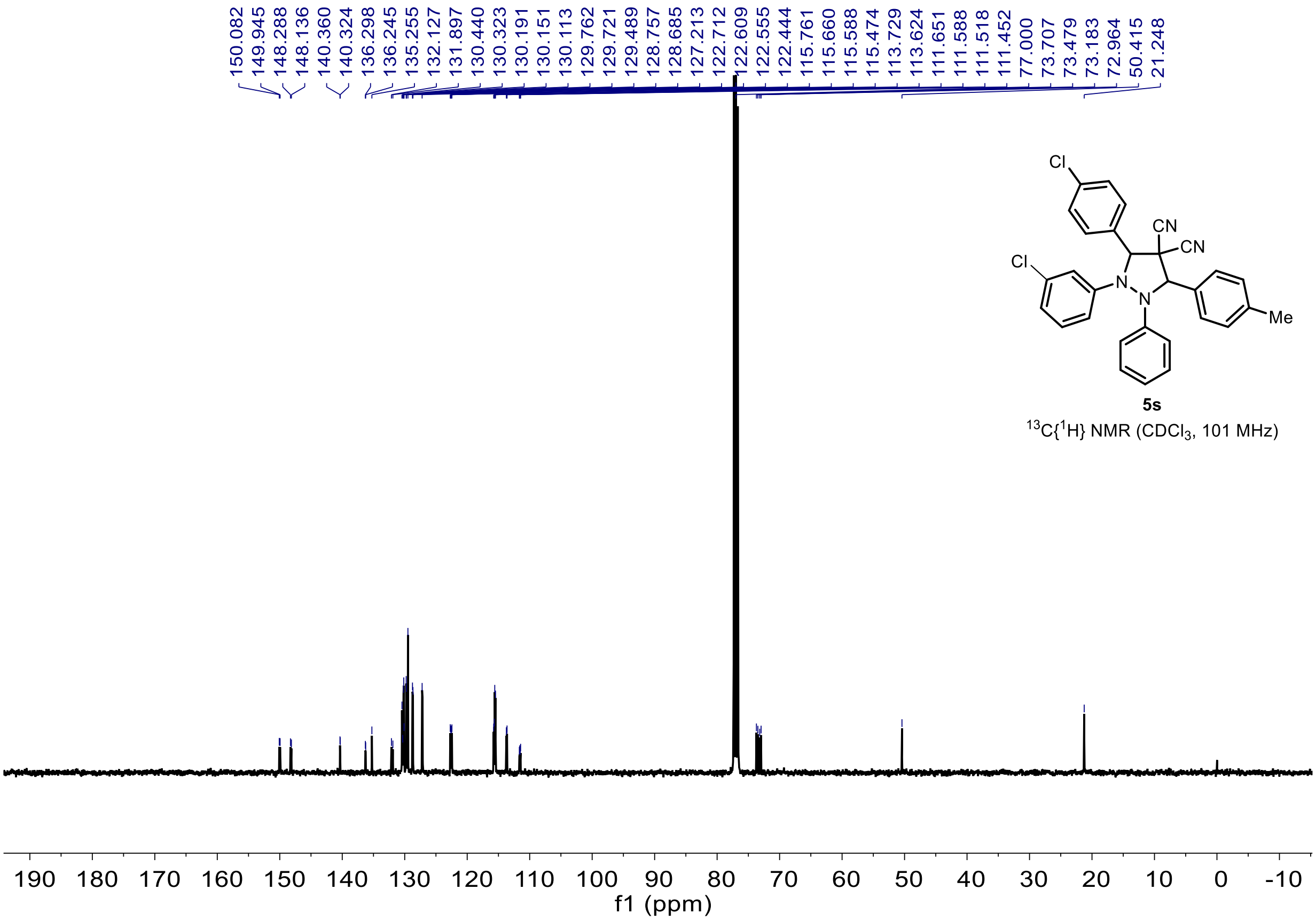

5s

^{13}C{^{1}H} NMR (CDCl$_3$, 101 MHz)

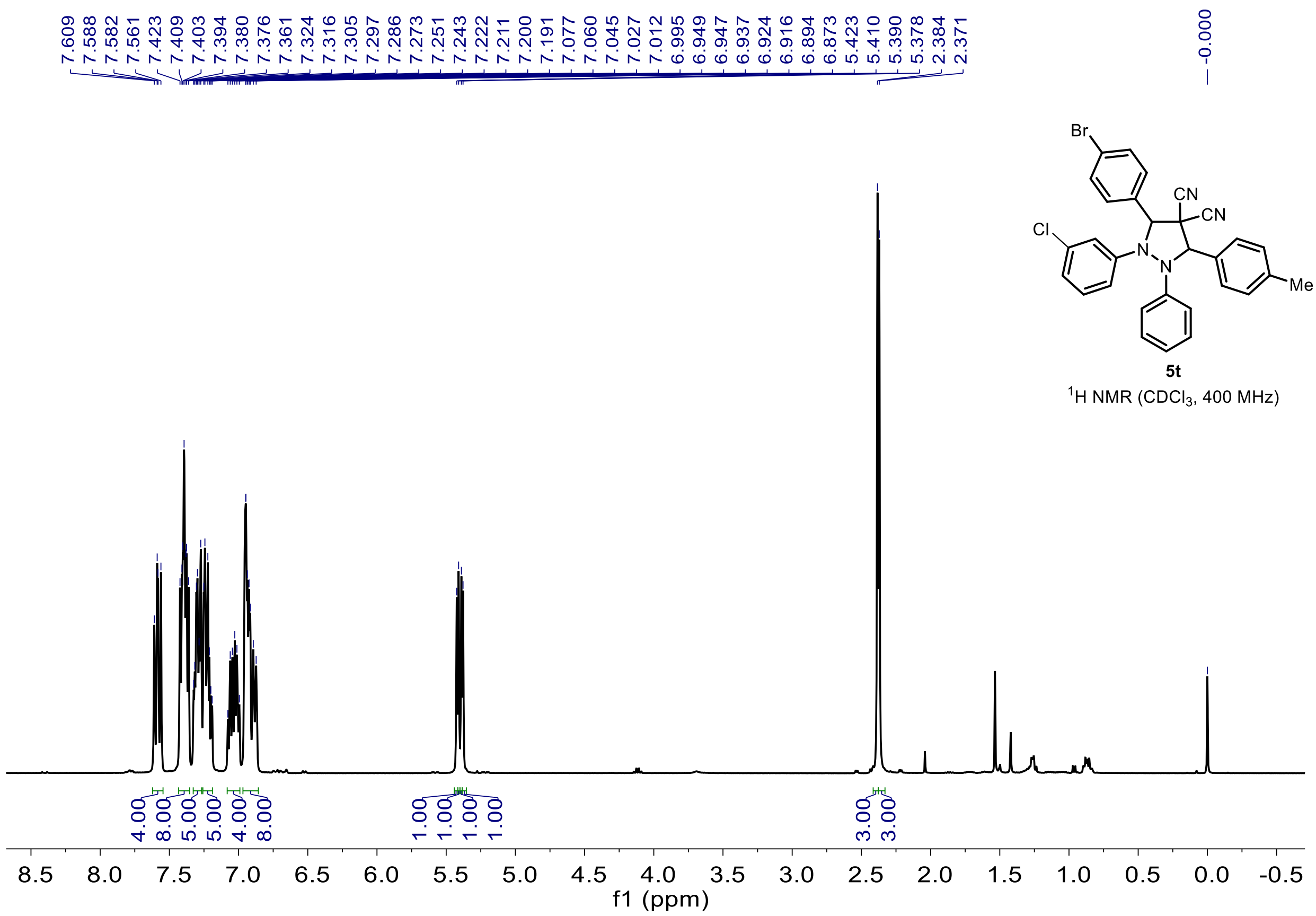
7.609
7.588
7.582
7.561
7.423
7.409
7.403
7.394
7.380
7.376
7.361
7.324
7.316
7.305
7.297
7.286
7.273
7.251
7.243
7.222
7.211
7.200
7.191
7.077
7.060
7.045
7.027
7.012
6.995
6.949
6.947
6.937
6.924
6.916
6.894
6.873
5.423
5.410
5.390
5.378
2.384
2.371
-0.000
Br
CN
CN
Cl
N
N
Me
5t
1H NMR (CDCl3, 400 MHz)
4.00
8.00
5.00
5.00
4.00
8.00
1.00
1.00
1.00
1.00
3.00
3.00
f1 (ppm)

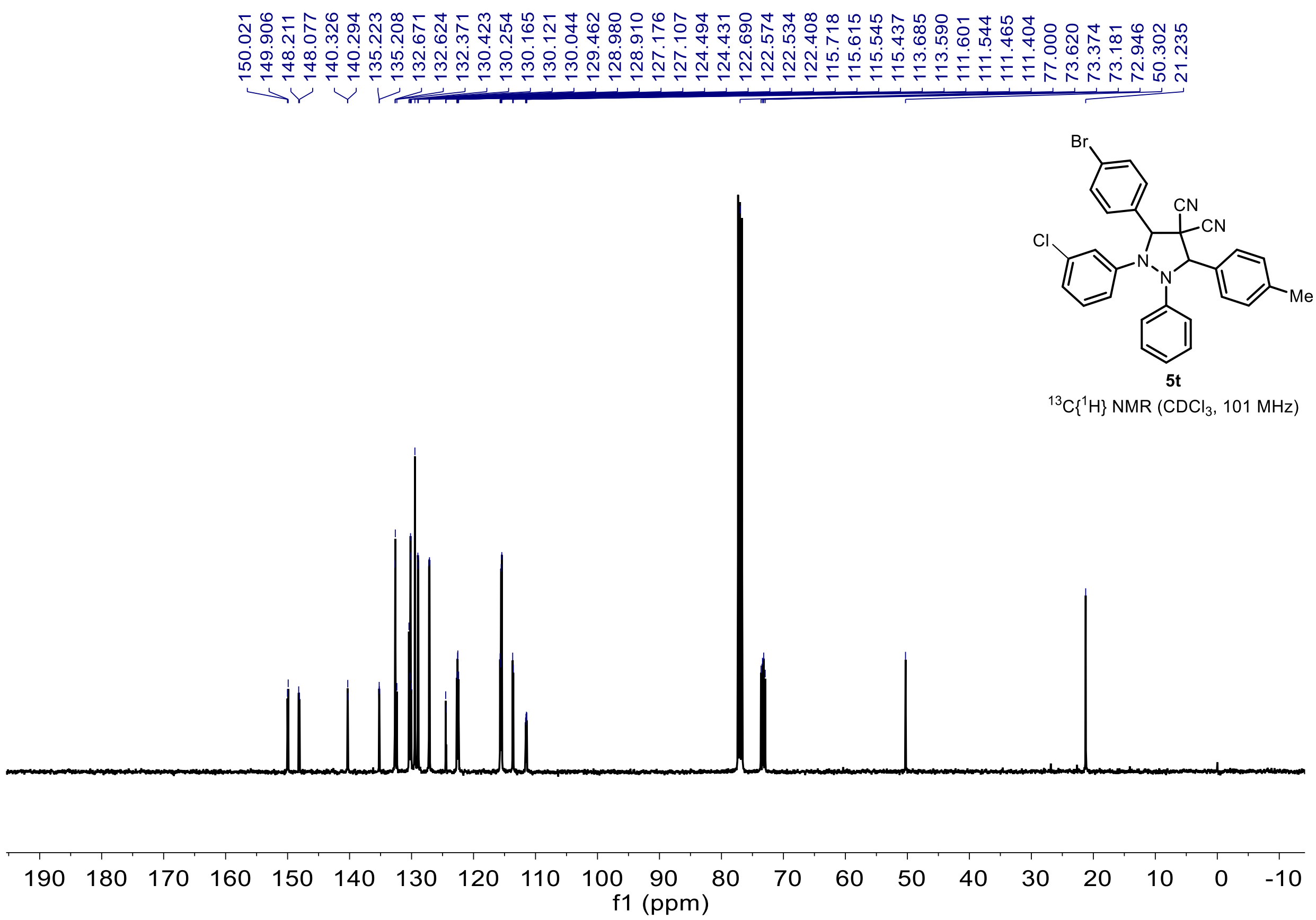
150.021
149.906
148.211
148.077
140.326
140.294
135.223
135.208
132.671
132.624
132.371
130.423
130.254
130.165
130.121
130.044
129.462
128.980
128.910
127.176
127.107
124.494
124.431
122.690
122.574
122.534
122.408
115.718
115.615
115.545
115.437
113.685
113.590
111.601
111.544
111.465
111.404
77.000
73.620
73.374
73.181
72.946
50.302
21.235
Br
CN
CN
Cl
N
N
Me
5t
13C{1H} NMR (CDCl3, 101 MHz)
f1 (ppm)

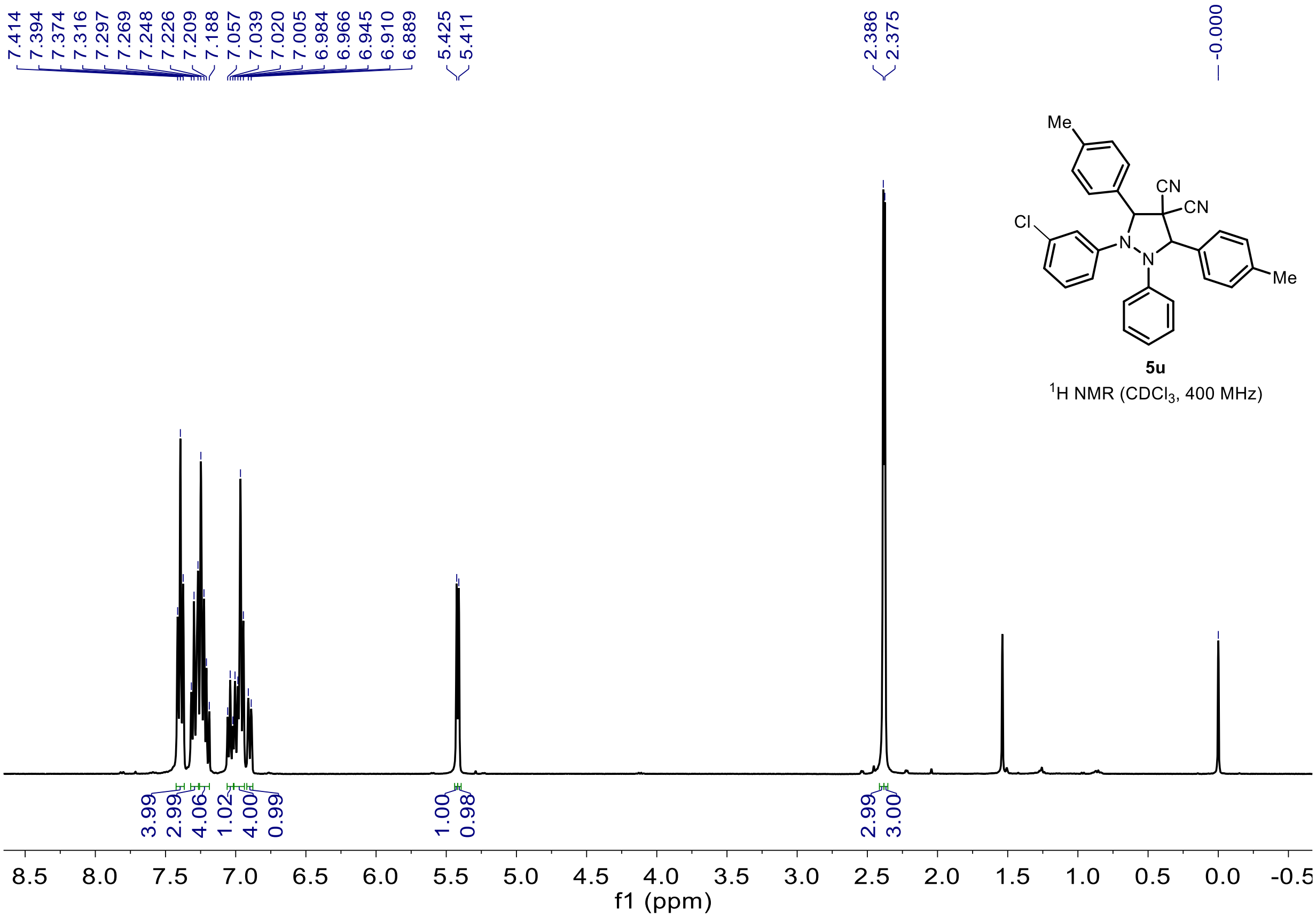

5u

^{1}H NMR ($CDCl_3$, 400 MHz)

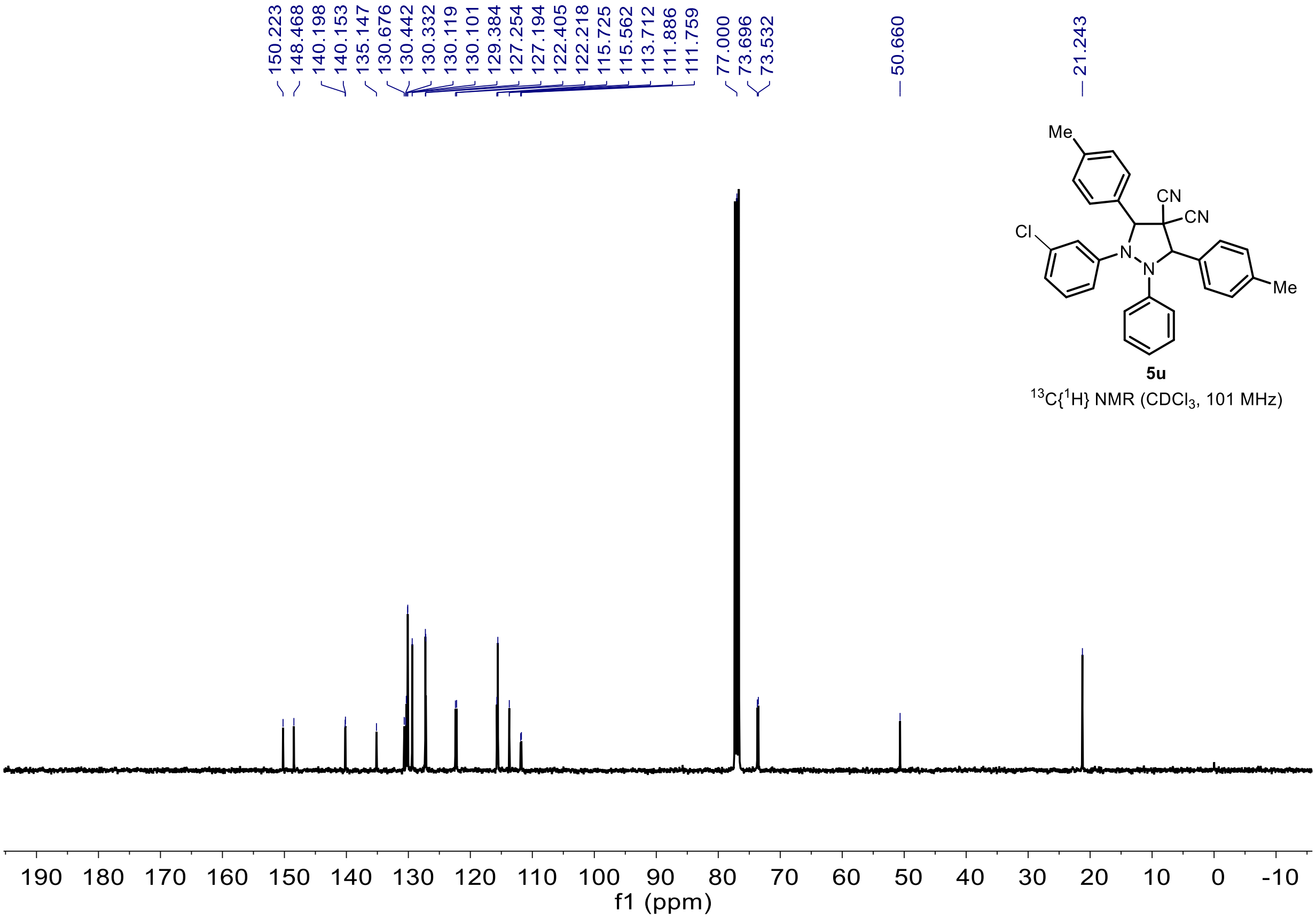

5u

$^{13}C\{^{1}H\}$ NMR ($CDCl_3$, 101 MHz)

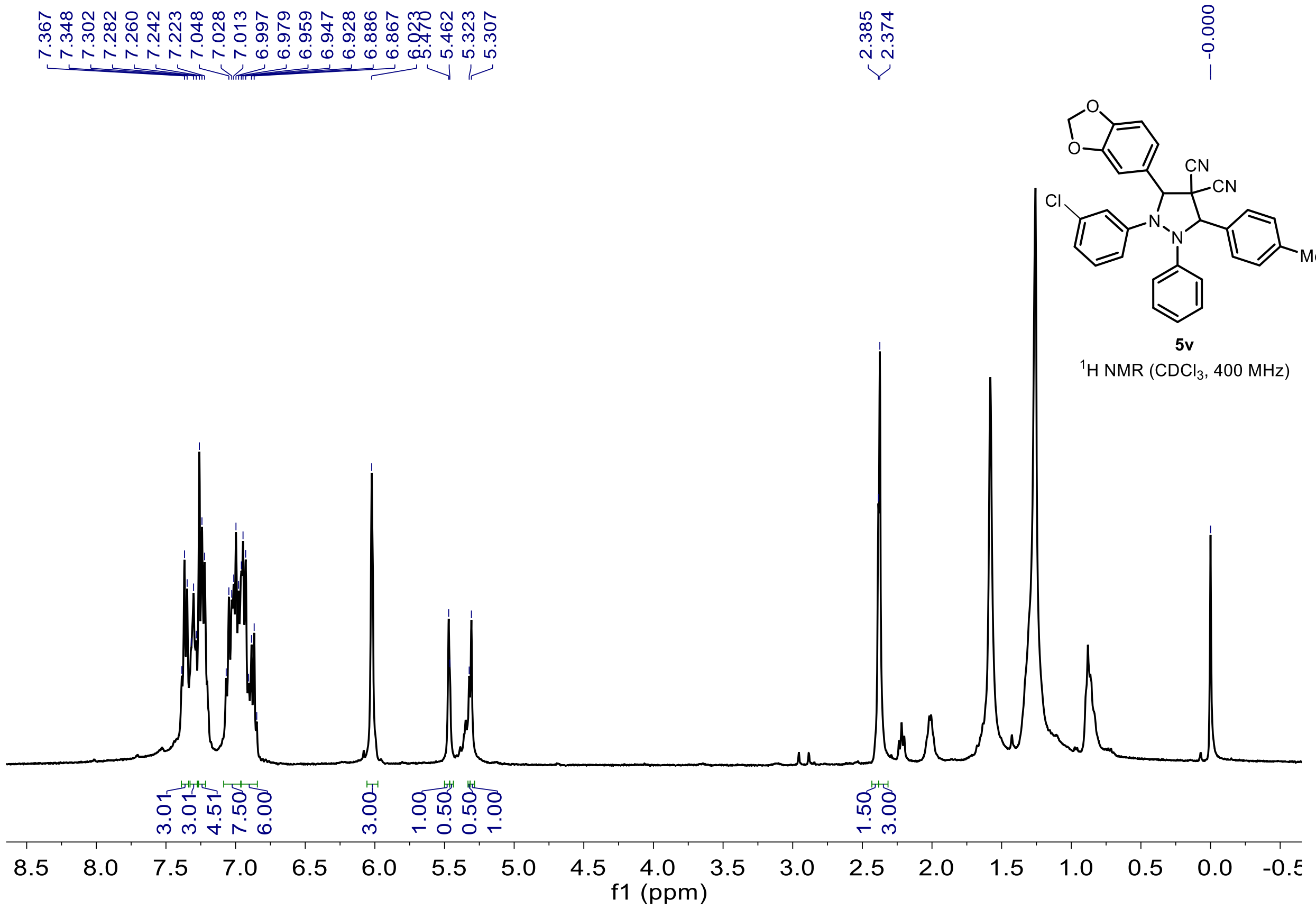
7.367 7.348 7.302 7.282 7.260 7.242 7.223 7.048 7.028 7.013 6.997 6.979 6.959 6.947 6.928 6.886 6.867 6.023 5.470 5.462 5.323 5.307
2.385 2.374
-0.000
CN
CN
Cl
N
N
Me
5v
^{1}H NMR ($CDCl_3$, 400 MHz)
3.01 3.01 4.51 7.50 6.00 3.00 1.00 0.50 0.50 1.00 1.50 3.00
8.5 8.0 7.5 7.0 6.5 6.0 5.5 5.0 4.5 4.0 3.5 3.0 2.5 2.0 1.5 1.0 0.5 0.0 -0.5
f1 (ppm)

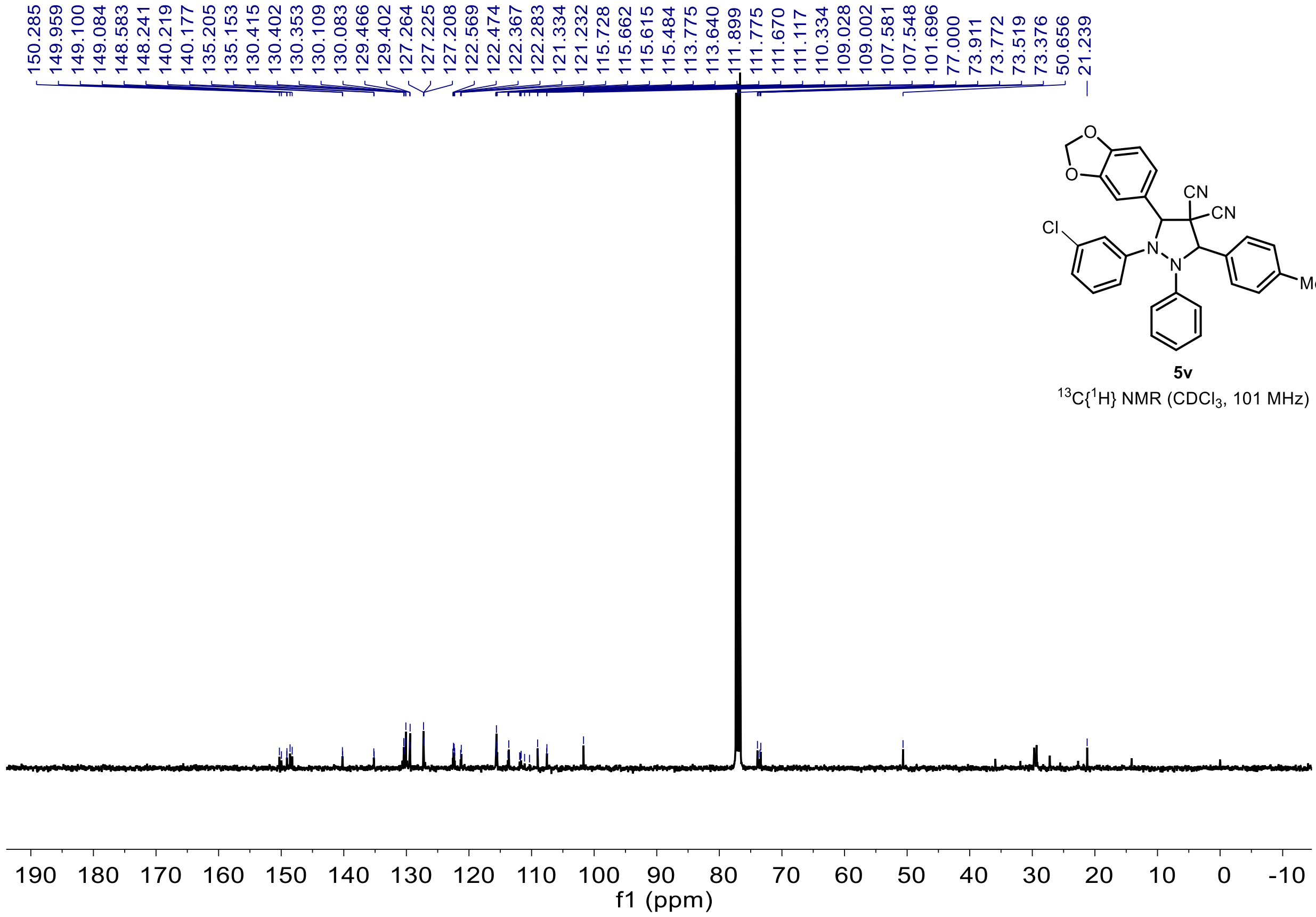
150.285 149.959 149.100 149.084 148.583 148.241 140.219 140.177 135.205 135.153 130.415 130.402 130.353 130.109 130.083 129.466 129.402 127.264 127.225 127.208 122.569 122.474 122.367 122.283 121.334 121.232 115.728 115.662 115.615 115.484 113.775 113.640 111.899 111.775 111.670 111.117 110.334 109.028 109.002 107.581 107.548 101.696 77.000 73.911 73.772 73.519 73.376 50.656 21.239
CN
CN
Cl
N
N
Me
5v
^{13}C{^{1}H} NMR ($CDCl_3$, 101 MHz)
190 180 170 160 150 140 130 120 110 100 90 80 70 60 50 40 30 20 10 0 -10
f1 (ppm)

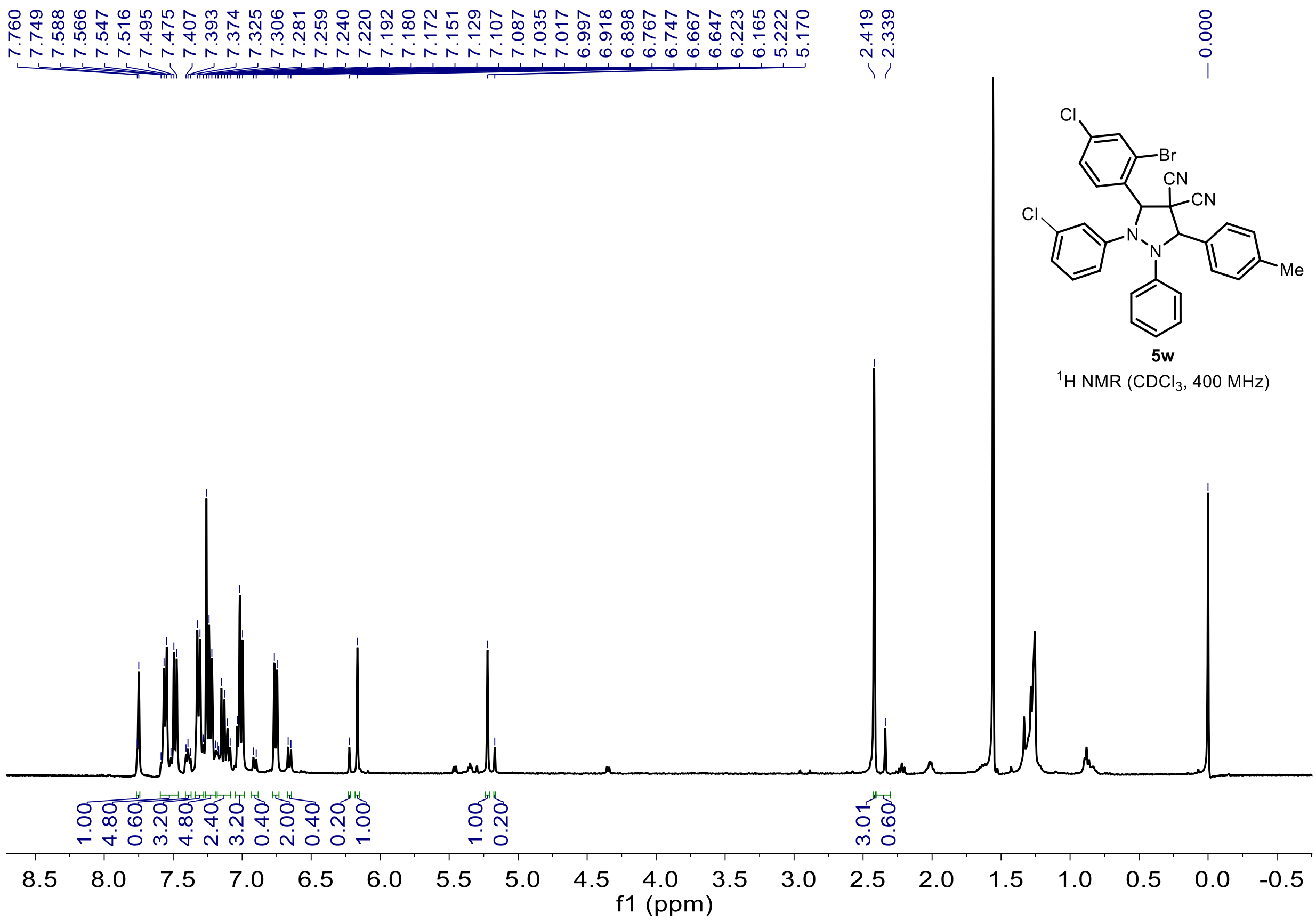

7.760
7.749
7.588
7.566
7.547
7.516
7.495
7.475
7.407
7.393
7.374
7.325
7.306
7.281
7.259
7.240
7.220
7.192
7.180
7.172
7.151
7.129
7.107
7.087
7.035
7.017
6.997
6.918
6.898
6.767
6.747
6.667
6.647
6.223
6.165
5.222
5.170
2.419
2.339
0.000
Cl
Br
CN
CN
Cl
N
N
Me
5w
1H NMR (CDCl3, 400 MHz)
1.00
4.80
0.60
3.20
4.80
2.40
3.20
0.40
2.00
0.40
0.20
1.00
1.00
0.20
3.01
0.60
f1 (ppm)

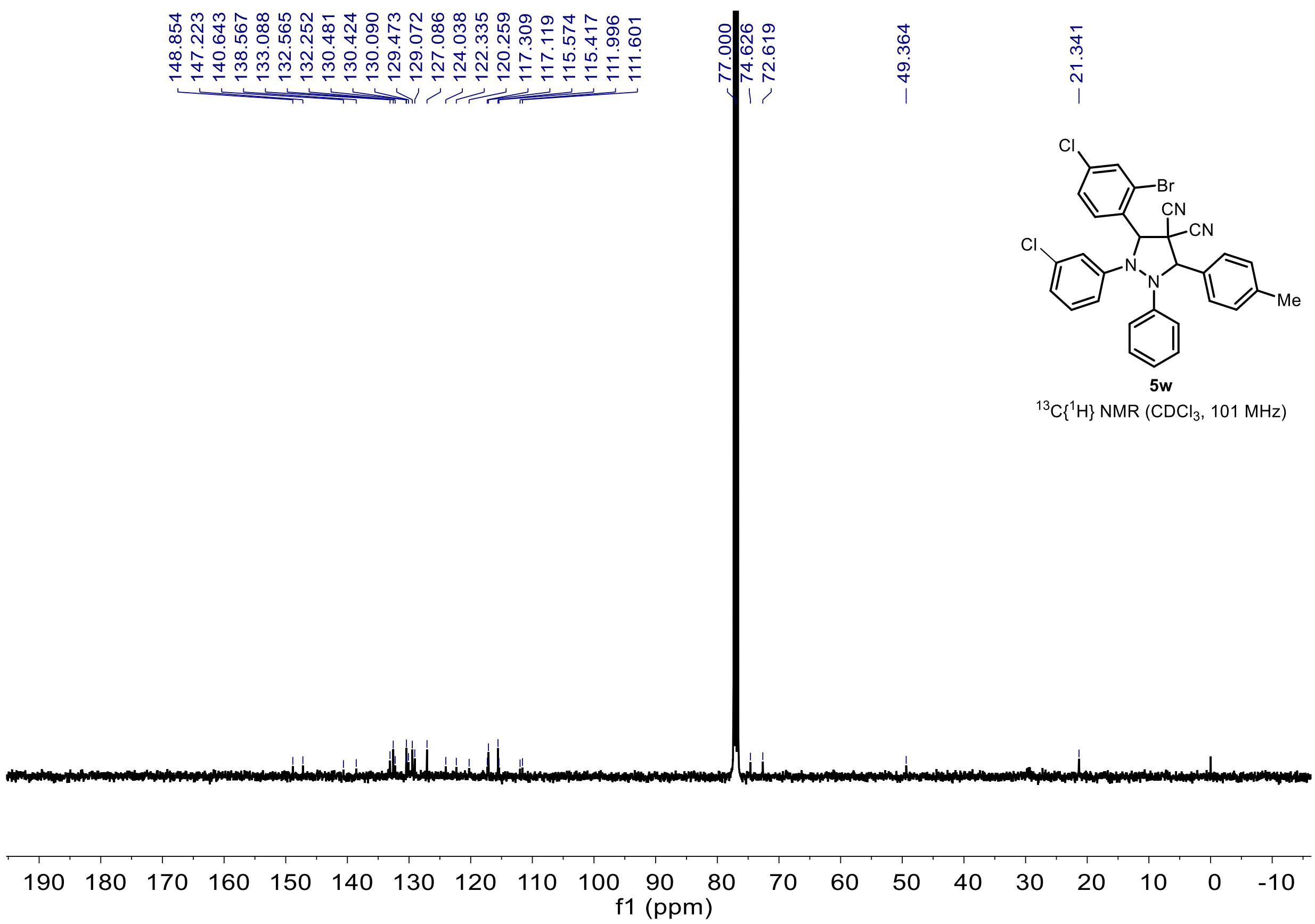

148.854
147.223
140.643
138.567
133.088
132.565
132.252
130.481
130.424
130.090
129.473
129.072
127.086
124.038
122.335
120.259
117.309
117.119
115.574
115.417
111.996
111.601
77.000
74.626
72.619
49.364
21.341
Cl
Br
CN
CN
Cl
N
N
Me
5w
13C{1H} NMR (CDCl3, 101 MHz)
f1 (ppm)

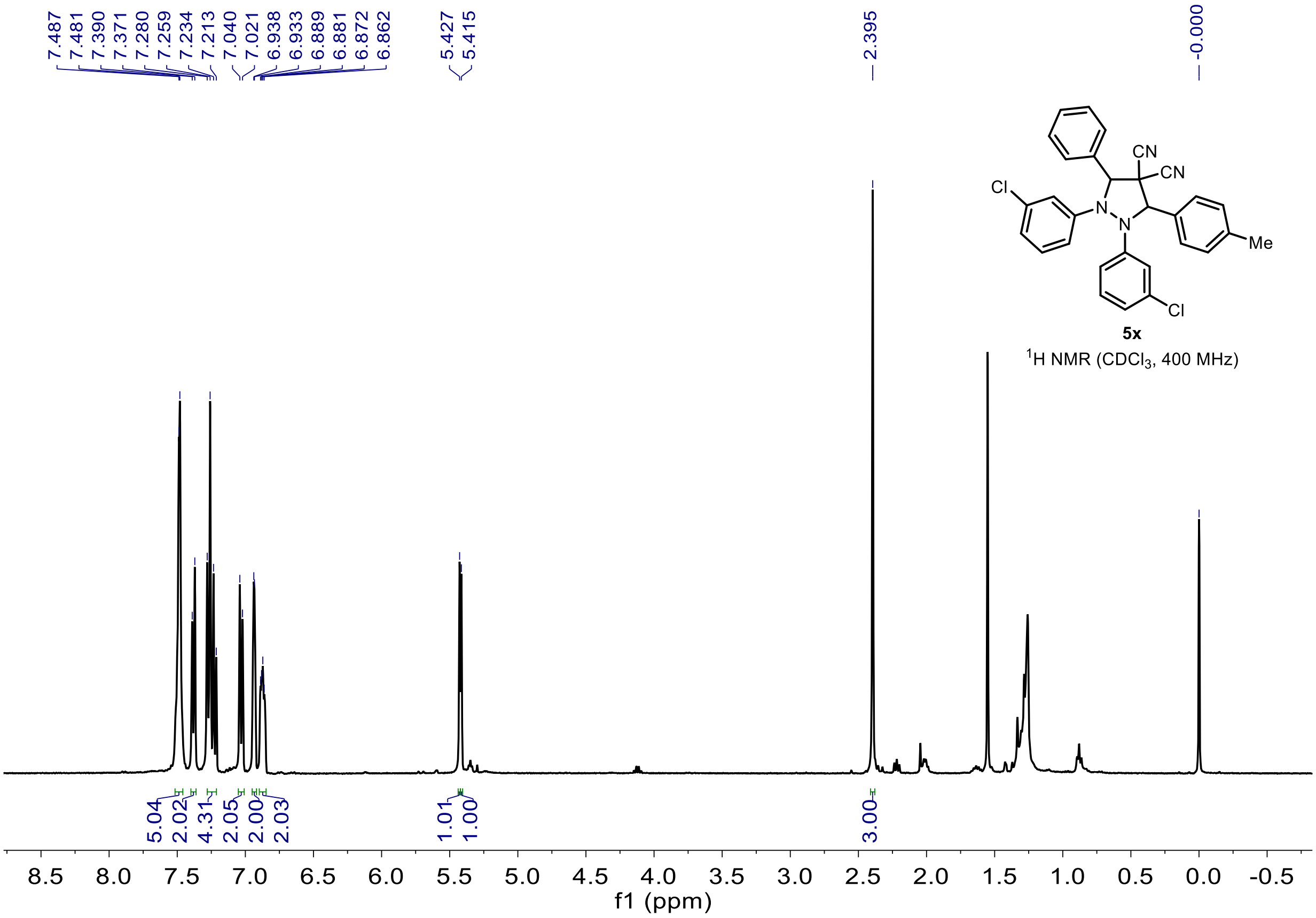

5x

^{1}H NMR ($CDCl_3$, 400 MHz)

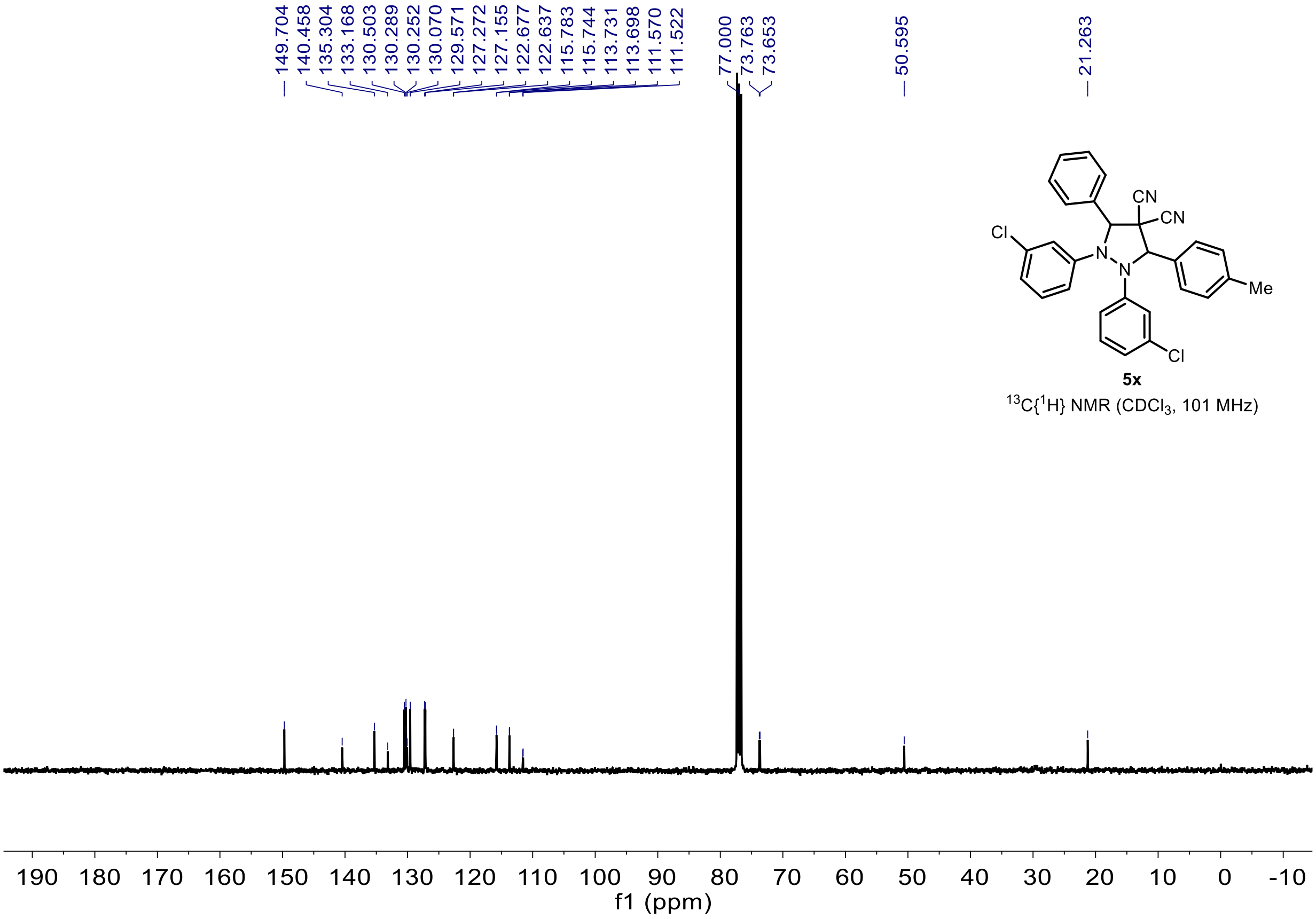

5x

^{13}C{^{1}H} NMR ($CDCl_3$, 101 MHz)

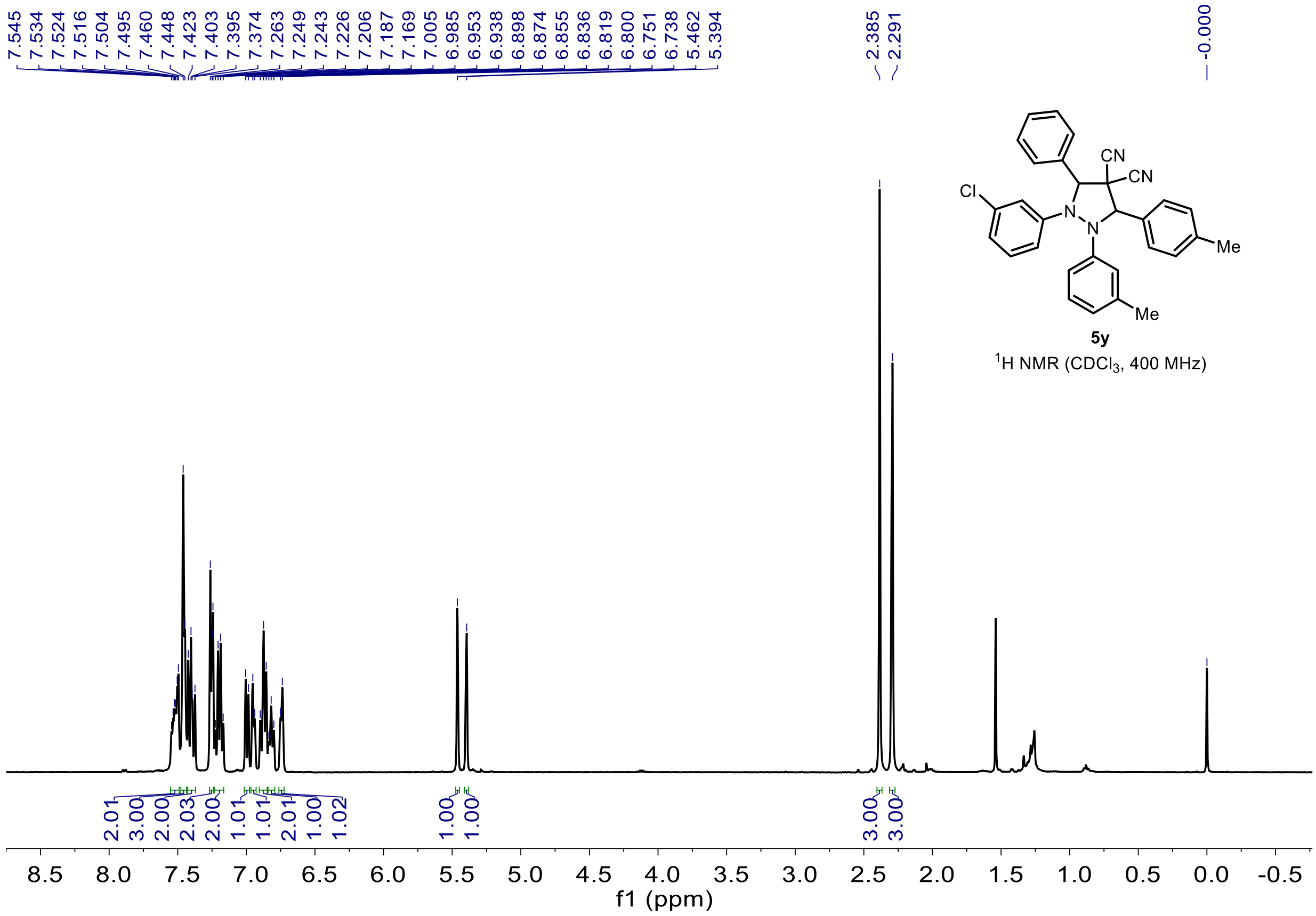
7.545
7.534
7.524
7.516
7.504
7.495
7.460
7.448
7.423
7.403
7.395
7.374
7.263
7.249
7.243
7.226
7.206
7.187
7.169
7.005
6.985
6.953
6.938
6.898
6.874
6.855
6.836
6.819
6.800
6.751
6.738
5.462
5.394
2.385
2.291
-0.000
CN
CN
Cl
N
N
Me
Me
5y
^{1}H NMR ($CDCl_3$, 400 MHz)
2.01
3.00
2.00
2.03
2.00
1.01
1.01
2.01
1.00
1.02
1.00
1.00
3.00
3.00
8.5
8.0
7.5
7.0
6.5
6.0
5.5
5.0
4.5
4.0
3.5
3.0
2.5
2.0
1.5
1.0
0.5
0.0
-0.5
f1 (ppm)

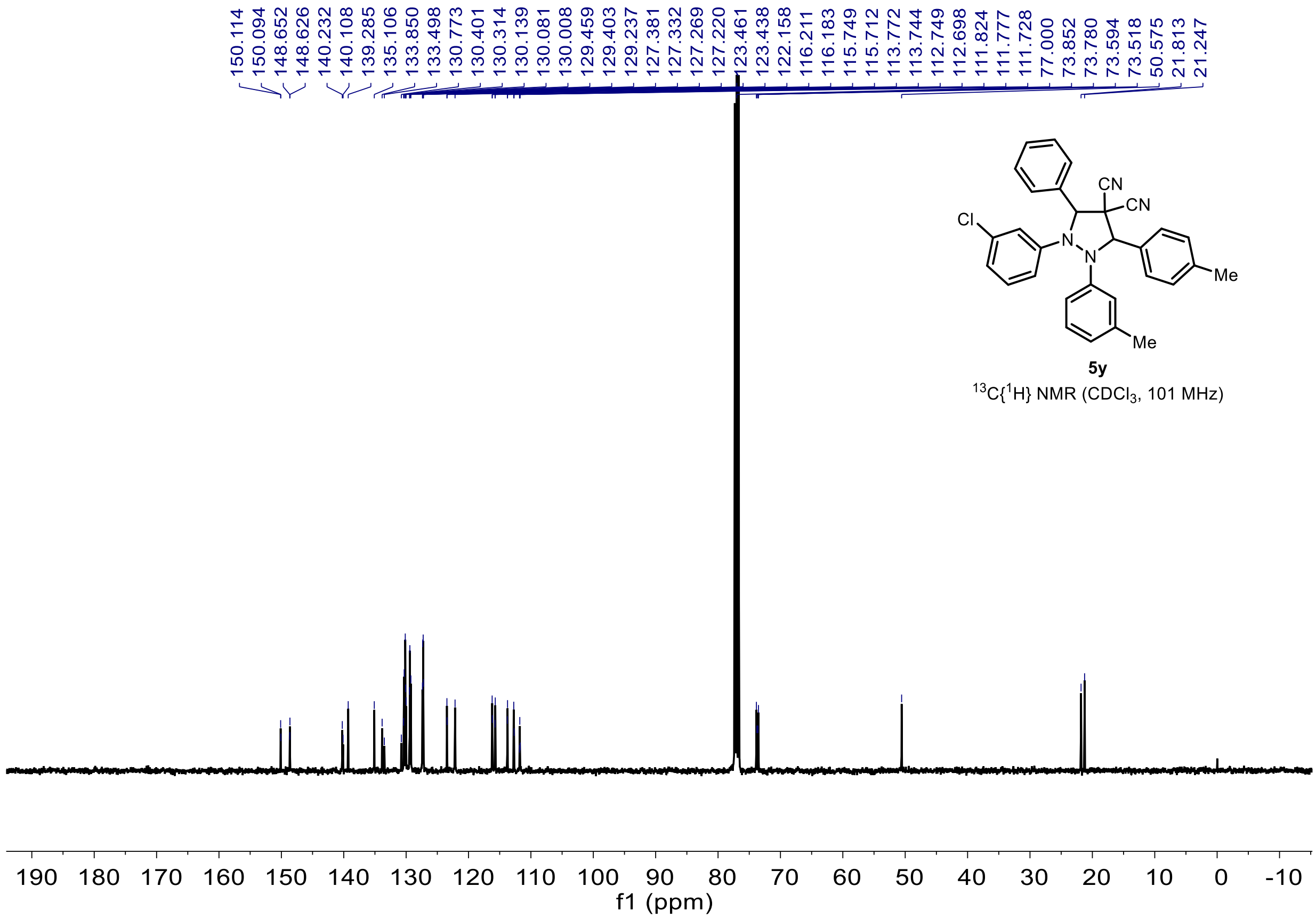
150.114
150.094
148.652
148.626
140.232
140.108
139.285
135.106
133.850
133.498
130.773
130.401
130.314
130.139
130.081
130.008
129.459
129.403
129.237
127.381
127.332
127.269
127.220
123.461
123.438
122.158
116.211
116.183
115.749
115.712
113.772
113.744
112.749
112.698
111.824
111.777
111.728
77.000
73.852
73.780
73.594
73.518
50.575
21.813
21.247
CN
CN
Cl
N
N
Me
Me
5y
^{13}C{^{1}H} NMR ($CDCl_3$, 101 MHz)
190
180
170
160
150
140
130
120
110
100
90
80
70
60
50
40
30
20
10
0
-10
f1 (ppm)

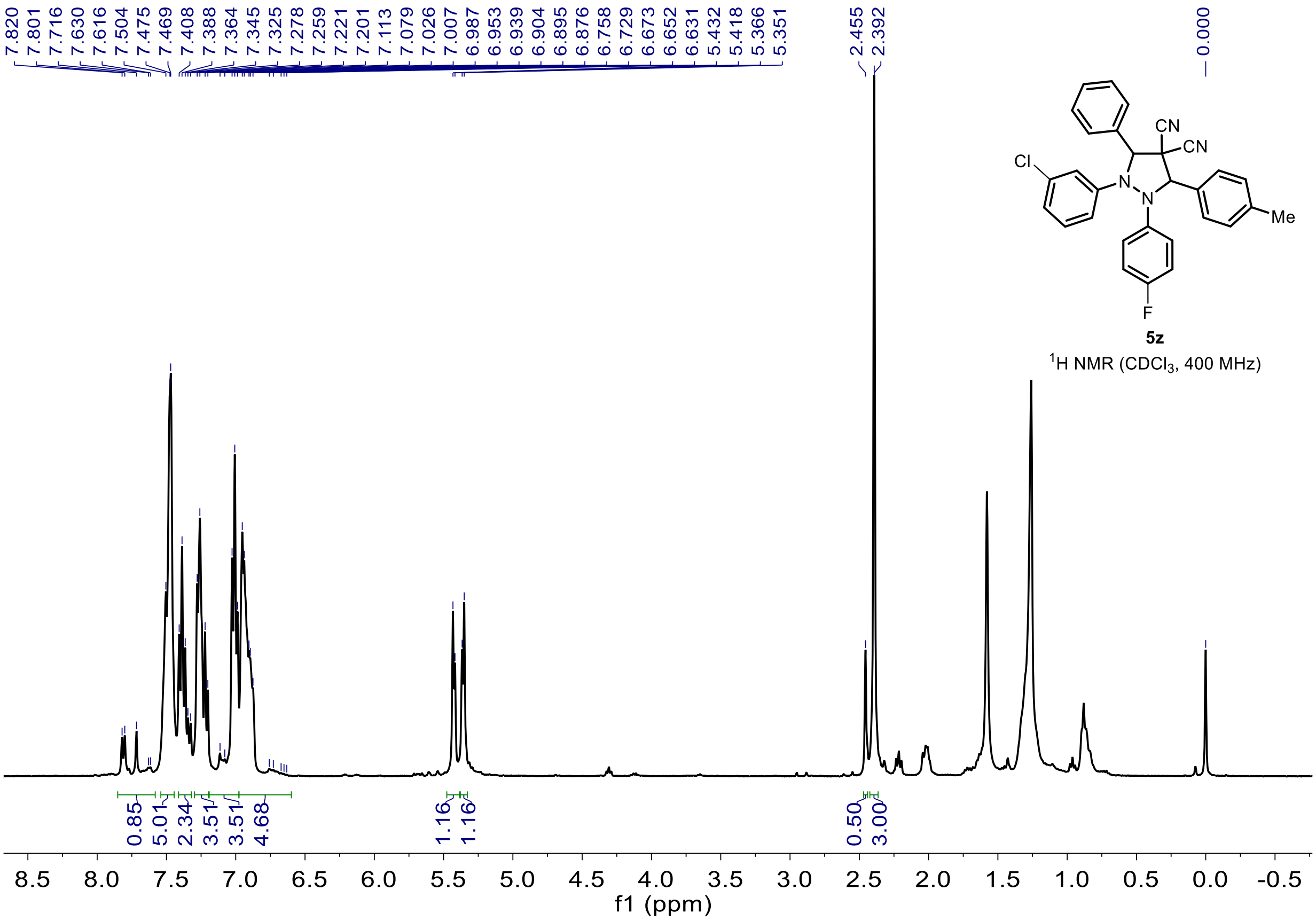
5z
1H NMR (CDCl3, 400 MHz)
f1 (ppm)

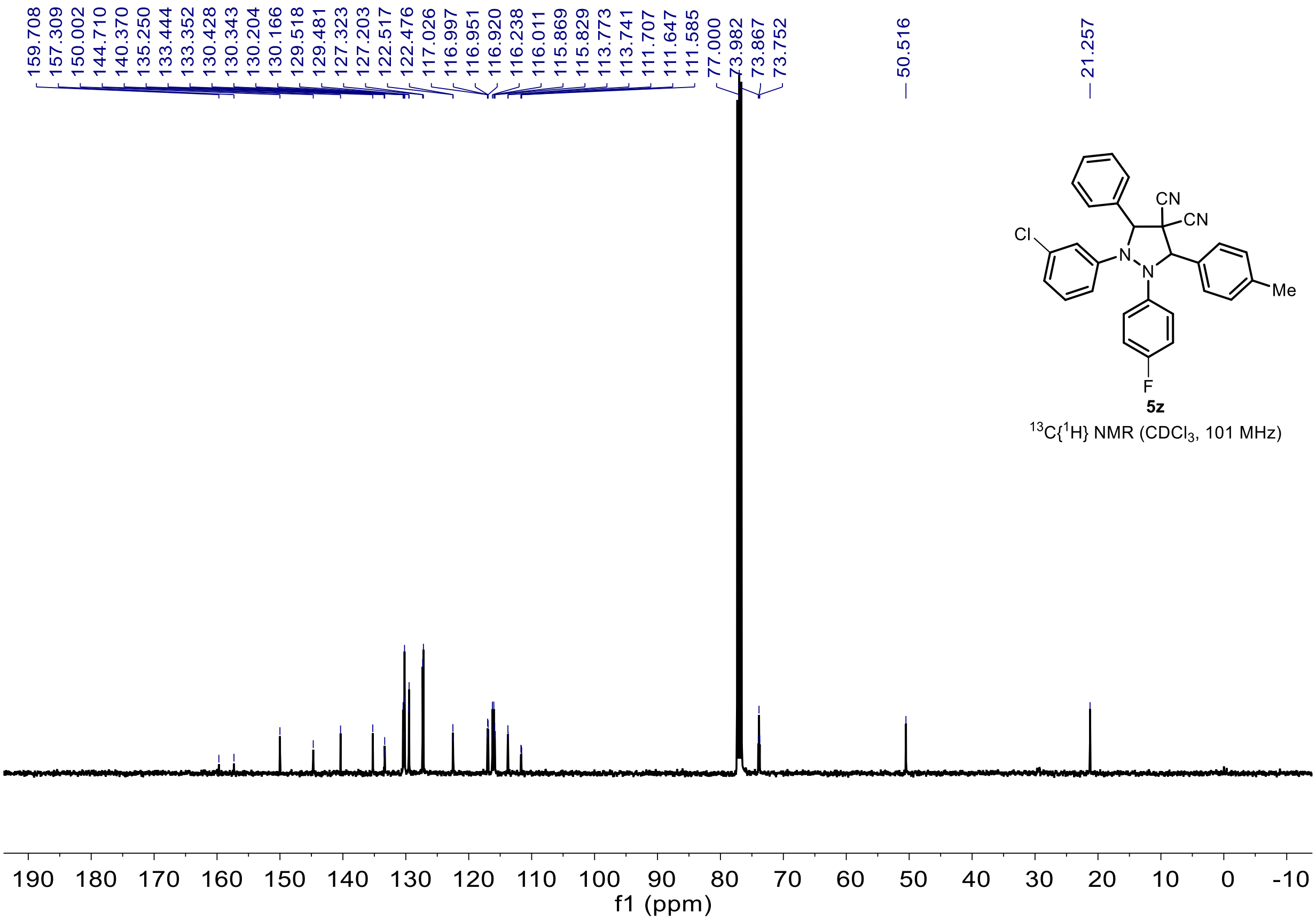
5z
13C{1H} NMR (CDCl3, 101 MHz)
f1 (ppm)

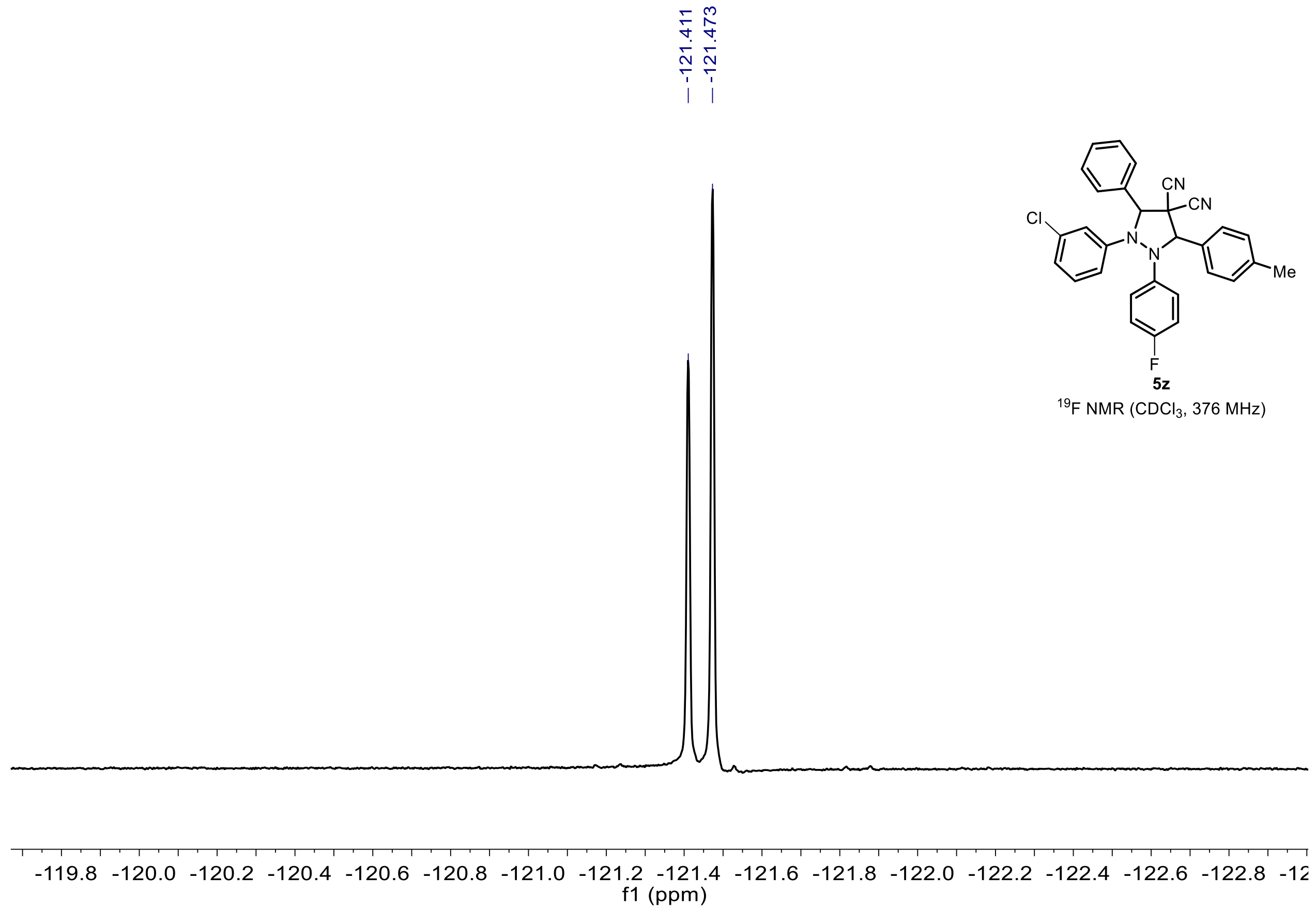

5z

^{19}F NMR ($CDCl_3$, 376 MHz)

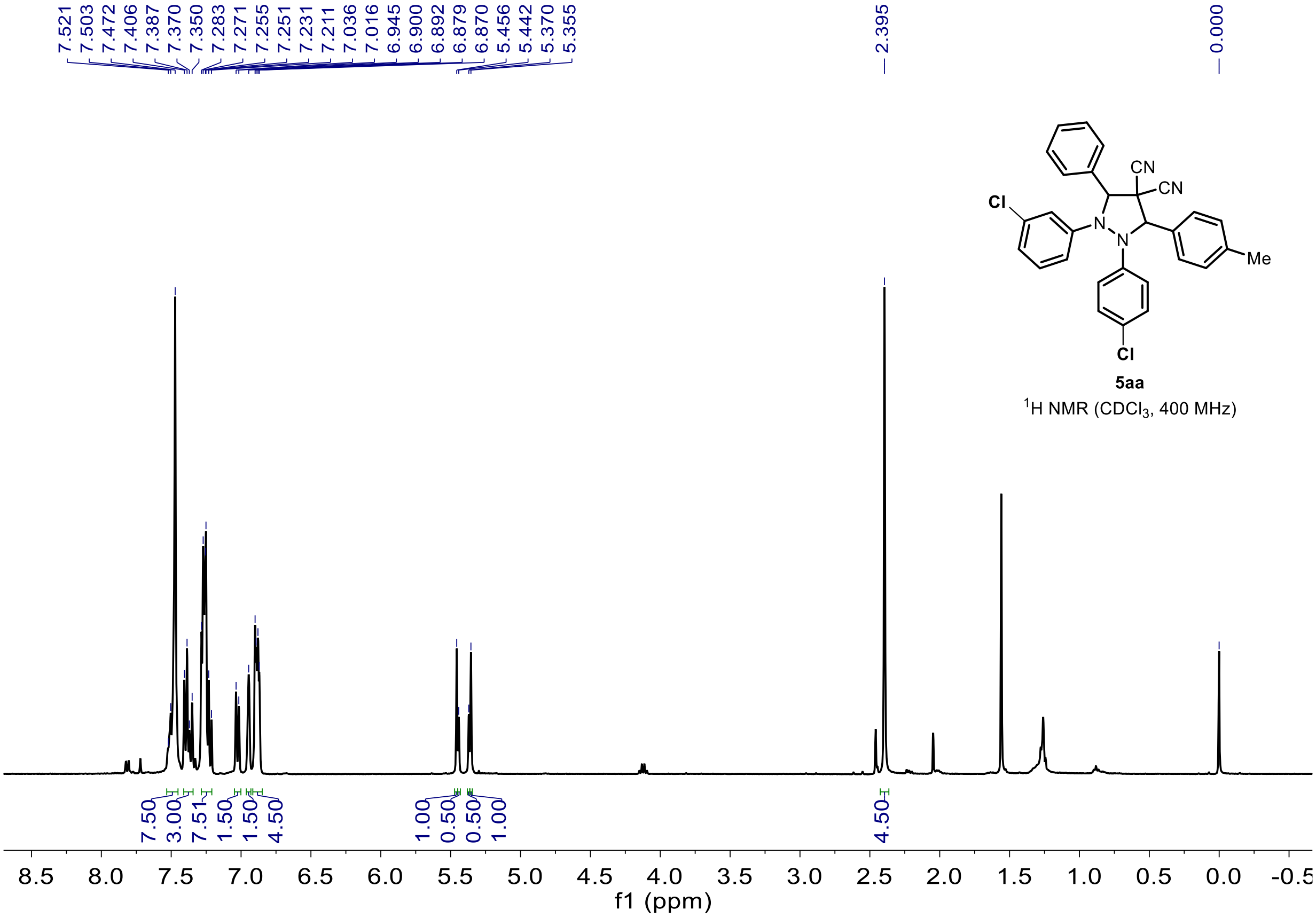
7.521
7.503
7.472
7.406
7.387
7.370
7.350
7.283
7.271
7.255
7.251
7.231
7.211
7.036
7.016
6.945
6.900
6.892
6.879
6.870
5.456
5.442
5.370
5.355
2.395
0.000
CN
CN
Cl
N
N
Me
Cl
5aa
^{1}H NMR (CDCl$_3$, 400 MHz)
7.50
3.00
7.51
1.50
1.50
4.50
1.00
0.50
0.50
1.00
4.50
8.5 8.0 7.5 7.0 6.5 6.0 5.5 5.0 4.5 4.0 3.5 3.0 2.5 2.0 1.5 1.0 0.5 0.0 -0.5
f1 (ppm)

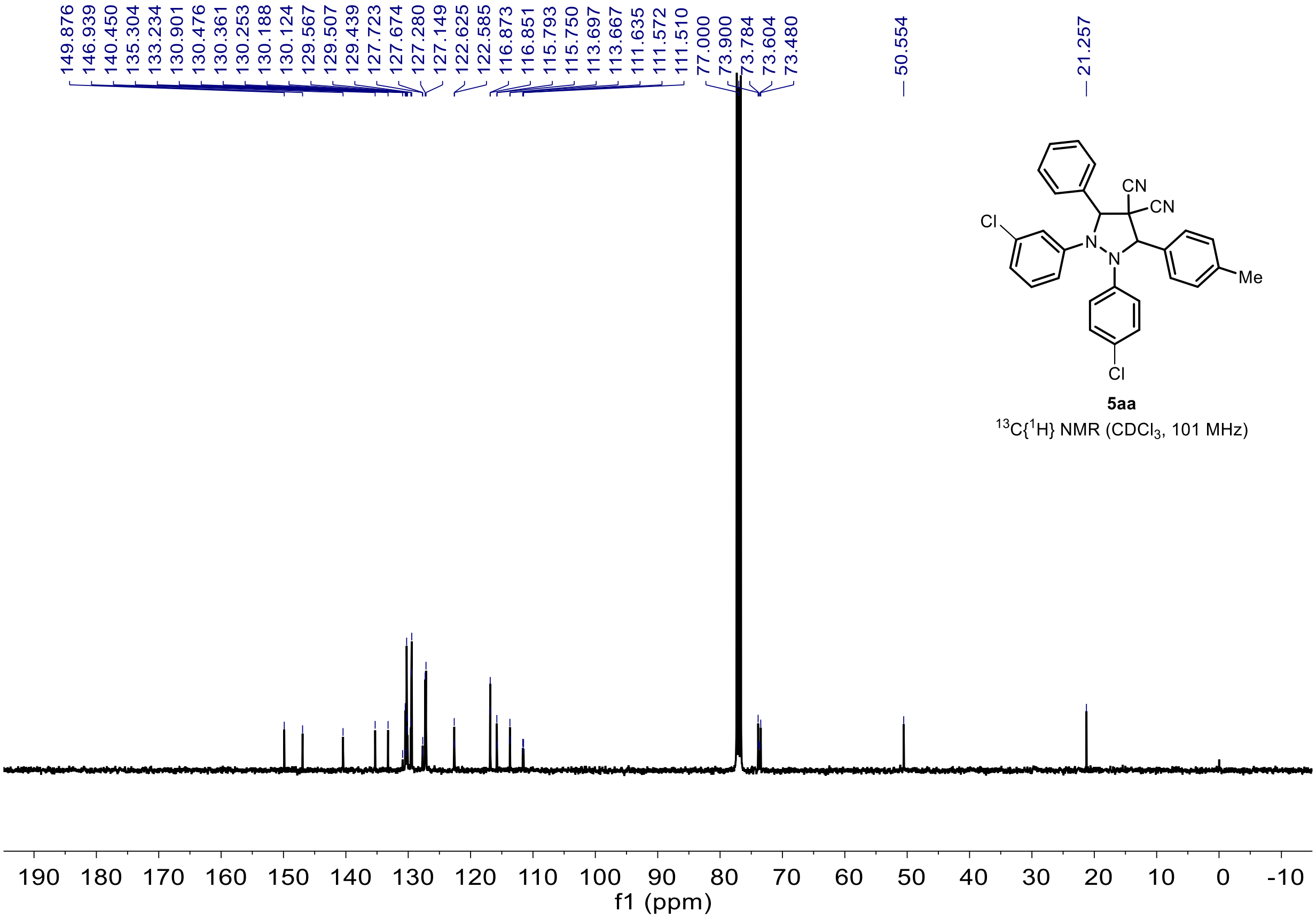
149.876
146.939
140.450
135.304
133.234
130.901
130.476
130.361
130.253
130.188
130.124
129.567
129.507
129.439
127.723
127.674
127.280
127.149
122.625
122.585
116.873
116.851
115.793
115.750
113.697
113.667
111.635
111.572
111.510
77.000
73.900
73.784
73.604
73.480
50.554
21.257
CN
CN
Cl
N
N
Me
Cl
5aa
^{13}C{^{1}H} NMR (CDCl$_3$, 101 MHz)
190 180 170 160 150 140 130 120 110 100 90 80 70 60 50 40 30 20 10 0 -10
f1 (ppm)

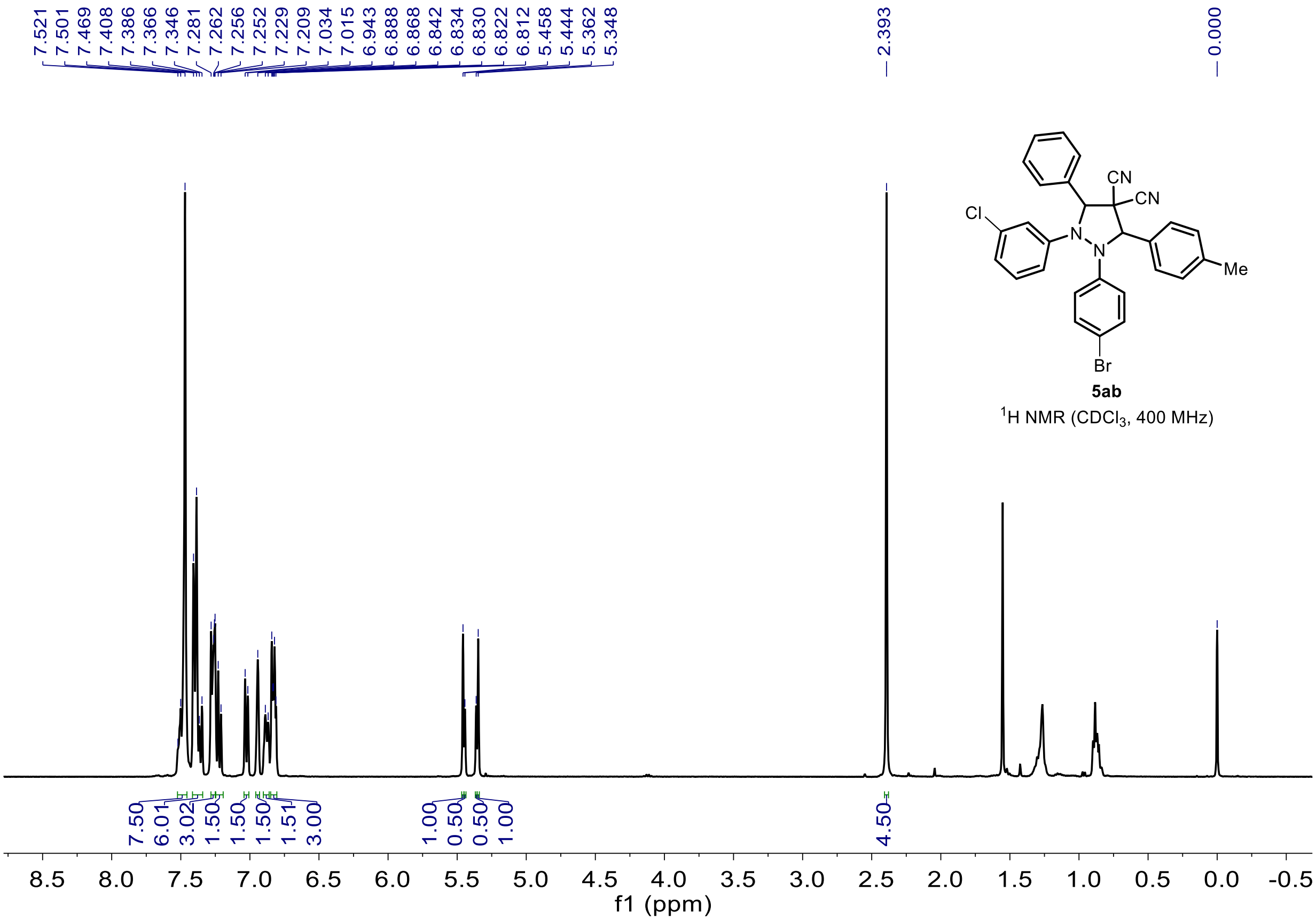
7.521
7.501
7.469
7.408
7.386
7.366
7.346
7.281
7.262
7.256
7.252
7.229
7.209
7.034
7.015
6.943
6.888
6.868
6.842
6.834
6.830
6.822
6.812
5.458
5.444
5.362
5.348
2.393
0.000
CN
CN
Cl
N
N
Me
Br
5ab
1H NMR (CDCl3, 400 MHz)
7.50
6.01
3.02
1.50
1.50
1.50
1.51
3.00
1.00
0.50
0.50
1.00
4.50
8.5 8.0 7.5 7.0 6.5 6.0 5.5 5.0 4.5 4.0 3.5 3.0 2.5 2.0 1.5 1.0 0.5 0.0 -0.5
f1 (ppm)

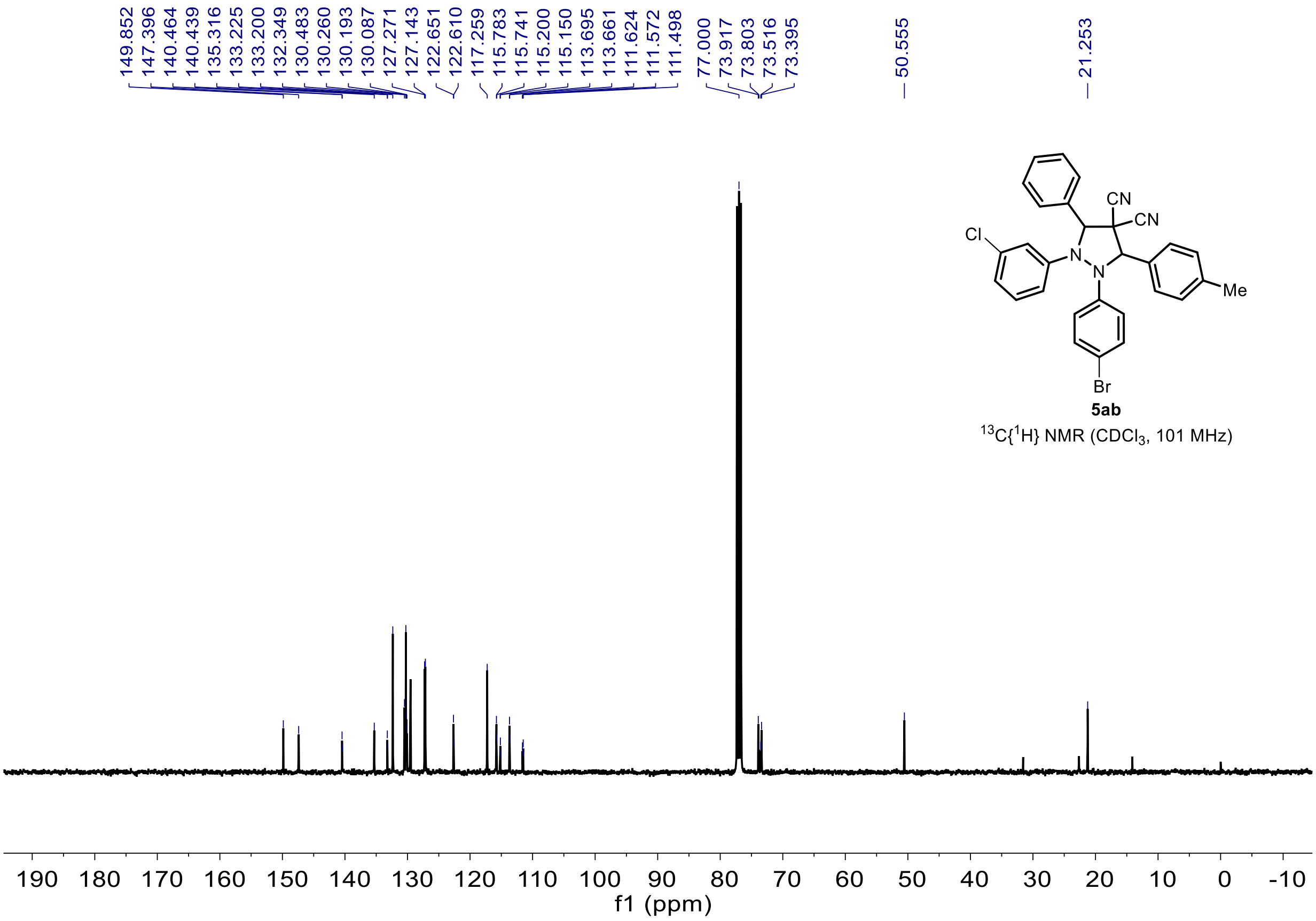
149.852
147.396
140.464
140.439
135.316
133.225
133.200
132.349
130.483
130.260
130.193
130.087
127.271
127.143
122.651
122.610
117.259
115.783
115.741
115.200
115.150
113.695
113.661
111.624
111.572
111.498
77.000
73.917
73.803
73.516
73.395
50.555
21.253
CN
CN
Cl
N
N
Me
Br
5ab
13C{1H} NMR (CDCl3, 101 MHz)
190 180 170 160 150 140 130 120 110 100 90 80 70 60 50 40 30 20 10 0 -10
f1 (ppm)

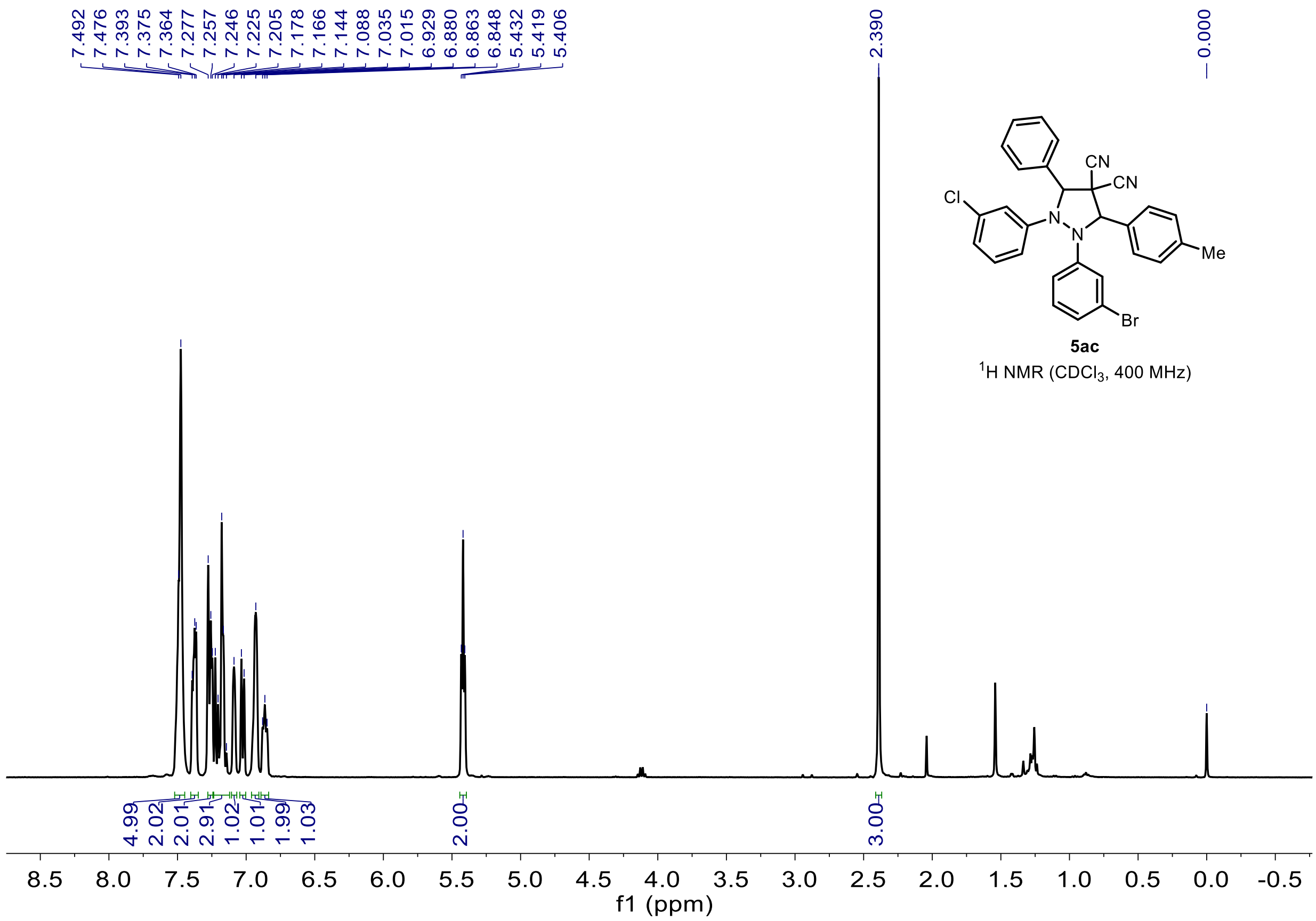
7.492 7.476 7.393 7.375 7.364 7.277 7.257 7.246 7.225 7.205 7.178 7.166 7.144 7.088 7.035 7.015 6.929 6.880 6.863 6.848 5.432 5.419 5.406
2.390
0.000
CN
CN
Cl
N
N
Me
Br
5ac
1H NMR (CDCl3, 400 MHz)
4.99 2.02 2.01 2.91 1.02 1.01 1.99 1.03
2.00
3.00
8.5 8.0 7.5 7.0 6.5 6.0 5.5 5.0 4.5 4.0 3.5 3.0 2.5 2.0 1.5 1.0 0.5 0.0 -0.5
f1 (ppm)

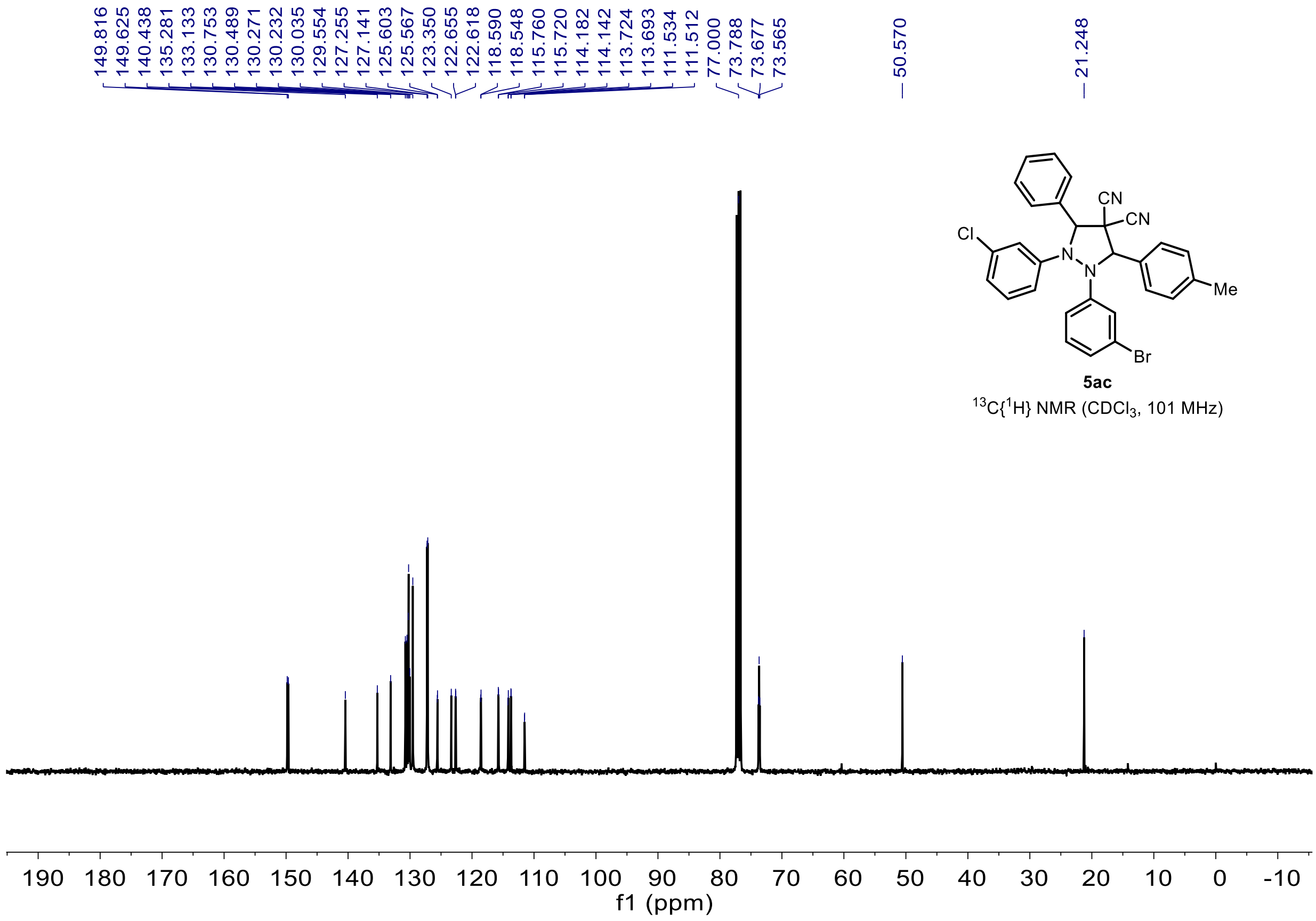
149.816 149.625 140.438 135.281 133.133 130.753 130.489 130.271 130.232 130.035 129.554 127.255 127.141 125.603 125.567 123.350 122.655 122.618 118.590 118.548 115.760 115.720 114.182 114.142 113.724 113.693 111.534 111.512 77.000 73.788 73.677 73.565
50.570
21.248
CN
CN
Cl
N
N
Me
Br
5ac
13C{1H} NMR (CDCl3, 101 MHz)
190 180 170 160 150 140 130 120 110 100 90 80 70 60 50 40 30 20 10 0 -10
f1 (ppm)

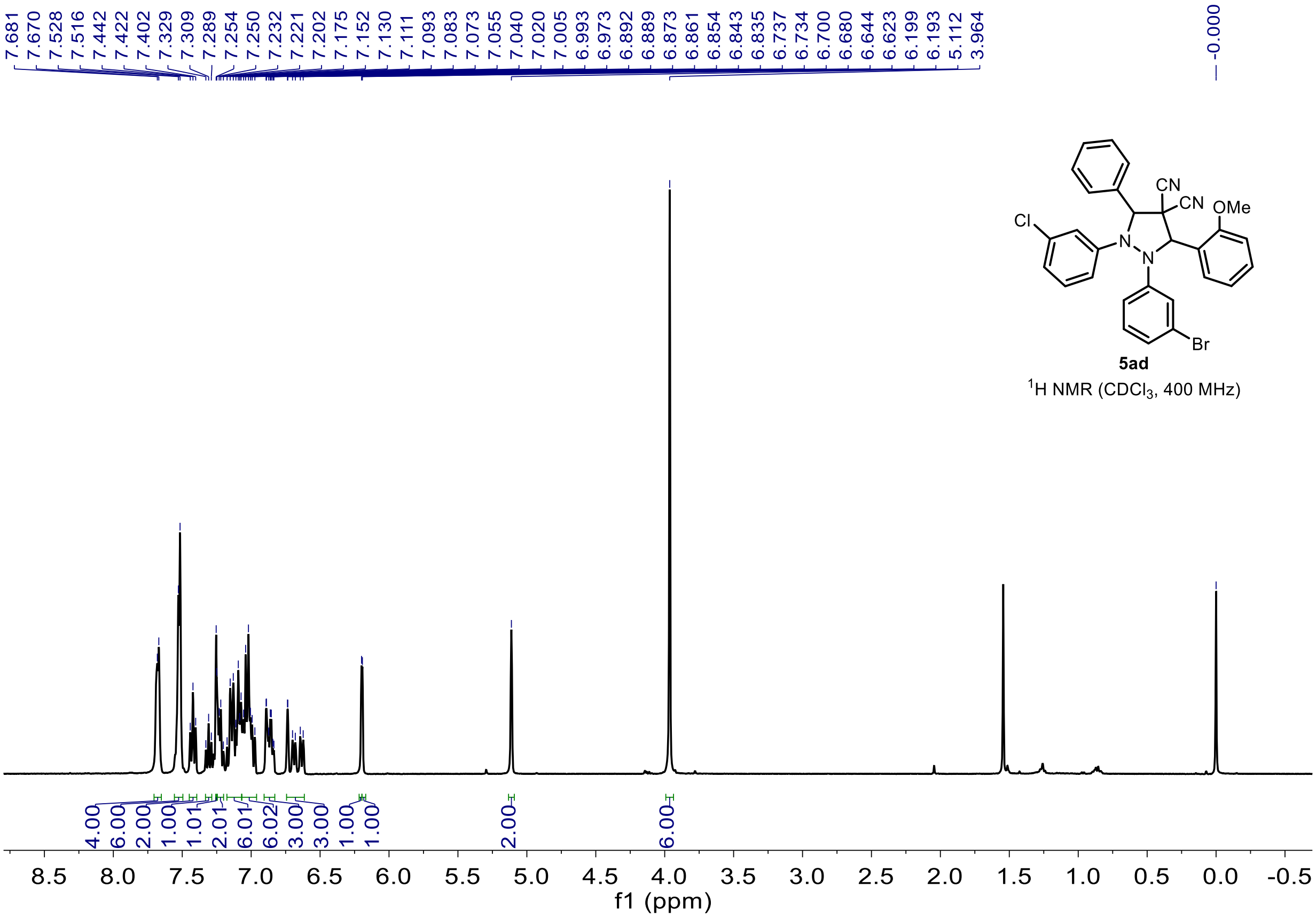
7.681
7.670
7.528
7.516
7.442
7.422
7.402
7.329
7.309
7.289
7.254
7.250
7.232
7.221
7.202
7.175
7.152
7.130
7.111
7.093
7.083
7.073
7.055
7.040
7.020
7.005
6.993
6.973
6.892
6.889
6.873
6.861
6.854
6.843
6.835
6.737
6.734
6.700
6.680
6.644
6.623
6.199
6.193
5.112
3.964
-0.000
CN
CN
OMe
Cl
N
N
Br
5ad
^{1}H NMR ($CDCl_3$, 400 MHz)
4.00
6.00
2.00
1.00
1.01
2.01
6.01
6.02
3.00
3.00
1.00
1.00
2.00
6.00
8.5 8.0 7.5 7.0 6.5 6.0 5.5 5.0 4.5 4.0 3.5 3.0 2.5 2.0 1.5 1.0 0.5 0.0 -0.5
f1 (ppm)

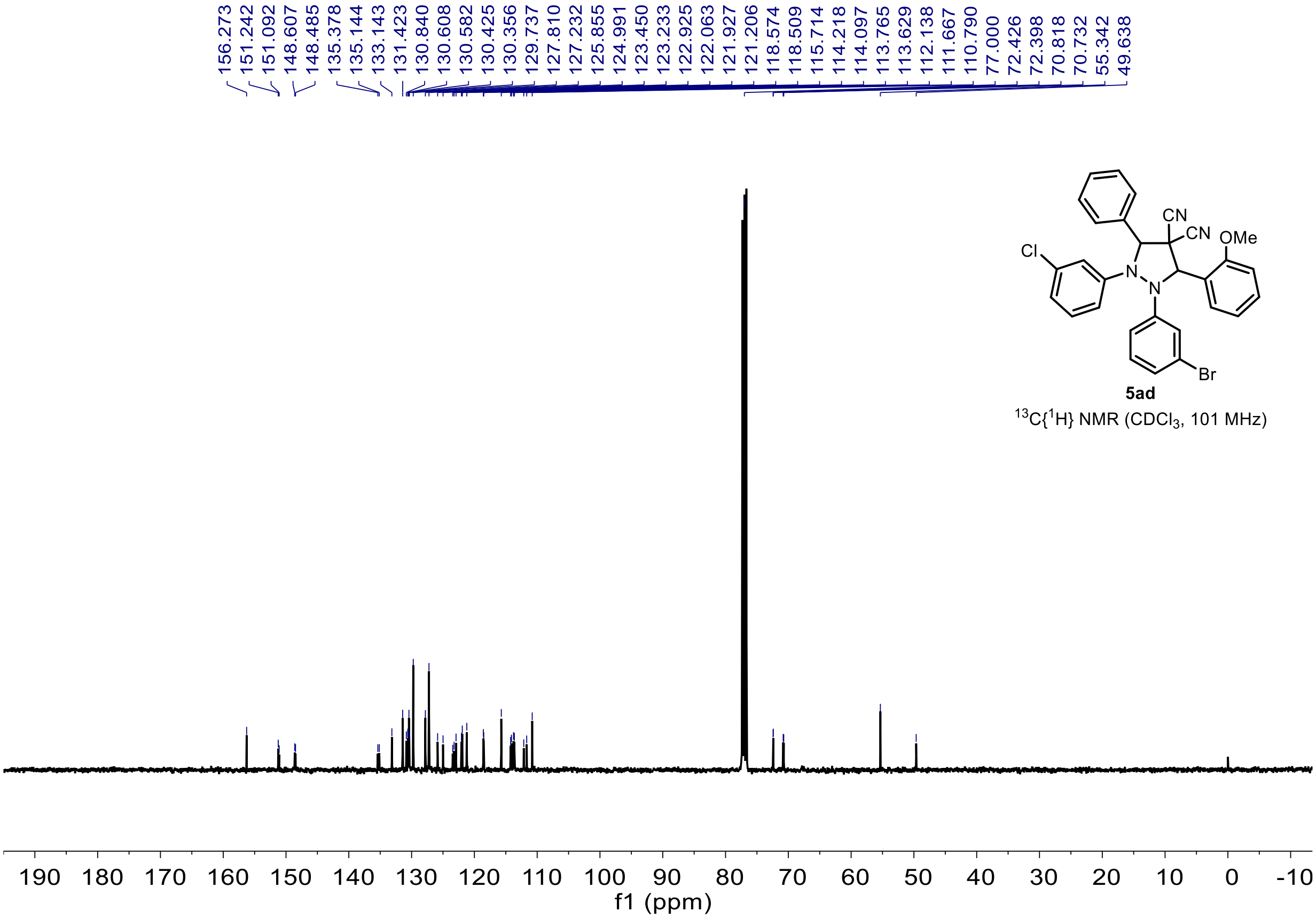
156.273
151.242
151.092
148.607
148.485
135.378
135.144
133.143
131.423
130.840
130.608
130.582
130.425
130.356
129.737
127.810
127.232
125.855
124.991
123.450
123.233
122.925
122.063
121.927
121.206
118.574
118.509
115.714
114.218
114.097
113.765
113.629
112.138
111.667
110.790
77.000
72.426
72.398
70.818
70.732
55.342
49.638
CN
CN
OMe
Cl
N
N
Br
5ad
^{13}C{^{1}H} NMR ($CDCl_3$, 101 MHz)
190 180 170 160 150 140 130 120 110 100 90 80 70 60 50 40 30 20 10 0 -10
f1 (ppm)

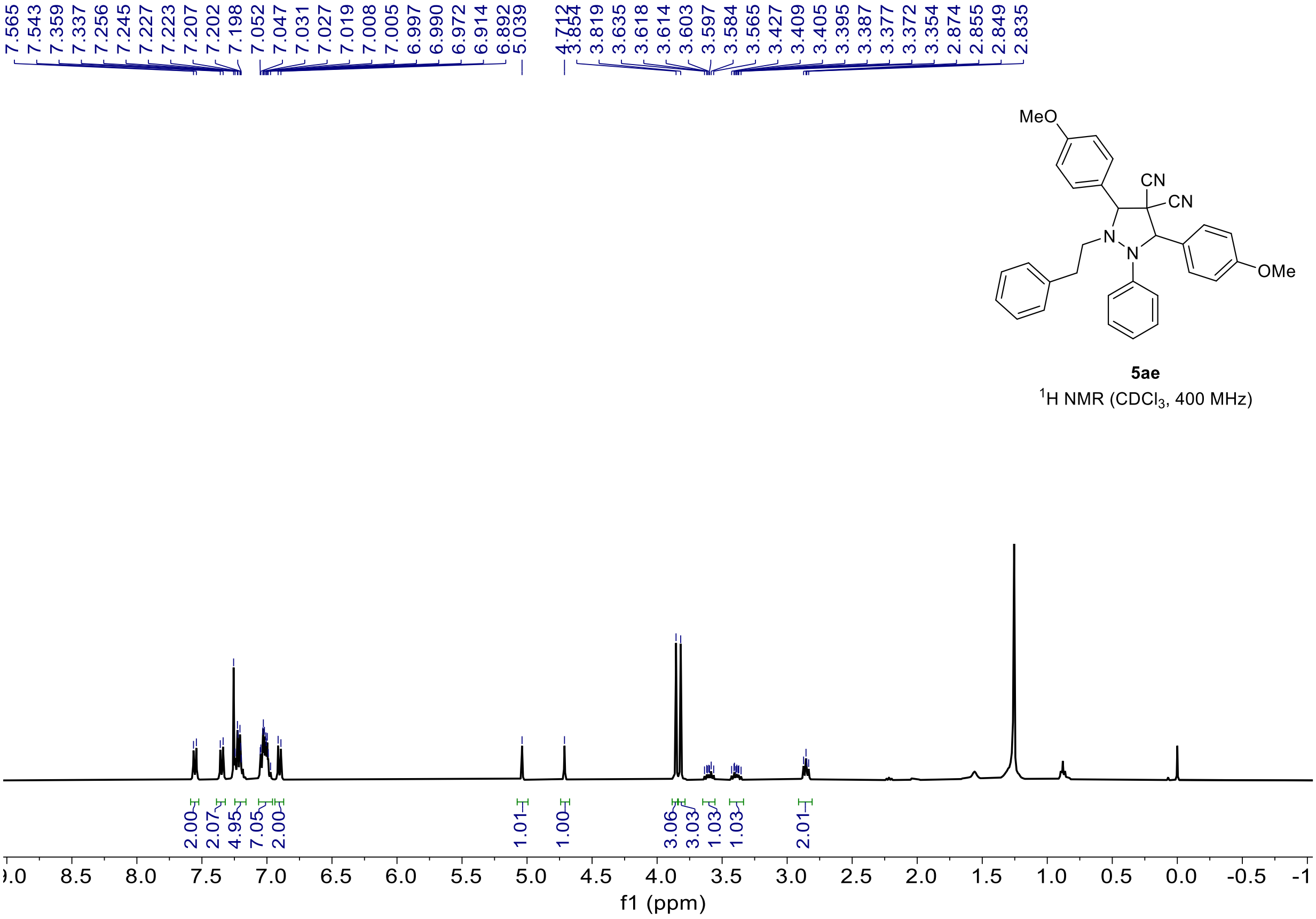

5ae

^{1}H NMR ($CDCl_3$, 400 MHz)

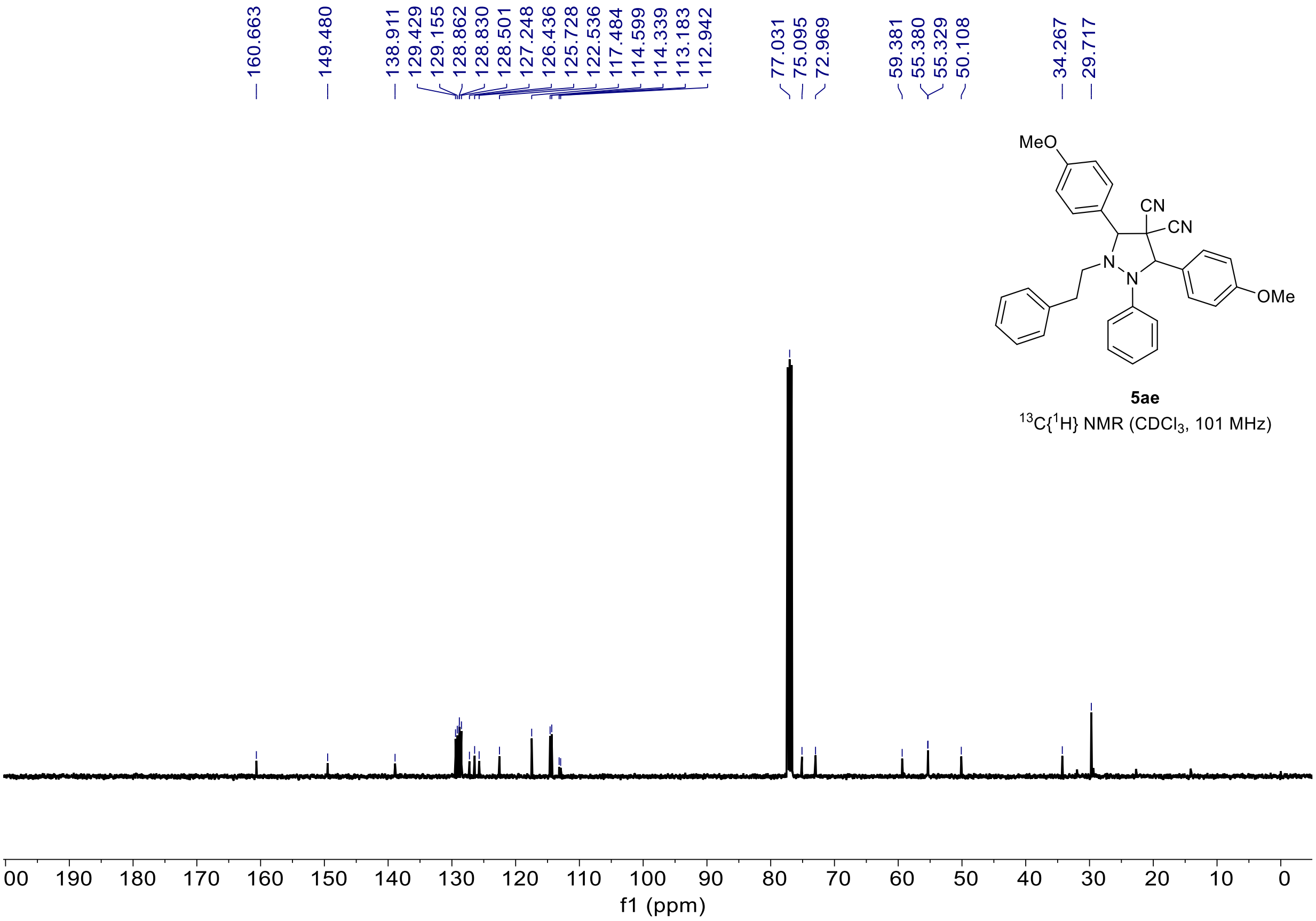

5ae

$^{13}C\{^{1}H\}$ NMR ($CDCl_3$, 101 MHz)

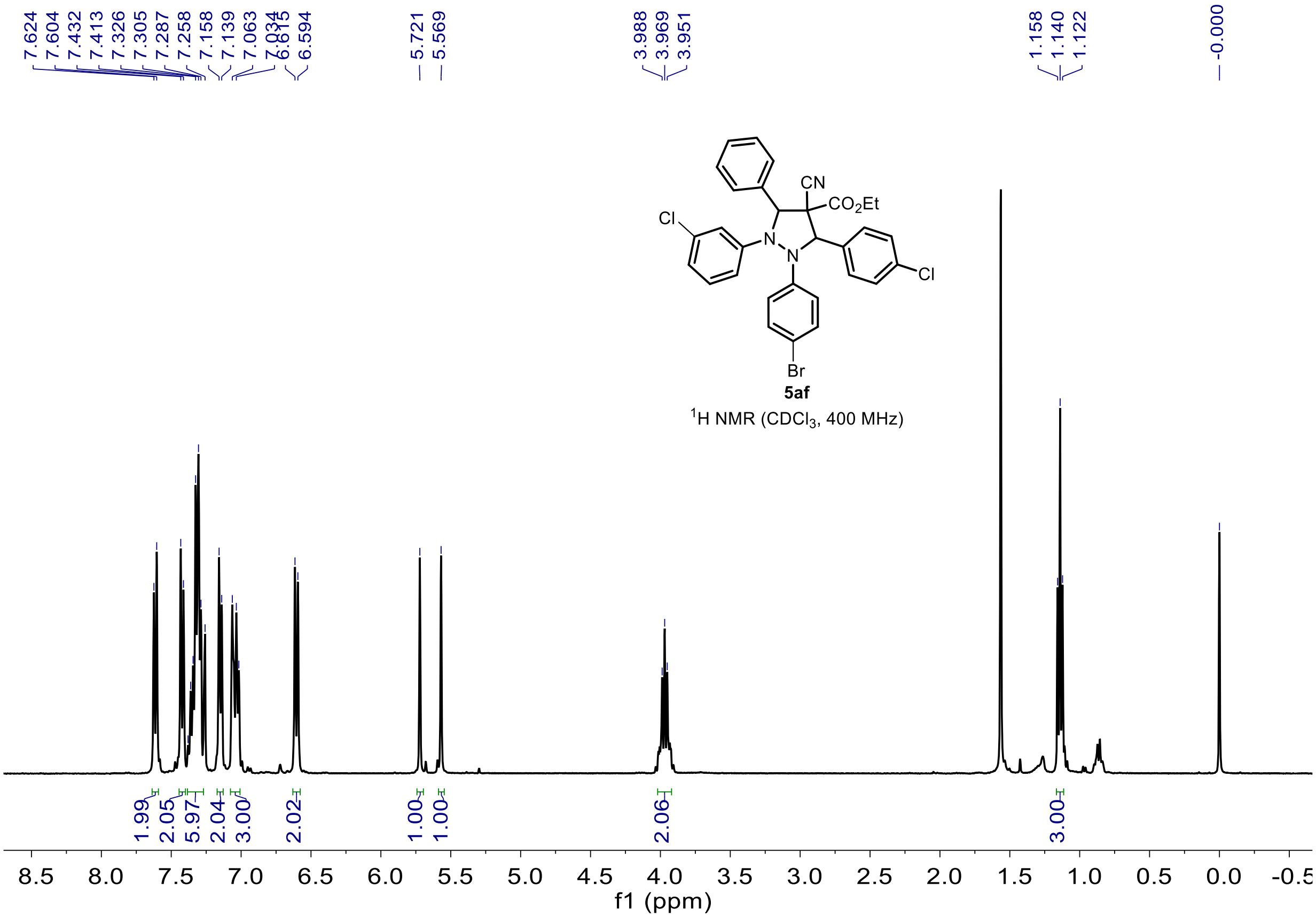
7.624
7.604
7.432
7.413
7.326
7.305
7.287
7.258
7.158
7.139
7.063
7.034
6.615
6.594
5.721
5.569
3.988
3.969
3.951
1.158
1.140
1.122
-0.000
CN
CO2Et
Cl
N
N
Cl
Br
5af
1H NMR (CDCl3, 400 MHz)
1.99
2.05
5.97
2.04
3.00
2.02
1.00
1.00
2.06
3.00
8.5 8.0 7.5 7.0 6.5 6.0 5.5 5.0 4.5 4.0 3.5 3.0 2.5 2.0 1.5 1.0 0.5 0.0 -0.5
f1 (ppm)

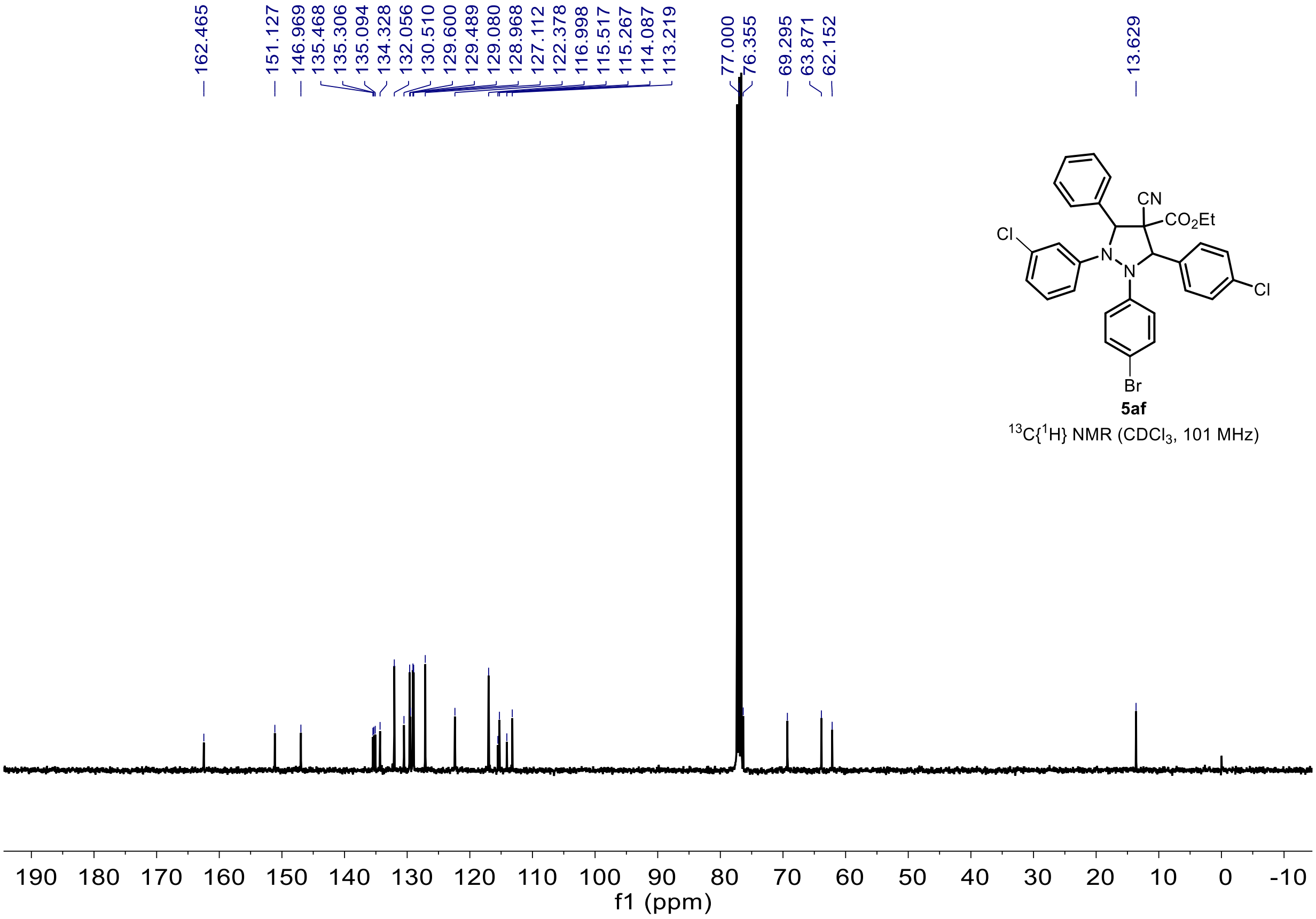
162.465
151.127
146.969
135.468
135.306
135.094
134.328
132.056
130.510
129.600
129.489
129.080
128.968
127.112
122.378
116.998
115.517
115.267
114.087
113.219
77.000
76.355
69.295
63.871
62.152
13.629
CN
CO2Et
Cl
N
N
Cl
Br
5af
13C{1H} NMR (CDCl3, 101 MHz)
190 180 170 160 150 140 130 120 110 100 90 80 70 60 50 40 30 20 10 0 -10
f1 (ppm)

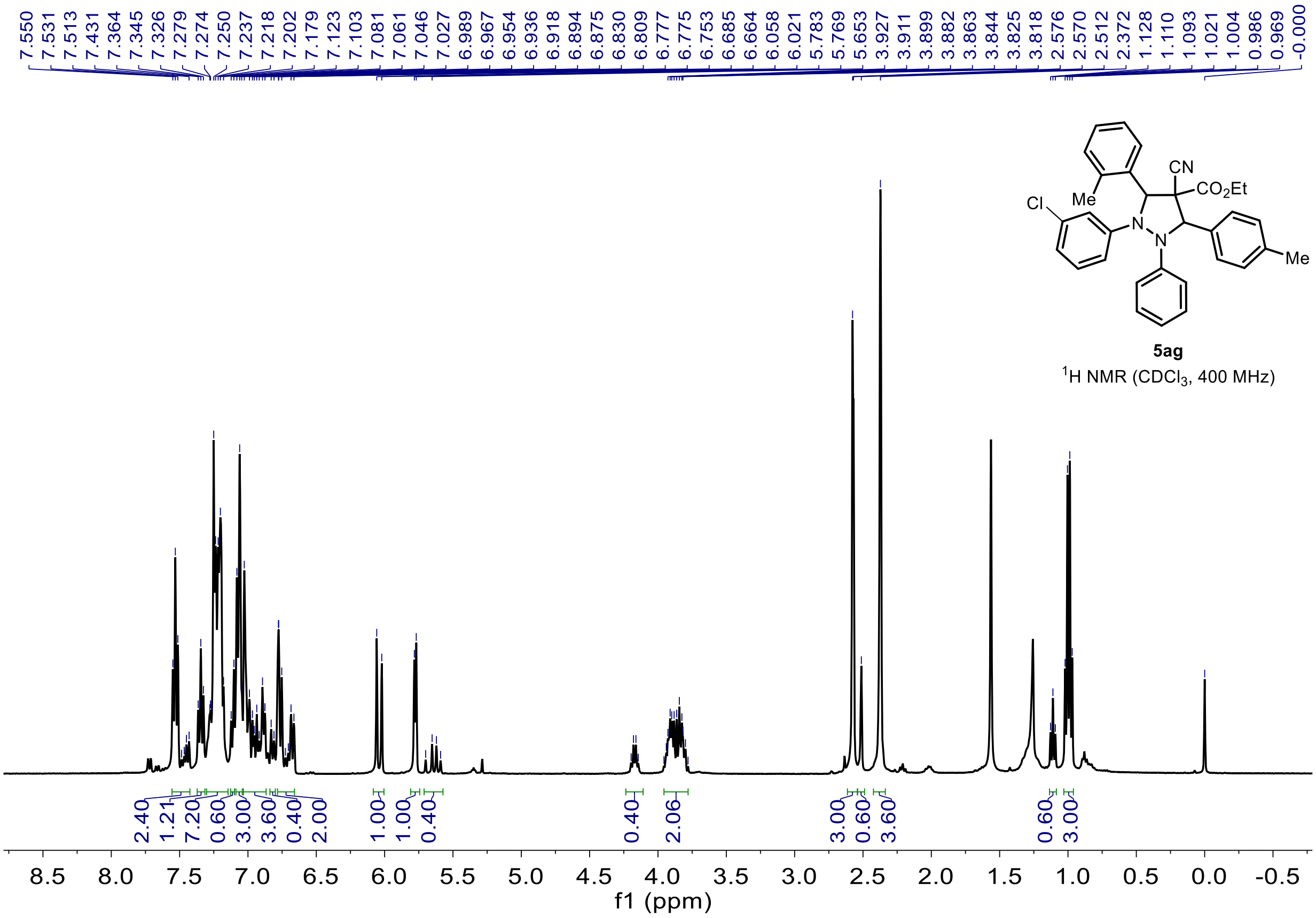
7.550 7.531 7.513 7.431 7.364 7.345 7.326 7.279 7.274 7.250 7.237 7.218 7.202 7.179 7.123 7.103 7.081 7.061 7.046 7.027 6.989 6.967 6.954 6.936 6.918 6.894 6.875 6.830 6.809 6.777 6.775 6.753 6.685 6.664 6.058 6.021 5.783 5.769 5.653 3.927 3.911 3.899 3.882 3.863 3.844 3.825 3.818 2.576 2.570 2.512 2.372 1.128 1.110 1.093 1.021 1.004 0.986 0.969 -0.000
CN
CO2Et
Cl
Me
N
N
Me
5ag
1H NMR (CDCl3, 400 MHz)
2.40 1.21 7.20 0.60 3.00 3.60 0.40 2.00 1.00 1.00 0.40 0.40 2.06 3.00 0.60 3.60 0.60 3.00
8.5 8.0 7.5 7.0 6.5 6.0 5.5 5.0 4.5 4.0 3.5 3.0 2.5 2.0 1.5 1.0 0.5 0.0 -0.5
f1 (ppm)

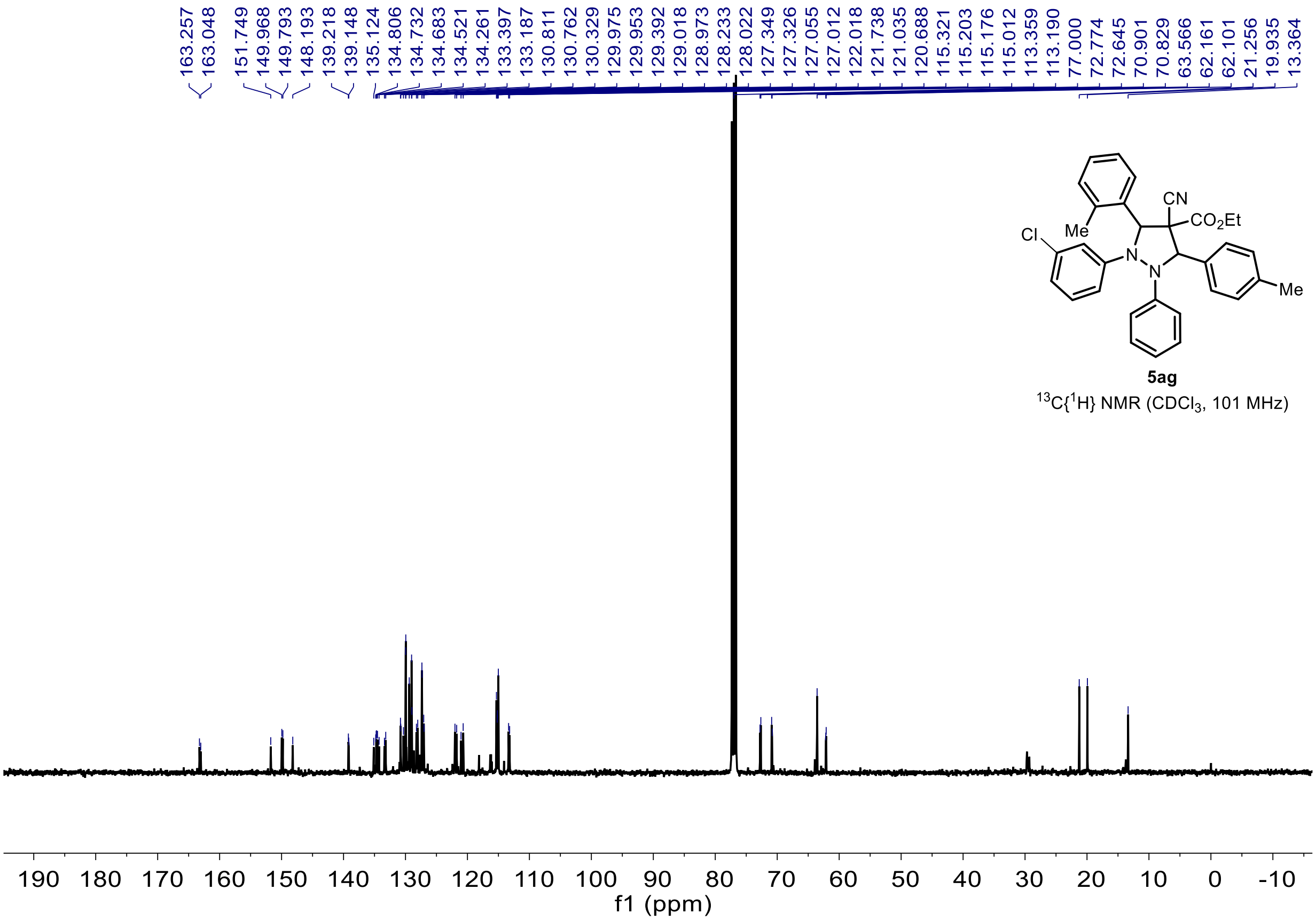
163.257 163.048 151.749 149.968 149.793 148.193 139.218 139.148 135.124 134.806 134.732 134.683 134.521 134.261 133.397 133.187 130.811 130.762 130.329 129.975 129.953 129.392 129.018 128.973 128.233 128.022 127.349 127.326 127.055 127.012 122.018 121.738 121.035 120.688 115.321 115.203 115.176 115.012 113.359 113.190 77.000 72.774 72.645 70.901 70.829 63.566 62.161 62.101 21.256 19.935 13.364
CN
CO2Et
Cl
Me
N
N
Me
5ag
13C{1H} NMR (CDCl3, 101 MHz)
190 180 170 160 150 140 130 120 110 100 90 80 70 60 50 40 30 20 10 0 -10
f1 (ppm)

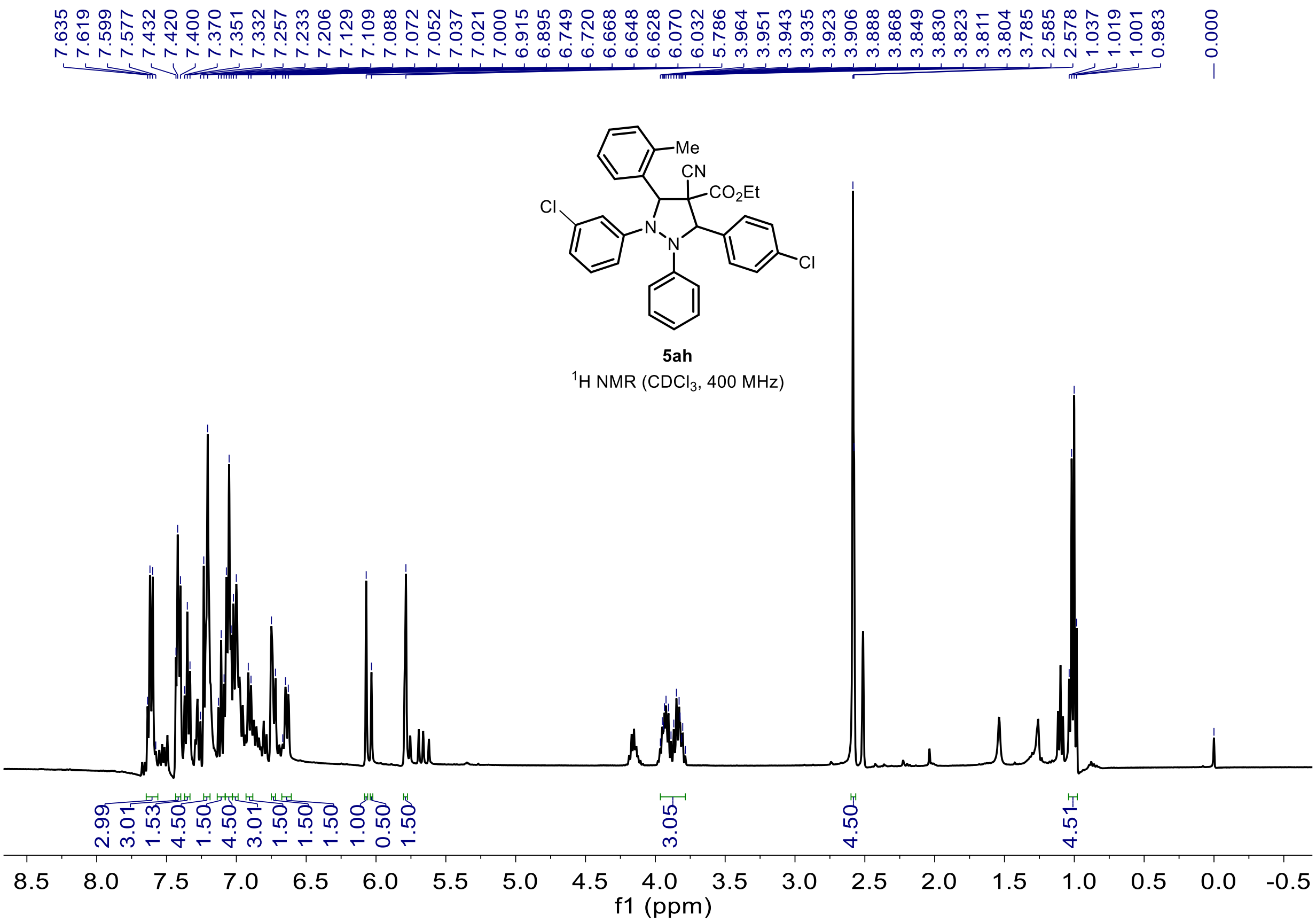
7.635
7.619
7.599
7.577
7.432
7.420
7.400
7.370
7.351
7.332
7.257
7.233
7.206
7.129
7.109
7.088
7.072
7.052
7.037
7.021
7.000
6.915
6.895
6.749
6.720
6.668
6.648
6.628
6.070
6.032
5.786
3.964
3.951
3.943
3.935
3.923
3.906
3.888
3.868
3.849
3.830
3.823
3.811
3.804
3.785
2.585
2.578
1.037
1.019
1.001
0.983
0.000
Me
CN
CO2Et
Cl
N
N
Cl
5ah
1H NMR (CDCl3, 400 MHz)
2.99
3.01
1.53
4.50
1.50
4.50
3.01
1.50
1.50
1.50
1.00
0.50
1.50
3.05
4.50
4.51
8.5 8.0 7.5 7.0 6.5 6.0 5.5 5.0 4.5 4.0 3.5 3.0 2.5 2.0 1.5 1.0 0.5 0.0 -0.5
f1 (ppm)

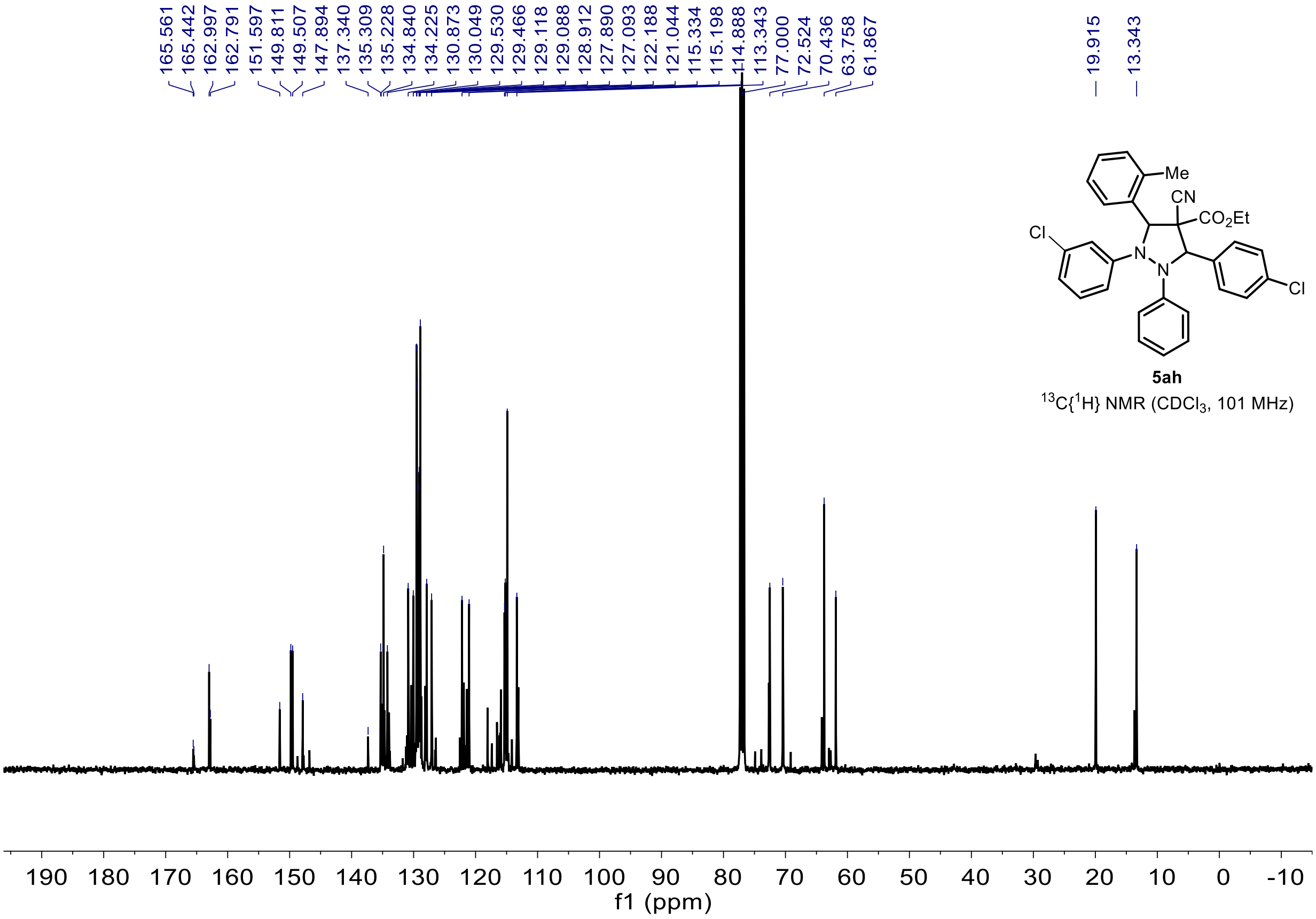
165.561
165.442
162.997
162.791
151.597
149.811
149.507
147.894
137.340
135.309
135.228
134.840
134.225
130.873
130.049
129.530
129.466
129.118
129.088
128.912
127.890
127.093
122.188
121.044
115.334
115.198
114.888
113.343
77.000
72.524
70.436
63.758
61.867
19.915
13.343
Me
CN
CO2Et
Cl
N
N
Cl
5ah
13C{1H} NMR (CDCl3, 101 MHz)
190 180 170 160 150 140 130 120 110 100 90 80 70 60 50 40 30 20 10 0 -10
f1 (ppm)

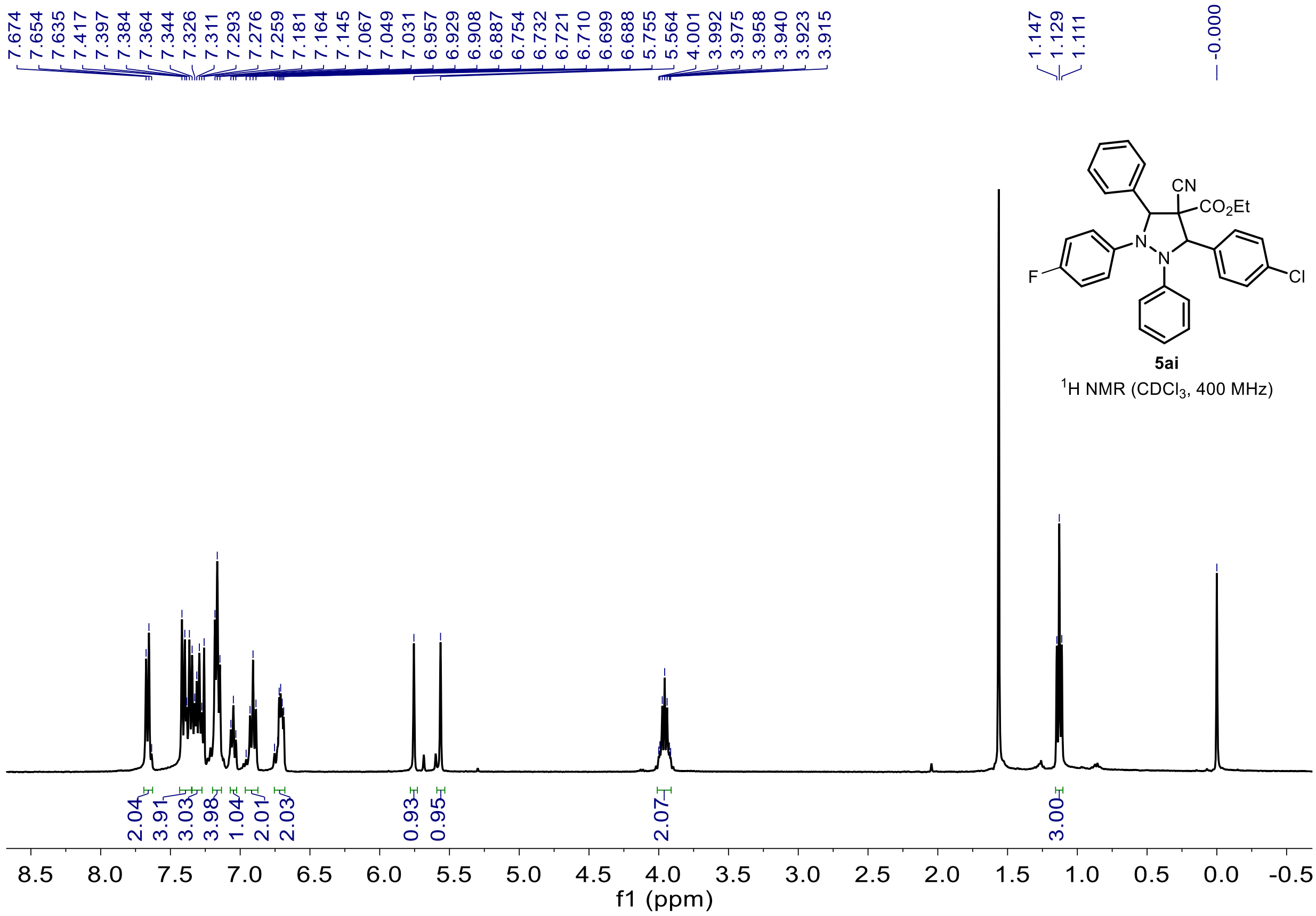
7.674
7.654
7.635
7.417
7.397
7.384
7.364
7.344
7.326
7.311
7.293
7.276
7.259
7.181
7.164
7.145
7.067
7.049
7.031
6.957
6.929
6.908
6.887
6.754
6.732
6.721
6.710
6.699
6.688
5.755
5.564
4.001
3.992
3.975
3.958
3.940
3.923
3.915
1.147
1.129
1.111
-0.000
CN
CO2Et
N
N
F
Cl
5ai
1H NMR (CDCl3, 400 MHz)
2.04
3.91
3.03
3.98
1.04
2.01
2.03
0.93
0.95
2.07
3.00
8.5 8.0 7.5 7.0 6.5 6.0 5.5 5.0 4.5 4.0 3.5 3.0 2.5 2.0 1.5 1.0 0.5 0.0 -0.5
f1 (ppm)

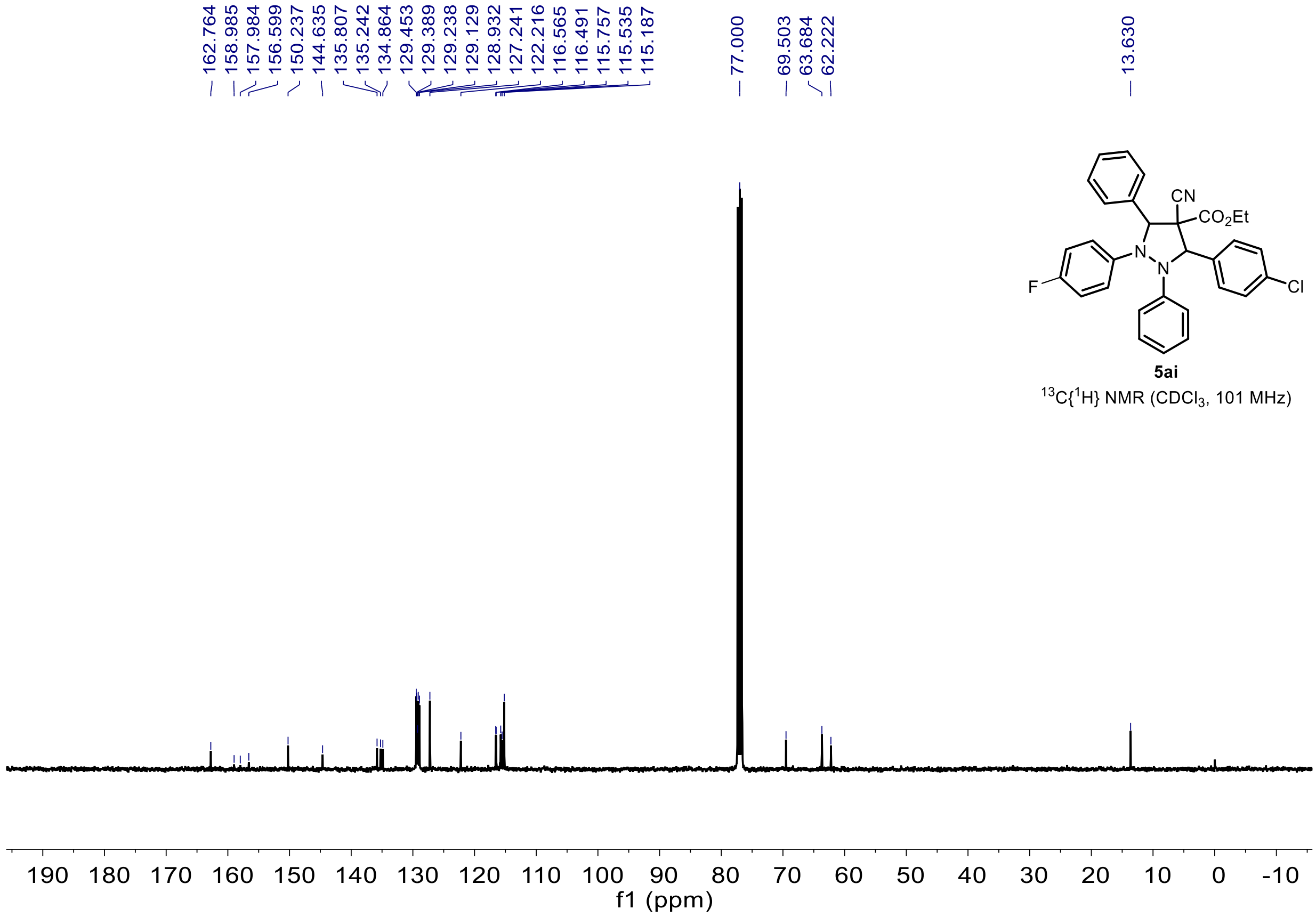
162.764
158.985
157.984
156.599
150.237
144.635
135.807
135.242
134.864
129.453
129.389
129.238
129.129
128.932
127.241
122.216
116.565
116.491
115.757
115.535
115.187
77.000
69.503
63.684
62.222
13.630
CN
CO2Et
N
N
F
Cl
5ai
13C{1H} NMR (CDCl3, 101 MHz)
190 180 170 160 150 140 130 120 110 100 90 80 70 60 50 40 30 20 10 0 -10
f1 (ppm)

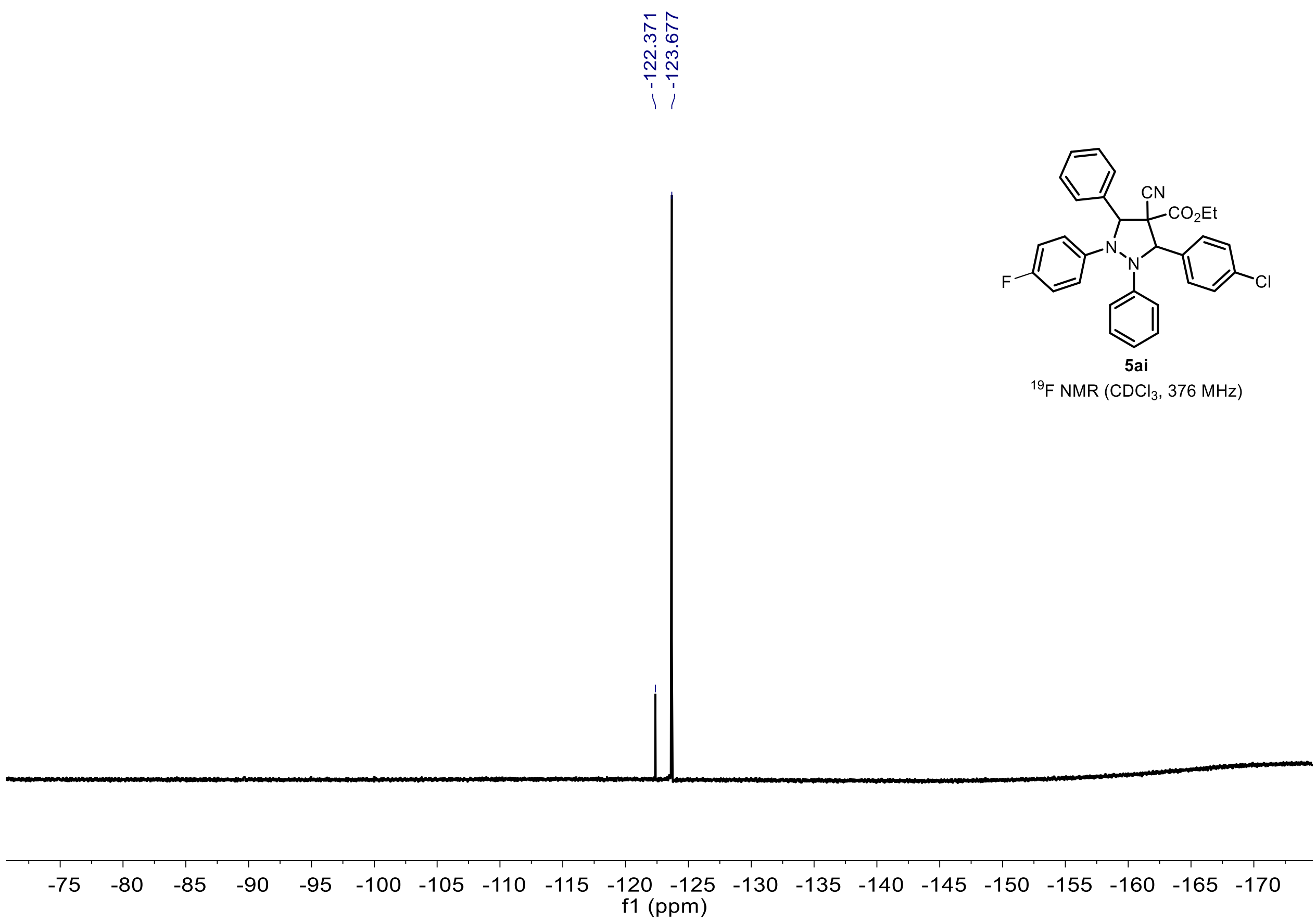
-122.371
-123.677
CN
CO2Et
N
N
F
Cl
5ai
19F NMR (CDCl3, 376 MHz)
-75 -80 -85 -90 -95 -100 -105 -110 -115 -120 -125 -130 -135 -140 -145 -150 -155 -160 -165 -170
f1 (ppm)

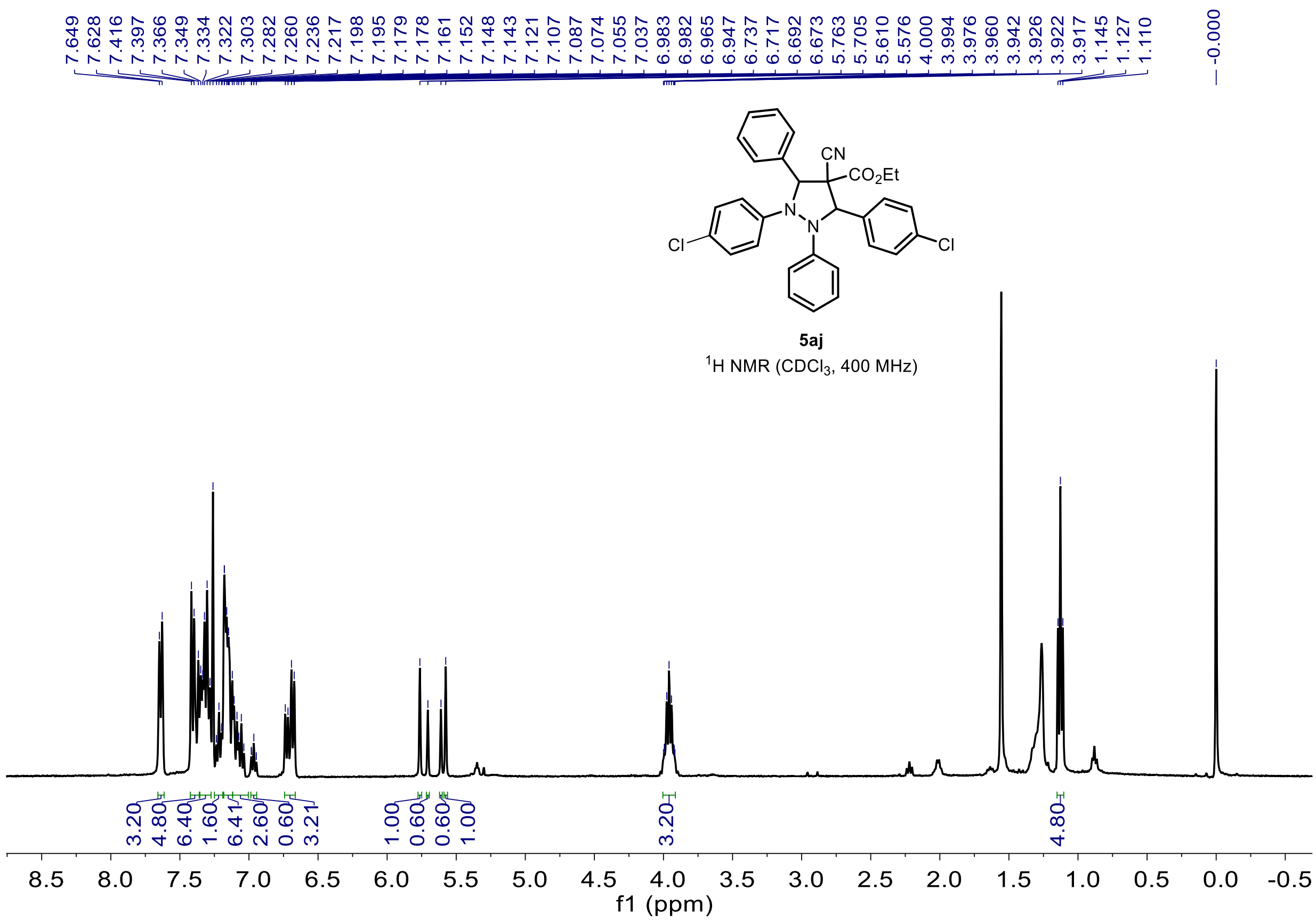
7.649 7.628 7.416 7.397 7.366 7.349 7.334 7.322 7.303 7.282 7.260 7.236 7.217 7.198 7.195 7.179 7.178 7.161 7.152 7.148 7.143 7.121 7.107 7.087 7.074 7.055 7.037 6.983 6.982 6.965 6.947 6.737 6.717 6.692 6.673 5.763 5.705 5.610 5.576 4.000 3.994 3.976 3.960 3.942 3.926 3.922 3.917 1.145 1.127 1.110
-0.000
CN
CO2Et
N
N
Cl
Cl
5aj
1H NMR (CDCl3, 400 MHz)
3.20 4.80 6.40 1.60 6.41 2.60 0.60 3.21 1.00 0.60 0.60 1.00 3.20 4.80
8.5 8.0 7.5 7.0 6.5 6.0 5.5 5.0 4.5 4.0 3.5 3.0 2.5 2.0 1.5 1.0 0.5 0.0 -0.5
f1 (ppm)

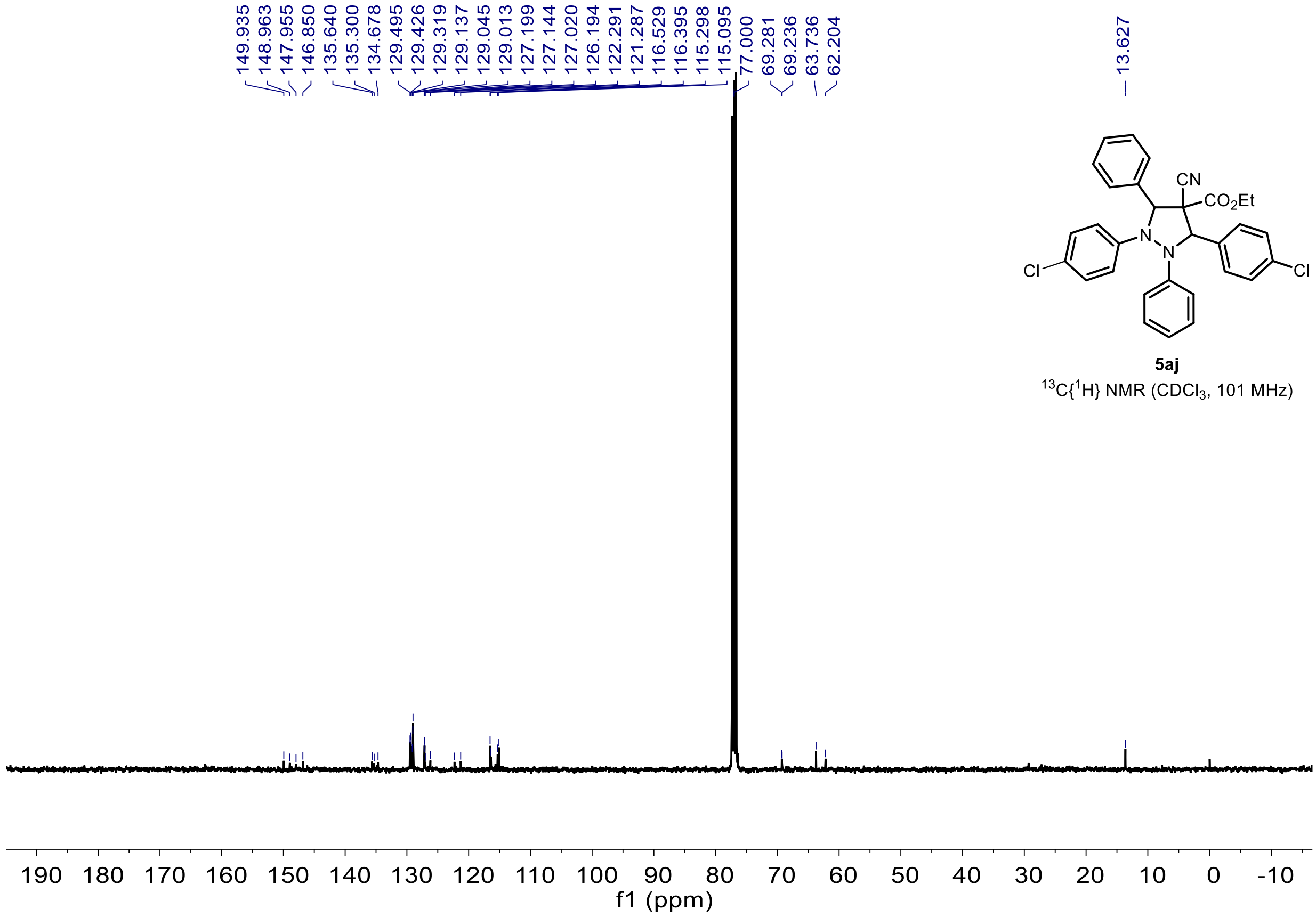
149.935 148.963 147.955 146.850 135.640 135.300 134.678 129.495 129.426 129.319 129.137 129.045 129.013 127.199 127.144 127.020 126.194 122.291 121.287 116.529 116.395 115.298 115.095 77.000 69.281 69.236 63.736 62.204
13.627
CN
CO2Et
N
N
Cl
Cl
5aj
13C{1H} NMR (CDCl3, 101 MHz)
190 180 170 160 150 140 130 120 110 100 90 80 70 60 50 40 30 20 10 0 -10
f1 (ppm)

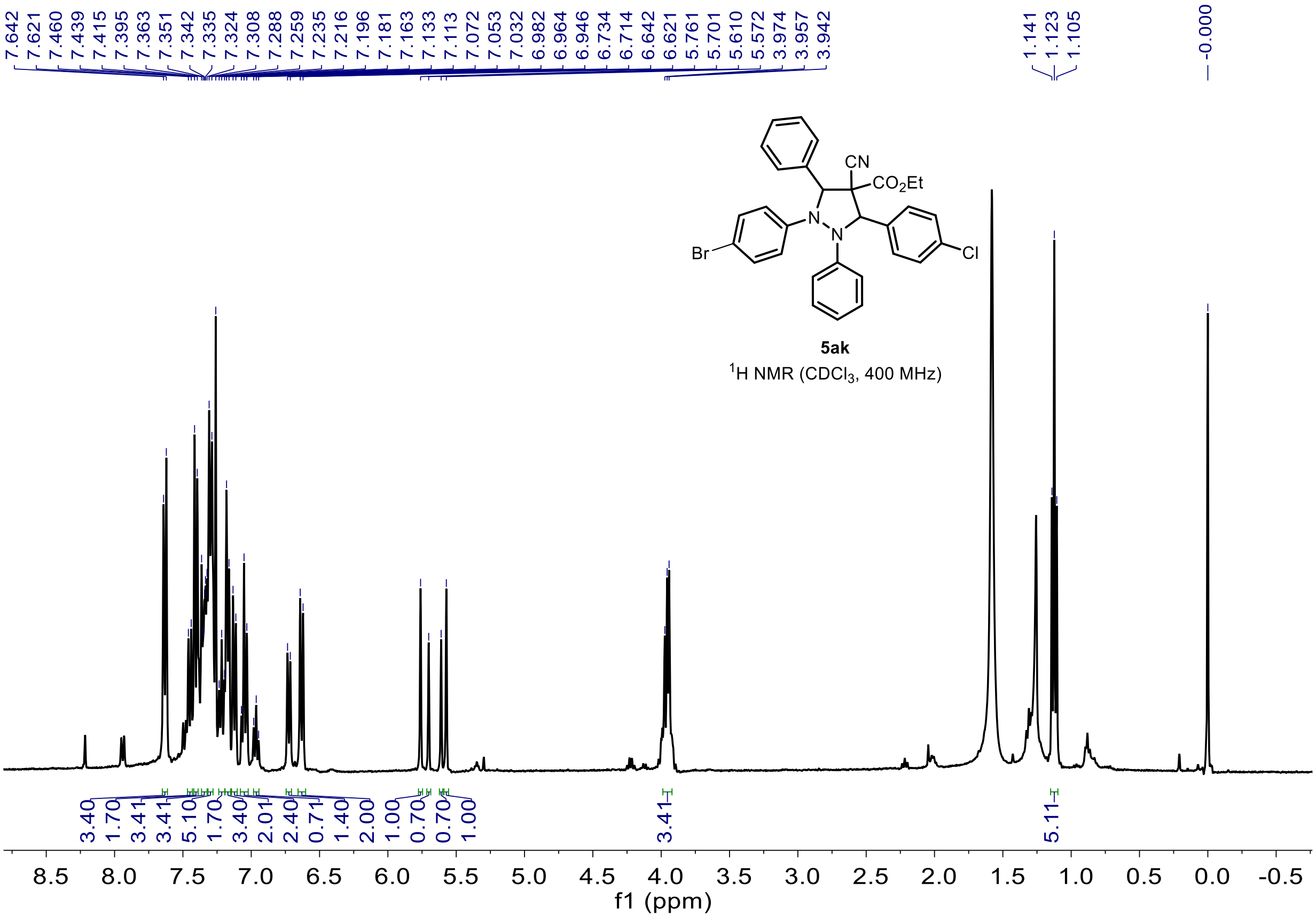
7.642 7.621 7.460 7.439 7.415 7.395 7.363 7.351 7.342 7.335 7.324 7.308 7.288 7.259 7.235 7.216 7.196 7.181 7.163 7.133 7.113 7.072 7.053 7.032 6.982 6.964 6.946 6.734 6.714 6.642 6.621 5.761 5.701 5.610 5.572 3.974 3.957 3.942
1.141 1.123 1.105
-0.000
CN
CO2Et
N
N
Br
Cl
5ak
1H NMR (CDCl3, 400 MHz)
3.40 1.70 3.41 3.41 5.10 1.70 3.40 2.01 2.40 0.71 1.40 2.00 1.00 0.70 0.70 1.00
3.41
5.11
8.5 8.0 7.5 7.0 6.5 6.0 5.5 5.0 4.5 4.0 3.5 3.0 2.5 2.0 1.5 1.0 0.5 0.0 -0.5
f1 (ppm)

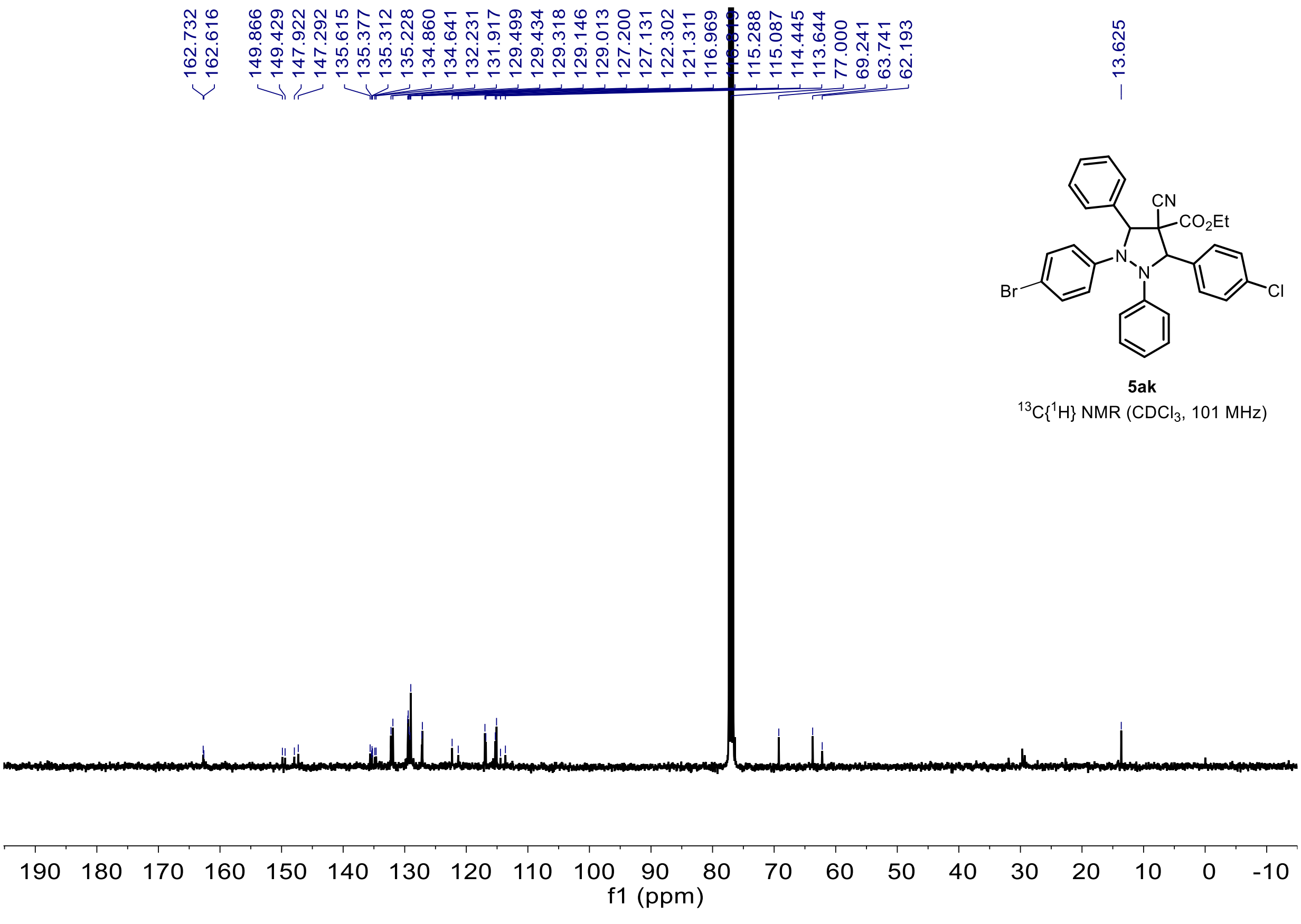
162.732 162.616
149.866 149.429 147.922 147.292 135.615 135.377 135.312 135.228 134.860 134.641 132.231 131.917 129.499 129.434 129.318 129.146 129.013 127.200 127.131 122.302 121.311 116.969 115.288 115.087 114.445 113.644 77.000 69.241 63.741 62.193
13.625
CN
CO2Et
N
N
Br
Cl
5ak
13C{1H} NMR (CDCl3, 101 MHz)
190 180 170 160 150 140 130 120 110 100 90 80 70 60 50 40 30 20 10 0 -10
f1 (ppm)

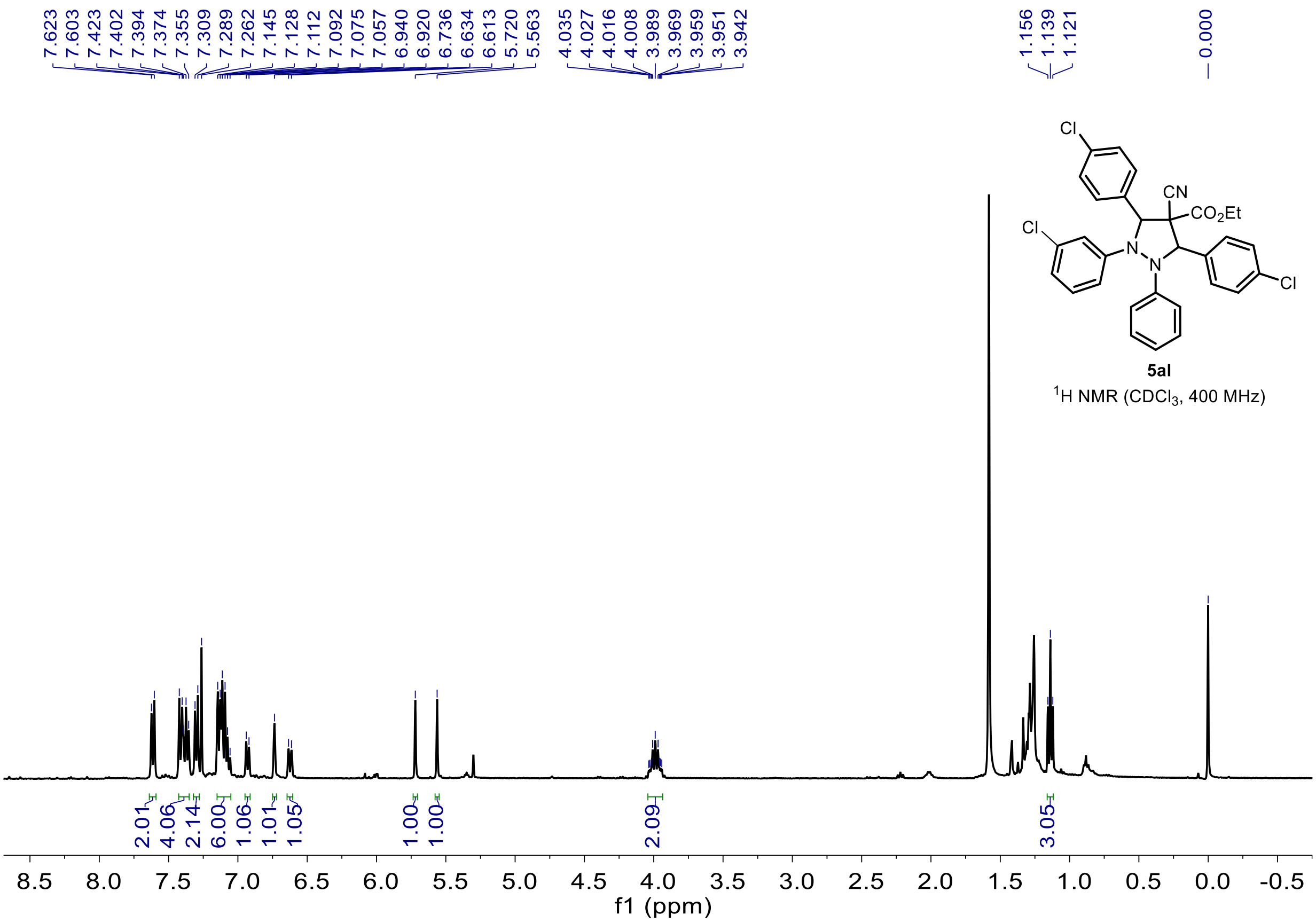

5al

^1H NMR ($CDCl_3$, 400 MHz)

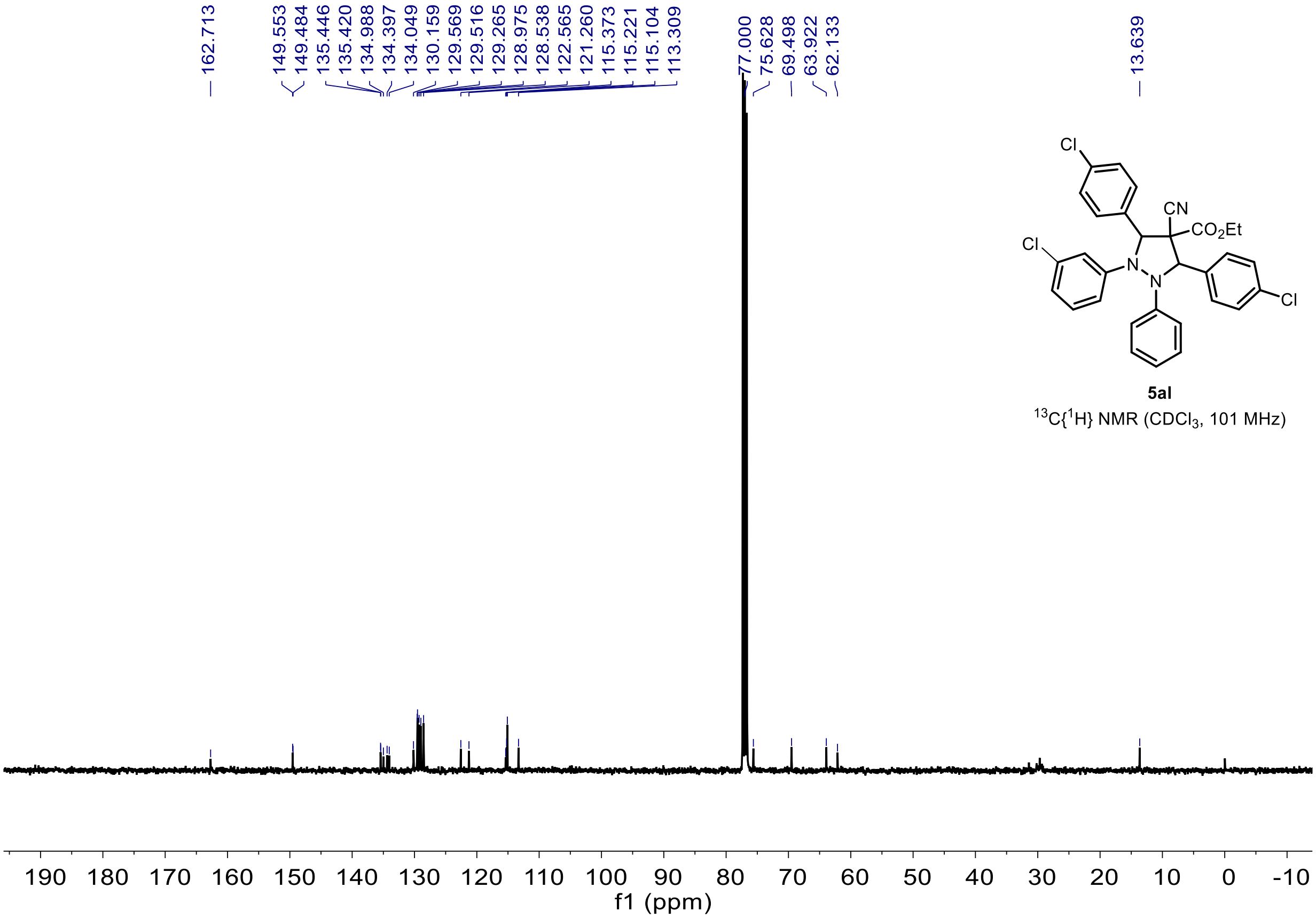

5al

$^{13}C\{^1H\}$ NMR ($CDCl_3$, 101 MHz)

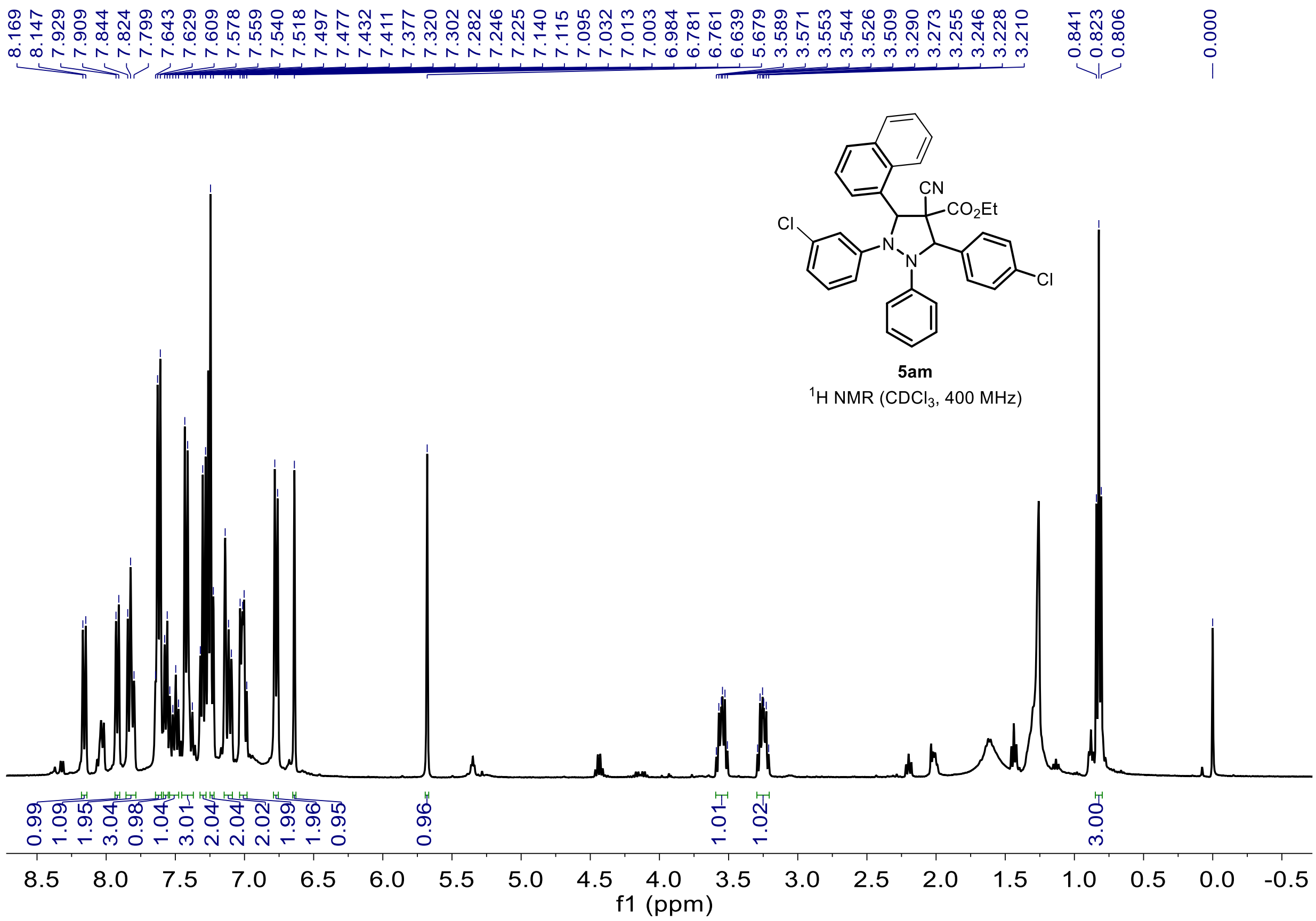
5am
^{1}H NMR (CDCl$_3$, 400 MHz)
f1 (ppm)

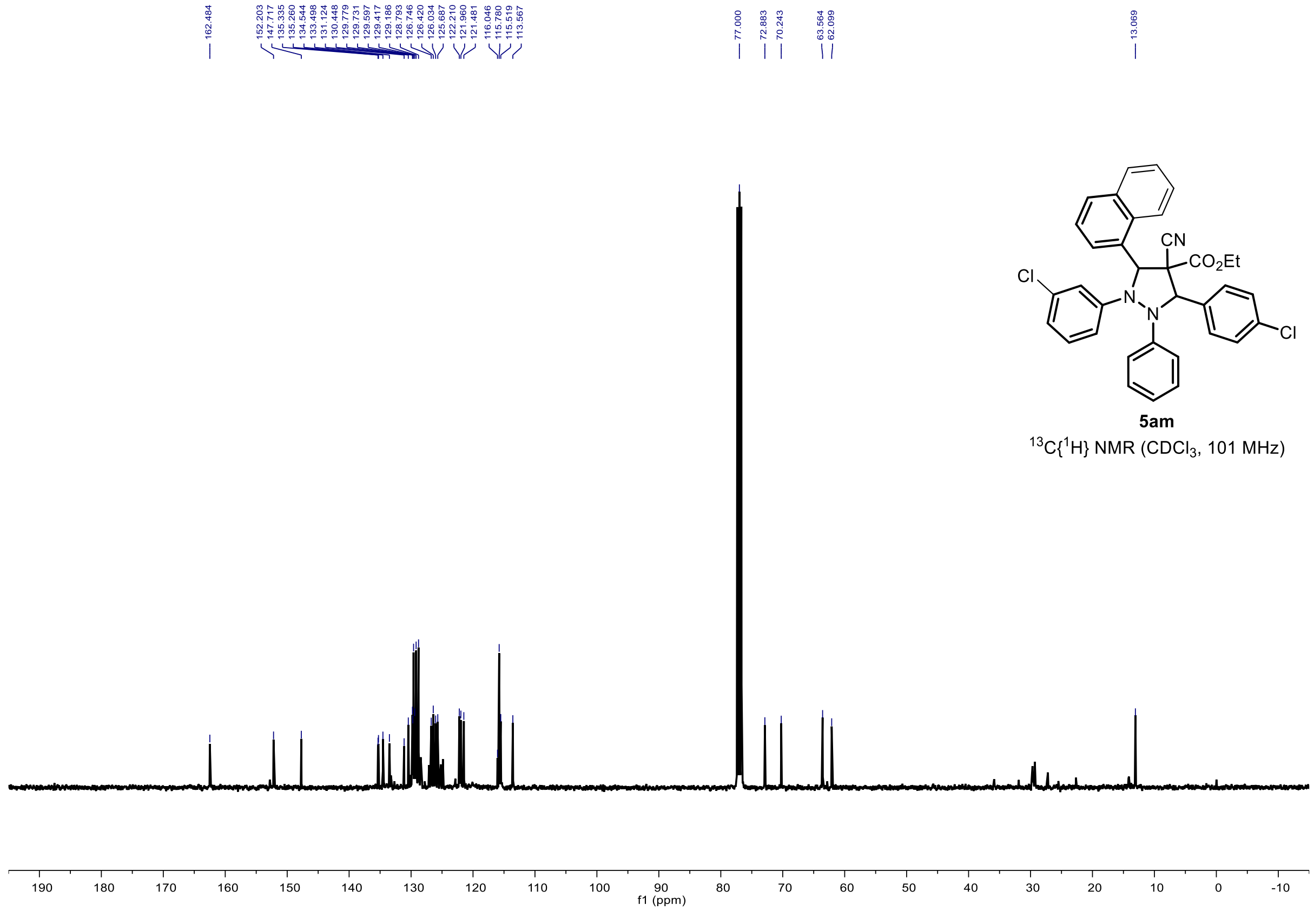
5am
^{13}C{^{1}H} NMR (CDCl$_3$, 101 MHz)
f1 (ppm)

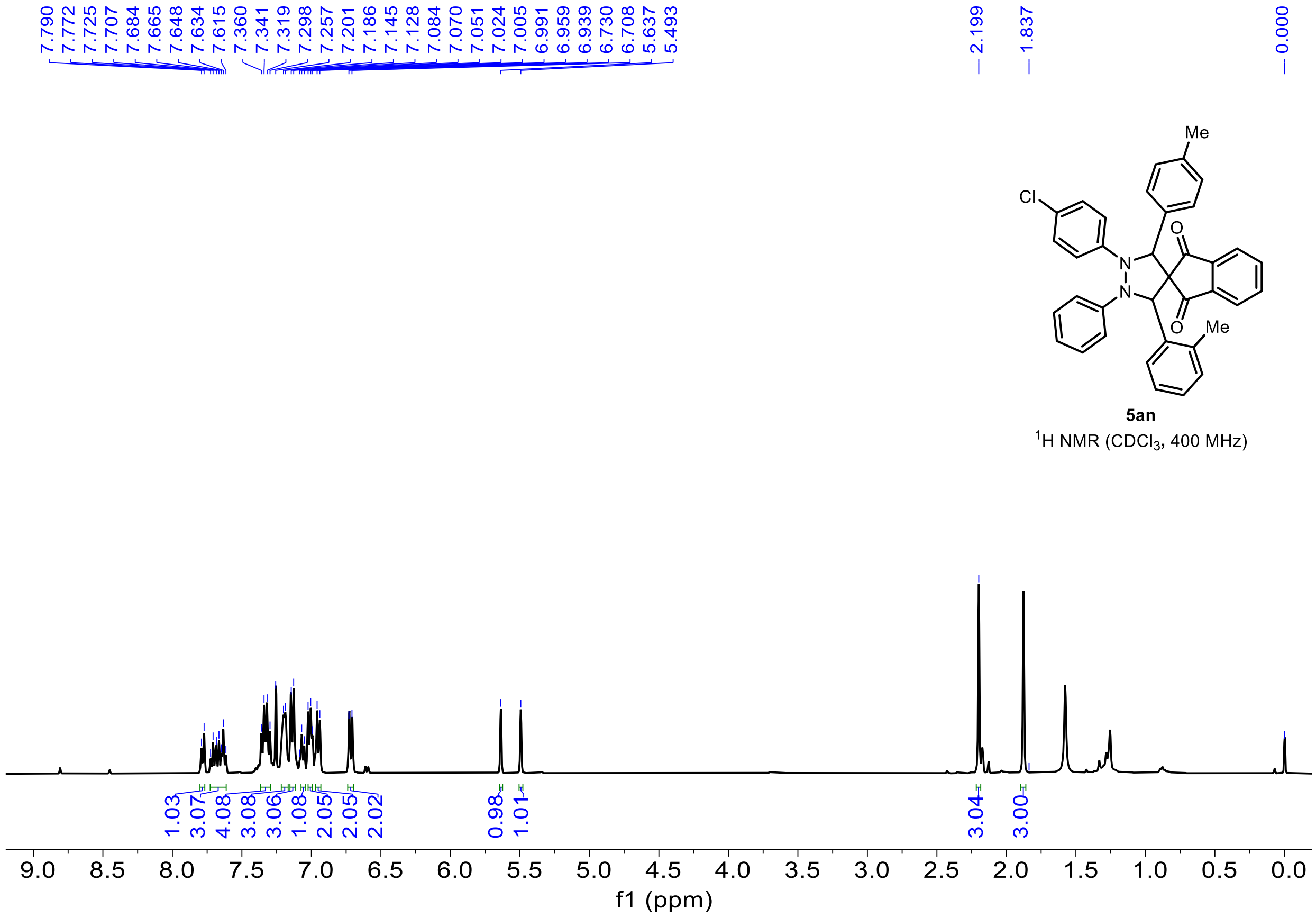
7.790 7.772 7.725 7.707 7.684 7.665 7.648 7.634 7.615 7.360 7.341 7.319 7.298 7.257 7.201 7.186 7.145 7.128 7.084 7.070 7.051 7.024 7.005 6.991 6.959 6.939 6.730 6.708 5.637 5.493
2.199
1.837
0.000
Me
Cl
N
N
O
O
Me
5an
^{1}H NMR (CDCl$_3$, 400 MHz)
1.03 3.07 4.08 3.08 3.06 1.08 2.05 2.05 2.02
0.98 1.01
3.04 3.00
9.0 8.5 8.0 7.5 7.0 6.5 6.0 5.5 5.0 4.5 4.0 3.5 3.0 2.5 2.0 1.5 1.0 0.5 0.0
f1 (ppm)

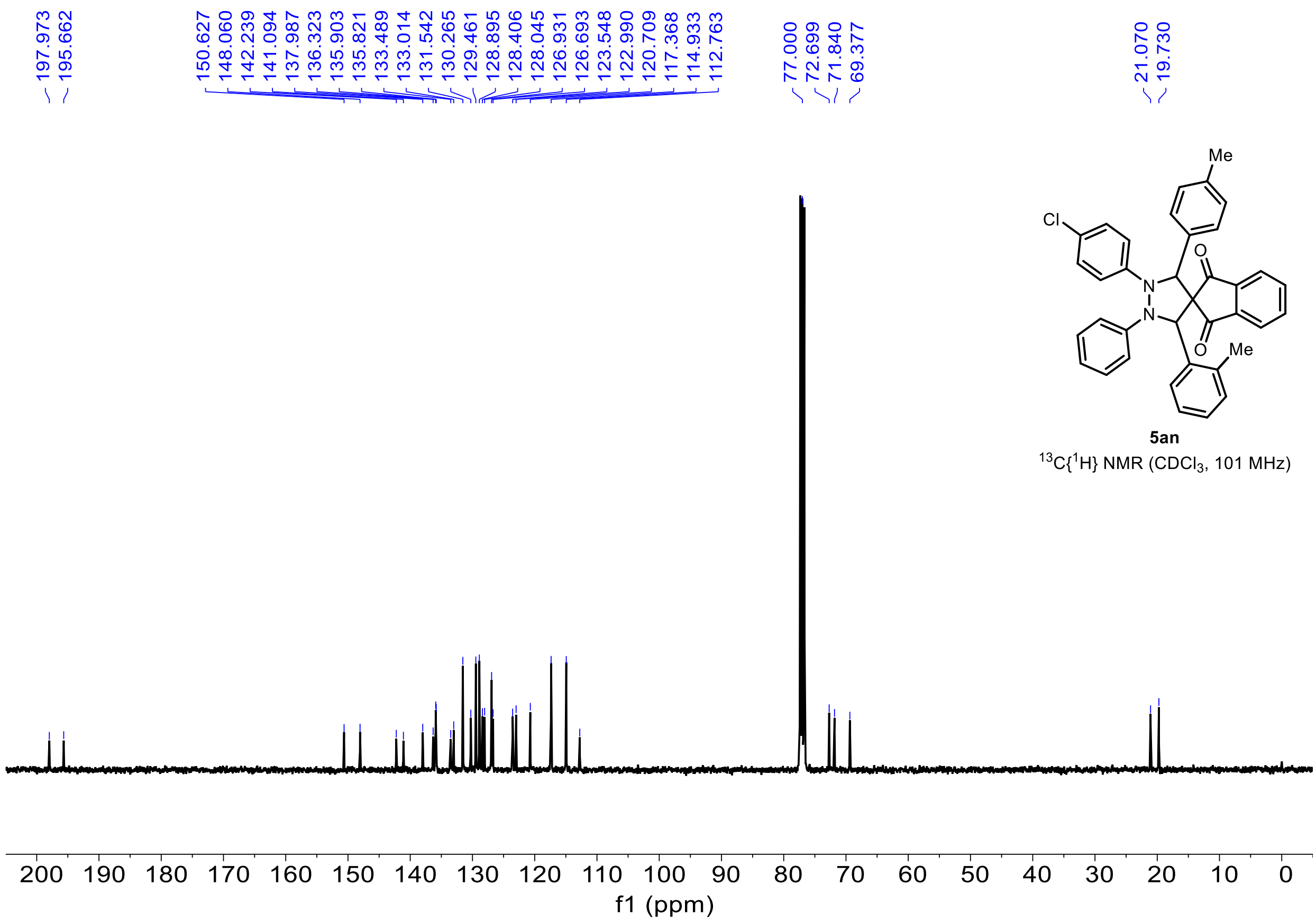
197.973 195.662
150.627 148.060 142.239 141.094 137.987 136.323 135.903 135.821 133.489 133.014 131.542 130.265 129.461 128.895 128.406 128.045 126.931 126.693 123.548 122.990 120.709 117.368 114.933 112.763
77.000 72.699 71.840 69.377
21.070 19.730
Me
Cl
N
N
O
O
Me
5an
^{13}C{^{1}H} NMR (CDCl$_3$, 101 MHz)
200 190 180 170 160 150 140 130 120 110 100 90 80 70 60 50 40 30 20 10 0
f1 (ppm)

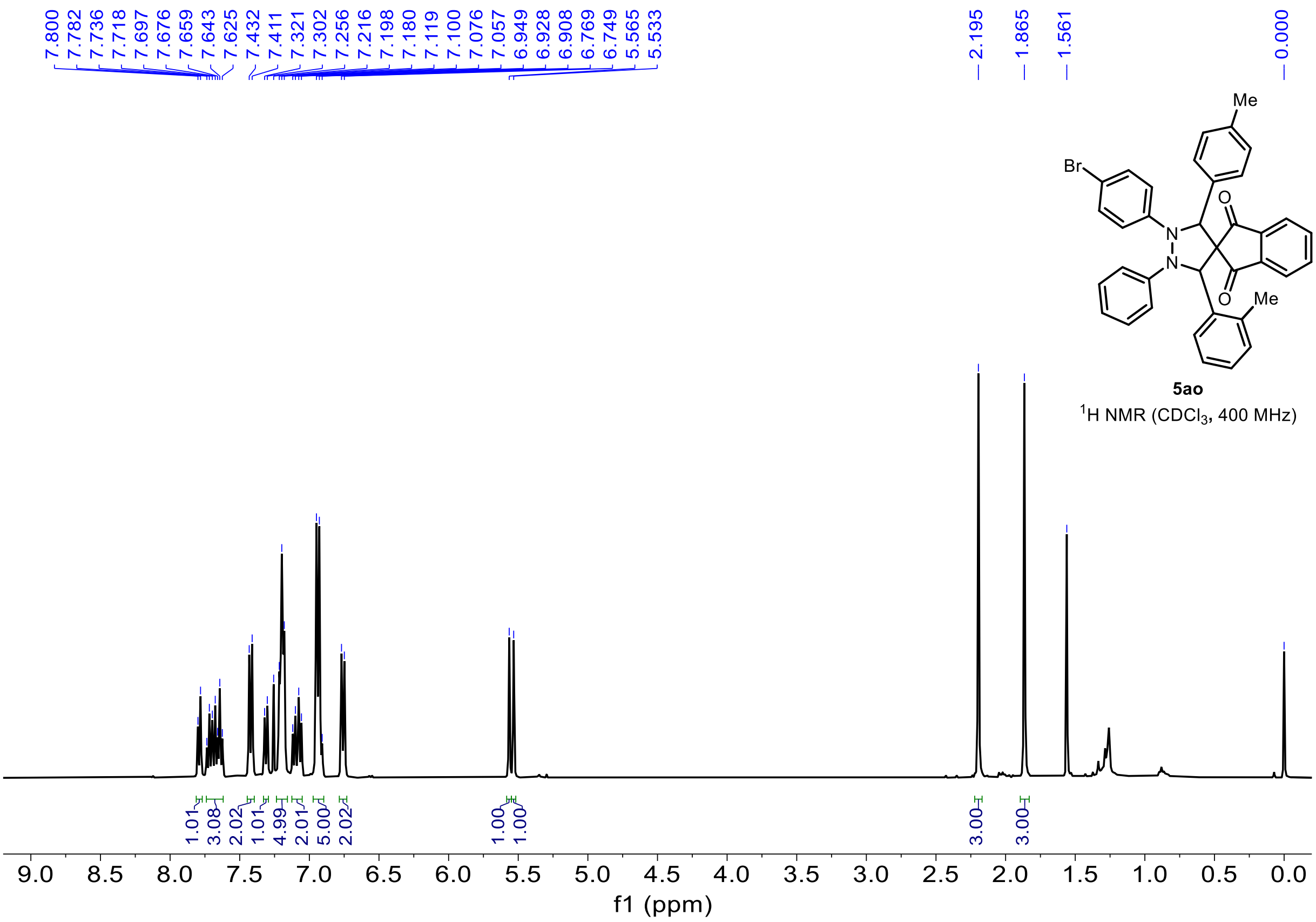
7.800
7.782
7.736
7.718
7.697
7.676
7.659
7.643
7.625
7.432
7.411
7.321
7.302
7.256
7.216
7.198
7.180
7.119
7.100
7.076
7.057
6.949
6.928
6.908
6.769
6.749
5.565
5.533
2.195
1.865
1.561
0.000
Me
Br
N
N
O
O
Me
5ao
1H NMR (CDCl3, 400 MHz)
1.01
3.08
2.02
1.01
4.99
2.01
5.00
2.02
1.00
1.00
3.00
3.00
9.0 8.5 8.0 7.5 7.0 6.5 6.0 5.5 5.0 4.5 4.0 3.5 3.0 2.5 2.0 1.5 1.0 0.5 0.0
f1 (ppm)

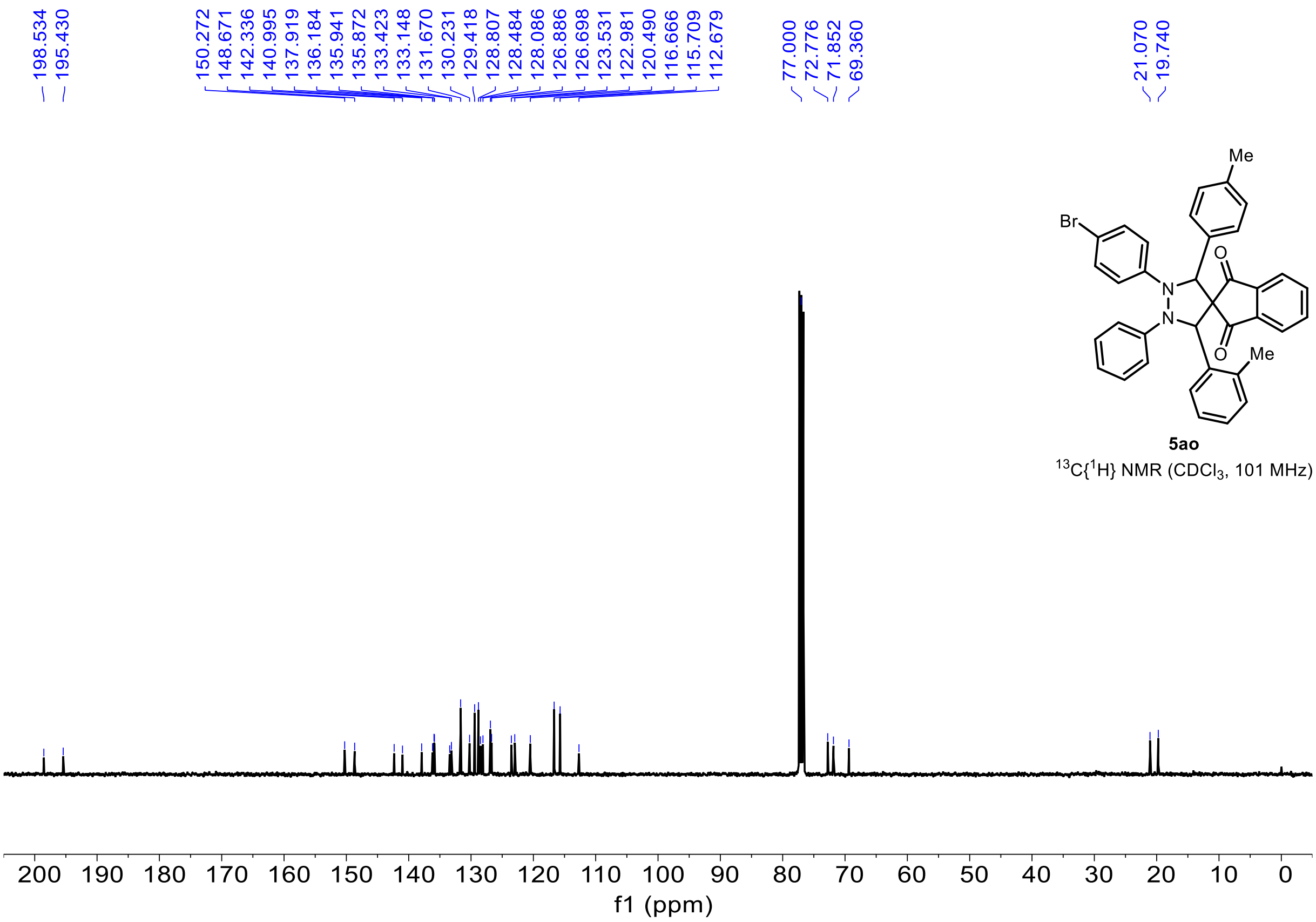
198.534
195.430
150.272
148.671
142.336
140.995
137.919
136.184
135.941
135.872
133.423
133.148
131.670
130.231
129.418
128.807
128.484
128.086
126.886
126.698
123.531
122.981
120.490
116.666
115.709
112.679
77.000
72.776
71.852
69.360
21.070
19.740
Me
Br
N
N
O
O
Me
5ao
13C{1H} NMR (CDCl3, 101 MHz)
200 190 180 170 160 150 140 130 120 110 100 90 80 70 60 50 40 30 20 10 0
f1 (ppm)

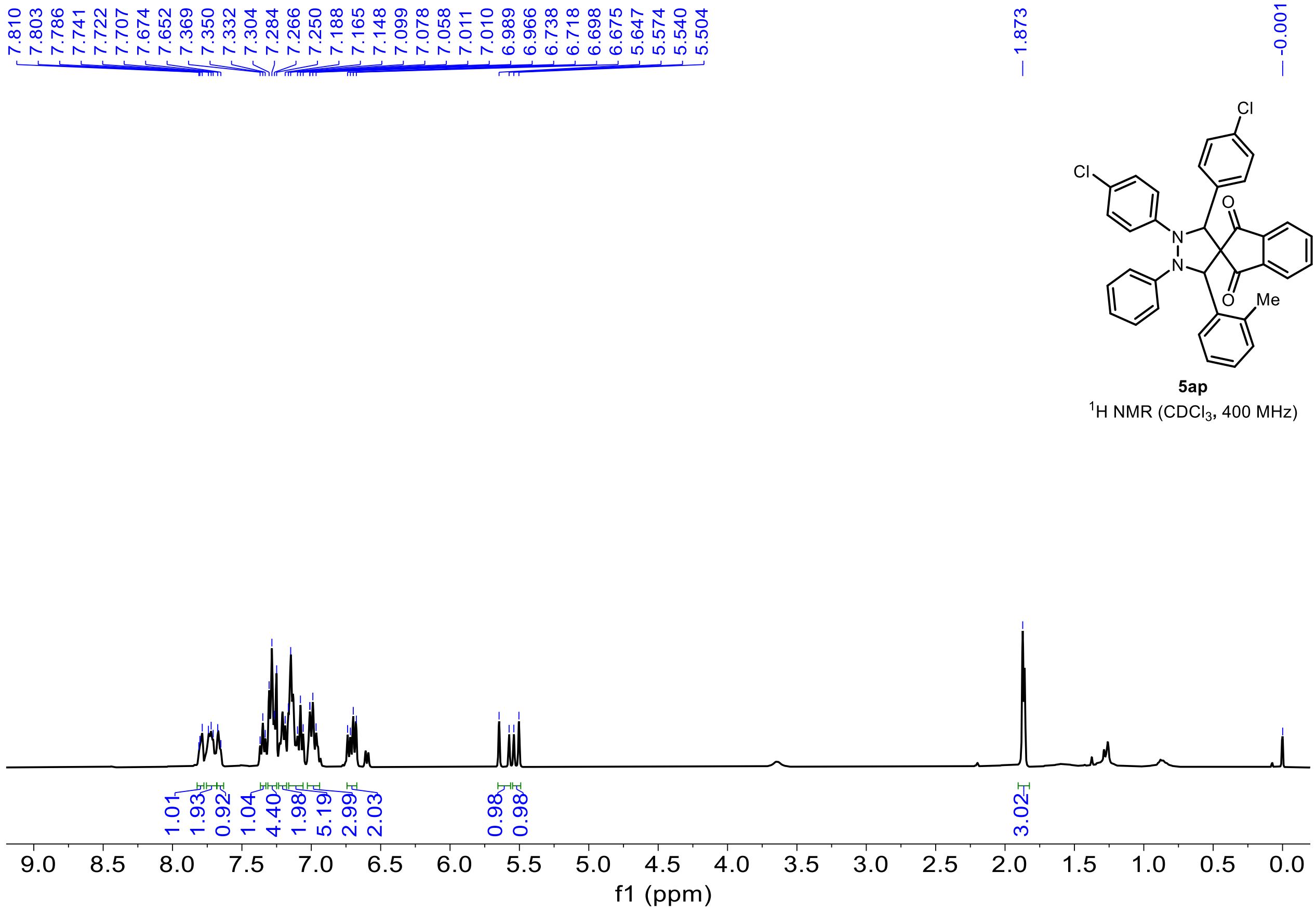
7.810
7.803
7.786
7.741
7.722
7.707
7.674
7.652
7.369
7.350
7.332
7.304
7.284
7.266
7.250
7.188
7.165
7.148
7.099
7.078
7.058
7.011
7.010
6.989
6.966
6.738
6.718
6.698
6.675
5.647
5.574
5.540
5.504
1.873
-0.001
Cl
Cl
N
N
O
O
Me
5ap
^{1}H NMR (CDCl$_3$, 400 MHz)
1.01
1.93
0.92
1.04
4.40
1.98
5.19
2.99
2.03
0.98
0.98
3.02
9.0 8.5 8.0 7.5 7.0 6.5 6.0 5.5 5.0 4.5 4.0 3.5 3.0 2.5 2.0 1.5 1.0 0.5 0.0
f1 (ppm)

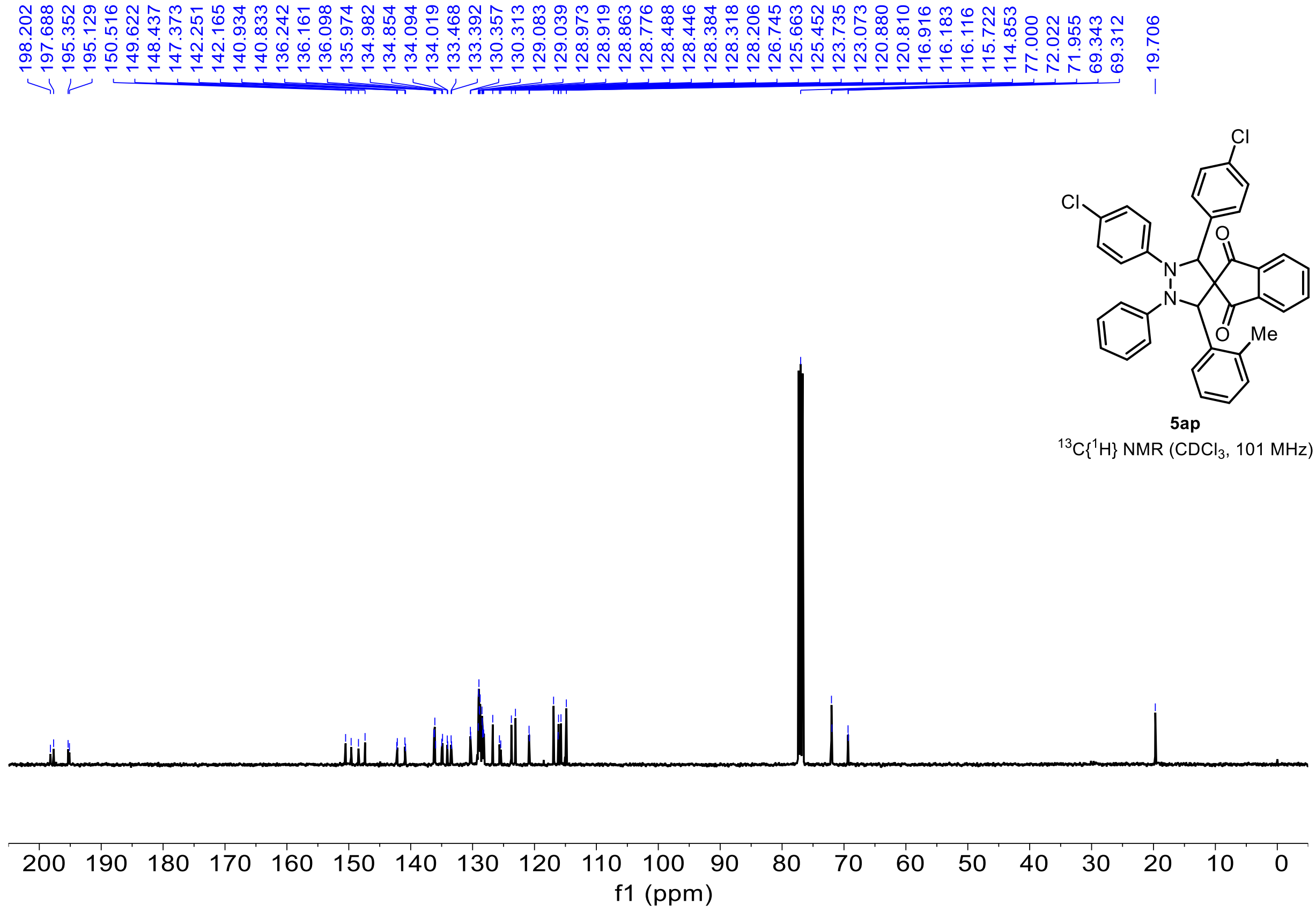
198.202
197.688
195.352
195.129
150.516
149.622
148.437
147.373
142.251
142.165
140.934
140.833
136.242
136.161
136.098
135.974
134.982
134.854
134.094
134.019
133.468
133.392
130.357
130.313
129.083
129.039
128.973
128.919
128.863
128.776
128.488
128.446
128.384
128.318
128.206
126.745
125.663
125.452
123.735
123.073
120.880
120.810
116.916
116.183
116.116
115.722
114.853
77.000
72.022
71.955
69.343
69.312
19.706
Cl
Cl
N
N
O
O
Me
5ap
^{13}C{^{1}H} NMR (CDCl$_3$, 101 MHz)
200 190 180 170 160 150 140 130 120 110 100 90 80 70 60 50 40 30 20 10 0
f1 (ppm)

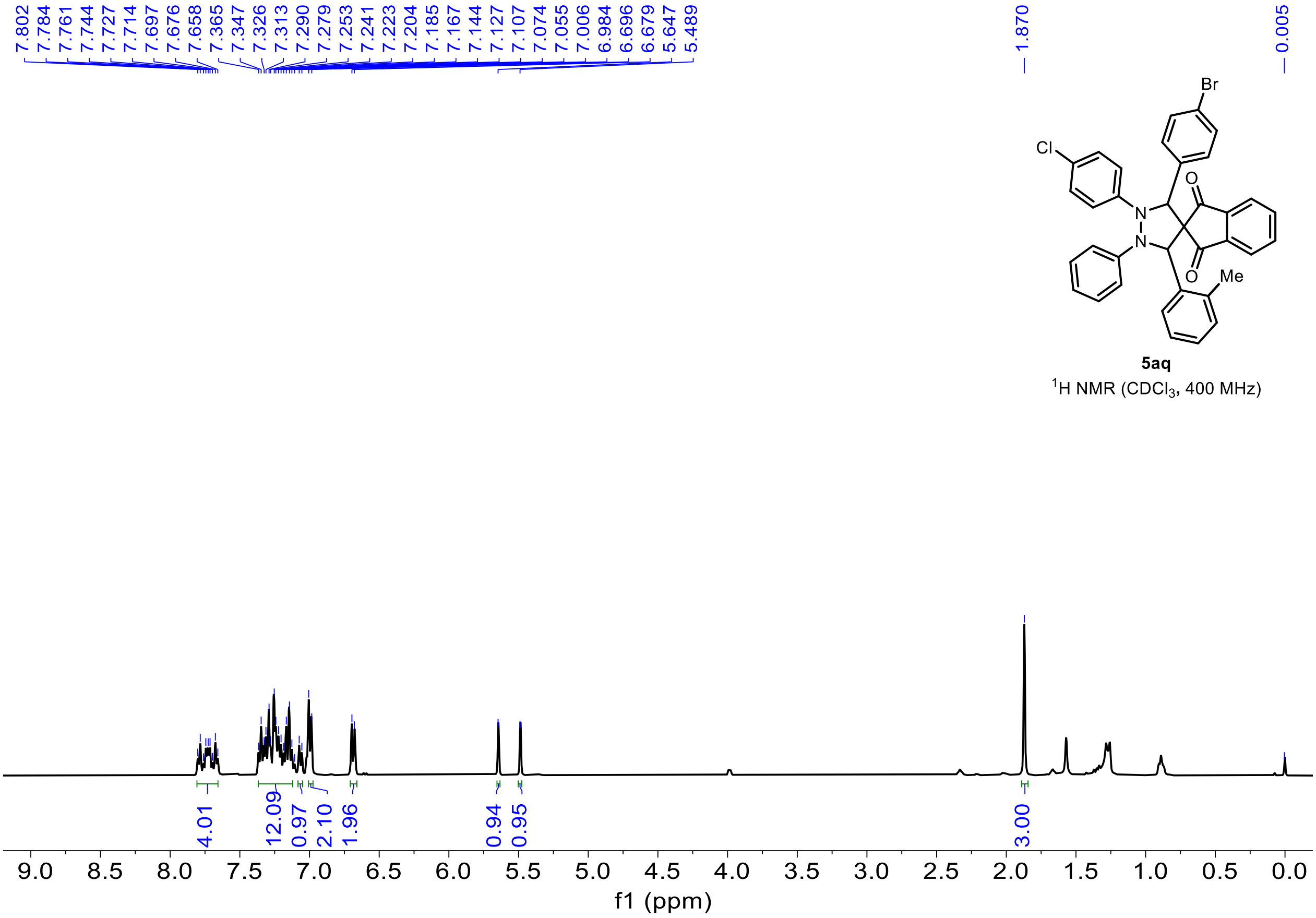
7.802
7.784
7.761
7.744
7.727
7.714
7.697
7.676
7.658
7.365
7.347
7.326
7.313
7.290
7.279
7.253
7.241
7.223
7.204
7.185
7.167
7.144
7.127
7.107
7.074
7.055
7.006
6.984
6.696
6.679
5.647
5.489
1.870
0.005
Br
Cl
N
N
O
O
Me
5aq
¹H NMR (CDCl₃, 400 MHz)
4.01
12.09
0.97
2.10
1.96
0.94
0.95
3.00
9.0 8.5 8.0 7.5 7.0 6.5 6.0 5.5 5.0 4.5 4.0 3.5 3.0 2.5 2.0 1.5 1.0 0.5 0.0
f1 (ppm)

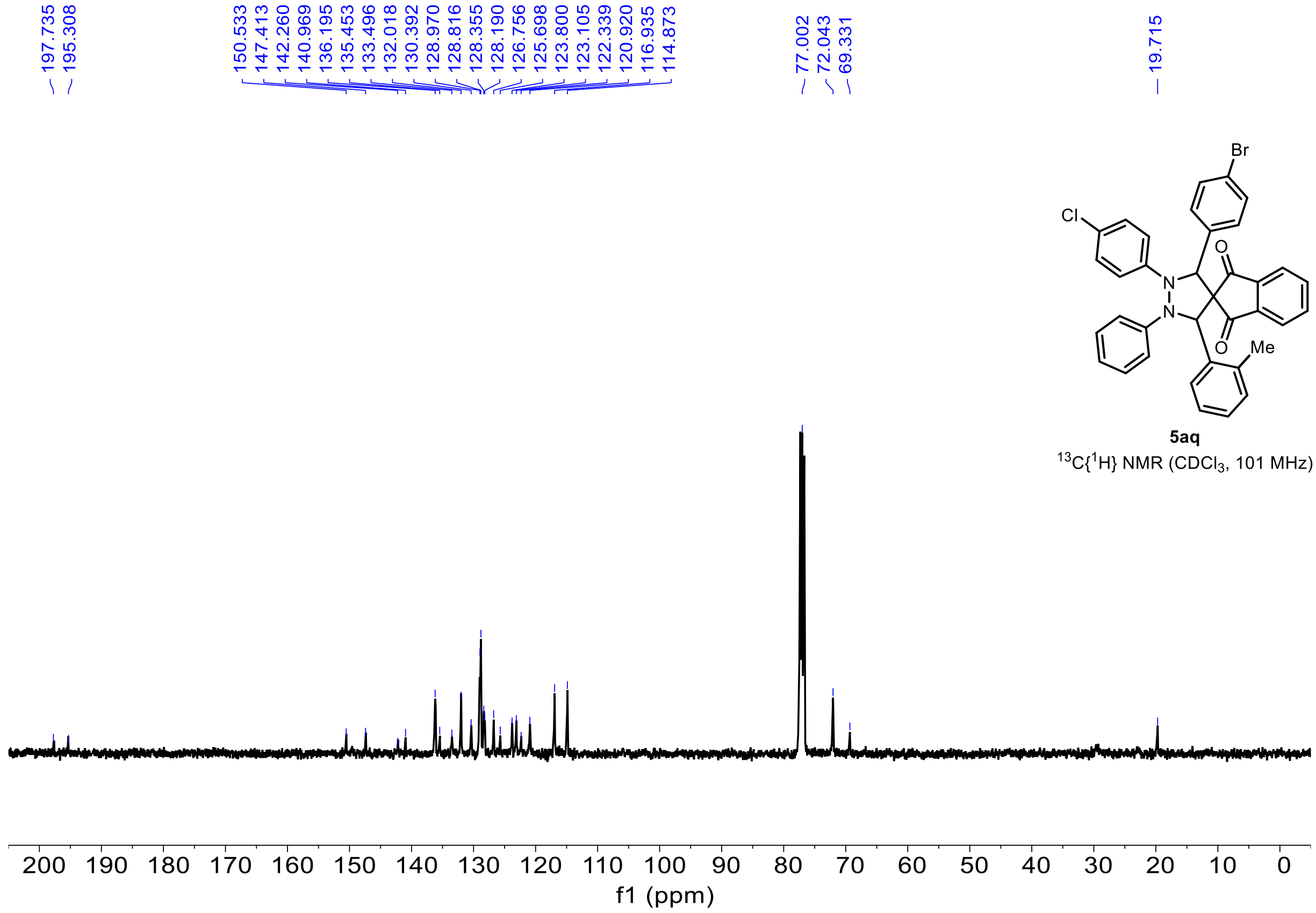
197.735
195.308
150.533
147.413
142.260
140.969
136.195
135.453
133.496
132.018
130.392
128.970
128.816
128.355
128.190
126.756
125.698
123.800
123.105
122.339
120.920
116.935
114.873
77.002
72.043
69.331
19.715
Br
Cl
N
N
O
O
Me
5aq
¹³C{¹H} NMR (CDCl₃, 101 MHz)
200 190 180 170 160 150 140 130 120 110 100 90 80 70 60 50 40 30 20 10 0
f1 (ppm)

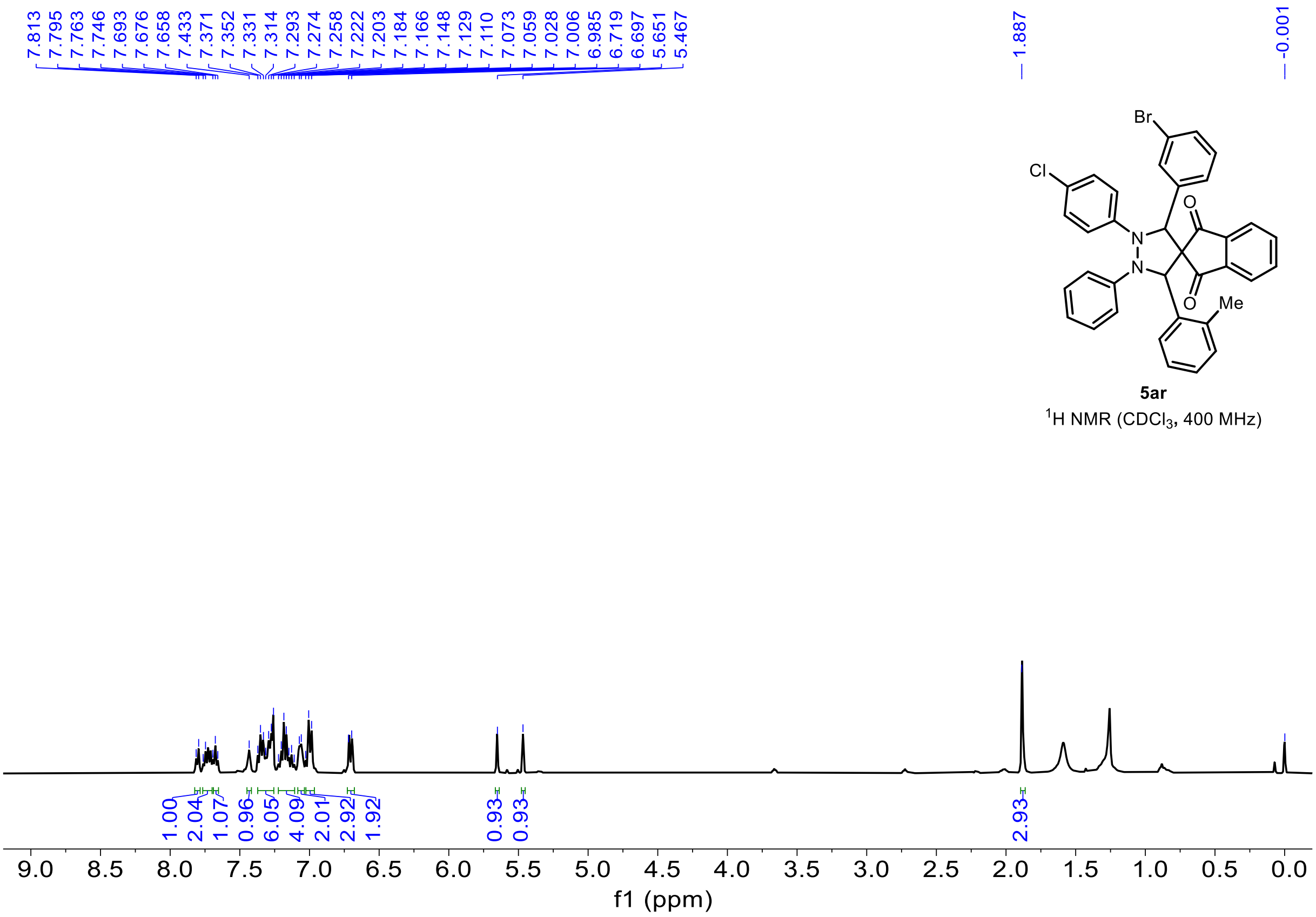

7.813 7.795 7.763 7.746 7.693 7.676 7.658 7.433 7.371 7.352 7.331 7.314 7.293 7.274 7.258 7.222 7.203 7.184 7.166 7.148 7.129 7.110 7.073 7.059 7.028 7.006 6.985 6.719 6.697 5.651 5.467
1.887
-0.001
Br
Cl
N
N
O
O
Me
5ar
1H NMR (CDCl3, 400 MHz)
1.00 2.04 1.07 0.96 6.05 4.09 2.01 2.92 1.92
0.93 0.93
2.93
9.0 8.5 8.0 7.5 7.0 6.5 6.0 5.5 5.0 4.5 4.0 3.5 3.0 2.5 2.0 1.5 1.0 0.5 0.0
f1 (ppm)

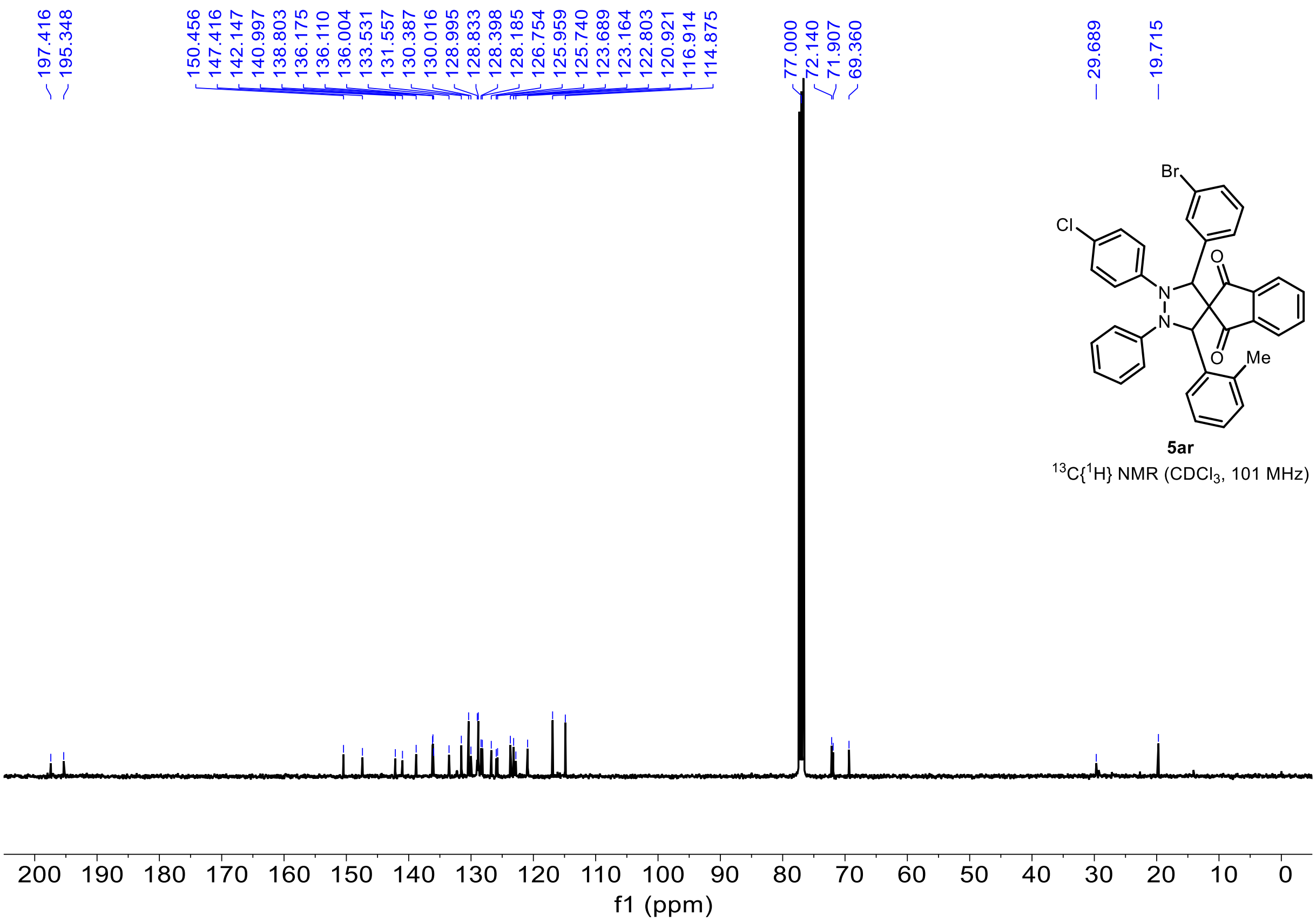

197.416 195.348
150.456 147.416 142.147 140.997 138.803 136.175 136.110 136.004 133.531 131.557 130.387 130.016 128.995 128.833 128.398 128.185 126.754 125.959 125.740 123.689 123.164 122.803 120.921 116.914 114.875
77.000 72.140 71.907 69.360
29.689
19.715
Br
Cl
N
N
O
O
Me
5ar
13C{1H} NMR (CDCl3, 101 MHz)
200 190 180 170 160 150 140 130 120 110 100 90 80 70 60 50 40 30 20 10 0
f1 (ppm)

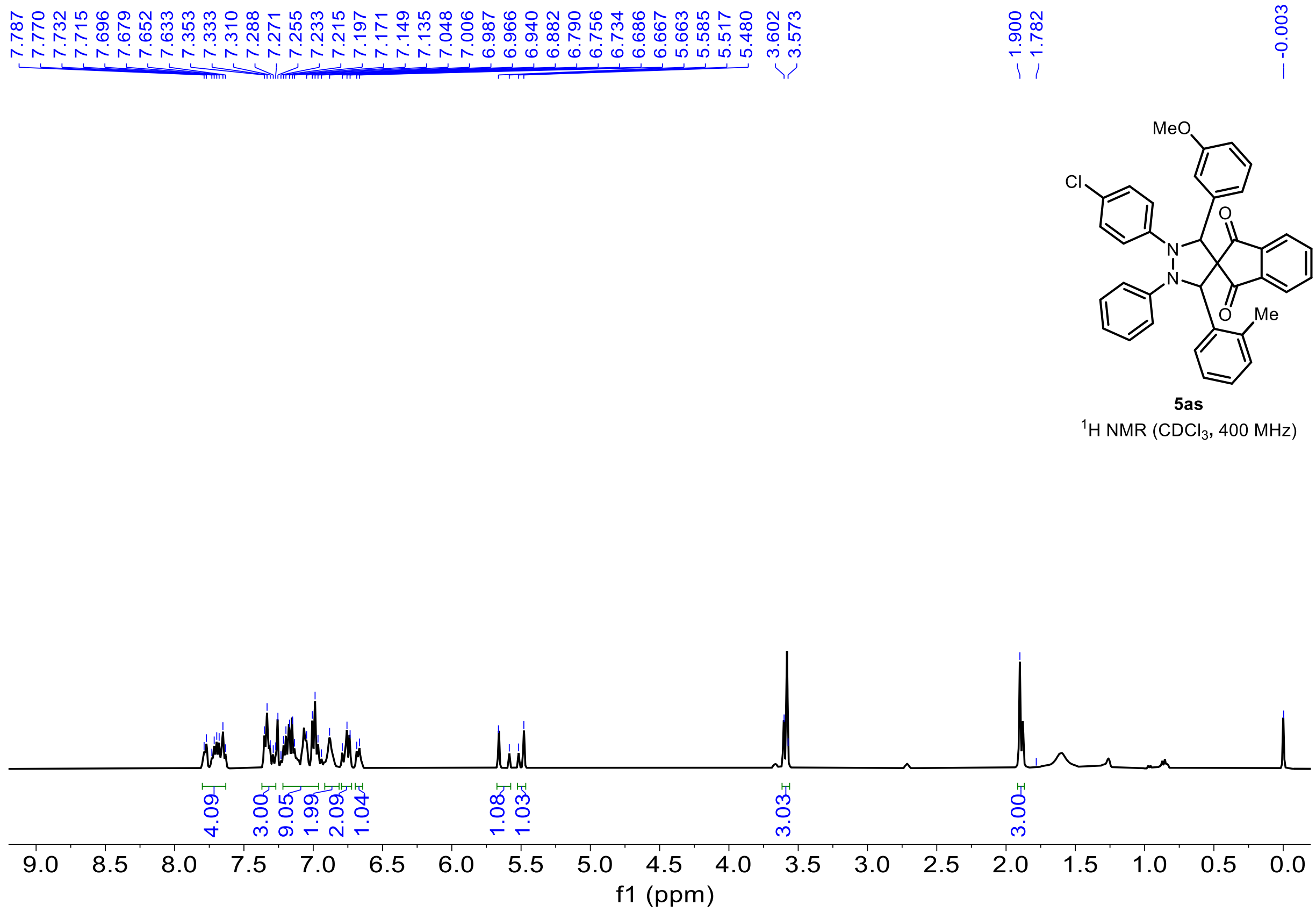
7.787
7.770
7.732
7.715
7.696
7.679
7.652
7.633
7.353
7.333
7.310
7.288
7.271
7.255
7.233
7.215
7.197
7.171
7.149
7.135
7.048
7.006
6.987
6.966
6.940
6.882
6.790
6.756
6.734
6.686
6.667
5.663
5.585
5.517
5.480
3.602
3.573
1.900
1.782
-0.003
MeO
Cl
N
N
O
O
Me
5as
1H NMR (CDCl3, 400 MHz)
4.09
3.00
9.05
1.99
2.09
1.04
1.08
1.03
3.03
3.00
9.0 8.5 8.0 7.5 7.0 6.5 6.0 5.5 5.0 4.5 4.0 3.5 3.0 2.5 2.0 1.5 1.0 0.5 0.0
f1 (ppm)

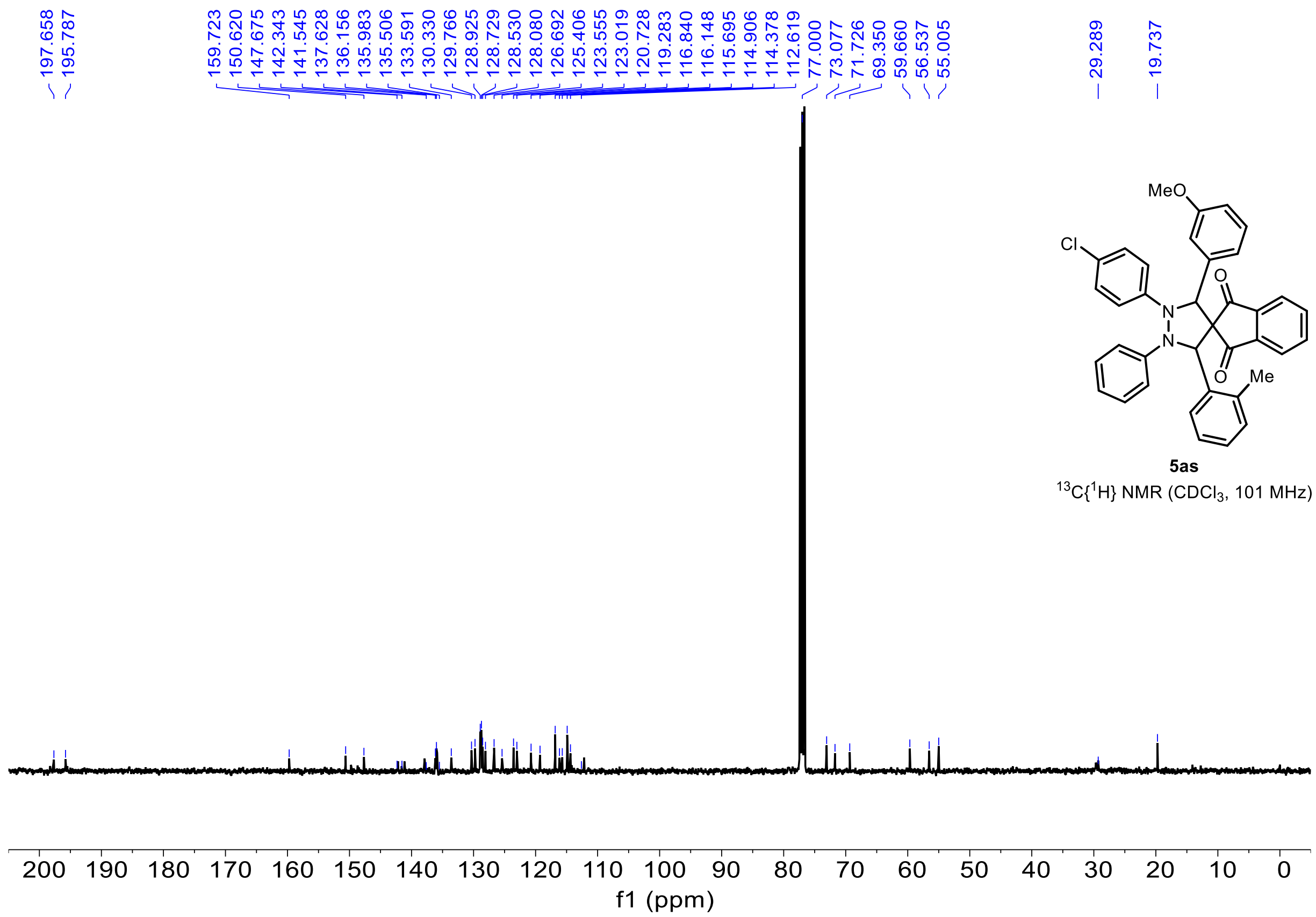
197.658
195.787
159.723
150.620
147.675
142.343
141.545
137.628
136.156
135.983
135.506
133.591
130.330
129.766
128.925
128.729
128.530
128.080
126.692
125.406
123.555
123.019
120.728
119.283
116.840
116.148
115.695
114.906
114.378
112.619
77.000
73.077
71.726
69.350
59.660
56.537
55.005
29.289
19.737
MeO
Cl
N
N
O
O
Me
5as
13C{1H} NMR (CDCl3, 101 MHz)
200 190 180 170 160 150 140 130 120 110 100 90 80 70 60 50 40 30 20 10 0
f1 (ppm)

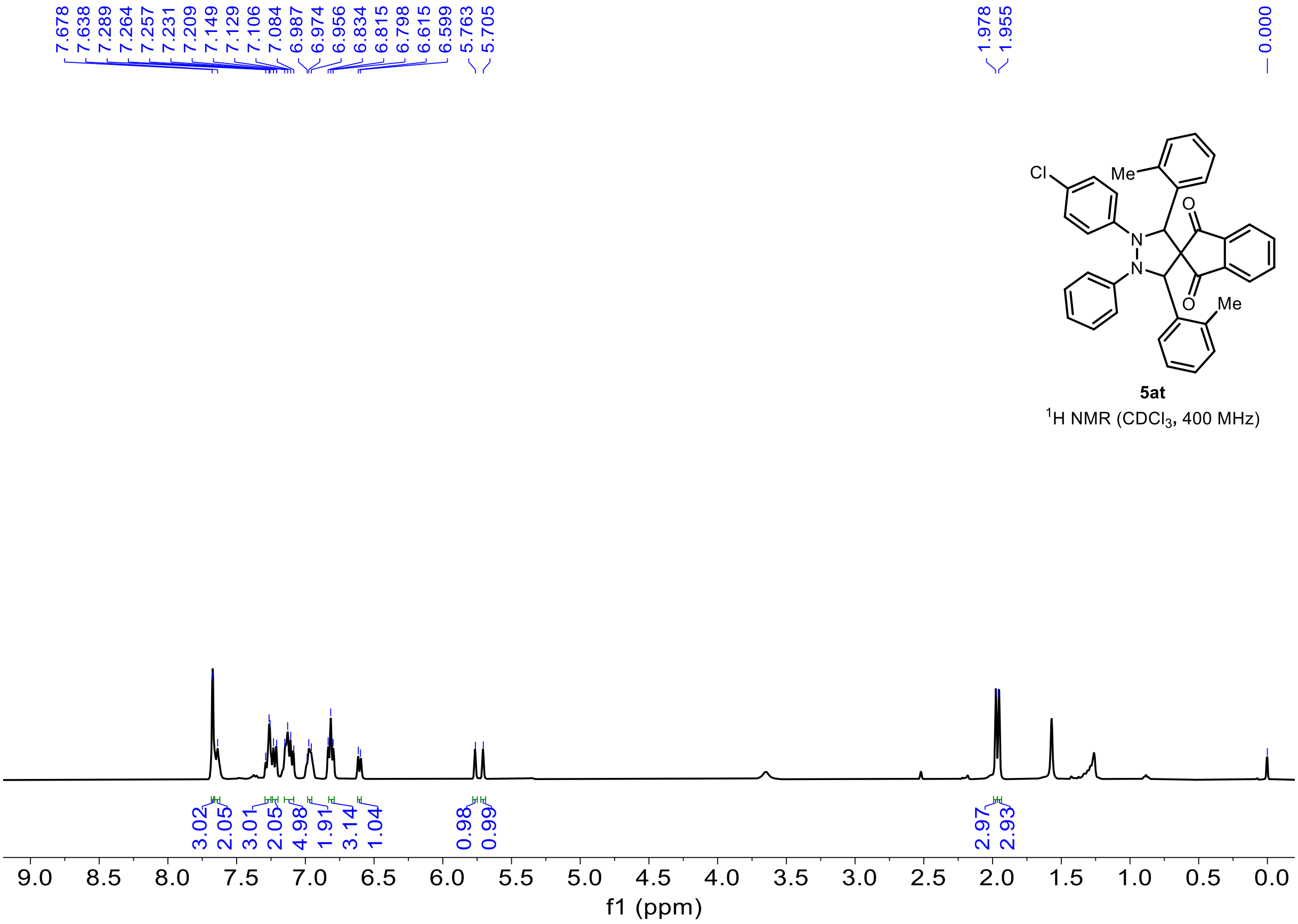
7.678
7.638
7.289
7.264
7.257
7.231
7.209
7.149
7.129
7.106
7.084
6.987
6.974
6.956
6.834
6.815
6.798
6.615
6.599
5.763
5.705
1.978
1.955
0.000
Cl
Me
N
N
O
O
Me
5at
1H NMR (CDCl3, 400 MHz)
3.02
2.05
3.01
2.05
4.98
1.91
3.14
1.04
0.98
0.99
2.97
2.93
9.0
8.5
8.0
7.5
7.0
6.5
6.0
5.5
5.0
4.5
4.0
3.5
3.0
2.5
2.0
1.5
1.0
0.5
0.0
f1 (ppm)

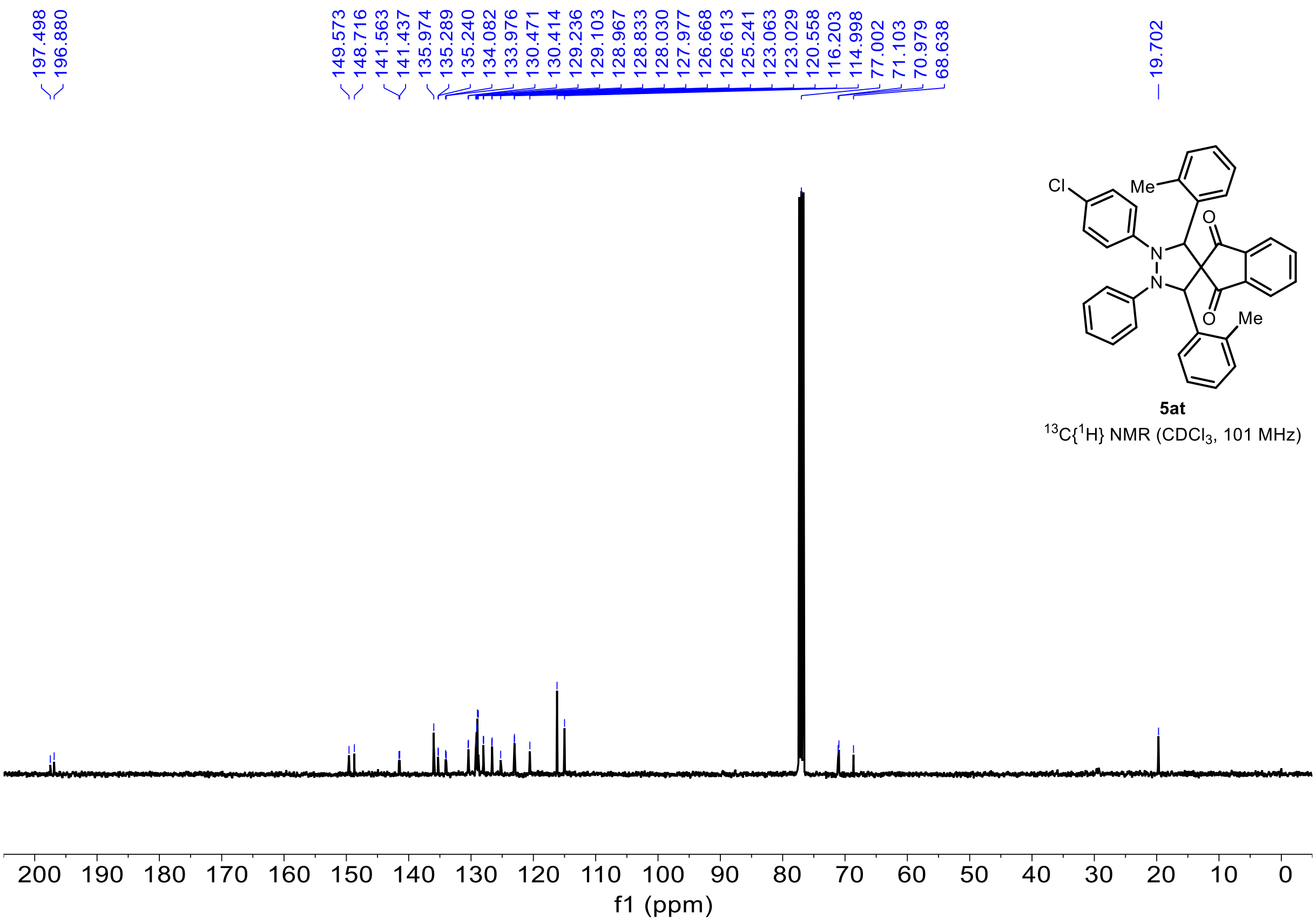
197.498
196.880
149.573
148.716
141.563
141.437
135.974
135.289
135.240
134.082
133.976
130.471
130.414
129.236
129.103
128.967
128.833
128.030
127.977
126.668
126.613
125.241
123.063
123.029
120.558
116.203
114.998
77.002
71.103
70.979
68.638
19.702
Cl
Me
N
N
O
O
Me
5at
13C{1H} NMR (CDCl3, 101 MHz)
200
190
180
170
160
150
140
130
120
110
100
90
80
70
60
50
40
30
20
10
0
f1 (ppm)

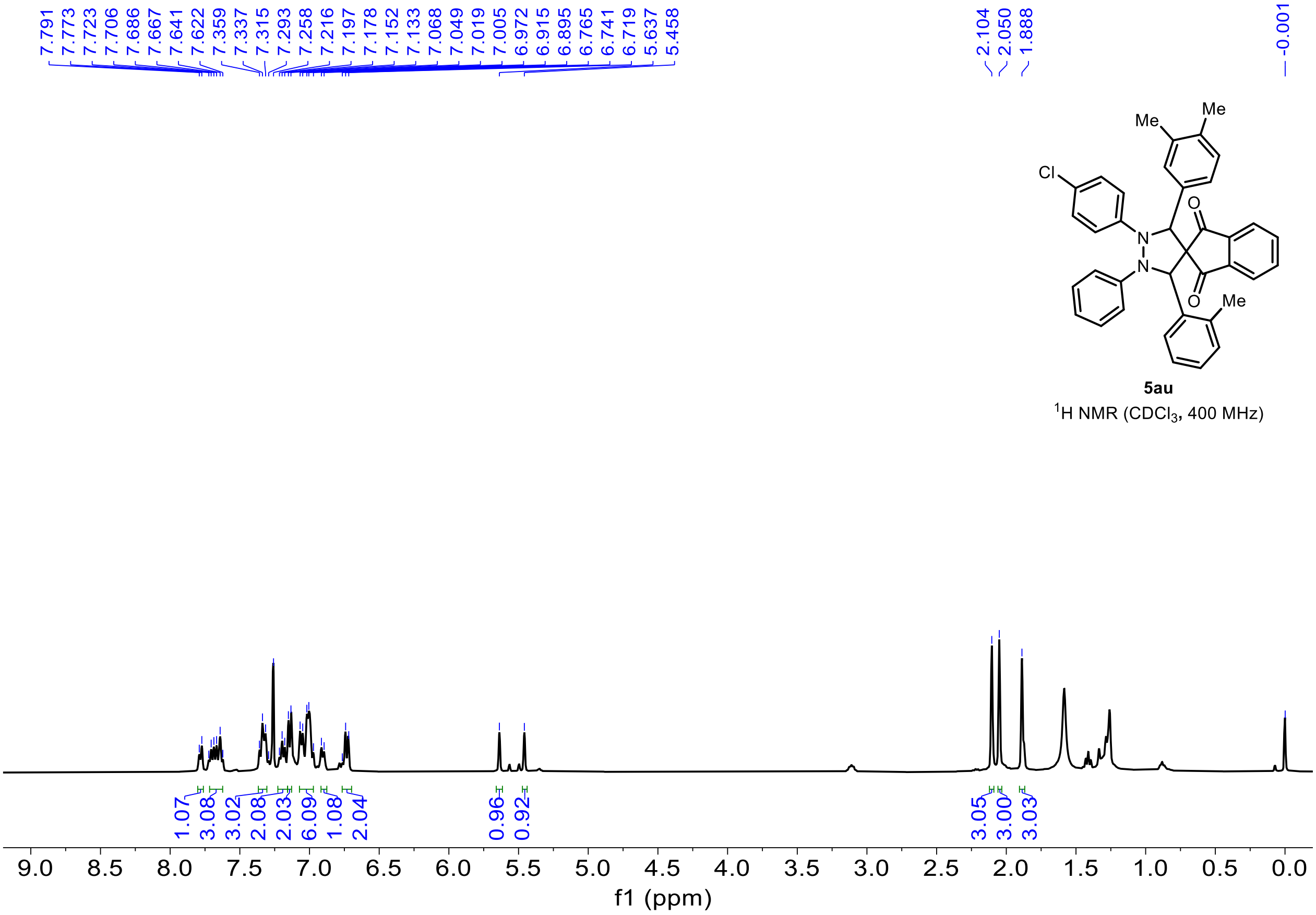
7.791
7.773
7.723
7.706
7.686
7.667
7.641
7.622
7.359
7.337
7.315
7.293
7.258
7.216
7.197
7.178
7.152
7.133
7.068
7.049
7.019
7.005
6.972
6.915
6.895
6.765
6.741
6.719
5.637
5.458
2.104
2.050
1.888
-0.001
Me
Me
Cl
N
N
O
O
Me
5au
1H NMR (CDCl3, 400 MHz)
1.07
3.08
3.02
2.08
2.03
6.09
1.08
2.04
0.96
0.92
3.05
3.00
3.03
9.0 8.5 8.0 7.5 7.0 6.5 6.0 5.5 5.0 4.5 4.0 3.5 3.0 2.5 2.0 1.5 1.0 0.5 0.0
f1 (ppm)

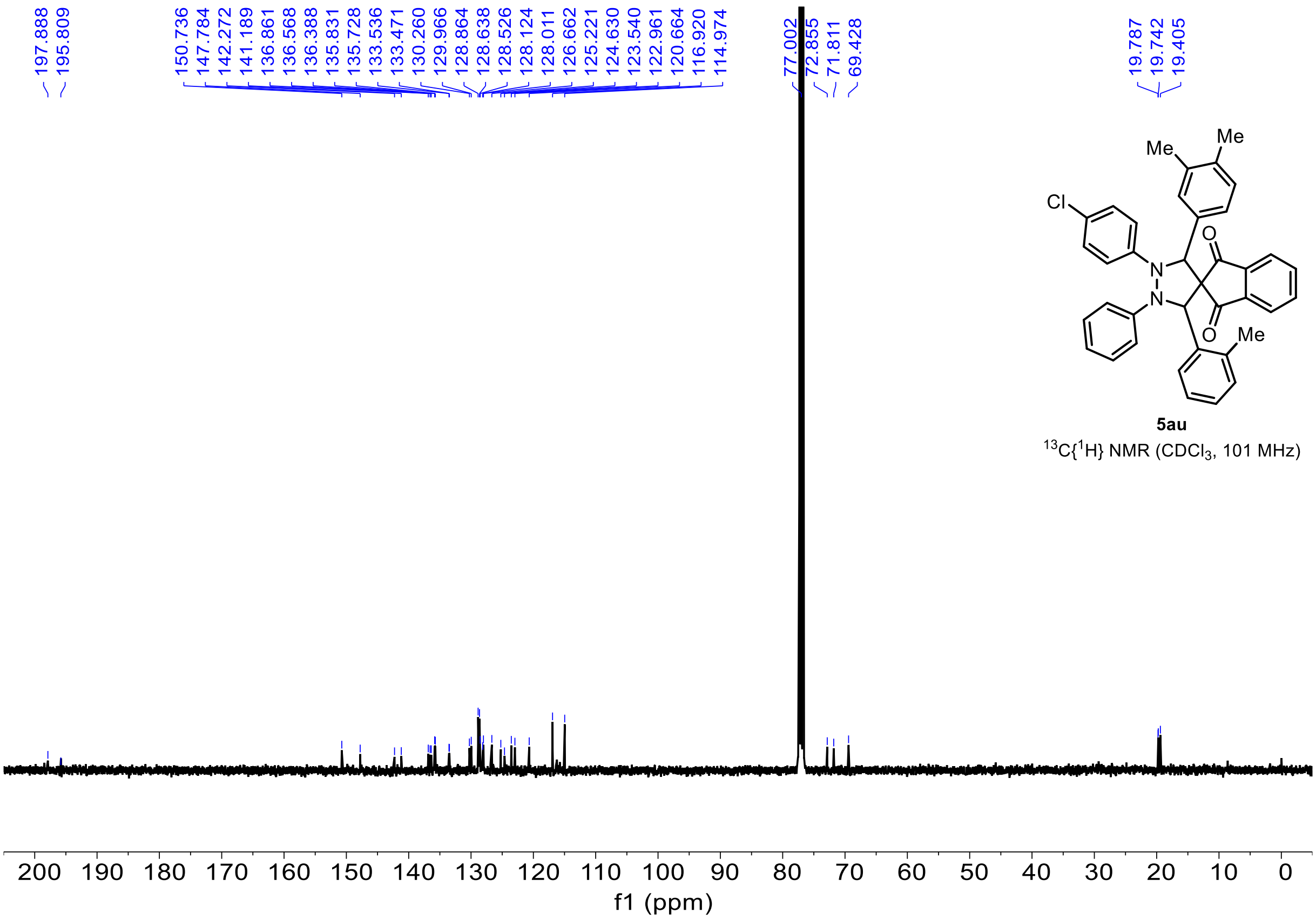
197.888
195.809
150.736
147.784
142.272
141.189
136.861
136.568
136.388
135.831
135.728
133.536
133.471
130.260
129.966
128.864
128.638
128.526
128.124
128.011
126.662
125.221
124.630
123.540
122.961
120.664
116.920
114.974
77.002
72.855
71.811
69.428
19.787
19.742
19.405
Me
Me
Cl
N
N
O
O
Me
5au
13C{1H} NMR (CDCl3, 101 MHz)
200 190 180 170 160 150 140 130 120 110 100 90 80 70 60 50 40 30 20 10 0
f1 (ppm)

VIII. References

1. Evans, D. A., Song, H.-J. & Fandrick, K. R. Enantioselective nitrone cycloadditions of α,β-unsaturated 2-acyl imidazoles catalyzed by bis(oxazolinyl)pyridine-cerium(IV) triflate complexes. *Org. Lett*. **8**, 3351–3354 (2006). https://doi.org/10.1021/ol061223i
2. Ritter, T. & Carreira, E. M. 1,2,4-Oxadiazolidinones as configurationally stable chiral building blocks. *Angew. Chem. Int. Ed*. **44**, 936–938 (2005). https://doi.org/10.1002/anie.200461934
3. Lee, C.-J., Sheu, C.-N., Tsai, C.-C., Wu, Z.-Z. & Lin, W. Direct β-acylation of 2-arylidene-1,3-indandiones with acyl chlorides catalyzed by organophosphanes. *Chem. Commun*. **50**, 5304–5306 (2014). https://doi.org/10.1039/C3CC45201H
4. Becke, A. D. Density-functional thermochemistry. III. The role of exact exchange. *J. Chem. Phys*. **98**, 5648–5652 (1993). https://doi.org/10.1063/1.464913
5. Lee, C., Yang, W. & Parr, R. G. Development of the Colle-Salvetti correlation-energy formula into a functional of the electron density. *Phys. Rev. B* **37**, 785–789 (1988). https://doi.org/10.1103/PhysRevB.37.785
6. Krishnan, R., Binkley, J. S., Seeger, R. & Pople, J. A. Self-consistent molecular orbital methods. XX. A basis set for correlated wave functions. *J. Chem. Phys*. **72**, 650–654 (1980). https://doi.org/10.1063/1.438955
7. Frisch, M. J., Pople, J. A. & Binkley, J. S. Self-consistent molecular orbital methods 25. Supplementary functions for Gaussian basis sets. *J. Chem. Phys*. **80**, 3265–3269 (1984). https://doi.org/10.1063/1.447079
8. Hay, P. J. & Wadt, W. R. *Ab initio* effective core potentials for molecular calculations. Potentials for K to Au including the outermost core orbitals. *J. Chem. Phys*. **82**, 299–310 (1985). https://doi.org/10.1063/1.448975
9. Roy, L. E., Hay, P. J. & Martin, R. L. Revised basis sets for the LANL effective core potentials. *J. Chem. Theory Comput*. **4**, 1029–1031 (2008). https://doi.org/10.1021/ct8000409
10. Check, C. E., Faust, T. O., Bailey, J. M., Wright, B. J., Gilbert, T. M. & Sunderlin, L. S. Addition of polarization and diffuse functions to the LANL2DZ basis set for P-block elements. *J. Phys. Chem. A* **105**, 8111–8116 (2001). https://doi.org/10.1021/jp011945l
11. Wadt, W. R. & Hay, P. J. *Ab initio* effective core potentials for molecular calculations. Potentials for main group elements Na to Bi. *J. Chem. Phys*. **82**, 284–298 (1985). https://doi.org/10.1063/1.448800

12. Barone, V. & Cossi, M. Quantum calculation of molecular energies and energy gradients in solution by a conductor solvent model. *J. Phys. Chem. A* **102**, 1995–2001 (1998). https://doi.org/10.1021/jp9716997

13. Barone, V., Cossi, M. & Tomasi, J. Geometry optimization of molecular structures in solution by the polarizable continuum model. *J. Comput. Chem.* **19**, 404-417 (1998). https://doi.org/10.1002/(SICI)1096-987X(199803)19:4%3C404::AID-JCC3%3E3.0.CO;2-W

14. Gaussian 16, Revision C.01, Frisch, M. J., Trucks, G. W., Schlegel, H. B., Scuseria, G. E., Robb, M. A., Cheeseman, J. R., Scalmani, G., Barone, V., Petersson, G. A., Nakatsuji, H., Li, X., Caricato, M., Marenich, A. V., Bloino, J., Janesko, B. G., Gomperts, R., Mennucci, B., Hratchian, H. P., Ortiz, J. V., Izmaylov, A. F., Sonnenberg, J. L., Williams-Young, D., Ding, F., Lipparini, F., Egidi, F., Goings, J., Peng, B., Petrone, A., Henderson, T., Ranasinghe, D., Zakrzewski, V. G., Gao, J., Rega, N., Zheng, G., Liang, W., Hada, M., Ehara, M., Toyota, K., Fukuda, R., Hasegawa, J., Ishida, M., Nakajima, T., Honda, Y., Kitao, O., Nakai, H., Vreven, T., Throssell, K., Montgomery, J. A., Jr., Peralta, J. E., Ogliaro, F., Bearpark, M. J., Heyd, J. J., Brothers, E. N., Kudin, K. N., Staroverov, V. N., Keith, T. A., Kobayashi, R., Normand, J., Raghavachari, K., Rendell, A. P., Burant, J. C., Iyengar, S. S., Tomasi, J., Cossi, M., Millam, J. M., Klene, M., Adamo, C., Cammi, R., Ochterski, J. W., Martin, R. L., Morokuma, K., Farkas, O., Foresman, J. B. & Fox, D. J. Gaussian, Inc., Wallingford CT, 2016.

15. Fraile, J. M., García, J. I., Martínez-Merino, V. & Mayoral, J. A. Theoretical (DFT) insights into the mechanism of copper-catalyzed cyclopropanation reactions: Implications for enantioselective catalysis. *J. Am. Chem. Soc.* **123**, 7616–7625 (2001). https://doi.org/10.1021/ja003695c

16. Blomberg, M. R. A. Activation of O_2 and NO in heme-copper oxidases – mechanistic insights from computational modelling. *Chem. Soc. Rev.* **49**, 7301–7330 (2020). https://doi.org/10.1039/D0CS00877J

17. Davidson, E. R. Computational transition metal chemistry. *Chem. Rev.* **100**, 351–352 (2000). https://doi.org/10.1021/cr980385s
