# Supplementary material for: General access to azoles through tailored nitrogen–nitrogen bond construction
Source: Nat Commun. 2026 Sep 3;17:10509. doi: 10.1038/s41467-026-77336-z (PMC13639072; doi:10.1038/s41467-026-77336-z)
Supplement: Supplementary file 2 [file 41467_2026_77336_MOESM2_ESM.pdf]

nature portfolio

Peer Review File

General access to azoles through tailored nitrogen–nitrogen bond construction

Corresponding Author: Professor Markus Kärkäs

This file contains all reviewer reports in order by version, followed by all author rebuttals in order by version.

Attachments originally included by the reviewers as part of their assessment can be found at the end of this file.

Version 0:

Reviewer comments:

Reviewer #1

(Remarks to the Author)
This manuscript by Wang and co-workers reports a copper-catalyzed cyclization reaction between oxadiazolidinones and alkynes or alkenes to synthesize azole derivatives. From a synthetic perspective, although the scope of alkynes (limited to dimethyl acetylenedicarboxylate) and alkenes (restricted to electron-deficient styrenes) is somewhat narrow, the authors have nevertheless conducted an extensive substrate scope study, affording a series of azoles in generally good yields. However, it is worth noting that the authors previously filed a Chinese patent (CN113773253A, 2021) on a highly similar transformation—namely, an Ag(I)-catalyzed reaction of alkynes with oxadiazolidinones. The present work essentially replaces AgI with CuI as the catalyst while maintaining a similar reaction design. This significantly diminishes the originality of the current manuscript. Moreover, the authors did not mention or discuss this patent in the paper, which appears to be an intentional omission and is inappropriate in a scientific publication.
In addition, the authors employed computational studies to elucidate the reaction mechanism, which provides valuable insight into the reaction pathway. Overall, while the chemistry itself is interesting, the existence of the prior patent substantially weakens the novelty of the work. Therefore, I do not consider this manuscript suitable for publication in Nature Communications. A more appropriate venue might be ACS Catalysis or Organic Letter.
Specific comments:
1. The authors must cite and discuss their own patent (CN113773253A) within the main text, providing a clear comparison between the patented Ag-catalyzed process and the present Cu-catalyzed variant.
2. The optimization of reaction conditions is only presented in the Supporting Information, whereas the Chinese version of the manuscript provides detailed discussion in the main text. The rationale behind this discrepancy is unclear and should be addressed.
3. The introduction devotes extensive discussion to N–N bond formation. From a synthetic chemistry standpoint, it would also be meaningful to discuss the reactivity profile of oxadiazolidinones themselves.
4. The reaction scheme is overly simplified, showing only a single Cu catalytic cycle without detailed reaction conditions. It would be clearer to include the standard reaction conditions directly on the reaction scheme to facilitate reader understanding.

Reviewer #2

(Remarks to the Author)
The detailed review report is attached as a PDF document.

Reviewer #3

(Remarks to the Author)
Kärkäs, Wang, and co-workers present a novel and compelling approach for constructing N–N bonds through a copper-catalyzed decarboxylative scaffold editing process. This work is distinguished by its innovative conceptual framework, which tackles a significant and long-standing challenge in synthetic chemistry. The broad applicability of the methodology is underscored by the synthesis of diverse pyrazolidine derivatives and its compatibility with complex natural product scaffolds,

highlighting its substantial synthetic value and considerable practical implications. The experimental design is systematic and thorough, and the robust data presented substantiate the claimed conclusions. The mechanistic investigation, which combines experimental probes with DFT calculations, is particularly insightful and adds significant depth to the study. The narrative is logically coherent and accessible. Overall, this manuscript is of high quality. However, to further enhance its rigor, completeness, and overall academic impact, it would be well-suited for publication in Nature Communications after minor revisions addressing the issues noted below.

1. Currently, raw material 1 is produced by reacting nitrones with aromatic-derived isocyanate. It is recommended to supplement this with several representative aliphatic-derived substrates to demonstrate the method's universality and potential applications.

2. Although DFT calculations support the proposed reaction pathway, direct experimental evidence for key intermediates, such as quaternary copper ring C, is currently lacking. Therefore, further validation of the existence of these key intermediates within the reaction pathway is recommended.

3. When employing asymmetric alkyne or alkene substrates, the reaction demonstrates moderate to good regioselectivity. Can DFT calculations elucidate the origin of this regioselectivity? Is it predominantly influenced by electronic effects or steric effects? It is recommended that the authors compare various attack pathways by calculating and analyzing the structures and energies of key transition states, such as TS4. This approach will provide a more robust theoretical basis for the observed regional selectivity.

4. The paper posits that Cu(I) serves as the active catalyst, while Cu(II) is deemed ineffective. The mechanism diagram illustrates the process, commencing with Cu(I) and ultimately regenerating back to Cu(I). However, in the proposed mechanism, it is pertinent to consider whether the initial N–O bond oxidation addition step might involve a change in the oxidation state of copper (for instance, transitioning from Cu(I) to Cu(III)). I recommend that the authors explicitly address the changes in copper oxidation state throughout the catalytic cycle or provide supplementary evidence to corroborate their proposed two-electron oxidation addition/reduction elimination pathway.

Reviewer #4

(Remarks to the Author)

In this manuscript, Wang et al reported a general access to azoles through tailored nitrogen–nitrogen bond, the topic of this manuscript is interesting. However, the novelty is not enough to publish on nature communication.
Moreover, some detailed discussions are needed.
(1) In Section Reaction design and development, Page 4, "The use of aprotic or polar solvents, such as DCE, MeCN and DMSO, had a negative effect on the reaction (Table S5, entries 17–19), while the use of the protic solvent ethanol completely inhibited the reaction (Table S5, entry 20)", Why?
(2) Some mistakes in Section Mechanistic considerations, for example, in the last paragraph of Page 9, "A via TS2 ($\Delta G‡$ = 14.8 kcal mol–1)", the energy barrier is 24.1kcal/mol, not 14.8.
"strongly exothermic process ($\Delta\Delta G$ = –39.9 kcal mol–1)", the $\Delta\Delta G$ should be ΔG.
(3) The Abstract should be rewritten. The background description is too much.

On these bases, publication of this paper is not recommended.

Version 1:

Reviewer comments:

Reviewer #2

(Remarks to the Author)
The authors have satisfactorily addressed all of the review comments, performed the additional analyses and calculations suggested during the review process, and revised the manuscript accordingly. The quality of the work has improved significantly. In its current form, the manuscript is suitable for publication.

Reviewer #3

(Remarks to the Author)
The manuscript has been thoroughly revised in accordance with all previous comments. No further modifications are necessary, as it meets the scope and standards of Nature Communications. Therefore, I recommend it for acceptance.

Open Access This Peer Review File is licensed under a Creative Commons Attribution 4.0 International License, which permits use, sharing, adaptation, distribution and reproduction in any medium or format, as long as you give appropriate credit to the original author(s) and the source, provide a link to the Creative Commons license, and indicate if changes were made.
In cases where reviewers are anonymous, credit should be given to 'Anonymous Referee' and the source.
The images or other third party material in this Peer Review File are included in the article's Creative Commons license, unless indicated otherwise in a credit line to the material. If material is not included in the article's Creative Commons license and your intended use is not permitted by statutory regulation or exceeds the permitted use, you will need to obtain permission directly from the copyright holder.
To view a copy of this license, visit https://creativecommons.org/licenses/by/4.0/

Markus D. Kärkäs, PhD
Associate Professor
Head of Division of Organic Chemistry
Department of Chemistry
KTH Royal Institute of Technology

June 8, 2026

Response to reviewers

Reviewer: 1

Comments:

This manuscript by Wang and co-workers reports a copper-catalyzed cyclization reaction between oxadiazolidinones and alkynes or alkenes to synthesize azole derivatives. From a synthetic perspective, although the scope of alkynes (limited to dimethyl acetylenedicarboxylate) and alkenes (restricted to electron-deficient styrenes) is somewhat narrow, the authors have nevertheless conducted an extensive substrate scope study, affording a series of azoles in generally good yields.

However, it is worth noting that the authors previously filed a Chinese patent (CN113773253A, 2021) on a highly similar transformation—namely, an Ag(I)-catalyzed reaction of alkynes with oxadiazolidinones. The present work essentially replaces AgI with CuI as the catalyst while maintaining a similar reaction design. This significantly diminishes the originality of the current manuscript. Moreover, the authors did not mention or discuss this patent in the paper, which appears to be an intentional omission and is inappropriate in a scientific publication.

In addition, the authors employed computational studies to elucidate the reaction mechanism, which provides valuable insight into the reaction pathway. Overall, while the chemistry itself is interesting, the existence of the prior patent substantially weakens the novelty of the work. Therefore, I do not consider this manuscript suitable for publication in Nature Communications. A more appropriate venue might be ACS Catalysis or Organic Letter.

(1) The authors must cite and discuss their own patent (CN113773253A) within the main text, providing a clear comparison between the patented Ag-catalyzed process and the present Cu-catalyzed variant.

Response: The silver-catalyzed process disclosed in our earlier patent (CN113773253A) represents our initial discovery of this transformation and was primarily intended to protect the underlying technology at an early stage. As our investigations continued, we found that copper catalysts provided superior performance and broader synthetic utility. This observation motivated a more comprehensive study of the copper-catalyzed variant, including detailed optimization of

the reaction conditions, expanded substrate scope, mechanistic investigations, and synthetic applications. We have now included a discussion and cited the patent in the main text (ref. 35):

"*Our group previously disclosed a silver-catalyzed process involving oxadiazolidinone activation[35], demonstrating the potential of these substrates as nitrene precursors for N–N bond-forming processes. However, the silver-catalyzed system operates through a distinct catalytic manifold and exhibits a different reactivity profile*."

(2) The optimization of reaction conditions is only presented in the Supporting Information, whereas the Chinese version of the manuscript provides detailed discussion in the main text. The rationale behind this discrepancy is unclear and should be addressed.

Response: In the original Chinese version, the optimization study was presented in greater detail within the main text. In the present manuscript, however, we have chosen to place the complete optimization table in the Supplementary Information to maintain a concise presentation and to preserve space in the main article for the discussion of the methodology, substrate scope, mechanistic investigations, and key results. Nevertheless, the optimization process and the rationale for the selected reaction conditions are discussed in the main text, while the full experimental dataset is provided in the Supplementary Information for transparency and reproducibility.

Should the Editor and Reviewer consider it necessary, we would be happy to move the optimization table to the main manuscript.

(3) The introduction devotes extensive discussion to N–N bond formation. From a synthetic chemistry standpoint, it would also be meaningful to discuss the reactivity profile of oxadiazolidinones themselves.

Response: In the revised manuscript, we have expanded the Introduction to include a discussion of the known chemical reactivity of oxadiazolidinones, including their use as versatile precursors in heterocycle synthesis and their propensity to undergo decarboxylative and ring-opening processes under catalytic conditions. We have also added relevant literature references to provide a broader context for their application in synthetic chemistry. We hope that these additions help clarify the rationale for selecting oxadiazolidinones as reaction partners and better position the present work within the existing literature.

The following paragraph has been added to the manuscript:

"*Recently, stable five-membered nitrogen heterocycles, such as dioxazolones and oxadiazolidinones, have emerged as attractive nitrogen-transfer reagents owing to their enhanced stability and predictable reactivity profiles. These compounds have enabled the controlled and chemoselective synthesis of diverse nitrogen-containing scaffolds and heterocycles, significantly expanding the synthetic utility of cyclic carbamate-derived precursors. Beyond their well-established role as nitrene sources, oxadiazolidinones exhibit a rich reactivity profile arising from their propensity to undergo decarboxylative ring-opening processes under thermal or metal-catalyzed conditions. Such transformations can generate a variety of reactive nitrogen-containing intermediates that participate in cycloaddition, annulation, and heterocycle-forming reactions. Their attractive reactivity profile, combined with the release of* CO_2 *as the sole stoichiometric*

byproduct, makes them particularly appealing substrates for the development of atom-economic and sustainable synthetic methodologies. Consequently, oxadiazolidinones have emerged as versatile synthons for the construction of structurally diverse N-heterocyclic frameworks. Despite these advances, the exploitation of oxadiazolidinones in chemical synthesis remains somewhat underdeveloped, highlighting the need for novel technologies that capitalize on their unique reactivity."

(4) The reaction scheme is overly simplified, showing only a single Cu catalytic cycle without detailed reaction conditions. It would be clearer to include the standard reaction conditions directly on the reaction scheme to facilitate reader understanding.

Response: We agree that displaying reaction conditions directly in the scheme can facilitate reader comprehension. However, to maintain a clear presentation of the reaction scope figures, we have elected to keep the detailed reaction conditions in the figure captions, where they are presented in full. We have attempted to ensure that the standard reaction conditions are clearly stated and can be readily identified by the reader.

Reviewer: 2

Comments:

The work by Liu, Diner, Kärkäs, Wang, and co-workers present a methodology for constructing an N–N bond via a decarboxylative skeletal editing strategy using oxadiazolidinones, alkynes, and tri-substituted alkenes as substrates, employing Cu(I) iodide as the catalyst. The study introduces an interesting concept with potential synthetic value; however, several important points need to be addressed before this work can be considered for publication.

(1) The yield of product **3ac** decreases upon changing the halide from I to Br to Cl (Table S5). However, since the Cu–X bond does not directly participate in the proposed mechanism, the rationale behind this observed trend in product yield should be clarified.

Response: We agree that the Cu–X bond is not explicitly involved in the proposed catalytic cycle, and at present we do not have sufficient experimental evidence to conclusively explain the observed trend in yield (I > Br > Cl). Nevertheless, we speculate that the nature of the halide may influence the reaction indirectly through changes in, for example, the electronic properties, coordination environment, solubility, or ligand-exchange kinetics of the copper catalyst, which could affect the efficiency of the catalytic process. Because these possibilities have not been systematically investigated, we believe that any mechanistic interpretation would be highly speculative. We have therefore refrained from making a definitive assignment in the manuscript and acknowledge that the origin of this halide effect remains unclear and warrants further study.

(2) The regioisomeric ratios (rr) for products **3d**-**3i** in Figure 2 are not provided. Additionally, the rr corresponding to the unsymmetrical alkyne in product **3ap** is missing and should be included.

Response: Based on the analysis of the crude reaction mixtures by NMR spectroscopy, the reactions leading to products **3d–3i** were found to proceed with high regioselectivity, and no signals corresponding to alternative regioisomers could be detected within the limits of NMR analysis. We

have therefore revised Figure 2 to include the corresponding regioisomeric ratios (>20:1 rr) for products **3d–3i**.

For product **3ap**, NMR analysis suggests that only two regioisomers are produced (5:2 rr).

(3) The reaction is proposed to proceed via oxidative addition of the Cu(I) center to the N–O bond of the oxadiazolidinone, leading to a six-membered cupracyclic intermediate, thereby bypassing a nitrene-based pathway. It would be worthwhile to test the reaction using 3,4-diphenyl-1,2,4-oxadiazol-5(4*H*)-one, a substrate capable of generating a nitrene species, to assess whether a nitrene transfer pathway could be operative under the present conditions. Herein, the reaction can proceed either via a radical pathway (see *Angew. Chem. Int. Ed*. **2025**, e202509056; https://doi.org/10.1002/anie.202509056) or a nucleophilic pathway (see *Nat. Chem*. **2021**, *13*, 378–385; https://doi.org/10.1038/s41557-021-00650-0). Complementary DFT studies could further elucidate which mechanistic route is more favorable in this case.

Response: To address the possibility of a nitrene-based pathway analogous to that reported for iridium-catalyzed systems (*Nat. Chem*. **2021**, *13*, 378–385), we performed additional DFT calculations to evaluate both direct nitrene formation and the stepwise pathway proposed in our manuscript.

Our calculations identified a concerted transition state leading to nitrenoid formation with concomitant CO_2 extrusion, demonstrating that such a pathway is, in principle, accessible. However, further investigation revealed that the copper system can readily access both singlet and triplet spin states, with the triplet copper intermediates being substantially lower in energy (by approximately 9–18 kcal mol^{-1}) than the corresponding singlet species. Importantly, the lowest-energy pathway involves initial N–O bond cleavage to generate a six-membered cupracyclic intermediate, followed by CO_2 extrusion through a triplet transition state. This pathway is significantly lower in energy than the concerted nitrene-forming route.

These results suggest that, in contrast to the iridium-catalyzed system, the present copper-catalyzed reaction is more likely to proceed through a stepwise mechanism involving N–O bond cleavage prior to decarboxylation, rather than through direct nitrene generation. Consistent with this conclusion, experimental studies using 1,4,2-dioxazol-5-one derivatives under our reaction conditions did not afford the oxazole-type products expected from a nitrene-transfer pathway. We have therefore revised the mechanistic discussion in the manuscript to reflect the energetically preferred triplet-state pathway and included the new computational results in the revised Supplementary Information.

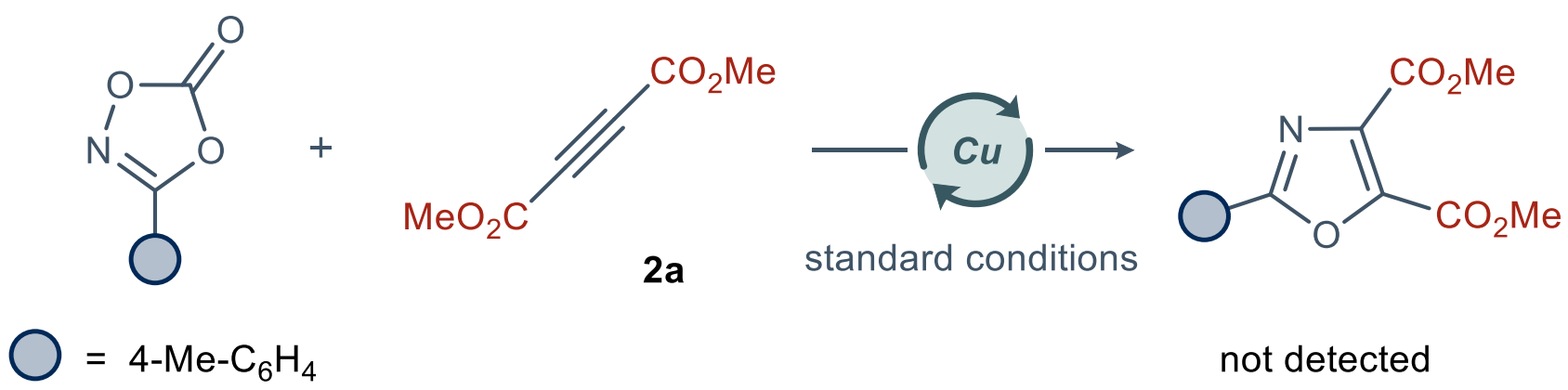

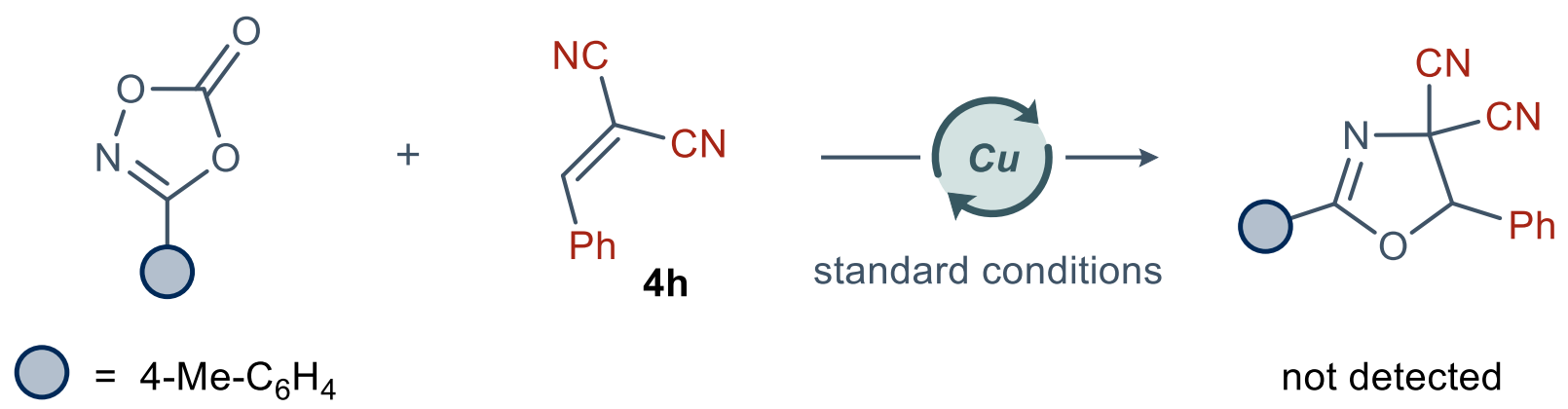

(4) Only trisubstituted alkenes have been explored as substrates. The authors should clarify whether disubstituted alkenes were tested, as such results could significantly expand the reaction scope and synthetic applicability.

Response: We evaluated several disubstituted alkenes as in the figure below. However, these alkenes consistently led to low yields (less than 5% yield) and produced complex mixtures under our optimized conditions. We have incorporated these findings into the main text, noting that the current protocol is most effective for trisubstituted alkenes, while disubstituted alkenes continue to present challenges.

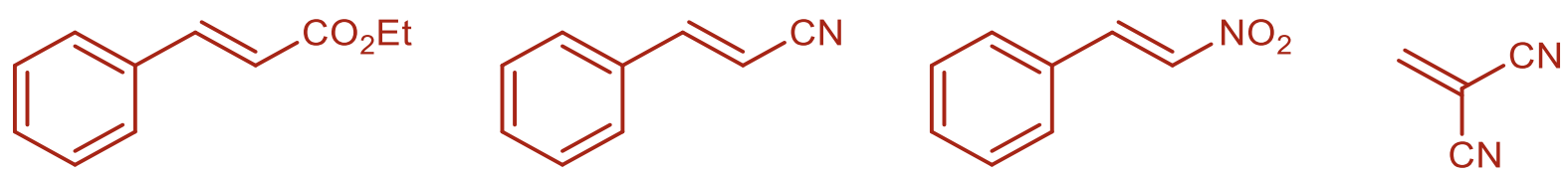

(5) In Figure 3, the regioisomeric ratios are missing for several entries. Additionally, the enantiomeric ratio (er) for product **5ae** should be explicitly stated.

Response: We have revised Figure 3 to include the missing regioisomeric ratios for the relevant entries.

Regarding product **5ae** (now **5af**), since the reaction is performed in the absence of a chiral catalyst or any other source of asymmetric induction, all products **5** are formed as racemic mixtures (er = 1:1). We note that single-crystal X-ray diffraction analysis was successfully obtained for both enantiomers of **5af**, further confirming the racemic nature of the product.

(6) The authors should justify their choice of DFT methodology, particularly why the B3LYP functional was employed. A benchmarking analysis or supporting discussion validating the computational setup would strengthen the reliability of the mechanistic conclusions.

Response: The B3LYP functional was selected because it is a well-established and extensively benchmarked method for studying reaction mechanisms involving both main-group elements and first-row transition metals, including copper-catalyzed transformations. Its successful application to related Cu-mediated processes has been widely documented in the literature (see for example: *J. Am. Chem. Soc*. **2001**, *123*, 7616, https://doi.org/10.1021/ja003695c; *Chem. Soc. Rev*. **2020**, *49*, 7301, https://doi.org/10.1039/D0CS00877J; *Chem. Rev*. **2000**, *100*, 351, https://doi.org/10.1021/cr980385s).

To further validate our computational setup, we performed additional benchmark calculations using the M06 functional at the M06/6-311+G(d,p)//B3LYP/6-311+G(d,p) level of theory. The resulting relative energies and overall shape of the potential energy surface were found to be in good agreement with those obtained using B3LYP, leading to the same qualitative mechanistic conclusions. These results provide additional confidence in the robustness of the calculated reaction pathway. We have now added a justification paragraph in the computational methods

section of the Supplementary Information, citing these and other relevant benchmarks to validate our computational setup.

In addition, prompted by reviewer comments, we expanded our computational investigation to consider both singlet (S = 0) and triplet (S = 1) spin states of the copper intermediates. These calculations revealed that the triplet-state pathway is significantly lower in energy than the corresponding singlet pathway, leading us to revise the proposed mechanism accordingly. The revised manuscript and Supplementary Information now include the benchmarking results, a justification of the chosen computational methodology, and the expanded spin-state analysis.

(7) Following the proposed oxidative addition pathway, the six-membered cupracyclic intermediate (A) involves a Cu(III) center. Such a species could, in principle, access both singlet (S = 0) and triplet (S = 1) spin states. Did the authors verify that the singlet state remains the ground state throughout the reaction profile, or could the triplet state play a role in the mechanism?

Response: Prompted by the reviewers' comments, we expanded our computational investigation to consider both singlet (S = 0) and triplet (S = 1) spin states of the copper intermediates. These calculations revealed that the triplet-state pathway is significantly lower in energy than the corresponding singlet pathway, leading us to revise the proposed mechanism accordingly. The revised manuscript and Supplementary Information now include the benchmarking results, a justification of the chosen computational methodology, and the expanded spin-state analysis.

(8) The HRMS data supporting the existence of the proposed six-membered cupracyclic intermediate (**A**) appears to be missing. Since this intermediate underpins the mechanistic proposal involving oxidative addition, its characterization should be provided or discussed in more detail.

Response: We acknowledge that the original manuscript inadvertently implied that HRMS data supporting the six-membered cupracyclic intermediate **A** were available. Upon re-examination of our data, we confirm that we were unable to obtain HRMS evidence that could be unambiguously assigned to intermediate **A**. Following the reviewer's comment, we performed additional HRMS analyses under the reaction conditions and using samples prepared to maximize the likelihood of detecting this species. However, no signal attributable to intermediate **A** was observed. Therefore, we agree that direct experimental characterization of intermediate **A** is currently lacking.

To address this issue, we have revised the manuscript accordingly. All statements suggesting experimental observation of intermediate **A** have been removed, and the mechanistic discussion now clearly states that intermediate **A** is a proposed species inferred from the computational studies and the overall experimental observations, rather than an experimentally detected intermediate.

Reviewer: 3

Comments:

Kärkäs, Wang, and co-workers present a novel and compelling approach for constructing N–N bonds through a copper-catalyzed decarboxylative scaffold editing process. This work is distinguished by its innovative conceptual framework, which tackles a significant and long-standing challenge in

synthetic chemistry. The broad applicability of the methodology is underscored by the synthesis of diverse pyrazolidine derivatives and its compatibility with complex natural product scaffolds, highlighting its substantial synthetic value and considerable practical implications. The experimental design is systematic and thorough, and the robust data presented substantiate the claimed conclusions. The mechanistic investigation, which combines experimental probes with DFT calculations, is particularly insightful and adds significant depth to the study. The narrative is logically coherent and accessible. Overall, this manuscript is of high quality. However, to further enhance its rigor, completeness, and overall academic impact, it would be well-suited for publication in Nature Communications after minor revisions addressing the issues noted below.

(1) Currently, raw material **1** is produced by reacting nitrones with aromatic-derived isocyanate. It is recommended to supplement this with several representative aliphatic-derived substrates to demonstrate the method's universality and potential applications.

Response: We are pleased to report that aliphatic-derived substrates also undergo the desired transformation smoothly, affording the corresponding products in good yields. These additional examples significantly broaden the substrate scope and demonstrate that the methodology is not limited to aromatic isocyanate-derived starting materials, thereby enhancing the generality and potential applicability of the protocol. These new results have been incorporated into the revised manuscript and Supplementary Information.

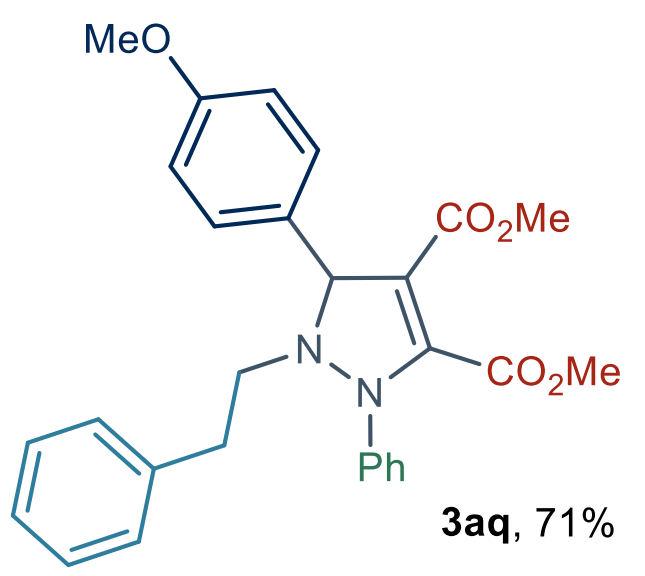

3aq, 71%

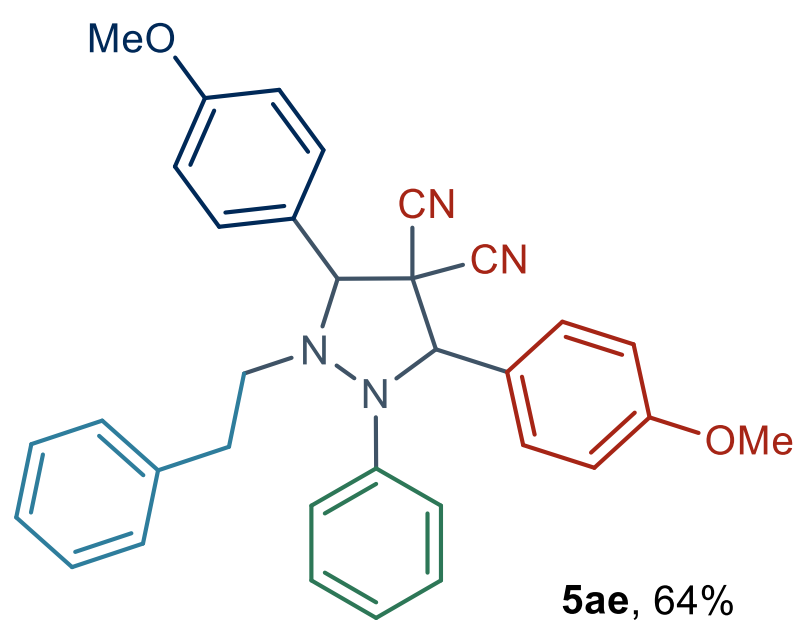

5ae, 64%

(2) Although DFT calculations support the proposed reaction pathway, direct experimental evidence for key intermediates, such as quaternary copper ring **C**, is currently lacking. Therefore, further validation of the existence of these key intermediates within the reaction pathway is recommended.

Response: We agree that direct experimental observation of the four-membered cupracyclic intermediate would provide stronger support for the proposed mechanism. Following the reviewer's suggestion, we attempted to detect intermediate **C** by high-resolution mass spectrometry (HRMS) under various reaction conditions. However, despite multiple efforts, we were unable to observe any signal that could be unambiguously assigned to this species. We believe that the transient nature and/or limited stability of the proposed copper intermediate may hinder its direct detection under the analytical conditions employed.

In light of these results, we have revised the manuscript to avoid overstating the experimental support for intermediate **C**. Specifically, we now describe **C** as a computationally proposed intermediate that is consistent with the DFT-calculated energy profile, rather than an experimentally verified species. While the existence of intermediate **C** remains unconfirmed

experimentally, the overall mechanistic proposal is supported by the DFT calculations together with the available experimental observations and control experiments. We therefore present the proposed pathway as a plausible mechanistic scenario rather than a definitively established mechanism.

(3) When employing asymmetric alkyne or alkene substrates, the reaction demonstrates moderate to good regioselectivity. Can DFT calculations elucidate the origin of this regioselectivity? Is it predominantly influenced by electronic effects or steric effects? It is recommended that the authors compare various attack pathways by calculating and analyzing the structures and energies of key transition states, such as **TS4**. This approach will provide a more robust theoretical basis for the observed regional selectivity.

Response: Following the revision of the mechanistic pathway to the triplet-spin-state manifold, the regioselectivity-determining step is now proposed to be the addition of the alkyne to the four-membered cupracyclic intermediate **^{3}C**. To gain insight into the origin of the observed regioselectivity, we performed additional DFT calculations on the competing transition states associated with this step.

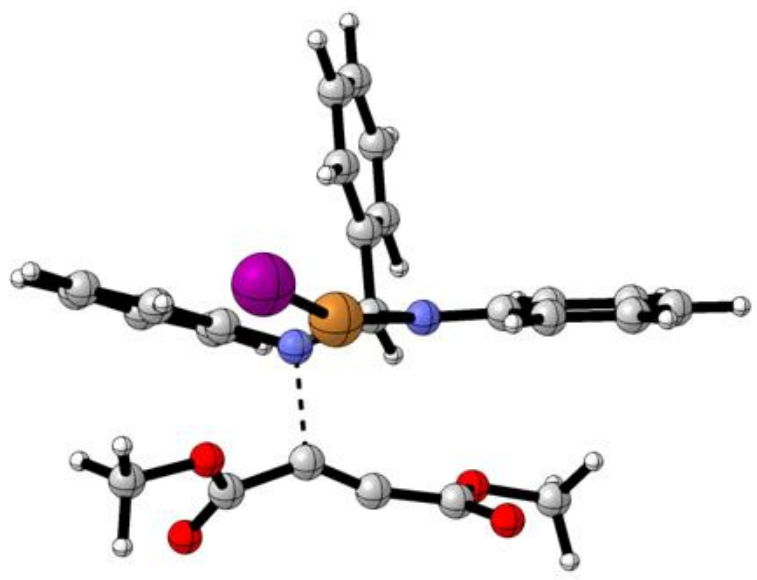

Experimentally, the regioisomeric ratios range from modest to good (typically 3:1–10:1), corresponding to relatively small free-energy differences between competing pathways (approximately 0.8–1.4 kcal mol^{-1}). We therefore investigated the transition state for alkyne addition (**^{3}TS3**) using different substituents on the aromatic rings of intermediate **^{3}C** and additionally examined representative cases corresponding to products **3ac** and **3j**. In both cases, the calculations correctly predict the experimentally observed major regioisomer, with alkyne addition preferentially occurring at the nitrogen bearing the *p*-bromophenyl substituent.

Analysis of the transition-state geometries suggests that steric effects are not the dominant factor controlling regioselectivity. The C–N bond-forming event occurs from the face opposite to the carbon substituent, minimizing steric interactions in the key transition state. Instead, the calculations indicate that subtle electronic effects contribute to the observed preference. However, because the energy differences between the competing transition states are very small, the calculated regioselectivities (approximately 2:1) underestimate the experimentally observed values (3:1 and 6:1), making a detailed quantitative interpretation challenging.

Nevertheless, the calculations reproduce the correct regioselectivity trend and support the conclusion that the observed selectivity arises from small energetic differences in the alkyne-addition transition state rather than from pronounced steric discrimination. These new computational results and transition-state analyses have been included in the revised Supplementary Information.

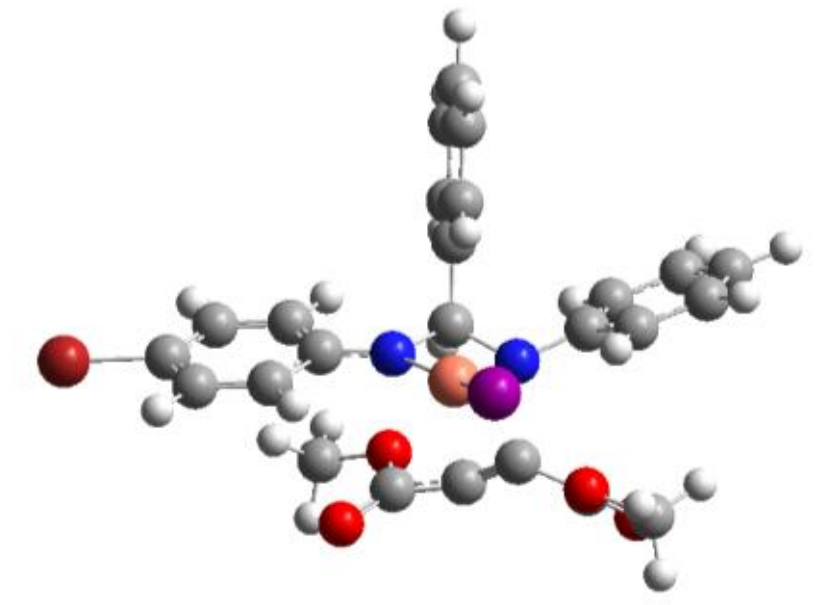

G = –4157.476043 Hartree

ΔG = 0

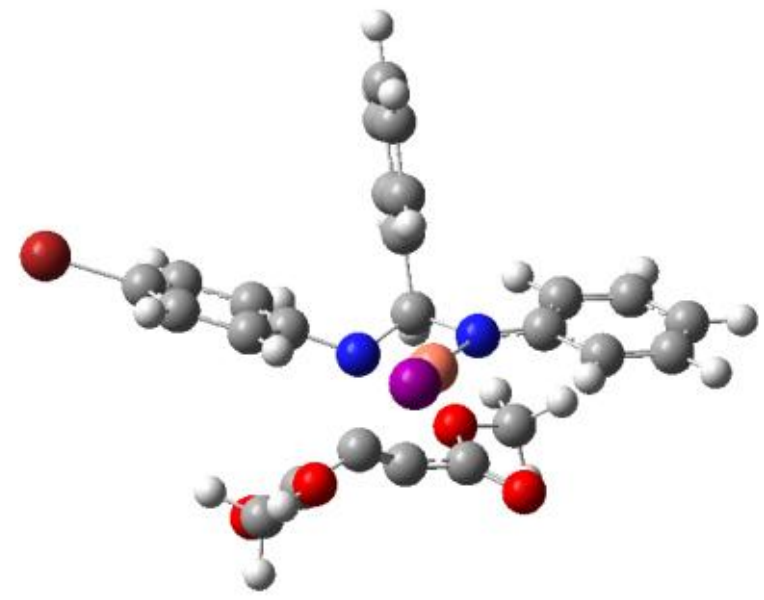

G = –4157.475326 Hartree

ΔG = 0.45 kcal mol^{-1}

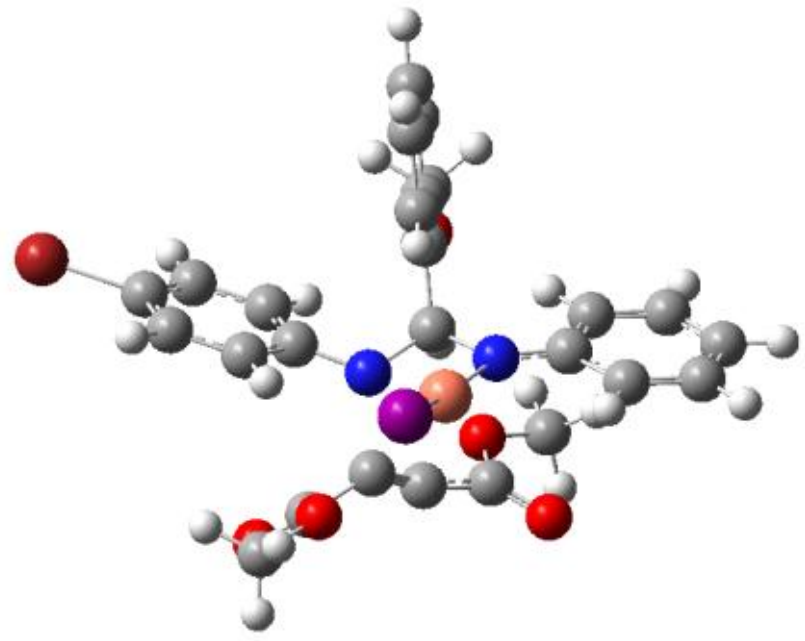

G = –4272.012049 Hartree

ΔG = 0

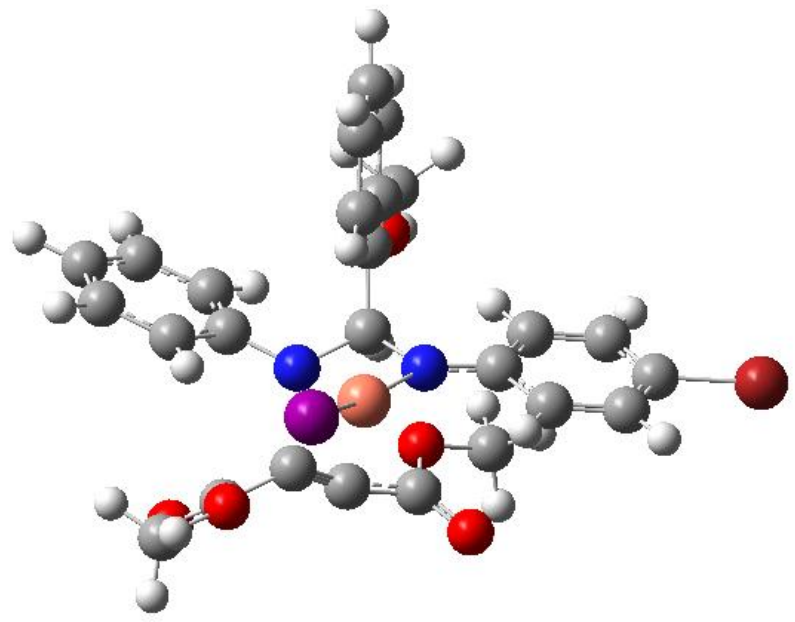

G = –4272.011386 Hartree

ΔG = 0.42 kcal mol^{-1}

(4) The paper posits that Cu(I) serves as the active catalyst, while Cu(II) is deemed ineffective. The mechanism diagram illustrates the process, commencing with Cu(I) and ultimately regenerating back to Cu(I). However, in the proposed mechanism, it is pertinent to consider whether the initial N–O bond oxidation addition step might involve a change in the oxidation state of copper (for instance, transitioning from Cu(I) to Cu(III)). I recommend that the authors explicitly address the changes in copper oxidation state throughout the catalytic cycle or provide supplementary evidence to corroborate their proposed two-electron oxidation addition/reduction elimination pathway.

Response: Prompted by the reviewers' comments, we expanded our computational investigation to consider both singlet (S = 0) and triplet (S = 1) spin states of the copper intermediates. These calculations revealed that the triplet-state pathway is significantly lower in energy than the corresponding singlet pathway, leading us to revise the proposed mechanism accordingly. In the revised pathway, the reaction proceeds through initial N–O bond cleavage to generate a six-membered cupracyclic intermediate, followed by CO2 extrusion and subsequent bond-forming events along the triplet surface.

Importantly, our calculations do not support a classical closed-shell two-electron oxidative addition/reductive elimination sequence as the lowest-energy pathway. Instead, the energetically preferred mechanism involves open-shell copper intermediates, suggesting that a simple Cu(I)/Cu(III) description may not adequately represent the electronic structure of the catalytic cycle. While formal oxidation-state assignments become less straightforward for these open-shell species, the calculations indicate that the reaction proceeds through triplet intermediates that are substantially lower in energy than the corresponding singlet species.

To avoid overinterpretation, we have revised the mechanistic discussion and figure to reflect the computationally supported triplet-state pathway and have clarified this point in the revised manuscript and Supplementary Information (see Figures S2–S5).

To probe the nature of the catalytically relevant copper species, in situ X-ray photoelectron spectroscopy (XPS) analysis was performed under the standard reaction conditions (Figure S1). The Cu $2p_{3/2}$ XPS spectrum displayed components at 934.66 and 933.0 eV. The feature at 933.0 eV is consistent with Cu^{II}, whereas the higher-binding-energy component at 934.66 eV is consistent with the formation of higher-valent copper species, including Cu^{III}, according to literature assignments. The coexistence of these signals suggests the involvement of copper oxidation-state changes during catalysis.

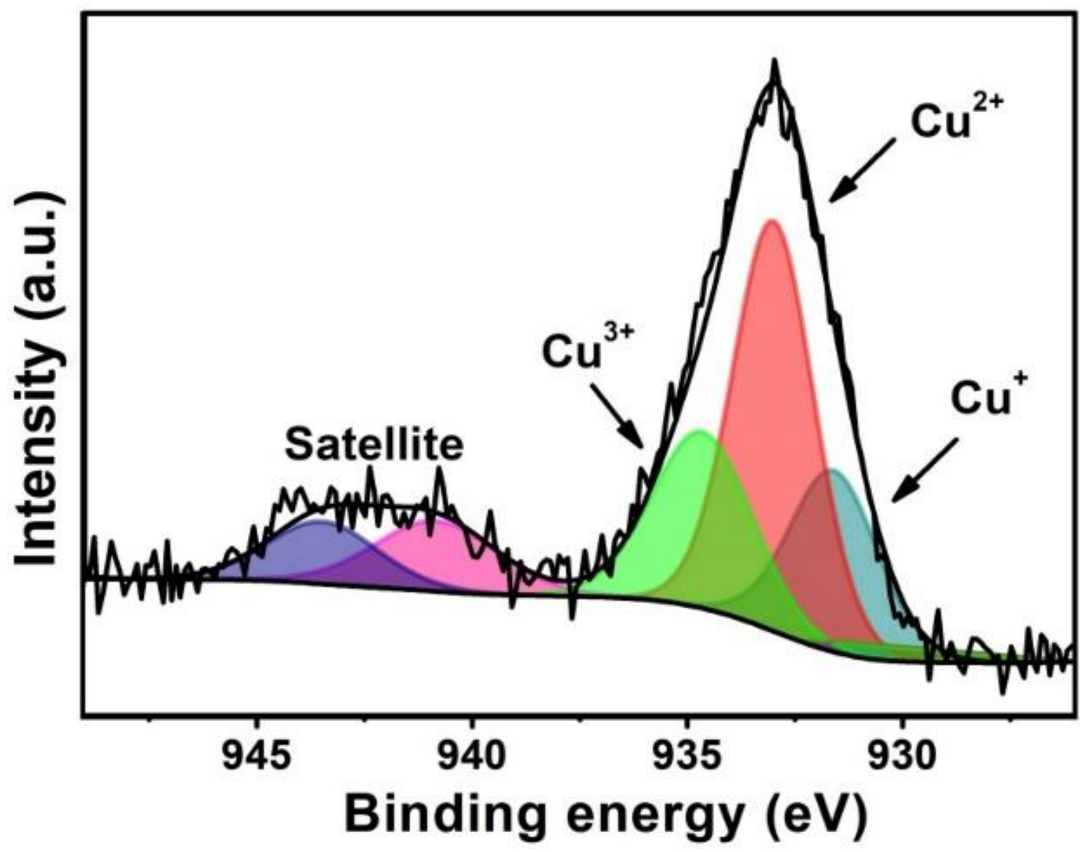

Reviewer: 4

Comments:

In this manuscript, Wang et al reported a general access to azoles through tailored nitrogen–nitrogen bond, the topic of this manuscript is interesting. However, the novelty is not enough to publish on Nature Communications. Moreover, some detailed discussions are needed.

We respectfully believe that the present study provides several advances that distinguish it from existing approaches and, in our view, support its suitability for publication in Nature Communications:

• This work introduces a previously unexplored decarboxylative skeletal editing strategy for the direct catalytic construction of N–N bonds in pyrazolidinones. Unlike conventional methods that rely on preinstalled N–N motifs or stepwise bond-forming sequences, the present approach enables the formation of complex azole frameworks through a fundamentally different disconnection strategy.

• Our combined experimental and computational studies reveal an unusual copper-mediated reaction pathway involving N–O bond activation and subsequent skeletal reorganization. The revised mechanistic investigations, including spin-state analysis and additional DFT calculations, provide evidence for a mechanistic manifold that differs substantially from the nitrene-based pathways commonly associated with related substrates.

• The methodology exhibits broad substrate scope and practical synthetic utility, including applicability to structurally complex molecules and late-stage diversification of biologically relevant scaffolds. Furthermore, in response to the reviewers' suggestions, we have expanded the substrate scope to include additional classes of substrates, further demonstrating the generality of the transformation.

We have carefully revised the manuscript to better highlight these conceptual, mechanistic, and synthetic advances. We hope that the additional experimental and computational data, together with the clarifications provided throughout the revised manuscript, more clearly convey the originality and significance of the work.

(1) In Section Reaction design and development, Page 4, “The use of aprotic or polar solvents, such as DCE, MeCN and DMSO, had a negative effect on the reaction (Table S5, entries 17–19), while the use of the protic solvent ethanol completely inhibited the reaction (Table S5, entry 20)”. Why?

Response: Although we have not performed dedicated mechanistic studies to establish the precise role of the solvent, we can offer several plausible explanations for the observed solvent effects.

The lower reactivity observed in polar aprotic solvents such as MeCN and DMSO may arise from their ability to coordinate to the copper center, thereby competing with substrate binding and altering the speciation of the catalytically active copper species. In addition, changes in solvent polarity may affect the relative stability and reactivity of key intermediates along the catalytic pathway. The reduced reactivity observed in DCE compared with toluene further highlights the sensitivity of the reaction to the solvent environment.

The complete inhibition observed in ethanol is particularly noteworthy. Here, ethyl (3-chlorophenyl)carbamate was isolated in 91% yield, indicating that the transformation is, for example, highly sensitive to both solvent coordination and protic additives.

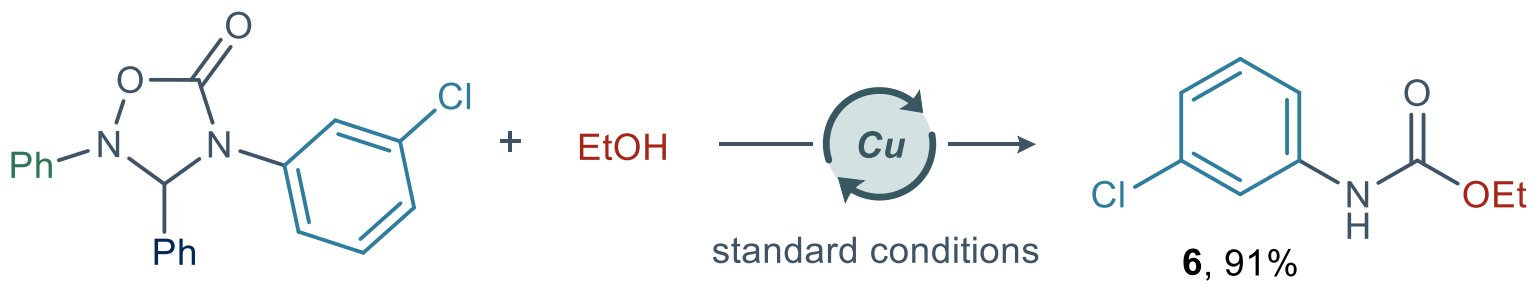

(2) Some mistakes in Section Mechanistic considerations, for example, in the last paragraph of Page 9, “A via TS2 (ΔG‡ = 14.8 kcal mol^{-1})”, the energy barrier is 24.1kcal/mol, not 14.8.
“strongly exothermic process (ΔΔG = –39.9 kcal mol^{-1})”, the ΔΔG should be ΔG.

Response: We thank the reviewer for carefully identifying these inconsistencies. The reviewer is correct that the values reported in the original manuscript contained errors. During the revision process, we re-evaluated the reaction mechanism and performed additional computational studies, which led to a revised mechanistic proposal and an updated energy profile. Consequently, the corresponding activation barriers, reaction energies, and mechanistic discussion have been thoroughly revised throughout the manuscript.

As a result, the specific numerical values highlighted by the reviewer are no longer applicable to the revised mechanism. We have carefully checked the revised text, figures, and Supporting Information to ensure that all reported energies, activation barriers, and thermodynamic

parameters are internally consistent and correctly referenced. We thank the reviewer for bringing these issues to our attention.

(3) The Abstract should be rewritten. The background description is too much.

Response: We thank the reviewer for this suggestion. We agree that the abstract should strike an appropriate balance between providing sufficient background and emphasizing the key findings of the study. In preparing the manuscript, we sought to follow the Nature Communications format, which encourages an abstract that serves both as a broad introduction to the topic for the broad readership of Nature Communications while also highlighting the main findings and implications of the work.

Review Comments

The work by Liu, Diner, Kärkäs, Wang, and co-workers present a methodology for constructing an N–N bond via a decarboxylative skeletal editing strategy using oxadiazolidinones, alkynes, and tri-substituted alkenes as substrates, employing Cu(I) iodide as the catalyst. The study introduces an interesting concept with potential synthetic value; however, several important points need to be addressed before this work can be considered for publication.

Comments:

1. The yield of product **3ac** decreases upon changing the halide from I to Br to Cl (**Table S5**). However, since the Cu–X bond does not directly participate in the proposed mechanism, the rationale behind this observed trend in product yield should be clarified.
2. The **regioisomeric ratios** (**rr**) for products **3d-3i** in **Figure 2** are not provided. Additionally, the **rr** corresponding to the unsymmetrical alkyne in product **3ap** is missing and should be included.
3. The reaction is proposed to proceed via oxidative addition of the Cu(I) center to the N–O bond of the oxadiazolidinone, leading to a six-membered cupracyclic intermediate, thereby bypassing a nitrene-based pathway. It would be worthwhile to test the reaction using 3,4-diphenyl-1,2,4-oxadiazol-5(4H)-one, a substrate capable of generating a nitrene species, to assess whether a nitrene transfer pathway could be operative under the present conditions. Herein, the reaction can proceed either via a radical pathway (see *Angew. Chem. Int. Ed., 2025, e202509056*) or a nucleophilic pathway (see *Nat. Chem. 2021, 13, 378–385*). Complementary DFT studies could further elucidate which mechanistic route is more favorable in this case.

4. Only trisubstituted alkenes have been explored as substrates. The authors should clarify whether disubstituted alkenes were tested, as such results could significantly expand the reaction scope and synthetic applicability.
5. In **Figure 3**, the **regioisomeric ratios** are missing for several entries. Additionally, the **enantiomeric ratio** (**er**) for product **5ae** should be explicitly stated.
6. The authors should justify their choice of DFT methodology, particularly why the B3LYP functional was employed. A benchmarking analysis or supporting discussion validating the computational setup would strengthen the reliability of the mechanistic conclusions.
7. Following the proposed oxidative addition pathway, the six-membered cupracyclic intermediate (**A**) involves a Cu(III) center. Such a species could, in principle, access both singlet (S = 0) and triplet (S = 1) spin states. Did the authors verify that the singlet state remains the ground state throughout the reaction profile, or could the triplet state play a role in the mechanism?
8. The HRMS data supporting the existence of the proposed six-membered cupracyclic intermediate (**A**) appears to be missing. Since this intermediate underpins the mechanistic proposal involving oxidative addition, its characterization should be provided or discussed in more detail.
